# Mothers’ knowledge and practices on breastfeeding and complementary feeding in an urban slum area and rural area in Kenya: A cross-sectional interview study

**DOI:** 10.1177/13674935221083451

**Published:** 2022-04-15

**Authors:** Kerttu Uusimäki, Lauriina Schneider, Crippina Lubeka, Judith Kimiwye, Marja Mutanen

**Affiliations:** 1 Department of Food and Nutrition, Faculty of Agriculture and Forestry, 98742 University of Helsinki, Helsinki, Finland; 2 Department of Food, Nutrition and Dietetics, School of Applied Human Sciences, 361757 Kenyatta University, Nairobi, Kenya

**Keywords:** breast feeding, complementary feeding, health knowledge, infant health, practice

## Abstract

Maternal breastfeeding and complementary feeding knowledge is an important determinant of childrens’ long-term health and development. This study aims to account for associations between knowledge and practices in Kenya and report the food consumption of children from birth to 18 months. In 2015 mother–child pairs were recruited from Mother-and-Child Health Centers; 415 in an urban slum in Nairobi and 364 in rural Machakos. Knowledge and practice scores were calculated from questionnaire variables and 24-h food frequency questionnaire. The associations of knowledge and practices were studied with regression analysis. Breastfeeding knowledge (Nairobi 6.3/9, Machakos 5.9/9) and practices (Nairobi 7.5/8, Machakos 7.2/8) were good in both areas. Complementary feeding knowledge was not as good (Nairobi 7.5/14, Machakos 7.1/14). Minimum meal frequency was reached by almost 80% of the children but dietary diversity was low (Nairobi 2.7 [SD 1.4], Machakos 2.4. [SD 1.3]). Only 27% of children in Nairobi and 13% in Machakos were fed a minimum acceptable diet. The complementary feeding knowledge score was associated only with minimum dietary diversity in Nairobi (OR: 1.29; 95% CI: 1.105–1.514). Infant and young child feeding knowledge and practices were on a similar level in both areas. Future interventions should focus on improving dietary diversity.

## Introduction

Poor nutrition during infancy is associated with negative outcomes in children’s growth, cognitive development, morbidity in later life, and overall economic productivity during adulthood (Neovita 2016). Mothers in developing countries feed their children in greatly varying communities and societal conditions which can either impede or enable good infant and young child feeding (IYCF) practises and therefore child nutrition (Stewart et al., 2013).

Demographic Health Surveys (DHS) from 49 low- and middle-income countries show that dietary quality is systematically lower in rural than urban environments (Baye and Kennedy 2020). Community and societal conditions also differ between rural and urban areas (Egede et al., 2017). Traditional diet patterns with low dietary diversity have been linked to poor growth (Tanaka et al., 2019). Factors that impair a mother’s ability to follow recommendations include the patriarchal system and misinformation about child-care and feeding among family members, traditional birth attendants, and health workers (Legesse et al., 2015; Mchome et al., 2020). Harmful feeding practices are sustained by social norms and cultural beliefs, especially in rural areas (Chakona 2020; Cislaghi et al., 2019). However, a rural lifestyle often means that mothers do not need to partake in work outside the home and thus do not need to leave their children and can breastfeed more often (Dede and Bras 2020). Women’s empowerment (Ickes et al., 2018), family support (Chakona 2020), and own production of food in a rural area may increase the chances of good child nutrition (Kuche et al., 2020). For example, household production of fruit and vegetables was associated with increased dietary diversity and children’s growth in Ethiopia (Kuche et al., 2020). In addition, the influence of social norms may also be positive and contribute to good IYCF practices (Nguyen et al., 2019).

Mothers living in urban environments face different challenges than mothers in rural areas: food needs to be bought, more processed food products are used and purchasing food from street vendors is common (Steyn et al., 2011). Mothers usually lack maternity leave and must leave children, even newborns, to go to work outside the home (Gebermariam et al., 2020). Global trends in food consumption and peer pressure may drive mothers to act against recommendations (Berhane et al., 2018). Mothers may desire to please key members of their social network to avoid negative sanctions, as seen in Congo, where refusal to give a baby water during the first 6 months was seen as socially unacceptable (Wood et al., 2020). Additionally, while access to information from the internet can be beneficial, media literacy may be poor (Kuyinu et al., 2020). Finally, in terms of health and nutrition for young children, slums have proven worse than rural and non-slum urban areas (Mberu et al., 2016). Hindering slum children’s good nutritional status are poor hygiene, diarrhea and other diseases, among other factors (Mberu et al., 2016). In their recent systematic review of urban–poor settings in low- and middle-income countries, Mutisya et al. (2021) highlight the need for situational analysis in urban poor areas and more evidence from these areas to better understand IYCF practices and their facilitators.

The 2014 DHS report from Kenya (Kenya National Bureau of Statistics et al., 2015) presents some child-feeding indicators for rural and urban contexts. According to these indicators, the initiation of breastfeeding is on a good level in both contexts, although on a slightly higher level in urban areas as reflected in the low pre-lacteal feeding percentages (12.4% urban and 17.2% rural). Exclusive breastfeeding lasts on average 3.7 months in urban and 3.0 months in rural areas. Complementary feeding indicators are constantly lower in rural than urban areas (Kenya National Bureau of Statistics et al., 2015). In general, these results from Kenya correspond to results from 32 other countries in Sub-Saharan Africa (SSA), which show that most breastfeeding-related indicators are at an acceptable level, while complementary feeding indicators are generally low (Gebremedhin 2019). DHS-level information is insufficient to guide the implementation of targeted nutrition programs, and conditions can vary considerably within a country (Harvey et al., 2017).

## Aim

### The aims of the study were

1. To assess the dietary diversity and meal frequency of infants and young children from birth to 18 months in an urban slum area in Nairobi and in a rural area in Machakos, Kenya.
2. To account for associations between maternal knowledge and practices in the study areas.

## Methods

### Study design and population

A cross-sectional interview study design facilitated collection of data concerning socio-demographics, mothers’ knowledge and practices on breastfeeding and complementary feeding, and food intake of children from birth to 18 months. The study was conducted in September 2015 in both a typical urban slum settlement in Ruaraka, Nairobi, and a rural setting in Masinga, Machakos County. Using convenience sampling, area health officers gathered the study population from public Mother-and-Child Health Centers (MCHCs), six in Nairobi and six in Machakos. The mother–child pairs were selected from mothers attending the health center on the day of the study with younger than 18-month-old children.

### Data collection

A 12-member team of Kenyan nutritionists and nutrition students from Kenyatta University, who spoke the local languages, conducted the interviews. All the interviews were performed by the whole team at one time in the same MCHC before moving to the next MCHC. Participants filled in an informed consent form after the interviewer explained the purpose of the study. The interview involved a mobile device-based questionnaire, with both closed and open-ended questions. Interviewers were trained beforehand on how to fill in the forms. Each interview lasted approximately 30–40 min.

The questionnaire was modified from the Kenyan validated Knowledge, Attitudes and Practices (KAP) survey form (Ministry of Health Kenya 2014). The questionnaire provides data and insights on the practices and knowledge on breastfeeding and complementary feeding. A food frequency questionnaire was added to the original questionnaire to record the variety of food children had consumed during the previous 24 h. The questionnaire was prepared in English and translated into Kiswahili for data collection.

### Knowledge and practice indicators

**Figure 1.**
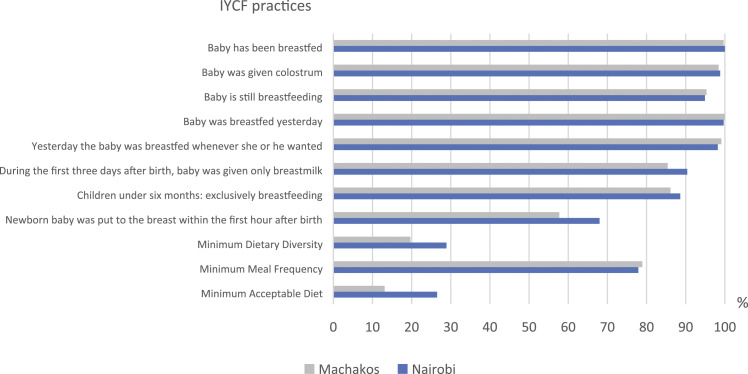
Proportion of mother–child pairs who reported to have acted upon infant and young child feeding (IYCF) recommendations, Nairobi and Machakos, Kenya.

Knowledge and practice scores derived from selected questionnaire variables (Figure 1 and Table 3) were used as indicators for breastfeeding knowledge and practice and complementary feeding knowledge. These indicators were calculated using equally weighted summative variable scores. Incorrect or uncertain (do not know) responses were scored as zero. For multiple answer possibilities, one or more correct answers were counted as a total score of one. Weights were not used, as the selected variables were considered equally important. The breastfeeding practice score ranged from 0 to eight for children younger than 6 months and from 0 to seven for older children. The maximum score was nine for breastfeeding knowledge and 14 for complementary feeding knowledge.

Evaluation of complementary feeding practices involved asking mothers to describe everything their child had eaten during the previous day and night. Food eaten during breakfast, lunch, dinner, and as snacks was recorded separately. World Health Organization (WHO) and UNICEF categories were used to calculate dietary diversity by categorizing foods into the following seven food groups: grains, roots, tubers; legumes and nuts; dairy products; meat (meat, fish, poultry, snails, liver/organ meats); eggs; vitamin A–rich fruits and vegetables; and other fruits and vegetables (WHO 2010). Dietary diversity score (DDS) was calculated for each child as the number of food groups from which the child had reportedly consumed foods for at least one spoonful of, during the previous day. Minimum dietary diversity (MDD) was determined as the proportion of children who consumed foods from at least four food groups. Meal frequency (MF) was calculated as the number of meals and snacks eaten during the previous day. Minimum meal frequency (MMF) is further defined as the proportion of children who had consumed complementary foods at least the minimum number of times during the previous day. This calculation used the WHO definition of minimum meal frequency: two times for breastfed infants aged six to 8 months, three times for breastfed children aged nine to 23 months, and four times for non-breastfed children aged six to 23 months. Minimum acceptable diet (MAD) is defined as the proportion of the children who reached both MDD and MMF during the previous day (WHO 2008).

### Data analyses

Data were cleaned, coded, and analyzed using IBM SPSS statistics software version 26.0 for Windows (IBM Corp, Armonk, NY). The distributions of variables were assessed with frequency tables and histograms and normality was checked by visual inspection. Continuous variables were presented as medians and interquartile ranges. The distributions of practice and knowledge scores were roughly normally distributed or skewed to the left, but as all the distributions were tightly around the mean and median, they were presented as means and standard deviations. Categorical variables were presented as percentages. Complementary feeding indicators involved assessing DDS, MDD, MF, MMF, and MAD. Data from Nairobi and Machakos were analyzed separately.

Univariate binary logistic regression was used to determine the variables to include in the multivariable model. The *p*-value of 0.2 of a Wald test was used as a cut-off point for inclusion, and thus number of children and adults in the household, and gender of a child in Nairobi; and mother’s age, marital status, and education level in Machakos were added to multivariable model. Multivariable logistic regression was used to study the association between the probability of child getting MDD and MMF, based on knowledge score and other previously assessed predictor variables. Multicollinearity was tested using the variance inflator factor (VIF). For the final model, the statistical significance was set at *p* < 0.05.

### Ethical considerations

The study applied the national and international ethical guidelines for biomedical research involving human subjects. The AMREF ethical committee in Nairobi approved the study (ESRC P111/2014). Permission to conduct the study was sought from the Ministry of Health, Kenya and facility health administrators. The study subjects gave their informed written consent.

## Results

### Socio-demographic characteristics

A total of 780 participants completed the questionnaire. After excluding one participant who reported that the mother of the child had passed away, the final sample comprised 415 mother–child pairs in Nairobi and 364 mother–child pairs in Machakos. Half of the children in the Nairobi sample, and 40% in Machakos, were under 6 months of age (Table 1).

**Table 1.** Household-, mother-, and child characteristics.

	Nairobi, *n* = 415				Machakos, *n* = 364			
	Median	(IQR)	*n*	(%)	Median	(IQR)	*n*	(%)
Age of mother, years	25	(7)	-	-	26	(9)	—	—
Age group of a child								
From birth to 5 months			211	(50.8)	151			(41.5)
6–8 months			58	(14.0)	58			(15.9)
9–11 months			65	(15.7)	68			(18.7)
12–18 months			81	(19.5)	87			(23.9)
Number of adults in the household									
1			11	(2.7)	18			(4.9)
2			329	(79.3)	194			(53.3)
3			50	(12.0)	71			(19.5)
4≤			25	(6.0)	81			(22.3)
Number of children in the household								
1			167	(40.2)	79			(21.7)
2			127	(30.6)	100			(27.5)
3			68	(16.4)	97			(26.6)
4			28	(6.7)	39			(10.7)
5≤			25	(6.0)	50			(13.7)
Marital status								
Married			362	(87.2)	292			(80.2)
Living together			2	(0.5)	0			(0)
Divorced, separated			6	(1.4)	12			(3.3)
Widowed			4	(1.0)	3			(0.8)
Single, never married			41	(9.9)	57			(15.7)
Level of mother’s education								
No education or less than primary			31	(7.5)	36			(9.3)
Primary school			178	(42.9)	211			(58.0)
Secondary/high school			132	(31.8)	88			(24.2)
College, pre-university			73	(17.6)	31			(8.5)
Postgraduate degree			1	(0.2)	0			(0)
Place of child’s birth								
Hospital			357	(86.0)	270			(74.2)
Health center, private clinic			31	(7.5)	14			(3.8)
Home			21	(5.1)	67			(18.4)
Midwife’s home			4	(1)	10			(2.7)
Other			2	(0.5)	3			(0.8)
Mother’s religion								
Christian			406	(97.8)	364			(100)
Other			9	(2.2)	0			(0)

IQR = interquartile range

Half of the households in Machakos and 70% of the households in Nairobi included one or two children (Table 1). In Nairobi, nearly 80% of households included two adults; in Machakos, 20% of households had three adults and 20% had four adults. The median age of mothers was 25 years in Nairobi and 26 years in Machakos. More than 80% of mothers in both areas were married. Half of the mothers in Nairobi had at least secondary school education and the respective percentage was 33 in Machakos.

### Breastfeeding practices

Nearly all children were reported to be breastfed during the time of the study (Figure 1). Over 98% of the children were given colostrum in both areas and 90% in Nairobi and 85% in Machakos were given only breastmilk during the first 3 days after birth. The vast majority (87% in Nairobi and 86% in Machakos) of children under 6 months were exclusively breastfed. Approximately 75% of mothers in both areas reported having acted according to breastfeeding recommendations in at least seven questions. The mean (±SD) breastfeeding practice score was 7.5/8 (0.7) for under 6 month olds in Nairobi and 7.2/8 (1.0) in Machakos. For children aged 6 months and more, the mean breastfeeding practice score was 6.2/7 (1.1) in both areas.

### Dietary diversity and meal frequency

In Nairobi sample, the mean (±SD) dietary diversity score (DDS) for children over 6 months of age was 2.7 (±1.4) and only 29% received a sufficiently varied diet (Figure 1). Similarly in Machakos sample, the mean DDS was 2.4 (±1.3) and 20% met the minimum criteria for dietary diversity. A large proportion of children, 40% in Nairobi and 49% in Machakos, were reported to have received food from only two or fewer food groups, and approximately 10% in Nairobi and 13% in Machakos did not receive any foods (more in Supplementary Table 2). Children ate approximately three times during the previous day in both areas and 78% in Nairobi and 79% in Machakos met the minimum meal frequency recommendation. Approximately 27% in Nairobi sample and 13% in Machakos sample were given a minimum acceptable diet, which is the combination of minimum DDS and minimum MF. Table 2 presents the frequencies of MDD, MMF and MAD by age groups.

**Table 2.** The prevalence of minimum dietary diversity (MDD), minimum meal frequency (MMF) and minimum acceptable diet (MAD) of babies aged 6 months or older in Nairobi and Machakos, in Kenya.

	Nairobi (*n* = 204)						Machakos (*n* = 213)					
	MDD		MMF		MAD		MDD		MMF		MAD	
	*n*	(%)	*n*	(%)	*n*	(%)	*n*	(%)	*n*	(%)	*n*	(%)
Breastfed children												
6–8 months	6	(10.5)	38	(66.7)	6	(10.5)	3	(5.2)	39	(67.2)	3	(5.2)
9–11 months	19	(31.1)	51	(83.6)	18	(29.5)	12	(18.8)	49	(76.6)	12	(18.8)
12–18 months	26	(40.0)	55	(84.6)	23	(35.4)	20	(26.7)	68	(90.7)	20	(26.7)
Non-breastfed children												
6–18 months	8	(38.1)	15	(71.4)	7	(33.3)	7	(43.8)	12	(75.0)	2	(11.8)
Total												
6–18 months	59	(28.9)	159	(77.9)	54	(26.5)	42	(19.7)	168	(78.9)	28	(13.1)

**MDD**: the proportion of children aged 6–24 months who had consumed foods from at least four different food groups during the previous 24 h (WHO, 2008). **MMF**: the proportion of children aged 6–24 months who had the recommended number of meals during the previous 24 h. The minimum is defined as two meals for breastfed infants aged 6–8 months, three meals for breastfed infants aged 9–23 months and four meals for non-breastfed children aged 6–23 months (WHO, 2008). **MAD**: the proportion of children aged 6–24 months who had both MDD and MMF during the previous 24 h (WHO, 2008).

Grains, roots and tubers (87% in Nairobi and 86% in Machakos), vitamin A–rich fruit and vegetables (55% and 49%), milk products (42% and 46%) and other fruit and vegetables (38% in both areas) were the most commonly consumed food groups. Conversely meat, fish, and seafood (23% in Nairobi and 11% in Machakos), legumes (21% and 11%) and eggs (8% and 2%) were less frequently consumed foods. Supplementary Table 3 presents frequencies of foods consumed from the seven food groups in three age categories from 6 months onward.

### Infant and young child feeding knowledge

**Figure 2.**
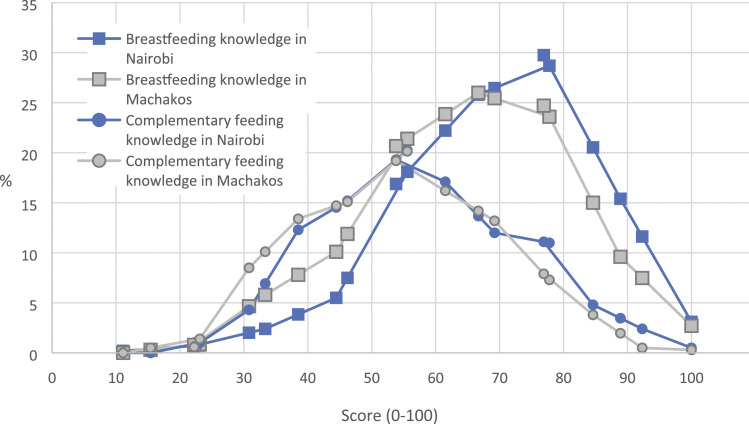
The distributions of mothers’ infant and young child feeding (IYCF) knowledge scores scaled as 0–100 in Nairobi and Machakos.

More than 94% of the mothers in both areas knew that exclusive breastfeeding should be continued for 6 months and that babies should not be given anything else during the first 3 days after birth (Table 3). Benefits of colostrum were less well known. Similarly, the importance of skin-to-skin contact or knowing what to do if a mother felt she had insufficient breastmilk were known by only about 50% of the mothers in both areas. The mean breastfeeding knowledge scores are shown in Table 3 and the distributions in Figure 2. In Nairobi, 75% of the mothers, and in Machakos 60%, achieved a score of at least six out of nine.

**Table 3.** Aspects of breastfeeding and complementary feeding knowledge that was asked in this study and the respective percentages of mothers who answered correctly. Knowledge scores are sums of correct answers.

	Nairobi, *n* = 415		Machakos, *n* = 364	
	***n***	**(%)**	***n***	**(%)**
**Breastfeeding knowledge**				
In the first 3 days, baby should not get anything but breastmilk	407	(98.1)	347	(95.3)
Exclusive breastfeeding should continue for 6 months	403	(97.1)	345	(94.5)
Newborn baby should be given the very first milk	393	(94.7)	334	(91.8)
Baby should be put to breast within an hour of birth	367	(88.4)	280	(76.9)
Breastmilk differs from cow’s milk	361	(87.0)	311	(85.4)
Benefits of colostrum	219	(52.8)	207	(56.9)
Importance of skin-to-skin contact	210	(50.6)	155	(42.6)
What to do when mother feels there is insufficient breastmilk	185	(44.6)	130	(35.7)
When mother is away, under 6 months old baby should be fed breastmilk with a cup	63	(15.2)	40	(11.0)
**Complementary feeding knowledge**				
First food should be introduced at 6 months	318	(76.6)	256	(70.4)
Baby should be given 3–4 meals + snacks after 9 months of age	291	(70.1)	268	(73.6)
It is relevant if a child is short for his/her age	278	(67.0)	254	(69.8)
Thick porridge is better than liquid for babies under 1 year old	247	(59.5)	186	(51.1)
Baby who has started eating should get daily				
...milk products	272	(65.5)	277	(76.1)
...fruits	220	(53.0)	180	(49.5)
...vegetables	144	(34.7)	74	(20.3)
...meat, fish, and/or eggs	41	(9.9)	30	(8.2)
Food groups				
Cereals, roots, and tubers	377	(90.8)	329	(90.4)
Fruit and vegetables	307	(74.0)	244	(67.0)
Meat, fish, and eggs	265	(63.9)	175	(48.1)
Pulses	211	(50.8)	184	(50.5)
Milk and milk products	130	(31.3)	103	(28.3)
Fats	20	(4.8)	27	(7.4)
**IYCF knowledge scores**	Mean	(SD)	Mean	(SD)
Breastfeeding knowledge score (max score 9)	6.3	(1.4)	5.9	(1.5)
Complementary feeding knowledge score (max score 14)	7.5	(2.0)	7.1	(2.0)

SD = standard deviation.

IYCF = Infant and young child feeding.

The proportion of correct answers to complementary feeding knowledge questions varied from less than 5% to more than 90% (Table 3). Although more than 70% of the mothers in both areas were aware of the proper age to introduce solid foods, few mothers understood the importance of feeding a variety of foods to small children. Only 35% of the mothers in Nairobi and 20% in Machakos considered important feeding vegetables to their children daily, and less than 10% thought that children should eat meat, fish, or eggs daily. Figure 2 shows the distribution of complementary feeding knowledge scores.

### Associations between knowledge and practice

The existence of high levels of breastfeeding knowledge and reported practice with only a little variance in this data made a logistic regression unnecessary, and consequently not conducted. In the complementary feeding questions, one additional correct answer was estimated to increase the probability of a child in Nairobi getting MDD by 1.29 (95% CI 1.105–1.514). The odds ratio for a one-step knowledge increase for MMF was 1.14 (95% CI 0.984–1.324) in Nairobi. In Machakos, there was no statistically significant associations between knowledge and MDD (OR 1.14; 95% CI 0.938–1.377) or MMF (OR 1.07; 95% CI 0.912–1.261).

## Discussion

This study aimed to evaluate IYCF practices in two different types of area in Kenya and to determine a relation between knowledge and practice in these areas. In the Nairobi slum and rural Machakos, we found similar levels of both IYCF knowledge and practices. Knowledge and practices regarding breastfeeding were better than those for complementary feeding. Gebremedhin (2019) previously reported that African mothers follow breastfeeding recommendations well, but lack correct knowledge on complementary feeding and feed their children accordingly.

### Breastfeeding practices and knowledge

Reported exclusive breastfeeding rates were exceptionally high in both areas, higher than the national average of 61% (Kenya National Bureau of Statistic et al., 2015). A study of over 4000 children in two Nairobi slums revealed that only two percent of children were exclusively breastfed for 6 months (Kimani-Murage et al., 2011) and 40% of children received pre-lacteal feeds during the first 3 days after delivery. The practice of pre-lacteal feeds has been found to increase the risk of cessation of exclusive breastfeeding (Lakati et al., 2010). The median age of transition to partial breastfeeding was 59 days in the resource-constrained settings of South Africa and Tanzania (Richard et al., 2021). Mohamed et al. (2018) discovered that exclusive breastfeeding in a high-poverty area in North-Eastern Kenya occurred at rates half of those in our study. Moreover, data from Tanzania (Dede & Bras, 2020) shows that mothers living in rural areas tend to practice exclusive breastfeeding more often than their counterparts in urban areas. One possible reason for the high exclusive breastfeeding rates in our study relates to self-reporting, which was likely affected by good knowledge on proper breastfeeding practices.

High levels of breastfeeding knowledge existed in both urban slum area in Nairobi and rural area in Machakos, which supports previous reports of approximately 90% of mothers in Kenya being aware of recommended breastfeeding practices (Amolo et al., 2017). Although more than 75% of the mothers in our study knew that a newborn baby should be put to the breast immediately, this was done by only 32% in Nairobi and 42% in Machakos. Over 95% of the mothers in our study knew that babies should get only breastmilk during their first 3 days; however, 10% of the children in Nairobi and 14% in Machakos were given pre-lactal feeds. Focus group discussions revealed that Kenyan mothers had little decision-making power during and after childbirth (Schneider et al., 2017), which may be the case in the current study. Nurses in South Africa were reported to give both pre-lactal feeds and advice that did not support exclusive breastfeeding (Jama et al., 2017). Also mothers’ beliefs affected the decision to exclusively breastfeed (Gewa & Chepkemboi, 2016). Awareness of optimal breastfeeding does not always lead to desirable behavior, as Kimani-Murage et al. (2015) discovered in two slums of Nairobi with a number of social and structural determinants. Social and cultural barriers do not only affect mothers living in slums (Wanjohi et al., 2017); middle-income mothers in Nairobi also find exclusive breastfeeding difficult due to inadequate social, healthcare and workplace support (Wainaina et al., 2018). However, our results suggest that recommendations are often followed even when the reason is unclear; only half of the mothers knew any benefits of colostrum, while almost all the mothers reported giving colostrum to their newborn.

### Complementary feeding practices and knowledge

Although the knowledge of timely introduction of complementary foods was good, the reported practice of timely starting of feeding was not always followed: over 10% of the children under 6 months had already been introduced to foods; likewise over 10% of the children aged 6 months or older did not receive any food. Giving porridge before 4 months has been associated with child malnutrition (Onyango et al., 1998), while a delayed introduction of complementary foods may also lead to malnutrition (Abdel et al., 1995). In addition, adverse beliefs may prevent mothers from offering certain foods to children from the age of 6 months (Chakona 2020), which affects dietary diversity. In this study, over 90% of mothers in both areas disagreed when asked if small children should eat meat, fish, or eggs daily. Reasons for not giving these usually include a lack of knowledge on how to prepare such foods, and a prevailing fear of suffocating (Bazzano et al., 2017; Kram et al., 2016). Consequently, mothers might not give these foods to their children until the age of 12–24 months (Bazzano et al., 2017; Kram et al., 2016). Foods of animal origin can also be considered unsuitable for small children, believed to taste bad to a child, or may be regarded as luxury food (Haileselassie et al., 2020). In some cultures, eggs are believed to slow down speech development and are consequently not given to a child under 1 year (Kram et al., 2016). This is regrettable, as animal products provide a number of nutrients needed by small children; an association between height gain and the consumption of milk and eggs was found in Western Kenya (Mosites et al., 2017).

The adequacy of complementary foods indicated by both dietary diversity and meal frequency in our study followed the pattern of complementary feeding across Sub-Saharan Africa (SSA) and Kenya. In our study, the percentage of the children aged between 6 and 23 months who were fed a MAD was 27% in Nairobi and 13% in Machakos, whereas the average figure in SSA countries was 10% (Gebremedhin 2019) and in Kenya 22% (Kenya National Bureau of Statistics et al., 2015). Although the proportion of children with MMF varied between 67% and 91%, lower MDD percentages (10–44%) resulted in low MAD percentages. A Ghanaian study revealed a similar pattern (Ghana Statistical Service, 2011), that is, more children were sufficiently fed in a day than received a MDD. These results are worrying, as in rural Kenya, dietary diversity is associated with the anthropometric status of children aged one to 3 years (Onyango et al., 1998) and eating from at least four food groups daily is associated with normal growth (Kuche et al., 2020). In addition, low dietary diversity leads to poor nutrition density of the diet, therefore increasing the number of meals needed (Pan American Health Organization, World Health Organization 2003). The MDD was most prevalent in the oldest age group, similar to another study in Kenya (Harvey et al., 2017). In both areas, similar proportions of children had MMF; however, MDD was more infrequent in Machakos than in Nairobi.

### Association between knowledge and practice

The only statistically significant association between knowledge and practice was seen in the proportion of children who got MDD in Nairobi. These results suggest that, along with deficiencies in nutrition knowledge, other barriers exist to feeding children with adequately diverse diets, especially in Machakos. The availability and accessibility of food differ between rural and urban areas. In rural areas of Kenya, the average monthly food expenditure per adult is over 20% higher than in urban areas (Kenya National Bureau of Statistics 2007). Some food is farmed in rural areas, which can cause seasonal variations in the diet, especially when transitioning to family foods (Waswa et al., 2021).

### Strength and limitations

Although the convenience sampling of the Mother-and-Child Health Centers (MCHCs) affects the generalizability of our results, choosing MCHCs that were accessible and receptive led to feasible data collection and a large sample size. The self-reported high level of exclusive breastfeeding in this study might have been affected by bias caused by social desirability and high levels of breastfeeding knowledge. Although recall bias is always possible in retrospective interview studies, as a data collection method, surveys are reliable due to the standardized questions for all participants. Our questionnaire’s open-ended questions made the data collection more flexible, as both mothers and interviewers could provide further explanation and comments. Using a local interview team increased the reliability of the results, as there was neither a cultural gap between mothers and interviewers, nor interpretation needed during the interviews. In addition, pre-training interviewers and using mobile-based questionnaires made the data collection procedure more homogenous. Using recommended IYCF indicators by WHO (2008) made the results comparable to other national results.

JK (Judith Kimiwye) selected the urban slum of the study, excluding the poorest areas, which affected our results. Fotso (2006) discovered a greater prevalence of intra-urban differences in child malnutrition than urban–rural differences. Our results build on the idea that existing evidence of nutrition knowledge does not always translate into practice, as urban Ghanaian mothers’ knowledge was not reflected in feeding practices (Gyampoh et al., 2014). Similarly, in Ethiopia, maternal knowledge of dietary diversity was put into practice only by 16% of mothers with babies aged six to 23 months (Agize et al., 2017).

### Implications for practice

Monotonous diets restrict complementary feeding practices in Kenya. While nutrition education should continue supporting correct breastfeeding practices, education should increasingly emphasize the need to diversify complementary diets with a special focus on animal products. The same interventions can be applied in both urban and rural areas, as these had similar infant and young child feeding knowledge and practices.

## Conclusions

According to our results, IYCF knowledge and practices, excluding DDS, are on a similar level in the Ruaraka slum area in Nairobi and the rural Machakos area in Kenya. Therefore, we conclude that the same interventions can be applied in both areas. We found breastfeeding knowledge and practices were mainly on a good level, while less was known about breastfeeding benefits and how special circumstances should be handled. We found larger gaps in knowledge and practices on complementary feeding. Despite children being fed frequently, food adequacy was poor, and monotonous diets resulted in low MAD. Thus, we recommend that future interventions and government programs should focus mostly on improving dietary diversity during the complementary feeding period.

## Supplemental Material

sj-pdf-1-chc-10.1177_13674935221083451 – Supplemental Material for Mothers’ knowledge and practices on breastfeeding and complementary feeding in an urban slum area and rural area in Kenya: A cross-sectional interview study Click here for additional data file. Supplemental Material, sj-pdf-1-chc-10.1177_13674935221083451 for Mothers’ knowledge and practices on breastfeeding and complementary feeding in an urban slum area and rural area in Kenya: A cross-sectional interview study by Kerttu Uusimäki, Lauriina Schneider, Crippina Lubeka, Judith Kimiwye and Marja Mutanen in Journal of Child Health Care.
