# Supplementary material for: Mothers’ knowledge and practices on breastfeeding and complementary feeding in an urban slum area and rural area in Kenya: A cross-sectional interview study
Source: J Child Health Care. 2022 Apr 15;27(4):612–27. doi: 10.1177/13674935221083451 (PMC10676620; doi:10.1177/13674935221083451)
Supplement: sj-pdf-1-chc-10.1177_13674935221083451 – Supplemental Material for Mothers’ knowledge and practices on breastfeeding and complementary feeding in an urban slum area and rural area in Kenya: A cross-sectional interview study [file sj-pdf-1-chc-10.1177_13674935221083451.pdf]

Supplementary table 1. Breastfeeding and complementary feeding related knowledge and practice questions, from the KAP interviews.

KNOWLEDGE	PRACTICE
**Breastfeeding**	
**Why is skin-to-skin contact important for newborn babies and preterm babies?**	**Was your baby ever breastfed?**
Keeps baby warm	Yes
Helps baby develop better	No
Increases baby's chances of survival	Don't know
It is not important	**Is he/she still breastfeeding?**
Other, specify	Yes
Don't know	No
**How long after birth should a child/baby be put to the breast?**	**How long after birth did you put him/her to the breast?**
Hours	Hours
Days	Days
Immediately/ <1 hour	Immediately/ <1 hour
Don't know	
**Within the first three days after delivery, should a baby be given anything to drink/eat other than breast milk?**	**During the first three days after birth was he/she given anything other than breast milk?**
Yes	Yes
No	No
Don't know	Don't know
**Should a baby be given the very first milk from the breast at birth or soon after?**	**Was he/she fed colostrum?**
Yes	Yes
No	No
Don't know	Don't know
**What are the benefits of feeding baby on colostrum?** [1]	**Was he/she breastfed yesterday during the day or at night?**
Nutritious to baby	Yes
Prevents diseases/infections	No
Helps baby's intestines develop	**Yesterday, was he/she breastfed whenever he/she wanted or on a fixed schedule?**
Nothing specific	Whenever child wanted
Other (specify)	On a fixed schedule
Don't know	
**Is there any difference between mother's milk and cow's milk?** [1]	
No	
Breastmilk easier to digest	
Proteins different	
Breastmilk protects against disease/infection	
Breastmilk helps baby's intestines develop	
Breastfeeding is good for baby's development	
Don't Know	
**If you felt that there was not enough breast milk, what would you do?** [1]	
Give baby formula/other milk	
Give porridge or other liquid food	
Breastfeed more often	
Get help from someone to improve breastfeeding	
Try to improve breastfeeding position/attachment	
Eat and/or drink more herself	
Other, specify	
Nothing	
**For how long IN MONTHS should a child be fed on breast milk without being given anything else NOT EVEN water?**	
X months	
**What (and how) should a young baby (under 6 months) be fed if you are away from the baby?**	
Breastmilk with bottle	
Breastmilk with cup	

Formula or other milk with bottle	
Formula or other milk with cup	
Water, tea etc with bottle	
Water, tea etc with cup	
Porridge with bottle/cup	
Porridge with cup & spoon	
Don't Know	

Complementary feeding

**At what age in months should you introduce first solid/semi-solid food to a baby?**	**Have you introduced your baby to other foods (liquids or semisolids) in addition to breastfeeding?**
X months	Yes
**What should a baby who has started eating, get daily?** [1]	No
Milk products	**Did he/she eat any solid, semi-solid, or soft foods yesterday during the day or at night?**
Meat/fish/egg	Yes
Fruit	**No**
Vegetables	Don't know
Don't Know	
**What type of porridge is better for babies under 1 year of age, liquid or thick?**	
Liquid	
Thick	
**How often should a baby be given food (meals and snacks) after 9 months?**	**Please describe everything that he/she ate yesterday during the day or night, whether at home or outside the home.** [2]
1 meal + snacks	Porridge
2 meals + snacks	Maize, rice, wheat, ugali sorghum, bread, or other foods made from grains?
3-4 meals + snacks	Pumpkin, carrots, squash, or sweet potatoes that are yellow or orange inside?
more often	White potatoes, white yams, cassava, or any other foods made from roots?
**Does it matter if a child is short for his/her age?**	Ripe mangoes, papayas, pawpaw guava (yellow or orange on the inside of fruit)
Yes	Any dark green vegetables?
No	Any other fruits or vegetables?
Don't know	Liver, kidney, heart or other organ meats?
**What are the main food groups?**	Any meat such as beef, pork, lamb, goat, chicken, or duck
Cereals, roots and tubers	Eggs
Milk and milk prodcts	Fresh or dried fish or shelfish
Fruit and vegetables	Any foods made from beans, peas, lentils, or nuts
Fats	Cheese or other food made from milk
Meat, fish and eggs	Sweets foods like cookies, cake, candy, soda
Pulses	Any other solid, semisolid or soft foods

[1)]multiple choice question
[2)]Interviewer marks the foods to either 1. Breakfast, 2. Lunch, 3. Dinner or 4. Snacks

Supplementary table 2. Means and frequencies of dietary diversity score (DDS) and meal frequency (MF) of children aged six months or older in Nairobi and Machakos in Kenya.

	Nairobi (n=204)				Machakos (n=213)			
	mean	(sd)	n	(%)	mean	(sd)	n	(%)
**Dietary Diversity Score (DDS)**	2.7	(1.4)			2.4	(1.3)		
0			20	(9.8)			28	(13.1)
1			13	(6.4)			10	(4.7)
2			48	(23.5)			66	(31.0)
3			64	(31.4)			67	(31.5)
≥4			59	(28.9)			42	(19.7)
**Children who had DDS 0 by age**								

6 months			12				12	
7 months			3				4	
8 months			1				2	
9 months			2				4	
10 months			-				3	
11 months			-				1	
12 months			-				2	
15 months			1				-	
17 months			1				-	
**Meal frequency (MF)**	3.0	(1.3)			3.1	(1.3)		
0			20	(9.8)			27	(12.7)
1			10	(4.9)			2	(0.9)
2			16	(7.8)			16	(7.5)
3			49	(24.0)			54	(25.4)
≥4			109	(53.4)			114	(53.5)

ÿØÿà JFIF , ÿÛ C

□□

□□□□□□□□-□□□□ $.' ",#□□(7),01444'9=82<.342ÿÛ C□

□□□□2!□!22222222222222222222222222222222222222222222222222ÿÀ □□

ä ö□□" □□□□□□ÿÄ □□□□□□□□□ □□□□□□□□□

ÿÄ µ□ □□□□□□□□□□□□ □}□□□ □□□□!1A□□Qa□"q□2□‘¡□#B±Á□RÑð$3br,
□□□□□%&'()*456789:CDEFGHIJSTUVWXYZcdefghijstuvwxyzƒ„…†‡ˆ‰Š’“”•–—
˜™š¢£¤¥¦§¨©ª²³´µ¶·¸¹ºÂÃÄÅÆÇÈÉÊÒÓÔÕÖ×ØÙÚáâãäåæçèéêñòóôõö÷øùúÿÄ -
□ □□□□□□□□□□□ □□□□□□□□□

ÿÄ µ□ □□□□□□□□□□□□ □□w □□□□□□!1□□AQ□aq□"2□□□B‘¡±Á #3Rð□brÑ
□$4á%ñ□□□□&'()*56789:CDEFGHIJSTUVWXYZcdefghijstuvwxyz,ƒ„…†‡ˆ‰Š’“”•–—
˜™š¢£¤¥¦§¨©ª²³´µ¶·¸¹ºÂÃÄÅÆÇÈÉÊÒÓÔÕÖ×ØÙÚâãäåæçèéêòóôõö÷øùúÿÚ

□□ □□□□ ? ÷ú(¢€
(¢€
(¢€
(¢€
(¢€
(¢€
(¢€
(¢€
(¢€
(¢€
(¢€
(¢€
(¢€
(¢€
(¢€
(¢€
(¢€
(¢€
(¢€
(¢€
(¢€
(¢€
(¢€
(¢€
(¢€
(¢€
(¢€
(¢€
(¢€
(¢€
(¢€
(¢€
(¢€
(¢€
(¢€
(¢€
(¢€
(¢€
(¢€
(¢€
(¢€
(¢€
(¢€
(¢€
(¢€
(¢€
(¢€
(¢€
(¢€
(¢€
(¢€
(¢€
(¢€
(¢€
(¢€
(¢€
(¢€
(¢€

(¢€
(¢€
(¢€
(¢€
(¢€
(¢€
(¢€
(¢€
(¢€
(¢€
(¢€
(¢€
(¢€
(¢€
(¢€
(¢€
(¢€
(¢€
(¢€
(¢€
(¢€
(¢€
(¢€
(¢€
(¢€
(¢€
(¢€
(¢€
(¢€
(¢€
(¢€
(¢€
(¢€
(¢€
(¢€
(¢€
(¢€
(¢€
(¢€
(¢€
(¢€
(¢€
(¢€
(¢€
(¢€
(¢€
(¢€
(¢€
(¢€
(¢€
(¢€
(¢€
(¢€
(¢€
(¢€
(¢€
(¢€
(¢€

(¢€
(¢€
(¢€
(¢€
(¢€
(¢€
(¢€
(¢€
(¢€
(¢€
(¢€
(¢€
(¢€
(¢€
(¢€
(¢€
(¢€
(¢€
(¢€
(¢€
(¢€
(¢€
(¢€
(¢€
(¢€
(¢€
(¢€
(¢€
(¢€
(¢€
(¢€
(¢€
(¢€
(¢€
(¢€
(¢€
(¢€
(¢€
(¢€
(¢€
(¢€
(¢€
(¢€
(¢€
(¢€
(¢€
(¢€
(¢€
(¢€
(¢€
(¢€
(¢€
(¢€
(¢€
(¢€
(¢€
(¢€
(¢€

(¢€
(¢€
(¢€
(¢€
(¢€
(¢€
(¢€
(¢€
(¢€
(¢€
(¢€
(¢€
(¢€
(¢€
(¢€
(¢€
(¢€
(¢€
(¢€
(¢€
(¢€
(¢€
(¢€
(¢€
(¢€
(¢€
(¢€
(¢€
(¢€
(¢€
(¢€
(¢€
(¢€
(¢€
(¢€
(¢€
(¢€
(¢€
(¢€
(¢€
(¢€
(¢€
(¢€
(¢€
(¢€
(¢€
(¢€
(¢€
(¢€
(¢€
(¢€
(¢€
(¢€
(¢€
(¢€
(¢€
(¢€
(¢€

(¢€
(¢€
(¢€
(¢€
(¢€
(¢€
(¢€
(¢€
(¢€
(¢€
(¢€
(¢€
(¢€
(¢€
(¢€
(¢€
(¢€
(¢€
(¢€
(¢€
(¢€
(¢€
(¢€
(¢€
(¢€
(¢€
(¢€
(¢€
(¢€
(¢€
(¢€
(¢€
(¢€
(¢€
(¢€
(¢€
(¢€
(¢€
(¢€
(¢€
(¢€
(¢€
(¢€
(¢€
(¢€
(¢€
(¢€
(¢€
(¢€
(¢€
(¢€
(¢€
(¢€
(¢€
(¢€
(¢€
(¢€
(¢€

(¢€
(¢€
(¢€
(¢€
(¢€
(¢€
(¢€
(¢€
(¢€
(¢€
(¢€
(¢€
(¢€
(¢€
(¢€
(¢€
(¢€
(¢€
(¢€
(¢€
(¢€
(¢€
(¢€
(¢€
(¢€
(¢€
(¢€
(¢€
(¢€
(¢€
(¢€
(¢€
(¢€
(¢€
(¢€
(¢€
(¢€
(¢€
(¢€
(¢€
(¢€
(¢€
(¢€
(¢€
(¢€
(¢€
(¢€
(¢€
(¢€
(¢€
(¢€
(¢€
(¢€
(¢€
(¢€
(¢€
(¢€
(¢€

(¢€
(¢€
(¢€
(¢€
(¢€
(¢€
(¢€
(¢€
(¢€
(¢€
(¢€
(¢€
(¢€
(¢€
(¢€
(¢€
(¢€
(¢€
(¢€
(¢€
(¢€
(¢€
(¢€
(¢€
(¢€
(¢€
(¢€
(¢€
(¢€
(¢€
(¢€
(¢€
(¢€
(¢€
(¢€
(¢€
(¢€
(¢€
(¢€
(¢€
(¢€
(¢€
(¢€
(¢€
(¢€
(¢€
(¢€
(¢€
(¢€
(¢€
(¢€
(¢€
(¢€
(¢€
(¢€
(¢€
(¢€
(¢€

(¢€
(¢€
(¢€
(¢€
(¢€
(¢€
(¢€
(¢€
(¢€
(¢€
(¢€
(¢€
(¢€
(¢€
(¢€
(¢€
(¢€
(¢€
(¢€
(¢€
(¢€
(¢€
(¢€
(¢€
(¢€
(¢€
(¢€
(¢€
(¢€
(¢€
(¢€
(¢€
(¢€
(¢€
(¢€
(¢€
(¢€
(¢€
(¢€
(¢€
(¢€
(¢€
(¢€
(¢€
(¢€
(¢€
(¢€
(¢€
(¢€
(¢€
(¢€
(¢€
(¢€
(¢€
(¢€
(¢€
(¢€
(¢€

(¢€
(¢€
(¢€
(¢€
(¢€
(¢€
(¢€
(¢€
(¢€
(¢€
(¢€
(¢€
(¢€
(¢€
(¢€
(¢€
(¢€
(¢€
(¢€
(¢€
(¢€
(¢€
(¢€
(¢€
(¢€
(¢€
(¢€
(¢€
(¢€
(¢€
(¢€
(¢€
(¢€
(¢€
(¢€
(¢€
(¢€
(¢€
(¢€
(¢€
(¢€
(¢€
(¢€
(¢€
(¢€
(¢€
(¢€
(¢€
(¢€
(¢€
(¢€
(¢€
(¢€
(¢€
(¢€
(¢€
(¢€
(¢€

(¢∈
(¢∈
(¢∈
(¢∈
(¢∈
(¢∈
(¢∈
(¢∈
(¢∈
(¢∈
(¢∈
(¢∈
(¢∈
(¢∈
(¢∈
(¢∈
(¢∈
(¢∈
(¢∈
(¢∈
(¢∈
(¢∈
(¢∈
(¢∈
(¢∈
(¢∈
(¢∈
(¢∈
(¢∈
(¢∈
(¢∈
(¢∈
(¢∈
(¢∈
(¢∈
(¢∈
(¢∈
(¢∈
(¢∈
(¢∈
(¢∈
(¢∈
(¢∈
(¢∈
(¢∈
(¢∈
(¢∈
(¢∈
(¢∈
(¢∈
(¢∈
(¢∈
(¢∈
(¢∈
(¢∈
(¢∈
(¢∈
(¢∈

(¢€
(¢€
(¢€
(¢€
(¢€
(¢€
(¢€
(¢€
(¢€
(¢€
(¢€
(¢€
(¢€
(¢€
(¢€
(¢€
(¢€
(¢€
(¢€
(¢€
(¢€
(¢€
(¢€
(¢€
(¢€
(¢€
(¢€
(¢€
(¢€
(¢€
(¢€
(¢€
(¢€
(¢€
(¢€
(¢€
(¢€
(¢€
(¢€
(¢€
(¢€
(¢€
(¢€
(¢€
(¢€
(¢€
(¢€
(¢€
(¢€
(¢€
(¢€
(¢€
(¢€
(¢€
(¢€
(¢€
(¢€
(¢€

(¢€
(¢€
(¢€
(¢€
(¢€
(¢€
(¢€
(¢€
(¢€
(¢€
(¢€
(¢€
(¢€
(¢€
(¢€
(¢€
(¢€
(¢€
(¢€
(¢€
(¢€
(¢€
(¢€
(¢€
(¢€
(¢€
(¢€
(¢€
(¢€
(¢€
(¢€
(¢€
(¢€
(¢€
(¢€
(¢€
(¢€
(¢€
(¢€
(¢€
(¢€
(¢€
(¢€
(¢€
(¢€
(¢€
(¢€
(¢€
(¢€
(¢€
(¢€
(¢€
(¢€
(¢€
(¢€
(¢€
(¢€
(¢€

(¢€
(¢€
(¢€
(¢€
(¢€
(¢€
(¢€
(¢€
(¢€
(¢€
(¢€
(¢€
(¢€
(¢€
(¢€
(¢€
(¢€
(¢€
(¢€
(¢€
(¢€
(¢€
(¢€
(¢€
(¢€
(¢€
(¢€
(¢€
(¢€
(¢€
(¢€
(¢€
(¢€
(¢€
(¢€
(¢€
(¢€
(¢€
(¢€
(¢€
(¢€
(¢€
(¢€
(¢€
(¢€
(¢€
(¢€
(¢€
(¢€
(¢€
(¢€
(¢€
(¢€
(¢€
(¢€
(¢€
(¢€
(¢€

(¢€
(¢€
(¢€
(¢€
(¢€
(¢€
(¢€
(¢€
(¢€
(¢€
(¢€
(¢€
(¢€
(¢€
(¢€
(¢€
(¢€
(¢€
(¢€
(¢€
(¢€
(¢€
(¢€
(¢€
(¢€
(¢€
(¢€
(¢€
(¢€
(¢€
(¢€
(¢€
(¢€
(¢€
(¢€
(¢€
(¢€
(¢€
(¢€
(¢€
(¢€
(¢€
(¢€
(¢€
(¢€
(¢€
(¢€
(¢€
(¢€
(¢€
(¢€
(¢€
(¢€
(¢€
(¢€
(¢€
(¢€
(¢€

(¢€
(¢€
(¢€
(¢€
(¢€
(¢€
(¢€
(¢€
(¢€
(¢€
(¢€
(¢€
(¢€
(¢€
(¢€
(¢€
(¢€
(¢€
(¢€
(¢€
(¢€
(¢€
(¢€
(¢€
(¢€
(¢€
(¢€
(¢€
(¢€
(¢€
(¢€
(¢€
(¢€
(¢€
(¢€
(¢€
(¢€
(¢€
(¢€
(¢€
(¢€
(¢€
(¢€
(¢€
(¢€
(¢€
(¢€
(¢€
(¢€
(¢€
(¢€
(¢€
(¢€
(¢€
(¢€
(¢€
(¢€
(¢€

(¢€
(¢€
(¢€
(¢€
(¢€
(¢€
(¢€
(¢€
(¢€
(¢€
(¢€
(¢€
(¢€
(¢€
(¢€
(¢€
(¢€
(¢€
(¢€
(¢€
(¢€
(¢€
(¢€
(¢€
(¢€
(¢€
(¢€
(¢€
(¢€
(¢€
(¢€
(¢€
(¢€
(¢€
(¢€
(¢€
(¢€
(¢€
(¢€
(¢€
(¢€
(¢€
(¢€
(¢€
(¢€
(¢€
(¢€
(¢€
(¢€
(¢€
(¢€
(¢€
(¢€
(¢€
(¢€
(¢€
(¢€
(¢€

(¢€
(¢€
(¢€
(¢€
(¢€
(¢€
(¢€
(¢€
(¢€
(¢€
(¢€
(¢€
(¢€
(¢€
(¢€
(¢€
(¢€
(¢€
(¢€
(¢€
(¢€
(¢€
(¢€
(¢€
(¢€
(¢€
(¢€
(¢€
(¢€
(¢€
(¢€
(¢€
(¢€
(¢€
(¢€
(¢€
(¢€
(¢€
(¢€
(¢€
(¢€
(¢€
(¢€
(¢€
(¢€
(¢€
(¢€
(¢€
(¢€
(¢€
(¢€
(¢€
(¢€
(¢€
(¢€
(¢€
(¢€
(¢€

(¢€
(¢€
(¢€
(¢€
(¢€
(¢€
(¢€
(¢€
(¢€
(¢€
(¢€
(¢€
(¢€
(¢€
(¢€
(¢€
(¢€
(¢€
(¢€
(¢€
(¢€
(¢€
(¢€
(¢€
(¢€
(¢€
(¢€
(¢€
(¢€
(¢€
(¢€
(¢€
(¢€
(¢€
(¢€
(¢€
(¢€
(¢€
(¢€
(¢€
(¢€
(¢€
(¢€
(¢€
(¢€
(¢€
(¢€
(¢€
(¢€
(¢€
(¢€
(¢€
(¢€
(¢€
(¢€
(¢€
(¢€
(¢€

(¢€
(¢€
(¢€
(¢€
(¢€
(¢€
(¢€
(¢€
(¢€
(¢€
(¢€
(¢€
(¢€
(¢€
(¢€
(¢€
(¢€
(¢€
(¢€
(¢€
(¢€
(¢€
(¢€
(¢€
(¢€
(¢€
(¢€
(¢€
(¢€
(¢€
(¢€
(¢€
(¢€
(¢€
(¢€
(¢€
(¢€
(¢€
(¢€
(¢€
(¢€
(¢€
(¢€
(¢€
(¢€
(¢€
(¢€
(¢€
(¢€
(¢€
(¢€
(¢€
(¢€
(¢€
(¢€
(¢€
(¢€
(¢€

(¢€
(¢€
(¢€
(¢€
(¢€
(¢€
(¢€
(¢€
(¢€
(¢€
(¢€
(¢€
(¢€
(¢€
(¢€
(¢€
(¢€
(¢€
(¢€
(¢€
(¢€
(¢€
(¢€
(¢€
(¢€
(¢€
(¢€
(¢€
(¢€
(¢€
(¢€
(¢€
(¢€
(¢€
(¢€
(¢€
(¢€
(¢€
(¢€
(¢€
(¢€
(¢€
(¢€
(¢€
(¢€
(¢€
(¢€
(¢€
(¢€
(¢€
(¢€
(¢€
(¢€
(¢€
(¢€
(¢€
(¢€
(¢€

(¢€
(¢€
(¢€
(¢€
(¢€
(¢€
(¢€
(¢€
(¢€
(¢€
(¢€
(¢€
(¢€
(¢€
(¢€
(¢€
(¢€
(¢€
(¢€
(¢€
(¢€
(¢€
(¢€
(¢€
(¢€
(¢€
(¢€
(¢€
(¢€
(¢€
(¢€
(¢€
(¢€
(¢€
(¢€
(¢€
(¢€
(¢€
(¢€
(¢€
(¢€
(¢€
(¢€
(¢€
(¢€
(¢€
(¢€
(¢€
(¢€
(¢€
(¢€
(¢€
(¢€
(¢€
(¢€
(¢€
(¢€
(¢€

(¢€
(¢€
(¢€
(¢€
(¢€
(¢€
(¢€
(¢€
(¢€
(¢€
(¢€
(¢€
(¢€
(¢€
(¢€
(¢€
(¢€
(¢€
(¢€
(¢€
(¢€
(¢€
(¢€
(¢€
(¢€
(¢€
(¢€
(¢€
(¢€
(¢€
(¢€
(¢€
(¢€
(¢€
(¢€
(¢€
(¢€
(¢€
(¢€
(¢€
(¢€
(¢€
(¢€
(¢€
(¢€
(¢€
(¢€
(¢€
(¢€
(¢€
(¢€
(¢€
(¢€
(¢€
(¢€
(¢€
(¢€
(¢€

(¢€
(¢€
(¢€
(¢€
(¢€
(¢€
(¢€
(¢€
(¢€
(¢€
(¢€
(¢€
(¢€
(¢€
(¢€
(¢€
(¢€
(¢€
(¢€
(¢€
(¢€
(¢€
(¢€
(¢€
(¢€
(¢€
(¢€
(¢€
(¢€
(¢€
(¢€
(¢€
(¢€
(¢€
(¢€
(¢€
(¢€
(¢€
(¢€
(¢€
(¢€
(¢€
(¢€
(¢€
(¢€
(¢€
(¢€
(¢€
(¢€
(¢€
(¢€
(¢€
(¢€
(¢€
(¢€
(¢€
(¢€
(¢€

(¢€
(¢€
(¢€
(¢€
(¢€
(¢€
(¢€
(¢€
(¢€
(¢€
(¢€
(¢€
(¢€
(¢€
(¢€
(¢€
(¢€
(¢€
(¢€
(¢€
(¢€
(¢€
(¢€
(¢€
(¢€
(¢€
(¢€
(¢€
(¢€
(¢€
(¢€
(¢€
(¢€
(¢€
(¢€
(¢€
(¢€
(¢€
(¢€
(¢€
(¢€
(¢€
(¢€
(¢€
(¢€
(¢€
(¢€
(¢€
(¢€
(¢€
(¢€
(¢€
(¢€
(¢€
(¢€
(¢€
(¢€

(¢€
(¢€
(¢€
(¢€
(¢€
(¢€
(¢€
(¢€
(¢€
(¢€
(¢€
(¢€
(¢€
(¢€
(¢€
(¢€
(¢€
(¢€
(¢€
(¢€
(¢€
(¢€
(¢€
(¢€
(¢€
(¢€
(¢€
(¢€
(¢€
(¢€
(¢€
(¢€
(¢€
(¢€
(¢€
(¢€
(¢€
(¢€
(¢€
(¢€
(¢€
(¢€
(¢€
(¢€
(¢€
(¢€
(¢€
(¢€
(¢€
(¢€
(¢€
(¢€
(¢€
(¢€
(¢€
(¢€
(¢€
(¢€

(¢€
(¢€
(¢€
(¢€
(¢€
(¢€
(¢€
(¢€
(¢€
(¢€
(¢€
(¢€
(¢€
(¢€
(¢€
(¢€
(¢€
(¢€
(¢€
(¢€
(¢€
(¢€
(¢€
(¢€
(¢€
(¢€
(¢€
(¢€
(¢€
(¢€
(¢€
(¢€
(¢€
(¢€
(¢€
(¢€
(¢€
(¢€
(¢€
(¢€
(¢€
(¢€
(¢€
(¢€
(¢€
(¢€
(¢€
(¢€
(¢€
(¢€
(¢€
(¢€
(¢€
(¢€
(¢€
(¢€
(¢€
(¢€

(¢€
(¢€
(¢€
(¢€
(¢€
(¢€
(¢€
(¢€
(¢€
(¢€
(¢€
(¢€
(¢€
(¢€
(¢€
(¢€
(¢€
(¢€
(¢€
(¢€
(¢€
(¢€
(¢€
(¢€
(¢€
(¢€
(¢€
(¢€
(¢€
(¢€
(¢€
(¢€
(¢€
(¢€
(¢€
(¢€
(¢€
(¢€
(¢€
(¢€
(¢€
(¢€
(¢€
(¢€
(¢€
(¢€
(¢€
(¢€
(¢€
(¢€
(¢€
(¢€
(¢€
(¢€
(¢€
(¢€
(¢€
(¢€

(¢€
(¢€
(¢€
(¢€
(¢€
(¢€
(¢€
(¢€
(¢€
(¢€
(¢€
(¢€
(¢€
(¢€
(¢€
(¢€
(¢€
(¢€
(¢€
(¢€
(¢€
(¢€
(¢€
(¢€
(¢€
(¢€
(¢€
(¢€
(¢€
(¢€
(¢€
(¢€
(¢€
(¢€
(¢€
(¢€
(¢€
(¢€
(¢€
(¢€
(¢€
(¢€
(¢€
(¢€
(¢€
(¢€
(¢€
(¢€
(¢€
(¢€
(¢€
(¢€
(¢€
(¢€
(¢€
(¢€
(¢€
(¢€

(¢€
(¢€
(¢€
(¢€
(¢€
(¢€
(¢€
(¢€
(¢€
(¢€
(¢€
(¢€
(¢€
(¢€
(¢€
(¢€
(¢€
(¢€
(¢€
(¢€
(¢€
(¢€
(¢€
(¢€
(¢€
(¢€
(¢€
(¢€
(¢€
(¢€
(¢€
(¢€
(¢€
(¢€
(¢€
(¢€
(¢€
(¢€
(¢€
(¢€
(¢€
(¢€
(¢€
(¢€
(¢€
(¢€
(¢€
(¢€
(¢€
(¢€
(¢€
(¢€
(¢€
(¢€
(¢€
(¢€
(¢€
(¢€

(¢€
(¢€
(¢€
(¢€
(¢€
(¢€
(¢€
(¢€
(¢€
(¢€
(¢€
(¢€
(¢€
(¢€
(¢€
(¢€
(¢€
(¢€
(¢€
(¢€
(¢€
(¢€
(¢€
(¢€
(¢€
(¢€
(¢€
(¢€
(¢€
(¢€
(¢€
(¢€
(¢€
(¢€
(¢€
(¢€
(¢€
(¢€
(¢€
(¢€
(¢€
(¢€
(¢€
(¢€
(¢€
(¢€
(¢€
(¢€
(¢€
(¢€
(¢€
(¢€
(¢€
(¢€
(¢€
(¢€
(¢€
(¢€

(¢€
(¢€
(¢€
(¢€
(¢€
(¢€
(¢€
(¢€
(¢€
(¢€
(¢€
(¢€
(¢€
(¢€
(¢€
(¢€
(¢€
(¢€
(¢€
(¢€
(¢€
(¢€
(¢€
(¢€
(¢€
(¢€
(¢€
(¢€
(¢€
(¢€
(¢€
(¢€
(¢€
(¢€
(¢€
(¢€
(¢€
(¢€
(¢€
(¢€
(¢€
(¢€
(¢€
(¢€
(¢€
(¢€
(¢€
(¢€
(¢€
(¢€
(¢€
(¢€
(¢€
(¢€
(¢€
(¢€
(¢€
(¢€

(¢€
(¢€
(¢€
(¢€
(¢€
(¢€
(¢€
(¢€
(¢€
(¢€
(¢€
(¢€
(¢€
(¢€
(¢€
(¢€
(¢€
(¢€
(¢€
(¢€
(¢€
(¢€
(¢€
(¢€
(¢€
(¢€
(¢€
(¢€
(¢€
(¢€
(¢€
(¢€
(¢€
(¢€
(¢€
(¢€
(¢€
(¢€
(¢€
(¢€
(¢€
(¢€
(¢€
(¢€
(¢€
(¢€
(¢€
(¢€
(¢€
(¢€
(¢€
(¢€
(¢€
(¢€
(¢€
(¢€
(¢€
(¢€

(¢€
(¢€
(¢€
(¢€
(¢€
(¢€
(¢€
(¢€
(¢€
(¢€
(¢€
(¢€
(¢€
(¢€
(¢€
(¢€
(¢€
(¢€
(¢€
(¢€
(¢€
(¢€
(¢€
(¢€
(¢€
(¢€
(¢€
(¢€
(¢€
(¢€
(¢€
(¢€
(¢€
(¢€
(¢€
(¢€
(¢€
(¢€
(¢€
(¢€
(¢€
(¢€
(¢€
(¢€
(¢€
(¢€
(¢€
(¢€
(¢€
(¢€
(¢€
(¢€
(¢€
(¢€
(¢€
(¢€
(¢€
(¢€

(¢€
(¢€
(¢€
(¢€
(¢€
(¢€
(¢€
(¢€
(¢€
(¢€
(¢€
(¢€
(¢€
(¢€
(¢€
(¢€
(¢€
(¢€
(¢€
(¢€
(¢€
(¢€
(¢€
(¢€
(¢€
(¢€
(¢€
(¢€
(¢€
(¢€
(¢€
(¢€
(¢€
(¢€
(¢€
(¢€
(¢€
(¢€
(¢€
(¢€
(¢€
(¢€
(¢€
(¢€
(¢€
(¢€
(¢€
(¢€
(¢€
(¢€
(¢€
(¢€
(¢€
(¢€
(¢€
(¢€
(¢€
(¢€

(¢€
(¢€
(¢€
(¢€
(¢€
(¢€
(¢€
(¢€
(¢€
(¢€
(¢€
(¢€
(¢€
(¢€
(¢€
(¢€
(¢€
(¢€
(¢€
(¢€
(¢€
(¢€
(¢€
(¢€
(¢€
(¢€
(¢€
(¢€
(¢€
(¢€
(¢€
(¢€
(¢€
(¢€
(¢€
(¢€
(¢€
(¢€
(¢€
(¢€
(¢€
(¢€
(¢€
(¢€
(¢€
(¢€
(¢€
(¢€
(¢€
(¢€
(¢€
(¢€
(¢€
(¢€
(¢€
(¢€
(¢€
(¢€

(¢€
(¢€
(¢€
(¢€
(¢€
(¢€
(¢€
(¢€
(¢€
(¢€
(¢€
(¢€
(¢€
(¢€
(¢€
(¢€
(¢€
(¢€
(¢€
(¢€
(¢€
(¢€
(¢€
(¢€
(¢€
(¢€
(¢€
(¢€
(¢€
(¢€
(¢€
(¢€
(¢€
(¢€
(¢€
(¢€
(¢€
(¢€
(¢€
(¢€
(¢€
(¢€
(¢€
(¢€
(¢€
(¢€
(¢€
(¢€
(¢€
(¢€
(¢€
(¢€
(¢€
(¢€
(¢€
(¢€
(¢€

(¢€
(¢€
(¢€
(¢€
(¢€
(¢€
(¢€
(¢€
(¢€
(¢€
(¢€
(¢€
(¢€
(¢€
(¢€
(¢€
(¢€
(¢€
(¢€
(¢€
(¢€
(¢€
(¢€
(¢€
(¢€
(¢€
(¢€
(¢€
(¢€
(¢€
(¢€
(¢€
(¢€
(¢€
(¢€
(¢€
(¢€
(¢€
(¢€
(¢€
(¢€
(¢€
(¢€
(¢€
(¢€
(¢€
(¢€
(¢€
(¢€
(¢€
(¢€
(¢€
(¢€
(¢€
(¢€
(¢€
(¢€
(¢€

(¢€
(¢€
(¢€
(¢€
(¢€
(¢€
(¢€
(¢€
(¢€
(¢€
(¢€
(¢€
(¢€
(¢€
(¢€
(¢€
(¢€
(¢€
(¢€
(¢€
(¢€
(¢€
(¢€
(¢€
(¢€
(¢€
(¢€
(¢€
(¢€
(¢€
(¢€
(¢€
(¢€
(¢€
(¢€
(¢€
(¢€
(¢€
(¢€
(¢€
(¢€
(¢€
(¢€
(¢€
(¢€
(¢€
(¢€
(¢€
(¢€
(¢€
(¢€
(¢€
(¢€
(¢€
(¢€
(¢€
(¢€
(¢€

(¢€
(¢€
(¢€
(¢€
(¢€
(¢€
(¢€
(¢€
(¢€
(¢€
(¢€
(¢€
(¢€
(¢€
(¢€
(¢€
(¢€
(¢€
(¢€
(¢€
(¢€
(¢€
(¢€
(¢€
(¢€
(¢€
(¢€
(¢€
(¢€
(¢€
(¢€
(¢€
(¢€
(¢€
(¢€
(¢€
(¢€
(¢€
(¢€
(¢€
(¢€
(¢€
(¢€
(¢€
(¢€
(¢€
(¢€
(¢€
(¢€
l’$Q´’:¢(,ÌÇ □Ô“N®ã

÷6ÿ
5çµ,$1"1^»□DWÿ ÇI

ÿ Ú□Á–WÒÛG

«x±¶Ñ=¼   å¿°îu8ü*ç‡~9xWÄ°õ®‘ooª[Ü]6Èžæ□□[˚Ê»-~•ä?□,ü#}â
ë□□Ãi5Û¢
□ï 1±ÉÞ0x-
÷p□¾+ÝÛág„W_˚Ö-4´°¼³˜JŸd>Z1□□'ÝÇÐ□ï@□•□ç¿□þ)'Ã¹´èŽ□Úƒ^+·ü|yA□□?°Ùë]w†õ¥
ñ□†ôýa!0-ä
0‰›qLŽ™ï@□”W”Ÿ□q□‰?ð^□`¾>ßöµý¨}íÛwlÙÓ?íWKñÇ«ðûB·Ô›N7æ{• ˆMåc*Íœí?Ýô

ÆŠðÍwö‹µƒI³m□KYu□ã□:O!1[Ÿîä`¹ú`

úä□ü□ûAIªkpiÞ$Óím£¹q□]Z-
Œx□•‰àžùãÒ€=ÞŠç¼oâ•ðg„îõÖ´7bÜ □‰6n,á~ö

:úW˜^~Ñ–
á¨®-tƒý¯+²ý‛¦Ý□J:3>Ñœö vê8È□¸V/ŠüOeàÿ

Ükz„W□Û@P2Ûª³•Ì□`□□Së^%áÿ Ú>õõHãñ

‟h¶.ÁZ[=á¢Þ!‹nÇ Çô®ÿ ãlÑÜ|Ôf…Õâ‘-Ý□NC□*□E ox-
ÇÚW□¬®®´«{Èc¶□Fâé□I$g□¬ÕÕW^~Í□ð.ëŸõöŸú□l|Fø×gàíEô}.Ñu□N0<âï¶(I□ ã-
oaŒg®x □W¢¾oÒÿ i-e/□û[D°-ÔŸ˜Z□□Àõ□™□úqøW¿i□í†» Á¬éÒùÖsÅæ#t<u□v
,□õ□□¥Ex¶—ûEé7V°Œ×ú<-fÞ0Öñ-È‘®□œ□□hÛêO8-
†yx?i=hjaî4=<Ønæ(ÙÄ¡□ß'□ÿ Àh□é□+Æ¼cñÿ KÒí-WÃp&£wq
ÌÍ1"8□

…`9/ê□□õíU~üy—Ä- ¶ÑüA§Û[=Û^à¸µ,□9ûªÊÄžO□Ï\qÜ {}□[PÔ-
t-:âþúe,ÖÞ3$²7EQÖ¾□□i□ÿ ·:h□5ªÚ©ÂÉ|Y□Ç®Õ#oÓ&€>‰¢¼ká÷Çh|K¬C£ë¶1XÝÜ6È'…‰
‰ÜôR□*OnNO□WiñÇ«ðûB·Ô›N7æ{• ˆMåc*Íœí?Ýô

ÆŠðí□ö^´¶Ñ¬_GÓ□mNâ/2hæ□´vÇ'å$`¹ã<c□□ø¦ø□ãìÚÞ¿m¤ø‡Oµ¶û[^¢¹µ,□\ð¡•‰àž3ž=;
€□l¼½´Ó-^êúê□[xþüÓÈ□□·$ð*-;VÓu^□}3P´¾…[kIm2Ê úeIæ¼Kãß•-
□nü□4ÒÆháœÞ□±·çÝ□›yû½r:×□ð×âÇü+Í2öÏû□ûCíS
wý«ÊÛ□Œccf€>¶¢¸¿□|F±ð□ƒl¼Aqg4í|□È¶F -
dß†nÀ□×□é^7qûHø‘§&ÛFÒ£‡<,,Gl}C□å@-
LQ^iðÇâå·□g›N°³□:¤Qù¡□÷$¨□□®y□dpsëž¸µñ/â,|:“MC¤¶ ×¢CÅÇ”□&ßö[?{ô □A¢¼□Å□
´D¶—
ñ[øoOµž%□Zy®·0.@%T)□:g¹Ï□ë«©ü}M□_ƒL¿ðÜ‰□$□Ip.°UdE}Á6r o^Ý¨□Ùè¤WV@êÀ©□

□□z×”ø[ãd>+ñÒxvÏA□A#È□ð]□ùP□¸¦Á€qýîô□êõ□åí¦□j÷W×PÚÛÇ÷æž@ˆ½¹'□S×ƒ|{ñòÃ□ß
,FšXÍ□3›Ã66üû±³o?w®GZ öÝ;VÓuˆ□}3P´¾…[kIm2Ê úeIæ®WÉ?□~,□Â¼Ó/lÿ ±□ľ>Õ0—
□Ú¼–¸□Æ66kè/□|F±ð□ƒl¼Aqg4í|□È¶F –dß†nÀ□×□é@□¥□ó=Çí#âFœ›m□JŽ□ð²□±õ

?•zoÃ‹–
Þ=žm:êÌXê‘Gæ„WÜ’ □¹ä□‘ÁÏ®zà□iŒþ.h-□ÖÓJÔí5)§x□pÖÑÆË´’□,àçå=«¸´¹KË8.£

hÖEÔ23ù×Ì´Oü”{ûÅÿ ¡É[:—íśa¥‡´»i!·#iïw-
0… áTŒ©ü }\oŽ~%hßä±MVÚþcxÇöXÑ±·Îæ_ï
Âøañz ÜI¦_Z%-
¬ˆdU‰ŽevçG§<sž¸âi•øüðßýs¸þqÐ¹èÕ·ˆô=bÍ%K{¸Ä‘¬ÀÜGëZUÇ|/t‹á^$Œ¨
‹f™Ž œ’kÍ<UûEµ¶£-·†tÈ. Š‹»ÂØ‶ÝQH {“ø
÷Ê+ÁüûCý»T†ËÄÚ}½¬S0AwjX$dôÜ¬IÇ¾xô¯xÎFE åúßÇ¯hš½ÆœÑjw’[¹ŽI-
aB›•Á ³©8#®1^¡\Fð›ÁzuÝÕìšDW×¼®÷ø˜Äœ#h>™÷4ÍCûEø6Y’7³ÖaV`

• □eTzœHN>€×«Z]A}i□Ý¬©5¼È$ŽD9

¤d□øWÊ?□l</§øÂ□|4¶±Ÿ#7qZ□å¤™8À□□Ž {z×Ð?
-.ì~□è0^«,þC>□¨Fvdÿ ÇJÐ□gE□P□^W«|}ð¦□¬^i³Yk□Ëi3Àï□1•,¤ƒŒÈ

2=+Õ+À>□Ã□ß□|l²Æ′ ÷$□P□ảâ€7ÿ á£¼ÿ @ÝsþüCÿ ÇkÕôûØµ=6Öþ â□˜Rd

0ÁX□2=pi~Ágÿ >•□ß±MÔ5
=#Nšúþâ;kH□t'Èpª(□Õ□åWŸ´,mn

Q.§v ãÍ,Ø□?÷Û)ý+±ð—Ž´□□[K.‹vdxqæÁ"”’<ôÈ=½ÆE m_êV:U©°Ôom¬íÁ
e¸•c@OA– TðÍ□Ä□Í

‰,R(tt`ÊÊFA□u□W□üyñî•ycuàèíïF£ku□¯+F¢"6gƒ»?Æ;zÖÏ€>3øqì<5áaeª ý»È¶°ó<~ü¿0
*¦s¿;sß□;P□gâŸ‰°„|Ia¡j0ß=Õê££Á□² g(
□Àõ□ 5Ù×□âï□xSFñvƒ§kZGÚõ;ÇU³¸û,ry□¸Pw1ÊüÇøs]7ˆ5»_□è7šÅêÊÖÖ'ù',@□#8à□□yõ
□*+Ï®þ3ø>ÇÃö:´÷7 ÞÆdŠÍc□>ÐÅrÊ

□□zž{TÞ□ø»á_□ê□O²šâÚõÁ1Áy□C&9;H$□íœÐ□wEGqq□-¼—□□¤PÄ¥ä'Û
ª□I$ô□åÚ‡í□à«+§†□ÔïUN<ÛkuØ~›ÙOé@-«EyN›û@xSTÕ-
4ø4ýie°™!BðE´3□□q!8ç°5êÔ ŒÁT³□□

’{W□ üW°ñL·k ø{^Ô□ÔâIcŽ□NøÁyW9Æq×ÚŸñ7Çúg,ô¡i□□ì‟jvóÇ□[¢□¬□

±,1ËŽ™ï^7ð—â®ƒà?

Þiú¥ž£4óÝ™ƒZÆŒ»v*€K:œä-Ýè□ém>í¯¬!°k[‹F•w□.T
□Ù€$gñ5f¹_□xÿ Iðuž›u©Cxé¨H#^A□’§ å•a•Ï½akß□ü□ k□¦I-
ÝÜ±6Éd´‰^8Û¸$°Î=³@-•ES¾Õl4Í.MNúê;{(Ó{Í)Ú íÿ ê¯5Ÿö…ðT7F□MRt□-tvÊ□ûüÌ□ô □
V¢±ü7â□□Å°oÛô[Ô¹„□® *Ñ·£)ä□_□x›Hð¦-ÚŽ³x-
Öàí\,YÛûª£’h□^Šòý;ãï,u□Am]ïìÕŽÑ=Ì GŸr¬H-ä
ê4O-èþ ñ^¥áë□¸7Zzy’JÊ¾S®@Ê0bOÞ□… u□W)â-
^:G†¼Q¤x~ö□Ç»Õ□□□†5(¥Ÿ`ÜK□9ô□°° (®S@øƒ¤x□Åz·‡lá¼KÍ-•fycQ□m}‡i

IçÔ
Ù×|A¥xgL}GX½ŽÖÕ

7?%□¢□É>Â€4¨¯(´7,□×’bÕBg-q·]Ÿ_¿»ô¯HÑõ-;Ä□dZ–•w□Õ¤¿vHÏæ□ê□±æ€/Ñ\•ïÄ]
Mñ£x^ñ§,émÍËÜÈ□`D
Xå‹dp=+™°ý |□o|m□ê7□ƒ□´En<¿¯ÌÁ±øP□©ÑT4mgOñ□•□§¥Ý%ÍœÃ)"~ ƒÈ#ÐóYþ*ñ¦ƒàË4¹
Ö¯D>fDQ(-
$„uÚ£ùô÷ □úÉñ7ˆ¬ü)áÛ½oPIžÖÔ)u…C9ÜÁ@ •:°ï\□□í□à›Ëµ,_í□5'-uÅ¸Ùÿ Ž3-
Ò»□□êúE□„o5mF□¾ÒV□,ˆ¨²‰Pã□□†□#¯□ ß□ø¯Oñ¦•-³¦Çq□»»G²áB¸*pr□#õ-Úçü□«èÚï„ì
õ□□ËìZl›ÄVþJÅ³

AùT•9□¥fx»â—…¼□qöMJñå½À&ÖÕ<ÇP□½È
ô$□ iè¯9ð÷Æï□ø‡PŽÅ..l'•,Æ/¢□®Ç Ü¬À©□è'H□ÄòÊê'¢-gc€ u$ö□ ê+Ëu•þ
°½kxŽ¡z□àÍk□ìü
2"õ□»_ ø¿Iñ®□Úž□$□□Jau-2ŒŽ 8#èÃ¦G4□»E□[PÔlô› ¯µ
˜--a]ŌK+aTP□š+Ên¿hO□[Ü˜¢MRå□Ç›□°…>ÿ 3)ý+´ð§Ž|?ãKg—
E¾□¼Xó`u)$□U=½ÆE \ñ/ˆ¬|)áû-kR2}-Ø□Ë□□Ø'
□$'Þ°ôoˆ?ÛÚe¾¥§øSÄ2XÎáRs□¸□g□¶ùÛ¶û□^mñßÇúeÆŸ{àÈà½□Œ□□É$ŒŠ!+´>
□ÝŸâ□»VÏÃ□‹>»³ðïƒá³Ô-ým'ÜÈbO+z&Xä>qÁç□□ìTQYÚÞ»¦xsJ—SÕ®ÒÖÒ/¼ï"'z □$û
Ñ¢¼˜~Ðþ
7>W'«□Î<ï³¦ß¯ßÏé^¡§ßÛêšm®¡g'™mu□Í□à•ÈÀ□py□□ ³Ep-'øÇá□
ßÉaqs=åÜGl±YFË>…‰
ŸläT¾□ø¹á?□ß-…•ÔÖ×¯þ®Þò0□'û¤□¤ûg4□Óø‡\¶ðÞ□y¬Þ$Ò[Ú'™"Â□r2□ •;úÔ-□ñ5—
Œ<;o-éñ\Emp*\(W□X©È□Ž ÷¬□$ÿ òK|Cÿ ^ßû0¬ß□¿òItŸ÷çÿ ÑÏ@-‰Eqž.ø¥áo□\}"R¼y
op µµO1Ôïr□ý □³<=ñ»Á¾!Ô#±K‹› å`±‹è,+±è7+0□êE z5□Wžø—
ãGƒü3¨Ia5ÅÅíÔGl©e□p‡Ð±!sì Å z□RÔu•3HHßSÔm,-VÛ□¹•c

}□â2k˜ð‡Å?
øÒïìzmÌÑ^í,-
®cØì□R0H?@s^)ñÇÇ°WŠ®m´‹□{Øî4›¹ã□§□UX,□åÃ□ÕOP(□éú+Î|'ñŸÃž0ñ□¾‰§Ùj±\Î®Uî!
Œ
Ú¥ŽJ¹#□é]W‰üa¡ø>Ánõ«å·W$G□□žCèª9?^ƒ½ nQ^UiûAø*æìC"êvÈN<é-Ô ÷ùXŸÒ½:ÎòÛQ³
†òÎxç¶™CÇ,m¹X-àÐ□õOVÔaÑô{íNá] ³·'âEŒ□ÅQK□2@Î□-sÞ0ø'á¿□□‹V°v°'w-
-°o•¯©□ >¤f¹□ï‹þ□ñwƒ|Eaiq=¥ãéwB8oc□d>Sp¤□öÎh□°ðoŒtÿ □hm«i•ÝEn³4%nUU·
áXŒr;×C^Oû<ÿ É6—
þÂ□ÿ è)^±@□>%ñ□□…<?u-jFO²Û Yb »□@□A#$';Ö–•ñ□û{L·Ô´ÿ
x†K□Ü*Nc· Œãvß;vßp+Í¾;øÿ L¸Óï|□□□£Q,â□$''D%v‡À;³üC·jÙøañgÃ÷v~□ð|6z'ß-²[
™

IåoDË□‡Î8<â€=ŠŠÄñ7‹´?□X-æ·|−Èä^Ó□žCèª9?Èw®
/Ú□Ár\□š□Z4Î<×·];^□ŸÒ€=bŠ££ë:v¿¦E¨éW‘]ÚK÷dŒþ`Ž û-j—‰¼]¡øBÁo5»ä¶G$F˜,ò-
EQÉþC½ mÑ^O□í□à¹.□M

-g-kÛ®ß¯

Oé^•£ë:v¿¦E¨éW‵]ÚK÷dŒþ`Ž û-h□õ□‵â?□i-□ÒÛQÖo□ÚÜ□«•K;□uTrMpÚwÇß□ßê
jï□f¬v‰î`□?Ä«□>¤P□Gáÿ ˆZO‰<Y«xrÎÞõ/4¶‵fy‵□m±ö□¤1'ŸP8®¶¼□á4‰7Ç-
□K□«ÆïrÊêr□□‵,□q^ó@□□SSÕ,tm:}CQ¹ŽÚÒ□Ý$²□□-
Ôûw¯0¹ý¢|□□í□vÚ½ÂƒÄ‵[Vúnp□J õª+Í|5ñ»Ã^*ñ□¦‹cc«GstYQ¦†0ƒ
[’®OAé]□¿ãÝ#Ãž%Òt+ä¹ûN¦q□ˆ«å§8Ë’À□Ÿ@h□¨¢¼Ý~8x:_□C¢ÛI{póN°%ÌPƒ
bvŽK□FOP1]¾¹¯i~□ÓQÕïb´µC□îzŸ@□$û

h□FŠòwý¡ü□·-P‡VtÎ<Õ·]¿^_?¥z□‡<Q£x³Mû~‹|—
PƒµÀ□4mèÊy□€6(®SÄ?□t•□x£Hðýì7•wª2,-

jQK>Á¸–□sè□ut Q\¦•ñ□HñŠõo

ÙÃx—š[:ÌòÆ¢6Úû

Ò□"Ï¨□.¹ã-+Ãþ(Òt
Èî•Ö§þ¦HÑLiÎ>bX□Ï 4□ÓQ^g¬üwðV'~Ö‹5Ýû!ÚÒYD□0□Þf\ýFEv-□ñ~‹ã-
4ßh·btC¶D`Uã>Œ§§×¡í@□"W3âï-ø□Á□FúÍÙYe□Åo□ï'Àî□aîp+™Ð¾:øC^Õí´ØÓR¶žæU†#q□
ígc…□Vld'Ö€=2Š(□Šà¼Oñ‡Â-□¾{□›¹®ï#8'□8üÂ‡Ð'BçÛ9□_Ãß□|□â□èì-
æâÂyHXÖú0□‰í¸□£ñ"€=□¸□Gâ~•eãFð□¶™ªêZª€Y,ãŒ¨ÊîÁguÆ□□žœõ-ÿ □ø'ÏÂ>□¹Öïâ¸-
Úßnô·PÎw0Q€H□Hï_6øwâf‹¥|^Ö¼]wk□□ìr,1¤heRÌ˜È. áHàš úcIÕfÔÖ□PHÔ4Ö…,í¼□üü
g*cv□~5¥Xž□ñE□Œ|=□µ§Åq□´ÌÊ«p□\□b□ 'ÛÖ•â¯□YxCÃ·□Þ¡□Ä¶Ð□

¶ê□Îæ
0 □©õ
•k□eÖ¡6Ÿo¨ÚM{ ÌÖÑÎ-$c§Ì äu□}jí|-á_‰š.‡ñg_ñ]Í-ûØj1Ê±G□heRò#□À¸□…=
í_Cø3ÆZwŽtGÕtÈn¡•'h
Ý"«n □~V#0ï@□|ñ
Iñ-ö©i¦ÛÞÅ&šÊ³□”E

IaòícŸºzãµu•ó¯Â□□é>□Ôüq¨ë7±ÚÛ,ñ [’í¾o•@äŸa]Ö□ñóÁ7úŠÚ;ßÙ«6Ñqs □þ%X•=È-ô□
ê□R++¨e`ÊFA□ Šã|]ñKÂÞ

[illegible]

□È©k˜ñ••ôï□é□ú–¡g}=¤Ò□ƒZF-å’27na€q[ZF«g®i□°¦Ÿ(-
Òê1$n=□cèGB;□@□h®GEø‹£x‡Å÷Þ□Ó!¼žk
Æk¥•~Î0p@mÙ<ñÓœ-Ü×]@□Ý]ÛXÚÉuwq□½¼csË3„E-¤ž□%•í¦£i□Ý•Ì7VÒ□Rh$

•Î8aÁä□ò□□Þ=Ò´Ý□PðŒÖ÷-¨_Z$'È'¯"□~ä°?Àz□Ú¹□†ÿ □ü9áo□é~¾²Õd»•¤

ðC□!ß+0Æ\-Œ;P□ÐÔUmCQ³Òl&¾Ô.b¶µ…wI,-…Q^euûBx*ÞäÅ□j—(

<ØmÔ)÷ù™Oé@--Es¾□ñÏ‡üilòè·ÂW‹-l

¥$□ê§·¸È®Š€
çüWã]□Á–
?iÖoV6a˜ OšY□Ÿ_êp=Mt□8885âZÏÂ□=7Â~"ñ□ˆµ+•w^□□Ê³ÌJ¤l□•UsÉ□²qè□ zoƒ¼a§xßC
:¶™□ÌP   š□·(ªû€□ð
           □äw®,¼Ÿöyÿ ’m/ý„%ÿ ÐR½□ÅFôxCZ:vÿ ·}†o³ìûÞfÃ·-ùÆ(□“ñ□Æ¿□ørþK).
n/®bb²%”aÂ0ê
□□Ÿ¡4ÿ

|eð‡Šu□ì-în,îå;bŠö0žaô

\ûgžÕÇ~Îi¡¾ƒ¨mH

¸·$È\□0Cµvíï·;°wëÚ³ÿ h+=6]c@ƒK,?øHç□,¶à □I□7c¾ï»Ÿz ú□ŠŽÜJ¶Ñ,Ì□P€9□
cŸÖ¤ □Šæ<cã-+ÀñXÉªEw"ÞÍäÇötVÁõ9aÇ=³X>#øÙàï□ê/a$÷7×□¶ÙEŒaÖ3Ü□f Ÿ¡4□è´W'¤
|Jð¦³áÛ□r□V8líp.>Ð6<$ô□{"Û□Ïl×1□í□à‰u□l[QŽ"ØûSÛ□/ëÃ□Çü□€=NŠŽÞâ□»h®-
åIa•□Ç"□«)□□-àŠç|Yãï

ø.4:ÍðI¤□£·‰KÊã×hè=Î□ tÔW•Y~Ð~
°°Xe□□¢‷□6{u(?ï†cúW§YÞÛj6pÞYO□Å´Ê-9cmÊÀ÷□€'¢¹+ß°□›ãFð½ãO□ÒÛ›
—¹□*À^□±Ë□ÈàzW3uû@ø&ÞøÛ!Ôn#□-
hŠÜy□ ™ƒcð □SªWZÆ™e{oew¨Ú[Ý\œA□³ª<§8ùTœ·>”Í+\ÓµÍ-=[L°[«)□²É□<ã¨ÇPG¡æ¾fñ
ÇÄÍ□ÄŸ□¼5â□;[øí4¹!iÒhÐHÁ&Þv€Ä-=Hæ€>©®OJø…¤ë-8Ô<%ooz·ö*í,’"□^R í!‰þ!Ô
£àŸŠÚ□□5K□?K´Ô¡š□

ì×Q"©]Áp
»s–□æþ□Õ,t_Ú□Æ:†¥u□-¤6ó——C€>x¿3íÞ€>,¢¼-?h/□5ÿ Ù‰Ô--
ØûI¶-_×□·cþ□^•gyo¨YÃwi2Oo2□ŽXÎU”ô
Ð□ÔQE □W□â□Œ>□ð-óØÜÝÍwy□Ä□ÙÇæ□>„’□>ÙÈªþ-øÛàÏ□ßGd·7□□ÊBÆ·Ñ„

OmÀ•‰□□ß^]%□□ÅÜ¡ŒpDÒ°^¤(ÉÇ¿□□àŸ□iž<Ò§Ôt¸.á†□Ì

·HªÅ,†ÈÚÍÆ□V§ˆ□äYÕ□èÎoý ×–
þÍÿ ò#j□ö□oý□□ {%□Ïx³Æú□,¬ÒãZ¼òš\ùP¢î’LuÂŽÞç□Þ¹□□ã□ƒuýE,D×V□ÈÛckØÕ□Ï¦åb
□ãŠ Ûð?Ä-'ÇðÞÉ¥[ÞÂ¶l‹'Ú�much□%³ŒmfôSÖ×„~Í?ñáâ?úí□òz÷z (¨®n`²µ–
æêhá,%/$’0UU□I' ¯0¾ý |□ivÐEý¥v ãÎ,Ülÿ Ç™Oé@-©\Ÿƒþ!i>5¾Õ-
4Û{Ø¤ÓYVcrˆ¡‰,>]¬s÷O\v«þ□ñ†‰ã–
=ï4[Á:FBÊŒ¥^2z□□ùô5â_□<G¤ø_SñÆ£¬ÞÇkl³Ä□nK¶ù¾U□’}… }□Ey~□ñóÁ7úŠÚ;ßÙ«6Ñqs
□þ%X•=È-õéêÊê□X2‘□AÈ"€8Ý#ān‡-xâ÷ÂvÐß-ý£J□$‘¨‰Œg

O¯P:Tþ$ø…¤ø[ÄšN‡}o{%Ö¨Ê°¼□…□³„□‰`G'°5ODñG…/¾$jÚ&Ÿ¤y□í°3\Þ}-4ó@*□Þ

ãË

£µp?□□ä¯ø□þ»Cÿ ¥□€=ÚŠFeE,Ä□□$“À□æ:¯Ç¯□i—
ïj’^ßlm-5¤!£Ï±f\ýFE t^4ø…¤ø□m6=NÞöfÔ□Ö/³"6Ò»s»s.>øéžõÖ×Íÿ □¼W¢ø¾?□_è·«q
□žá]qµãlÃÃ)ä□↑Õî^/ñnŸà-□µ□J;‰-
ÖE□m°†r[§□□úÐ□õ□ç:ïÆÏ□h&Õ%’îæiáIŒ6±+4Jê□□Ë

□@I®ÛOÖ¬u□□ß[Ža□„öâàI7Éµ□Î[=0:Ð□…□å°•Çÿ □ØÝ´□6¡z□àËm Ùøoe'ò®¿Â~9ðÿ •mž]
□÷Ìx±æÁ"ì’<ú©íî2=è□£¢¹□□øëJð<V2j‘]È·³y1ý□□°}NXqÏlÖ□ˆþ5ø;ÃzƒØÉqs}q□m•lc

÷< OÐš ôJ+›ðŽ´-
ÙÉ>t]âÇ«²HóÓ#ÓÜdWI@u=B'J¼Ô®wy<òlm¨¥Ž®`x#Ç°W•¬.®ô¨o!KiDN·HªÙ#
•¬Ã-
•t7óCo§\Ïr›àŽ'ynr F;ñ\¿Ã¯xoÄ°Å×†4¿ìÛ8îLRCöháËíR[H<ÏZ ì(®J÷â.…¦øÑ¼
/xÓÁt¶æåîd
°"‚rÅ²8-•ÌÝ~Ð>
·¾6ÈuˆÁÇÚ"·-_×æ`Øü(Ôèª6³§øƒJƒSÒî'æÎa"'?PAäèy«ô QE QE QE
 QE QE QE QE QE QE QE QE QE QE QE QE QE QE QE QE QE Q
E QE QE QE QE QE QE QE QE QE QE QE QE QE QE QE U{ë+mJÂâ
ÆòšÚâ6ŠXÛ£)"¬W5ã¿Eà•fk7»A2DbGÚ~c×8=hÁ¼kð[ÑåšóÃ„ê-°ƒ¥ÄcÓ-
ðäúVÃï‹šï„uh4Ýjâ{- IåMÎL-
Ã8%IäcûSŽ;ö;O•-¹µM¨\ÚÉŒù2Ú9o¦P2þµóÿ ‹/Wâ7Å
åÐl•gŽ(®°¡K°3,ÇÐu Bý¥ˆmGÃ¤A†bú¥z÷Ã/ù&^ÿ ¯ÿ •y?í¥Ë
§†.3A
KlÏŽ„+Ÿ¨ùUßüjðÖ…ðÞÎËPiÿ µ,!0-ªDO,vØÚ1œ'Žx4Á7üœ‡ýÌCÿ GW§~Ò-
ò$ib_ý%xç†õ_ã>"ª^CäÏ{«ÃrÑ€@Ü0Æ{`ŒzŠö?ÚCþD/þÂKÿ ¢ä ýŸ|5¥Åá×Í²I©
\NñùÎ(‹•µ}3Ôúþæ¿-tK=â6û(R¾´K§DPå™Iß`?RkâçÅ•ÂÚ$°

¿¾Ú□‛¦,é#g□wU`¹=z□;öÇ<oÄO□‰?□□]&   –7□ÙY#

4ƒ'□□²ÌOÓ□□í□¯$Ô□gx¯¦$Ësic3‶Ü³FOó®SöuðÖ—|5=rîÙ'¼µ•a·2 D@Œ-
□ûÞý¿□]¯ÅË□Ò¾□O§!ÊÚGi >¡]□úW‶ü□ø—§ø&â÷OÖ□’Âñ-O´Æ¥ŒN□9QÉR=9□ïž 6?hý

ÎÇZÑµ[hR)¯£-9Ê

o1"ÃS‡Æ}…kj·'_~Ê□³JIeH¡□ú%ÎÁú(®-
ã/□¬|u¯ØÅ£ù'XXÆË□¬…L®än -qò¨çž□zOŒtI|;û3Å¥Ü)IáŠÝ¥Cü.Ó+°ü
□@□¿g)E¿…<A3

ˆî□Èú&kÇ|)â□&ÛÇ©¯øªÚ{û□6K‰b‰□Ì’¶H$1 •Ç={W²~Íñ¬¾□×£q•{•R=ŠW’è†ÏÀ-
□¤¶ñ>“□ý¥¤¯oq□Ð,™S÷dUn□f- ûÐ□eñ?â‡f¼qá□±Ùi:„Zœ2#[O<□(Aœ2-
W'□gŒu□»/ÙòîY¾□êÖÎIH/$òóØ4jHŽOãJþ8ø □y‹¥é

ØÏ”°Íôæ0?Zîü□ªh□×ƒ□ÿ ÃZrXiÒ□@…mÒ˜d□U8ç□ô □š~□è:wˆ~#YÚêp
‹hãyü¦û®Ê8

;ŒöïŠõ?Ú;J²O é□,[Æ—□^‹euP□–Ñ¹ÛôÊ
óï€?òT!ÿ ¯I¿•¯Ný£ÿ äŸéßö□Oý□-
Cû=øwKO□Ë®›T}F{‡ˆÌã%□q…_AÉÏ¯à+Ê¾$YA¢|k½ŠÂ5,4»·™□□□³*9Ç§ÌI¯jýŸÿ ä˜§ý~Mý
+Ç~/□Ép¿ÿ ®¶¿ú*: õ•Ú#R–Óáý½¤LT^Þ¢IŽèªÍ•ûè)ü+Î~□|Gð□•4Y×QÒõ õiå%î
,6□< ¨
88êO□ëÔ~=èW□ÇÃ¦¸¶□□ôë…°uQ"åá•□á¸□ì□ywÂÏ□ø□MÑ¤Ó<a¢Ø5ÂÊ^+é´õ¸Ü‡+□¥²

qÛ□Ú€9□-
x‹E×<hÚç†m.,"uI□e□c"`y`□ˆ□ÂŸ®kÙ¿hiLÿ □4Y˜`É|ŒG¦bsS/Ž>□•JÒÖÇAÓ®¦žd‰□□□□+
3 .«À'·4~Òò$i□ó□_ý□% /ìûáÝ./□6¹öT}Jây#iÜ□U□ *ú

ç×ð□äß□,môO‹□ÿ Ùñ,□LW□□`+•RHúœŸÆ½»à□ü’è?ëêoæ+Æ¾;□ÉW½ÿ ®0□è□€=‹ã¶“¦ÉðëPÕ
OµmB6□□ìÂ¦U_5x□Œ□Éã=Ír?³÷‡tMkÃÚ¼º®□§ßI□Òª=ÕªJTlè
□□]÷Æè$Ÿá6±å©b†□ z   S&¼£àŸÄO□x7KÕìõË™–ÌÒ¬Ð²ÂÒ□ùpWå□□•×Žh□Ú~!Kàë□

Aqâëh¥°¶™^Þ□¤–•□□ª,3Á<-1×¥yeÇí□¡Ájú}‡,Uôò
y2L‘)_BŠŒ?
‹öŒšKè<-}□fÓæ†W□±□¹,□Ÿr¸ü□hø+â×□¼)ðîÎ□…ÓU,□³ZÅnCÏ/v2cn ç$ä

Üb€<ûà” ü`Ó

KåÇ ¸ù3œ/”ä

÷è+³ý¦?ãóÃ□õÎãùÇ\oÁ™•ÇÆ}6v]¦Srû}3

†»/Úcþ?<7ÿ \î?œt□Ý|□ð®™¥ü<³½[xå°Õ□Íq+ %•$□ÿ t□Ó×&¸oÚ;ÃB9t¯□A□
ÃìSàp□ËF□-ãð□ê¿
?ä—
ø{þ½□ó5kâ□‡□Š¼□ªé*»§’□öÿ õÕ~düÈÇÐš ó-?â□ücŒÚ‰›ý>ÚÜéyÏ>g□§ë±•¿□X¿³w‡÷O«ø
ŠTá ³€'ÜáŸôÙùšñ□ª^Ç£M£ù¤YÉp·□□ÿ žŠ¬ þL□!_b|1ðÿ ü#_□4‹□M·□□Ú'ÈçÌ□˜ƒî2□ð

°¼·ã¶“¦ÉðëPÕOµmB6□□ìÂ¦U_5x□Œ□Éã=Íz•yïÆè$Ÿá6±å©b†□ z S&€8-
ÙûÃ°&µáí^]WGÓï¤ŽéU-êÕ%*6t□□À¯Pø…/ƒ¬<9□Ç‹-¢-ÂÚe{x
’Z@□
ª□Ï□ðxÇ^•âß□þ"x{Á°^¯g®\Énf•f…–□□?Ë,¿(8<

¼sZ?´dÒ_Aákè
6Ÿ42¼mŒ¯Ì□Œû•Çäh□[□Ú□C,Õôû□□«éä□òd™"R¾…□□~□Å|□”Œ□a‰|¸ä□&s…òœ□žý□z□,¾-
x□ÂŸ

ìàH]5X Û5¬Vä<ò÷c&6àžrN@íÆ+Ïþ

Ìn>3é³²í2›—Ûé˜d4□¥ûDÿ ÉG·ÿ °l_ú□•ï^□ðŽ‡¤x&ÓM·Óáh.m‘®|Ä

gfPX¿¯_Ãµx7í

|I¶ [illegible]N•[illegible] [illegible]¿%wž[illegible]øõá¸¼
êâ{}RÒ[illegible][illegible]¶Ž&a9Q€Q°

ãø^Ç½ y?†âÿ „cãµ¥•¡`–ÚÙ²Cž|³!‹ÿ A5ÝþÓñùá¿úçqüã®3á•□ßŒ¾2[êoÝ»}Nå—
¢a‹•ü|¨ük³ý¦?ãóÃ□ðÎãùÇ@□Z–¥.™û–Û¼

VI¬¢ ·ÈþëÈ□¿5,?□óO„þ6ð ·□ç¾¾ÖôëÛ«ù6–¼□C□ˆ□gv78Á'□;
õoì9üCû3ÛXZ!’äX$Ñ"Œ–(ûð□rB•>µå?
|Qàí

[Û?□hÖ—PÎUá°šÉn

Dd□ ,pxéÜ{Ð□_Š¾.ðßŒõ ›MOAÓî-&òÙ.Ìñ"y§#iùXäõ□Ÿjú{ÀWrß|?ðýÌä´¯a

ö=X„□?Ž3^m}ãï,v'o‡CÓo[û□hŠ□þ>Š?ZöK3□±·6¨±Û˜×ÊE]¡W□ □N;P□õâß□>□xƒÆþ,M[L¾
ÓÒÝmR.êGV□□'□Q†9õõ¯h' ŸOJò□□ö□ð†¡ : °Ògyd‰¦OÁ•□□□(□Â5_□kÿ
üGi.«¦YLÿ ~□:   íæÁ□ÇÓŽ8##ÔWÔß□¼i□Ž¼+□-□>DÊæ□^AÈÏ □àú□A-
ZðO•_□4□□¶•a¢   %·³g'îdŒ¦ö`
□8ãœ□úW§|□Ñnt_‡rÞ^FÑý¾á®cB9òÂªƒ□}¤□b(□Õ¨¯
ðWÇ]_Ä¾;³Ñîô«□¬o%hÐÅ¿Í□,FI$7Nx□îÔ WË>□ð•ÿ ‹þ+x¶ÒÃÄ□:,•ÜÜÊÓ@¬Å
ÇŸ•§

¼sž½«êjùÏá¾»¥h-
□üg>-¨[YC$·(□<□□7Ú3□žø□□Øi_□¼A§k□WÒüGÕ.#·•%x□) •+□TæcÁÆ:-½+Óu□'N×4™ôíZ□
žÆP

±»□□)

2A□,□ü+□þ□G,¿èhÒ¿ð%□Æ¹oŽ7×R|({□"c%-ÄÐ™¥…²□Ý□9Èþ□v}A □□ø…ðËÃP□6□SL,□ùL6v
æDÿ ÈjA¯3ð□¡£ÉûDÞÉá‰□û□ò□6yq´jA□]€V €□-1ÛŽ+wÀ§á□Ÿ,,.oN†÷¢ÝMàÔBI7›□œ□|œg
8Ú:b¹Ï□ê□F«ûDµö□f¶šT±Iöh’□
áa
X(à□ÊÇñçš ï?hOù&‹ÿ _ñ□&®ÛÀ□ðO<5ÿ `«_ý□µÇü~¶-ãá„Ï□□X.á‘ñÙrW?›
ŸáçÄ?
Kàß□é-¬[Ç¨}ž
²±>gš @1Ž„Ž½9

Kã□ü•ÿ □□×h□ô W¢|Wÿ ’[âúöÿ Ù…yßÆù+þ□ÿ ®Ðÿ é@¯Dø¯ÿ $·Ä?õíÿ ³
 â~□øCÿ „FßÄ·6‘Ýj7O
W•C□U\®□-„ã9ëÎ+□ãž•gáÏ□x_Ä□\□ÚÝË3□L*□3FÈÊØ□þb3ì+Ð>□ÿ É%Ò□ßŸÿ G=q¿´□_
□×iÿ ö• v□□àøW¨ý“~Ó$^~Þ¾^ñŸÃ;sV~□/‡□áŽ™ö□da6ªo‰Ûþ·-
¼ó3þÖzöÇlU/Ž’êÑü3¹□ZHQçE¼1ŽVß

XñÛpP}‰í^Yk¤ü□Ô-m&›]Õl'ò“Î·Ãã~□rLl99èØô

“àû[ÿ ÂàñxÐqÿ □þÇ*#ÿ W»Í□1Û□ó1í^ó\OÃ¹¼□o§>•àË»9□~òTI

™ÏMÌ□æ?Èv®Ú€3<Gÿ "¾-ÿ ^S□è□¼»öoÿ ‘□Sÿ °›□è¨ëÔ|Gÿ "¾-ÿ ^S□è□¼» öoÿ ‘□Sÿ °›□è¨è□—í(HÐô□8?i“ÿ A□è¾□ð

•á¿

Á¦Åamp^0n&ž□f□^ä¶sÇ ì+Î□i_ù□h?õó'þ,+Û-ÿ ãÚ/÷□ò □□ý õÄ]wÃÚ□ÛL°P□îí
ÆçRûx□••ªøÏsZ□□>□éúbé¶ž□Ôá²UØ [H6'î<Î□□ãÆ™{§kž□ñ-¤□hôé□&ÇD+
t'Ð□¸géë^…¥|Rð^-§Gx¾!°¶Ü¹hnçX¤CÜ□b2~™-"□ä?
u»□¾5êCÃv×□Ú-£□°¶'Bùx□²B' □¸

z6*÷Ä□?á5øÿ ¢øVñØéöÈ»â□□¾C3ý
(
Ÿa^¯áÿ ^^□ñN½s¤h·Ow%´>t"¤dE□À`1êyì1ï^Oñ9¦ðGÆ½□ÆrA$š|Á□Œƒº©□×ë°,=□
öMCÁž□Ôôs¥\è¶FÏnÕ□!UÙî¤r§ÜWŒü□ÓbÑ¾-
ø³K□Ýá³I-ÑŸï□IÂ,}ð+Ñõ/Œ~□°Ò□üzÌWlW1Û[,esØm?wþ□Šóo,□ÒêŸ□<W¨OlÖÓ]G4Ï□g13N
SŸLâ€/|Zÿ ’ÙàOúímÿ ¥5îõàÿ -□ãGñ‡„¼R!i--%PØé°9□•I÷□ÇÐ×w{ñ—
Àöš/öŠk pY7%¬*LÌ□ºWøO×□€8_…_ò]¼wÿ]n¿ô¤V7Åß□éò|bº²ñ□w□□□’^ÒÚÀ□23.óÁ
s”□‘À«Ÿ□¯fÔ¾,ø«P¸µ{Yná-
á |æ=ó+c•=j□‰kqà□Œú?Ž^ÞItÙ,¬Îƒ8`†7_®Â□Ï^}(□B_Žß□'ÓN›'‡u&±+³ìæÎ□/-›|ÌVwì
÷©+x‡Äö□zÎ4v"âÝ%ë□ÞBƒ,@b¤gŸáö¯Tƒâ_,®,…ÚxŸLXÊîÛ%Â£ÿ ß□□ŸlTþ□ñÆ‰ã)u□Ñ%šx¬
]□æhÊ#-□ü¹äôî□ x·Žô
_□~Ñ¶z5ëÈ¶·1Gæùg□ªÆÏŒöÎÜ~5í—
>□ðÝÇ‡dÐ†□g□‹Fc□□¨)ÇÞ□®á×=s^S«□ÉÖé_õÄ□é;×»P□‡~Í·3□dx,ÁÜ˜ ¹ŠE□ƒ2°?ú ¬Í>Æ
^□´V®šÂ
‹□ H□ÞNP^™c
G¦æ,G~j÷ìßÓÅ_õÚ□ý«Y—□€øYñÿ PÔµHä]'W□7žªN□F

X□×kŒ□×†@=ÃTð—‡õ□<ØßèöS[‘€¾PR¿î‘,§Üb¹ÿ ‰Vpiÿ □µ‹+dÙoob±D¹'j©P□O°¨ußŒž

Ñô¦»ƒU‡Qœ¯î--NçsØî•súô¥ø□{.¥ðOR¾šÝ-¥¹Óc™àc"□6ÒTôéœ~□□—
ðšÿ û+àL□ŽÐßd†î|-û^Fþ•ä¿□þ x[Ã—š-³â}>ÿ R×nç.·)

r□SÉ ³Œ1bs•Ð

z×®ü"±]Sàm¶žíµ.£»•• i$_ë\OÁÿ [illegible]Yø[illegible]óXð[illegible]$¤[illegible]lÂèË[illegible]³[illegible]~Ð¬ ì[illegible]U
ô#¿L€düOø™à¯[illegible]øu¡³Ò5[illegible]µ^ŸZÞêh"\

üÊÌ®N
çŒ-@¯Sµ‡Wñ‡À□à□ŸûNïKòÁsƒ)^9'ûÁzÿ µZÚ¿Å_□èö□q/ˆl®H□X¬ä□»-
L.qøàVž-ã□7Eðzxžñ.EƒG

„‚y‘VB dg¶áž}zÐ□•ü*øƒá¿   hÃÂÞ#²}R†W□M5¹Ä¤±#yÆA •ÈÆ æ½¿J□J□~~Ž–
bÖå¼ï2Ð(II nÊðO□Ÿjáõÿ □ü+ñ^†Ók□¦□u□Ã´»□pƒý‘þ°`+—ýœ¾Ùý™â

4ÇH□(-<Ïïa·ãß-^h□Û«Â>:]\ë^1ð·,ã™¢·»’9$Çv’O-
Iÿ w□ù×»×…üxÓ/´Ï□xwÆöp™SOtIHèŒ’y‘çØ’Ã?OZ õ½/Âz□□¥Ç§Yi6‰l‹´©…X¿»□2Äúšðÿ
□é°|:øÿ Ï¢
¶²ÔÚ/2□øE□9□Ô□NŒ□cÓ ¯QÓ~/x#PÓ□õµÛ{RW/□Á+"-ão□Ã5æ1]Ÿ‹ □□´ýKL†S¢h¾[□‡R¡„l\
□;□s€:àg□□ ìÿ hù&-ÿ _°ÿ ìÕ×ü>ÿ ’sá¿û□[ÿ è±\‡í□ÿ $Å¿ëö-
ýš°ÿ ‡ßòN|7ÿ `Ë□ý□(□¤¯%øýáí[[ð•”úd□\¥•Á–
â□Á,T®7àuÇ=;1>µëUÍx›Çz□„5□:Ó[¸’Ø_‰<©ü²Ñ®Í¹

G#;‡lzâ€9o□|Oð‰4Û}&HítÉJˆÿ ³îâU•=6©ÆÒ;

àŸJèü{ª
ü7Õ¯4äKvµµ□[□”(ˆ±□»@àcpÀö¯6øÁwðÛUð-Õõµæ•>°v›i,$V•ÛpÎý•F3÷¿

k Ñ4-[Ä¿³´ZMîó¨\X·’%<□®Z□IõPƒé@-kðÇâ?,|□¢"¨i:□Æ¹4ŒÓÝÇ□OÆ~UVg□

`ž□$õâ©üOøƒáo□□?Pðö•□c–ÚÎ□ÝI

qîLdrŽI`ÁHÈãšî>

üDÑ´o□□Â+â
´Òïl&•'Úÿ v¬¬Å^,xV

X□qÛß□æ±ñkÁz@E□Ì7óHÁR□<‰Ù‰8ê§hüH □>!\ÉyðWS°™
K6œ':'□¬v'1øÖWÂkÿ ì¯□0j;C}'□¹ð{íy□úWAñ_þIo^□ëÛÿ f□…ðŠÅuO□¶Ú{¶Ô°Žî□>□¤'□
- y□Ã□-□ðåæ¥¬øŸO¿ÔµÛ¹Ë-ÊC□•□òH,ã

Xœàt□žµ£ñ?âg,¼sáÖ†ÏHÔ"Ö#uk{© ‰p3ó+2¹8+ž0y□µ¾□ø°ÏÀ□šÇƒüU
Óf□FXå˜□›ö…`O`B©□¡□úgÔõ□$¾ Ñí□â_□Ù\•2±YÈ'v>˜\ãñÀ

<S¨ÙþÎé¯;È°€ÓR1+pÛ™„Aþ¼†ÍQø□àý.□□Á¯ÜYÃ>¡¨I#yÒ f•□Ê□\ôåI>¹ö□Øø ËOo□|/¿·±Gß□d³ÛÇ Ã□ùdE>„□□yÇÁ¯‰Z-‘áUðÖ¿xšmÝ„²□ÍÀ*®¬Å^'³□,0qÛß -´<- áôñ□¾½□•o

§ eIá]„†□□À`7□òs^IûK□È+ÃßõÞoý□k»°ø§¢ëž7³ðÞ„¯¨‰□G¹¼□"(□©#□|Ù8□éÈäô®+ö”¶•
ü?¡Ý*□□WR#·¡e□□è&€=Àt□ó□^ü_¢·Ç=GPñe¥Íþ™¦3[[ZD‹

„(Ê³□·;ÛëŽ1^í¡|Cð·ˆ%°µ°Ö–å½¼•r[);Á
¹ƒ

p@□¯¥y□íçü*ß□ ·š¾©□‹ £ë
çí
¥€Y
³-:íuäuÇ=Å hk□□>-kú,úUöƒ«Io$e□□X□wÇ□□yÁ□ˆ«ß³Ž¡uqá□NÊbíokv

%°□ë-QøŒÿ À½ë¾"âO,£³ûSx£K1ã8[…gÿ ¾□ÍúUß
ø³Lñ-—.¥¤ùÆÕ'h□Ë-Íå@9□®9ïfí@-+ð³Lµñ÷Äÿ □x—Z‰.Å¬¡¡Ša¹T»0N□]ª˜‡¥z'Å-
□h□Ï,õ[é´øb½°³—æ
ˆ□#,ˆX) r§□Áõã□¼ËÀÚÕ·Â¯Š~!ÑuòÖ¶W□ˆî□IP□□□□□
V<ö={ãµø‡ñ[ÃòøjûCÐ§þÙÔµ+i-Ö;@YcVB□Ù€Ç "□žœàs@

ýž□ä›Kÿ a □ô□¯X¯'ýž□ä›Kÿ a □ô□¯X □,ý □ä˜·ý~Ãÿ ³W_ðûþIÏ†ÿ ì□oÿ ¢År-
´□ü"□ÿ ¯Ø□öjëþ□É9ðßýƒ-
ÿ ôX □-¶²‹âOí□ªA«□?NÑÄ□m˜ü…b`□~…Ø±□ù□í×¾□Ðu□5´ë--Éí□vù^B€£ýœ

□¸é^□{|ß
¾=ÞêÚ”2□djâF3¢“òHC1-¥\r:ãê+Ô¯þ.ø-ÃM{Ñ¯[Üar°ÛåäsØ□ì~¸÷ □9ø,óøoâ□$<-&w²•Í
t

□Š9□†ö%[Ÿ ôªöÖQ|Iý¡õH5qçéŰ8•–³-
•¬L□/Đ»□#¿"´þ□iwúÇŠ|AãÛëv,+ö’;pz6ù7¾=BíUÏ×ĐÖEíó|*ø÷{«jPÉý‘«‰□ÎŠOÉ!

Äz•qÈë•¨ □r½ð¶ƒ¨é-§]höOhË·Êò□□-
ìàp}ÇJñŸ,Ï?†þ'ø£Áâg{(ü×@Çø£•(obU¹ú□Jôkÿ ‹¾□°Ó^ôkÖ÷□\¬6ùy□ö□{-
®=ë„ø!¥ßë-)ñ□•o-Ú□¯ÚHíÁèÛäÞøõ
µW?_C@□~ Aÿ ¯Çý□Â×ŽÇO¶Eß□8□ò□ŸèY@\û
ö=CÁž□Ôôs¥\è¶FÏnÕ□!UÙî¤r§ÜW□üOi¼□ñ¯Eñœ□I&Ÿ0A# îªcuúì □_Â½□RøÇà‹□
ßÇ¬ÅvÅs□µ¸&W>›OÝÿ •b€<ßàž›□□ñoÅš\

ï□šMnŒÿ xªN□□ï□_BWÏŸ□îî5□‹>*¾°µ{Iîá–áà|æ2ó+mçžõô□ xÏí-o?á

Ó□[þÈo¿□·¦v□¹ÿ Ç¿□W}`¾□O□Ä :iðà¶□/·Ê)Žw□µëžsï\'í□-êh°□m□ÃhMy•HÀ9À+°®_□ã
{W□°/À›«¡p¾"Ô F9û)□□_l˜‰ÿ Ç¿□ é□gF”ÛxœBdþÉ□Qý”?@Ø}ßŽß/?…eüx°þÕø‰áM4Êb□h
¶æ@2W|»s•Æ½{Á7^□m□,ü!qdö6ýc·|²“ÝÁù²}[“^]ñ‡þJÿ •?ë´?úP(□Öô□□è:-—
m§Ùiv«□¾ÖBñ+1qÈrHÉlóšñ¯-
FÞ9øû¤øFîGþÍ´Uß□¶□~ïÎsìJ…\ûWÐ□ó÷ÄÁsàO□□WŽM´’éÓí□²

„!•×ë³□g¯àh□Û□Ã:□zwözèÖ□Ïnß#ìé³LW‡øVÔx□ö□¹ðþžÌšeú²^·d□1yª?à,
ƒ×□õ¯Q□¼
tï¶ÿ ÂEj□nï/æó>›1»?…y¯ÃÔ¹øƒñ§PñÐ¶'□*ÓrÀÎ1¹¼¿-□ë·,qœ□zŠ Ÿâ×ü-
Ï□□koý)¯w¯□øð—□?Œ<%â'
Imi*†ÇMÑÈ$
O¸Î>†»»ßŒ¾□´Ñ□SXK,É¹-
aRfcýÒ¿Â~¸□□Âü*ÿ 'íã¿úëuÿ ¥"ªüu±MSâG„´é$xÒíVÝ□:¨y,'?□Lø%{6¥ñgÅZ…Å«ÚËw

·□□ç1ï™[□□ëKñâöM7â/„ï¢€Ï%²¬É□ë!YA
øã□□ì°W,ü9£i□K³ÑìÅ®Ý®-□s'©rFXýkÆ<□j¾
ý¤/ü;a¹,.ÕÔEž□˜¼õð□`-zF›ñ"Á□†Ž/åÖ#³p¹'Úà□*-à >oø

kÎþ□›•□ükÕ¼n°I-Ÿn□DÎ;”□"ývdŸOÄP□¿□à¹ðÇÅí3Æzž•&§ $hŒ□îXØ+.

x□□¼g,{úzg‡ü_à¯□4□Âk+›¸H•-îa□h™y□U†r8ås□Z[¯‰-□·ñ□ÿ ‡5MB;+»mªâð□Š@È□†<c

□8ükÄþ(¯…□á*Ðåø{-±×d¸ùÆ–ÀÇ»+°•¿(lçîþ4□ôÝrÿ □µ¹ü;ð÷ZÕ-
˜¥ÄPl‰ÇTw`□‡¸,□á]8Î□z÷Åsž?Ðeñ7€õ□"Ü□q<□‰IÆçR□GâT
à>□x/JO□Åâ;Ë8nµ□ù$e–d

cEr˜\ô$©$õ9«□|□¤Ýø□ï[,Ê□5□□Ž&‰□™□°VVÇQƒ‘ŸOsX□□>%hÚ-
†ÿ á□ñ□ÈÓ.ìf□Dn□ª²³□*Oð°bÜ□v÷©¾.üKÑµŸ□?…¼7sý©□˙È‘±¶RÊª□6□þ&$□□žþÔ□ß|(Ön
5ß†z5åÛ´—□6…ÝŽKynP□{’□W□ø□þNSÅŸõÆ□ý□□z•Ãß

Ëá_□é:<øûD1□˜□œHì]†{à±-
…yw•?äå<Yÿ \gÿ Ñ‘P□»ÑE□□á?□?äã¼eÿ \n□ôtUîÕóÆ‡-Xø?öŒñ,°äëg□ÚË□Ë/
7´r)'°!zû×°è¾ Ò¼Gi-Ö□}□å¼R˜ZH³´8 ‘žü0éë@=ü
ðn‘â□□x†óW€\Ç§O°;w□•ÙÝùaÜ□½:sÍz□Æÿ

i3ü6¼Ô□ÆÞ+½=¢xeŽ0¤□ê…r;a•- W;ð□þFo□ÿ ×hÿ ô9k»øÍÿ $—
^ÿ r/ý□”□□ÃýnX>□iú¼çÌ{=:Vù»ˆ·€?$□â□□þ x[Ã—š-³â}>ÿ R×nç.·)

r□SÉ ³Œ1bs•Đ

z×³|.±]Sàv•§»mK«Kˆ□ú□’EþµÀ|ñuŸ€/5•□øªA¦Ì.Œ±Ë0!7í
ÀžÀ…R□B;ôÈ□OÄÿ ‰ž
ñÏ‡Z□=#P‹X□Õ-î¦,%ÀÏÌ¬Êäà®xÁä
÷O‡Z…Ö©ðïA¼½.n-ÑC³õl|»•Ô

þ5SWø«à•-Ñ®%ñ•É+œ,wcé…Î?
êtûØõ-6ÖúuŠæ™Æ €GcÍ Y¯ž5›ƒðëã†¡â?i3^éWû¾Ív±‡òó·sÆå
W#ßèzâ#ø•à•VçQÒ5FÖÚKiä¶žßS
¹F*H-ò°$zçÔ
 Òðÿ ˆ<%ã cÔ´‰ì/.-Á!¼°'‡#†—
'ž‡šó/ÚWþ@Zý|Éÿ ŠçnãÐcøïáßøW'!Vš3x,É0¸ù›{mòó8ô®‹ö•ÿ •ƒÿ _2ê"€=wTÑ
l¼Eá©´•B?2Öê Ž;Ž8#Üî+æ´ñoˆþÚx‹ÀG2Nòm²¸Sþ«~2ëþò•@ìß•}
 âÏYø/ÁÒëxfHÂApe"•Gó>€^)£ü*Ô|sà}[Åz¬ŽÞ!ÔßíV!ŽQ",=-
 ì SÒ€=ká£ð7…#¶•TêwXšöAÏÏŽ-
E}r{×m^]ð[Çoâ]´]MØk:PÈ$á¤ˆpçœ•°Þ½F€8Ÿ‹¿òJuÿ úà¿ú1j¯Á/ù$:-
ý¼êù+Câ¥´·
üA(YÅ©|E`Çô¸,?ü-¤|4²ÓõMbÞÎêÎIUâ"ÌFpTc$a»zPŽ—W:×Œ|-
à¸æh-îäŽI1Ý¤"ËRŸÃ~uëÚ_„ôK•N²ÒmÙiS
±v$e‰õ5äŸ-4Ëí3Ä-ñ½œ&TÓÝR:#$ždyö$°ÏÓÖ»Í7â÷,51/[]·µ%rð\²!î6÷ü3@-]â6
‡_ôôDÖZ›EæA-
¢G1°éÑ€ìztôM|óÙø·ñËOÔ´Èe:&‹å±¸u*FÅÁ#±g8®qÁ¯¡¨ ®sâü"Ÿÿ Ø2ãÿ Eµt
uç^9ñç„îüâ
+i²ÜËa<QÄ—
Y˜¡ žNh3öyÿ 'm/ý„%ÿ ÐR½JîîÞÆÎk»©'xPÉ$Žp@É&¼Oàw‹ü;¡øKMWZ±³¸7Ò8Šy,¶Ò
©ƒƒÛƒ]ßÅKK-oáN±-•Lï,1ÌžIÝæF\íÇ\¨=:ÐŽK¢OñÇRë

4é´X □ùÚ«ÎaŽI:îUQ•'Ðg9□□š"Ã—S|,ñÛOñ□E{›«Ç>V¶diŠö,¹á½ú8□§8®Ãà×Ä□
Ùø

ËF½Ôí4ÛëF•H·N"Y79`Á•□,□\ñéŠÍøáã_

ëþ□µÐ4{¸u]FK´‛>È|Ñ-□□0à±Î -¦€=7Æ?□t□□i□z¥üwWV×Ž□&³U|åw□’Àc-õÉY~Ðž□¿¿¶³‹
OÖÄ—□¬JZ□°
□□q!=û
îü□¦Üèþ Ñ4ëÜý¦ÚÎ(å□çk□□_Ã§á[´□áß´¯ü€´úù“ÿ A□è¾□ð□‡|5áè¬-
ôëyÌ‛□´M<Jï9#□ÄŽžÝ□yÏí+ÿ ¯-□þ¾dÿ ÐE{u¿ü{EþàþT□ó‡ü□¤j?□µo

\#•&ÒY.□²1□À ª□è7ã×□Þ½sâ§…4Yþ□ê,=6Ò□±·ó–š(U

Epp¸□□21ï\o„¿äç|Gÿ \eÿ ÚuéŸ□ÿ äšx□þ¼$þT□‡ð6î[¯…Zh•‹□d-%'û¡É□ðÎ+Ï~□é-
¿□>#ø‹Å□ìKx-Î-□□□È
- Áà…TÀ□Ô□îþ□□É,µÿ ¯™¿ô*óÏ□k0ü!ø•®è~
□o§Þ°òîv□0¬Æ7ãøJ±□□Áú□ {□ü□¡x—
EŸM¼Ó-€t+□©□‡…±Ã)□□ýzW˜~Îz¥ÓiÚî…på£±™$^□□»÷□□Û(□Ôšë¼Iñ□Â:6‹=Í-
-o¨^l?g··;Ë>8ÜG
3×?-`þÏþ□½Ò¼9□ßÆñË«H•□¸Á1¦pßð"Íø {Ð□'ã½□×Å´mž•zò--ÌQù¾YÁ*±³ã=³·-
•{eÏ,<7qáÙ4!£ÙÇbÑ˜Â$*
q÷•ë¸uÏ\×”êßòu°WýqúNõîÔ□áß³mÌßÙ- °w&(.b‘G`Ì¬□þ€*/‹ò\<□ÿ]-¿ô¦□Ù¿§Š¿ë´-
ûV¢øÝ?ö?Äß□k—
ßc·xÝ˜

ÿ «˜;□®□ □{¯›tï
iÞ/ý¢uû-UZKKy%¹h□À”©@□ŸO›?…{®□ã?

x†øÙi□½µíÂÃç²BIÂd

žÃ–□uæ¼›Àßòr¾+ÿ ®3ÿ èqP□}ñ
Â:□ïÃ•^□¦ZEöK)'¶1B¨bdRÃn□□ŒcX□u□?
•§ghì®¦E□’□□|□Å□wž3ÿ ‘□Äö□¹ÿ ÑM^□û;□É8¹ÿ °”¿ú□t□□ü4wƒÿ è□®□ß^¿øímë□-õ-
ƒÚ·‹tDº·□6†□p¡]□¸Œ7□Ž
dsÚ½□¹¿è2ø›ÀzÆ‘n□¸ž

Ä¤ãs©

£ñ*□ p□¼□¥'ƒbñ□åœ7Z□ü’2Ë2□1¢¹L.z□T’zœÕ□Ž>
Ònü□w-Áe□□□□G□D□L^X++c¨ÁÈÏ§¹¬?ƒŸ□´m□Ã□ð‹xŠäi—v3H"7
UYY‹□'øX1n

;{Ôß□~%èÚÏ†ŸÂÞ□¹þÔ¿ÔdHØÛ)eU

□ ÿ □□ ÀÏ❏j ë<□¬Ük¿□c¼»v’ài÷□»±Éo/z□OrBŠÀý›ÿ äFÔÿ ì$ßú*:ë<=áÙ|+ðthóãí□é
Ó4À□âG

ì3ß□ˆü+"ý›ÿ äFÔÿ ì$ßú*: çíì!øƒûGêpêÊ.,t•}¶ïÊ□ˆª□#Ó{□#¿5ëþ#ð□‡<Q¤>Ÿ}¦[Û
ˆ¦†5I!=Š□8út=ÅxÝõúü1ý¡/5MM□4-U]¼ð¤á$Á'□®Ù□'×□úW£x‡ã'…4-;~™|šÆ¡/Ëmgg-
gsÐ□□Ê3•□@h□‹ýšF,<F?é´ÉëÝëÂ¿fØÞ+O□Ç"”''…YHä□"½Ö€<[ö•Ön-<3¥i□;*j□

ÒàýõŒ.□ûnu?€¯EðÏ,tO□x~ÛK·Ó-X¬ag•¢ViŸ31#œŸË¥p´?‡îµ?
Xjö±´ŸÙ"1˜(ÉXÜ [è
¯ç]□†þ1øGUðý½Ýþ±ocx±□´ÛÎJ²¸□íþðÏLgóâ€:½□ÂÚ'‡no§Ñôø¬Úù•§Xr□Šç□^‹Ôô□¼□á□ƒ
t□□xëÄ7š¼□æ=:}ÑÛ¸ÌnÎïË

àméÓžkÙü□ãëO□]êçM´™tû□Ž8®ä□ûC0bØ\qŒ

¼üÃ Wœ| ÿ ‛›Æ¿õÚ?ý

Z è¾7øcIŸáµæ –6ñ]éí□Ã,q… □T+‘Û

xõ□°„×’ß|,ðüÓ1g□æ,ŸDvAú(ªÿ □¿ä’ëßîEÿ £’□ƒ_òIt-
÷%ÿ ÑÏ@□'•¿äå|Wÿ \gÿ Ðâ£ã□ü•ÿ □□×h□ô Qàoù9_□ÿ ×□ÿ ô8¨øÃÿ %□ÀŸõÚ-
ý(□□Ø|qÖ'Ò>□Þ
w)%ì©hX-B¶K□ÅT□Æ□ð›ÁšF‘ðÿ K¸66òÞj□ës<ÒF□›xÜ□'°□

}}iÿ □4□•□á-ôv‘´·□Ž—k□Œ-
•Øÿ €–?…`ü/ø«á‘à]?OÕµH,/´øE»¤çhu^□”÷ùqÇ\ƒí@□OÆÿ □hÞ□ñ.ƒ□¤Z-¡Ôe□:□Æ#

•–□Wøs¿□8ãë^‡û@□É0“þ¿!þµæ-
□üsgã}sF:E¼Í¦i×□□ß:□Y¤r„…□°□:óÏAÆ}?ö€ÿ ’a'ý~Cýh□×Â_□h°O•tûãi□Íî§n—□Ï4a›

2□g¢€qïÖ¹ïÚ□V}#Á°^‡b□½½ìÄ:D6¯—□□L

ƒ,§ì×¢ü=ÿ ’qá¿û□Ûÿ è±\/í□áË-_Â□š•¤M+i“3Êª2DN fü
®}²{P□'‡~3ü:ðÆ‰□—§èZ¼qG□Wak

éN9f>g$û×7 ø£H¿ø÷¤ê~□±º²³¾"□«y#TÜÌ□9ÚŒFÜmo¨&½{Â_□¼+®èV³ÞkVV□Â5□□]Ì±□p9Á
l□□ä□üëFÃâW…õ□□[ø□K¿7÷"+¶ûuÝ□□RN_§nÙ □<ý¥□ä□ ÿ ×ÌŸú□¯Eð□€<=á}□+;]>
]ã-}Äñ«I1#•Äöòè+Î□i_ù□h?õó'þ,+Û-ÿ ãÚ/÷□ò □□ðÝ„-□ý§o4□1□□W□?î"
…
Ð‰v•è□p+è
ð'ÿ 'h□è□þÚW»P□o^□äYÕ□ëÎoý ×-þÍÿ ò#j□ö□oý□□z—^□äYÕ□ëÎoý ×-
þÍÿ ò#j□ö□oý□□ rþ;Ð-|QûFÙè×¯"ÚÜÅ›åœ□«□>3Û;qø×¶\ø#Ãw-□"B□=œv-□Œ"B §-
x-»‡\õÍyN-ÿ '[¥□k□ÿ ¤ï^í@-□û6ÜÍý'â
□rb,æ)□v

ÊÀÿ è□½Æ¼'öoéâ¯úí□þÕ¯v □Š(□Š(□Š(□Š(□Š(□Š(□Š(□Š(□Š(□Š(□Š(□Š(
□Š(□Š(□Š(□Š(□Š(□Š(□Š(□Š(□Š(□Š(□Š(□Š(□Š(□Š(□Š(□Š(□Š(□Š(
□Š(□Š(□Š(□Š(□Š(□Š(□³õ-□Mñ□—.™«Z%Õœ¸Ý□□9□,□äqZ□P□"\~Ï-
šbñÍ«@¹Ï—□Â□ÿ Ç□ŸÖ»□ ü;ðÏ,ËI¤X□¹aµ®fbò'é"Ð{

WSE gk°

™âM&m3VµK›Iz£pA□□#•G¨®□Nø□à□?R[ÇŽúñU·-
½ÌÀÅŸp□□=‰#Ö½BŠ ægø}á[□□Åâ)4ˆÎ«□#$âGP¥   ò□ÛÀ tí\□í!ÿ "F—
ÿ a%ÿ ÑrW²×7ãO□i~;ÒbÓuY.£Š)„ÈöÎ□ƒ GpF0Çµ yGÃ?‡>□ñÇÂËFÕ-□nc¹™RêÝ¶J□wLà,=
ˆ8í^‰á□…-□ðeçÛ¬-
æ¸½ „¹»pî€ðvà >¸ÍmøOÂ°□ƒt□ô}1§{tv}Ó°g%ŽNH ~•¹@-uñÏþI6«ÿ] ÿ Ñ©^kð_ÁZ-
•<□¬Úk6žg—x†)□í’"S□-ý

Gµ{¿Š<5câï□\è°‹L¶×□K4,□ÁV

□$□ÔzV□,|□¤ø
O¹³Ò¥»•.$□H÷.¬Ù□□î¨□ü(□□Ã_□¼!á•N=F□n¯n¢mÑ5ì□ÄmØ…U□#±
â»=kDÓ¼E¤Í¥êÖÂæÊly‛□+œ□G*Ā□□ÐÖ…□□‹á¿   h~□´–
×B°□□Ìûä□GrÍŒg,I¬ÿ □ü;ð×•6É«Øæå□j\ÂÅ$□Ó#¨ö ×UE y$□ᵝ·ƒ#‟s]k□

ýÆ¸L~ˆ□ë^• øwLðÖ‰□□¥Ûù6QnÂ□,IbI$žNI-J(□–Ð>□øOÃ□¡Ô´m!mo
”ó<é õ 3□?□Z^"ðÆ□âÍ9týrÈ]Ú¤,UBì˜p□□□ ô'¿z×¢€34□i^□Ò×MÑí□-¢±q□vnOS–
$ŸÎ²5o†þ□×5Ó–êZ:O¨’„Ìf‘rT ¹PÁN □Gjê¨ □eWR¬□)□ Œ,+Í5Ÿ•- ÕîÞå
»ÓÙÎYl¦
™öVV□è0+Ó(□4Ñ~□ø7EÔ-ï•/îæ·‘e•í7 €Êr

□.y□□v~#ð¶‹âÛ□±×,EÝ²H%T22a€#9R□BkbŠ ÍÐ´□/Ã:Ziš= µ³F,±†fäòNX’□Ç×>□øGÄš¿
ö®¯£¥Íî□L†i□!zd+ □□]U□□□Õ¬□Ö“Z]B‟[Ì†9#q•u#□□^_'ì÷à©/Lêú¤qîÏÙÖåv}2T¶?àUê
ÔP□6±á]□^Ð□DÔlRk□UXã$ƒ-Ñ…*ÝA□½q:_À_□iš’^´w·¡□rÁw2´@û€ Ÿ¡$W§Ñ@□ÝŸ€|/§ø™¼
Gk¥$Z³31œJýX□Ä.í£ ž,¤ñ7,|;ã□³□oi¢ìŪnò□šñ•ÝŒòŒ3Ðu®,Š «¦é¶z>›o§X@
´·A□Q,HU□²y«□H‘FÒHÁQ□fbp □M:ªjš|Z¾“y¦Îò$7p¼□4Mµ,°
àö84□ò&ƒamã¯ŒqÇmj±Ø^êOpbQ€

\•l¨ÇÔ×ØÕÂø3á7‡<□ªI©i¯{=ÓÆb□w"¶Å$□€ª¾ƒšî¨ ¨n-`¾´šÒê□šÞd1É□Œ«©□
ŠšŠ ò™?g¿□IzgWÕ#□v~Î·+³é’¥±ÿ □®÷Xð®‹¯h
¢j6)5,*¬q’A□hÂ•n •Þ¶h □0Òþ□ø+LÔ’õ£½½□Û–
¹•¢□Ü□□ý "°»? ø_Oñ3xŽ×JHµffc8•ú°!ˆ]ÛFA=□t”P□7®x□Ã-$ÕàÕum)no UXå2È¸
I …`□$õ□Ïx□àŸƒüI©¾¡$7V7□¶é~Å"¢ÈÝÉR¤□ôÅz-□□ÏøSÁz,ì-×E´ò¼Â

²»n’R:noè8ç¥-&ðO‡|aöoíí4]›mÞQó^2»±žQ†z

µÐQ@□tÝ6ÏGÓmôë□□□–è#Š0I
£¶O5Äx‟àÇƒ¼K}%ìÖ‟Ù]JwI%”□7ŸR¤□Ï¸□× Ñ@-Ueû>x&ÖPó□Nì□÷'¹ -
ûáTþµêh‹□k□(TP□•Ø
u□ Wœë¿□<□®ÝÉtm'°šBYÍ”»□‰ï´,£ð□½□Š óm□àg,tk¤¹k[•BD9Q}(u□ÝT(?ˆ5é
¡T*€□

:
Z(□–Ò¾□øCDÖÿ ¶tí□□{ðX¬□Ü„'ƒµI*¼□Ð
êh¢€
â5□„>□Õ5□‹ûÍ□=ÍÌ•,®.¦@ÌÇ$á\□"è+·¢€<ÿ þ□—ÃÏú□¿òvãÿ ŽWk

—co¤Å¥%´fÂ(□°Ààzùav…9ÎF□9«tP□□ÿ
‡À?lûWü#vþfs·Ì‟gýñ»oáŠÛ‡ÁÞ¶ñ

Zì□dQj0Áöxå□™U#Æ6„□hãŽ•¹E Esm□å¬¶×PÇ4□©I#‘C+©ê□=EršoÂß□i□´Z¥Žƒ

W‛>øÜË#□aÐ…,T□ô®ÂŠ ÃÕü-
ëÚ½†«©éâ{Û□□m)‛×a□¸p□□‛žA-□WK²Ö´»□7Q€Ogp›%Œ±]Ãê□#ð«”P□~‰¡é¾□ÒaÒô›ame

ï. ìØÉ,ybIäž¦©ø‹Áú□‹ >Éý¹§‹¿²1x3#¦ÂqŸ°Fz

¾•¹E xçí□□©ZøKN·µiRÂêä¥ãÇß□*§ØòqþÈ«ú0ø.tKd…¼0Ñ¤`n½□ ÛÝ÷á‰¯M¼²µÔ-
$´½¶ŠæÚQ‡Šd

¬=Áà×□7Á¿‡óÌe□

D□œá.%Eüƒ•@–FðèWß□´□ðê–°C$o{%°a□□0Œô]œ□pIÀëÏÒÕ•¡øgDðÔ□

- ¦[Y#}ó□aŸýæê□□Z´□□Ä□][Ko2□ŠT(ê{©□#ò¬¿

x[Eð•„–:□□´¶’S+ ‘ß.@□Ë□z□[□P□'‰<#¡x°Þ□5Û□w□

^ dtÚHĂû¤f¶•B¨P0 À□´P□&†+ˆ^□âIb‘J°:†V□¨ õ□Å\|ð□ÕĂžO

@□œâ9d•□i•`?Jî(

í@Ò<?jm´□:ÚÊ□Ë,□…Ü}IêO¹©uM'OÖì-ÇS³†îÕþôS
ažÇØûÕÊ(□'Ò~□ø+D¿[ë□□Û¥Ê□Èò3Ë´ú€ì@>â¼ÛáWü—
o□ÿ ×[¯ý)□îõ‡¥ø?AÑuËýkOÓÖ□Fü±¹˜Híæ□mÇ,H□<ð□ hêZeŽ±a-
Ž£i□Õ¬£□□È□Oÿ _Þ¹½/áo,tkõ½²ðý²Ü)Ü-#<¡O¨

Ä□ô□×Ñ@□Ú□….4M/_¾×,¬DZ•ðÅÌþc□üƒÐœ

@è□hßXZjvrYßZÃsm ÃÅ2□V-àÕŠ(□†?□< n<ÿ øG!ßœàO.ßûçv?Jë´í.ÃG³[=6Ê
Keé□□„\úàw÷«tP□□¾□ÐgñT^'"O□¬Ä»□çÍ~□Ò¿w;z□:Vå□P□‡|-
øOí□Øzx´û[□›□;ï#8ûÄã©éëVµ¯□é>"²û-±§Á{ 9
*çiõ□¨>â´¨

GHø_à-

õo,<?l—□w#ÊÏ.Óê7“ƒî+¢Õt»-kK¸Óu□□öw ²XË□Ü> ,?
¹E gèš-›áÍ&□/I¶□ÖPîòâ

ÍŒ’O,I<"ÔÕ-□Á¾□ñNÓ-i□×n£
#)W□Ð:á±íšÝ¢€8ý/ág,4k•¸³ðí¯š§*Ó□›iõ□ÉÁ®®æÖÞöÖKk¨"žÞEÚñJ□•‡¡□,*Z(□‡oƒÞ {
¯´

A¿9ÀšPŸ÷Èm¿¥v□V6šmœVv6Ñ[[D6¤P UQì□X¢€
Žx!¹□à¸‰%†E*ñÈ¡•□ê□=EIE pó|-ð□ÅÉ•ü9 rs^å•□þùV□ô®¯JÑôÝ

Élô»□,í”äG□□□õ8ê}êí□□—¯øwJñF-tÝfÐ]Z□

c.Éó

‡*Aýjå…□¶™§ÛXYÄ"µ¶□bŠ0IÚŠ0□O=□X¢€
ÎÖt□'ÄV□dÖ4û{Ø□Ê¬È

ÓêP}ÅhÑ@fŸð›À°eÚÝ[xvÛÍS•ó•å ÿ °ìGé]ŸJ(

o^ø□áO□Ü□□_D¶¸¸<□†cvú²□Oâj=□áÏ„<9p·□^ƒk□èr’¾é]O¨g$□Â°Š(□ž«¥ÙkZ]Æ›¨À'³¸M
’ÆX®áõ□□øTz&‡¦øsI‡KÒm…µ”;¼¸ƒ³c$�much□O$õ5¡E akþ□ðçŠv•kH¶»u□Y□J¸-•×□•lÖf—
ð³Á□5ÊÜYøv×ÍS•i‹M´ú•äà×aE □Ëk¿

<!âKÃwªèVó\·Þ•□¢gÿ x¡□ük©¢€24
è~□·h4]2ÞÉ-
ï˜×æ□ǂ˜ò□□W5=.ÃYÓå°Ô-"°´”aâ•r□ÿ _Þ-Ñ@□¶ƒðãÂ>□Ô†£¤h±[Ý€BÊe’B Œ□ncŽ8â¶õm□
L×l□ž«coynN|¹□0□ÔzqW¨

-ƒÞ ,àNž□€¸9ÃÍ+¯ýòX•Ò»+[Kk□hí--â··ŒmH¢@Š£Ð□À©¨

O□øCÃþ*•□[Ò ¼òøG`UÔz□R□□lÕ}□À>□ðì3Ç¥h¶öþ|f9æwd#□w±-
•l×GE ex□ÃzG…tßìí□ÌZÚ□

žX‛Ÿæ8ÉË□{
Õ¢Š Ë×ü;¥x£K:n³h.-
‡1—dù‡C•
þµrÂÆÛLÓí¬,â□ZÛF±E□$íE□□'ž,¬Q@□5}□L×¬□ž-aoynNvL□€>£ÐûŠåí¾□x□ÖäO‡-ÙÁÎ%-
I□þùf#ô®ÞŠ dQG□I□1¤q
¨ƒ□@è □*ž¯¢išõ‛³Õ¬-ï-ÉÎÉ•0□ÔzqWè

"Ûá□€mnDñørÝœ□âYd'□i-b?Jí"Š8"H¡•#• UD□
□@ éO¢€)êšNŸ-Ø=Ž§g□Ý«ýè¦@Ã=•±÷®sJø]à-□ýo¬|?l—□w#ÈÏ.Óê□± ûŠëè

k□
hšf¿}®ÙØˆµ+á‹™ÄŽwŒƒÐœ

ƒ □³E□□á?–¯¤–‰ð¾›ªI<^□’A‰Ù‹ ?9

□ôêU9ï□ê'¾

=Š°ÿ Â#å□ n□‰1ŽàüÄýy®ÿ TÒ4ínÉ¬õK(/-˜äÇ<aÆ}yè}ë'?□~-
□|ßøGcÝœãí3còßŠ òÿ C§Ý|}žëÁ1I-
‡a‰þÐT0Œ©□□□ç□L□□¦G□½¿Wð~ƒ¯jö□®§§‰ïl□5´¦G]„6áÀ -Fy□¯i:.™¡Y‹M*ÂÞÊÜ□ì,0 Ÿ
SŽ§ÜÕê *µþŸgªYÉg□k□Õ´ƒ□□È□[ð5fŠ á‡Áß
•<xr□ùÎ

Ò•ÿ ¾wcô®ÊÊÊ×N´ŽÒÊÚ□khÆ□(P"¨ö□ŠžŠ ©©i-:Å„¶:□¤7V²Œ<S e?ý□žæô¿…¾
Ñ¯ÖöËÃöËp§r´Œò…> ;□□ÐW_E ciþ□Ñ4½~û\²±□jWÃ□3ùŽwò□Bp9□ □ä_□□ä¯ø
□þ»Cÿ ¥□½Ú°õ□□è:ö¯aªêzxžöÁƒ[JduØCn□□□äg□h□3Tø[à□cPkûß□Û½Ë□ÎÑ»Æ□ú□Œ□?Q]&
›¥Øhö1Ùi¶□ÚZÇ÷b…□¨ü□□ž·E sÞ ð7†<S —YÑ-®¦□-w)&=7©□-Ù¦h- ð¯…î>Ñ£è¶ö÷□À˜-
'Àï†rHü□t"P□E□P□5¯|>ðŸ‰®MÎ¯¢[\\½2î□ÛêÈA?□;@ð□…|1?Ÿ£è-
ÖÓã□c™$□Ù˜'?□]□□ V□□ƒô□7Ä—~!´ÓÄZ-â•žãÌs¼□
ùIÚ9QÐv-Ê(¢Š(□□ñ□€ü1âÉ'moH†êd]«.æ□ñé¹□${□ÐÐ¼?¥xkM□~□e□¥¨bþZ□
rÇ©$'Iàr}+JŠ ÃÐ| øbêúçGÓÅ¬×Ì-á„ŽÛÈ$□¼N>ñéŽµ{XÑì5ý&ãKÕ-ÅÅ•À□X‹2î ,9R□ä

†¯Q@□t}-Ã@Òmô½2Ü[Ù[,"ˆ1m ’O$’y'©ª□ÿ ƒ|9â•§ZÒ-
®ÝF□FR®□ uÃcÛ5»E qú_ÂÏ□h×+qgáÛ_5NU¦-
6Óê7“ƒ]…□P□\¶¹ðãÂ-$»k-WB¶šá¹yP´NçÕŠ□Oã]M□□‡áÿ □øwÂ¡ÿ ±4›{6q†‘Agaè]‰l{f—
Äž□Ð¼]o□□í€»Ž□/□2:m$`ýÒ3[tP□□ˆ¼□ x²;Xõ»□µÇhKB¦i□)8Ï
Ã=□\ÖÜq¤1$Q"¤h¡UT`(□ □ê(□žµð?‡,¼Q/‰-tÅ‡V”±’t•ÀbÃ□”Ý·Ÿ§^z×CE□ Œ¡”« A□
÷®0ü%ð!ÔEÿ ü#-ÂpûðÂgýÍÛ□

WiE G<□ÜÀð\D’Ã"•xäPÊÀõ□-¢¸¹¾□x□âäÎþ□€99ÄrÊ‹ÿ |«□úWqE RÒ´}7C²[=.Æ
;e9□À□F}N:Ÿz»E□ W ß□>-»□>-□''□“□ùo®þŠ óÿ øR_□?è^ÿ ÉÛ□þ9]Õ-¬6VpÚ[F#,□Ö8Ð
□
¨À•©¨

GWøaà-réî¯ü?jÓ¹Ë¼E¡,}NÂ2}êÎ…ðûÂ~□¸□□N‡k□ÂýÙ›2:ý□É#ð®–
Š (¢Š Äñ'„4/□[Á□»`.ã•ËÆ

®›I□?tŠÚU
¡@À□ RÑ@□vž□Ðl|Msâ;m<&-r¥f¸ó\î□□ùIÚ>èè+KQÓ-5m6ãO¾„Mis□ŽXÉ#rž£#‘øUª(□7Að
þ—á•)4Í-Ô[Y£□XÃ³òNIË□□Ž□]ðÆ‰âkuƒZÓ-
ïQ>á•~dÿ u‡#ð5-E q°wÂ□□éWKskáÛo5NTÌï0□×

ÄWd □□□E□□‡/ƒô□üU□‰äÓÃk1.Ä¹ó_□´¯ÝÎÞ„Ž•¹E□□‡áß□è>□û_ö-ž-
>ÖÁæÄŽûÈÎ>ñ8êzzÕ□sÃÚG‰l>Ã¬ØCyo»pI□Ý> ŽA÷□´è

wÃ~□ðÏ„f–m□JŽÒY—cÉæ;±\ç□bH□□□jšÇÁú

•âK¿□Úiâ=VíJÏqæ9Þ   □ü¤ítt□«rŠ †îÒ
û)ìî£□[ÜFÑJ„‛¹X`Ž=□gø{ÃZG…4Ó§h-
bÖÔÈe1‰□òÄ NX“ØwíZÔP□E□P□5¯|>ðŸ‰®MÎ¯¢[\\½2î□ÛêÈA?□;@ð□…|1?Ÿ£è-
ÖÓã□c™$□Ù˜’?□]□□□□ðGso-¼È-)P£©î¤`ŠËðç…´_ XIc¡Ù
Ky%2°   □òä□œ±' □±E fkž□ÑüKd-5•>
Ø□Ê¬«ÊŸU#□~†²ô‡^□ðÍßÚô•

Þ
'÷fbÒ:ÿ °\'?
éè

m
Âš'†^õô{□j×²y·□Hí½¹çæ'□O□□šÙ¢Š GE'□□C+

□# •Jâî>□ø

êðÝIá»a!9"7‘□?î+□ý+µ¢€+XiÖZU”vz}¬6¶ÑŒ$P U-
€¬Í□Áú□†.¯®t}<ZÍ|Áî□Hí¼,HûÄãï-˜ë[”P□□cG°×ô›•/T·□□W
b,Ë¸□□åH#•:□4}-Ã@Òmô½2Ü[Ù[,"ˆ1m ’O$’y'©«ÔP□□□ƒô□;Ä—
~!´ÓÄz-Ú•žãÌs¼□ ùIÚ>èè;Q«ø?A×µ{□WSÓÄ÷¶

□ÚS#®Â□pà□□#<ƒ[”P□\v¡ð«Àú¥û^ÝxvØÎÍ¹Œnñ†>¥U€?•v4P□;¨x□Âúž□c§ÜèÐ}’ÆO2Ú□‹D
±·®□Œþ=jö¿áÝ+Å□[i°Í °´g

c.Éó

‡*AýkRŠ ¯acm¥éöÖ□q□-m£X¢Œ□vªŒ□"ÏAÞ¬□□ ŒƒÔ□(

3PøMàMNå®.|9l$c�"ä»Â ú#□[z□…4

ÆÉ£i6¶{†□ãO□‡¡cÉüMlQ@□ž$ð†…âëx ×l□Üp9xÁ•Ói#□î‘[J¡T(□ `
Z(□□þ□ý□þ□ÏøJ?³ÇöÖÝŸió¦ÝŸw;~ï□+rŠ(□9àŽæÞ[y•<R¡GSÝHÁ□—áÏ
h¾□°’ÇC²□-òHet□;åÈ□9bO@+bŠ Ã—Áú

þ*‹Äòiáµ˜—b\ù¯ÀÚWîçoBGJÜ¢Š Ãðïƒô ý¯û□O□Ÿk`óbG}äg-
xœu==krŠ(¢Š(¢Š(¢Š(¢Š(¢Š(¢Š(¢Š(¢Š(¢Š(¢Š(¢Š(¢Š(¢Š(¢Š(¢Š(¢
Š(¢Š(¢Š(¢Š(¢Š(¢Š(¢Š(¢Š(¢Š(¢Š(¢Š(¢Š(¢Š(¢Š(¢Š(¢Š(¢Š(¢Š(
¢Š(¢Š(¢Š(¢Š(¢Š(¢Š(¢Š(¢Š(¢Š(¢Š(¢«_ê6:U©°ÔomìíÁ Ëq*Æ€žƒ,@©`ž□˜#
žÞT-□□:I□□VSÈ Ž□ (¢Š (¢Š (¢Š (¢Š (¢Š (¢¯¼½µÓí$»½¹†ÚÚ1—
ši□"óŽXð94□=□□-Ýµõ¬wVw□\[È2’ÂáÑ‡¨#ƒSP□E□P□E□P□E□P□E□P□E□P□ERÔu□3GŽ95=FÒ
É$m¨×3¬a• ,FM]ÎFE □QE □QE □U&Ö4ÄÕ□K}JÍu□]ËhgQ+

g!3’0□nÕv€
(¢€
(¢€
(¢€
(¢€
(¢€
(¢€
(¢€
(¢€
(¢€
(¢€
(¢€
(¢€
(¢€
(¢€
(¢€
(¢€
(¢€
(¢€
(¢€
(¢€
(¢€
(¢€
(¢€
(¢€
(¢€
(¢€
(¢€
(¢€
(¢€
(¢€
(¢€
(¢€
(¢€
(¢€
(¢€
(¢€
(¢€
(¢€
(¢€
(¢€
(¢€
(¢€
(¢€
(¢€
(¢€
(¢€
(¢€
(¢€
(¢€
(¢€
(¢€
(¢€
(¢€
(¢€
(¢€
(¢€
(¢€

(¢∈
(¢∈
(¢∈
(¢∈
(¢∈
(¢∈
(¢∈
(¢∈
(¢∈
(¢∈
(¢∈
(¢∈
(¢∈
(¢∈
(¢∈
(¢∈
(¢∈
(¢∈
(¢∈
(¢∈
(¢∈
(¢∈
(¢∈
(¢∈
(¢∈
(¢∈
(¢∈
(¢∈
(¢∈
(¢∈
(¢∈
(¢∈
(¢∈
(¢∈
(¢∈
(¢∈
(¢∈
(¢∈
(¢∈
(¢∈
(¢∈
(¢∈
(¢∈
(¢∈
(¢∈
(¢∈
(¢∈
(¢∈
(¢∈
(¢∈
(¢∈
(¢∈
(¢∈
(¢∈
(¢∈
(¢∈
(¢∈
(¢∈

(¢€
(¢€
(¢€
(¢€
(¢€
(¢€
(¢€
(¢€
(¢€
(¢€
(¢€
(¢€
(¢€
(¢€
(¢€
(¢€
(¢€
(¢€
(¢€
(¢€
(¢€
dÌR □z…$~TúŽãþ=¥ÿ pÿ * ù/Â_□ü[¡ë□šî¥ªê:†•÷&
¨§□¤B□□à□…`

F1Ó□3_YÛÜCwm□Í¼‹,□ ’7S•ÊFA-ÄWÍÿ ¼)□•>□ø§G—jÊ×1Ém!ÿ –
r„;OÓ±ö&»□□>,žK+¿□êÛ£Ôt¦o%$ûÞXl2}Q¿B=(□□·µ□øi¿ì□í;¿ìÏ#þ<üæò¿ãÛvvg□ÝÎkÙ+
Â¿æìÿ í‡þÚW¢øö□]ÅaeàÙ-m|æ□¶[^NFaP□Ü□□\·@O□¥ v□W‹j□~#YXK¨YüH¿°¿‰

,ÕŒ‹□□3´□ä}2¸úWKðwÇ7¾7ð¬Ïª□mBÊo&Y□B‰TŒ«□8□¨8ã•z w•|-
âÝwÆ:>§¢xŒéúm¨AqmçH›ˆrÌÁTa²¤

□t÷¯B¯□ø§¯júoÅ/□ÙØêw-
Ö·□D&†□™□Pf î á¸ãšßøßªj□GÃ©.´Ûë‹;□µD¾m¼¦7ÁÎFG4□èôW□èv¿□~'i0jPø†m□L†%†Ûc
¸’é” Ò1R Ë□É8ô□Mwþ)ñmÇÃo†ö—
:¤«¨ë"(í□’q<ûycßhÁ'×Û4□ÐÑ^□¤xSâŸ□tèµ½CÆsèét¢X- gB□ò¹T* #□I8ëQé¾2ñ—
Ão□Ùø{Æ·£RÒïJˆ¯
ÜT□´8r□8?y[$u-à□Gí□ÿ $ÂOúü‡ú×]ð÷þIÇ†ÿ ì□oÿ ¢År?´□ü‟ ?ëò-
ë\Ÿ…tÏ‰>7ð¦™□ž¬<5¡ÚZÇ□»G¸MsµBïÈÃ`ãÔ□@zÐ□¿W%ñ□ÃÚ÷‰¼0¶-□Õ¿³/EÂÈÒ™^0è□□¥•□
9 þ□åÚ_Š¼cðÏâ□—‡|]©¶«¥_2ˆîdbä□;C†?7□ï)Ï□;□ÛüoÕ5□#áÔ—
ZmíÅ□ÇŰ¢_6ÞS□àç##š ì¼5a□¥økN±ÔïMíô□*MpI>c

§'“õ<šÕ¬□□ÝO{à=□êêgšâm>□’Y□,ìP□IîMoÐ□Ey¿‹4o‰-!ñ4öšN³□‡áøÕ6\Gƒ4Ä¨,xù†

GU□w®#Åš/ÄO†zjøŠ×Ç7zÅ¬2*Ï□Ðb□1Àù]˜□I□,□#-À-ÿ Edx[_□ø[MÖV?/í□,Œ™ÎÖþ!ø□Š
ùûÂž.ø‡¬ë:ß‡t□¹®nn.‹□ëÉZE²‰Y□Û»!s‘Øôàg □Ó□WÏ>*Ó>&|5´‹ÄcÆ3ê¶É"-Är»²!'□(Ä
•¤ñ‘ƒÈ¯nðÎ¿□ˆ¼)§ëŠ¢$º·□°“Ââ□ô□□Ÿj Ø®□ãgü’-
wé□þ□Ž¼ò□□xãâÿ ‰ïàðÖ¯&‰ Y6□Ñ±F ’□’¿33`œd ?Z-
□¬>"xKÁ·ú^³©□□A½òÔÞ6ZKw□+□ÄüÀ□¸ä‘ÏPx -³ð{þIF•ÿ \Ÿÿ F=w□Ãü-ÿ ’Q □k'ÿ Ñ•]
Å xŸÇ½o[Ó¯ü3e¤j÷šp»iƒ›iÚ-Ç1□’¤□□ãÇ½hü□ñ~©ªE«øoÄ7RÏ«is□4íºB™ÚÀž§k

§ûÂ°¿h/ù□ü□ÿ]eÿ Ðâ¨üh□á]|vÒüNŸ»Ó5q¶èŽ□œ$¿—Èÿ Z ÷ÊñïŒž*Ö□VÑ¼-
á»Ù-u□Öó®%•Ê:GÈQ¸r□

Ç□áG-zìÓÅoo%ÄÒ*C□□w'…P2Oå^□ðÞ)<kã?□xþí□’‰%µˆa÷r¸ãÝc
□ûæ€6ÿ gýwVÖü9«□WR°¾hn”F÷2´Œ ¯#s□qí^½^!û5ÿ È¹-ÿ ×Ú□è□Óø³Fø‘â□Oi¤ë0h~-
•SeÄx3LJ,Ç□˜`äuQÇz ôŠ+À<Y¢üDøg¦¯ˆ-|sw¬ZÃ"¬ñ]□!C□□•Ù•□•8
òlíìÞ□×âñ□„´ýt¨….m„Ò)<Fqó

ú□□ lÑ^□oâ/□ü^ñ□ü>□Õ□CĐ,Ûož„£°9ÚI1cŒà□ ýz-□Ã□<+âM8Ÿ□□oèÒÎ©x·

KÇ□<·ÎIà□ı□Ò€2□i_ù□h?õó'þ,+Û-ÿ ãÚ/÷□ò¯□ý¥□ä□ ÿ ×ÌŸú□©môOŠ-?²]ZM|øbÉÔ□;(
□ÕÊv.W□ž:ŸÀ
öÚ+Å¾□øÏÄ¶Þ7¿ð'‹n□ÕÝ°±†v9|®

7ue*w□yüøî|{□•®â°²ðlÖ¶¾s?Û/'#0¨□n□□®['□Ò€;
+Åµ□†¿□¬¬%Ô,þ$_Ý_Ä†AjÆE•È□Ú

ò>™\}+¥ø;ã›ß□øVgÕ
¶¡e7‟,Š¡DªFU^□□Ô□qÇ½ q°·ü□n•ÿ \Gþ"½{µxN-ÿ '[¥□k□ÿ ¤ïW|gã□oÆ^%_□ü?□Ñ‛¿Óµ
H\¨@□ 8è£¹□"À÷ öš+□Í#ðgƒ7jz¥Õòiöí-ÅåÓ—’Le˜ò□ 3è+Ç´□Câ?Åë›«ý3X>–Ð¢•Ç-
’ÅXŸ@W

Ç□dä

x □□¢¾|×[âgÂSo«Oâ□×ôv•G2Ü3>   ìÛ²ËžÌ□^¾‡ÕïüomÃ9|ee□›□ØþÑ□MýãÀVÇ£□□z□ ë(¯□ð¿‡¼□ħ□D•ÄÓüA°Ó□åßÉ·´

□□b¼„u□□}N:×ª ø#Iñ□‹¢ÍgâMa5k‘pÆ
€

|-«€Ù ç;•~½h□¥¢Š(¢Š(¢Š(¢Š(¢Š(¢Š(¢Š(¢Š(¢Š(¢Š(¢Š(¢Š(¢Š(¢Š(¢
Š(¢Š(¢Š(¢Š(¢Š(¢Š(¢Š(¢Š(¢Š(¢Š(¢Š(¢Š(¢Š(¢Š(¢Š(¢Š(¢Š(¢Š(
¢Š(¢Š(¢Š(¢Š(¢Š(¢Š(¢Š(¢Š(¢Š(¢Š(¢Š(¢Š(¢Š(¢Š(¢Š(¢Š(¢Š(¢Š(
 ¢Š(¢Š(¢Š(¢Š(¢Š(¢Š(¢Š(¢Š(¢Š(¢Š(¢Š(¢Š(¢Š(¢Š(¢Š(¢Š(¢Š(¢Š
(¢Š(¢Š(¢Š(¢Š(¢Š(¢Š(¢Š(¢Š(¢Š(¢Š(¢Š(¢Š(¢Š(¢Š(¢Š(¢Š(¢Š(¢
Š(¢Š(¢Š(¢Š(¢Š(¢Š(¢Š(¢Š(¢Š(¢Š(¢Š(¢Š(¢Š(¢Š(¢Š(¢Š(¢Š(¢Š(
¢Š(¢Š(¢Š(¢Š(¢Š(¢Š(¢Š(¢Š(¢Š(¢Š(¢Š(¢Š(¢Š(¢Š(¢Š(¢Š(¢Š(¢Š(
 ¢Š(¢Š(¢Š(¢Š(¢Š(¢Š(¢Š(¢Š(¢Š(¢Š(¢Š(¢Š(¨î?ãÚ_÷□ò©)")xdAÕ"•ùP□
^þÍ_ò ×□ëê?ý□Õo‹ÚEß,¼k¦|EÑcÀ3*^"ð
ã□û:eO¸õ5Óü□ðn»àí-
Uƒ]³□²Ü\+F¢T"r…Æ~Rq^ƒâ□□ÓÄš□î□|»-î¢1·ªžÌ=ÁÁ-â€<?GÕmuÏÚnÏU²}ö×vk,g¾□$Cî:
q]ßÄß‰7^□°Óô]□Á/uÍG-JI'¨
m^□□‰l€2:W
ðÏáGŠü)ñ6ßPÔmbþÍ´Y"]$èD™FUÚ¹Ü3œò□v?□>□j¾&Ô´¿□xrê8µ•7□□S´8VÞ¥O
@Á‰ëÁÏ·
□ë¤|s¼A1ñ-‰bXdÀcBWÛ"□þf²ÿ fŸù□øƒþ»Ãÿ µiy?□¼I□Ó/×LÐmœmšö□S)^ûv»`ý6ýEhü□
ðN³à¨5ë}ZÛÉIîPÛ7šŽdE

7|¤ã¨àâ€9¿Œ?òWü ÿ]¡ÿ Ò•]'í□ÿ $ÂOúü‡úÔ_□ü□¯xƒâ7„õm2ÈMea,ms!™□Ë
(bpH'•@koâÿ †õ_□x□M7F¶□7□iŽA□'S*3žX□ßÖ€5þ-ÿ É8ðßýƒ`ÿ Ð□ygí,%û□†ÛŸ
K8|□{□ãô□^½àý>çIð^‰§^ ŽêÖÆ□e@Á¶² □dpyôªž;ðeŸŽ¼3."tæ□

% ·œ

˜¤□ÁÇq,A-†€7íd†[8$¶*`xÕ£+Ð©□cð¯
ý¤Ìrÿ Â3m□ÝxÏ>Å_½få□Ôãò©´½'ã_ƒì×GÓcÓõK□FÈ$’XÎÅì□öVÇ±Î:
Óð¿Âÿ □ê~/‡Åß□/ã¹¼·*Ööq□U
ò¹À
□žB®ry'®@.ü{

¿
° Ç . .

}O5Ø|<!¾□xl,□þÍ€qþà¬□‹þ□Õ|UàGÓtkasvnc□FdTÊŒç- wõ®&ÃÁ¿□ü□i□^□»·Ôlf•^K
§LÛÊ@Þ□b□3žU†{Žô□Oö□Û>¥á[ks›ÒgÀ^¸c□_Ô□~5Ö~Ð-
òL$ÿ ¯È□+dx[áŸŠ5¯□Ãâÿ ˆ□14öÌ-□œl□

¼®vüªªyÀ''¯|õ¿□ü7ªø«À’i°5°¹»ûLr□ÌŠ™QœòÄ

þ´□±ð÷þIÇ†ÿ ì□oÿ ¢Åt•‹àý>çIð^‰§^
ŽêÖÆ□e@Á¶² □dpyô-ª ñÿ □|IñF¯ã›Ÿ□ø□ÂÝîm7
›ËžB•Àb□8 □□NI=□¯/ñ□Hø-oàMFãÄ¾#Ò®t•åyöÖñ¨vÌ¨□□B½□iûÝ»Öö½ðÿ Æ~□ø…{âï□µ-×
Û‹4ö"²©Ë□Î§q ©`□□A□>µ¼Eá_Šþ<Ðî"]{□□#Mñivn£í2□°□·□Žü¶2□□À□}ð‹þIW‡ÿ ëƒ□èm
^}ð□þFo□ÿ ×hÿ ô9kÓþ□è÷Ú□€4}/R„Cyo Yc

□i,N2
□¯jãþ□ø/^ð®»â‹□bÈ[Ã{*□v□#ù€4„œ)8ûÃ®:Ð□ßÆp□ÂM{#ø!ÿ ÑÉY~□□¿ìñ□‡
>iÓo□c®íÒâ°O‰z-ÿ ˆ¾-júV—□žöá#□F]Wq□+-X€8□©£á®‰}áÿ ‡zN"ªÀ!¼•$□Å½\

ÈÌ□A ðE pŸ³t□Ÿ□j±®<õÔ
?®Ó□mýCWOñµ•~□k€•   ò Éê|øë^—
á¿Ž¼□â‹ÍKáô•\é÷g›I]WhÉ!X9 •ž□□ãñÉ®ü<ø‹ã•"î÷Å7Ñ
^"&ÃH´uT2ú±ÎÞ™êI÷□¨□□ð{þIF•ÿ \Ÿÿ F=w□Ë|8Ñ¯¼?ðûHÒõ8D7–ñ°-
0á¶’ìq□H<□Ò°š ð□Ú
þF?□ÿ ×Y□ð8«´øÕáŸøH¾□ÝÉ□n»ÓOÛ"Àä…8ÿ ¾I?P+/âÿ ,µï□ë^□¸Ñ¬–â+)\Ü1™□`-
□□æ#?tôÍz³¢ÈŒŽ¡‘•

¤d□é@;jß□šóà□□Œs□Ö.Ûû.P

[b□¹½÷!@}Ü×®x?Ã+á/†¶úIP'KV′àŽò°%¾¸'@+Ê|1ðKUÓ~*-
Õåª□Â;gr×□ò™'Œ □Æ»s»9ÛœŒ|¦½þò6šÊx"-
xÙGÔŠ ñoÙ¯þEÍoþ¾Óÿ @«þ"ø"â□_Ç7>□ð%…»ÜÚn□7—<…+€Ä□p $

œ’z□[ß□|□®x?DÕ-õÛ1k,÷*ñ¨•$Ê…Ær¤ŠÇ×¾øÏÃ□□¯|]àVµ°ûqfžÒvU9b□Ôî
□,□□□#§Ô□□â.‘ñZßÀš□Ç‰|G¥\é□Êóí-ãPí™P.□…z6Ó÷»w®çÂK+~Î□`Ïœt{ ˜ë»□cõ®oÄ^□ø
¯ãÍ

é5×±°,4ß□—fê>Ó û¡›q□iËc qÜz_ÃÍ□óCø}¤i:¤□+¨!)4[ƒ€K1ÆA
ðh□„ýœ$„ø□R•Hó×Rbã¾

qíü8oÖ½ˆ□:œW…Mð×Ç>□ñ-Þ¥ðöâ
‹
£–²™Ô□3□¬□€@ÉÃ□□ë□□'Â¿□¼Qâ=3Tñ-ý½…†›p—
1éö¬□™"□®v’1‘Ô±ï€3š Ëý¥□à□ ÿ ×ÌŸú□¯n·ÿ •h¿Ü-
Ê¼¿ãwƒuß□é:L:□□°’ÞáÚE2¤{AP□ù^ÍzŒ*R□ÔõU þT□á`cöµlw‡Ÿ□ôJì>&üI°ð…ÖŸ¢è¶
{®j8òRL•@[jð□,Kd□‘Ò³ÿ á
×¿á¡□Š¾Ä?±|¬}£ÎN¿gòñ·;¾÷µMñSáÖ«âmKKñ□‡.£‹YÓp□%;C…mêTô

□ž¼□ûr□ž°GÇ;Ä□□è-%†L□4%}²!oæk/öiÿ •O^?ë¼?ú
V—"ñ«Ä□□2ýtÍ□ÙÆÙ¯`e2•ï·k¶□ÓoÔV□ÁŸ□ë>
ƒ^·Õ-¼"žå□³y¨æDPÃwÊN:Ž

(□‡ñÎ•.¹ûDÅ¥Á{-”·VF5¸ˆüÈM»þ••±=*ÿ Á•m<□â□CÀZõœ6Z“Ï°+Œ`ÌØá
w□r‡¾HêEtš‡,õéÿ h□?Å□Ù□£Å□□qç Áò□q·;°‘Ú¯üXøn|g§E¨é Eâ
,□□6Ï5AÎÂÝˆ<©ì~¹ □þ1,-ðŸ_□ç□□8þïš›¿LÕ?•²@ÿ
´¥„®ôyÖ\u□æ±çð+úV×… Öõ•□ÿ gxÛMX¯^7µ¹O5□O□□ß”$□A9ç¨'Ž+Ìm<□ñ+áÎ
¡t¾
»¶Ô´¹ßx·□‘O±er l`eO8□ô □çÆ‰`□á>¶'#çX•□îþjc-□
§ð“J□Pø)§iÚœ>mµÔw
Ñ7xÚWÿ □Šå.|□ñâ-õ²øÞößMÒ`}æÖÙ”′}@RFq‘-
cŒð+Ô<CáëÙ|□ú'…ï□naŽ$³•X□□FR□‘Î□\-½yÍ y¤¿

üoà)'»ð□¾×□EŒ‡M°Æ[Ø□ò1÷ùMv_
¾"7□t»±wf¶š□ƒª\Æ™Øwg

□är¬0sŒuæ¹syñÙ 6Ùš<••¿Ú□ãÝþö7•ÿ Ž~□Óü,ø{/□4«Ç¿°K□WP‘d¹xòUvç
äòÌIã¯µ wÔW–Úh¿□ÓãDšŒ÷³Ÿ
—r□Ýƒ□C□
¢,ä0lsŽÙÍz• □QE □QE □QE □QE □QE □QE □QE □QE □QE □QE □QE □QE □QE □QE □QE
□QE □QE □QE □QE □QE □QE □QE □QE □QE □QE □QE □QE □QE □QE □QE □QE □QE □Q
E □QE □QE □QE □QE □QE □QE □QE □QE □QE □QE □QE □QE □QE □QE □QE □QE □QE □
QE □QE □QE □QE □QE □QE □QE □QE □QE □QE □QE □QE □QE □QE □QE □QE □QE □QE
□QE □QE □QE □QE □QE □QE □QE □QE □QE □QE □QE □QE □QE □QE □QE □QE □QE □QE
□QE □QE □QE □QE □QE □QE □QE □QE □QE □QE □QE □QE □QE □QE □QE □QE □QE □Q
E □QE □QE □QE □QE □QE □QE □QE □QE □QE □QE □QE □QE □QE □QE □QE □QE □QE □
QE □QE □QE □QE □QE □QE □QE □QE □QE □QE □QE □QE □QE □QE □QE □QE □QE □QE
□QE □QE □QE □QE □QE □QE □QE □QE □QE □QE □QE □QE □QE □QE □QE □QE □QE □QE
□QE □QE □QE □QE □QE □QE □QE □QE □QE □QE □QE □QE □QE □QE □QE □QE □QE □Q
E □QE □QE □QE □QE □QE □QE □QE □QE □QE □QE □QE □QE □QE □QE □QE □QE □QE □
QE □QE □QE □QE □QE □QE □QE □QE □QE □QE □QE □QE □QE □QE □QE □QE □QE □QE
□QE □QE □QE □QE □QE □QE □QE □QE □QE □QE □QE □QE □QE □QE □QE □QE □QE □QE
□QE □QE □QE □QE □QE □QE □QE □QE □QE □QE □QE □QE □QE □QE □QE □QE □QE □Q
E □QE □QE □QE □QE □QE □QE □QE □QE □QE □QE □QE □QE □QE □QE □QE □QE □QE □
QE □QE □QE □QE □QE □QE □QE □QE □QE □QE □QE □QE □QE □QE □QE □QE □QE □QE
□QE □QE □QE □QE □QE □QE □QE □QE □QE □QE □QE □QE □QE □QE □QE □QE □QE □QE
□QE □QE □QE □QE □QE □QE □QE □QE □QE □QE □QE □QE □QE □QE □QE □QE □QE □Q
E □QE □QE □QE □QE □QE □QE □QE □QE □QE □QE □QE □QE □QE □QE □QE □QE □QE □
QE □QE □QE □QE □QE □QE □QE □QE □QE □QE □QE □QE □QE □QE □QE □QE □QE □QE
□QE □QE □QE □QE □QE □QE □QE □QE □QE □QE □QE □QE □QE □QE □QE □QE □QE □QE
□QE □QE □QE □QE □QE □QE □QE □QE □QE □QE □QE □QE □QE □QE □QE □QE □QE □Q
E □QE □QE □QE □QE □QE □QE □QE □QE □QE □QE □QE □QE □QE □QE □QE □QE □QE □
QE □QE □QE □QE □QE □QE □QE □QE □QE □QE □QE □QE □QE □QE □QE □QE □QE □QE
□QE □QE □QE □QE □QE □QE □QE □QE □QE □QE □QE □QE □QE □QE □QE □QE □QE □QE
□QE □QE □QE □QE □QE □QE □QE □QE □QE □QE □QE □QE □QE □QE □QE □QE □QE □Q
E □QE □QE □QE □QE □QE □QE □QE □QE □QE □QE □QE □QE □QE □QE □QE □QE □QE □
QE □QE □QE □QE □QE □QE □QE □QE □QE □QE □QE □QE □QE □QE □QE □QE □QE □QE
□QE □QE □QE □QE □QE □QE □QE □QE □QE □QE □QE □QE □QE □QE □QE □QE □QE □QE
□QE □QE □QE □QE □QE □QE □QE □QE □QE □QE □QE □QE □QE □QE □QE □QE □QE □Q
E □QE □QE □QE □QE □QE □QE □QE □QE □QE □QE □QE □QE □QE □QE □QE □QE □QE □
QE □QE □QE □QE □QE □QE □QE □QE □QE □QE □QE □QE □QE □QE □QE □QE □QE □QE
□QE □QE □QE □QE □QE □QE □QE □QE □QE □QE □QE □QE □QE □QE □QE □QE □QE □QE
□QE □QE □QE □QE □QE □QE □QE □QE □QE □QE □QE □QE □QE □QE □QE □QE □QE □Q
E □QE □QE □QE □QE □QE □QE □QE □QE □QE □QE □QE □W˜ü_ø‘«|>þÆþËµ²ŸíÞ□™ö¥s□
ž^1µ‡÷Ïé^aÿ □âÏú□è¿÷ê_þ9@NÑ_1□ÄGø³þ•z/ýú–ÿ ŽQÿ □âÏú□è¿÷ê_þ9@-
NÑ_1□ÄGø³þ•z/ýú–ÿ ŽQÿ □âÏú□è¿÷ê_þ9@NÑ_1□ÄGø³þ•z/ýú–ÿ ŽQÿ □âÏú□è¿÷ê_þ9@-
NÑ_1□ÄGø³þ•z/ýú–ÿ ŽQÿ □âÏú□è¿÷ê_þ9@NÑ_1□ÄGø³þ•z/ýú–ÿ ŽQÿ □âÏú□è¿÷ê_þ9@-
NÑ_1□ÄGø³þ•z/ýú–ÿ ŽQÿ □âÏú□è¿÷ê_þ9@NÑ_1□ÄGø³þ•z/ýú–ÿ ŽQÿ □âÏú□è¿÷ê_þ9@-
NÑ_1□ÄGø³þ•z/ýú–ÿ ŽQÿ □âÏú□è¿÷ê_þ9@NÑ_1□ÄGø³þ•z/ýú–ÿ ŽQÿ □âÏú□è¿÷ê_þ9@-
NÑ_1□ÄGø³þ•z/ýú–ÿ ŽQÿ □âÏú□è¿÷ê_þ9@NÑ_1□ÄGø³þ•z/ýú–ÿ ŽQÿ □âÏú□è¿÷ê_þ9@-
NÑ_1□ÄGø³þ•z/ýú–ÿ ŽQÿ □âÏú□è¿÷ê_þ9@NÑ_1□ÄGø³þ•z/ýú–ÿ ŽQÿ □âÏú□è¿÷ê_þ9@-
NÑ_1□ÄGø³þ•z/ýú–ÿ ŽQÿ □âÏú□è¿÷ê_þ9@NÑ_1□ÄGø³þ•z/ýú–ÿ ŽQÿ □âÏú□è¿÷ê_þ9@-
NÑ_1□ÄGø³þ•z/ýú–ÿ ŽQÿ □âÏú□è¿÷ê_þ9@NÑ_1□ÄGø³þ•z/ýú–ÿ ŽQÿ □âÏú□è¿÷ê_þ9@-
NÑ_1□ÄGø³þ•z/ýú–ÿ ŽQÿ □âÏú□è¿÷ê_þ9@NÑ_1□ÄGø³þ•z/ýú–ÿ ŽQÿ □âÏú□è¿÷ê_þ9@-
NÑ_1□ÄGø³þ•z/ýú–ÿ ŽQÿ □âÏú□è¿÷ê_þ9@NÑ_1□ÄGø³þ•z/ýú–ÿ ŽQÿ □âÏú□è¿÷ê_þ9@-
NÑ_1□ÄGø³þ•z/ýú–ÿ ŽQÿ □âÏú□è¿÷ê_þ9@NÑ_1□ÄGø³þ•z/ýú–ÿ ŽQÿ □âÏú□è¿÷ê_þ9@-
NÑ_1□ÄGø³þ•z/ýú–ÿ ŽQÿ □âÏú□è¿÷ê_þ9@NÑ_1□ÄGø³þ•z/ýú–ÿ ŽQÿ □âÏú□è¿÷ê_þ9@-
NÑ_1□ÄGø³þ•z/ýú–ÿ ŽQÿ □âÏú□è¿÷ê_þ9@NÑ_1□ÄGø³þ•z/ýú–ÿ ŽQÿ □âÏú□è¿÷ê_þ9@-
NÑ_1□ÄGø³þ•z/ýú–ÿ ŽQÿ □âÏú□è¿÷ê_þ9@NÑ_1□ÄGø³þ•z/ýú–ÿ ŽQÿ □âÏú□è¿÷ê_þ9@-
NÑ_1□ÄGø³þ•z/ýú–ÿ ŽQÿ □âÏú□è¿÷ê_þ9@NÑ_1□ÄGø³þ•z/ýú–ÿ ŽQÿ □âÏú□è¿÷ê_þ9@-
NÑ_1□ÄGø³þ•z/ýú–ÿ ŽQÿ □âÏú□è¿÷ê_þ9@NÑ_1□ÄGø³þ•z/ýú–ÿ ŽQÿ □âÏú□è¿÷ê_þ9@-
NÑ_1□ÄGø³þ•z/ýú–ÿ ŽQÿ □âÏú□è¿÷ê_þ9@NÑ_1□ÄGø³þ•z/ýú–ÿ ŽQÿ □âÏú□è¿÷ê_þ9@-
NÑ_1□ÄGø³þ•z/ýú–ÿ ŽQÿ □âÏú□è¿÷ê_þ9@NÑ_1□ÄGø³þ•z/ýú–ÿ ŽQÿ □âÏú□è¿÷ê_þ9@-
NÑ_1□ÄGø³þ•z/ýú–ÿ ŽQÿ □âÏú□è¿÷ê_þ9@NÑ_1□ÄGø³þ•z/ýú–ÿ ŽQÿ □âÏú□è¿÷ê_þ9@-

NÑ_1□ÃGø³þ•z/ýú—ÿ ŽQÿ □âÏú□è¿÷ê_þ9@NÑ_1□ÃGø³þ•z/ýú—ÿ ŽQÿ □âÏú□è¿÷ê_þ9@-
NÑ_1□ÃGø³þ•z/ýú—ÿ ŽQÿ □âÏú□è¿÷ê_þ9@NÑ_1□ÃGø³þ•z/ýú—ÿ ŽQÿ □âÏú□è¿÷ê_þ9@-
NÑ_1□ÃGø³þ•z/ýú—ÿ ŽQÿ □âÏú□è¿÷ê_þ9@NÑ_1□ÃGø³þ•z/ýú—ÿ ŽQÿ □âÏú□è¿÷ê_þ9@-
NÑ_1□ÃGø³þ•z/ýú—ÿ ŽQÿ □âÏú□è¿÷ê_þ9@NÑ_1□ÃGø³þ•z/ýú—ÿ ŽQÿ □âÏú□è¿÷ê_þ9@-
NÑ_1□ÃGø³þ•z/ýú—ÿ ŽQÿ □âÏú□è¿÷ê_þ9@NÑ_1□ÃGø³þ•z/ýú—ÿ ŽQÿ □âÏú□è¿÷ê_þ9@-
NÑ_1□ÃGø³þ•z/ýú—ÿ ŽQÿ □âÏú□è¿÷ê_þ9@NÑ_1□ÃGø³þ•z/ýú—ÿ ŽQÿ □âÏú□è¿÷ê_þ9@-
NÑ_1□ÃGø³þ•z/ýú—ÿ ŽQÿ □âÏú□è¿÷ê_þ9@NÑ_1□ÃGø³þ•z/ýú—ÿ ŽQÿ □âÏú□è¿÷ê_þ9@-
NÑ_1□ÃGø³þ•z/ýú—ÿ ŽQÿ □âÏú□è¿÷ê_þ9@NÑ_1□ÃGø³þ•z/ýú—ÿ ŽQÿ □âÏú□è¿÷ê_þ9@-
NÑ_1□ÃGø³þ•z/ýú—ÿ ŽQÿ □âÏú□è¿÷ê_þ9@NÑ_1□ÃGø³þ•z/ýú—ÿ ŽQÿ □âÏú□è¿÷ê_þ9@-
NÑ_1□ÃGø³þ•z/ýú—ÿ ŽQÿ □âÏú□è¿÷ê_þ9@NÑ_1□ÃGø³þ•z/ýú—ÿ ŽQÿ □âÏú□è¿÷ê_þ9@-
NÑ_1□ÃGø³þ•z/ýú—ÿ ŽQÿ □âÏú□è¿÷ê_þ9@NÑ_1□ÃGø³þ•z/ýú—ÿ ŽQÿ □âÏú□è¿÷ê_þ9@-
NÑ_1□ÃGø³þ•z/ýú—ÿ ŽQÿ □âÏú□è¿÷ê_þ9@NÑ_1□ÃGø³þ•z/ýú—ÿ ŽQÿ □âÏú□è¿÷ê_þ9@-
NÑ_1□ÃGø³þ•z/ýú—ÿ ŽQÿ □âÏú□è¿÷ê_þ9@NÑ_1□ÃGø³þ•z/ýú—ÿ ŽQÿ □âÏú□è¿÷ê_þ9@-
NÑ_1□ÃGø³þ•z/ýú—ÿ ŽQÿ □âÏú□è¿÷ê_þ9@NÑ_1□ÃGø³þ•z/ýú—ÿ ŽQÿ □âÏú□è¿÷ê_þ9@-
NÑ_1□ÃGø³þ•z/ýú—ÿ ŽQÿ □âÏú□è¿÷ê_þ9@NÑ_1□ÃGø³þ•z/ýú—ÿ ŽQÿ □âÏú□è¿÷ê_þ9@-
NÑ_1□ÃGø³þ•z/ýú—ÿ ŽQÿ □âÏú□è¿÷ê_þ9@NÑ_1□ÃGø³þ•z/ýú—ÿ ŽQÿ □âÏú□è¿÷ê_þ9@-
NÑ_1□ÃGø³þ•z/ýú—ÿ ŽQÿ □âÏú□è¿÷ê_þ9@NÑ_1□ÃGø³þ•z/ýú—ÿ ŽQÿ □âÏú□è¿÷ê_þ9@-
NÑ_1□ÃGø³þ•z/ýú—ÿ ŽQÿ □âÏú□è¿÷ê_þ9@NÑ_1□ÃGø³þ•z/ýú—ÿ ŽQÿ □âÏú□è¿÷ê_þ9@-
NÑ_1□ÃGø³þ•z/ýú—ÿ ŽQÿ □âÏú□è¿÷ê_þ9@NÑ_1□ÃGø³þ•z/ýú—ÿ ŽQÿ □âÏú□è¿÷ê_þ9@-
NÑ_1□ÃGø³þ•z/ýú—ÿ ŽQÿ □âÏú□è¿÷ê_þ9@NÑ_1□ÃGø³þ•z/ýú—ÿ ŽQÿ □âÏú□è¿÷ê_þ9@-
NÑ_1□ÃGø³þ•z/ýú—ÿ ŽQÿ □âÏú□è¿÷ê_þ9@NÑ_1□ÃGø³þ•z/ýú—ÿ ŽQÿ □âÏú□è¿÷ê_þ9@-
NÑ_1□ÃGø³þ•z/ýú—ÿ ŽQÿ □âÏú□è¿÷ê_þ9@NÑ_1□ÃGø³þ•z/ýú—ÿ ŽQÿ □âÏú□è¿÷ê_þ9@-
NÑ_1□ÃGø³þ•z/ýú—ÿ ŽQÿ □âÏú□è¿÷ê_þ9@NÑ_1□ÃGø³þ•z/ýú—ÿ ŽQÿ □âÏú□è¿÷ê_þ9@-
NÑ_1□ÃGø³þ•z/ýú—ÿ ŽQÿ □âÏú□è¿÷ê_þ9@NÑ_1□ÃGø³þ•z/ýú—ÿ ŽQÿ □âÏú□è¿÷ê_þ9@-
NÑ_1□ÃGø³þ•z/ýú—ÿ ŽQÿ □âÏú□è¿÷ê_þ9@NÑ_1□ÃGø³þ•z/ýú—ÿ ŽQÿ □âÏú□è¿÷ê_þ9@-
NÑ_1□ÃGø³þ•z/ýú—ÿ ŽQÿ □âÏú□è¿÷ê_þ9@NÑ_1□ÃGø³þ•z/ýú—ÿ ŽQÿ □âÏú□è¿÷ê_þ9@-
NÑ_1□ÃGø³þ•z/ýú—ÿ ŽQÿ □âÏú□è¿÷ê_þ9@NÑ_1□ÃGø³þ•z/ýú—ÿ ŽQÿ □âÏú□è¿÷ê_þ9@-
NÑ_1□ÃGø³þ•z/ýú—ÿ ŽQÿ □âÏú□è¿÷ê_þ9@NÑ_1□ÃGø³þ•z/ýú—ÿ ŽQÿ □âÏú□è¿÷ê_þ9@-
NÑ_1□ÃGø³þ•z/ýú—ÿ ŽQÿ □âÏú□è¿÷ê_þ9@NÑ_1□ÃGø³þ•z/ýú—ÿ ŽQÿ □âÏú□è¿÷ê_þ9@-
NÑ_1□ÃGø³þ•z/ýú—ÿ ŽQÿ □âÏú□è¿÷ê_þ9@NÑ_1□ÃGø³þ•z/ýú—ÿ ŽQÿ □âÏú□è¿÷ê_þ9@-
NÑ_1□ÃGø³þ•z/ýú—ÿ ŽQÿ □âÏú□è¿÷ê_þ9@NÑ_1□ÃGø³þ•z/ýú—ÿ ŽQÿ □âÏú□è¿÷ê_þ9@-
NÑ_1□ÃGø³þ•z/ýú—ÿ ŽQÿ □âÏú□è¿÷ê_þ9@NÑ_1□ÃGø³þ•z/ýú—ÿ ŽQÿ □âÏú□è¿÷ê_þ9@-
NÑ_1□ÃGø³þ•z/ýú—ÿ ŽQÿ □âÏú□è¿÷ê_þ9@NÑ_1□ÃGø³þ•z/ýú—ÿ ŽQÿ □âÏú□è¿÷ê_þ9@-
NÑ_1□ÃGø³þ•z/ýú—ÿ ŽQÿ □âÏú□è¿÷ê_þ9@NÑ_1□ÃGø³þ•z/ýú—ÿ ŽQÿ □âÏú□è¿÷ê_þ9@-
NÑ_1□ÃGø³þ•z/ýú—ÿ ŽQÿ □âÏú□è¿÷ê_þ9@NÑ_1□ÃGø³þ•z/ýú—ÿ ŽQÿ □âÏú□è¿÷ê_þ9@-
NÑ_1□ÃGø³þ•z/ýú—ÿ ŽQÿ □âÏú□è¿÷ê_þ9@NÑ_1□ÃGø³þ•z/ýú—ÿ ŽQÿ □âÏú□è¿÷ê_þ9@-
NÑ_1□ÃGø³þ•z/ýú—ÿ ŽQÿ □âÏú□è¿÷ê_þ9@NÑ_1□ÃGø³þ•z/ýú—ÿ ŽQÿ □âÏú□è¿÷ê_þ9@-
NÑ_1□ÃGø³þ•z/ýú—ÿ ŽQÿ □âÏú□è¿÷ê_þ9@NÑ_1□ÃGø³þ•z/ýú—ÿ ŽQÿ □âÏú□è¿÷ê_þ9@-
NÑ_1□ÃGø³þ•z/ýú—ÿ ŽQÿ □âÏú□è¿÷ê_þ9@NÑ_1□ÃGø³þ•z/ýú—ÿ ŽQÿ □âÏú□è¿÷ê_þ9@-
NÑ_1□ÃGø³þ•z/ýú—ÿ ŽQÿ □âÏú□è¿÷ê_þ9@NÑ_1□ÃGø³þ•z/ýú—ÿ ŽQÿ □âÏú□è¿÷ê_þ9@-
NÑ_1□ÃGø³þ•z/ýú—ÿ ŽQÿ □âÏú□è¿÷ê_þ9@NÑ_1□ÃGø³þ•z/ýú—ÿ ŽQÿ □âÏú□è¿÷ê_þ9@-
NÑ_1□ÃGø³þ•z/ýú—ÿ ŽQÿ □âÏú□è¿÷ê_þ9@NÑ_1□ÃGø³þ•z/ýú—ÿ ŽQÿ □âÏú□è¿÷ê_þ9@-
NÑ_1□ÃGø³þ•z/ýú—ÿ ŽQÿ □âÏú□è¿÷ê_þ9@NÑ_1□ÃGø³þ•z/ýú—ÿ ŽQÿ □âÏú□è¿÷ê_þ9@-
NÑ_1□ÃGø³þ•z/ýú—ÿ ŽQÿ □âÏú□è¿÷ê_þ9@NÑ_1□ÃGø³þ•z/ýú—ÿ ŽQÿ □âÏú□è¿÷ê_þ9@-
NÑ_1□ÃGø³þ•z/ýú—ÿ ŽQÿ □âÏú□è¿÷ê_þ9@NÑ_1□ÃGø³þ•z/ýú—ÿ ŽWÐ>

Ön<Eàý+X°Ž(ç»·Y]b□('Ó$œ~4□¹E□P□E□P□E□P□E□P□E□P□E□P□E□P□E□P□E□P□E□P□E□P□
E□P□E□P□E□P□E□P□E□P□E□P□E□P□E□P□E□P□E□P□E□P□E□P□E□P□E□P□E□P□E□P□E□P□‡~Ñ
□&-¬□Â7ý—¥Þßy_jó>ËnòìÏ•Œí□□Áü•xwü!>+ÿ ¡cZÿ À
□øšûŠŠ øwþ□Ÿ□ÿ Ð±-□a□¿üM-
ð„ø¯þ…•kÿ %ÿ âkî*(□áßøB|Wÿ BÆµÿ €□ÿ ñ4□Â□â¿ú□5¯ü —ÿ ‰¯¸¨ □‡□añ_ý
□×þ Kÿ ÄÑÿ □OŠÿ èXÖ¿ð□_þ&¾â¢€>□ÿ „'Å□ô,k_ø□/ÿ □Gü!>+ÿ ¡cZÿ À
□øšûŠŠ øwþ□Ÿ□ÿ Ð±-□a□¿üM-
ð„ø¯þ…•kÿ %ÿ âkî*(□áßøB|Wÿ BÆµÿ €□ÿ ñ4□Â□â¿ú□5¯ü —ÿ ‰¯¸¨ □‡□añ_ý
□×þ Kÿ ÄÑÿ □OŠÿ èXÖ¿ð□_þ&¾â¢€>□ÿ „'Å□ô,k_ø□/ÿ □Gü!>+ÿ ¡cZÿ À
□øšûŠŠ øwþ□Ÿ□ÿ Ð±-□a□¿üM-
ð„ø¯þ…•kÿ %ÿ âkî*(□áßøB|Wÿ BÆµÿ €□ÿ ñ4□Â□â¿ú□5¯ü —ÿ ‰¯¸¨ □‡□añ_ý
□×þ Kÿ ÄÑÿ □OŠÿ èXÖ¿ð□_þ&¾â¢€>□ÿ „'Å□ô,k_ø□/ÿ □Gü!>+ÿ ¡cZÿ À
□øšûŠŠ øwþ□Ÿ□ÿ Ð±-□a□¿üM-
ð„ø¯þ…•kÿ %ÿ âkî*(□áßøB|Wÿ BÆµÿ €□ÿ ñ4□Â□â¿ú□5¯ü —ÿ ‰¯¸¨ □‡□añ_ý
□×þ Kÿ ÄÑÿ □OŠÿ èXÖ¿ð□_þ&¾â¢€>□ÿ „'Å□ô,k_ø□/ÿ □Gü!>+ÿ ¡cZÿ À
□øšûŠŠ øwþ□Ÿ□ÿ Ð±-□a□¿üM-
ð„ø¯þ…•kÿ %ÿ âkî*(□áßøB|Wÿ BÆµÿ €□ÿ ñ4□Â□â¿ú□5¯ü —ÿ ‰¯¸¨ □‡□añ_ý
□×þ Kÿ ÄÑÿ □OŠÿ èXÖ¿ð□_þ&¾â¢€>□ÿ „'Å□ô,k_ø□/ÿ □Gü!>+ÿ ¡cZÿ À
□øšûŠŠ øwþ□Ÿ□ÿ Ð±-□a□¿üM-
ð„ø¯þ…•kÿ %ÿ âkî*(□áßøB|Wÿ BÆµÿ €□ÿ ñ4□Â□â¿ú□5¯ü —ÿ ‰¯¸¨ □‡□añ_ý
□×þ Kÿ ÄÑÿ □OŠÿ èXÖ¿ð□_þ&¾â¢€>□ÿ „'Å□ô,k_ø□/ÿ □Gü!>+ÿ ¡cZÿ À
□øšûŠŠ øwþ□Ÿ□ÿ Ð±-□a□¿üM-
ð„ø¯þ…•kÿ %ÿ âkî*(□áßøB|Wÿ BÆµÿ €□ÿ ñ4□Â□â¿ú□5¯ü —ÿ ‰¯¸¨ □‡□añ_ý
□×þ Kÿ ÄÑÿ □OŠÿ èXÖ¿ð□_þ&¾â¢€>□ÿ „'Å□ô,k_ø□/ÿ □Gü!>+ÿ ¡cZÿ À
□øšûŠŠ øwþ□Ÿ□ÿ Ð±-□a□¿üM-
ð„ø¯þ…•kÿ %ÿ âkî*(□áßøB|Wÿ BÆµÿ €□ÿ ñ4□Â□â¿ú□5¯ü —ÿ ‰¯¸¨ □‡□añ_ý
□×þ Kÿ ÄÑÿ □OŠÿ èXÖ¿ð□_þ&¾â¢€>□ÿ „'Å□ô,k_ø□/ÿ □Gü!>+ÿ ¡cZÿ À
□øšûŠŠ øwþ□Ÿ□ÿ Ð±-□a□¿üM-
ð„ø¯þ…•kÿ %ÿ âkî*(□áßøB|Wÿ BÆµÿ €□ÿ ñ4□Â□â¿ú□5¯ü —ÿ ‰¯¸¨ □‡□añ_ý
□×þ Kÿ ÄÑÿ □OŠÿ èXÖ¿ð□_þ&¾â¢€>□ÿ „'Å□ô,k_ø□/ÿ □Gü!>+ÿ ¡cZÿ À
□øšûŠŠ øwþ□Ÿ□ÿ Ð±-□a□¿üM-
ð„ø¯þ…•kÿ %ÿ âkî*(□áßøB|Wÿ BÆµÿ €□ÿ ñ4□Â□â¿ú□5¯ü —ÿ ‰¯¸¨ □‡□añ_ý
□×þ Kÿ ÄÑÿ □OŠÿ èXÖ¿ð□_þ&¾â¢€>□ÿ „'Å□ô,k_ø□/ÿ □Gü!>+ÿ ¡cZÿ À
□øšûŠŠ øwþ□Ÿ□ÿ Ð±-□a□¿üM-
ð„ø¯þ…•kÿ %ÿ âkî*(□áßøB|Wÿ BÆµÿ €□ÿ ñ4□Â□â¿ú□5¯ü —ÿ ‰¯¸¨ □‡□añ_ý
□×þ Kÿ ÄÑÿ □OŠÿ èXÖ¿ð□_þ&¾â¢€>□ÿ „'Å□ô,k_ø□/ÿ □Gü!>+ÿ ¡cZÿ À
□øšûŠŠ øwþ□Ÿ□ÿ Ð±-□a□¿üM-
ð„ø¯þ…•kÿ %ÿ âkî*(□áßøB|Wÿ BÆµÿ €□ÿ ñ4□Â□â¿ú□5¯ü —ÿ ‰¯¸¨ □‡□añ_ý
□×þ Kÿ ÄÑÿ □OŠÿ èXÖ¿ð□_þ&¾â¢€>□ÿ „'Å□ô,k_ø□/ÿ □Gü!>+ÿ ¡cZÿ À
□øšûŠŠ øwþ□Ÿ□ÿ Ð±-□a□¿üM-
ð„ø¯þ…•kÿ %ÿ âkî*(□áßøB|Wÿ BÆµÿ €□ÿ ñ4□Â□â¿ú□5¯ü —ÿ ‰¯¸¨ □‡□añ_ý
□×þ Kÿ ÄÑÿ □OŠÿ èXÖ¿ð□_þ&¾â¢€>□ÿ „'Å□ô,k_ø□/ÿ □Gü!>+ÿ ¡cZÿ À
□øšûŠŠ øwþ□Ÿ□ÿ Ð±-□a□¿üM-
ð„ø¯þ…•kÿ %ÿ âkî*(□áßøB|Wÿ BÆµÿ €□ÿ ñ4□Â□â¿ú□5¯ü —ÿ ‰¯¸¨ □‡□añ_ý
□×þ Kÿ ÄÑÿ □OŠÿ èXÖ¿ð□_þ&¾â¢€>□ÿ „'Å□ô,k_ø□/ÿ □Gü!>+ÿ ¡cZÿ À
□øšûŠŠ øwþ□Ÿ□ÿ Ð±-□a□¿üM-
ð„ø¯þ…•kÿ %ÿ âkî*(□áßøB|Wÿ BÆµÿ €□ÿ ñ4□Â□â¿ú□5¯ü —ÿ ‰¯¸¨ □‡□añ_ý
□×þ Kÿ ÄÑÿ □OŠÿ èXÖ¿ð□_þ&¾â¢€>□ÿ „'Å□ô,k_ø□/ÿ □Gü!>+ÿ ¡cZÿ À
□øšûŠŠ øwþ□Ÿ□ÿ Ð±-□a□¿üM-
ð„ø¯þ…•kÿ %ÿ âkî*(□áßøB|Wÿ BÆµÿ €□ÿ ñ4□Â□â¿ú□5¯ü —ÿ ‰¯¸¨ □‡□añ_ý
□×þ Kÿ ÄÑÿ □OŠÿ èXÖ¿ð□_þ&¾â¢€>□ÿ „'Å□ô,k_ø□/ÿ □Gü!>+ÿ ¡cZÿ À
□øšûŠŠ øwþ□Ÿ□ÿ Ð±-□a□¿üM-
ð„ø¯þ…•kÿ %ÿ âkî*(□áßøB|Wÿ BÆµÿ €□ÿ ñ4□Â□â¿ú□5¯ü —ÿ ‰¯¸¨ □‡□añ_ý
□×þ Kÿ ÄÑÿ □OŠÿ èXÖ¿ð□_þ&¾â¢€>□ÿ „'Å□ô,k_ø□/ÿ □Gü!>+ÿ ¡cZÿ À
□øšûŠŠ øwþ□Ÿ□ÿ Ð±-□a□¿üM-
ð„ø¯þ…•kÿ %ÿ âkî*(□áßøB|Wÿ BÆµÿ €□ÿ ñ4□Â□â¿ú□5¯ü —ÿ ‰¯¸¨ □‡□añ_ý
□×þ Kÿ ÄÑÿ □OŠÿ èXÖ¿ð□_þ&¾â¢€>□ÿ „'Å□ô,k_ø□/ÿ □Gü!>+ÿ ¡cZÿ À
□øšûŠŠ øwþ□Ÿ□ÿ Ð±-□a□¿üM-

ð„ø¯þ…•kÿ %ÿ âkî*(□áßøB|Wÿ BÆµÿ €□ÿ ñ4□Â□â¿ú□5¯ü –ÿ ‰¯¸¨ □‡□áñ_ý
□×þ Kÿ ÄÑÿ □OŠÿ èXÖ¿ð□_þ&¾â¢€>□ÿ „'Å□ð,k_ø□/ÿ □Gü!>+ÿ ¡cZÿ À
□øšûŠŠ øwþ□Ÿ□ÿ Ð±-□à□¿üM-
ð„ø¯þ…•kÿ %ÿ âkî*(□áßøB|Wÿ BÆµÿ €□ÿ ñ4□Â□â¿ú□5¯ü –ÿ ‰¯¸¨ □‡□áñ_ý
□×þ Kÿ ÄÑÿ □OŠÿ èXÖ¿ð□_þ&¾â¢€>□ÿ „'Å□ð,k_ø□/ÿ □Gü!>+ÿ ¡cZÿ À
□øšûŠŠ øwþ□Ÿ□ÿ Ð±-□à□¿üM-
ð„ø¯þ…•kÿ %ÿ âkî*(□áßøB|Wÿ BÆµÿ €□ÿ ñ4□Â□â¿ú□5¯ü –ÿ ‰¯¸¨ □‡□áñ_ý
□×þ Kÿ ÄÑÿ □OŠÿ èXÖ¿ð□_þ&¾â¢€>□ÿ „'Å□ð,k_ø□/ÿ □Gü!>+ÿ ¡cZÿ À
□øšûŠŠ øwþ□Ÿ□ÿ Ð±-□à□¿üM-
ð„ø¯þ…•kÿ %ÿ âkî*(□áßøB|Wÿ BÆµÿ €□ÿ ñ4□Â□â¿ú□5¯ü –ÿ ‰¯¸¨ □‡□áñ_ý
□×þ Kÿ ÄÑÿ □OŠÿ èXÖ¿ð□_þ&¾â¢€>□ÿ „'Å□ð,k_ø□/ÿ □Gü!>+ÿ ¡cZÿ À
□øšûŠŠ øwþ□Ÿ□ÿ Ð±-□à□¿üM-
ð„ø¯þ…•kÿ %ÿ âkî*(□áßøB|Wÿ BÆµÿ €□ÿ ñ4□Â□â¿ú□5¯ü –ÿ ‰¯¸¨ □‡□áñ_ý
□×þ Kÿ ÄÑÿ □OŠÿ èXÖ¿ð□_þ&¾â¢€>□ÿ „'Å□ð,k_ø□/ÿ □Gü!>+ÿ ¡cZÿ À
□øšûŠŠ øwþ□Ÿ□ÿ Ð±-□à□¿üM-
ð„ø¯þ…•kÿ %ÿ âkî*(□áßøB|Wÿ BÆµÿ €□ÿ ñ4□Â□â¿ú□5¯ü –ÿ ‰¯¸¨ □‡□áñ_ý
□×þ Kÿ ÄÑÿ □OŠÿ èXÖ¿ð□_þ&¾â¢€>□ÿ „'Å□ð,k_ø□/ÿ □Gü!>+ÿ ¡cZÿ À
□øšûŠŠ øwþ□Ÿ□ÿ Ð±-□à□¿üM-
ð„ø¯þ…•kÿ %ÿ âkî*(□áßøB|Wÿ BÆµÿ €□ÿ ñ4□Â□â¿ú□5¯ü –ÿ ‰¯¸¨ □‡□áñ_ý
□×þ Kÿ ÄÑÿ □OŠÿ èXÖ¿ð□_þ&¾â¢€>□ÿ „'Å□ð,k_ø□/ÿ □Gü!>+ÿ ¡cZÿ À
□øšûŠŠ øwþ□Ÿ□ÿ Ð±-□à□¿üM-
ð„ø¯þ…•kÿ %ÿ âkî*(□áßøB|Wÿ BÆµÿ €□ÿ ñ4□Â□â¿ú□5¯ü –ÿ ‰¯¸¨ □‡□áñ_ý
□×þ Kÿ ÄÑÿ □OŠÿ èXÖ¿ð□_þ&¾â¢€>□ÿ „'Å□ð,k_ø□/ÿ □Gü!>+ÿ ¡cZÿ À
□øšûŠŠ øwþ□Ÿ□ÿ Ð±-□à□¿üM-
ð„ø¯þ…•kÿ %ÿ âkî*(□áßøB|Wÿ BÆµÿ €□ÿ ñ4□Â□â¿ú□5¯ü –ÿ ‰¯¸¨ □‡□áñ_ý
□×þ Kÿ ÄÑÿ □OŠÿ èXÖ¿ð□_þ&¾â¢€>□ÿ „'Å□ð,k_ø□/ÿ □Gü!>+ÿ ¡cZÿ À
□øšûŠŠ øwþ□Ÿ□ÿ Ð±-□à□¿üM-
ð„ø¯þ…•kÿ %ÿ âkî*(□áßøB|Wÿ BÆµÿ €□ÿ ñ4□Â□â¿ú□5¯ü –ÿ ‰¯¸¨ □‡□áñ_ý
□×þ Kÿ ÄÑÿ □OŠÿ èXÖ¿ð□_þ&¾â¢€>□ÿ „'Å□ð,k_ø□/ÿ □Gü!>+ÿ ¡cZÿ À
□øšûŠŠ øwþ□Ÿ□ÿ Ð±-□à□¿üM-
ð„ø¯þ…•kÿ %ÿ âkî*(□áßøB|Wÿ BÆµÿ €□ÿ ñ4□Â□â¿ú□5¯ü –ÿ ‰¯¸¨ □‡□áñ_ý
□×þ Kÿ ÄÑÿ □OŠÿ èXÖ¿ð□_þ&¾â¢€>□ÿ „'Å□ð,k_ø□/ÿ □Gü!>+ÿ ¡cZÿ À
□øšûŠŠ øwþ□Ÿ□ÿ Ð±-□à□¿üM-
ð„ø¯þ…•kÿ %ÿ âkî*(□áßøB|Wÿ BÆµÿ €□ÿ ñ4□Â□â¿ú□5¯ü –ÿ ‰¯¸¨ □‡□áñ_ý
□×þ Kÿ ÄÑÿ □OŠÿ èXÖ¿ð□_þ&¾â¢€>□ÿ „'Å□ð,k_ø□/ÿ □Gü!>+ÿ ¡cZÿ À
□øšûŠŠ øwþ□Ÿ□ÿ Ð±-□à□¿üM-
ð„ø¯þ…•kÿ %ÿ âkî*(□áßøB|Wÿ BÆµÿ €□ÿ ñ4□Â□â¿ú□5¯ü –ÿ ‰¯¸¨ □‡□áñ_ý
□×þ Kÿ ÄÑÿ □OŠÿ èXÖ¿ð□_þ&¾â¢€>□ÿ „'Å□ð,k_ø□/ÿ □Gü!>+ÿ ¡cZÿ À
□øšûŠŠ øwþ□Ÿ□ÿ Ð±-□à□¿üM-
ð„ø¯þ…•kÿ %ÿ âkî*(□áßøB|Wÿ BÆµÿ €□ÿ ñ4□Â□â¿ú□5¯ü –ÿ ‰¯¸¨ □‡□áñ_ý
□×þ Kÿ ÄÑÿ □OŠÿ èXÖ¿ð□_þ&¾â¢€>□ÿ „'Å□ð,k_ø□/ÿ □Gü!>+ÿ ¡cZÿ À
□øšûŠŠ øwþ□Ÿ□ÿ Ð±-□à□¿üM-
ð„ø¯þ…•kÿ %ÿ âkî*(□áßøB|Wÿ BÆµÿ €□ÿ ñ4□Â□â¿ú□5¯ü –ÿ ‰¯¸¨ □‡□áñ_ý
□×þ Kÿ Ä××_

-
n,¾□h6×PK□ñÚ*¼R¡VSèAä□ê(□Š(□Š(□Š(□Š(□Š(□Š(□Š(□Š(□Š(□Š(□Š(□Š
(□Š(□Š(□Š(□Š(□Š(□Š(□Š(□Š(□Š(□Š(□Š(□Š(□Š(□Š(□Š(□Š(□Š(□
Š(□Š(□Š(□Š(□Š(□Š(□Š(□Š(□Š(□Š(□Š(□Š(□Š(□Š(□Š(□Š(□Š(□Š(
□Š(□Š(□Š(□Š(□Š(□Š(□Š(□Š(□Š(□Š(□Š(□Š(□Š(□Š(□Š(□Š(□Š(□Š(
□Š(□Š(□Š(□Š(□Š(□Š(□Š(□±<]â[□□x^÷]°†I¡µ
˜âÆæ,á□_v□·^}ñ»þI□·õ·ÿ ÑñÐ□¿•<{¦xûI–öÁ$,XËžÞR
!<ƒÇPyÁö>”¾<ñ½¯€´H5;»I®’k•·T„€A*Í“žØS^□áÖŸáfµá•□©s kÖq¥àä„r□ïÄ□8öÜ□w_´K
¬ž Ò•□26©□V□ •*^h□×¡•g,9”□²(až¸#4úå<IâK¯
x2ÖúÇI¹Õ.ä□A□´
NY—,Ø□ã□Ï□¾kÏ5/□üfÑtéuëý□K[□WÌšÙ@c□w$□Ý•Äã½ {.£s%ž™wu□»ÜK

/"BŸzB□•£Üã□Íü<ñ^¥ã□□Í¨jš;é“%ËB±°l:€§pÜ□êHü?□?‡ü]‰|□‰-
!ò™íä□Âç;$L,¹ã#+×ŒŒV?Â/□jž4ð„úŽ®a71Þ¼ Ä›□P¨Ã#×æ4□ßQ_?X|Xñþ»â□KÃ°-›eu|.Ÿ
Ê˜¦Õ,□$□Ù8þï$û`’1ßCã=sÂ_□®õŸYÁ

¡ÁŠm•ÕA gB@$îÏ \â€=

Šñ;O□üf×ì—YÓ4=6

G™□□m□"-‡ælôïÆzŠë>□|Ho□

Ý;S²□□îžqqn2□

à•□#□,

q‘Ï< AáŸ□jú¯Åÿ □xfçÈþÎ±€É XðàtAÉÏ9ÜkÑëæ©¼I«øsã¯‰Îƒ¥□KT¾□ko□ÎÕbQ·□:€□÷□ë
ž+¥»ø‰ñ□À·¶w>6ÑìäÒ.¤ØÒZíÝ□ë€U^È□8=ppz•□é-?ñ6¡á?

6§¦éO©\ ’?)s…□9c□œq□ÄVÖ‹}6§¡X_ÜÚ½¤÷6ñË%»ç13(%NpxÎ:
å~&x°óÃ□□Ÿ]Ñ%…¥w‹Ê‘×z”sÔ□q]/‡oæÕ|1¤ê7□D÷vpÏ
A•¹•1Ç¶M iÑE□ QE□ QE□ QE□ QE□ QE□ QE□ QE□ QE□ QE□ QE□ QE□ QE□ QE□ QE□ Q
E□ QE□ QE□ QE□ QE□ QE□ QE□ QE□ QE□ QE□ QE□ QE□ QE□ QE□ QE□ QE□ QE□ QE□
QE□ QE□ QE□ QE□ QE□ QE□ QE□ QE□ QE□ QE□ QE□ QE□ QE□ QE□ QE□ W-
ãß□Úx□A‹U»µšée¸[tŽ"□ÜU›$žƒ
□JêkÈ?hÏù□´ßû
Çÿ ¢¥

ÿ Áž/ÓüoáäÕôå’4ÞÑI□˜Ý□Ž ãØƒô"°
ðo†2?€þ)j>□□˜Xj‘-Å‘lã;w®3þÉe'¹Q^½âÿ □Ãá_ ê:ÔØ?fˆ˜Ôÿ □‡„_Åˆ

ORøÇ¢éþ>‹Âkisq3\Çk%Ìev$Œ@Æ3‟,@?C^□_$Xè7□~¯à=rý□¯õÍWíR3õ)çG´Ÿs-l÷

+è•-ø«Wðå-”□

…6-©ßÈÑÂª¤Ç□ 9r;sê:□'Š ëè¯□Õ¼mñkÁ¶é¬ø‡FÓfÒ÷¨™"+˜òzeX'é"'šõ;Ÿ□é–
ž□-,™™tóh·@□7□Àm_÷‰!~¦€7ë□Æº½Öƒà½[U²Ùö›[vx÷®@oq^_cã/‹~-
´:×‡ô=:ßKf&□åeÝ(□□Y†~¸QZ·þ%ÕüMð_Å□ëº;éz□ª=¼±•eW)Ü¡¹□ŸSÓ- v

uûß□x□JÖu/íw*þa□v©+#(ãè¢°šùÇáçŠ>!_ø:ËEðf□j-´åušöäŒ;³³í]Ä

Œ□9=

FkÑ¾□|DÔ|M©j^-ñ□ŒvZö•-
'c□+¨m§Œœ□Jô8;□□□é□W•ëž.ø…«x†ûJðw‡□□[□L2_ß□¢F□Óq□MÇ□<f¡ð□Ä_□Çã¤ðo•´Ø-
ï§Œ½¼ðp□ ·8$□@8#□#□z iøçÆÚ¿‡þ!xKE±ò>Ç©Î±Ü‡□,CH□fž85èõâ¿□?ä°ü>ÿ ¯¨¿ôrÖ—
<>%ê×¾,‡Â>□†□ÍM_ý.îEÝ□ u□éÇsÛ É □X¢¸¿□xò/‡þ□¶¹¾D¼Õ'□(`•äYd nnä(üO
wÍq□¯ün□_Úÿ Ø:w`·Ìû□Á¿n3÷wïÎ?‡;½¨□Úè®3á÷Ä+?□ørmCË□·V‡mä□·□8È`□º@8ú□Û'f²
ñ÷Ä¯□Éy□àÝ&Â□"Þc□□-]ò□□Á,ÝpAà 3Œž´□íôW=à½CÄ:—‡-
o□é'éúšÊèñFr¬ ðÃ"ŒýOL÷®†€
(¢€
(¢€
(¢€
(¢€
(¢€
(¢€
(¢€
(¢€
(¢€
(¢€
(¢€
(¢€
(¢€
(¢€
(¢€
(¢€
(¢€
(¢€
(¢€
(¢€
(¢€
(¢€
(¢€
(¢€
(¢€
(¢€
(¢€
(¢€
(¢€
(¢€
(¢€
(¢€
(¢€
(¢€
(¢€
(¢€
(¢€
(¢€
(¢€
(¢€
(¢€
(¢€
(¢€
(¢€
(¢€
(¢€
(¢€
(¢€
(¢€
(¢€
(¢€

(¢€
(¢€
(¢€
(¢€
(¢€
(¢€
(¢€
(¢€
(¢€
(¢€
(¢€
(¢€
(¢€
(¢€
(¢€
(¢€
(¢€
(¢€
(¢€
(¢€
(¢€
(¢€
(¢€
(¢€
(¢€
(¢€
(¢€
(¢€
(¢€
(¢€
(¢€
(¢€
(¢€
(¢€
(¢€
(¢€
(¢€
(¢€
(¢€
(¢€
(¢€
(¢€
óï□ßòHµ¿-¿þ□Ž½□¹?‰~-¾ñWÃýOFÓDfîãÊ1‰□j•²£-~Šh□□ÃÂ-þ3ø□¥hómY-
O□íäaþ®P>Vþ‡ØšñO□x¦â÷áe·„õ}Ñêú-®□ìsó□„r¨-
ð□òý6×Ó^□ÒîtO□é□eÞÏ´ÚŰ¤Rl9□€ç□½y‡Å□ƒ·þ(ñ

Zç‡¾Ì³Ì^E+ì‡ÁÁíÁú

¹4□Øø×Çpx□ÁvWíoö«©Õ!¶ƒvÐÍ·$±ô□ù•ß#•×ï¾2j^□Õ.oôí.ÇM’Êcq□Ê□BPîÀ,H;sÁæ»¯ˆß
□ÆÞ□´Óà°H5
□–ÞW□c□¸*qÈ□ŽG ®:÷Â?□|K¦É£êúöŸo`P¤□□□ç□à□«’□B2□□ç4□«ð‹þH|¿îÝ□Žoìíÿ $òóþ
Â’ÿ è¸ë□á÷„µ?

ü3m□PòVõÄã□û”oÎ2qMøEá□SÁž□ŸNÕÄ"æK×œ□Ÿx
U□dúü¦€9/□(¿ð‘xéö□Âò0□□ãtÜR~ÑÆOì=□Nï²›Æ2ã×hÇé°º_…þ
Õü%ªø¢ãSò<½Féd·ò¤ÜJƒ!Éã•¾?Zé|oàû/□øfm–ñÌDº’□Õrb•g□ŽýH#Đž•h□~/
,B,□¾VÑ³oLvÇµx§‡À?µ□ˆ□–ß$Z;oLì‹wþ?×Þ□gá¯□□%’hÖ□Þ™-
”KåÃpåY£AĐe“wOcŽÕØü7øt¾
·»»¾¼û~·~Û®îŽHë□ªO''I''qé@□□,6ÿ ÃGxÃ8ÏÙ_-
÷ÜUÑ|w0□…wÞvÝæh|•ßßÞ:{íÝ\®·ð›Å÷Ÿ□5□□èú¥¾□/˜&±“yÌ‡ ¯□l□”c=A□§CSMðëâ□Žoì£ñ
Þ¯jšM£ï6ö¸Ý!õÂ€2FFIã'□“@

ñØ□~ÍZ(“;¾ÍeŒúm□ý1^§à¯ù□ü;ÿ `Ëoý□µ‹ñ;Âw¾'ð

š&□□+2É□Ž7mŠ□OAéÅtž□° ›JðÆ"§\□3ÚYÃ□…

Få@§-Ù□□§E□P□E□P□E□P□E□P□E□P□E□P□E□P□E□P□E□P□E□P□E□P□E□P□E□P□E□P□E□P□E□P□E□
P□E□P□E□P□E□P□E□P□E□P□E□P□E□P□E□P□E□P□E□P□E□P□E□P□E□P□E□P□E□P□E□P□E□P□E□P□E
□P□E□P□E□P□E□P□E□P□E□P□E□P□E□P□E□P□E□P□E□P□E□P□E□P□E□P□E□P□E□P□^AûF□È…¦ÿ ØZ
?ý□-zýy÷Åÿ □ê¾4ð-žŸ¤□MÄ7épÂY6□¡□N□®XP□)ñ—
G¸¶Ð|9ã]4m½ÑÚ□î□ð□□'Ø?□ÿ lÕ/□x,?Š:Ç„<)¤H~Ë|□þø¡□Ä□?)÷U

qÜ•¯d¿Ñ!Õ¼/6‰{ƒ□ö¿g□Žqòã#Ü-GÒ¼çàÿ ÂÛï□ßjZ–µöv¼‘D□þKî
-rÍÓ¹
ïÁõ

ß‹ vñZø÷á½¼□¬pÅx‰□(ÀU□Ä …uÿ □~"Kàßìý;L°□úÖ¤û-¡bvŽ@□•ÉÉ 1žyãšÿ □ü□«ø—
Æ-□Ôôñ□¶Ó.„·&I6□»Ñ¸□ç…4¿□¾-ê-,¸Òõ•
ú;MkK}Ð´¿u†àÃœ□□FG□9æ€<ïâ

ß□.<
¨ÉâK]2ÛHa□¸Š□…×÷‹ ·□'ø¶÷«Þ23•Ù—Dò³´‹q/û¹?×mK¯x□â×Œô™m5ÝcOŽÞ5Ü–
±0Q;Ž›¶®1ß"€@â½□OðZÝ|+µðŽ¶□&Ía˜Äs±Ç
©îA þ□□§à•$x□ÃÞF<¯ìÛ}¸ÿ ®kúÕ□‰ÿ òL¼Aÿ ^□üÅyæ›àï‹Þ□´:&…–é³iŠH,YB"□'9Ã)#é
ó□Ú°[_□xŠ□†zþ□¨ë?ÚšÖ¨-BòHÞZ1 m

ÜãŽ¸□¸â€/|□P¿
4, 2³□Žÿ ¾zåt!³ö£ñ□^□±\ãÞ(□ó®ûáÎ•{á• éZ6£åý®Ù_Ì□¶å□¤f□ý□V-›à½
Z×ã†¯âÉ□ÙwV‹□dIóî □à®=Pþ”□ƒ{ñ+Æ-'ñ.££ø□FµšßO□{Û¦□œ‘‘- □AÀä•3Ç riÿ
oü/Ÿ
Ÿ□ý—ûCÊ_+ìØÛåfLg□ó°°I>□øóÁþ&Õ/ü
¨Ù□□JO1à¸□(rH□□•—qÁ□>¢™£ü2ñÂüGÑ¼Sâ□V×P‘□µË,„y □µT`

sÐ □'ë@□>;ÙÞj-;ð•-
Ÿ7‟{qû¨$ÞWc™ S‵Èç□Ö‡ÀmKKÓ□Pð-Þž4ÿ □Ã+□ÌŸ~åA<gÕ□ˀ8ÇÌ3óWMãŸ□êþ ø…á-
jÇÈû-™:Ér^L0
n□9È□□Ä¿†×zõý§‰|12YøŽÑÔïÝ°L£¦O÷‡©ê8=¨□□ø²sñƒÀKu□°ùñýï»»Î□³í□¹¯o¯?ñ—€n>
ø;N‹U’□/□Z ‵f‡-
□J@Þ¾»I□§#□®9åN□ñºk#¤>½¦¬□|³z□o+Œ}à›³ŽøÏ½ Eðr4‟â7Ä□l□´ãtÊ□ýÒ

²lü6î§Ià?□ü8°»¼ð□ôWúLŽf}.äe¿ ~ñÆ9R□à

□íü%ðæÓÂž ¼Đm¯$ûUôn./Đm}ì¥C/ \ð3×žõÅÃá¯□□E¹Ò,<Aasd2‛]ÌA‛□·,¥³ÿ }c±

ëáÇŽâñ÷‡Zÿ ìße»,_&â□Û€l□
ŸB□~˜#ž§°®7á·•WÀ^□k□¹□W—□™®fQ…-
€□¯| ;õÉ<t□• □QE □QE □QE □QE □QE □QE □QE □QE □QE □QE □QE □QE □QE □QE □Q
E □QE □QE □QE □QE □QE □QE □QE □QE □QE □QE □QE □QE □QE □QE □QE □QE □QE □
QE □QE □QE □QE □QE □QE □QE □QE □QE □QE □QE □QE □QE □QE □QE □QE □QE □QE
□QE □QE □QE □QE □QE □QE □QE □QE □QE □QE □QE □QE □QE □QE □QE □QE □QE □QE
 □QE □QE □QE □T-wl¬U®"□□□\qIöË_ùù‡þû□□=□□Û-□çæûìQöË_ùù‡þû□□=□□Û-□çæ-
ûìQöË_ùù‡þû□□=□□Û-□çæûìQöË_ùù‡þû□□=□□Û-□çæûìQöË_ùù‡þû□□=□□Û-□çæ-
ûìQöË_ùù‡þû□□=□□Û-□çæûìQöË_ùù‡þû□□=□□Û-□çæûìQöË_ùù‡þû□□=□□Û-□çæ-
ûìQöË_ùù‡þû□□=□□Û-□çæûìQöË_ùù‡þû□□=□□Û-□çæûìQöË_ùù‡þû□□=□□Û-□çæ-
ûìQöË_ùù‡þû□□=□□Û-□çæûìQöË_ùù‡þû□□=□□Û-□çæûìQöË_ùù‡þû□□=□□Û-□çæ-
ûìQöË_ùù‡þû□□=□□Û-□çæûìQöË_ùù‡þû□□=□□Û-□çæûìQöË_ùù‡þû□□=□□Û-□çæ-
ûìQöË_ùù‡þû□□=□□Û-□çæûìQöË_ùù‡þû□□=□□Û-□çæûìQöË_ùù‡þû□□=□□Û-□çæ-
ûìQöË_ùù‡þû□□=□□Û-□çæûìQöË_ùù‡þû□□=□□Û-□çæûìQöË_ùù‡þû□□=□□Û-□çæ-
ûìQöË_ùù‡þû□□=□□Û-□çæûìQöË_ùù‡þû□□=□□Û-□çæûìQöË_ùù‡þû□□=□□Û-□çæ-
ûìQöË_ùù‡þû□□=□□Û-□çæûìQöË_ùù‡þû□□=□□Û-□çæûìQöË_ùù‡þû□□=□□Û-□çæ-
ûìQöË_ùù‡þû□□=□□Û-□çæûìQöË_ùù‡þû□□=□□Û-□çæûìQöË_ùù‡þû□□=□□Û-□çæ-
ûìQöË_ùù‡þû□□=□□Û-□çæûìQöË_ùù‡þû□□=□□Û-□çæûìQöË_ùù‡þû□□=□□Û-□çæ-
ûìQöË_ùù‡þû□□=□□Û-□çæûìQöË_ùù‡þû□□=□□Û-□çæûìQöË_ùù‡þû□□=□□Û-□çæ-
ûìQöË_ùù‡þû□□=□□Û-□çæûìQöË_ùù‡þû□□=□□Û-□çæûìQöË_ùù‡þû□□=□□Û-□çæ-
ûìQöË_ùù‡þû□□=□□Û-□çæûìQöË_ùù‡þû□□=□□Û-□çæûìQöË_ùù‡þû□□=□□Û-□çæ-
ûìQöË_ùù‡þû□□=□□Û-□çæûìQöË_ùù‡þû□□=□□Û-□çæûìQöË_ùù‡þû□□=□□Û-□çæ-
ûìQöË_ùù‡þû□□=□□Û-□çæûìQöË_ùù‡þû□□=□□Û-□çæûìQöË_ùù‡þû□□=□□Û-□çæ-
ûìQöË_ùù‡þû□□=□□Û-□çæ-
ûìQöË_ùù‡þû□□=□□\A+mŽhÝ½□•5% □QE □QE □QE □QE □S^D‰wHê‹Ó,p*/¶Zÿ ÏÌ?÷Ø
è¨>Ùkÿ ?0ÿ ßb•¶Zÿ ÏÌ?÷Ø è¨>Ùkÿ ?0ÿ ßb•¶Zÿ ÏÌ?÷Ø
è¨>Ùkÿ ?0ÿ ßb•¶Zÿ ÏÌ?÷Ø è¨>Ùkÿ ?0ÿ ßb•¶Zÿ ÏÌ?÷Ø
è¨>Ùkÿ ?0ÿ ßb•¶Zÿ ÏÌ?÷Ø è¨>Ùkÿ ?0ÿ ßb•¶Zÿ ÏÌ?÷Ø
è¨>Ùkÿ ?0ÿ ßb•¶Zÿ ÏÌ?÷Ø è¨>Ùkÿ ?0ÿ ßb•¶Zÿ ÏÌ?÷Ø
è¨>Ùkÿ ?0ÿ ßb•¶Zÿ ÏÌ?÷Ø è¨>Ùkÿ ?0ÿ ßb•¶Zÿ ÏÌ?÷Ø
è¨>Ùkÿ ?0ÿ ßb•¶Zÿ ÏÌ?÷Ø è¨>Ùkÿ ?0ÿ ßb•¶Zÿ ÏÌ?÷Ø
è¨>Ùkÿ ?0ÿ ßb•¶Zÿ ÏÌ?÷Ø è¨>Ùkÿ ?0ÿ ßb•¶Zÿ ÏÌ?÷Ø
è¨>Ùkÿ ?0ÿ ßb•¶Zÿ ÏÌ?÷Ø è¨>Ùkÿ ?0ÿ ßb•¶Zÿ ÏÌ?÷Ø
è¨>Ùkÿ ?0ÿ ßb•¶Zÿ ÏÌ?÷Ø è¨>Ùkÿ ?0ÿ ßb•¶Zÿ ÏÌ?÷Ø
è¨>Ùkÿ ?0ÿ ßb•¶Zÿ ÏÌ?÷Ø è¨>Ùkÿ ?0ÿ ßb•¶Zÿ ÏÌ?÷Ø
è¨>Ùkÿ ?0ÿ ßb•¶Zÿ ÏÌ?÷Ø è¨>Ùkÿ ?0ÿ ßb•¶Zÿ ÏÌ?÷Ø
è¨>Ùkÿ ?0ÿ ßb•¶Zÿ ÏÌ?÷Ø è¨>Ùkÿ ?0ÿ ßb•¶Zÿ ÏÌ?÷Ø
è¨>Ùkÿ ?0ÿ ßb•¶Zÿ ÏÌ?÷Ø è¨>Ùkÿ ?0ÿ ßb•¶Zÿ ÏÌ?÷Ø
è¨>Ùkÿ ?0ÿ ßb•¶Zÿ ÏÌ?÷Ø è¨>Ùkÿ ?0ÿ ßb•¶Zÿ ÏÌ?÷Ø
è¨>Ùkÿ ?0ÿ ßb•¶Zÿ ÏÌ?÷Ø è¨>Ùkÿ ?0ÿ ßb•¶Zÿ ÏÌ?÷Ø
è¨>Ùkÿ ?0ÿ ßb•¶Zÿ ÏÌ?÷Ø è¨>Ùkÿ ?0ÿ ßb•¶Zÿ ÏÌ?÷Ø
è¨>Ùkÿ ?0ÿ ßb•¶Zÿ ÏÌ?÷Ø è¨>Ùkÿ ?0ÿ ßb•¶Zÿ ÏÌ?÷Ø
è¨>Ùkÿ ?0ÿ ßb•¶Zÿ ÏÌ?÷Ø è¨>Ùkÿ ?0ÿ ßb•¶Zÿ ÏÌ?÷Ø
è¨>Ùkÿ ?0ÿ ßb•¶Zÿ ÏÌ?÷Ø è¨>Ùkÿ ?0ÿ ßb•¶Zÿ ÏÌ?÷Ø
è¨>Ùkÿ ?0ÿ ßb•¶Zÿ ÏÌ?÷Ø è¨>Ùkÿ ?0ÿ ßb•¶Zÿ ÏÌ?÷Ø
è¨>Ùkÿ ?0ÿ ßb•¶Zÿ ÏÌ?÷Ø è¨>Ùkÿ ?0ÿ ßb•¶Zÿ ÏÌ?÷Ø
è¨>Ùkÿ ?0ÿ ßb•¶Zÿ ÏÌ?÷Ø è¨>Ùkÿ ?0ÿ ßb•¶Zÿ ÏÌ?÷Ø
è¨>Ùkÿ ?0ÿ ßb•¶Zÿ ÏÌ?÷Ø è¨>Ùkÿ ?0ÿ ßb•¶Zÿ ÏÌ?÷Ø
è¨>Ùkÿ ?0ÿ ßb•¶Zÿ ÏÌ?÷Ø è¨>Ùkÿ ?0ÿ ßb•¶Zÿ ÏÌ?÷Ø
è¨>Ùkÿ ?0ÿ ßb•¶Zÿ ÏÌ?÷Ø è¨>Ùkÿ ?0ÿ ßb•¶Zÿ ÏÌ?÷Ø
è¨>Ùkÿ ?0ÿ ßb•¶Zÿ ÏÌ?÷Ø è¨>Ùkÿ ?0ÿ ßb•¶Zÿ ÏÌ?÷Ø
è¨>Ùkÿ ?0ÿ ßb•¶Zÿ ÏÌ?÷Ø è¨>Ùkÿ ?0ÿ ßb¦VWPÊÁ”ô ä□ Z(¢€
(¢€
(¢€
(¢€
(¢€
(¢€
(¢€
(¢€

(¢€
(¢€
(¢€
(¢€
(¢€
(¢€
(¢€
(¢€
(¢€
(¢€
(¢€
(¢€
(¢€
(¢€
(¢€
(¢€
(¢€
(¢€
(¢€
(¢€
(¢€
(¢€
(¢€
(¢€
(¢€
(¢€
(¢€
(¢€
(¢€
(¢€
(¢€
(¢€
(¢€
(¢€
(¢€
(¢€
(¢€
(¢€
(¢€
(¢€
(¢€
(¢€<_^µ¸o□j,¶ò•nd
„<üÆ³þÇuÿ >Ó□ß□½âŠ ð□±Ý□Ï´ß÷Á£ìw_óí7ýðkÞ(□□û□×üûMÿ |□>Çuÿ >Ó□ß□½âŠ ð□±
Ý□Ï´ß÷Á£ìw_óí7ýðkÞ(□□û□×üûMÿ |□>Çuÿ >Ó□ß□½âŠ ð□±Ý□Ï´ß÷Á£ìw_óí7ýðkÞ(□□
û□×üûMÿ |□>Çuÿ >Ó□ß□½âŠ ð□±Ý□Ï´ß÷Á£ìw_óí7ýðkÞ(□□û□×üûMÿ |□>Çuÿ >Ó□ß□½â
Š ð□±Ý□Ï´ß÷Á£ìw_óí7ýðkÞ(□□û□×üûMÿ |□>Çuÿ >Ó□ß□½âŠ ð□±Ý□Ï´ß÷Á£ìw_óí7ýðk
Þ(□□û□×üûMÿ |□>Çuÿ >Ó□ß□½âŠ ð□±Ý□Ï´ß÷Á£ìw_óí7ýðkÞ(□□û□×üûMÿ |□>Çuÿ >Ó
□ß□½âŠ ð□±Ý□Ï´ß÷Á£ìw_óí7ýðkÞ(□□û□×üûMÿ |□>Çuÿ >Ó□ß□½âŠ ð□±Ý□Ï´ß÷Á£ìw_ó
í7ýðkÞ(□□û□×üûMÿ |□>Çuÿ >Ó□ß□½âŠ ð□±Ý□Ï´ß÷Á£ìw_óí7ýðkÞ(□□û□×üûMÿ |□>Ç
uÿ >Ó□ß□½âŠ ð□±Ý□Ï´ß÷Á£ìw_óí7ýðkÞ(□□û□×üûMÿ |□>Çuÿ >Ó□ß□½âŠ ð□±Ý□Ï´ß÷Á
£ìw_óí7ýðkÞ(□□û□×üûMÿ |□>Çuÿ >Ó□ß□½âŠ ð□±Ý□Ï´ß÷Á£ìw_óí7ýðkÞ(□□û□×üûMÿ
 |□>Çuÿ >Ó□ß□½âŠ ð□±Ý□Ï´ß÷Á£ìw_óí7ýðkÞ(□□û□×üûMÿ |□>Çuÿ >Ó□ß□½âŠ ð□±Ý□
Ï´ß÷Á£ìw_óí7ýðkÞ(□□û□×üûMÿ |□>Çuÿ >Ó□ß□½âŠ ð□±Ý□Ï´ß÷Á£ìw_óí7ýðkÞ(□□û□
×üûMÿ |□>Çuÿ >Ó□ß□½âŠ ð□±Ý□Ï´ß÷Á£ìw_óí7ýðkÞ(□□û□×üûMÿ |□>Çuÿ >Ó□ß□½âŠ
ð□±Ý□Ï´ß÷Á£ìw_óí7ýðkÞ(□□û□×üûMÿ |□>Çuÿ >Ó□ß□½âŠ ð□±Ý□Ï´ß÷Á£ìw_óí7ýðkÞ(
 □□û□×üûMÿ |□>Çuÿ >Ó□ß□½âŠ ð□±Ý□Ï´ß÷Á£ìw_óí7ýðkÞ(□□û□×üûMÿ |□>Çuÿ >Ó□ß
□½âŠ ð□±Ý□Ï´ß÷Á£ìw_óí7ýðkÞ(□□û□×üûMÿ |□>Çuÿ >Ó□ß□½âŠ ð□±Ý□Ï´ß÷Á£ìw_óí7
ýðkÞ(□□û□×üûMÿ |□>Çuÿ >Ó□ß□½âŠ ð□±Ý□Ï´ß÷Á£ìw_óí7ýðkÞ(□□û□×üûMÿ |□>Çuÿ

>Ó□ß□½âŠ ð□±Ý□Ï´ß÷Á£ìw_óí7ýðkÞ(□□û□×üûMÿ |□>Çuÿ >Ó□ß□½âŠ ð□±Ý□Ï´ß÷Á£ì w_óí7ýðkÞ(□□û□×üûMÿ |□>Çuÿ >Ó□ß□½âŠ ð□±Ý□Ï´ß÷Á£ìw_óí7ýðkÞ(□/ð□¼ÑxŒ´•È ‹ä7,¤

¢½BŠ(¢Š(¢Š(¢Š(¢Š(□–ñüo/‡□ÆŒíç¯
2z□ó□±Ý□Ï´ß÷Á¯x¢€<ìw_óí7ýðhû□×üûMÿ |□÷Š(□ÁþÇuÿ >Ó□ß□•±Ý□Ï´ß÷Á¯x¢€<-
ìw_óí7ýðhû□×üûMÿ |□÷Š(□ÁþÇuÿ >Ó□ß□•±Ý□Ï´ß÷Á¯x¢€<-
ìw_óí7ýðhû□×üûMÿ |□÷Š(□ÁþÇuÿ >Ó□ß□•±Ý□Ï´ß÷Á¯x¢€<-
ìw_óí7ýðhû□×üûMÿ |□÷Š(□ÁþÇuÿ >Ó□ß□•±Ý□Ï´ß÷Á¯x¢€<-
ìw_óí7ýðhû□×üûMÿ |□÷Š(□ÁþÇuÿ >Ó□ß□•±Ý□Ï´ß÷Á¯x¢€<-
ìw_óí7ýðhû□×üûMÿ |□÷Š(□ÁþÇuÿ >Ó□ß□•±Ý□Ï´ß÷Á¯x¢€<-
ìw_óí7ýðhû□×üûMÿ |□÷Š(□ÁþÇuÿ >Ó□ß□•±Ý□Ï´ß÷Á¯x¢€<-
ìw_óí7ýðhû□×üûMÿ |□÷Š(□ÁþÇuÿ >Ó□ß□•±Ý□Ï´ß÷Á¯x¢€<-
ìw_óí7ýðhû□×üûMÿ |□÷Š(□ÁþÇuÿ >Ó□ß□•±Ý□Ï´ß÷Á¯x¢€<-
ìw_óí7ýðhû□×üûMÿ |□÷Š(□ÁþÇuÿ >Ó□ß□•±Ý□Ï´ß÷Á¯x¢€<-
ìw_óí7ýðhû□×üûMÿ |□÷Š(□ÁþÇuÿ >Ó□ß□•±Ý□Ï´ß÷Á¯x¢€<-
ìw_óí7ýðhû□×üûMÿ |□÷Š(□ÁþÇuÿ >Ó□ß□•±Ý□Ï´ß÷Á¯x¢€<-
ìw_óí7ýðhû□×üûMÿ |□÷Š(□ÁþÇuÿ >Ó□ß□•±Ý□Ï´ß÷Á¯x¢€<-
ìw_óí7ýðhû□×üûMÿ |□÷Š(□ÁþÇuÿ >Ó□ß□•±Ý□Ï´ß÷Á¯x¢€<-
ìw_óí7ýðhû□×üûMÿ |□÷Š(□ÁþÇuÿ >Ó□ß□•±Ý□Ï´ß÷Á¯x¢€<-
ìw_óí7ýðhû□×üûMÿ |□÷Š(□ÁþÇuÿ >Ó□ß□•±Ý□Ï´ß÷Á¯x¢€<-
ìw_óí7ýðhû□×üûMÿ |□÷Š(□ÁþÇuÿ >Ó□ß□•±Ý□Ï´ß÷Á¯x¢€<-
ìw_óí7ýðhû□×üûMÿ |□÷Š(□ÁþÇuÿ >Ó□ß□•±Ý□Ï´ß÷Á¯x¢€<-
ìw_óí7ýðhû□×üûMÿ |□÷Š(□ÁþÇuÿ >Ó□ß□•±Ý□Ï´ß÷Á¯x¢€<-
ìw_óí7ýðhû□×üûMÿ |□÷Š(□ÁþÇuÿ >Ó□ß□•±Ý□Ï´ß÷Á¯x¢€<-
ìw_óí7ýðhû□×üûMÿ |□÷Š(□ÁþÇuÿ >Ó□ß□•±Ý□Ï´ß÷Á¯x¢€<-
ìw_óí7ýðhû□×üûMÿ |□÷Š(□ÁþÇuÿ >Ó□ß□•±Ý□Ï´ß÷Á¯x¢€<-
ìw_óí7ýðhû□×üûMÿ |□÷Š(□ÁþÇuÿ >Ó□ß□•±Ý□Ï´ß÷Á¯x¢€<-
ìw_óí7ýðhû□×üûMÿ |□÷Š(□ÁþÇuÿ >Ó□ß□•±Ý□Ï´ß÷Á¯x¢€<-
ìw_óí7ýðhû□×üûMÿ |□÷Š(□ÁþÇuÿ >Ó□ß□½•ÃJÉá½=X□a
ä□,+VŠ (¢Š (¢Š (¢Š (¢Š (¢Š (¢Š (¢Š (¢Š (¢Š (¢Š (¢Š (¢Š (¢Š (¢Š (¢Š (¢Š
(¢Š (¢Š (¢Š (¢Š (¢Š (¢Š (¢Š (¢Š (¢Š (¢Š (¢Š (¢Š (¢Š (¢Š (¢Š (¢Š (¢Š (¢Š
(¢Š (¢Š (¢Š (¢Š (¢Š (¢Š (¢Š (¢Š (¢Š (¢Š (¢Š (¢Š (¢Š (¢Š (¢Š (¢Š (¢Š (¢
Š (¢Š (¢Š (¯3ñ□Ä}^□□□□ø3IMOZTßpòŸÝ@0

□ g□rH□ rN□ü3□ñV?□ZÛø"JÓ%Ò¦Ýç\[°†‡å$□□<àt?Z ôš(®âG□'ðm®□m¦Ø‹ícT˜Ãg
çfF□N9<²€23ž¼P□qExž©ã‹~□²:Þ½£iSéhËçÇ□□Ñ□@ÆUŽ

H□ù…zþ•ªA-è¶Z¥°a□Ü   2□ê□

àûŽ”□vŠŽiá·□ÌžT‰2□ç`£?SMk»d¸[w¸‰ga•Œ¸

~ƒ- MEE=Õ½¨Sq<P†8S#…Éô□©zŒŠ (¨®.-í□=ÌñB„ãtŽ□~´ô‘%@ñ°°0Èe9□€□ED.-É”
â&õ£xù>¾Ÿ□,□□]Gæ[Í□±ž7FÁ‡æ(□J+ƒ¿ø‘□—Äý?Â€Z5¥Í«K%á¸□Êp□í#§ð

ÿ ÅR|TÔ
ü(Öï4û²□”›&·"þš(8`~¢€;Š+—ðF¡□ð□‡d½¼Q4Ö1|ÓËó9Ú;"É®¢€
*□¶Úý§ìßi‡í-
óËÌ□¿.µ= □QE □QE □QE □QE □QE □QE □QT5½bÓ@Ñ/5kæ+mi□‘ñÔã -äà□s@□è¯□²ñ—
ÅŸ□Ù□kÃú□›o¥1&Þ9˜□•AÇVaž□p¢»O†Þ>>7Ó.-òÓìZ¾Ÿ
ŠòÜ□ '8 -@ÈaƒÈ"€;z(®□ÆŸ□ÿ áÔ¢Ð4m.}[ÄW1‡†Ö5;□IÀgoN   Àì9#- w4WŸü!ñŽ«ão
]ê:¿‛çÅ|ð¯’›FÐˆÃ¹îÆ´ümãÛO□Çm ³¹Ô5[Ü‹;□e%¥#Ôààr;□è:Ð□[EyŸÂÏ□øƒÅš·‰lõû{ky
tÙ£□a…1å±2□Rrs,ƒõ¯L □ŠŽIá…‛e•□ÈÛP3□¸ú□SM[»f¹ku¸ˆÎ£& ãpN´□5□

·VðÊ‘Kq□rI÷□Ü□ßAÞ¦ □Š,âöÖÔ¨¸¹†□ßtI \ý3S□□□□AèE -□□Ûm<—
›íPùHJ»ùƒj‘Ô□ØÔ‘Ë□Ñ¬‘:°0Èe9□ñ □Ñ\□ÿ Äˆ,¾'éþ□□Ñ-.mZY/□À-S€çi□?€wþ*ÇøçªI□
Ã›{□2ùÐ>¡□ù¶Ó□¸aò2§Ô~”□ê”U;kØ□□X¥¹ŒO$jB;□ÍÇ§SW(¢ ûm¯Ú~Íö˜~Ñÿ <¼Á»òëSÐ□
E□P□E□P□E□P□E□P□E□P□E□•â?□iþ□Ð®5}MÝm`□!□s1'□@õ&€5h¯□Ð¾)x—Zø¡£é□:BizF£

“C□èLÒF□B®Ol”éŽž½kÓ<Q-Káï□Þê°ióê□@£e¬ –
‘‹□□€xç'Ž€Đ□½□â·ž)øÉe§I¯Üx□KŠÂ$2Éh~úF□I#~î□^þÕéž

ñE¿Œ|+g-ÛÄa□,-□rcppÃ=ù□B(□zŠ+Ì¼sñN}-ïPÑ¼3¥K©j¶0´×r²□□¢□ÜK-ç-
AÈäž(□Óh®Sá¯ˆo¼Sà□7XÔ¼³wqæ

KµNÙ□G@+šñwÄ□□7•_ÁÞ
Òíîµ("□ÝOr~XÁ ñÈ□□^I<œc4□ê□W□Y|@ñ¿†<W¥hþ:Òì¾ËªKäÁwhFCd

ðH □□□□œóÐû□ □Tm<)2BÒ •Á*…†æ□®□zlWvÓÈñÃq□•□Ãª8%~ t
¨¨MÕ¸¹□Æâ!9□□□□ˆúu©¨ ¢ –úÒ –□n ŽVû¨ò Çè*bB,I

I4 ´Uv¿³Ku¸k¸

p²™□Óô9ÅM½6oÜ6c;³Æ=h□ÔW□¦üJ¶¾ø‷«øbO±Åeel³E{ö‛‰IÙ•ôþ3Ðÿ □b|[Ô®`ñ□€□•ì±Ã>
¥-òe!d□£Æpy□ŸÎ€=bŠ„]Û□“l."3□“□ñ»-
Nµ18□4 QPAyktX[ÜÃ1N□ËpØúâ§ □Š(□Š(□Š(□Š(□Š(□Š(□Š(□Š(□Š(□Š(□Š(
□ŠÊñ'ˆl|-
áû½gQf□öÉ’ª2ÎIÀQîI□¼°ÏÅ_□¼Ql°¶‹ é¶:l£}°\˚ß"vå˜□Ÿ\(#¥ {5□□á[½r÷ÃÖÓxŽÂ+□T
-□A□□Q† □,zŒ-¦¶IÀÉ □Šñx> |AñÍÝìÞ□ÒlcÑí¦1-ÍÙ□¤aÏsÔ,

ã<šÞð□Ä□oVñ6¡á?□é‘YkVqyÁ ?#¯ËÁäó†□□pFzc□□J¢Š‡íVù”}¢,Ãþ·ç'ûÞŸ• MEG

ðÜÄ%,XåŒôxØ0?ˆ¦ÃuorÎ°\E)C‡□á¶Ÿ|t
¨¢ ŽúÒiÚ□® ’UûÈ²□Ãê(□z*9§†Þ?2yR$È□□,ŒýM5îí£□ {ˆ-
gû±³€Íô□M MECuu□•»Oq4qF½ZG
?3_ÃŸˆ°øÛB’öñm,.Ví-ÖÜ\□X ¤□

□;±øP□uEyN•□√ß´†±f×s›TÓ□¬&C±~XÏ
Ó©?□z„□V÷A□½ÄS□8o-
Ã`ûâ€&¢šî±¡w`ª£%˜à□L·°·»MöÓÅ2□□Ñ¸aŸÂ€%¢Š(¢Š(¢Š(¢Š(¢Š(¢Š(¢Š(¢¼ÛÇ□
□5]3Ä¶¾□ðž™□£¯\'˜þiù!^¸##œ

ä• Ç\ñ,~!øïÁ:½Šx÷K³:Eäž_Û-9ò□àHã®□ÉÁÁâ€=žŠ□□d□ƒUu-
F×HÓ.u□Ù|«[hÚY_□á@Éàrh□Ȭ□â7?□õûÿ □øu4Ý□ì:□§|-
ñOy-d°]è□¨Î□ ã¦yîy□Û$uŠ6‘Ø*(,Äö□€□ExWŒ>7êâÐ_xSJ+£¥È·:•ìGlÒ□NÔ\Ž0¤úôÈ^þè
§r)õ□ □¢Š€ßZ□-cu□”ŒUßÌ□Tú□ØÐ□ôS□XÞ!*H•□□

A-¹¦Ás□Òo·ž9•□n•Ã

þ□□-□□ $œ□Ôš,
Û[¢ÂÞæ□Šýá□•±õÅ OEG$ðÂÈ²Ê^dm¨□€Ü}□©¦‹»crm…ÄFp2bÞ7céÖ€&¢²<M®Eáß

ê□›˜‹ÛÛÉ,QI O5•I
□¾;Vo•¼goâï
éÚ”-koytu¢L□”«2ð:ô\þ4□ÔÑ^OðßQ¹›â_Ä$º¼™à,ì□Ye%c□Üp À□□ê•\Au™o4rÆxÝ□□˜
(¦M4Vñ□f•#•z³° ~&ˆfŠâ!¯,2¤±žŒŒ□-
ÄP□è¢Š (¢Š (¢Š (¢Š (¢Š (¢Š (¯2ñÏÅ9ô{½CFðÎ•.¥ªØÂÓ]ÊÈD□ˆ□q,{œ}□#’x®‹á¯ˆo¼
Sà□7XÔ¼³wqæ

KµNÙ□G-
@(□«¢¼ßÅ~)ñÔ¾(Ÿ@ðw‡QÖÙ□¦Ôo□";”7ÈI□Œã¹Îxã5GÂß□¼Qiã^<!ã□2ÚÞòê2ö·6Çå~§žH í
□Œ□,= =ZŠ++Ä~"Óü+¡\jú›°ÚÀ□B.æbN□•êM jÑ^9¡|Rñ.µñCGÒ.t„Òô□F□&†)Ð™¤Œ,…\žÙ)
Ó□=z×¥x¯ÄVþ□ðÅþ¹u□I□ª□#S,ìHU\öÉ#žÔ□³Exµ¯‰¾2k:tzæ•¢i)a*y°[±□äŒò□/žG¸ÏaÈ®
çá¿Ž□•<1ý£%¨µ»†S□ÄJr»À□+ß□-ý9□õ
□…□„…□’ □’j□¿²Kt¸k¸□.p²□□Öú□àÐ□Š)□,qÄey□c□%Ù€ zæˆfŠâ!,2$‘·FF□-
ÄP□è¦»¤h^FTE□,Ç Tv÷V÷h^Úx¦PpZ7

?J šŠ□§…&HZT□¸%P°ÜÀuÀïMŠîÚi^(®"’Hþú#,Wê;P□ÔW/ãÿ □¯,ü)wª¢A=Ô[<«i%ÚdËªŸs€
sÇ¥hø{Ä□šþ•ay□öþ}Í¬w□□s□1îPHõàœP□½□äß□uYçð¾·q©_É
MMÿ ys1;FÕîÇŠõhåŽhÖH□]□d2œƒøÐ□è¨®.-í#ó.gŠ□Î7HáF~¦žŽ’ t`ÈÃ!”ä□@

¢Š(¢Š(¢Š(¢Š(¢Š(¢Š(¢Š(¢Š(¢Š(¢Š(¢Š(¢Š(¢Š(¢Š(¢Š(¢Š(¢Š(¢Š(
 ¢Š(¢Š(¢Š(¢Š(¢Š(¢Š(¢Š(¢Š(¢Š(¢Š(¢Š(¢Š(¢Š(¢Š(¢Š(¢Š(¢Š(¢Š
(¢Š(¢Š(¢Š(¢Š(¢Š(¢Š(¢Š(¢Š(¢Š(¢Š(¢Š(¢Š(¢Š(¢Š(¢Š(¢Š(¢Š(¢
Š(¢Š(¢Š(¢Š(□æŸ□ê>9ŸÅ¾/¹ð–•gs5Õó□›»²□’7¹
¹a×=0z
î4□ˆ^1Ð|e¦øwÇš]¬i©°ŽÖî×¡rp:□□É Ž□ÎzV

•¯¯Áï‰–#¶×,®□±õ‰¾Óks□n□æb□8Î□□{ä

9©n5—øÁñ+Ã•¢X\Ç¢èS}¢{ÉãÚ　　　Ü¬W¯□Ø

¼�harmonic

ìu•þ
Ó5+«
‹ ›Ï>ÚV†M¶ÌFå88?QX□□¯<=â□hþ,ƒYk
»w7□L…

ù_#)·¨9Uù»□=kÕdÑô¹¤i%Ól ÞF9fh□’}IÅy□Å^gð÷□|/â©´·½ðöŸ□ŽH"L¬.□Žìt□□Hé’˜â€8
ÍwÇ-,ñ-Ÿ¥hÞ6Š]□A»e77É`á®□□ƒƒÇP□Ê=ðF□}#£Aam¡ØA¥²6Ÿ□°-
³#n□□Q´ƒßŒsÞ¼wÆ_□<+â_ _h°~•□□{□□†□$¶ $‡î±äò□#□ä□-z-
Ã□□ÿ @øs£é°š”»Ž6gBrSs³□>à0-Ô□Ê~Ð Ÿ‡□□Ôê1□é/\æ¯ðVÒÓáõö½{«ß\øŠ□V¾’äË”,«¼®
□Éàc$ç8<t®“öƒÿ ’uoÿ a(□ð□®ÃÅ¿òKµÏû□Ü□ê-
□*ð?ÃEø□á¨|OâýoQ¼žé□+UIqä¢1L’AÉÊ“úœ’q·ð{]°Ò¼+â};S□îbðäòlr□åš«□£Ûä$}k¢ø
)ÿ $‹BúOÿ £ä®[á],j—_□´ù Xî¯^□#°o5Oó

¿□ø ~*ÚÜøÃÆ7×‟µÌÎ–ÖðÉ±#□Oã□œ€□:dç5kD±¸øWñ‡Oðå□ýÅÆ□-ÄYb□²c‟□:`nÜ£œ

ÚØâ

̄ì®|!ã
Ëy-'w,h¢Ᵽ¬Œ□

Œä†□□=±Î†ƒwsñOâå□Š-´û‹o□è°”Š[…Á-
NqŽÙË□@Î□Œà‘@□î‘àfñ×ÅŸ□Z]jW□ÚL□ï%Ô0>□vÞÛ□§-
7'8í×#nÇAO†?□4]7E°¹:N³□,¶òÉ»æ□±ù□¤-¼‘Þµþ□ÉTø‹ÿ _¿û<”¾<ÿ ’éàO÷_ùš ãµ□†~
□‹ã-‘á¨á¸]2öÍîeO8îß‰O□Ô□‘k¾ñï†´ß
ü□Öô□*7KTUp$rÄ“2□É¬?ˆ°šxGãw†üS¨Á9Ò–ÅàibMß?ïA-
†õ?C[>8ñ=□Œ>□kz¾□□Â[H□(¸@¬vÌ œ□xâ€9¯□ü□ÓüEàû=WÄ□-ô÷—vÊmü©p¶Ñãä□
ç□· g□Ö¬|<ÕüC7Áï□éÖ³M>©¤´ÖöŒ gU
Â¯rGÍ·ð□ ¯Køyÿ $ëÃßõá□þ,+Ëþ□ø,/
øWÆúÔö³ÜÅkª’ñ@2ä□
ž}3“ì(□□À¾□ø{ã□□Þ9õëëo□9ÌË$áÌÉûªÃ

;ðIúWÒQ'•□G¹›j…ÜÇ$ã¹¯›~!x¿Àž6Òÿ âE¡ÞŸ□O"y□Gj□÷□□ß´üäŒ□Ôç□ô□‡¢¾ƒÃZT:›□Ô□
Ò%¹fl‟(A»'¿9æ€4¨¢Š (¢Š (¢Š (¢Š (¢Š +‟ø™¢]ø‹áÖ³¥Ø.û©bW•□W(êûG¹Û•Æ°Ê¡
-ê©¡èw°¤•M:ZBÒ´PŒ» 2q@-=àÿ •žÐ¼%c£ëVz•¶£§Ä-¤‰
Ý¸§□dŒ-9□□æ³~□ø®Æ_‰-6ñ□Å=®’öo{":å†□NH□ÎXþ5Ð^|tðLÐ
˜4{ÛÍK□Š□-□>îÀ¾N□}3ô-o…-□ÔíáÖ|Kâ;q□§¯Íæ½³¯ú¨¹!H=3¸ü§
î(□mþ;x-ææ(□æð4®□□jØÉ8¯G□B.□àE□™”#H□n*9 ž¸äþuU4]*7WM2Í]NC□□□}zW7â¿‰þ□ðf
¨šv¯=ÂÜ¼B`±@\m$□Ïàh□’ýœÿ äBÔ□ì)'þ‹ Ž½m …çIÚ(ÌÈ
¤…Få□□ öÎ□å_;|□ø‟áß
xv}#Všxînu□"l„°íeE□‘î□{/‹þ h>□6‹-M25Öã□Š"ù
Œç□:Š â>□ÿ ÉEø—ÿ aOý«=zý|Ýðÿ âw†ü;ã□□jZ„·+m«_yö¥!,Jù’·#·

+è»K¨o¬à»·mÐÏ□Ë□c□V□□□ØÐ□•ü~·°»¾ðmµ”æ
¹odHe

Wc"□VÈä`ó'X¾;øKcà□□7‰tÍVüë:|'I-
ËI□1™Â' r§s□9=óžµÓ|gÿ 'Ÿáÿ ý…□þ…□oülÿ 'C®ÿ Û□ý□ q□□¡ñ?,ßÄ°ö³}w¯êVŸlŽa
-äÜŠF9à€z□Ðc□-o□xêöÓà-
Þ¹w!¸¼ÒÄ¶ñÉ)Ü]•□=Þ oQô□Üø[þIn‹ÿ `X?ôB×˜ü6Ð$ñ?À=sGˆ•5ÍÔÞVN□u□Ê
ì7(□x;á□•Œ<=□‰|[¨j7zž¦

ùY¶ˆÔýÞÇ'úú

• Ž*ßÃ–¾ðWÅ□Wáü·ÒÝé~GÚlLÜ”à6□§□□õ+œ

Õ?□üaÓ¼'á¸<;â«□F×SÓ□ÀU`Îõt`•AÆ□<qœó•£ðæ□ÿ □|IÕ~!\ØËg¦´-
fÓÖa†q€7□l)Ïl¶□84□Ä|-øm□ŽcÔ®u}Fåt‹[ÇT²,B»å eŽx-
.□□ŸQŽzÿ ØÉàoŒ°·ƒ-.ç›GžÐ\Ã□Í¸«aN~¿xqÔc=*ïìûÿ "Æ·ÿ aGÿ ÐV•?äç¤ÿ °Hÿ ÐE
qÚÇÃ?□ÅñËHðÔpÜ.™{f÷2§œwoÄ§†ê□Èµ¿ñƒÃZw„þ□Úi:J<vÉª#/˜åŽY\’I§üEÔÓÂ?□¼7â•F
Î”¶/□K□nùÿ z□ü7©ú□‹âÏ‰ì|_ðzÏXÓ£¸ŽÕõTEûB□c´H
À'Š mÇÀÛ□à«½SQÕ¯®¼BmZäÜy¿&ð»,àŒ‘À□'=øé[¾□×µýoà

×–
²Í>µ□¬ðÇ(É‘Ê’□□ËmÆ;’=k¿Ô□äN»ÿ °{ÿ è³^cð»Ä□xWàKkSÚÏs□-Ä…â€eÈ2□Ï>™Éö□€9□□
ø7áïŒtkxç×¯-¼Pç3,‟„□ß'î«

0ïÁ'é_IDžTI-æmª□s□"Žæ¾mø…âÿ □xÛKÿ ‰□‡z|Q<‰äI□¨GÜXg~Óó'2□SœWÐ^-Šú□□iPêl_
PKH-
å™²L¡□ìžüçš Ò¢Š(¢Š(¢Š(¢Š(¢Š(¨æ,□˜ü¹âŽTÈm®¡†AÈ8>„□RV_ˆ¼C§ø[DŸWÕ□ÒÒ□
¡Ê!c-

□îh□Í<Iÿ '9á?û□¿ò¸¯_¯›µŸ‰Þ□½øÙ ø¦□nN™ebÐLÆ□□1□t^ÿ }kÖ®¾(èkðúïÅö+=Õ¤

aXŠ□f— □9è9□<ñêx □ø-â»o

ø*ò"Áïõ□žÒÒ ~gf□K□è çë•Þ©xF;□.Ÿ□í&ñ□xŒCÍ¹□…Š¼□Âàw□P}Á¯)ð¯□|5wâ‰<aã›ë‹-]
[□V‘[3Ch ðG¸íéÔåŽG»xcÅú□•´Û™tÍ×□ñH#•.!Ç8Èàð□úÔ□‡£|gð~»¬ZévW□□ì°•G□{v ±è
í[Þ5,□¼□â™#Š4ytË–‘•@.D,2O~ □¯

"¦ÛÊ²Á§ÚE"ý×HUHú□+Î¾$|PðÎ□§x‡ÂóÏqý¦ö2Û…X]òDv•ßð!@□-
□¿ä'h¿Yÿ ôs×□ñ>á¼+ñ.Ó]ð•ù—Ä÷ˆ±\éI ›ÍM □@é•ª6õà□V¾□üHðíŸ†4?
Ï4ëª4'F□É%
<ŒÊ7□b+>-}>□|^ñ%ÿ ŠtÛ©`Õ]žÎþ(÷•Œ¶BŒãŒ□

FÑÅ UÑ5Ùüeñ[O□¤:EÎœá´Ý- h‘å'#,Ç9%TóÃp□¡ú"¾wñÿ ‹,~-
]hú□„´Ë¹ïã¹□›é!Ø"L`ò2Bä,IÇÝ□kèp0 '>ô□àÿ □ô{Í□ä'†´«□æ²šæÄÇö…$□BÒoÆ:ü¹□ïœ
VWÄ• [ü-Ó´Ÿ□ø[S¿·¼[‘lï$□‹□F!¸ tR□èr?-ÃÅ¿òq^

ÿ ¯'ÿ Ú´~ÑŸòO´ÿ û
Eÿ ¢¥

/□ü□µÑ¼
}®É«ß\x’Ö?µÉveù□ÁË□‘Ÿ\□ç<ûVþ-ñ□Q±ø□§ø□%?Ú×±%ªLy>fJ³ýpŒ~µÛ|Dÿ ’sâúñ—
ÿ A¯4_
Ýø«öiÒm,#2^Û/Ú¡ˆur²8
{ífÀîp(□Þ□ð□E¿Ða°×oõ)õ‹¸ÄÓÎ³□±Ød□□sŒà“œõâ¨ø'TÔì¼=ãÿ □ê—
mvt[IÖÚWÎ|½Ž¸äýÞ□•Û'Ú®èß4+?□Áo-Yj1jö±□¦…!□;¨Á
’1Ó□qŽœÕo□hú□Ç†¼{ãM^Ñí%Öín□Þ□2Å±Î~‡*□LíÏB(□□áwÂk?□xN□[Ä□•ä¶Ùx¬- —
h…C□Ää--“□ÇsœñÑü)µ’×Uñ¯€/®%»Òí□£„;r#}ÊÀ□Ó ƒÇCŸZèþ□□É)Ó?ë¤ßú1«á÷ü-¿-
□¼ŸÎ€8Í□á‡†ï¾4kþ□š+“¦YY,Ð¨˜†

D][¿ßjØøÍ¡□:×Àz□ˆímåÜ=½«´‡1ŸÝ…mÝs‟œŠv©â;_‡¿uÍ_\,élu□=□ÞH£Ý¸í‹ž£ŒÆËõÅXø—
«Á¯Ýü3Õ-cš8.ïÄ±¬Ê□À-□2□#õ
>2ø7§x[À×ZõŽ©¨¾¹`□á®š\
□pÜ@□¯RG9ÏsV¾"ëZ¾¯ðgÃZ‹=ÏØîÌ'V’ØaÙvàñÓ□³×Œí¯Bø©ÿ $»Ä?õê□†®KJñ
ö—à□†>□]^ÂîâÖþÑ□¼□«ªmÇ

□Îîž€Ð□|□à□ K-XøƒÂ□õÔ³Z□Ò@n□,
•ùÐ€ËÉú□W¯×Î0^h-$ø³á«¯‡Z]Õ›Åp$Ô¤Ž/*#□W?(8Q·x< r□Zú:€
(¢€
(¢€
(¢€
(¢€
(¢€
(¢€
(¢€
(¢€
(¢€
(¢€
(¢€<kö‹ ¹•|-£YÆ¥Äú€bƒøŠ©Àÿ Ç¨—WøÕmdoÓÃú<VÐ¦EŒxf□□`-
=;□ŸjÛøÝá«ï□x!&Ó!i¯4ë•¹□ Ë2 C□-£
þ□±?á¡tGÐ□¢Ó¯›[h¶‹O(lóqýìýÜûgÚ€7tŸŒš□Þ□¶ñ.¬’Ún¹6rÅ□□6Ì□w-Åp□□sZ>□ø¯á_□k
)¤ésÜµÓ£:¬°□□□“ÏÒ³>□x6m□áøƒ\²O>òå®Í´ñ,b□B¨
ô8□õ□±]†-¡Bú□§□“mmi}=œ°Ã4qª□vB□ä□\P□,YkšÇ•<_¬i?

sâ-(–šKe¶yRÝú}åää d□7□¨□Õü□¹°×uýcÄ:ž¦×-.¸□.-¤□Ëò"□□"□□ùTg¶ ï"□ðãâF‹ðóA—
Ã~#Òï´ýJÞwi□`Ýæ"Ð·
ç□tÆ æ®ø^óþ□ïŽ0x·CÒç³Ò,–š;›‰P!□ö2Œã,ß2ŒdðۖÞ-ë_8i-□>:ø³ã+[½FâßI·¿y.a•Èi
Û{□-œ|Üœ'ÐuÈú>¼‡áwü•O^¿õûÿ ³É@□N©à[Í□â">□ðî»yc¥ë¶¢iƒ9$F7-
SŒdþí±Óƒ,pMhø¿ÁvŸ   u□□ø‡Ãw—«›Å·°ŽiCyªpO@8 0#éŒb°_□□ÉÏøSþÁ2□è74¿-
ÿ ä^Ð□i(Ÿú
□|[Ôõ=WÄz□€´«Ç³þÖ&KÉ"¯•'1×¦□É□ð□gø□àV‰¥xjëQÐoµ(5k□Zâ)š`w²□Ø8
□□ã,1ƒW>.ØjZ/Š<=ãí:ÑîãÒÿ uy□u□äœû□□Æ{qU<EñËGÖ<;s¦xvÃS¹Õïákx¡h0QœmÏ□äŒä□
œš çüeâ[¯□~Ï□F¡xáïF¢•Nÿ Þe□□ÇÜŒ□ïš×¹ø□dÞ
»Õ5-ZúïÄFÕ®šçÍù<À¥,àŒ'Ð□N{ñÒ³<má™ü%û>èÚeÚ…¼þÑŽk…-
Âî$8ü□□÷□íú□ü‰×□ö□□ý□h□Ïü□amñ;àÖ□oâVžèÁ#fQ!WfŒ°ROs´àç¯Zã¾
ü8ð÷‰<>Úæ¥

ïyk©2Ç¶b«„Ta•=É®ëàBîøUn½3q0ÿ Ç«ˆøOãÍ;À¶□^□ÖìïÓV}XªÅ□!¹p‰ÎHèWŸb1š <Mák•□
~Ðz¦‵-£-•³ÚD×o□□Ï□□2ƒ±ÉÇ^;óŒT¾#ð□·Â□□xSVðåÝâCyv-
nâš]ÛÔ•Èè2□'¯B□□ÑiŸòs°×ý,Óÿ @Š—ã—úï□□ØY□¥ Tøäìu_

Á«Ëy□…d•…ëÚç;ò1»¯AÈï÷±šÝøwàŸ□i°«xƒÂ□Ô÷P<□□ □□D□ □Ã□"ñÐúÕß□üHðÿ …uXôO□é—
W□·vÞi•@²ÄAb
°'œc'¯Q^yàFÓuo•qj^□Óîì´□í\j□"¤lÄ602p
yd/û$ã□€>□¢Š(¢Š(¢Š(¢Š(¢Š(¢Š(□Ã|S¨Íðëã„Þ,Ôln.4mRÕaó¡]Æ6□ª@Î□s□8
ÏF¬_Šÿ □¼7ã□•'K·¾–a:N³I□D□nG®s,GNõê-.ø¥áÿ kŸØ°å•öÙ
YDË x˜□F:ç·¥yö¯¬□Âà¾±ðÇ…4‰ì´□®V}Fùá□Œ

ð ã¡$□É8à M v¯ñOÃÞ□Ñô;□v{•¾—L·□„p□ûÈ□'× ×Eáo□h->°¼m,¼ðDDSÇq

Ðw

˜<□FkjM+N™c□XÛJ#@‰æD-µG@2:TWSi¾□Ò//š□í--
ãiæòb□…□' rp(□Ì>/¨_□ü4U (ÔØ □ y□W°□□ÁäWÍß□þ'xoÄ~*ðf¡§KrÖúMéžè¼%H]ñ-□~□«
Û<'ã½
Æ––w:<Ò¼vŒ□ÞlE
ä□>½□å@□í □Vß

4È`‰"‰5H,¢(UQåKÀ□¥zü□ê“ýÑ_9|aø□á¯□øR×MÑæ¸{ˆ¯Òvó!(6□‘O'Ý…{□ƒ¾#øwÆ—
3YèóNÓÛÄ$u–□Ÿ.q•~¤P□__3|-
øm□ŽcÔ®u□Bäi6·Ž±ÙÁ!]Ò□71Ï□□£ŽO¨Ç?L×□þÏ¿ò,k□ô□□ý□h□‡Òü
{sñ'Xøo□ˆ/aðí¶Ûéã□óH˜B«é»÷‹“Œqœ□□[ÇÃ‘|,øÁá¨t+«¯ìÝg6óÛÍ&ìœãÐd□
ÊGpAìq[^-ÿ “žñgý,£ÿ Ðm©~'□ÉXøwÿ _gÿ CJ «ã³}ãßŠv¾□Šúk=-Ú□s¨y'□!Æì{ðP

ð 'ž•□ãŸ„ö>□ðìž)ðž¡¨Zj□aYNé·o\…' Áç'± ŒsW|t÷¿□þ-
[xìXÏu£^[‹{Ó□ÉC€¿AÀB3×□qÖªxÛâ¥§•4□ð§„4íBóPÔÊÆÅ¢Ø#MÀžçÓ□œ 9
_□u+¿□h

/í%[illegible]÷×ò[illegible]²#[illegible][illegible]Ì|±•G#[illegible]Ó½Mã/ƒzo…¼
s¯Xêšƒë–[illegible]n[illegible]é¥Ú$mÃq

¯RG9ÏsV<}¢ÿ Â:Ÿ
4•ÊÍiv±;/F`bÜG±9¯Eø©ÿ $»Ä?õê□˜

[Ä°M••~
ÙøƒYY$¿²Òd°ŠD}¿½òþb@à,P□V□Á¿‡>-—ÃZ□‹ ž□ÛUÌ¯»Î;2$t/Ð
è¼?g>¡û?Eem□'â}□XâAÕ˜£ ?□\×Áï^ú=®ƒ¡x.[{ïíq$°□!□□.ï'sœ`ã§QéÍ szG□›Ç?□|ek
u¨Ïm¤Ûß¼—PÀøiÛ{l-œrrsŽÝr6ìt□øcñ³EÓt[«"¤ë02Ëo,›¾a»'
AëÉ□ë_áwü•O^¿õûÿ ³ÉKãÏù.ž□ÿ uÿ ™

?ŠÂÊãâ¦™iã+›Ûo
□LÀðgg›ÎK`-s€p3•½□5ßü:ðo‡<6/u□

jÓ_X_□□Lë,hW=□□yÇ<ŠÍñ•Äÿ □éšåï†<Q¤ÜÍ□A□|-Ë,2nPÀ€NxÎ3Ž ×3ðj(®>
ø‹ Rðå□åŸ„d€$QÏ□

Ù^™'8ýçs€Ã=h□Ü¨¢Š (¢Š (¢Š (¢Š (¢Š (¢Š ç<k□1x
Å2G□hòé—–#*€\ˆXdžü +□à—ü'M□ë?þŽzÏø'ñCÃ:v•â
Ï=Çö›ØËn□`%wÉ□Ú7□À…a|□ø'áÛ?

h~□ži×Ti$Œ/’J□y□”n-ÄP□·□□“Ò¼ZÎañ□ã´:¶œ□š/‡aò□È9Ydù±´ÿ ¼ßB□=Åg|^ø˜Ï®Éà»[
©4Û□BjWÁ
;) ”E□ã□ž™<p2N×ƒ¾'ü6Ð,,|?¢½äQ³ª□{c°I□€]Ïr□ŀÃ€□ tž ø¿á/

ëw□F£qt.íÈ□□íË H□sô"·ü=â□□Ç□□Ô,□Ú,Œ¥q<8Ã©□¡ô8 Ö”úV•u)–
âÂÖY□W’□b□□*Ž·¬é>□ðôÚ•â‹k
r7,□t,À

(÷4□ç>$ÿ "œðŸý,ßù\W□å};JÕ¼□ªYkwB×O'□ËpN<¬□U½ðÀqß§zðígâw†ï~6h>)†["¦YX´□1
„†

DÝ□¿ßZì~#j□âWÂG¿ðŸÚ.¡,õ^x|²®è€î]½ðY[ð □<Óþ"xûGõmÍŽ‟

—°%©6öú×ØŸ1F8□=□ cp%s□Jöo„□>±ð□#Ãú‡Ûã-F’êv]¬f n□□‡

=9ç9<Æ“ñãÁ¶~-¶¶“O¿¶’□□3g□°•□□!N@Ç×JŸàn•{□ø□]“Om;NÕ®–
[□R1µ□sÀÀùpê□ïŠ ôO□□È–®ÿ Ø:ãÿ EµxgÂÿ „¶~1ð”Z·ˆ5+É-Îø¬- —h…C□Ç•y-
“□Çsœñî~.ÿ ‘/]ÿ °uÇþ‹jäþ□□É)Ó?ë¤ßú1¨□Ëü
à[Ï□kZÇ…µ}zñô□□Ý´□f□ñæ9gQŒä□û¶>Ùã□&°□□é•ðûãtÞ□Óî§—GÔlŒé

¯»c□œýFÖ□ô<ç□-□„?òP~&ÿ ØX□èÛŠ]CþNsIÿ °[ÿ è2P□V¡aqñoâ¶-¢ß_ÜÛøwAù

í´É(;NzŒçw8à

¹ª-9ðB|$□□‹¼#}y□Šåa¹¶šMèêÜóÓå;pAÏPF1W/u9¾□|YÖu]FÆæo□ë¿¼□À»ŠÈNãè2

þ3œ□}ª¿□<d¿□Í‡„|%ey$or³]ÝÍ□Ô□□FHÏÝä’N9

æ€□â…□ÏŠþ+øNÓL¾k□¿ÓFÛ€H)□□
tëòäc¾qP|@øoeðÏDÓüOá›ûø¯ìî£Gye□¼□ó€□|□:□Mt^$·KOÚ□Á□Ñ

G□žcAì□ V§Çßù&□□×Ü?ÌÐ□_Æß

iZÇ€ßÅÒÅ Ô-í XXHv„y□,:¾ÕµðËáÏ‡´-
7Hñ□œ3ÿ hÜéÑ4Žó□¹'□›□ëOø□as©|□»·´…¥˜YÛÉ±FIUdfü"□øU□.ÿ □´]vÇFðÍ¤7¿Ú□Új,Ì
Ñ□□˜ÕTüÙïÔqù□ ó?…¿□¡ñÌz•Î¯¨Ü®'mxê-PHW|§«-ÃŒ

9>£□õþ□±"Àß□uo□Z]Ï6•= ¹†9›qVÂœý~ðã¨ÆzUßÙ÷þE•oþÂ•ÿ -*□ÉÏIÿ `'ÿ Š å<So¢
ê□□µ[?ˆ—·¶–□4□YV+

Ò□9ÁÀ<œôÈ95ë¾□ð¦•áM□htMJký>êcq□¼«"€@□V^□ã-qž'ø§à;»íG@ñV‰w#YO$
Mj®□ƒ□Ès•$r□□□Í3à-µÊZøŠî□.-ü?sx□KŠàò□□É‡-
èJûP□°ÑE□ QE□ QE□ QE□ QE□ QE□ QE□ QE□ QE□ QE□ QE□ QE□ QE□ QE□
QE□ QE□ QE□ QE□ QE□ QE□ QE□ QE□ QE□ QE□ QE□ QE□ QE□ QE□ QE□ QE□ QE□ QE
□ QEsž4ÿ „¯û"/øD>Åöÿ 8yŸkû¾^

qïœP□GEx□µã□‹°

¹¥i□§H□z¤,;``«)9□“Û¨®¿HO‹’5êk□hÐDÖS□e□□IöŸ-¸□€Ø'#□Í zu□Ìx
=rßÂ□ãÄšŒ□×û□šxœ2ìÏ °àãÖ·ÚþÉ–Òá®à□¹ÂÈd□[èsƒ@□(¦<±Ç□•äEŒ

—f □ëš!š+ˆ„°È’FÝ□□□□□@□¢›$‰□m$Ž¨Š2YŽ üi•\Áu™o4s'MÑ¸aùŠ –ŠdÓEo□-
iR8×«; □âh†h®"□Ã*K□èÈÁ•üE >Š*□‹»k@¦æâ(C□□#…Ïç@□QH□`□
ƒÈ"¡¸½µµ*..a„·Ý□H□?LÐ□ôR□□□□AèECöÛO%æûT>R□®þ`Ú¤u□ö4□=□ÈåŽhÖH□]□d2œƒøÓn.
-í#ó.gŠ□Î7HáF~¦€%¢š²FÑ UÔÆFw□Æ=sI

Ñ\D²Ã"I□}×F□¡□ ú*4ž□%'$-
6'<oE`Jç¦GjoÚ-ó(ûDY‡ýoÎ>O÷½?□ šŠŽ□á¹^K□±Ë□èñ°`□□M†êÐå□`¸ŠR‡

□Ãm>øé@□QHHPI □É&¢ ·¼¶»

m®!˜)Ãyn□\P□ÔQ_Ä½M□Ã?□Í§ß/•□¾7ÛËó!Ü□Px

ÒŠæ>□O-ÏÃ½□iåyek4,îÅ˜ñÜšéè□’Í□□4³H‘Æ¿yÝ€□êM<□@ ä-õäŸ□.f×u/

x

ÊB&Õ.Vk’½R□8É□ÇÞoûg^¨‹m§YG□)□´□□w□*(□□&€'¢¼Ÿâþ©<W^
}>þDŠmPnky˜W ¯\-zšõ,p2zP□EAoyku»ì÷0Í·†òÜ6>¸§‹ˆMÁ·□FfUÜc

7□ëŽ¸ (¦M4Vñ4³J'F¿yÝ,□õ&œ□e

¤FAèh¢Š (¢Š (¢Š (¢Š (¢Š Ž{x.¢1ÜC±Ÿá'CÈÒÅ

pF#†4□□¢¢€□à)ôP□H@ ,2□Pih
ðXY[Hd·´,'=Z8Â“ø□V(¢€#š□ncòçŠ9S9Úê□gèiìªêU”2‘,□È"-
Š dQG□K□1¤q¨Â¢(} ¤Š□a.bŠ8Ì•¹Ê(□•©õ5%□□□Å¥µÚ…¹·Še□ H□€üêTED□Š□@À `
Z(□4,□¤’Hâ•-B
²¨□ˆõ=èx!’hæx£icÎÇ*
\õÁíRQ@□Ïo□ÌF)âŽXÏT‘C□ø□□¼□ßìæ□Ì;vùeFÜzc¦*J(□¨‹□*"…E□UQ€□ ¦Å□
0oòbŽ=ìY¶(□‰êN:š’Š ¯□…□¼Í,6□G+utŒ□□□V(¢€
(¢€
(¢€
(¢€
(¢€
(¢€
(¢€
(¢€+¥…œW□â;H□cÖEŒ□?•Z±E□ Uk□:ÆñÃÝYÛÎà`4±+□=9□fŠ ¢š.•□«¦™f¬§!„
□>½*{›+KÐ¢êÖ□Âýß60Øúf§¢€(□aéô
±ÿ Àtÿ
¾ 0□AE□□□°C3#K□r□Ûr□PvŸQèie†9âh¦□$□†□□A□ê□>Š EUU
□□ □À□È`†Ú1□□$Qƒ•¨¡Gä*J(□
ì-.□^âÖ□Y>é’0Ä}3S□□□E□□□0Cn¬°Å□a˜³□P2ORqÞ•"-
´}£Ê•ÎÛ³ÌÚ7môÏ\T”P□sÛÃs□Šx£-3Õ$PÀþ□šö-Ò[‹w·‰¡□Äl€¨ÇN:TÔP @#□¥G

□ÛÄ",$Š1Ñ□B•ø
’Š ¯□…□¼Í,6□G+utŒ□□□V(¢€
(¢€
(¢€
(¢€
(¢€
(¢€
dÐÅq□E<I,m÷‘Ô0?PiôP□□ì=#þ•V?ø

ŸáS‹□1hm□¤□Øõ„F6uÏN•jÅ□□Cû□Hÿ U□þ□§øU›kKk8Ìv¶ñ@„ä¬H□□ë□SQ@□TŸJÓ®¥2ÜXZÍ
!êòB¬OâE[¢€)Ã¤é¶ò¬°iö‘H¿uÒ□R>„
±4□\Fcš$-3Õ]C□ø□’Š ŠÞÖÞÑ6[A□)×lh□~•-
□P□m□/2LÑFÒ !□¨, õÁíDÐCp□'Š9T□À:† Ž‡š’Š k¢ÈŒŽ¡‘†□Xd□èhŽ8á□c‰□#Q…U□ {
u□□□ØÚKp·□ZÀó/I□0X~=jb□□□□-□4´P□!†+x-
("H£^ˆŠ□□ÀR$□Ç,’¤Q¬’c{ª€[□2{Ô”P□7□¶÷j□æÞ)•N@‘□ □□t-
ðJc2C□˜ÛrnPvŸQèjJ(□²F’ÆÑÈŠèÃ

¬2□ô"šmà6ÿ g0Æa□o—´mÇ¦:T”P□6ö¶ö^RÚ□¡BrV4
?JšŠ(¢Š(¢Š(¢Š(¢Š(¢Š(¢Š(¢Š(¢Š(¢Š(¢Š(¢Š(¨□ÆÑnMÊÚÀ'=e□•ÇñëSÑ@□
□Q@□\YZ]íûM¬3méæÆ□•J^‘ DUTQ€ª0□:Š *4,□¤’Hâ•-B
²¨□^õ=êJ(□3□-:ÎbŒÌ£jÈTn□Ð□&,□€¢h£•+□Pê

□èF{Ô”P□PCcio+K□¬□HßyÒ0¤ýH©è □æ,□˜ü¹âŽTÎv°†□ú□□€F□J(□á,+x„PD‘F:"(P?□Mk
Kf¹[-·ˆÎ£□RƒpüzÔÔP□b□EĀ¸□F&+´É´n#Ó=qD°C>Ï:(äØÁÓzƒµ‡qž†¤¢€#žÞ
˜ü»ˆc–
3ü2(aù□!,+x„PD‘F:*(P?□RQ@□□Q@□□Q@□□Q@□□Q@□□Q@□□Q@□□Q@□\ZÛÝÇåÜÁ□Éœí‘□□ÈÓ
¢Š8c□Å□Æ‹ÑT`□ÂŸE □Ž‹"2:†F□ea□G¡¥¢€(□aéô
±ÿ Àtÿ
³mgmf…-má□IÉX□('×ŠšŠ t=$’N—dIÿ §tÿ
žÚÂÎÈ±µ´,□ßxÅ□\ýp*Å□ TpÁ□°²Ã□q†bÌ□@É=IÇz’Š ŒA
ÎÓ¬Q‰˜□i□•Ä

ÄÐðC,±É$Q¼'ä£2,W=p{T"P□2†R¬□□`ƒÞ¡·³µ´ÝökhaÝËyh□?\TôP□rÁ

Ì•,QÈcmÈ]AÚ}G¡§I□K□G"+£

2°È#ÐŠu□ Ô•"•c•□□F□T`□è□F¶-Ép× o□ÎÃ□ @□ýOZšŠ •
†)$’8£G□,ìª□b=Oz-□dš9ž(ÚXó±Ê,W=p{T”P□7□-
×j□æÞ□”r□ˆ□□Î¤DH□$j¨Š0□F §Q@□□Q@□□Q@□□Q@□□Q@□□Q@□□Q@□'Ò´ë©L·□□³Hz¼•«□ø‘
I

“¦ÛÊ²Á§ÚE"ý×HUHú□*å□□R}+N°”Ëqak,‡«É
±?‰□ÄÑ´¸¤Y#ÓlÑÔåY`PAõ□□zŠ)“C□ÄM□ñ$±·ÞGPÀýA§Ñ@□?°ô•ú□Xÿ à:□.[,Þ□X„VðÇ

c¢F¡@ü□IE V}>ÊK•´=•»M×Ì1)oÏ□«4Q@□@`A ƒÁ□›

1[Ä±A□E□ôDP ~□ŸE G□□Âò<QF□!ÜåT□ÇÔúÐ`„Ü
ƒ□~p]¢M£p-™ëŠ’Š l‘Ç4m□¨®•ÁV□□ð¦Am□¬~]¼□Â□vÆ□Gä*Z(□6,□™&h£iP□ŽT□Pzàö¢h!¹
ˆÅ<I,gª:†□ð5%□ t¨a´¶·wx-â‰¤9vD
[ëŽµ5□□□0Cn¬°Å□a˜³□P2ORqÞ•"´}£Ê•ÎÛ³ÌÚ7môÏ\T”P□{‹□K¦V¸µ,f_°dŒ1-
LÕ€ □€;QE □QE □QE □QE □QE □QE □QE □QE □QE □QE □QE □QE □QE □QE □QE □QE
□QE □QE □QE □QE □QE □QE □QE □QE □QE □QE □QE □QE □QE □QE □QE □QE □QE □QE
 xÿ Å□ù*ÿ

ÿ ëóÿ j%z?‹¿äK×□ì□qÿ ¢Ú¼ãâ‡ü•□‡□õùÿ µ□½Åßò%ë¿ö

¸ÿ Ñm@-uðóþMÖëþ¼¯¿özä~□ü%³ñ□„¢Õ¼A©^InwÅem□»D*□î<ƒÉlœ

;œçŽ»áçü›-×ýy_□ìõ³ð;þIN™ÿ]&ÿ Ñ•@-_à_□Þx»ZÖ<-«ë×• xní£û4o□1Ë:Œg
□Ý±öÏ□É5Óø?Lo‡ß□¦ð®Ÿu<°>£dgHe}Û□

çê6°Ï¡ç8Íh|!ÿ ’ƒñ7þÂÃÿ FÜRêòsšOý,ßÿ A’€8Ÿ-
x«Gñ□Äûí/ÅZ□ý·‡t²bŠÞÉre˜`1oÄ·8è1ÆsYv- ðÇ…üu¡Þx□PÕ

□□
□ý¥Ð;Z2À`zõn¹Á ×W-<□

þ-êšÎ³£5÷‡õ¡¹fXVC‡

~÷□Èn22□=±]&•ñWÁ□§ˆ´í3EĐ.'°¹□QeK□A□OÞ'9Àêxà□@□÷ÅaeqñSL´ñ•Íí·…□¦`x3³Íç%°
□9À8□ÆÞƒšïþ□x7Ãž□□°‡†5i¯¬/,□¦u-4+ž„

¼ãžEføÇâ□„tÍr÷Ã-(ÒnfŠ •¾Ke-□7(`@'<g□ÇPk™ø5□W-
□|E©xrÎòÏÂ2@□(çÈ□l¯L"œ~ó¹Àaž´□í□•òizMæ¡(&;X-v□¸U,□•xg□¾-
CñCN°ñ□‹ï¯g¸¼□ÖÞ8eØ±¢œqÁÆ

@□ □óÇ¹j¶ ªè÷°t•µ.–ä•• e+ýkÂü□ñ□ßá¦™sá□XÞÛÜÙNæ□Š–ÁÔœã¨êrA□□□0

Æ×H¹ø?à/□]&-.¡i□ótø§_õ□ü -yË0Î08Ï□®kÁß□l|aáè|KâÝCQ»Ôõ0gÊÍ´F§îö98çÐd

q]□Z½ßÆ□ øšÖ□"k
IT&•-Ã`Ü0ù□=‡Ìª

2□zœW7à¿Œ:w„ü7□‡|Ua¨Úêzb˜
¬□Þ£îŒ□□8ÀçŽ3žp .|9kï□|PÕ~Ë}-Ð—ä}¦ÄÍÉN□`zpXR¹ÀÍq¿
~□Eã˜õ+•_Q¹]"ÖñÕ, •®ùH□cž□Ë□Ç'ÔcžßáÌ7þ2ø"ªüB¹±-ÏMh>Í§¬Ã

ã n-ØSžÙl□pj_Ù÷þE•oþÂ•ÿ - Rð□Œž□øË«x:Òîy´yí□Ì1ÌÛŠ¶□çë÷‡□F3Ò°¼So¢ê□□µ[?
ˆ—·¶-□4□YV+

Ò□9ÁÀ<œôÈ95Õ§üœôŸö   ú□¤ñ?Å?□Ýßj:□Š´K¹□Êy!□kUpÄ□nCœ©#□xàŽh□bËÂšW„þ□x-
□□RšûO°³¹¸‰ÐU'@0'…eàŽ:Óþ□□É'Ò□Þ›ÿ Fµr□□t{½GÂÞ4†Ö+›]□Si"Ò£¹'*□dR□#□$g%}«
3À□4ÿ xZO

ø‹NÔ"Ô´ù¤UŠ8•Þ
Ýƒ’0rO¶0Fh□¥øsÿ %•â□ýv_ý□×□¤x□øëâÏŒ-nõ□‹}&Þýä¹†□!§mì□zqórrGA×
#□àÆ£u«üCñ–£ydöW□%%kg□□òÄ€r□éŽÕ¥ð»þJ$Ä_úýÿ Ùä

'Tð-æƒñJ-
□øw]¼±ÒõÛQ4Áœ’#□Ë)Æ2□vØéÁÁ8&´|_à»O„°¿†üCá»ËÕÍâÛÝG4¡¼Õ8' □□□□ôÆ1]/ˆ?äçü)
ÿ `™?ô□š_•ÿ ò/h?ö□Oý□Ð□=ñ‹ÅV·ž=´ð®–©]ØøzÞ%-
ûì‹™%v□”{ŒmÆz□O5ÄjÚ¿,ü9=Ž¯ð÷SÕáÕ`™wÇr§Ëxûç§R #¡□½□â5¬¾

ø§eã™ô"¨è"Â ½Q□□┼
±³8<□•¤g©□qVÛã□Ã÷xcÓü;uys+□H¢Óã□q=9=~™ □ø§«_ø□Rð¯ƒ´Û™,ã× ží—
ïyG□□¦@□É□ð+□â□Á•'Ã>

½Ö4□Fþ□mb-¦sK¹n□•□8□□¡ôã§zÞøÁk}¢x—Ã^:²µ′æ□-ü»ÅAÊÇœ• ¸Ïc•ZÃñÿ Æ□7Å^

¿Ñ¼;¦j□2ÜCºæYbÚ¶ñ,
□,rz□Nzž”□ê_□?äšø{þ¼’ºªå~□□É5ð÷ýy%Tø¯âOøF>-ê71¾Û«•û%¿8;ß#
ú…Üß… rŸ□?â³ø¥âO□¿Ïeh~Á§1åp8,¾‡hÏý´5›¨é’|Wø½ªèú•åÄ^Ð□(,□Û¾N□öÉ;¹ÇE□Þ½-
á·†ÿ á□ð□™§:m¹1ù÷9□>küÄqÂÿ ÀkÍouvøQñ‹XÔõKK—ÐµÔó□xSv-
,□#¸□œàƒì@0|uðêÛÀ¾&ðÄš]ýÌšmÞ¢Ÿè³¾ï.@Ëó□8
ƒŽ™□ês^·ñ#ÃKâ;+4Ô<Jtm□□f¿Pâ??8Ø
±ÀÇ=r9□p+É¼sñ□/□x□Ã?ÙzmÔzMž¤€^N›|ÙY—å□ À□ëžz

ý□Ç□òüQá›-rÞîçÂ‘’.c·$fLóœc’¸Ç ðØ4□Çø–
□□ø'TÑµŸ□x‰ç¾†éV{qqæ□N¤ä□ÁÆ□ä□×yñ□□Â□ñsÃ^2O’ÊôýŠý°(þ□Ì□Ý`@ÿ ¦uæ-:Ö<□¨ÚØ
Áàß

Kj±\¡šù£*

AÂrI9Á98û½ëè□Šž□ÿ „›áæ§k□n¹•>ÕoÆNôç□Ü®åÿ •P□-
ñ¦âmjïÃž□²r'ÕnÖIÊòR%8□Žã%›þ□^¯km□•œ6¶è□□#XãAÑUF ü…xgÁÆ¾ñ·Œn<]ª©oì»□tûrÜ
üû0Ì□©□Éÿ ®•ï□ QE□ QE□ QE□ QE□ QE□ QE□ QE□ QE□ QE□ QE□ QE□ QE□ QE□ QE□
QE□ QE□ QE□ QE□ QE□ QE□ QE□ QE□ QE□ QE□ QE□ QE□ QE□ QE□ QE□ QE□ QE□ QE□
QE□ QE□ QE□ QE□ QE□ QE□ QE□ QE□ QE□ QE□ QE□ QE□ QE□ QE□ QE□ QE□ QE□ QE
□ QE□ QE□ QE□ QE□ QE□ QE□ QE□ QE□ QE□ QE□ QE□ QE□ QE□ QE□ QE□ QE□ QE□ Q
E□ QE□ QE□ QE□ QE□ QE□ QE□ QE□ QE□ QE□ QE□ QE□ QE□ QE□ QE□ QE□ QE□ QE□
QE□ QE□ QE□ QE□ QE□ QE□ QE□ QE□ QE□ QE□ QE□ QE□ QE□ QE□ QE□ QE□ QE□ QE□
QE□ QE□ QE□ QE□ QE□ QE□ QE□ QE□ QE□ QE□ QE□ QE□ QE□ QE□ QE□ QE□ QE□ QE
□ QE□ QE□ QE□ QE□ QE□ QE□ QE□ QE□ QE□ QE□ QE□ QE□ QE□ QE□ QE□ QE□ QE□ Q
E□ QE□ QE□□□ÛÃ,'É$Q¼''Q™A+ž¸=©ä□□□□<□ih □â,□a□Å□qÄ□□"€ })a†+x-
("H£^ˆŠ□□ÀSè □ã,□^GŠ(Ñ¤;œª€XúŸZ

□›•pb•Î
´I´n□Ó=qRQ@□’4-3□ˆ®•ÁV□□ð¨íìím□-µ´0ƒÔF•sùTÔP□7□-
×j□æÞ□”r□ˆ□□Î¤DH□$j¨Š0□F §Q@□Aqgkw·í6ÐÍ·•ó□6>™©è `

, žÊÒéÕî-a•"î™#

GÓ5=□ □••QÃ□6êË

QÆ□‹ 0E□$õ'□êJ(□?"´}£Ê•ÎÛ³ÌÚ7môÏ\Tw□6—L-qk□Ì¿tÉ□b>™«□P 0□j…í-
¥□''Þ'™>ìŒ€²ý□QSQ@□¤□Ç,'¤H²I□îª□ltÉïBA

RI$qF□!□ÙT□Äzžõ%□□□,□□g1FfQµd*7□è□□A□ÀQ4QÈ□ƒ(u□□t#=êJ(□□

x ÷¨
±³µfk{H!fêcŒ)?•X¢€j¼v6Å$QÚÀ'É÷Ñc 7Ôw«PR4Š5Ž4TEUQ€ ÇxÏÀÒxÃ]ðý
Ä÷Èšf™9žkB„ùíë@ÎqŽ1ô&»:(¨æ,+ˆŒSÄ'Æz£¨`RQ@ýŽÔÇfÚ'Ñ®Á„#¡±©$%£'
Ñ†
°È?…:Š ®¶k •m +nˆÆÐÞ¸ÇZÉñ¬Ú´>Õ[Cµ'çSh

vñÆ@`ÌBî□î -
ü+zŠ ã¾□xUü!à;□:â1□ì™¸°□þz7cî Uÿ €×cE□ QE□ QE□ QE□ QE□ QE□ QE□ QE□ QE□
QE□ QE□(¢¸□ñ³áâ±□Ä# ã‹9ÏþÉ]_‡üE¥x£K□–•uö›6r,O-
‟æ□F□□úP□¥□Ëëß□¼#á›Ãg«ëpAr1°□W•—?Þ□

?□ÔÐ¼G£øšÈÞh°„7□□µŒg•>Œ□
ýE jQE□ QE□ QE□ QE□ QE□ QE□ QE□ QE□ QE□ QE□ QE□ QE□ QE□ QE□ QE□ QEqÚŸÅO
□é□Íeyâ□q:□¬±#Ê□ú□Š@?• v4VrkÚ\°□k±ÞÅ&-°µÁ¹L²ùj
cÇ<`ñ×Šä?ávü<ÿ ¡‡ÿ $®?øÝ z□□OJÕlµ½.ßSÓ¦ó¬î□|RmeÜ=pÀ□øŠ¹@□□Q@□
□Q@□□Q@□□Q@□□Q@□□Q@□□Q@□□Q@□□Q@□□Q@□□Q@□□Q@□□Q@□□Q@□□Q@□□U]GR²Ò,e¾Ôn¡µµ^eå™
Â¨üM Z¢¸Ý?â¿•µKô²µñ□¹□Ûj‰#’5cè□”□Ö»* (¬eñƒƒ'‰G‡cÔá“VØÎm£Ë□□®â□□û□□lÐ□E□
P□E□P□E□P□E□P□E□P□E□P□E□P□E□P□E□P□E□P□E□P□E□P□E□P□E□P□E□P□E□P□E□P□E□P□E□P□E
□P□E□P□E`x›ÆÞ–ðp¶:ö¢,þÕ»ÉýÌ’□Û□ßqN1¸uõ¬-+âÏ,5½RßMÓõ¿:òåöE-
Ùf]Íé’€□ÄÐ□iEUÔu+-"Æ[íFê□[X†^Yœ*•Ä×/§üWð6©~–
V¾!·3»mQ$rF¬}□2•úÐ□eE□P□E□P□E□P□E□P□E□P□E□P□E□P□E□P□E□P□E□P□E□P□E□P□E□P
□E□P□E□P□E□›-øƒHðå—Û5•B
(Ài[□□¢Ž¤û
Ò¢¹}□â/„¼Q□ò□YŠâë□„F7•˜

£?…hxƒÅz□…mÒ}oS,Í$ÎÀä−|uÂŒ"ø
Ø¢¹¿□xûÂÞ*¸k}□Y,æu□¼¢-□'Ü…p -
Jé(¢Š(¢Š(¢Š(¢Š(¢Š(¢Š(¢Š(¢Š(¢Š(¢Š(¢Š(¢Š(¢Š(¢Š(¢Š(¢¹}{â7„
|3xlõ}n□.F7Bªò²çûÁ□ÇãZš□ˆô□□Y□Í□P†ò v±Œò§Ñ•ä-
¨ □J+Œ¼ø™¤Xß\ZI¦ë¬ðHÑ³G¦JÊH8$□r8ëIañ?B¿Ö,´±m«[ÜÞ¹Ž□´Ø¼JÄ

žM v”QE □QE □QE □QE □QE □QE □QE □QE □QE □QE □QE □QE □QE □QE □QE □QE □QE
□QE □QE □QE □QE □QE □QE □QE □QE □QE □QE □QE □QE □QE □QE □QE □QE □QE □Q
E □QE □QE □QE □QE □QE □QE □QE □QE □QE □QE □QE □QE □QE □QE □QE □QE □QE □
QE □QE □QE □QE □QE □QE □QE □QE □QE □QE □QE □Ë|Bñ=Çƒü!>µm□RI

±)YT•ÚÎ□¸□□àœsÖ°šà>3□É8¹ÿ ¯«oý□´□N□□|FŸN‷^Óü1¦E¤¢□VÊòg□²Æ9Î□åRG;NOÖ°X|w
¡ÿ Â□kâÛË†´Ó'□□™‵œ£1Û´…□’□Ž□t¬□R□ Šó□†W°□‡ÀÍ□ãHÒ□µoWÍ
iö•ƒxûC,w·□□ŸÂ€4?ávü<ÿ ¡‡ÿ $®?øÝt¾-ñ^‹âí:kÍ
÷ívñ9‰ßÊxðØ□□p□B+šÿ „·â-
ý□□ü¯Ûÿ …tþ□Ôµ½NÆYuÍ□û□ádÚ•}±.w®□Í¹□□9□ö □/øañ?Á°ÃýHÔµ¥·¾…\I□A)
LŒGÌ□oB;×°ØßÙê-Q^X]Csm(ÊK
†V-ÄSåµ·š□†X"′&ûÈè
Ÿ¨¯4øoäišß□£ÒSv…k|-k□_pJ#&eNÝvŒ□j §àíSIð&©-é~-XôýRæúk¤Ô®S
{□□,²z•îç¿®kSÀÖ+□åÍwÅze‹Øèw-
ñÁ h¼¯¶893„ã□°$s′}j¿,4=?â□‡âñWŠ6ê÷W¦@-Ò±0Y.â»#N€à

±ù½êM
9<□ñ>/□é×“\è——□v-
‶Hdk□VÆ□•!□L-ÿ ¨□¦QE□ QE□ QE□ QE□ QE□ QE□ QE□ QE□ QE□ QE□ QE□ QE□ QE□□Á
øßÇ—þ□ñ.‰¦ÙéGRþÒŠm°D□šò®Ý€□áW$î$□□šÏ»ñ—•¼/=•÷‹4} h·S¤□I§Ìí-
¡s€dÝÃ□]¿á›¾"ÿ ’Ïà¿úõ¾ÿ Ð□□ÿ ä’ëG¸kr?ïüt□Òø“Åš'„l¢¼×o~É□²yHþSÉ-
Á8Â□z□\Ïü.ß‡Ÿô0ÿ ä•Çÿ □®›Äš-·¦YE.…áÿ í©ÚM¯□Û□ÛbàüÛœ`ó••zæ□á-
ø‡ÿ DÃÿ +öÿ á@□¼uãK=Gá¬Wš□¦□□fî=:+Ò-□Œ;□vù€#
¯ÉÇ-7Gñ×Â¿

ZÅ¡éÚµŒ1 □JBì®}Z@»XŸRj□ÄÙou/‡°.©â□□X
=jÞâúÅ¦[…XC2□²Œ□C□Ï□é□XhÇEx-ÖÇû,ÄK&Å□ùxÎqÓ□

o□}□¾□ëí§y□Ñô›—^ÛãË ÄÇ+Ž0sž+›ð—ÅŸ□CáÍ□M›_Š+¨l †E’□UUÕ ±]½G\â-|□Vÿ …xª
µ—Û.□-üó□óŽ½³°»›û□>öÆK}BÖÞkB§zL€¦ÞùÏ□□b□¢¹□',T-)□2HŒ□X-„□ÔSëÎ¾
å|□y□.ï¦ÇªÜ¦œÌIÍ¸a·□Ó;«Ñh ¢Š(¢Š(¢Š(¢Š(¢Š(¢Š(¢Š(¢Š(¢Š(¢Š(¢Š(¢
Š(¯;ñ□•õ•/ÇÏá}/D]Jy¬RkeRSk-`Í#‟•□□Ó9ã<×¢W□iÿ %ïQÿ ±~/ý□h□;O□ø³Dñ6—
¥xËMÒÖÛUs□-æ™#•Iq□Žœž™¯8Ùñ□Ä□ øSR□v·ªý–èÆ%□ýžWùI □ª‘ØÖWÅ-
¿à£ßþ□›!ÿ ¡Ö¯^µß□iº□ƒDð_öÅ©Œ1¹þÔŠß□‟•ÚÃ<qÏ½ dÿ Âíøyÿ C□þI\□ħºgÅ-
>âöËAÕ#°‟SÓ4ÝA.¯¬c]Í,XÆàŸÅ·==ý3Oÿ „·âý□□ü¯Ûÿ …-6Ôï.üUá¯
%ì°e¶¬%’êâ□Û+,kŸ)±'©□ôÅ cøÏÄ>□ñ¿„□Bðì0ë:□ò□´,□yµ9-
¼□‘û ¾øÏN†½□ôI.|)□‹q¨]G ·Ž□ní¤Ù+□□q□Ûv□=y5Èø—
á×‡ô□□ãVÐ□Ðu=6Ýå†úÚB¤í□±.Oï□Ç;²k©ð^³sâ□i□µäB;›«e’EQ€[¹□Ðõ-
Z âÎ‡¦ø□ã□…,4«8ím—M°;PrÇŒ-'-
>çšõ□óíkþK□…ÿ ì□u^ƒ@□□Q@□□Q@□□Q@□□Q@□□Q@□□Q@□□Q@□□Q@□□Q@□□Q@□□Q@□□Q@□□Q
@□□Q@□□Q@□□Q@□□Q@□□Q@-gqñ□Ä·>4×<-¢hv÷——
’Æ!žFhá†2□‹LÙ9;Ž P23éZ:□‹¼C□‹×ÂÞ/Óì`½¸·k‹;-=ØÃ8_¼¸~C□Ïù□•Á?òQþ
ÿ ×Õ§þ‰£Å||_øz{•Ô•ÿ ¿
@□u□Š¾
Ð5k□/SÖ¼‹Ûr□±ý-
fÚH□r¨AàŽ† ³øÃà=BúÞÊ×]ó..$X¢O²N73□□’˜□žõ>±â?□Yj×□úg€□'¬□□□ßöÄ0ùƒ ‟±†G9□
úT□~(ñä÷Öñ]|8û5»È«,ÿ Û•?-
¤òÛ@ÉÀç□- eüC×tß□üFðF««\ýšÊ□õ òìgÁ1 □('©□«§Ð>!xOÅ□?fÑõ»{‹,2!`Ñ»}□À'ð®”¢
±□¨tt¯1øÏce-•c¨[C□~!MB□Óe□@•¤.>PG$c'-Â€/|UÓnn-4-Diòjzv™¨-ÍýŒk½¤Œ□7□þ-
¾žþ™¬□□ø‡Ã-7ðƒh^□†□gS¾@¶□A□6§#÷’□?t□ß□éÐÖ×Ž5+»¯□øsÂ1_Ë§Zj¾t—
70¶É]c\ˆ‘¿„±êG=1ï_Ä¿

¼?¤è7□¶„

ƒ©é¶ï,7ÖÒ□'h-‰r□x

9Ý"@□Þ—k-Ž'ei<Æi •"yOñ²¨□¿□3Vë
ÁzÍÏ^|□¤j×'□în--I□F□nä□CÔ}kv€
(¢€
(¢€
(¢€
(¢€
(¢€
(¢€
(¢€
(¢€
(¢€
(¢€
(¢€
(¢€#¸□Ãm,ª□(…€>Â¼¯Ã^=ñß□´;{-□BÒ¡(¸¹¼Ô□D,I2r±"'Ä□□˜œg#µz□÷üƒî□ë"□#\wÁïù%
□□ýr□ý□ô□wÀþ-¹ñ,□…®§`,5•.ãì÷–êÛ—8Ê²Ÿî'œ};Õ
¿Œ^□°½žÎç^Ù<□4R'Ù';YN□ÈL-Gj‹Â<|Zø^□MÚqÿ È□¥»ñO□a½ž+o‡-
h□$eŽoíÈ□ÌPxm¤ddsŽÔ□gFø©à¿□jÐiz^³ö‹Û,DQ}-dÝ€Iå•□À=Mqkâ?
Ëñ□Ä□ÿ ‹µ□E□]Ïön™m82□¶ d•F□$-
?{□c□ÚhÞ#ñ¥î-□¾©à/ìÛ''Í»þØ†o/,GÈ£''□•ZÁøsa§ÛøÛÇV×6-
çV□W{•'ã-g'/Ì¸'•½zzÐ□Qáï□øCÅ×aôkû;Ë»u$□□IQO□€à69ÆG-rž!{□

üY_□øŠÑ¥Ñn,□ÖÚøÄdK□Cd†□;wgï{ýj_□ÛÚÇñKÀÒéñÆšÄ—RùÆ
□½¯°O˜¾:Œg□÷§"'□~!xƒHÖg‟û'D0¤zZ9E¸g]ÆIqË€@ÀéÓñ ƒQ}?Æÿ □|=uáˆ’q¤\™ïµˆS□lÇ
ú•ÿ òÐ¶z

à-
s^¥^SâÍ&ÛáÅî™¯ø]šÇíz„V·ZTmû‹µsƒµ:+ŒpWÿ ×êÔ QE□ QE□ QE□ QE□ QE□ QE□ QE□
QE□ QE□ QE□ QE□ QE□ V?‹5iô jÚµ²FóÙÚÉ4k(%K($g□□~5±\ÇÄ□ù&Þ#ÿ °|ßú

cOñwÄ□iPê¾-ðþ•□‰‰X□NGY.›1•Tü«œà±ä`×Gá¿□Yk>
—ÄwÑ¶ž¶žb_ÄÀ¹·xþøàdúôÏ5§á?ù□t?û□Ûÿ èµ®;áì÷V°WŽ¦±³ûeÔ^%Ô

6Þh•Ía³
¸ð¹õ4□gþ□oÃÏú□□ôJãÿ •Öÿ †|sáÏ□½Êè:□ÚÚØ)”y□G·vq÷Ôg¡éXð–üCÿ ¢aÿ •û□ð--
]ñ□å½üÚ÷„ÛG□ñï„.¡□ÉŸ,H□□ËŒ

½s@□OƒµM'Àš¦ ·¥øµcÓõK›é®"R¹L%ìLr
Éê?»žþ¹-O□X-ÿ •5ß□é–/c¡Þ[Ç□□¢ò¾ØàäÎ□Œ

À‘ÎIõªþ□Ðôÿ ˆ>-
‹Å^(Û«Ý^™□[JÄÁd»Šì□:□€2Çæ÷©4(äðoÄø¼#§^Ms¢^X5ÚZM!‘¬Y[□V<„=0{þ -™^{¢-
øK>)jzáùôï□©Ól□f¸ngqî□□é[¾=ñ□x[Á÷°„#uë□□œ`d¼ïò □¾
Î=□©|□áÕð¯„tý$•ÓÇ-û‰3’ó7Ìç=þb□ (□ ¢Š(¢Š(¢Š(¢Š(¢Š(¢Š(¢Š(
¢Š(¢Š(¢Š(¢Š(¢Š(¢Š(¢Š(¢Š(¢Š(¢Š(¢Š(¢Š(¢Š(¢Š(¢Š(¢Š(¢Š(¢Š
(¢Š(¢Š(¢Š(¢Š(¢Š(¢Š(¢Š(¢Š(¢Š(¢Š(¢Š(¢Š(¢Š(¢Š(¢Š(¢Š(¢Š(¢
Š(¢Š(¢Š(¢Š(¢Š(¢Š(¢Š(¢Š(¢Š(¢Š(¢Š(¢Š(¢Š(¢Š(¢Š(¢Š(¢Š(¢Š(
¢Š(¢Š(¢Š(¬□□ørÏÅZ+éWòN□<‘ÈZ□□²Œ□rA□G¥kÑ@□dxcÃ¶~□ðí¦‰`ó=-®ý□;□s¹Ë□□ ê
ÇµkÑ@□UÔ´ëm_L°Ó¯□½µÌM□ª¬T•#□‘È«TP□Ÿ□Â¤Óöy'Äþ+6}□¡ÕO”G¦1œ~5Øè°&›áí.□7I´Ž
ÖÒ!òÆž½É'’O©æ´(

ëá>ˆ×óÝéZ–¹¡5Ã——=&øÀŽÇ©Û,□á•[>□ðF•áIn.,–â{ë□□÷·"□f□

Å□oaŠèè □Š(□Š(□Š(□Š(□Š(□Š(□Š(□Š(□Š(□Š(□Š(□Š(□Š(

< ß

Yßx›L×¥’qw§G,pª°ØD€□Ü1“ÓŒ□G‰ü;gâÏ□]h—
ï2ZÜìÞÐ0W□]\`□GU□«^Š (¢Š -¨XZj°|ö□Ð$ö³¡ŽXœpÊk„Oƒš□Æ-_Wñ

°X<i•¨·Ù±ýÝ g•z%□□□ªx_JÕ|9ýƒ,□

žª‹[9ˆÇ°,»Hé,rçá-—8òoüEâ•BË½•Ö¨Í
B •z••®›e•”Ám
Ž(×
ª;SÑE QE QE QE QE QE QE QE QE QE QE QE QE ‘-
‡,ãñlÞ$Y'ûl¶kfÈXy{nÈÎsïøV½‘¯xvÏÄGL7•2g_Å¨Cå0’<í•r¿1È>õ¯E V-
‰ü%£ø°Æ;]ZÜ¿’þd3Få$…½U‡#ùp+rŠ óñð‹H˜¢jšç‰5kE
‹;ýIž-:| ç]†£¤E}¡É¥Css§DÑˆÒ[<©!QŒl8ùzc§JÐ¢€<Ýþ

ÙË}□ôž2ñ‹ÞB¥#¸mL□□OP□f@>•ÛèzOö-‘

Ÿý¡□˙yE□Úoæóf|’~fÀÎ3□ì□hÑ@□□Q@□□Q@□□Q@□□Q@□□Q@□□Q@□□Q@□□Q@□□Q@□□Q@□□Q@□
□Q@□□Q@□□Q@□□Q@□□Q@□□Q@□□Q@□□_‡,ô□kXÕmä□§Õ¤ŽIÕØ□R‹´m □8õ&•CÃ¶z—
ˆ´mrg˜]i>□ÙÕ□□o5□¶áŒž□□#ñ-z(¯¢Š(□”ñ□€tï□j«ª□GWÓo„B□>›xÐ³($€zŽçµE¢|6Ñt}Z
=Z{□KWÔ¢□ŠëU°3¼_îô ûã5ØQ@□~'ð–
•âë□íukrþKù□Í□”’¯□õV□□åÀ®l|"Ò&(š¦¹âMZÑH"Îÿ Rg‡ŽŸ(□ù× Q@

†□-àŽ□cXâ•B" ÀU□ □ØSè¢€
(¢€
(¢€
(¢€
(¢€
(¢€
(¢€
(¢€
(¢€
(¢€
(¢€
(¢€
(¢€□,k4/□gk©S•CY¾□Ð-</áû=□Åæ{kU*□3□ç,[’ □IíZ´P□F•áÛ=3Ä:Î·□Ìnµ□'í
ì
/”›□hÆG□œ“øV½□P□\§‰>-èþ$ÔâÕZ{ý;T•<±}¦Ü□%+èN□?-
k«¢€9•□x□GðÅä×ð5Ýî§2ì’ÿ PœÍ;/÷w-ƒè*/□ü<Ñ|G©¦ªò_iú¢¦Ï·i×□
Jö□ŒƒùfºÊ(□ŒÒ>□èÚf¯□«wyªë7öç0O«^□ÌGÕF Ï¾+³¢Š (¢Š (¢Š (¢Š (¢Š
(¢Š (¢Š (¢Š (¢Š (¢Š (¢Š (¢Š (¢Š *Žµ¤Ûëº%î“tÒ-½ä-

•□□,°ÁÁ Œþ□zŠ -§ÙE¦é¶¶□-1[B□¡s’U@□>ø□GAðíŸ‡F¦,ÞgþÑ¿—
P›Í`q$˜Ü□ ax□□'Þµè □Š(

ëá>ˆ×óÝéZ−¹¡5Ã——=&øÀŽÇ©Û,□á•[>□ðF•áIn.,−â{ë□□÷·"□f□

Å□oaŠèè

]kÃZö©£ß^Ë9TææUu`#y1…gÉ+Ô`Ž½ëjŠ(¢Š(¢Š(¢Š(¢Š(¢Š(¢Š(¢Š(¢Š(¢Š(
¢Š(¢Š(¢Š(¢Š(¢Š(¢Š(¢Š(¢Š(¢Š(¢Š(¢Š(¢Š(¢Š(¢Š(¢Š(¢Š(¢Š(¢Š
(¢Š(¢Š(¢Š(¢Š(¢Š(¢Š(¢Š(¢Š(‰ø…ñ*ÇáçöwÛl..¾ÝæìòYFÝ›3œúï•qðÒzý u-
ûí?Æº/‹ï¾ .•ÖwÖö¿`ó·ùÊÇvÿ /Ç¦Ãù×™Å:kðÓÿ ï‡ÿ
êÿ á¤ô?ú ê?÷Ú•ðÒzý uûí?Æ¹OøgMoþƒšýðÿ áGü3¦·ÿ AÍ?þøð

¯þ□OCÿ

£ÿ }§øÑÿ □'¡ÿ Ð□Qÿ ¾Óük”ÿ †tÖÿ è9§ÿ ß□þ□□Ã:k□ð□Óÿ ï‡ÿ
êÿ á¤ô?ú ê?÷Ú□•ðÒzý uûí?Æ¹OøgMoþfš□ýðÿ áGü3¦·ÿ AÍ?þø□ð

¯þ□OCÿ

£ÿ }§øÑÿ □'¡ÿ Ð□Qÿ ¾Óük”ÿ †tÖÿ è9§ÿ ß□þ□□Ã:k□ô□Óÿ ï‡ÿ
êÿ á¤ô?ú ê?÷Ú□•ðÒzý uûí?Æ¹OøgMoþƒš□ýðÿ áGü3¦·ÿ AÍ?þø□ð

¯þ□OCÿ

£ÿ }§øÑÿ □'¡ÿ Ð□Qÿ ¾Óük"ÿ †tÖÿ è9§ÿ ß□þ□□Ã:k□ð□Óÿ ï‡ÿ
êÿ á¤ô?ú ê?÷Ú□•ðÒzý uûí?Æ¹OøgMoþfš□ýðÿ áGü3¦·ÿ AÍ?þø□ð

¯þ□OCÿ

£ÿ }§øÑÿ □'¡ÿ Ð□Qÿ ¾Óük”ÿ †tÖÿ è9§ÿ ß□þ□□Ã:k□ð□Óÿ ï‡ÿ
êÿ á¤ô?ú ê?÷Ú□•ðÒzý uûí?Æ¹OøgMoþƒš□ýðÿ áGü3¦·ÿ AÍ?þø□ð

¯þ□OCÿ

£ÿ }§øÑÿ '¡ÿ ÐQÿ ¾Óük”ÿ †tÖÿ è9§ÿ ßþÃ:kðÓÿ ï‡ÿ
êÿ á¤ô?ú ê?÷Ú•ðÒzý uûí?Æ¹OøgMoþfšýðÿ áGü3¦·ÿ AÍ?þøð

¯þ□OCÿ

£ÿ }§øÑÿ □'¡ÿ Ð□Qÿ ¾Óük”ÿ †tÖÿ è9§ÿ ß□þ□□Ã:k□ô□Óÿ ï‡ÿ
êÿ á¤ô?ú ê?÷Ú□•ðÒzý uûí?Æ¹OøgMoþƒš□ýðÿ áGü3¦·ÿ AÍ?þø□ð

¯þ□OCÿ

£ÿ }§øÑÿ □'¡ÿ Ð□Qÿ ¾Óük”ÿ †tÖÿ è9§ÿ ß□þ□□Ã:k□ð□Óÿ ï‡ÿ
êÿ á¤ô?ú ê?÷Ú□•ðÒzý uûí?Æ¹OøgMoþfš□ýðÿ áGü3¦·ÿ AÍ?þø□ð

¯þ□OCÿ

£ÿ }§øÑÿ □'¡ÿ Ð□Qÿ ¾Óük”ÿ †tÖÿ è9§ÿ ß□þ□□Ã:k□ð□Óÿ ï‡ÿ
êÿ á¤ô?ú ê?÷Ú□•ðÒzý uûí?Æ¹OøgMoþfš□ýðÿ áGü3¦·ÿ AÍ?þø□ð

¯þ□OCÿ

£ÿ }§øÑÿ □'¡ÿ Ð□Qÿ ¾Óük”ÿ †tÖÿ è9§ÿ ß□þ□□Å:k□ð□Óÿ ï‡ÿ
êÿ á¤ô?ú ê?÷Ú□•ðÒzý uûí?Æ¹OøgMoþfš□ýðÿ áGü3¦·ÿ AÍ?þø□ð

¯þ□OCÿ

£ÿ }§øÑÿ □'¡ÿ Ð□Qÿ ¾Óük”ÿ †tÖÿ è9§ÿ ß□þ□□Ã:k□ð□Óÿ ï‡ÿ
êÿ á¤ô?ú ê?÷Ú□•ðÒzý uûí?Æ¹OøgMoþfš□ýðÿ áGü3¦·ÿ AÍ?þø□ð

¯þ□OCÿ

£ÿ }§øÑÿ □'¡ÿ Ð□Qÿ ¾Óük”ÿ †tÖÿ è9§ÿ ß□þ□□Ã:k□ð□Óÿ ï‡ÿ
êÿ á¤ô?ú ê?÷Ú□•ðÒzý uûí?Æ¹OøgMoþƒš□ýðÿ áGü3¦·ÿ AÍ?þø□ð

¯þ□OCÿ

£ÿ }§øÑÿ '¡ÿ ÐQÿ ¾Óük”ÿ †tÖÿ è9§ÿ ßþÃ:kðÓÿ ï‡ÿ
êÿ á¤ô?ú ê?÷Ú•ðÒzý uûí?Æ¹OøgMoþƒšýðÿ áGü3¦·ÿ AÍ?þøð

¯þ□OCÿ

£ÿ }§øÑÿ '¡ÿ ÐQÿ ¾Óük”ÿ †tÖÿ è9§ÿ ßþÃ:kðÓÿ ï‡ÿ
êÿ á¤ô?ú ê?÷Ú•ðÒzý uûí?Æ¹OøgMoþƒšýðÿ áGü3¦·ÿ AÍ?þøð

¯þ□OCÿ

£ÿ }§øÑÿ □'¡ÿ Ð□Qÿ ¾Óük”ÿ †tÖÿ è9§ÿ ß□þ□□Ã:k□ô□Óÿ ï‡ÿ
êÿ á¤ô?ú ê?÷Ú□•ðÒzý uûí?Æ¹OøgMoþƒš□ýðÿ áGü3¦·ÿ AÍ?þø□ð

¯þ□OCÿ

£ÿ }§øÑÿ '¡ÿ ÐQÿ ¾Óük”ÿ †tÖÿ è9§ÿ ßþÃ:kðÓÿ ï‡ÿ
êÿ á¤ô?ú ê?÷Ú•ðÒzý uûí?Æ¹OøgMoþfšýðÿ áGü3¦·ÿ AÍ?þøð

¯þ□OCÿ

£ÿ }§øÑÿ □'¡ÿ Ð□Qÿ ¾Óük”ÿ †tÖÿ è9§ÿ ß□þ□□Ã:k□ô□Óÿ ï‡ÿ
êÿ á¤ô?ú ê?÷Ú□•ðÒzý uûí?Æ¹OøgMoþƒš□ýðÿ áGü3¦·ÿ AÍ?þø□ð

¯þ□OCÿ

£ÿ }§øÑÿ □'¡ÿ Ð□Qÿ ¾Óük”ÿ †tÖÿ è9§ÿ ß□þ□□Ã:k□ð□Óÿ ï‡ÿ
êÿ á¤ô?ú ê?÷Ú□•ðÒzý uûí?Æ¹OøgMoþƒš□ýðÿ áGü3¦·ÿ AÍ?þø□ð

¯þ□OCÿ

£ÿ }§øÑÿ □'¡ÿ Ð□Qÿ ¾Óük”ÿ †tÖÿ è9§ÿ ß□þ□□Ã:k□ð□Óÿ ï‡ÿ
êÿ á¤ô?ú ê?÷Ú□•ðÒzý uûí?Æ¹OøgMoþƒš□ýðÿ áGü3¦·ÿ AÍ?þø□ð

¯þ□OCÿ

£ÿ }§øÑÿ □'¡ÿ Ð□Qÿ ¾Óük”ÿ †tÖÿ è9§ÿ ß□þ□□Ã:k□ô□Óÿ ï‡ÿ
êÿ á¤ô?ú ê?÷Ú□•ðÒzý uûí?Æ¹OøgMoþƒš□ýðÿ áGü3¦·ÿ AÍ?þø□ð

¯þ□OCÿ

£ÿ }§øÑÿ □'¡ÿ Ð□Qÿ ¾Óük”ÿ †tÖÿ è9§ÿ ß□þ□□Ã:k□ô□Óÿ ï‡ÿ
êÿ á¤ô?ú ê?÷Ú□•ðÒzý uûí?Æ¹OøgMoþƒš□ýðÿ áGü3¦·ÿ AÍ?þø□ð

¯þ□OCÿ

£ÿ }§øÑÿ □'¡ÿ Ð□Qÿ ¾Óük”ÿ †tÖÿ è9§ÿ ß□þ□□Ã:k□ð□Óÿ ï‡ÿ
êÿ á¤ô?ú ê?÷Ú□•ðÒzý uûí?Æ¹OøgMoþfš□ýðÿ áGü3¦·ÿ AÍ?þø□ð

¯þ□OCÿ

£ÿ }§øÑÿ □'¡ÿ Ð□Qÿ ¾Óük”ÿ †tÖÿ è9§ÿ ß□þ□□Ã:k□ô□Óÿ ï‡ÿ
êÿ á¤ô?ú ê?÷Ú□•ðÒzý uûí?Æ¹OøgMoþfš□ýðÿ áGü3¦·ÿ AÍ?þø□ô

¯þ□OCÿ

£ÿ }§øÑÿ □'¡ÿ Ð□Qÿ ¾Óük”ÿ †tÖÿ è9§ÿ ß□þ□□Ã:k□ô□Óÿ ï‡ÿ
êÿ á¤ô?ú ê?÷Ú□•ðÒzý uûí?Æ¹OøgMoþƒš□ýðÿ áGü3¦·ÿ AÍ?þø□ð

¯þ□OCÿ

£ÿ }§øÑÿ □'¡ÿ Ð□Qÿ ¾Óük”ÿ †tÖÿ è9§ÿ ß□þ□□Ã:k□ô□Óÿ ï‡ÿ
êÿ á¤ô?ú ê?÷Ú□•ðÒzý uûí?Æ¹OøgMoþƒš□ýðÿ áGü3¦·ÿ AÍ?þø□ð

¯þ□OCÿ

£ÿ }§øÑÿ □'¡ÿ Ð□Qÿ ¾Óük”ÿ †tÖÿ è9§ÿ ß□þ□□Ā:k□ô□Óÿ ï‡ÿ
êÿ á¤ô?ú ê?÷Ú□•ðÒzý uûí?Æ¹OøgMoþƒš□ýðÿ áGü3¦·ÿ AÍ?þø□ô

¯þ□OCÿ

£ÿ }§øÑÿ □'¡ÿ Ð□Qÿ ¾Óük”ÿ †tÖÿ è9§ÿ ß□þ□□Ã:k□ð□Óÿ ï‡ÿ
êÿ á¤ô?ú ê?÷Ú□•ðÒzý uûí?Æ¹OøgMoþƒš□ýðÿ áGü3¦·ÿ AÍ?þø□ð

¯þ□OCÿ

£ÿ }§øÑÿ □'¡ÿ Ð□Qÿ ¾Óük"ÿ †tÖÿ è9§ÿ ß□þ□□Ã:k□ð□Óÿ ï‡ÿ
êÿ á¤ô?ú ê?÷Ú□•ðÒzý uûí?Æ¹OøgMoþƒš□ýðÿ áGü3¦·ÿ AÍ?þø□ð

¯þ□OCÿ

£ÿ }§øÑÿ '¡ÿ ÐQÿ ¾Óük”ÿ †tÖÿ è9§ÿ ßþÃ:kðÓÿ ï‡ÿ
êÿ á¤ô?ú ê?÷Ú•ðÒzý uûí?Æ¹OøgMoþfšýðÿ áGü3¦·ÿ AÍ?þøð

¯þ□OCÿ

£ÿ }§øÑÿ □'¡ÿ Ð□Qÿ ¾Óük”ÿ †tÖÿ è9§ÿ ß□þ□□Ã:k□ð□Óÿ ï‡ÿ
êÿ á¤ô?ú ê?÷Ú□•ðÒzý uûí?Æ¹OøgMoþfš□ýðÿ áGü3¦·ÿ AÍ?þø□ð

¯þ□OCÿ

£ÿ }§øÑÿ □'¡ÿ Ð□Qÿ ¾Óük”ÿ †tÖÿ è9§ÿ ß□þ□□Ã:k□ð□Óÿ ï‡ÿ
êÿ á¤ô?ú ê?÷Ú□•ðÒzý uûí?Æ¹OøgMoþfš□ýðÿ áGü3¦·ÿ AÍ?þø□ð

¯þ□OCÿ

£ÿ }§øÑÿ □'¡ÿ Ð□Qÿ ¾Óük”ÿ †tÖÿ è9§ÿ ß□þ□□Ã:k□ð□Óÿ ï‡ÿ
êÿ á¤ô?ú ê?÷Ú□•ðÒzý uûí?Æ¹OøgMoþƒš□ýðÿ áGü3¦·ÿ AÍ?þø□ð

¯þ□OCÿ

£ÿ }§øÑÿ □'¡ÿ Ð□Qÿ ¾Óük”ÿ †tÖÿ è9§ÿ ß□þ□□Ã:k□ð□Óÿ ï‡ÿ
êÿ á¤ô?ú ê?÷Ú□•ðÒzý uûí?Æ¹OøgMoþƒš□ýðÿ áGü3¦·ÿ AÍ?þø□ð

¯þ□OCÿ

£ÿ }§øÑÿ □'¡ÿ Ð□Qÿ ¾Óük”ÿ †tÖÿ è9§ÿ ß□þ□□Â:k□ô□Óÿ ï‡ÿ
êÿ á¤ô?ú ê?÷Ú□•ðÒzý uûí?Æ¹OøgMoþƒš□ýðÿ áGü3¦·ÿ AÍ?þø□ð

¯þ□OCÿ

£ÿ }§øÑÿ □'¡ÿ Ð□Qÿ ¾Óük”ÿ †tÖÿ è9§ÿ ß□þ□□Ã:k□ð□Óÿ ï‡ÿ
êÿ á¤ô?ú ê?÷Ú□•ðÒzý uûí?Æ¹OøgMoþƒš□ýðÿ áGü3¦·ÿ AÍ?þø□ð

¯þ□OCÿ

£ÿ }§øÑÿ □'¡ÿ Ð□Qÿ ¾Óük”ÿ †tÖÿ è9§ÿ ß□þ□□Ã:k□ð□Óÿ ï‡ÿ
êÿ á¤ô?ú ê?÷Ú□•ðÒzý uûí?Æ¹OøgMoþƒš□ýðÿ áGü3¦·ÿ AÍ?þø□ð

¯þ□OCÿ

£ÿ }§øÑÿ □'¡ÿ Ð□Qÿ ¾Óük”ÿ †tÖÿ è9§ÿ ß□þ□□Ã:k□ô□Óÿ ï‡ÿ
êÿ á¤ô?ú ê?÷Ú□•ðÒzý uûí?Æ¹OøgMoþƒš□ýðÿ áGü3¦·ÿ AÍ?þø□ð

¯þ□OCÿ

£ÿ }§øÑÿ '¡ÿ ÐQÿ ¾Óük”ÿ †tÖÿ è9§ÿ ßþÃ:kðÓÿ ï‡ÿ
êÿ á¤ô?ú ê?÷Ú•ðÒzý uûí?Æ¹OøgMoþƒšýðÿ áGü3¦·ÿ AÍ?þøð

¯þ□OCÿ

£ÿ }§øÑÿ □'¡ÿ Ð□Qÿ ¾Óük”ÿ †tÖÿ è9§ÿ ß□þ□□Ã:k□ô□Óÿ ï‡ÿ
êÿ á¤ô?ú ê?÷Ú□•ðÒzý uûí?Æ¹OøgMoþƒš□ýðÿ áGü3¦·ÿ AÍ?þø□ð

¯þ□OCÿ

£ÿ }§øÑÿ □'¡ÿ Ð□Qÿ ¾Óük”ÿ †tÖÿ è9§ÿ ß□þ□□Ã:k□ô□Óÿ ï‡ÿ
êÿ á¤ô?ú ê?÷Ú□•ðÒzý uûí?Æ¹OøgMoþƒš□ýðÿ áGü3¦·ÿ AÍ?þø□ð

¯þ□OCÿ

£ÿ }§øÑÿ '¡ÿ ÐQÿ ¾Óük”ÿ †tÖÿ è9§ÿ ßþÃ:kðÓÿ ï‡ÿ
êÿ á¤ô?ú ê?÷Ú•ðÒzý uûí?Æ¹OøgMoþfšýðÿ áGü3¦·ÿ AÍ?þøð

¯þ□OCÿ

£ÿ }§øÑÿ '¡ÿ ÐQÿ ¾Óük”ÿ †tÖÿ è9§ÿ ßþÃ:kðÓÿ ï‡ÿ
êÿ á¤ô?ú ê?÷Ú•ðÒzý uûí?Æ¹OøgMoþƒšýðÿ áGü3¦·ÿ AÍ?þøð

¯þ□OCÿ

£ÿ }§øÑÿ □'¡ÿ Ð□Qÿ ¾Óük”ÿ †tÖÿ è9§ÿ ß□þ□□Ã:k□ð□Óÿ ï‡ÿ
êÿ á¤ô?ú ê?÷Ú□•ðÒzý uûí?Æ¹OøgMoþƒš□ýðÿ áGü3¦·ÿ AÍ?þø□ð

¯þ□OCÿ

£ÿ }§øÑÿ □'¡ÿ Ð□Qÿ ¾Óük”ÿ †tÖÿ è9§ÿ ß□þ□□Ã:k□ô□Óÿ ï‡ÿ
êÿ á¤ô?ú ê?÷Ú□•ðÒzý uûí?Æ¹OøgMoþƒš□ýðÿ áGü3¦·ÿ AÍ?þø□ð

¯þ□OCÿ

£ÿ }§øÑÿ □'¡ÿ Ð□Qÿ ¾Óük”ÿ †tÖÿ è9§ÿ ß□þ□□Ã:k□ð□Óÿ ï‡ÿ
êÿ á¤ô?ú ê?÷Ú□•ðÒzý uûí?Æ¹OøgMoþfš□ýðÿ áGü3¦·ÿ AÍ?þø□ð

¯þ□OCÿ

£ÿ }§øÑÿ □'¡ÿ Ð□Qÿ ¾Óük”ÿ †tÖÿ è9§ÿ ß□þ□□Ã:k□ð□Óÿ ï‡ÿ
êÿ á¤ô?ú ê?÷Ú□•ðÒzý uûí?Æ¹OøgMoþfš□ýðÿ áGü3¦·ÿ AÍ?þø□ð

¯þ□OCÿ

£ÿ }§øÑÿ □'¡ÿ Ð□Qÿ ¾Óük”ÿ †tÖÿ è9§ÿ ß□þ□□Ã:k□ô□Óÿ ï‡ÿ
êÿ á¤ô?ú ê?÷Ú□•ðÒzý uûí?Æ¹OøgMoþƒš□ýðÿ áGü3¦·ÿ AÍ?þø□ð

¯þ□OCÿ

£ÿ }§øÑÿ □'¡ÿ Ð□Qÿ ¾Óük”ÿ †tÖÿ è9§ÿ ß□þ□□Ã:k□ð□Óÿ ï‡ÿ
êÿ á¤ô?ú ê?÷Ú□•ðÒzý uûí?Æ¹OøgMoþfš□ýðÿ áGü3¦·ÿ AÍ?þø□ð

¯þ□OCÿ

£ÿ }§øÑÿ □'¡ÿ Ð□Qÿ ¾Óük”ÿ †tÖÿ è9§ÿ ß□þ□□Ã:k□ô□Óÿ ï‡ÿ
êÿ á¤ô?ú ê?÷Ú□•ðÒzý uûí?Æ¹OøgMoþƒš□ýðÿ áGü3¦·ÿ AÍ?þø□ð

¯þ□OCÿ

£ÿ }§øÑÿ □'¡ÿ Ð□Qÿ ¾Óük”ÿ †tÖÿ è9§ÿ ß□þ□□Ã:k□ô□Óÿ ï‡ÿ
êÿ á¤ô?ú ê?÷Ú□•ðÒzý uûí?Æ¹OøgMoþƒš□ýðÿ áGü3¦·ÿ AÍ?þø□ð

¯þOCÿ

£ÿ }§øÑÿ □'¡ÿ Ð□Qÿ ¾Óük"ÿ ††Öÿ è9§ÿ ß□þ□□Ã:k□ð□Óÿ ï‡ÿ
êÿ á¤ô?ú ê?÷Ú□•ðÒzý uûí?Æ¹OøgMoþƒš□ýðÿ áGü3¦·ÿ AÍ?þø□ð

¯þ□OCÿ

£ÿ }§øÑÿ □'¡ÿ Ð□Qÿ ¾Óük”ÿ †tÖÿ è9§ÿ ß□þ□□Ã:k□ð□Óÿ ï‡ÿ
êÿ á¤ô?ú ê?÷Ú□•ðÒzý uûí?Æ¹OøgMoþfš□ýðÿ áGü3¦·ÿ AÍ?þø□ð

¯þ□OCÿ

£ÿ }§øÑÿ □'¡ÿ Ð□Qÿ ¾Óük”ÿ †tÖÿ è9§ÿ ß□þ□□Ã:k□ô□Óÿ ï‡ÿ
êÿ á¤ô?ú ê?÷Ú□•ðÒzý uûí?Æ¹OøgMoþƒš□ýðÿ áGü3¦·ÿ AÍ?þø□ð

¯þ□OCÿ

£ÿ }§øÑÿ □'¡ÿ Ð□Qÿ ¾Óük”ÿ †tÖÿ è9§ÿ ß□þ□□Ã:k□ð□Óÿ ï‡ÿ
êÿ á¤ô?ú ê?÷Ú□•ðÒzý uûí?Æ¹OøgMoþƒš□ýðÿ áGü3¦·ÿ AÍ?þø□ð

¯þ□OCÿ

£ÿ }§øÑÿ □'¡ÿ Ð□Qÿ ¾Óük”ÿ †tÖÿ è9§ÿ ß□þ□□Ã:k□ð□Óÿ ï‡ÿ
êÿ á¤ô?ú ê?÷Ú□•ðÒzý uûí?Æ¹OøgMoþƒš□ýðÿ áGü3¦·ÿ AÍ?þø□ð

¯þ□OCÿ

£ÿ }§øÑÿ □'¡ÿ Ð□Qÿ ¾Óük”ÿ †tÖÿ è9§ÿ ß□þ□□Ã:k□ô□Óÿ ï‡ÿ
êÿ á¤ô?ú ê?÷Ú□•ðÒzý uûí?Æ¹OøgMoþƒš□ýðÿ áGü3¦·ÿ AÍ?þø□ð

¯þ□OCÿ

£ÿ }§øÑÿ □'¡ÿ Ð□Qÿ ¾Óük”ÿ †tÖÿ è9§ÿ ß□þ□□Ã:k□ð□Óÿ ï‡ÿ
êÿ á¤ô?ú ê?÷Ú□•ðÒzý uûí?Æ¹OøgMoþfš□ýðÿ áGü3¦·ÿ AÍ?þø□ð

¯þ□OCÿ

£ÿ }§øÑÿ □'¡ÿ Ð□Qÿ ¾Óük”ÿ †tÖÿ è9§ÿ ß□þ□□Ã:k□ô□Óÿ ï‡ÿ
êÿ á¤ô?ú ê?÷Ú□•ðÒzý uûí?Æ¹OøgMoþƒš□ýðÿ áGü3¦·ÿ AÍ?þø□ð

¯þ□OCÿ

£ÿ }§øÑÿ □'¡ÿ Ð□Qÿ ¾Óük”ÿ †tÖÿ è9§ÿ ß□þ□□Ã:k□ð□Óÿ ï‡ÿ
êÿ á¤ô?ú ê?÷Ú□•ðÒzý uûí?Æ¹OøgMoþƒš□ýðÿ áGü3¦·ÿ AÍ?þø□ð

¯þ□OCÿ

£ÿ }§øÑÿ [illegible]'¡ÿ Ð[illegible]Qÿ ¾Óük”ÿ †tÖÿ è9§ÿ ß[illegible]þ[illegible]Ã:k[illegible]ð[illegible]Óÿ ï‡ÿ
êÿ á¤ô?ú ê?÷Ú[illegible]ðÒzý uûí?Æ¹OøgMoþƒš[illegible]ýðÿ áGü3¦·ÿ AÍ?þø[illegible]ð

¯þ□OCÿ

£ÿ }§øÑÿ □'¡ÿ Ð□Qÿ ¾Óük”ÿ †tÖÿ è9§ÿ ß□þ□□Ã:k□ð□Óÿ ï‡ÿ
êÿ á¤ô?ú ê?÷Ú□•ðÒzý uûí?Æ¹OøgMoþƒš□ýðÿ áGü3¦·ÿ AÍ?þø□ð

¯þ□OCÿ

£ÿ }§øÑÿ □'¡ÿ Ð□Qÿ ¾Óük”ÿ †tÖÿ è9§ÿ ß□þ□□Ã:k□ô□Óÿ ï‡ÿ
êÿ á¤ô?ú ê?÷Ú□•ðÒzý uûí?Æ¹OøgMoþfš□ýðÿ áGü3¦·ÿ AÍ?þø□ð

¯þ□OCÿ

£ÿ }§øÑÿ □'¡ÿ Ð□Qÿ ¾Óük”ÿ †tÖÿ è9§ÿ ß□þ□□Ã:k□ô□Óÿ ï‡ÿ
êÿ á¤ô?ú ê?÷Ú□•ðÒzý uûí?Æ¹OøgMoþfš□ýðÿ áGü3¦·ÿ AÍ?þø□ð

¯þ□OCÿ

£ÿ }§øÑÿ □'¡ÿ Ð□Qÿ ¾Óük”ÿ †tÖÿ è9§ÿ ß□þ□□Ã:k□ð□Óÿ ï‡ÿ
êÿ á¤ô?ú ê?÷Ú□•ðÒzý uûí?Æ¹OøgMoþfš□ýðÿ áGü3¦·ÿ AÍ?þø□ð

¯þ□OCÿ

£ÿ }§øÑÿ [illegible]'¡ÿ Ð[illegible]Qÿ ¾Óük"ÿ †tÖÿ è9§ÿ ß[illegible]þ[illegible]Ã:k[illegible]ð[illegible]Óÿ ï‡ÿ
êÿ á¤ô?ú ê?÷Ú[illegible]•ðÒzý uûí?Æ¹OøgMoþfš[illegible]ýðÿ áGü3¦·ÿ AÍ?þø[illegible]ð

¯þ□OCÿ

£ÿ }§øÑÿ □'¡ÿ Ð□Qÿ ¾Óük”ÿ †tÖÿ è9§ÿ ß□þ□□Ã:k□ô□Óÿ ï‡ÿ
êÿ á¤ô?ú ê?÷Ú□□z×‡u¨¼Gáë□b□ž(¯"□¬nAe□±Å|õÿ

é-ÿ ÐsOÿ ¾ü+ß|£Káï□izDÒ¤²Ú@"g@pÄw□ □°(¢€
(¢€
(¢€
(¢€
(¢€
(¢€
(¢€
(¢€
(¢€
(¢€
(¢€
(¢€
(¢€
(¢€
(¢€
(¢€
(¢€
(¢€
(¢€
(¢€
(¢€
(¢€
(¢€
(¢€
(¢€
(¢€
(¢€
(¢€□7ú¦ª•noõMU(¢Š
(¢Š(¢Š(¢Š(¢Š(¢Š(¢Š(¢Š(¢Š(¢Š(¢Š(¢Š(¢Š(¢Š(¢Š(¢Š(¢Š(¢Š(¢²<Sq
©Ùø[R°Ñü³¨A□K□‘7□W’1Ü•□-ø¬ß‡ž*o□x6ÓT›Ë□yh®U8
"žxí‘ƒ•z êh®□â‡Œ¯ü)¥éðhË□š¾£t!·GMÙ□ï□;œ•ð*ŸQ¶ñkxóA’ßW´•I[l^Ûn
ÒÈ7o*¤dƒ•Ç<có íh®#Åß□#ð×‰¼?¤Ç□´ë©\ù7.Ó…6ãr Hÿ •□Ï¥v6÷V÷h^Úx¦@pZ7

ôâ€&¢¡’òÖ–naI[î£8

~,¥$($• ä"@
EWkû5•'k¸□.p²□□Öú-†¥ycŽ#+È«□□.Ç □\Ð□è¦E4SÆ$†D’6èÈÀƒøŠä¼/oâˆ¼[â'Ö5[k-1¦ÿ
B,7

Ð©bT□ ÛòàsÔþt□ØQQ
›re□x‰‹ý`Þ>O¯¥,□□ÜÇæA4r¡ãtl□~b€$¢ŠŠææ□;Y®®□G

(ÒHç¢¨□'ò□□-□ä?

~*j~'ñ„Úf¯□P[^Dóé»P©!X•¹þ.□sêt½z€
*9§ŠÞ?2iR$Î7;□□úšO´Ûý£ìþ|^~3åo□±ëŽ´□-□Çø^ßÅ□x·ÄO¬j¶×ZcMþ…□n□¡RÄ¨
□·åÀç©üë¬žâ□hüË‰£‰:n‘,□ÌÐ□”Sc‘%□d□ÕÑ†C)È?□ygÁ=Niü3¬Üj7ÒH□R□Þ\JNÑµ{±â€=V
ŠlrG4k$N®•Èe9□ñ¥$($• ä“@
Ex□îî<sñb÷Ã¿ð”¾•£éö‹*=¼À,¬B□ÎàÌžø
}ëÕ¼9dÐðí†›}¬•FeÊ¥ÜçkM-%@É9À u=(□jŠŠ;›yfxcž'-?¾Šà•úŽÔ±O□Â³C*HªÅIF□□u□w
(¨üø|ÿ #ÍO;nï/pÝ•\uÅr^□ƒÄðx¯ÄrëZµ-Î-eÍœ□à´+¸•□ 6ü¸□=HÏ½ v4W
§|F¶½ø‹ªøjO±Çggn³Eyö•ûÂvdz□□è□†»„‘%□d□ÕÑ†U”ä□ê□ :Š*□ï-
f™¡Šæ□•>ò+,Ãê(□j*9§ŠÞ?2iR$Î7;□□úškÝ[Ç:À÷□,Ï÷cg□›è(□j(¨~×möŸ³ý¢/?□ò·•ß—
Z šŠòï,××w¶>"7wSNWSp¦Y
`c¶kÓ¥-8ci%uŽ5□fc€□¹ □ÑHŽ²"°0ea□Àä□LYáyž□•

¨d

7(=2;P□”T3ÞZÛ2¬÷0ÄÏ÷DŽ□Ÿ¦jj (ª×□□□£ì¹¼·…ñ□²Jª□$N¶½µ¼

mnaŸoÞò¤□•®(□z*□ï-m™V{˜bgû¢G
OÓ4ù%Ž□šYdT•FK±À□ë@□¢¸o |F·ñ

³â□□¯±ÚG¦]y0Iö•ûõÜãw8þæxõ®ç#□Ï-´ QPÁwmu»ì÷□Í·†òÜ6>¸¯3ø³}wiâ/□¥µÔÐ¤°–
$Xä*□\uê□ õ**µÛ□Ÿ³}¢/?□ò·•ß—ZóŸŽw—
V^□†KK™-Ý¯âRÑ9BF×8Èú□Ê€=2Š†Ñ‹YÀÌI&5$žüR
ËV¸6âæ□8ë□q¸~□h□z(¨c¼µšf†;˜^TûÈ®
□¨   ¨¢¸□‹^!○

ü>¾–□Ùsw‹8O¡|äû□¡¹õÅ v•Í□ÄbHdI#=□□□□□O¯*×//>□|!Ò¬t´UÔæxí¼Æ□,M
.ïƒ×□□3íéJš□‰<□ãÝ□IÕµ÷Ötýh4dÍ
£E0Ç+ŽÙ+ø□õ □T¦K4PDešDŽ5êÎÀ□øšóß^>)Õ□WÓü□ái¶kwÌ-yÔgì°õ$ú□□>¸□9□Òø£ÃŸð•x
"÷CžFžYm¶$²`□•@*Çí N(□ ÎFE□Â|!ñ□ëÿ □¬üö-
sbÆÎRz□˜Ú□ï'¿^5ÝÐ□E□P□E□P□E□P□E□P□E□P□E□P□E□P□E□P□E□P□E□P□E□P□E□P□E□P□E□P□E
□P□E□P□E□P□E□P□E□P□E□P□E□P□E□P□E□P□E□P□E□P□E□P□E□P□E□P□E□P□E□P□E□P□E□P□
E□P□E□P□E□P□E□P□E□P□E□P□E□P□E□P□E□P□E□P□E□P□E□P□E□P□E□P□E□P□E□P□E□P□W#ÿ
V¿J§W#ÿ V¿J u□Q@□□Q@□□Q@□□Q@□□Q@□□Q@□□Q@□□Q@□□Q@□□Q@□□Q@□□Q@□□Q@□□Q@□□
Q@□□Q@□□Q@□□Q@□□Q@□□Q@□□Q@□□Q@□□Q@□□Q@□□Q@□□Q@□□Q@□□Q@□□Q@

›ýSUJ·7ú¦ª” QE QE QE QE QE QE QE QE QE QE QE QE QE QE QE QE QE QE QE QE QE QE QE QE QE QE QE QE QE QE QE QE QE QE QE QE QE QE QE QE QE QE QE QE QE QE QE QE W•|=ÿ Š;â
•ˆüÿ %¥Ñûe€'ŒuÚ¾§iÁÿ ®uëõä
¸Ð5?øëOuÆ8,qÈßÉ žÃï©?íŠ 6ÿ ÂgñðŸ¿§xfª™ÿ £?ù

Ÿâ¿ù8?□ÿ ×›□íZÓø3£Mgá
5›Üµþµ;]Êì0ÅrBçÿ -oø□fx¯þN□Â?õæßûV€9¿‰þ□Ñ!ø‹áŸ.)—
û{Qo·□4□Ù’<íþïßjõM□Àö>□ðö¥§xjI,ç»V)<¬d1ÈWj¶□¡ÁÅqŸ□ÐM/\ðgˆ^
d±Ó¯‹Ü4k’¿4l□Ô…l}+^ûÆ□Ç□

|G7„Rô\A□…□ãÚÏÀ-³□’v’□CŸÂ€8}OÀŸ

ì¬®£Ö|h×□ó+□.MĐb%Çu□=z,sî+ ø}¬^jÿ □õ□¶LÓ=¤□vÉ#œ±A□á“í»-
@+€ĐuŸ†ö>□ŠÚçÂ÷z□^Ù

o□ŠO™) 8□9àg□□ŸcÖ°ÿ …|ü

ñ/□Ë^qÿ l□€2>□ü*³ñw…"Õ5íFñíÎø¬í —h…C□Ç•y-
“□Çsœñ[Á>□¼ñ^³«øcV×/□BðíÛGöhß-c-
u□Îp>B}³Æ2MzgÁOù%ºoûó□èÆ¬□„ßò>üIÿ ° ÿ Ñ³Ð□□□é-à-
•□x^Âêy4□BÌÎ‘JùØÀg?Qµ†}□5□á—ü”ÿ ˆ?õùÿ ³½-
ÿ üœ¦•ÿ `Æÿ Ðd¤øeÿ %?â□ý~□ìï@□†“à-ñ·ÅO□ÚÜê3ÛiP_¼—0Àøi›{l-œ|Üœã·\•«-
□>□üfÑ´í-êäéZÄ

²Á+îänÇäB•zòEjü2ÿ ’Ÿñ□þ¿?öw¥ñÏü–ß□ÿ °ÿ ÌÐ□¬Wœ|h×_Mð`Ò–œ
ÝbeµŒn ìÈ.yíÑOûõèõâšÖ›□Äÿ Œóé7M+hÚ□©I|¶#t‡¨□±Ü@úG@□xúÃOðž‹á□kC¼µšçÃòG

¢□W3Fy$àç□ ·gþ°□ö»+È5
□{ÛgßÄk,l?‰XdÈ×šÞ|□ðƒÙN-‰w□ËFÂ)□à□¯Ž □ÆqRü□Öå½ð„Ú-
æå¾Ñ§6ò#ýà„’ ¹ú□Ëÿ □ □>?dü<€

§P‹ÿ Az“Â?□tÝ2]3^Ô/ï®µÈØ\Ë0›äw#¡□dŽzç'ñÅ3ãçü“ë□û□Åÿ ½z]—üxÛÿ ×%þT□å¿

¿ä§üAÿ ¯Ïý•ë
Ã¾-OŒ>"Öµß□]Ý□2ÖàÛØÛC&Ð□õÇ□IÇRsõÝøeÿ %?â□ý~□ìï\ÿ †|KÂ□□ë~-
ñ□¥Òé÷□□âÎæ(÷□½3î□ÛÓ¡□} =
Á_□O□õ{ö±ÕîgÒ.Pyvsòc"<¶z-8è:óÒ¼—
á•Ã¨¼k-£q«j□+¥[]²¥œ□□ß)êÇ°ã□ŽO¨Ç>³à¿^GÆúÍòXh÷0é□ñ•—
³ðd"#+•Ààç©éÏZç¾□□Èµ¬ÿ ØMÿ ô□
~□²"Á?□5O□ÚÝÏ."= ¹Š9[%[
sõûÃÜcÒ½~òÒ□û+‹;…-□ñ´R(8Ê°Á□ú□òÔÿ "•"þÁ_ÐW¬P□ÎúGÃO

Þ|f×|14W□M³²Y¡Q1

□ˆO-ßïµm|hÒZÚ□húL†Ý¢˜ÛZ¹r

x□*□Ýr8ç-jø{þNOÅ_ö

•ÿ A·£ã□üŒ¾□ÿ °™ÿ Ð¢ □ß□ü(Ñ|!©[ê–
×W³j□ÂÑÈòH6H[©ÛŽ=†□>µ…ð□þE=[þÂoÿ %zÍx□ƒ|ckð¦ÿ ^ðç‰-/□ý-§·’□·y ñÜŽ□
Aúç□□ÒÛÿ ÉÊ]□Ø,□ê+Uü□g

¡ñ□âU•Ê–,âá¡‘AÆUš@F~†²|□â |SñÖ}^K l¢ŸO>Drƒ¹¢□B·ã×Ž>½kwá—
ü”ÿ ˆ?õùÿ ³½ r□7ÃO

Þüb×|54w'M²³Y¡Q1

□ˆº·□¾ÕïzN—k¢i6ºe’²Û[F#Œ3n8-¦¼sSñ□·€~;ëZ¶µ□È±Ô,□
’(÷n;cç¯L£□®+Øt]Z□wE³Õm£-8.â□¢Ì¡\□Ó □üè□#ÇZ-
Î½áï±Å®□-ÛÍ□yp8Ý □rç#□8ç=«Ã¼a¡x□Ãš:ßøKÅ□6¹i"2□º□□9Á ¨ □×
ãƒ]ÿ Çˆ¯ŸÃº\Š“É¤Gv□PH>ñ^6çÛïuã${WšøÃZøyuá‡³ðŸ†§K¼+=ÜˆG□¡†rK1$’□Óæë@□ÿ Æ
□Ùu?ƒz5ô˜□ÝMk3c¦Z&'õ5Vãà¥“x6ëSÔu[ë¯□□f¹7-oÉæ□Ü□□dŽ€’sßŽ”ï‰¿òB|3ÿ n_ú$×ª
êò(Ý□‹ƒÿ è³@-W□•uK_Ùò-TÜ»j¬ÆÂ;†9lî 6□¼□uõ□©tÏ□šmß†-®îu=B=zxÖá®-
@V9XnÆÞ¤□zç$Œät¬]□Ã·^$ýœžÖÊ&-î
·¹Š5□¹Vä□S´¶□sÅmi_□ôÈ<5ihúf¡>½□K□ÙR?-I@Û•Ý@'¶
□Æ□Z Ÿà□rC¢k±LûåM@«¾s¸…ää×¤ø—
E‹Ä^□Ôt‰p□ê□•XôVê-ø0□ð¯7ø□Ó6•¯5Ä~\ÇQ&DÆ6¶ÞGç^»@-aðsÄ@ø

êÃS☐*}☐I"¸☐-R1-☐úc

?à5Íø/RžËÃ~4ø›súUÛ°Ú,œ… ð=×s ÿ €V?ÄÈµ/   xßY¶Òcsoâ»eM©ÿ =
€øõbC~□×¬Ýø!□áTIá
b,Acå#tV˜|Ùö□ùüh□Â4›¯ j-O□ã-_\¹×.Yšf^|±œñƒƒž1íÛ□WQà□□Þiþ□ñ¥-
›wqwi¥ÀÓérÌ¿<jr¼ƒÓ+c Áõ©¼-
ñ□Ãž□ÐãÐ<[ImªX□□1²Wó□r3žAÁüzçšß¾ñŽŸâŸ†^/ŸÃzséqÛÛ„iî!H-`ÙÞ£□□¶î□ë-
_Z æ´?…ú

¿ðÆO□jZ¥ÛêsÁ5ËÝ4À¤n¥¸`G=9ÉÏ^•wáœ¶ž□ø=¬ø¶
v[ò®…Ý‰Y
œFqé¹ð~•æ°l~□m>□wiâ•µe□ám™□)□ue%@□
õÎ:dõ¯sÒäðç□¾□j:□…□ÛÅ□¹…mf□^)>ònäýæ□Ý‟ž{æ€<‡I°ð□©d÷þ2ÕõË□rå™¦h‡Ë□Ï□89ã-
Ý±ÅvŸ
eµñ^›â_□ÝÜ]_h□•{7□”‛cßÀÏo°§□:úÔ>□øƒáÏ

hqè--Ð$¶Õ,‰˜Ù+ùƒ9□Ï àþ=sÍzW€¼]£ø¶=B]□HžÆÞÝÑL’@±‰‰□¦Òzcõ-´□å□þ□xwÄ>%ñu-
¡□ÃC¥Þˆ-,LT…Ý ä÷áEz‡•¼-
□µ¥éö·~!:7‡ír/□8O8aB□ìp1ƒ×=G□□çš□Œ¬>□x÷Æðëö×Š×·¾}¸†
Å×t„u#¨pGãW~28‶^ð¾¡¬[^OáM»§Š□ƒ¼òCz□¸Ç ðØ4□ÊøŽ-
x3SÒ5□□ø□æ½ŠéVx□ûÃ's□□□□#□s]§Çk{‹»ï□ÛZL`¸-
òDŠPHØÄÆ□Èä`úWœøßWðV¡me□„<=%ªÅp†kÖB¹È8NIÎpNN>ïzôoŽ·□□—þ

¸´€Ü\Åzï dpc!p99<q@ø‡àž—¥xJóS³ÔõÖìák¿µ<œHè7—¶pqÎAÇ&²¼c¯Üø‟ö Ñ5
×ßvoÒ)ŸûÌžbäû•>æµ5ÿ •šf³á;-3MÓ5í«èZÔ[ò#gNäõ8ã$ãY¾5ðýÇ†~ èšmâ…°[èä
™ Ïæ6ß¨Â€:Ÿ‹þ+»ðç,4ëK
‡¶¸ÔH¦O¼‘ª,ÛHèyQŸBkÊï#ø[‹!Óµ]wûb8ËÃpÉ€Ò‘‘Ž}ò=Mz¯ÅÏßë>Òu2ÜÜÝéR,Æ
»‹FTnÂ÷ä/-™¬ÛŒ~ k–çA-
+-£}°XÆØ=ðÙ •s•¥ E7‰<A®þÎï¨[É;êþâæhóæ4Jøfõû¸Éÿ xÕox+áî¹ý•}¢k·±ë-òBÓ
…²à°(GNÙ^=Íw7?ôÝÀZo‰_E¾†ÂêQ¶Ž%%°Äd

□¼zî-µäþ.Ö<3âýWJ>□Ònàñ#]«ùÐÁä€9$°□□□ƒ»□ rh□é*ò?ŒÙ»×ü□¥1Ì□z‰ó□±ÃD£ÿ C5ëƒ
8ç-yÇ□k□ðŸˆ1˜ôíG

□û[X□è£@□□Äo□ÉãO
ÿ gÛ\,□□L·6îùÛ½A□ls,□þ•Ãx”ëÚuí—
Œül°d□èÑ²ØØÙÈÌn®XqÉè2□î@^•ì†DXŒ¥ÔF□qrx□×>•äzR7Åoˆ□-N¤ø[C•Çe□

.fà–#¸èyí´c‟@□>□ÖÛÃZ□ýÝö‛©kž/Ô#□wëk□&Î□†T99□□IP?°:ŠõÏ

ø—Nñf‡

-¦;□$%J¸ÃÆÃª°ìEy¦—®iþ□ø-ã□â;ƒi□øK›YäF+"

•«•Ï\}TŠØø!asgà››«ˆ□□ï¯¤¹□□c÷eT□üvŸÀ□@□ÿ □?Ñ|_ãí58,□K÷kØ|ò©ý□þUëuä□□3¨j
^2ñ□•Üj□—îÏ¸.çÿ F-
zå □QE □QE □QE □QE □QE □QE □QE □QE □QE □QE □QE □QE □QE □QE □QE □QE □QE
□QE □QE □QE □QE □QE □QE □QE □QE □QE □QE □QE □QE □QE □QE □QE □QE □QE □QE
□QE □QE □QE □QE □QE □QE □QE □QE □QE □QE □QE □QE □QE □r?õkôªur?õkô □QE□
QE□ QE□ QE□ QE□ QE□ QE□ QE□ QE□ QE□ QE□ QE□ QE□ QE□ QE□ QE□ QE□ QE□ QE
□ QE□ QE□ QE□ QE□ QE□ QE□ QE□ QE□ QE□ É¿Õ5T«s□ªj©@□□Q@□□Q@□□Q@□□Q@□□Q@□
□Q@□□Q@□□Q@□□Q@□□Q@□□Q@□□Q@□□Q@□□Q@□□Q@□□Q@□□Q@□□Q@□□Q@□□Q@□□Q@□□Q@□□Q@
□□Q@□□Q@□□Q@□□Q@□□Q@□□Q@□□Q@□□Q@□□Q@□□Q@□□Q@□□Q@□□Q@□□Q@□□Q@□□Q@□□Q@□□Q
@□□Q@□□Q@□□Q@□□Q@□□Q@□□Q@□□Q@□2hb¸‰¢š4’6êŽ ƒø□}□ ŠªŠ□T*□€ À□˜ÐBó$Í□4¨□G
*
.zàö©(□É□K□G"+£

□a•□
l0Eo□Š□’(ÇEE
□à*J(□□gl·&à[Â'=e□7Ç-:;xaŒÇ□1¤d’UT s×Š’Š d0Åo□Å

i□kÑ□@□ð□‘Á

O#Ç□#Hw9U ±õ>µ%□□□,□8œÄžp]¢M£p-™ëŠ□□b’I#‰□ä »*€[ê{Ô”P□i□1I$‘Ä^ò□]•@-
õ=èx!’T•âF’<ìr •Ï\-Õ%□ TqÁ

RI$qF•!Ë²¨□•¹ïRQ@□G□¼0¼•□1£Hw9U ±õ>µ%□□□ÐCq—
<I*g;]C□ÈÔ”Q@□¤□Å$’G□#ÈAvU ·Ô÷¤žÚ
¨ü»^#™3•² aù□-Š j"D□#UDQ€ª0□6(!□XC□F□‹0E□$õ'□êJ(□?"-
?Ïò“ÎÛ·ÌÚ7cÓ=qRQE F°B³´Ë□ \ Î□n v&‰ †fC,Hæ6Ü…”□§Ôz□’Š *□--
®J□íâ”¡Ê—@ÛO¶jj(□?"?Ïò“ÎÛ·ÌÚ7môÏ¥ □1I$‘Ä^ò□]•@-õ=êJ(□)ímî”-
Ä□Lªr□^□□øÔ 08□Q@□@ ,2□Pj°éöI

•¥¥°Å'ßA□□¾£□Õš(□9màž/*Xc’1□‘Ô□ÇN*B20zQE 2□b·ˆE□I□c¢"…□ð□Áil·□à[Ä'#□Pƒq üzÔÔP□qA□□ü¨’=ìY¶(□˜õ'ÔÔ”Q@□Éo

Ï□Ë

nñ•ÈÌ •>£Ò¤¢Š ,âÊÖè©¸¶†b¿tÉ□l~uÎü@ðÄþ*ðEî□c"C;„h·p„«□´ã±Çò®¦Š ñ½7Ä-
□tm.ÛGO□ÚHÖÑ,1J¯òaF□8|vìGá]/Ã/□êž-□Æ-®´CTÖg□Í□$□‹-
8Èã9sÓ#□ÍwôP□□□V·EMÅ´3□û¦HÃcó©•U□*¨U□ □ ¥¢€!-
ÖÞy□I-â‘Ðå□Ð□¿BzT□□J…$Eta,¬2□:Š ®¶6k□…m
□+n□#□AõÇ-I$□LÈÒDŽÑ¶ä, •>£ÐÔ”P□+il—□p¶ñ
Û¬□□ãøõ§M□7□ùsÄ’¦sµÔ0ü□IE □\ØY›•´□H

Ý|Ï,nüúÕŠ(□□C)V ©□ ÷¨mìím
□khaÝ÷¼¸ÂçëŠžŠ +›ñç†Ç‹<□¨é*□žé¾ÜžÒ¯+Ïl‘,}
®’Š æ<□□ý×€,,|E`Ñ\-¹µ¸,l6õ\¦O®å□?S[°n™e£ØGc§ZÅmk□vE□áFNOêjÕ□□
□ö¶÷AEÄ□J□å|Ä□ƒíšÅñ´šœ~

Õ□E´’çP’□□1Æ@`[å,3ýÐIü+~Š æ>xdøOÁV□\€
¾mÉ□ånXduÇ
Ÿök§¢Š (¢Š (¢Š (¢Š (¢Š (¢Š (¢Š (¢Š (¢Š (¢Š (¢Š (¢Š (¢Š (¢Š (¢Š (¢Š (¢Š
(¢Š (¢Š (¢Š (¢Š (¢Š (¢Š (¢Š (¢Š (¢Š (¢Š (¢Š (¢Š (¢Š (¢Š (¢Š (¢Š (¢Š (¢Š
(¢Š (¢Š (¢Š (¢Š (¢Š (¢Š (¢Š (¢Š (¢Š (¢Š (¢Š (¢Š (¢Š (¢Š *ä□ê×éTêä□ê×é@

¢Š(¢Š(¢Š(¢Š(¢Š(¢Š(¢Š(¢Š(¢Š(¢Š(¢Š(¢Š(¢Š(¢Š(¢Š(¢Š(¢Š(¢Š(¢Š(
¢Š(¢Š(¢Š(¢Š(¢Š(¢Š(¢Š(¢Š(¢Š(¢Š(□²)hÈ□j¿□þƒó«TP□_!ý□çG□þƒó«TP□_
!ý□çG□þƒó«TP□_!ý□çG□þƒó«TP□_!ý□çG□þƒó«TP□_!ý□çG□þƒó«TP□_!ý□çG□þƒó«TP□_!
ý□çG□þƒó«TP□_!ý□çG□þƒó«TP□_!ý□çG□þƒó«TP□_!ý□çG□þƒó«TP□_!ý□çG□þƒó«TP□_!ý
□çG□þƒó«TP□_!ý□çG□þƒó«TP□_!ý□çG□þƒó«TP□_!ý□çG□þƒó«TP□_!ý□çG□þƒó«TP□_!ý□
çG□þƒó«TP□_!ý□çG□þƒó«TP□_!ý□çG□þƒó«TP□_!ý□çG□þƒó«TP□_!ý□çG□þƒó«TP□_!ý□ç
G□þƒó©ås□.ãª©#5—ý«?÷#ü•øÐ□ß!ý□çG□þƒóª_Ú³ÿ r?Èÿ •Ú³ÿ r?Èÿ •]ò-
Ð~ty□è?:¥ý«?÷#ü•øÑý«?÷#ü•øÐ□ß!ý□çG□þƒóª_Ú³ÿ r?Èÿ •Ú³ÿ r?Èÿ •]ò-
Ð~ty□è?:¥ý«?÷#ü•øÑý«?÷#ü•øÐ□ß!ý□çG□þƒóª_Ú³ÿ r?Èÿ •I□¥,³¢2

pp□ YòÐ~ty□è?:µE UòÐ~ty□è?:µE UòÐ~ty□è?:µE UòÐ~ty□è?:µE Uò-
Ð~ty□è?:µE UòÐ~ty□è?:µE UòÐ~ty□è?:µE UòÐ~ty□è?:µE UòÐ~ty□è?:µE Uò-
Ð~ty□è?:µE UòÐ~ty□è?:µE UòÐ~ty□è?:µE UòÐ~ty□è?:µP]ÌÖöæE •GZ g□þƒó£È□AùÕ/íYÿ ¹-
ä□Æ´m&iíÖF □ž"□-
•þƒó£È□AùÕª(□¯□þƒó£È□AùÕª(□¯□þƒó£È□AùÕª(□¯□þƒó£È□AùÕª(□¯□þƒó£È□AùÕª(□¯•
þƒó£È□AùÕª(□¯□þƒó£È□AùÕª(□¯□þƒó£È□AùÕª(□¯□þƒó£È□AùÕª(□¯□þƒó£È□AùÕª(□¯•þ
ƒó£È□AùÕª(□¯□þƒó£È□AùÕª(□¯□þƒó£È□AùÕª(□¯□þƒó£È□AùÕª(□¯□þƒó£È□AùÕª(□¯□þƒ
ó£È□AùÕª(□¯□þƒó£È□AùÒ_]=¨B•Niç5OûV□ìGùñ
¾Cú□Î•!ý□çSBæH□Î2Ê ÅI@□|‡ô•-Cú□Î-Q@□|‡ô•-Cú□Î-Q@□|‡ô•-Cú□Î-Q@□|‡ô-
•-Cú□Î-Q@□|‡ô•-Cú□Î-Q@□|‡ô•-Cú□Î-Q@□|‡ô•-Cú□Î-Q@□|‡ô•-Cú□Î-Q@□|‡ô-
•-Cú□Î-Q@□|‡ô•-Cú□Î-Q@□|‡ô•-Cú□Î-Q@□|‡ô•-Cú□Î-Q@□|‡ô•-Cú□Î-Q@□|‡ô-
•-Cú□Î-Q@□|‡ô•-Cú□Î-Q@□|‡ô•-Cú□Î-Q@□|‡ô•-Cú□Î-Q@□|‡ô•-Cú□Î-Q@□|‡ô-
•-Cú□Î-Q@□|‡ô•-Cú□Î-Q@□|‡ô•-Cú□Î-U□ÛÙ-¤UER□Ï4□'□þƒó£È□AùÕ/íYÿ ¹-
ä□Æ•íYÿ ¹ä□Æ€.ù□è?:<‡ô•RþÕŸû'þGühþÕŸû'þGüh□ï□þƒó£È□AùÕ/íYÿ ¹ä□Æ•íYÿ ¹-
ä□Æ€.ù□è?:<‡ô•RþÕŸû'þGühþÕŸû'þGüh□ï□þƒó£È□AùÕ/íYÿ ¹ä□Æ•íYÿ ¹-
ä□Æ€.ù□è?:<‡ô•:Îá®`.à□»□UŠ «ä? üèòÐ~ujŠ «ä? üèòÐ~ujŠ «ä? üèò-
Ð~ujŠ «ä? üèòÐ~ujŠ «ä? üèòÐ~ujŠ «ä? üèòÐ~ujŠ «ä? üèòÐ~ujŠ «ä? üèò-
Ð~ujŠ «ä? üèòÐ~ujŠ «ä? üèòÐ~ujŠ «ä? üèòÐ~ujŠ «ä? üèòÐ~u^çQ-
□‡•U□_Pj/íYÿ ¹ä□Æ€.ù□è?:<‡ô-
•RþÕŸû'þGühþÕŸû'þGüh□ï□þƒó£È□AùÔvW²\ÊÊê □Ï□z€*ù□è?:<‡ô•Z¢€*ù□è?:<‡ô-
•Z¢€*ù□è?:<‡ô•Z¢€*ù□è?:<‡ô•Z¢€*ù□è?:<‡ô•Z¢€*ù□è?:<‡ô•Z¢€*ù□è?:<‡ô-
•Z¢€*ù□è?:<‡ô•Z¢€*ù□è?:<‡ô•Z¢€*ù□è?:<‡ô•Z¢€*ù□è?:<‡ô•Z¢€*ù□è?:<‡ô-
•Z¢€*ù□è?:<‡ô•Z¢€*ù□è?:<‡ô•Z¢€*ù□è?:<‡ô•Z¬Øu
d°XŠ¦ÒØÈ□€,y□è?:<‡ô•Z¢€*ù□è?:<‡ô•Z¢€*ù□è?:<‡ô•Z¢€*ù□è?:<‡ô-
•Z¢€*ù□è?:<‡ô•Z¢€*ù□è?:<‡ô•Z¢€*ù□è?:<‡ô•Z¢€*ù□è?:<‡ô•Z¢€*ù□è?:<‡ô-
•Z¢€*ù□è?:<‡ô•Z¢€*ù□è?:<‡ô•Z¢€*ù□è?:<‡ô•Z¢€*ù□è?:<‡ô•Z¢€*ù□è?:<‡ô-
•Z¢€*ù□è?:<‡ô•Z¢€*ù□è?:<‡ô-
•Z¢€*ù□è?:°€ª zŠu□ QE□ QE□ QE□ QE□ QE□ QE□ QE□ QE□ QE□ QE□ QE□ QE□ QE□
QE□ QE□ QE□ QE□ QE□ QE□ QE□ QE□ QE□ QE□ QE□ QE□ QE□ QE□ QE□ QE□ QE□ QE□
QE□ QE□ QE□ QE□ QE□ QE□ QE□ QEq_□|pþ□ðÒ\ZB³êWr^-
"nFâ2XŽ¤□AÜŠ íh¯□°Ñ>3Xé□®·Š-
å°Ž#4šzÆ§€2T□›K{□Àšô/‡^0-7ð}¾¬Ñ¤WAš□¨Ó;VUÆqžÄ□qÛ8æ€:°(¢€
(¢€
(¢€
(¢€
(¢€
(¢€
(¢€
(¢€
(¢€
(¢€#¸ÿ •i□Ü?Ê¹Úè®?ãÚ_÷□ò®v€
(¢€
(¢€
(¢€
(¢€
žÏþ?"ÿ z ©ìÿ ãò/÷¨□~Š(□Šâµ[?^□Æ±u-Ÿ«Xx□K‰¶ÛÊ--êyø□b□íQžƒ¯□_ÁÞ
ñ□x·Tõ‡‰¥·»¼´·K»{ëxü±4Lqó/@A#§¿Ô€w´W%â□|m¨ëcÐï,t}1#

×òÄ.&‛Îr□2p ã;½x¬•□\ñG‡üwgá_□^Ûj°êpÉ-†¡

□□-□ÜÈè8□sÇ·'< z%□W□ã-
□kqø□Ið¯†ÚÖ□GPŽIä¼°]ë□IÝWø˜œõãóÈ íè¯+Ö5□□ü?[][Uñ□§ˆ4-¹Ž
¨□ŠÛK□s□É³®□¯åÜz¥ □QE □QE □QE □QE □QE □QE □SRÿ •&ú•çVê¦¥ÿ -Mõ-
Î€1+sNÿ •$úŸçXu¹§□Ç’}Oó
TQE □QE □QE □QE □QE □QE □W5ão□Íáý"8ôøÄúÖ¡ µÓ-ÏñJßÄ□ÛQó□íïYß

5•cXÐ5íËÕ½¼³Õ'³3¬K□eL□Â€=h□¶¢Šá5ß□xƒUñLÞ□ð^µ†kHÒMGR°]ém¿•E_âr9çŒP□wEr:

•ã]7V•õo□Úë□{+
cm9mäSŽ6”8<ã¯jë¨ ¢Š(¢Š(¢Š(¢Š(¢Š(¢Š(¢Š(□7Wû±}Oô¬°ÔÕþì_Sý
+.€:
_øô‹ýÁST6¿ñé□û,¦ □Š(□Š(□Š(□Š(□Š(□Š+□ñ×ˆõ
□i¡x}ÐkÚ‰-□Œ¡Å¬+Ì“0<p8 õ'Û□□ÙQ\¯Ã}fÿ Ä□´□SS˜Myq□□d□□q

Ã¢€ t ÔI"Å Hì b{ @

¢¼¿IÔ<uñ□ÖMoGÖí|;¢¼Ž¶(l−æiÑX®÷Þp¹
ôÿ ë□Ÿ□ø“Y›\Õ<+âqnÚÆž‰4w6ëµ. n□ŽÄ□□õö

ÞŠ(Š(Š(Š(Š(Š(Š(Š(Š(Š(Š(Š(²uoõéþïõ-jÉÕ¿×§û¿Ö€3è¢
Š (¢Š (¢Š (¢Š (¢Š ÙÒÿ ãÐÿ ¾¥]ªZ_üz-
÷Ïô«´ QY^">[‡HøG-m®5'eHþÔûc@O.Øä€9Àä×¯ÿ ÂÉðŽ?ˆ¥ñ6Ÿ«Ch¢[9´õ…vgæØãæ8-
¸þ"êtVKê—
wž]WG³W‹=µ¼®1eC-Ýy®6óKø©„š¢x£J'î52)4ñä69Ø%'·o¨ë@-'Eax7Äiâßé Úâ
Eäýª2Z<çk«`¦å8-{Ë¨ì¬§»—>\´"…?Ê€&¢¼·D"â•ô^¼Aiâk
Òë/ke,Ü™ owïÇoÓ¥oøÄš¾®úÎ'¯Çoý«£\ˆ&žØÊÃ*À-‡'ôéÒ€;:(¢€
(¢€
(¢€
(¢€
(¢€
(¢€
(¢€0o¿ãö_¨þU^¬_Çì¿Qüª½ QE _Òåáÿ Üþ¢µë#Iÿ •‡ÿ súŠ× Š(Š(Š(Š+/_Õ
®4}8Oi¥ÝjwRH"†ÚØ

_
9Áf<"ñË-”□©Eyç…5Ï□Ïñ□UÑ¼CshR=>+¤µµŒl□™±□äncŽ¤ñž€W]â)µ¸t‡ÿ „zÖÚãRvT•íO¶4
□òíŽH□œ

M jÑ^Y¯ÿ ÂÉðŽ□?^¥ñ6Ÿ«Ch¢[□9´õ…vgæØãæ8-¸þ•é:eôzž•g¨D¥c°•&Un 2†□üè□Õ□OUšú
ßJ°—LµK«åŒ˜ y□+¿`Iè+f¼Òþ*Aa&¨ž(Ò¤»□L‡JM<y□Žv
IßíÛê:Ð□¤QX^□ñ□x·Â:v¸'y?jŒ-•9ÚêÅX□é¹N+v€
(¢€
(¢€
(¢€
(¢€
(¢€
(¢€
Âµÿ •,□¾kv°-□ä
Ÿïš Ý¢Š(¢Š(¢Š(¢Š(¢Š(¢Š(¢Šæ¼N<]sskeá¦±³□ÕšçQ°-a‹'…Hûž§'Š éh¯3:¿Œ|□
âm□ÓÄZµ¶»¤j÷Kd·+h¶òÁ3}Áµx*□‡§□L □ŠÂñCø"Ú[Áá^¬…ÌÒm-
æñŽËtÇÞÚ9cØ□Î¸}oQñßÃõ´Ö5]~Ó_Ò□æ8náû
ÛÉ□¹Æä+×□×òô õZ(¢€
(¢€
(¢€
(¢€
(¢€
(¢€
(¢€
(¢€
(¢€
(¢€
(¢€
(¢€
(¢€
(¢€
(¢€
(¢€
(¢€
(¢€
(¢€
(¢€
(¢€
(¢€
(¢€
(¢€
(¢€
(¢€
(¢€
(¢€
(¢€
(¢€
(¢€
(¢€
(¢€
(¢€
(¢€
(¢€
(¢€
(¢€
(¢€
(¢€
(¢€
(¢€
(¢€
(¢€
(¢€

(¢€
ñïŒ?Ä□†ñÈ?tu>Aè□{

□Jö□óŸŒ>□Ô|K Ù_èŠ[WÒgûD
§

Êq¸/ûYU?ð□u4□èÝkÇ□gŒ□

kH¿ê□¢Û?ï'ý1Un~.x³RÒ_J±ð.©

½‚~Q‟cì‰ˆÁp
äzàœ

äâ»¯…þ□—Á^
ƒN°*o¦‘®n¶œ•#``} Qõ□€;*(¢€
(¢€
(¢€
(¢€
(¢€
(¢€
(¢€
(¢€
(¢€
(¢€#¸ÿ •i□Ü?Ê¹Úè®?ãÚ_÷□ò®v€
(¢€
(¢€
(¢€
(¢€
žÏþ?"ÿ z ©ìÿ ãò/÷¨□~Š(

cÅðœ›˜ÿ á□>□ÞWï?´¼ï3~OÝÙÆ1Ž½ë”ø□$.Ÿã]NÃÅV×□øÂú!1¹y□á¸□x

Ð□□ýÜgŒ’qÆ-×Ž<E¢jwv°¿,uK»e™þËw£
¸□Å"·rd□lc<õÏ□WD³ÖüYñ□×Åšž•6•§i¶²AeotGŸ3¿

ì£îŒqƒ@-ŒÌ□K1□@É' ¯:Ñ   ñ¿Ä□øJ¡□û□FŠK=:_ùú™¸-
Eõ@>P{þu›ñ□\ÕîüB¾□"Ãþ$—Ã^Š÷³é6M+Þd□å□È
œá^9<Ž+oEñí¹šÃH´ð'‹¬-Ë‰¼m."åÃ
' ,w|ª;ŸJ ïkÎ~&¥•Åæ•□ƒ_□Âa□y4¯ìð¦D□□É»å□v;¿

õÔêúíþ•â□□N¶Ðî¯m¯ÙÖ{È‰Ùh□

□àõÏr:w®SÄv°ï†þ#□□éÚ<ÚÕ…Îž,®mí^óáÃî

Š~ð<p=ý¨□ž¹¹×ìµý2çâ¤[´˜$•-fÓðl£¸ì×□ïd-‡îƒÐc5í ,□□
ô"¼£ÄŰö»ñ□A¸ðÞ□àÝfËíÛcšóX€[Ç□n□°□%□□«ÓìmVÇO¶´V.°D±□n¤(□'ò
□QE □QE □QE □QE □QE □QE □SRÿ •&ú•çVê¦¥ÿ -MõÎ€1+sNÿ •$úŸçXu¹§□Ç’}Oó
TQE □QE □QE □QE □QE □É¦ŽÞ 'šEŽ(Ô»»□□P2I>”ú¯□cm©é×6□‘ù¶·143G’7#

'Èàö

'ÁÐËâ½~□□ÞÆËlU-´H\cË·ÏÍ6;4‡ÿ □Àä□O„ßò
ñýŒ7¿ú□¤ÿ …%ðóþ…ïü•¸ÿ ã•“ð·áÎ•¤]_ê÷:$ö°•®©u□”“<ªE¿Ý\)8`T¶□ƒŸZ õjñ□
ZøÇV×üiý‰«iúU·öõÂKu-·Ÿ9+€ª□□»Bã¯<šô½;]¿½ñV«¤M¡Ý[YÙ,m

¡!>]É`
Æ8ö'§8®6S-|>ñ®µ□b _ë:□µ"Ý□§ ’kyñ‡Êq•Ýsô÷ mõŸ□xCÆ□>‘â]N×ZÓu‡h
½Kao,2□□□W,□Oÿ V□¥Wš[Å-øûÆZ>«{¢]è°□Œí<Qß ·□3‘…%□vªõÿ õñét QE□ QE□ QE□ Q
E□ QE□ QE□ QE□□›«ýØ¾§úV]jjÿ v/©þ•—
@□□¯üzEþà©ª□_øô‹ýÁSP□E□P□E□P□E□P□E□P□E□P□-
_U³Ðô‹-RþQ□-¬fI□Øv-¤ô□¹5ÈøCJ¼žËSñvµ□M_X˜”‰¿åÒÔ□åÄ=

>fõ'ž•Õëz-•â="}+V¶ûE”û|È·²nÚÁ‡*A□□Þ¸»□,□□ÖÚS□‡□˜□•ÅåÁç□□□ \ø=ÿ $Ÿ@ÿ ®R□
è×®ÎêÝ.í&¶|„-6•±×□b¼ëàÿ •´ÿ □øbÃW—
IšË_¸·x®Úf‘_o˜H□□áxUì?Zë|;®ßëSê±ÞèwZZÙÝ´□¼äâå□I□
p}²=Í p>□×5ÿ ‡°,~□Ö<!-ê+dÎ-×°L□xæŒ±`O#iç½.□©êZ×ÇU½½Ñ'Ò?âŸeXg‘ZG□Ï□f
÷IbF□<VÓ|HÕc-o'Ãß□ý¸□□Ç□¼□ÿ ×lã-ø«Þ

ÐµdÔõ/□x‘"‹WÔ,F–±>õ´□>ì{»’NI□f€;*(¢€
(¢€
(¢€
(¢€
(¢€
(¢€
(¢€
(¢€
(¢€
(¢€
(¢€
(¢€
ÉÕ¿×§û¿Öµ«'Vÿ ^Ÿîÿ Z Ï¢Š(¢Š(¢Š(¢Š(¢Š(□gKÿ •Cþùþ•v©i□ñè□ß?Ò®Ð□7□ìû□ÿ
cò¾Õå·"çgfü|»±Î3Œâ¼‡Æ6ÿ □gÒØø¢×M¼ðìx"P¶ðü¯□ÒF§'&Q’¼r□,}EzGŠµ□wKÓb»Ðt˜õ
YRaö‹V—Ëv‹□%   ãp;zçŒ×!¨øó_ÖtÉôÝ□À~ ‹R¸•¢□j□,6ñ□□Ü\Ÿ˜

ç□f€;½
P°Õ´□□í,ƒa4*Ð`c
Ž□;c¦;b°ü{â7Ñôq§éÉö□wTÍ¶Ÿl¿x¹□.}□AÉ=8¨¬,î¾-|.†ÖÒÊmZóNµÀ·¶RÍ4¬Ù;@□Ææ'¦p+
†ð¿‰åÓo'×5Ï□xÛPñ□ÚíšátcåÀ□¢„□ùP~dòh□Óü!áôð¯„´Ý□□IöHB»ŽŒä-
b=‹□Z×2A□¬²\²,□…¤i□
□

IÏlV_‡ü@|A¥Ë|4}[N1ÈÑ‹}JÛÈ•°□È\ž

qŸPk□>¥ñ□á¶¥□Þ•q ÞßC5°ÛÝ□¹OEcòƒƒôéž´□ÀéPøÆ
[÷ød$ÿ „aÃ5¬z Pw□ù□®îvúýœuêk·øcs¢¶"{ic□ì:´W□õhµøú3·VsÐƒŽ□ã□ëY:•õ¿

è-z6³à/□½å”)n$Óm-â□□ ¡ƒ□ È□9--
□ézÍç‹µ¿□k□qÒ•üQ[ÛX³†□FŸÇ&8□ñÇQÏâ□ßÑE□ QE□ QE□ QE□ QE□ QE□ QE□□ƒ}ÿ -
²ýGòªõbûþ?eú•åUè ¢Š(□þ“ÿ □þçõ□¯Y□Oü|?ûŸÔV½ □QE □QE □QE □QY^!×?áÓ>Ûý—
©ê_8O#M·ó¥ç<íÈàc- rz_ü—}{þÀöÿ ú□ww□ìû□ÿ cò¾Õå·“çgfü|»±Î3Œâ¼rÏÅ÷-
ÿ □µ?□?□üblî¬"¶D□Ió□+d’7c•z&³®k±ørÇWÐt
/%‵’K□>é¼‰Ö"¤□2p$□nAÏz à<coñ□}-•Š-tÛÏ

Ç‰5
o□ÊñÍ$jrre□+Ç!H'ÔWªhZ…†- Øßid□ ¡Vƒ□□\p1Û□1Û□Âj><×õ•2}7Fðˆ"Ô®#h„š„
□¼E†7□'æ□9Ç□®‹DÓ'ð'Ãx,`·}JëM³fòaÎg‶-*¼□Ë□□□ö

¦¹?-ø•ô}□iúr}£]Õ3m§Û/Þ.F
ŸEPrON*yüI©Gà□â□ðõéÔM¸•i*¥¥

N□□□xÎO□Æx¯:ð¿‰åÓo'×5Ï□xÛPñ□Úíšátcå À□¢„□ùP~dòh□Óü!áôð¯„´Ý□□IöHB»ŽŒä-
b=‹□[u'áÍ□þ□-9ï?²u]3l¦?'S¶òd8 î
"òóŒúƒZô QE□ QE□ QE□ QE□ QE□ QE□ V□¯ü„□ýó[µ…kÿ !□ÿ |Ð□í□Q@□□Q@□□Q@□□Q@□
□Q@□□Q@□□Wœ|Nñ□½i5ž‹¥i:ä-
wi°÷PÒ¬ÌòÇ-Hòãè□œu'€r□ □ýÿ á<ø‹§[Y|ú/†n>Ówr>ì·`|')îW©úãÒ½-¼ÇEñÕ†ƒ¥ÛézgÃ
¿□[ÚB6¢□#ó$ïäž¤šì|O®ßèPXÉa¡]jÍsv□H-äƒ
6s!À<

{

zŠ ¿¬□j□dÜ□b}•ûKhò>Û»ÉÎFwmù±Œô¯$ñCx°ÒîÇRø…iiyá«9Òi□Cr#Ž@p¯*H7°,z□□®zW¢x
¯Yñ

†öw:F€u‹/œ^E

›gN›Y□á‡ÞÈëÓ¥qþ"ñ□ˆ<y¢\xsFð~³§›à!¸½Ö!□G□düÄ

’ç□q@−¥

ÑÜÁ□ð¸x¤Pèêxe# Š}UÓlcÓ4«=>&-□¬
"Ô…P£ùUª (¢Š (¢Š (¢Š (¢Š (¢Š (¢Š (¢Š (¢Š (¢Š (¢Š (¢Š (¢Š (¢Š (¢Š (¢Š (¢
Š (¢Š (¢Š (¢Š (¢Š (¢Š (¢Š (¢Š (¢Š (¢Š (¢Š (¢Š (¢Š (¢Š (¢Š (¢Š (¢Š (¢Š (
¢Š (¢Š (¢Š (¢Š (¢Š (¢Š (¢Š (¢Š (¢Š (¢Š (¢Š (¢Š (¢Š (¢Š (¢Š (¢Š (¢Š (¢Š
(¢Š (¢Š (¢Š (¢Š (¢Š (¢Š Žã›i□Ü?Ê¹ý□ýÆü«¤¢€9½□ýÆü¨Øÿ ÜoÊºJ(□›Øÿ ÜoÊ••ýÆü
«¤¢€9½•ýÆü¨Øÿ ÜoÊºJ(□›Øÿ ÜoÊ□□ýÆü«¤¢€9½□ýÆüªkDaw□*~÷¥oQ@□□Q@□□Q@□□Q@□□Q
@□□Q@□□Q@□□Q@□□Q@□□Q@□□Q@□□Q@□UÔA6l ÉÈ«TP□7±ÿ ¸ß•mià‹$□`äÿ :µE □QE □QE
□QE □QE □QE □QE □QE □QE □QE □QE □QE □QE □QE □QE □QE □QE gjÊYbÀ'"Óð¬Í•ýÆ
ü«¤¢€"µ□µˆîŠ–
Š(¢Š(¢Š(¢Š(¢Š(¢Š(¢Š(¢Š(¢Š(¢Š(¢Š(¢Š(¢Š(¢Š(¢Š(¢Š(¢Š(¢Š(
¢Š(¢Š(¢Š(¬-UXÎ˜R~^ÃÞµh

ocÿ q¿*6?÷□ò®’Š æö?÷□ò£cÿ q¿*é(

ocÿ q¿*6?÷□ò®’Š æö?÷□ò£cÿ q¿*é(

ocÿ q¿*6?÷□ò®'Š ¥¦□-H
•˜õü*í□P□E□P□E□P□E□P□E□P□E□P□E□P□E□P□E□P□E□P□E□P□E□P□□ò1¼□…$gÓÚ«ì□î7å]%
□□Íì□î7åFÇþã~UÒQ@□:Z°¸|©-
/qïZÔQ@□□Q@□□Q@□□Q@□□Q@□□Q@□□Q@□□Q@□□Q@□□Q@□□Q@□□Q@□□Q@□□Q@□□Q@□□Q@□b[#
ô%N7-Õ·E □QE □QE □QE □QE □QE □QE □QE □QE □QE □QE □QE □QE □QE □QE □QE □Q
E □QE □QE □QE □QE □QE □QE □QE □QE □QE □QE □QE □QE □QE □QE □QE □QE □QE □
QE □QE □QE □QE □QE □QE □QE □QE □QE □QE □QE □QE □QE □QE □QE □QE □QE □QE
□QE □QE □QE □QE □QE □QE □QE □QE □QE □QE □QE □QE □QE □QE □QE □QE □)¾b□}□
:mÇü{KþáþUÎÐ□Iæ'÷×ó :"€ÀŸ-sufÃþ?£ü□` □Ú(¢€
(¢€
(¢€
(¢€
(¢€
(¢€
(¢€
(¢€
(¢€
(¢€
(¢€
(¢€
(¢€
(¢€
(¢€
B@□$□ïKU5/øòo¨þt□gÌOï¯çG˜Ÿß_Î¹º(□¤ó□ûëùÒ,□È
ý+š-□/þ=?àF€.ÑE□ QE□ QE□ QE□ QE□ QE□ QE□ QE□ QE□ QE□ QE□ QE□ QE□ QE□ QE
□ …,õ }M'˜Ÿß_Î³õ□»□Ôÿ JË

“ÌOï¯çJ□7B□Ò¹ª×Ò□ãÝÿ ßþ”□~Š(□Š(□Š(□Š(□Š(□Š(□Š(□Š(□Š(□Š(□Š(□Š
(□Š(□Š(□Š(□Š(□Š(□Š(□Š(□Š(□•²¯V□êik'Vÿ ^Ÿîÿ Z Ôó□ûëùÑæ'÷×ó®nŠ é
<Äþúþt¡ƒt
ý+š-m'ýDŸï□J Ð¢Š(¢Š(¢Š(¢Š(¢Š(¢Š(¢Š(¢Š(¢Š(¢Š(¢Š(¢Š(¢Š(¢Š(¢
Š(□¥Ô□□ ýhó□ûëùÖ□÷ü~ËõÊ«Ð□L

FE□□§üzEþè©¨ ¢Š(¢Š(¢Š(¢Š(¢Š(¢Š(¢Š(¢Š(¢Š(¢Š(¢Š(¢Š(¢Š(¢Š(¢Š
(¦ù‰ýõüéÕÍ¿úÆúš èÃ+-□¡¥¬□+þ>_ýÏê+^€
(¢€
(¢€
(¢€
(¢€
(¢€
(¢€
(¢€
(¢€
(¢€
(¢€
(¢€
(¢€
(¢€
(¢€
(¢€
(¢€
(¢€
(¢€
(¢€
(¢€
(¢€
(¢€
(¢€
(¢€
(¢€
(¢€
(¢€
(¢€
(¢€
(¢€
(¢€
(¢€
(¢€
(¢€
(¢€
(¢€
(¢€
(¢€
(¢€
(¢€
(¢€
(¢€
(¢€
(¢€
(¢€
(¢€
(¢€
(¢€
(¢€
(¢€
(¢€
(¢€
(¢€
(¢€
(¬o□h□Äþ-¸Ò>ßsb—
□D’Ûœ9@Aeú□‘ø÷é@□ðüDðÅ×‹bðÍ®¢·□”›†ØT°□ªX©qÆp□òë]MxOü#:W„ÿ hO□iš=·“n°k»d

å¤r.2Ì{“•ùzW»P□E□P□E□P□E□P□E□P□E□P□E□P□E□P□E□P□E□P□E□P□w-
ñí/û‡ùW;]□Çü{KþáþUÎÐ□Vl?ãú?Çù□-Vl?ãú?Çù□ Ý¢Š(¢Š(¢Š(¢Š(¢Š(¬□□x¯Bð-°
O-êpY¤™Ø□’ÏŽ¸Q’□□[□å¾!{□

üY_□øŠÑ¥Ñn,□ÖÚøÄdK□Cd†□;wgï{ýh□®ð÷•¼-
â«†·Ñu˜.gQ»Ê*Ñ¹□ÈW ‘ô®’¼·Q}?Æÿ □|=uá^’q¤\™ïµ^S□lÇú•ÿ òÐ¶z

às^¥@□]][ÙZÉsu<p[Ä»¤-V
ª=I<
ãáø¹à9ïÖÊ?□Û™™¶ŒÇ B□ß+·õ¬/ˆ°Ž"yã}□Ãþ
¾†×B··}Rõ&"jÜ□Û"Cê7díç5³¥üAøu-K□‹ e©iï¸„ŠÚKv•   ì□z…'Ð
 îh¢Š (¢Š (¢Š (¢Š (¢Š (¢Š (¢Š *¦¥ÿ -MõÎ-ÕMKþ<›ê?• bQE□ VÎ—
ÿ -Ÿð#XÕ³¥ÿ Ç§ü□Ð□Ú(¢€
(¢€
(¢€
(¢€
Ê×üK£x^Ãí°Ö¡□œ□áKä³EQ’Çè□j×š|Dð—™gàï□ë’
          o59íœ-ÅÓï0!<G□èŠ=¹=É □G·¸ŠîÚ+˜-
|2 ‘□□Ê‘□yö¬_□xÓÃž□□ÿ njÐZ4ƒ)□Ë»□Pª   Ç¾*ß‡?äWÒ□ëÊ-
ý W□ðÆÒ□rÿ Ä>0½‰f¿°Ôå··’A"□¼xTUÏO|uÀ

¿ÃÞ/Ð<W

’hz¤□,?¾«•uôÊ°□□Â¶ëÌüee□‡þ$ø?Ä6□‰¼××‡L¼ò×h•d.ìu
ŒçØzW¦P□?ˆ<W¡xVÝ'Öõ8,ÒLì

IgÇ\(É?€ª~-ñ÷…¼UpÖú.³□Ìê7yEZ7#¹
à□>•Èø…íü3ñe|Kâ+F—
E¸°K[kã□‘,e□’□`íÝŸ½ïõ£Q}?Æÿ □|=uá^’q¤\™ïµ^S□lÇú•ÿ òÐ¶z

à-
s@-¥E□P□E□P□E□P□E□P□E□P□E□P□E□P□n¯÷búŸéYu©«ýØ¾§úV] □¯¤ÿ Ç»ÿ ¿ý+"µôŸø÷□+
ÿ ¥ _¢Š(¢Š(¢Š(¢Š(¢Š(®CUø¥à•□ýìoüAn—(Û]#G—iî   E -
c]=ô2Üi÷0ÛËåM$L'ÉýÆ €□□^Uà-
□øoÁ¾□>-ñ□Qh°Å□oµÅs□7_1Ä^Øýîxé"ÇLb€=CJÕôýsOŽÿ K¼†îÖO»,M¸g¸ö>Õv¸□†:<öOâ□
XXI¦iúµïŸea"lhã□□Ê□□n»{ +¿

_□x·@ð´)&·ª[Ù‡ûŠä—op£,□□PxsÇ-□ñkË-‡ªÅw$K¹ã□ÈÀg□ÚÀ□{×™øWÆ-
†ëPñ□Š5KOímBêAl’)™ím‘Š"ª¨%3,s□œ×§øwÄ^□ñA“PÐ¯-.åA²I#M²¨<€À€À□wô □ê(¢€
(¢€
(¢€
(¢€
(¢€
(¢€
(¢€
(¢€
(¢€
(¢€
(¢€
(¢€
ÉÕ¿×§û¿Öµ«'Vÿ ^Ÿîÿ Z Ï¢Š(-m'ýDŸi□JÉ-m'ýDŸi□J Ð¢Š(¢Š(¢Š(¢Š(¬eñƒƒ'‰G
‡cÔá“VØÎm£Ë□□®â□□û□□\Õôá«isØ□»«U˜□i-dÙ"Œ,v·lŽ3èkÎÎ‡¦ø□ã□…,4«8ím—
Mº;PrÇŒ-'->çš ôù$H£i$uHÐ□fc€ u$×□~,ø□u□±□ZyÛ¶î□Ìyÿ ®˜Ùøæº•KN´ÕôéôûøDÖ·
²XÉ 2úqÍekšV•màÛûKË
Hô^m]ž□□ª"…' □Á□ˆç4□ºŽ²"º0da•e9□zŠuq□ □î?…~-
[ÒþoÙÉ]ý|²ìcü6mÇµv”□□®ø³AðÓÛ¦¯©Ãk%Ë„†3-w$ã…PN3ß□-šò^Þ□Ó<?àãu□-
çQ¹ÔíMÅýÛù“ËûÁÁnÊ;(Àã¥zõ □QE □QE □QE □QE □QE □QE □QE `ß□Çì¿Qüª½X¾ÿ •Ù~£
ùUz è-?ãÒ/÷EMPÚ□Ç¤_îŠš€
(¢€
(¢€
(¢€
(¢€
(¢€2<Gâ•□ÂZr_ë—¢ÒÕå□«ùnùr □
è§·jæ øÍà
›ˆ ‡_Ý,®□□ìsŒ’p□Üªß□/í´«_ ê□²ùV¶Þ"¶-
i6“µBHIÀÉ<zV¾•ñ?ÁzýêYéÚý»ÜHv¤r£Â\ú□ê2}… uÕ□ÍÌ□vÒ\ÜÍ□0D¥¤’F
ª□RIà
-¼ßâéIâð¶™xå4›íj□¯yÂºrB1ô'ŸÃÚ€5m¾,ø□ïP[□¼Gmç³m□ÑÑ
ÿ |¨_Ö»:ÅÖ¼/¤ë>□ŸDžÊÝlÞ#□j±€"8á—û¤u□ô¬?„z¤ú·Ã□□k§/<HöìÄäŸ-
Ù□þ:□ vÔQE □QE □QE □QE □QE □QE □QE □Í¿úÆúšé+›□ð•õ4□wJÿ •—
ÿ súŠ×¬□+þ>_ýÏê+^€
(¢€
(¢€
(¢€
(¢€
(¢€
(®□Æ>$-□Ð^ê(¾Ñ□-½□¨ë<ïÂ/Ó¹ö□€%-/ÐOŠ¿á□□Š□gfól□‰□nî[□AÇ8Îkn¼{OðÙð×Ä¯□Cq
/Ú5+˜u
›û“ÖiÙ□±ú

ƒØW°Ð□;¬hÎì□Td±8 z×□?Åß□[^›I<Ild□□HätÏûê¥□ŽÎø¯¨[²è>□¼¾[+□^í•ôí'–
>Í□ïuÝÛvT□ûêM7â'Ã□R=□ÇTÓaƒˆÖ³²CéË□
ø"@□ü3Es□sÁ"É□Š□□

C)□□-ã□údI□p¢B¨±*€Šƒ
□`1ÚŸ@□□Q@□□Q@□□Q@□□Q@□□Q@□□Q@□□Q@□□Q@□□Q@□□Q@□□Q@□□Q@□□Q@□□Q@□□Q@□□Q@□
□Q@□□Q@□□Q@□□Q@□□Q@□□Q@□□Q@□□Q@□□Q@□□Q@□□Q@□□Q@□□Q@□□Q@□□Q@□□Q@□□Q@□□Q@
□□Q@□□Q@□□Q@□□Q@□□Q@□□Q@□□Q@□□Q@□□Q@□□Q@□□Q@□□Q@□□V?Š4øI|=s¤ý¾æÇÏÛûûfÃ®□-
×□ □8ñ'üœç„ÿ ì□ÿ Êâ½~¼iÿ gëi.’éü[¬5Â□©)Áu□ð

r:ŸÎ½Â-□ÿ „OB]3ûNóQÄ□'□tÛ˜g°ô-
âh□zŠ(□Š(□Š(□Š(□Š(□Š(□Š(□Š(□Š(□Š(□î?ãÚ_÷□ò®v°+•øö—
ýÃü«• □¬Ø□Çô□•ò5Z¬Ø□Çô□□ò4□»E□P□E□P□E□P□E□P□E□P□E□P□^hˆž=ø…â□#YžOì□□Â'éhå□á
•w□%Ç.□□□§OÇÒë"ñÃÍ□Äzšj¯%öŸª*lûv□p`"¯`HÈ?-
h□•ñf"mðâ÷L×ü.Ícö½B+[-*6ýÅÚ¹ÁÚ•□Æ8+ÿ ëõjã4□†z6™«ÁªÝÞj°Íý¹Ì□ê×†s□õQ€3ïŠÖ
Õü)a-xƒFÖ®f°K•%•àH¤
ŒX w

sÓ±• qÞ ²°-t□õ[X&,óH’Ú×í□□Dé!~3Æí-ÇÖ®üeµÒ□Ã=JKØ¡□Ä©ö7Ú7¬»†Ð□ó×§l×Mâ□
é>.Ó’ÏU□˜FâHe‰ÊI
ÿ yXt?¥aé¿
t[=JÞþÿ PÖ5¹í[u°Õ¯Lë
v*¸□?\Ð□U¤}§û□Ãí¹û_Ùãó³×~Ñ»õÍ]¢Š (¢Š (¢Š (¢Š (¢Š (¢Š (¢Š *¦¥ÿ -Mõ-
Î-ÕMKþ<›ê?• bQE□ VÎ—ÿ -Ÿð#XÕ³¥ÿ Ç§ü□Ð□Ú(¢€
(¢€
(¢€
(¢€
ä~)□É0ñ□ýy·ó□×W ®ü/¶ñ□Íì—~+ñR[Ý±2YÅ¨□n□þ□…□Ûí@□G‡?äWÒ□èÊ-
ý W□ð,UµÓµý□B□óLÕî□ã=v1Ê·Ðó□¥nxcÀ±ø^óÏ‹Äž"Ô#□ù+m¨_y° ã□WhÁ□Àö&™â-
‡:6¿«•an5□/T)±ï4Ë“□’(è□àƒõÆzzP□?□¤MOÇž□Ð !îcÔ?´¥Qÿ ,ã‰I□½□9□é^•\×†|□£øV
{‹»_´Ýj□#□ß^ÌfžAèXöàt□ ô«□áK <k□ŠŒ×_oŽÐÚ□ÄƒÊÙ’s·□Ï>¸ö

E□<{ñ
Ä□F³<ŸÙ:!…#ÒÑÊ-Ã:î2KŽ\□□□NŸ•O□i6ß

/tÍ□ÂìÖ?kÔ"µºÒ£oÜ]«œ□©Ñ\c,¿þ¾«Ä□□4_□êiª¼—Ú~¨©³íÚuÁ,R½•#
þY¨4□†z6™«ÁªÝÞjºÍý¹Ì□ê×†s□õQ€3ïŠ ìè¢Š (¢Š (¢Š (¢Š (¢Š (¢Š ÍÕþì_Sý+.
µ5□»□Ôÿ JË □µôŸø÷□+ÿ ¥dV¾"ÿ -ïþÿ ô
ôQE □QE □QE □QE □QE cø¯X—Ãþ□Õux"□Ëgjò¢-…€ã>Þ¾ÕÉxwÀš□‰<3k«øˆ□ój□•²K-
ÝÃ"³p□² □□€N>\□ô)¡Žâ□!š5’)□££Œ†R0A□Åp?ð¨t{wuÒµÏ□i□®Å□¦Ÿ©4qs×,
ýh□ž□¹¼Ò¼k¯ø9µ µ□;NŠ□íf•÷É qþ¥›¾;g°¯D¬O

øOGð•”¶ÚU»!™üÉæ‘ËË3z³-OòäÓ|;áK

Ïª Íe5Ô□©ÝµÜÞ|□,»u
€0>¹>ô□Æü□°Ó-<#=¨´ ·MZÂú{[×òÀ”°s•Ç®0@Jt¶ö¶ÿ ´□—öJG□¯¤HÚ¢Â □nù
ãø³ ·¯8Åmk_

ô}[Z—X¶¾Õ´{ùÀ□□iWf□6?¼0Gò-O

x7Gð”S•:)^âå·\]ÜHdšcêÌ□•À □ú(¢€
(¢€
(¢€
(¢€
(¢€
(¢€
(¢€
(¢€
(¢€
(¢€
(¢€
(¢€
ÉÕ¿×§û¿Öµ«'Vÿ ^Ÿîÿ Z Ï¢Š(-m'ýDŸï□JÉ-m'ýDŸï□J Ð¢Š(¢Š(¢Š(¢Š(¯>Ö¿ä¸ø_
þÁ·UÚjúwö¶•qaöÛË/9vý¢Ê_.hùÎU°px®□þ

ÙË}□ôž2ñ‹ÞB¥#¸mL□□OP□f@>”□èìÊŠY^U□$“€□yÅÜ’üR¿û□¡xü□k.n®‡Úr)ÿ W-
ý2□rÝÈÀõ®’_□ÛÜø&o
Ýjú½Í¼ÊRKÉîCÜ°-»□Êàÿ w§N+Ÿ·øG□¥¼vöÞ7ñ¬0D¡cŠ=T*¢Ž€ ˜□€=
8Ò(Ö8ÑQ□□UQ€ è §V7‡ü<4□.[□«êÚ˜’Fs>¥sçJ¹ m□••ÆqêM7Â~□±ðv„šFŸ-
Ì¶é#H□åÃ>Xäò ¥ s?□?äHƒþÂV¿ú0W ×•j_□,õ`É}âÿ □Ï
Iæ^dÔƒ"°9□)CŒvô®“Ã>□ÿ „i®Oöþ»ªùáF5[Ï<GŒýÎ□3ž~,€:□(¢€
(¢€
(¢€
(¢€
(¢€
(¢€
(¢€0o¿ãö_¨þU^¬_□Çì¿Qüª½ t□Ÿñé□û¢¦¨m?ãÒ/÷EM@□□Q@□□Q@□□Q@□□Q@□□Q@□Ê®0Ê□□ž
Fkƒø½§èÓ|:Õ®µ(`□A kY™@u›ø□·\"□ÇlÖ×‰ü□§øª[Yînµ
;«PÂ□‹
"
¨lddqØv¬«□…š-¾£□ö¥□ë³[¶ø?µïLë□v!p□çš é|8×•á•%µ□ßm6P›□Ý|Íƒv}óš5í□Lñ6‘.—
«Z-Å¤¸%I ,:□G
•Z‹Å-□³ño‡î4[ùn"¶œ©f·p®6°a,A□G¥G-øVÇ]Ðíô«™ï"ŽÙ‘¡žÚàÇ*2©PÁ‡|□×Ö€8□oáï‡<;
Ý_ë^#ñ%Ö“k□ccuª7’Øè□@□ç€□{×Cð¯G›Bøk£YÜÆc□£iÝ□ÁS#□ ŽÄ□□ðªö?
ôH/à¼Ô¯õ□v[vß□ÕïLé□z…À□ñÍw4 QE□ QE□ QE□ QE□ QE□ QE□ QE□ W6ÿ ë□êk¤®mÿ Ö7
ÔÐ□Ý+þ>_ýÏê+^²4¯øù□?¨-z (¢Š (¢Š (¢Š (¢Š (¢Š +Ë¼C□Œ×âgöÄ-□þÚÓ¬
□iŸñ3†ÝQÝG™!VÉ-Õz

□ïšõ□(□Ä5¯□xÎ_‰¾□°ŸÀžMü0]‹{/íx[í Üw□…Ú9Áë^Ïc-
Äú}´×vße¹’%imüÀþS‘’»‡□ƒ‘‘×□B÷Ã–wÞ&Òõé^qw¦Ç,pª°□D€□Ü1“ÓŒ□PøŸÂ-
-,,Æ□ù®£[+´»•ìò□%×8□ ñÏ±÷

?âu-Ÿü&^□¾Õ †m2;é-¦□¨h÷J‹ ³p<uLóé]O•l´cà]Z=N
e°ŠÒCó □□S´¯£g□Ç9ÅikÚ

›â]"m/V¶[‹Iq•$,□èA□,=k’ƒá

†$„j□¦»«Z@Á¢²Ô/Ì•)□>@□□ĐĐ□§Ã/µÿ Â´ð÷ÛwyßcO½×gð□ã»k«¤UTPª P0 □□K@□□Q@□□Q
@□□Q@□□Q@□□Q@□□Q@□□Q@□□Q@□□Q@□□Q@□□Q@□□Q@□□Q@□□Q@□□Q@□□Q@□□Q@□□Q@□□Q@□□
Q@□□Q@□□Q@□□Q@□□Q@□□Q@□□Q@□□Q@□□Q@□□Q@□□Q@□□Q@□□Q@□□Q@□□Q@□□Q@□□Q@□□Q@□
□Q@□□Q@□□Q@□□Q@□□Q@□□Q@□□Q@□□Q@□□Q@□□Q@□□Q@□□Q@□□Q@□□Q@□□Q@□□Q@□□Q@□□Q@
□□Q@□□Q@□'<È•3•ÊFk;û
ÿ Ïoüwÿ ¯ZtP□göAÿ žßøïÿ ^¤·ÓŒ□¬žní½¶Õú(¢Š(¢Š(¢Š(¢Š(¢Š(¢Š(¢Š(¢Š(
 ¢Š(¢Š(¢Š(¢Š(¢Š(¢Š(¢Š(¨n`ûD□=ÛrG8ÍME f□d-
ùíÿ Žÿ õèþÈ?óÛÿ □ÿ ëÖ•□□™ý•□ç·þ;ÿ ×«-
¶ÿ f‡ËÝ»œç□©è □Š(□Š(□Š(□Š(□Š(□Š(□Š(□Š(□Š(□Š(□Š(□Š(□Š(□Š(□
Š(
×-
Ÿj□7íÛžÙª¿Ù□þ{□ã¿ýzÓ¢€3?²□üöÿ Ç□ïõnÒÛì±²oÝ"œã□bŠ (¢Š (¢Š (¢Š (¢Š (¢Š (¢
Š (¢Š (¢Š (¢Š (¢Š (¢Š (¢Š (¢Š (¢Š (¢Š (¢Š (¢Š (¢Š (¢Š (¢Š *•Ý—
Ú¤Vó6àc¦jå□□™ý•□ç·þ;ÿ ×£û
ÿ Ïoüwÿ ¯ZtP□göAÿ žßøïÿ ^-Ú[}•□wîÉÏLUŠ(¢Š(¢Š(¢Š(¢Š(¢Š(¢Š(¢Š(¢Š(
 ¢Š(¢Š(¢Š(¢Š(¢Š(¢Š(¢Š(□„úižv"ÍÆîÛj?ìfÿ =¿ñßþ½iÑ@

†?*□□9Ú1š}□P□E□P□E□P□E□P□E□P□E□P□E□P□E□P□E□P□E□P□E□P□E□P□E□P□E□P□E□P□E□P□E□
P□Y•¤–b|î§?wÿ ¯ZtP□;K-
²Ê_ÌÝ•Æ1Š¹E□ QE□ QE□ QE□ QE□ QE□ QE□ QE□ QE□ QE□ QE□ QE□ QE□ QE□ QE□ QE
□ QE□ QE□ QE□ QE□ QE□ QE□ QE□ QE□ QE□ QE□ QE□ QE□ QE□ QE□ QE□ QE□ QE□ Q
E□ QE□ QE□ QE□ QE□ QE□ QE□ QE□ QE□ QE□ QE□ QE□ QE□ QE□ QE□ QE□ QE□ QE□
QE□ QE□ QE□ QE□ QE□ QE□ QE□ QE□ QE□ QE□ QE□ QE□ QE□ QE□ QE□ QE□ ÙË‰ß□Ú
Åg□k•ùáÿ •□ðªýÇü{KþáþUÎÐ□Ÿö¸ÿ ž–
ø÷ÿ Z•íqÿ <?ñïþµfQ@□□Ûãþx□ãßýj³iyö¢ÿ &Ý¸ïšÃ–=#¬ß‡õ □:(¢€
(¢€
(¢€
(¢€
(¢€
(¢€
(¢€
(¢€
(¢€
(¢€
(¢€
(¢€
(¢€
(¢€
Šæ□ªÂdÛ»□³Š–ªj_ñäßQüè□¿ö¸ÿ ž–
ø÷ÿ Z•íqÿ <?ñïþµfQ@□□Ûãþx□ãßýj?µÇüðÿ Ç¿úÕ™E kE©ù²¤~N7□gwÿ Z´+Ÿ´ÿ •¸¿Þ□Ð
P□E□P□E□P□E□P□E□P□E□P□E□P□E□P□E□P□E□P□E□P□E□P□E□P□E□P□E□P□E□P□k»¿²„;7nÏ|U_í
qÿ <?ñïþµ.¯÷búŸéYt□§ý®?ç‡þ=ÿ Ö£û\□Ï□ü{ÿ –Y”P□Ÿö¸ÿ ž–
ø÷ÿ ZŸ□§æÊ‘ù8Üq□ßýjÉ©–?ãî/÷… t□QE □QE □QE □QE □QE □QE □QE □QE □QE □QE □
QE □QE □QE □QE □QE □QE □QE □QE □QE □Rîûì²*ù{²3×□n²uoõéþïõ □ÿ k•ùáÿ •□ð¨
þ×óÃÿ –ÿ ëVe□□§ý®?ç‡þ=ÿ Ö£û\□Ï□ü{ÿ –Y”P□Ÿö¸ÿ ž–
ø÷ÿ Z•íqÿ <?ñïþµfQ@□Ñj~l¨žN7□gwÿ Z´+Ÿµÿ •¸¿ß□ÐP□E□P□E□P□E□P□E□P□E□P□E□P
□E□P□E□P□E□P□E□P□E□P□E□P□E□P□ õ/"f•ÊÎÞû¿úÕö¸ÿ ž–
ø÷ÿ Zª_□Çì¿Qüª½ iÿ k•ùáÿ •□ð¨þ×–
óÃÿ –ÿ ëVe□□§ý®?ç‡þ=ÿ Ö£û\□Ï□ü{ÿ –Y”P□Ÿö¸ÿ ž–
ø÷ÿ Z•íqÿ <?ñïþµfQ@□□Ûãþx□ãßýj³iyö¦aåíÛïšÃ––
'ïËô□□©E□P□E□P□E□P□E□P□E□P□E□P□E□P□E□P□E□P□E□P□E□P□E□P□Y–«□b<ž‡–
{ÿ –ZUÍ¿úÆúš Ñþ×óÃÿ –ÿ ëQý®?ç‡þ=ÿ Ö¬Ê(□Oû\□Ï□ü{ÿ –Gö¸ÿ ž–
ø÷ÿ Z³(□?íqÿ <?ñïþµhC'›
ÉŒn□Ås•¿gÿ –q□»@□ÑE□ QE□ QE□ QE□ QE□ QE□ QE□ QE□ QE□ QE□ QE□ QE□ QE□ QE
□ QE□ QE□ QE□ QE□ QE□ QE□ QE□ QE□ QE□ QE□ QE□ QE□ QE□ QE□ QE□ QE□ QE□ Q
E□ QE□ QE□ QE□ QE□ QE□ QE□ QE□ QE□ QE□ QE□ QE□ QE□ QE□ QE□ QE□ QE□ QE□
QE□ QE□ QE□ W7ã•□ê–□Ð□ëJÑçÕo¦□má·‰I□˜□¹±ÎÑ□Ì□ÇZé(□□ÔügñkÂ–□Û°ö…¥É¥«/Ÿ

L7D □¨bG\gæ□½zö□ªÛëš--«i»È»…f@Ý@#8>ã¥yßÆ½}WÃ‰á□□7:Î¶é□Vñœ²¦ðw-
L‵´gßÒ»¿
èÇÃÞ□Òô†`Ïil‵;/BÀ|Ä{g4□¯E□P□E□P□E□P□E□P□E□P□E□P□E□P□E□P□E□P□E□P□w-
ñí/û‡ùW;]□Çü{KþáþUÎÐ□E□P□ZzGY¿□ëY•§¤u›ðþ´□§E□P□E□P□E□P□E□P□!â_□Ïasu¥è:d
šž«o□›9l¥½ªíÜ

•êG!W$ûV‡,5ÛŸ□ø/KÖo#Š;‹¸wÈ°,□□'À$žÞµ¡- 4□KÞÚ_ý ×7ð›þIg‡ÿ ëØÿ èM@□xƒXñwö
Çö_†4+i□F¯.£©JÉn¤çåP¿3-9ÇJÏÐ<aâ□¼_…|]¦Y[ß\ÀÓŪÝiîÍ

Á~òá¹□ O>ŸLiø›Uñ□—□»h>□þÛw,%¯·Gmå□Œ-
œ|Ùç§¥p^□ÕgÕ¾(Åsã+i´□n+g‹JÓ^?Ü”<»¬¹"GÇ□°;-À-·\‡‹¼[□¤êšvƒ é©¨kz^w□&“dPÆ½
]Ï\{

¸>Àõõç?□£,ÂëHñ□Ž¥öO□Z—ŽÂ□
Noƒ□š□□üÄ□′:gé€□o<aã□□Íkuâý+F›Gžt,K-&I7[-
8□•úŒú□†}2¼^}{Qñ?ˆt-'â□“xoO□G<6¥
G¨N9
Ó}Õ ó³¯bsŠöŠ (¢Š (¢Š (¢Š (¢Š (¢Š (¢Š *¦¥ÿ -Mõ-
Î-ÕMKþ<›ê?• bQE□ QE□□5§ü}Åþð®,¹ûOøû‹ýá]□ □QE □QE □QE □çž0ø‹{§XêÍá-$ß6”□
í—· ¥´,½Pt2?l/□¹¯C®Câˆ□á□†±ÿ >•üÅ tzUÛßèö7’*¬—□ñÊÁz□Ê Çç\-
ßˆ¼w©ø×_Ñ¼5□‡□×I0+6¤'ÞæHÃñ°ã×°íÖ»?

□È¯¤ÿ ×”?ú ®*ë@ñî—
ã□□ë-□>□{MU-Ø□E¦Þ<¸,`□□□ç¹í@□'†¿á6ûLÿ ð”ÿ Â?äl-Oö_□»vyÝæqŒzWI\7‡<i«?‰ÿ
á□ñ^"□Ÿ«<&{i-¥ß□Ò□½·<,98>‡§□îh□+«¨,-&°¹•b·…

’HÇ□T

'□
óï□øÿ Xñ?Œ.¬/4ë{M6K□·ØpÂf^É±
äãæ□v èEOâ—
o□ø¢/□Û1þÍ¶Ùu®J§ªg1ÛçÕÈÉÿ dSl'cøó|^¡Q|;□UQ€□•ÐP□ ÑE□ QE□ QE□ QE□ QE□ QE
□ QE□□›«ýØ¾§úV]jjÿ v/©þ•—@□□Q@□Miÿ -
q□¼*□šÓþ>âÿ xP□AE□P□E□P□E□P□E□P□Xž#ñ5·‡£·Cmu{}vÌ--
V±î'f□Ï²¨ÈË□ -°03žô□Æx□ÅZÏ^u□□YëV6-sé·)
Ånåö†]Øf'

G¨ VßŠ<IgáM
mRõd‘T„Ž□†^i□áQGrMrÞ ÿ ‘ßÇÿ ö□•ÿ EÕ□ŠW·ÐøÃÀ¶Ö:wö”-wqq□¡”F-Hã]„±àmÜ[ð
É©|V»Œ^C¡ønÒ□□…•ÕÌ-p□¡eù□®òÕç’Î.a□ÎÑ©’
Û,1□®{àñšóÍG]ø¡¢ØË«^hž□°±□L³ZÚO(¸T□œ3|¤□è□Ò»□□Xµñ□‡e«Y-û=Ü+*□ê2:-
pxü(□BŠ(□Š(□Š(□Š(□Š(□Š(□Š(□Š(□Š(□Š(□Š(□Š(□²uoõéþïõ-jÉÕ¿×§û¿
Ö€3è¢Š (¢Š (¢Š š×þ>âÿ |WA\ý¯ü}Åþø®,€
(¢€
(¢€2¼Cª^i□CÜéúTú¥áeH-a!w18Ë1áTu'µpú§Š~#xfÅµ½kAÑ.4˜°×0ióÈn
NìK|-□oÐs^›\7Å□QÇ‡□†ôõ□ë:ölí □¢Ÿõ’7¢ªç'é@□5Æ´ƒÃGZ°¶žý-Ø\A□°åæ

□P□¾EqWzïÅ+[)5võæ„m#S#éËs#]„□`¸|„ãÓ?Jîô}94•□ÃL•·%•´vêÄu□¡Aý+'Ç-#•Ã^□¸¸U
2ß\□³ØÛ ËÏ;ðª□~y>Â€/xo^µñG‡,u« Â
¸÷…nªrC)÷□□øV-sž□ðôž□ð6"¢ÌÀÍo□ïpr□³□`=@,EnÝÝÁag5ÝÔ«□¼□ÒK#□□P2Iü(□œñ·Šgð
í□µ¾™o□Ö·¨IåY[I□§□»¾9Ø«Éü*o□ø,ëÅ>   Ó5«ØáŽâé□•!□
!Ùx□"ÛÖ°<+i>¿.¥ã-N&I/mÚ□.
□6öc$□vi□Ì}±V¾□□É'Ð?ë"Ÿú5è□¸¢Š(¢Š(¢Š(¢Š(¢Š(¢Š(¢Š(□□ûþ?e
ú•åUêÅ÷ü~ËõÊ«Ð□E□P□E□P□E□P□ZZOß—
è+6´´Ÿ¿/ÐP□¥□Q@□axŸWÕô»[tÑ4I5[ë™<´]â8¢□Éy□ô-ÝúVí□□æw¾2ñ¯„&µ»ñ†•£É¢Ï2Ã-
Ö•$›-K□□áú•§çÐ-ÛÄ□ÚåÕŒi j6Ö7"@^K‹□9J`ð□F

qÏµqÿ □_þ□Y´ï□Øþòëpš;‹Ò9□Ö¨Á‹ ·¡$ □□Ê½□€<þòËâ□…”÷—^0Ñ£··□¥•Î˜pª£$ýÿ AZÿ

õMg[ðMŽ©®ù□k°Ý*l□gî‰ù
-ã□èEd|KžMY´•□Ú;,ÚÜÿ éL‡˜í#ù¤>ÙÀ□×'ìi0Çm□pBŠ'F¡□□`*□€□ fø"Ä□^
□ðõæµ¨□û=ªn*¿yÉ8U-ä□?□ã□¹ñRâÄj°xsBŽÕ—
Ì]:k‰□Ù^ n□@Øì□*Óø«¢_ëÞ ¼¶Ó"3ÞC$w□Â?å®Æ□¯¹ÆxîEP·øÓá9`Dœê□jDaôãc+L-Ýx\-
Î€:Ÿ xš×ÅÞ□¶Ö-□â□åd…þôR)Ã)ú-
Ì`ÖÝy§Á»"ukâÉVÚ{X_Ä□□%¼ëµâÜ□"eìG□□«Òè ¢Š(¢Š(¢Š(¢Š(¢Š(¢Š(®mÿ Ö7Ô×I
\Ûÿ ¬o© □ÑE□ QE□ VýŸüyÅþí`VýŸüyÅþí OE□P□E□P□E□P□;â-
□□dÞC¥éúuÆ©-\GæEi□Úª™Æù$?*&A□<ú□U~-x—QñW‡g½Õ ¶†ê□Ù-™mƒlù

?ˆ‟þz
ë03žõçß□¿äVÔ¿ì1wÿ ¡Ð□─‹¼[□¤êšvƒ é©¨kzˆw□&‟dPÆ½]Ï\{

¸>Àá^xÃÆþ□šÖëÅúV•6•<é□—ZL’n·,p
+õ□ôÿ

Íñ:8,.´•□Xê_dñ%©xì □´æø0ù¡1¯ÌGûC¦~˜çg×µ□ø‡JÒ~
Ù7†ôñ$sÃjP´z„ã•-7ÝP□;:ö'8 □h¢Šå<uâ
½.ÂÛJÑðúö¯!¶±_ùçÇÏ)ÿ e□Ÿ®(□ŸÕþ$ê1xîÇJÒlífÑÿ ´¢Ó//%

XÎà-Hð@ù □'<œW¥×•x›@´ðÅ¯ÃÍ&Ï,□kðo'¾ô®C□v÷'&½V€
(¢€
(¢€
(¢€
(¢€
(¢€
(¢€
(¢€
(¢€
(¢€
(¢€
(¢€
(¢€
(¢€
(¢€
(¢€
(¢€
(¢€
(¢€
(¢€
(¢€
(¢€
(¢€
(¢€
(¢€
(¢€
(¢€
(¢€
(¢€
(¢€
(¢€
(¢€
(¢€
(¢€
(¢€
(¢€
(¢€
(¢€
(¢€
(¢€
(¢€
(¢€
(¢€
(¢€
(¢€
(¢€
ã¾%xé<□áƒ¨-
¿Ú.ç□&Ù□ÜÞA;›Øc äôã¨ìj+‹[{¸ü»˜"™□ÎÙ□0Ï®□ |çà/□ø7H¿ŸÄÞ'Ô¯5□□Þd¼¦Ø•·SÆÔ÷Ç
□□□Àã9÷¯□x—MñnŠš®•#½«³
2!B□8<□±ý‡¤□Ð*Çÿ □Óü*Ü□Cm□Š□’(×¢F¡@ü□ IE□P□E□P□E□P□E□P□E□P□E□P□E□P□E□P□
E□P□E□P□w-
ñí/û‡ùW;]□Çü{KþáþUÎÐ□E□P□ZzGY¿□ëY•§¤u›ðþ´□§E□P□E□P□E□P□E□P□□ã/ˆþ□ÐWPÑu=
WÈÔM³□□Ùåo¼‡oÌªG9õ¬□„~>ðÌ¾□Ð<.šžu'
'Ù¼‰>ðÜÄnÛ· ÏZõz(□ŠÖ~%iþ□×®tÿ □iú…□¢m6ú^□¥,`T□Ê,A□'Œ-™ï\åÖ¯□Ä¿□xi¼?ktú^
•tonuI`h£8□F›€$’9ãÒ½bŠ ÂÕüWa¢øƒFÑnaº{•Y•`h£

ŠT□w□ñ×°5ÆxšöO□üW‹Å□-…ÕÎ‰6™ö4°,#/Øä□¸î□•□ïïìkÔ(□ñ¯Žt?ø^çÃ~□·°Öõ
Ò‰-ËI□8□`w»:•¸Áæ½bÂ -
të[y¥2Ë□H•!þ6 □□□±E □QE □QE □QE □QE □QE □QE □SRÿ •&ú•çVê¦¥ÿ -Mõ-
Î€1(¢Š (¢Š šÓþ>âÿ xWA\ý§ü}Åþð®,€
(¢€
(¢€
(¢€
ò□Š□ü&Þ□ñ□†Æ«ÿ □o)-þÏöy□Ög¦í»□□â½bŠ â<□ãï

ø†ÎÇFÒõ?´j□ö(ÒÃäH›B…Vå”□,GCQÝ|]ðž›}seªÍ}§\A+FRæÆ_Ÿ□□R ,□P}+»¢€<ÏIž□+|HÓ
|Kmauk¡è¶Ó‰½ÅÔF6»-Q´íSÎÀ½ýk¯□□ØGãX¼*aºû|-
†ìH#-PL‛,ÙÎxôÇ½nÑ@-□/Â{3ªj□ö¾*ñ]ƒßÜ½ÌÑÙj"$.Ç=□vè3ž ®FßÀ□ü]»Òÿ á,ñX
£¥ÇÛ□£þ•Ù—□
íå;ã□kÛ¨ □Š(□Š(□Š(□Š(□Š(□Š(□Š(

Ý_îÅõ?Ò²ëSWû±}Oô¬° (¢Š *kOøû‹ýáPÔÖŸñ÷□ûÂ€:
(¢€
(¢€
(¢€
(¢€
Âñ7Œ´□□Ão.½□ôD¸b±&I7□,~âœu□kvŠ ðï□üQðn—
âŸ□Þ^k-U¾¡|’Ú¿Ù¦o1B`œ□$sëŠì¾%Yß¡ðÿ Š´«IofÐîÌÒ[Â2ò[°í“hîp□ëé]ý□□åú·Å•□[Ñ
ntß

[ßjšÕÜM□Vkhëå³

fBÀ(□9<žŸ•v~
ÐŸÃ>□Òti\<¶¶ád+Óyå±í’kvŠ (¢Š (¢Š (¢Š (¢Š (¢Š (¢Š (¢Š (¢Š (¢Š (¢Š (¢Š (¢
Š +'Vÿ ^Ÿîÿ ZÖ¬•[ýz□»ýh□>Š(□Š(□Š(
-□ãî/÷Åt□ÏÚÿ ÇÜ_ïŠè(¢Š(¢Š(□šñßŒ-¼□á‰µ‹ˆ^vÞ"†%ã|Œ

□=‡□"íë^uá_ˆ^□Ó®§×uÏ□-÷ˆ¯□,Ó-□ÆÈ□¨†!åðƒó'"^ÕE sw>8Ñbð<Þ/·šK-)#gWŽ6V"□³□
X□>n9Ç¯Jó□

üGõeÞ°<Sâ¿□#jûJÚY%¤í□•□þ□>^□CüOø

+Üè

o□ø«EñN—.¥¢Þ□»H¤h□ÄN„0 ‘† ž□íÞ±£ŸGø¹à ãC¨Úi×□å±ÂÅ0Øàñ÷†
_~3]•□□ç3ü+Xl¤ ãŸ□mHÎÔþÖùp□LléU>
øtÙø7LÖŽ±«Íö›gO°Os°Ö/Þ-R<|§åëŸâ>µê4P□E□P□E□P□E□P□E□P□E□P□E□P□E□P□
E□P□□÷ü~ËõÊ«Õ‹ïøý—ê?•W □Š(□Š(□Š(□´´Ÿ¿/ÐVmii?~_ □J(¢€
â~%xú/□èöìª†úùÌVÍ*±Š<cs¾ÐN□
à□MvÔP□•xSâ□Ão□ÛÏ,¾){íZõ¼Ûëùlgß;ú□Ýüª:□□□^³e{o¨éö÷ö²y-×1,Ñ>□ÜŒ2

□#ƒÞ¬Q@-uà□øŸÅ:ïŽ%□-å□ìí,Ÿù÷ŒüÎ=™ùü□w□¾§□‹£^ê—+#Ai
Í"Ä»˜ªŒœ□^*è

□•E séâû□<□□ŠRÚùìd‰f□$□¦
H□Ø□läàž□5ÍÏñ‹@»…¡ðÜ□úÞ¨Ã□YÁg"üÝ·³(
¾§œW¢Q@□Ï€ü=uáÏ□□u
□MNîy/o□:□¤9`>œ□Âºj(□Š(□Š(□Š(□Š(□Š(□Š(□¹·ÿ XßS]%soþ±¾¦
€□E□P□E□P□[ö□ñç□ûµ•[ö□ñç□û´□=□Q@□□Q@□□Q@□×‰|□á•□]Ãk®ê□dšdó#_"Y2¹ÆrŠGQ^u
ð§â/…,tét‹□Weõî¯3[ÅöyNñ#□œ…ÀÎ{‘ŽõíTP□—
øšöO□üW‹Å□-…ÕÎ‰6™ö4°,#/Øä□¸î□•□ïïìk?Æ¾9Ðü□á{Ÿ□ø^Þë[Ô/J${-$Hà!•Þìê6ã□šö
(□šÖ<Ikà•G]T]]=Ä±X‡,=äÈWï6Hãƒê}ª§‰~□Ùø—
Ä□ëm-ëšmì6ßfVÓnÄ?&âÇøIäžyì+°¢€<CÆÿ □Î•¨xU□‹|Wwö½f(7]j>a‡!ŽøÎß•Æ8?Zõý□Kþ
ÅÒ Óþß}□áîÿ I¿›Í™òÄüÍ□œg□Ø
Ð¢€
(¢€
(¢€
(¢€
(¢€
(¢€
(¢€
(¢€
(¢€
(¢€
(¢€
(¢€
(¢€
(¢€
(¢€
(¢€
(¢€
(¢€
(¢€
(¢€
(¢€
(¢€
(¢€
(¢€
(¢€
(¢€
(¢€
(¢€
(¢€
(¢€
(¢€
(¢€
(¢€
(¢€
(¢€
(¢€
(¢€
(¢€
(¢€
(¢€
(¢€
(¢€
(¢€
(¢€
(¢€
(¢€
(¢€
(¢€

(¢€
(¢€
(¢€
(¢€
(¢€
(¢€
(¢€
(¢€
(¢€
(¢€□20zS<¨ÿ çš□ß"ŸE 3Ê•þy§ýò(ò£ÿ žiÿ |Š}□ Ï*?ùæŸ÷È§**ýÕ□è)h □Š(□Š(□Š(
□Š(□Š(□Š(□Š(□Š(□Š(□Š(□Š(□Š(□Š(□Š(□□¨a† •CKE 3Ê•þy§ýò(ò£ÿ ži
ÿ |Š}□ Ï*?ùæŸ÷È£Ê□þy§ýò)ôP□Dqƒ□Š□Ò□E□ QE□ QE□ QE□ QE□ QE□ QE□ QE□ QE□ Q
E□ QE□ QE□ QE□ QE□ QE□ ŒŠßyAúŠo•-
üóOûäSè □yQÿ Ï4ÿ ¾E-T□ðÍ?ï'O¢€□åGÿ <Óþù□¢8ÁÈE□éN¢€
(¢€
(¢€
(¢€
(¢€
(¢€
(¢€
(¢€
(¢€
(¢€
(¢€
(¢€
(¢€
(¢€
(¢€
(¢€
(¢€
(¢€
(¢€
(¢€
k"1Ë*Ÿ¨§Q@

ò£ÿ žiÿ |Š<¨ÿ çš□ß"ŸE 3Ê•þy§ýò(ò£ÿ žiÿ |Š}□ Ï*?ùæŸ÷È£Ê□þy§ýò)ôP□DQƒ□Š□Ò •E□ QE□ QE□ QE□ QE□ QE□ QE□ QE□ QE□ QE□ QE□ QE□ QE□ QE□ Ó□1ÉE'ÔŠO*?ùæŸ÷ È§Ñ@

ò£ÿ žiÿ |Š<¨ÿ çš□ß"ŸE 3Ê•þy§ýò(ò£ÿ žiÿ |Š}□ Ï*?ùæŸ÷È£Ê•þy§ýò)ôP□<¨ÿ çš□
ß"•QWî¨AN¢€
(¢€
(¢€
(¢€
(¢€
(¢€
(¢€
(¢€
(¢€
(¢€
(¢€
(¢€
(¢€
g•-
üó_ÊŸE 3Ê•þy§ýò(ò£ÿ žiÿ |Š}□ Ï*?ùæŸ÷È£Ê•þy§ýò)ôP□<¨ÿ çš□ß"œ □ `{RÑ@□□Q@
□□Q@□□Q@□□Q@□□Q@□□Q@□□Q@□□Q@□□Q@□□Q@□□Q@□□Q@□□Q@□□Q@□□Q@□□Q@□□Q@□□Q@□□Q
@□□Q@□□Q@□□Q@□□Q@□□Q@□□Q@□□Q@□□Q@□□Q@□□Q@□□Q@□□Q@□□Q@□□Q@□□Q@□□Q@□□Q@□□
Q@□□Q@□□Q@□□Q@□□Q@□□Q@□□Q@□□Q@□□Q@□□Q@□□Q@□□Q@□□Q@□□Q@□□Q@□□Q@□□Q@□□TsO□´□<
ò¤QF
<ŽÁU@îIè(□J+□´ñï„¯¯…•·^´Ùn□,ª-
ÂüÄô ô'é]□ □QE □QE □QE □QE □QE □QE □QE □QE □QE □QE Gpqm)□v-
åX-lŸóÑ¿:ß¸ÿ •i□Ü?Ê¹Ú □›'üôoÎ□6OùèßŒ2Š □›'üôoÎ□6Oùèß□2Š □›'üôoÎ•6Oùèß•2
Š □›'üôoÎ□6Oùèß□2Š □›'üôoÎ¦´'ÍÜ@»□»Ö«Tö□ñù□ûÔ□¿E□P□ERÔu•/HE}OR³²Vû-s:Æ□
Óq□5¥í®¡l·6W0ÜÀßvXd

§èG□□=□ZûQ±Ó-þÑ¨^[ÚCœy—□¬kù’□%†§aªÛý£N¾¶¼‡8ó-åY□?U$P□ª(¨®.`´□ç¹š8aA-’F
ª=ÉàP□´VnŸâ□□W•¢Óu□>öE□d¶¹I□ú…&´¨ ¢Š(¢Š(¢Š(¢Š(¢Š(¢Š(ªº‰+fÄ□

G"-UMKþ< ›ê?• cù²□ÏFüèódÿ ž•ùÓ(□ù²□ÏFüèódÿ ž•ùÓ(□ù²□ÏFüèódÿ ž•ùÓ(□ù²□
ÏFüèódÿ ž•ùÓ(□ù²□ÏFüèódÿ ž•ùÓ(□m)™’]ÌO#©-
ÍÒ~ä¿QZT QEgj□þ□¤H±êZ½…"·*·7)□?@ÄP□□□ÈfŠæ□š
RXœe]□2°õ□uª°Ž±¥é□«êz•□’7Ýk™Ö0~›ˆ
´T6——·öËsgs□Ä□÷e…Ã«}□â¦ □Š«}©Xévþ~¡{mi

qæ\J±®~¤□I§êš~-□ŸM¿µ¼„□□-¦Y□?U&€-
ÑE□ QE□ QE□ QE□ QE□ QE□ QE□□□ª³*Åµ^äô?JÍódÿ ž□ùÖŽ¯÷búŸéYt ÿ 6OùèB•-lŸóÑ
¿:e□ ÿ 6OùèB•-lŸóÑ¿:e□ ÿ 6OùèB•-lŸóÑ¿:e□ ÿ 6OùèB•-lŸóÑ¿:e□ ÿ 6OùèB•-lŸó
Ñ¿:e□ ÿ 6OùèB□léäµš□I9<Ÿ-aÖæ□ÿ -Iõ?Î€-QYmâm□oþÂÚÞš/7mû9»□ÌÏ¦Üç5©@□□™}âM
K¸□ú†µ§ZNzGqt‘·äH5¡□Ñ\B’Ã"I□Œ«£□□z,:Ð□è¢³µ□□FÒ$Xõ-^ÂÉÛ•[›”ŒŸ b(□FŠd3Es
M□©,N2®Œ□Xz,:Óè ¢Š(¢Š(¢Š(¢Š(¢Š(¢Š(¢Š(¢Š(¢Š(¢Š(¢Š(¬-QÙg@¬GËØû
Ö-dêßëÓýßë@□¼Ù?ç£~ty²□ÏFüé”P□üÙ?ç£~ty²□ÏFüé”P□üÙ?ç£~ty²□ÏFüé”P□‹i$7Q□ìF
áÞ·«Ÿµÿ •¸¿ß□ÐP□E□P□E□P□E□€ ' w¬Ëo□èw·¦Ê×ZÓ§»□y□]#?ýò

h□NŠ+,ø“B[ÿ °6µ§
Üíû9°O3>›sœÐ□¥□Q@□□□ÕÕ½•´—7sÅ□¼csË+„T-¤ž ¤´¼µÔ-c°²¹†æÞA”š□

Œ=A□□ šŠ(□Š(□Š(□Š(□Š(□Š(□Š(

KÙ□^H□°□è□µWódÿ ž•ùÔ·ßñû/Ô□*¯@□ódÿ ž•ùÑæÉÿ =□ó¦Q@□ódÿ ž•ùÑæÉÿ =□ó¦Q@□ód
ÿ ž•ùÑæÉÿ =□ó¦Q@□ódÿ ž□ù×DŸq~•ÍWJŸq~″ ´QE □QM'Há□¤•Õ#Q–f8 z"@

¢³¬<A¢ê³<:v¯ay*rÉor’2ýB“ŠÑ □ŠË¾ñ&…¦\
}CZÓ-'=#¸°HÛò$□ÑŠXç‰eŠE’7□WC•ÃÔ□ }□É¦ŠÞ 'žDŠ(Ô»Èì□U@É$ž€
Âÿ „ïÁÿ ô5è□ø1‡ÿ Š

,Š†ÒîŰþÖ;«;ˆ®-å□£-□

Ž=A□□š€
(¢€
(¢€
(¢€
(¢€
(¢€
(¢€
ç^Y<Æýãu=ë¢®mÿ Ö7ÔÐ□ù²□ÏFüèódÿ ž•ùÓ(□ù²□ÏFüèódÿ ž•ùÓ(□ù²□ÏFüèódÿ ž•ùÓ
(□ù²□ÏFüèódÿ ž•ùÓ(□ù²□ÏFüèódÿ ž•ùÓ(□ù²□ÏFüèódÿ ž•ùÓ(□}-
™¢"s□ów5~³ôŸõ2□½RA«i·WóØ[ê□"^ÛŒÍo□ÊÒF?ÚPr?□ ¹EG<ðÛ@óO*E□

³ÈÁUG©'¥RÓõý□W‘£Óu{
Ù□e–Úå$+õ
M hÑE6I#†6’WT□FY˜à□êM :ŠÎ°ñ□‹ªÌðéÚ½…ä©Ë%½ÊHËõ
N+F€
(¢€
(¢€
(¢€
(¢€
(¢€
(¢€
(¢€
(¢€
(¢€
(¢€
(¢€
(¢€
(¢€
(¢€
(¢€
(¢€
(¢€
(¢€
(¢€
(¢€
(¢€
(¢€
(¢€
(¢€
(¢€
(¢€
(¢€
(¢€
(¢€
(¢€
(¢€
(¢€
(¢€
(¢€
(¢€
(¢€
(¢€
(¢€
(¢€
(¢€
(¢€
(¢€
(¢€
(¢€
(¢€
ç<ká;□è‰§jW—□ÖqÎ·□˜\.õP~V'øyÏà+£ªZµ□ž-¦\iwØ0^ÆÐ²□ÚX□r□¾(□Ã~)øká®‰à‰F•-
1k
È-□ÞäÉ#□Ãvá¸ämÝÉï□¥{/„□ù|□¢®§æ}´YEçy™ß»hÎìÿ □¯¾kË<]ðCÃÚ/‡ou½
öúÂÿ M…®âv›ræ1»Ó
ñÁ□ƒ]÷Ã□□Þø£áö™ªj$5Û‡ŽI À•«□Ý•p9÷Í uôQE □QE □QE □QE □QE □QE □QE □QE □QE
 □QE Gqÿ -Òÿ ¸□•sµÑ\□Ç´¿î-
å\í □QE □QE □QE □QE □=Ÿü~EþõASÙÿ Çä_ïP□ý□Q@□´ß□ü/q¬Þë:¦Ÿ□£wrrÏ¨be•@ÀTVù
T□¦yë\¯€,ìlþ'x□<1□á±□BE„æ□»Ï"3Ó…ÎqÓ8ô®·Äß□¼1ã
¸®µÍ4ÝO□~Tn'’=«’q…`:“Ö¹]

ÞãÀ□□4ÿ XßOu êV’Ï□µÃo{7NNÖë°ã□=ÿ P

·Rð.•-kÿ Û:½Ÿö„ë□Š(®□™

@g;c<dç’sÒ¸æÑl<-ñ·A‡Ã°%”:¥•ÁÔm
□bÚ‹˜ß`à□ÜqéîsÞx“Äš□.ô¦¾¾fbÌ#,□†én$?u□{±¬_□øzýu
Ï□ø‰Tk°Š□X□ål-Ç+
žç»-çõ ì«Ë~%ÇfÞ5ð³x˜□á?Þ‰w’
□$|†\vôÏ□{f½J¼ÿ Ä×wž)ñ‹x□ÖálìE□Úõ)ÄjòHŒÛDQ†□

õ-ƒíî□ÎøëBðÄWz□>

³²·ñKÞE-¡Ó□T¬YË¼>8òö÷=□ö*ò=gÁëð«M¹ñOƒ®^□-Õ□ö□tÞdwQ,□Ã™□œä□{v¯U²°Kë
{¸Ã*O□Ê¡°€À□ŸÎ€'¢Š(¢Š(¢Š(¢Š(¢Š(¢Š(ªš—
üy7Ô□·U5/øòo¨þt□‰E□P□E□P□E□P□E□P□E□P□¦"÷%úŠÒ¬Ý'îKõ□¥@□qéðëÁ¶BòóPÓmof¸v
šâïSÄ¬I9ûÍÂ□ÓŒp+°®?Ä?
ü!â□J]KUÒÚ[éUU§[™Pà

□mè=(□□á□□ÛËâ„Ò™ÛÃ_Ú'û,’J-?y°ž©œ {ãë]$Ÿ□|3u-Þk□Ž›-§ytÃ-~□Ë□□€¨-ò¨-
LóÖ°|□=þƒã-[Á□ßÍ¨i¶-‘ÝÙK9□,
Ä□□7~¹-Â°O□x®□ZÅ□P5î¯xLv□|_~wþŠ:-<□@□□…4Ûo

|f×4]□L:D°\w"Z¡&8n
€6•áÊäãú□^¡\¿,¼/6•kw{©Î·Zî©/Ú5
…û»¿†5ÿ aG□ñúWQ@□Þ¥à_□ë:ÿ öÎ¯f5
Ö!□q]□ðÄ£9Û□ã'<"šâôÍ3IÓþ7ÛÇá□â†Ñtù?¶b³ÿ P‡?»□□">qÇ ú×uâo□h-1Š
Þ=zÃíinY¢-t'í'□ûŒ3Ðu®□M#þ□□¼;oáû»□ì]jðÚ\i"ÈdXØŽ$ŒžF;óÿ Ö õŠ(¢€
(¢€
(¢€
(¢€
(¢€
(¢€
(¢€3u□»□Ôÿ JË-M_îÅõ?Ò²è ¢Š(¢Š(¢Š(¢Š(¢Š(«òi±êþ□ŸNšiáŠå□7x-
c€O8=ª…nißñäŸSüè□•ñO•þ□h^
½[Í'N³†;w□ÎTyûñòísó3g□□9-Ÿ□¦¶Ÿ
4Ä¼Þ5•bByÿ x6□—»=ñ·9¬»¿,Þ

wk•:ÚëKÔ□□#¼µ»—z?\€ÌGZg„üsz>□jšæµ‹»--K^$-0□]□°0Ç□9□?• `|9Ò¼
yáIŽ¿g§Ë¯@Ò□`êÁLñÉ¸å˜¿ täqøæ·~□Z
h¼A&š³'†f¿-¤¬›°S;&îv□Æ>†©Û|3³ñæ›□^¼]u%Æ©□l'DlÈ…-
□€eTÀù^□«î-□□júµ¯‰uo□ë7I□.›□s[_*□i!nŠê8

8□□□̌□y\z|:ðm•¼¼Ô4Û[Ù®□¦¸»Ôñ+□N~óp tã□
ì+•ñ□Âÿ □x§R—RÕt¶-
úUUiÖæT8□□€Ûz□J ÃøE□vòø¡4¦vð×ö‰þË$’‡•Þl'ªg -øú×¦Wœø&{ý□ÇZ·,%¿›PÓm-#»²-
r□X□ˆ□&nýr=…z5 □QE □QE □QE □QE □QE □QE □QE □QE □QE □QE □QE □“«□̄O÷□̶kVN
-þ½?Ýþ´□ŸE□P□E□P□E□P□Ö¿ñ÷□ûâ°
çí□åî/÷Åt□ QE□ QE□□—â□□ÏÄÚKéwï8´‘Õ¥He1™ 9ÚHçiî□pŸ□ü□á-
;áö¥{i¤Zi×Z|□{[«T□Ê²/ÝùÇ''□œõõ¯NfTRÌ@P2I<□^otÍñOWŽÖ □å□°œ=Äý□§2-□=bSÔ÷=
:f€:•>Úo□x□Æ□Nk^g¾Ó¢ûL□6É□4`¾□c’k□Tøaàhü/wht++xc□ÛíJŸ¾□ •Þaù‰□y8õ®è □□□
¼ïÄWÓøÿ QŸÂZ$¬°LO³ZÔ£û w·Œ÷süG¢Ž□\P□ŸÂ›ÛíCá†…s¨Èò\´,¥Üä²«²¡'¿Ê□šìj□KH,,
à³µ‰b·,5Ž(×¢¨□ ~□‘ã-SRÑ¼#¨ßhö□_j1Æ□¼□ÆdbìBƒ´r@Îâ=□

WÄäøçÄ²ør"[CÑÔ\ê®>ìóã1Ûû□÷›è□□¯ü-ÿ ’O □k)?ôk×-áÏ□EáŸ
□f',<q,î¯-ÕÔšG3Nü¼Œw÷? -•□à—ˆ~×àÝ3EþÇÕáû5³¿Û¦¶Ûk/ïO
&~cótÇcé@-¥E□P□E□P□E□P□E□P□E□P□E□P□E□P□E□P□□÷ü~ËõÊ«Õ‹ïøý—
ê?•W □Š(□Š(□Š(□°Tû‹ô®j°Tû‹ô □¢Š(¬O□øWLñ\6¶ú²Í-
¬□ù¦Ýe+□§□□ÀûÀuÆkn¡°° ·±´-êêd†Þ□/$’6□Tu$Ð□”|Sð®‡á¿□ÚëÞÓm´ÍjÎò□föQˆŒŒÎ□F
•Ù□<óÇ¦k°ø□®^hž□□ïÇ ©_Ï□…›Ÿá’VÆï¨□?QY□d□-
□<Ggâ+Èd‡ÃzkùšU¼ªU®åÿ Ÿ†□¢•à□žÿ X>6[Csá•□ÝçìQk--tC□ÄD:·#‘÷°Ð□ôÏ
ü0Ñã]2ò]□ûRn'—Pž)n&□õc¼’
=…w°f™e£éÐØiÖéoi□"8“¢,I8üI®u¾□ø!´æ²ÿ „gNX™vï□□0{‰>ö}óYŸ□®n'ð□
C4ïq□¥ÜöÖÓ1ÉxU¾Sžã¨-Â€7ü{ÿ $ïÄ¿ö
¹ÿ ÑM\—„->□]øsF,[□□Í¨µ”□XÙ-šS&Á»pë»9ÎyÍzUÅ¼7vÒÛ\Ä“A2□äŽE

®¤`,□PEs7Ÿ□|□}höÒxcKDa□Ð[,N>Œ Ö€:KKKk
Xílíâ··ˆmŽ(P"
ô p*jóÿ ƒ÷WSxFòÎ{™.¡Óµ;‹+Iä92B„m9ïÔ• ¯@ □Š(□Š(□Š(□Š(□Š(□Š(□¹·ÿ Xß
S]‰soþ±¾¦€□E□P□E□P□E□P□E□P□E□P□E□P□□□Ä'x{@ac□ŸX¾˜ZéÖÿ ß™‡□ÿ ²¿xŸAï\Ï,<:
ž□ø¹s§ù¦{" $×w□÷§□ç‰ÜýOè□gÙxŠæ×â

¥¬jÞ□ñM÷Ø‹ YébÇM2Å□g□.âFYñÔ□□□5□·•¶ü^»Õ□á□ñQó4t·û
Ó□Ò□□çyMÜ'lç- t~!³•Æß□ ðÖ YôM&Ä_\[□!n'fÂ□ÇP□?Ÿ-Cñ-
Á□F□áy¼Iá½>ÛIÖ4}·PMg□‹ r©□•,à0+ž¿Èš¹§0³øû¬Ã/Ê×ú43CŸâ□ÛH¯åZß□/b±øaâ
%`□--
ç»>□□øš ét«åÔô‹-A□□ê□˜□@Ê□úÖ□ˆü+¦x®□[}Yf-Ö
|Ón²•ŽSŒ□à}à:ã5?†m$Óü)£ÙL□-
ÞÆ□œ-ÅP□üªõÕÕ½□¤·WS$6ð©y$‘°ª£©&€<£âŸ…t?□øn×^ðþ›m¦kVw•
7²ŒDdfp
0\nÈÉçž=3^»^w¦Aqñ□Äv~"¼†H|7¦¿™¥[Ê¥Zî_ùø`z(þ yïõôJ (¢Š (¢Š (¢Š (¢Š (¢Š (
¢Š (¢Š (¢Š (¢Š (¢Š (¢Š (¢Š (¢Š (¢Š (¢Š (¢Š (¢Š (¢Š (¢Š (¢Š (¢Š (¢Š (¢Š
(¢Š (¢Š (¢Š (¢Š (¢Š (¢Š (¢Š (¢Š (¢Š (¢Š (¢Š (¢Š (¢Š (¢Š (¢Š (¢Š (¢Š (¢Š
(¢Š (¢Š (¢Š (¢Š +’ñï□ ñÎ□k□Ô.4ûË9|ëk˜y(Þã#=°□xë]m□□ãrü!ñn¯□°×¾
Þ]i@•ðª±i□□á²Ý}Îq^-£i□Z□•k¥iÑ□--
•$küÉõ$ä"êjõ□ QE□ QE□ QE□ QE□ QE□ QE□ QE□ QE□ QE□ QE□□□Çü{KþáþUÎ×G*—
…Ðue f²¿²§þô□™ÿ
£E^þÊŸûÑþgü(þÊŸûÑþgü(□•□{û*□ìGùŸð£û*□ìGùŸð
4Uïì©ÿ ½æ□Â•ì©ÿ ½æ□Â€(ÑW¿²§þô□™ÿ
?²§þô□™ÿ
£SÙÿ Çä_ïTÿ ÙSÿ z?Ìÿ …I□□4S£³&□äàš Ô¢Š(□,°ø□¬[jwwž□ñ®¡¤Çw3Ï-
´°%ÜJìIbŠøÛ’I-□

x□=□U¸Öõ□RëYÖç•ÊkÛ•□dyÎÈÐp£<×[E y÷^þkšÇ□□Äš□‹ÿ ³ä†!□´2i©r-
Æ□â»Û ±ç8Ï8Î*ÖŸá□□[ê6Ó^|Eû]¬r«Moý‰□~j□Êî□+‘ÆGJíè

-
B¿Ô|A£j6ÚíÕ•µƒ;Og□;.Ã□€ÜŽ˜î□^Õ□â□□G¯jÖÚÞŸªÝèÚÝ´f□¼¶□·Çœít<0É5×Q@-w'Ã}
Z1Ãâß□^k:z8v²ŠÒ;Hå ä□Ù’Ã=«ÐÕU□*€ª□ □
Z(¢Š(¢Š(¢Š(¢Š(¢Š(¢Š(ªš—
üy7Ô□:·P]ÂÓÛ˜Ð€I□h□□Š½ý•?÷£üÏøQý•?÷£üÏøP□□*÷öTÿ Þ•ó?áGöTÿ Þ•ó?á@□h«ßÙSÿ
 z?Ìÿ …ÙSÿ z?Ìÿ … Q¢¯□eOýèÿ 3þ□□eOýèÿ 3þ□□FŠ½ý•?÷£üÏøQý•?÷£üÏøP□ÚOÜ—
ê+JªXÚ½²¸r§q□Á«t „□□=□yä?

|A¤)¶ðï•õ+□;ø-®-c»òÇ÷Qœ,£Ò½□Š æ¼'àËO

Ë•µÜê□•ó□»¿°lÉ)□□²ŽÂ¹»¯‡-%>0Ô|E§xçì—
□‡j‰4˜çhbÎDjÎÜ(ö□=MzM□□Èh~-ñ•†¯□Î-ã¿íK$ÝæZ□dC□™• |êr0p}ñŠÒ}
ýük□¸5Û¥°KC□ÒÀ>S>Ió□8Ï>™ã¯jÝ¢€8-gÀ°…Æ½s-x□Åz†‹wu´ÜF#[ˆ□ª…□å· à□š4O Ik¯Ã
¯ø‡^»×µKud¶y£X¢ƒw□¤kÀ$w®ÖŠ (¢Š (¢Š (¢Š (¢Š (¢Š (¢Š (¢Š ÍÕþì_Sý+.¶¯-d¹□□
¨ÛœäÕ?ì©ÿ ½æ□Â€(ÑW¿²§þô□™ÿ
?²§þô□™ÿ
 £E^þÊŸûÑþgü(þÊŸûÑþgü(□•□{û*□ïGùŸð£û*□ïGùŸð
4Uïì©ÿ ½æ□Â•ì©ÿ ½æ□Â€(ÑW¿²§þô□™ÿ
?²§þô□™ÿ
 £V5□

ßÄž□–J°šâ□æ ‰mäÙ"2¸ee=ˆ □›û*□iGùŸð-□HZ
u•ÈÈÏJ à□€<[$□Êo‰Z“iÄmd[(Ör¾žvwgÞ°-;Âš6—
áQá«{@t¿%¡xœäÈ□;‹-ääó[TP□œÚü:ñ&• ±ðÿ Ä□ë-18ŠÚâÆ+–•□º®Ø

ÜWCá?□ÚøXÞ\›Ë□GT¿`÷w÷M™%
`□Ø(ì?úÕÒÑ@□>-Ðo´yµg¾×.µD½ºiáIÁ□Ù□H×“ÀöÀö□ÌÃðçÄ□B›o

øÿ R°Ó¿,ÚêÖ;¿,□u□È*=+Ñ(

kÂ~

´ð°¼¸û]Î¡©ß0{»û¦Ì’‘Ð{(ì+¥¢Š (¢Š (¢Š (¢Š (¢Š (¢Š (¢Š (¢Š (¢Š (¢Š (¢Š (¢Š
+'Vÿ ^ŸîŸ ZÖª7Ör\Ê¬…@□□š Ç¢¯□eOýèÿ 3þ□□eOýèÿ 3þ□□FŠ½ý•?÷£üÏøQý•?÷£üÏøP
□□*÷öTÿ Þ•ó?áGöTÿ Þ•ó?á@□í□ãî/÷Åt□•□›4s£–L+□pMjÐ□E□P□E□P□7ãŸ

]ø¿ÃRhö°Äš_œàË*EæoAœ¡□—ƒÆyíŽõÏZø#Ç–V±ZÚüIHmáP‘Ç‡-ÕUG@□kÑh

nç@Ö¯|□6‰qâY

«4m□jÑÚ¬mËg"5 □—åà•^µËi?

üi¡i□éÚgÄXí-!□H‟@ƒ□Ü’Ù$÷'‟^›E cx□MÖ´ý.[}k_:½ÛHÌ—BÑ-
Ê) □Ú¹□□□Ÿ□jo„ôKï□hIa¨ëw:ÍÂÈÌnîA

A9□-'□ë[tP□&□Î,H³□êW>™□□àÿ

•
øRÃC□_jû"2ùÞ^ÍùbßW'□}krŠ (¢Š (¢Š (¢Š (¢Š (¢Š (¢Š (¢Š Á¾ÿ •Ù~£
ùUzÓ¹ÓæšáäR˜n™5□öTÿ Þ•ó?á@□h«ßÙSÿ z?Ìÿ …-
ÙSÿ z?Ìÿ … Q¢¯□eOýèÿ 3þ□□eOýèÿ 3þ□□FŠ½ý•?÷£üÏøQý•?÷£üÏøP□□éSî/Ò²?²§þô□™
ÿ
×Q…□ÐP□ÑE□ Wñ□Áw¾6²²´¶×N™□¼Þt±›Q:NF6†RÀ□□'□
ç§□ØQ@-|¾□ø„ª□~' `□ [à~µÔßø~□sÂ□¡k²›åšÝb¹˜
ŒÈà□Þ 8S¸n p□lQ@-n¿□¼FÖ_Ù3üDÔßEÛå›uµ□f1ôÚfÉnœt®¸xr
/□?‡´YŸK‰mš
y¡å¡$œr lœç9'½mQ@□ãxjþO□Á¡¿ˆõ□¾‰□□V#¶ge ääœƒŒ□IÈïX□x□Å×±›MGâEü¶

6¼vÚ|PJËÜy “úW¡Q@□ú&‹aáÝ-ÛJÓ
□Ú[®Ô@r}I'¹$’O½hQE □QE □QE □QE □QE □QE □QE □Í¿úÆúšé+!´¹Ë’□>O©ÿ
 ¡E^þÊŸûÑþgü(þÊŸûÑþgü(□•□{û*□ïGùŸð£û*□ïGùŸð
4Uïì©ÿ ½æ□Â•ì©ÿ ½æ□Â€(ÑW¿²§þô□™ÿ
?²§þô□™ÿ
 £E^þÊŸûÑþgü(þÊŸûÑþgü(□•□{û*□ïGùŸð£û*□ïGùŸð
□Oú™?Þª□xdEãÛ□□}¬“6ž¶?fòúa÷îÝŸÃ□ükVÆÙí£er¤“ž*Õ rþ.ðM¿Š%²½ŠúçLÕì□›[ûloLõ
R?‰O§øœäAðæþÿ R³»ñoŠ®µø¬¤□ÛÚ□d¶‡xèÎ«•ä{ÿ ‚Šïè

/□èWúì□1Øk·ZK[Ý¤ò=¸$Ì‹œÆpG□>ãŽ†³> x.÷ÆÖVV–ÚéÓ!·›Î–3j'IÈÆÐÊX□□□àä□ôâ»
(□Ï—Â□•B¯Äà□

4
|□Ö»«□® °¶†îçíW1Ä«-Ç-□Íp -
´p¹98□3SÑ@□□Q@□□Q@□□Q@□□Q@□□Q@□□Q@□□Q@□□Q@□□Q@□□Q@□□Q@□□Q@□□Q@□□Q@□□Q@□
□Q@□□Q@□□Q@□□Q@□□Q@□□Q@□□Q@□□Q@□□Q@□□Q@□□Q@□□Q@□□Q@□□Q@□□Q@□□Q@□□Q@□□Q@
□□Q@□□Q@□□Q@□□Q@□□Q@□□Q@□□Q@□□Q@□□Q@□□Q@□□Q@□□Q@□□Q@□□Q@□□Q@□□Q@□□Q@□□Q
@□□Q@□□Q@□□Q@□□Q@□□Q@□□Q@□□×`^Îz($â©ÿ jÁýÙ?!þ4□zŠ£ý«□÷dü‡øÑý«□÷dü‡øÐ□ê*
šjP;ª□|±ÀÈ-
ãW(¢Š(¢Š(¢Š(¢Š(¢Š(¢Š(¢Š(¢Š(¢Š(¢Š(¢Š(¢Š(¢Š(¢Š(¢Š(¢ŠŽy-□ŒŽ
□Ò€$¢¨ÿ jÁýÙ?!þ4□jÁýÙ?!þ4□zŠ£ý«□÷dü‡øÑý«□÷dü‡øÐ□ê*•ö¬Ý"òãGö¬-
Ý"òã@□¨ª?Ú°□vOÈ□•-
Ú°□vOÈ□• ^¢¨ÿ jÁýÙ?!þ4□jÁýÙ?!þ4□zŠ£ý«□÷dü‡øÑý«□÷dü‡øÐ□ê(□#4P□E□P□E□P□E□
P□E□P□E□P□E□P□E□P□E□P□E□P□E□P□EAqu□°RáŽî˜□□ö¬Ý"òã@□¨ª?Ú°□vOÈ□•-
Ú°□vOÈ□• ^¢¨ÿ jÁýÙ?!þ4□jÁýÙ?!þ4□zŠ£ý«□÷dü‡øÑý«□÷dü‡øÐ□ê*•ö¬Ý"òãGö¬Ý"ò-
ã@□¨ª?Ú°□vOÈ□□[ŠEš%'s†éš }□Q@□□Q@□□Q@□□Q@□□Q@□□Q@□□Q@□□Q@□□Q@□□Q@□□Q@□□
Q@□□Q@□□Q@□□Q@□□Q@□□U{‹Èíœ+†$Œð(□Å□GûV□îÉù□ñ£ûV□îÉù□ñ
ÔU8õ(d'P+åŽ□@ÿ □¹@□□Q@□□Q@□□Q@□□Q@□□Q@□□Q@□□Q@□□Q@□□Q@□□Q@□□Q@□□Q@□□Q@□
□Q@□□Q@□□R]B□ehÙ_+× S?µ`þìŸ•ÿ □ ½EQþÕƒû²~CühþÕƒû²~Cüh□õ□GûV□îÉù□ñ£ûV□îÉ
ù□ñ
ÔUíX?»'ä?Æ□íX?»'ä?Æ€/QT□µ`þìŸ□ÿ □°

@>´ ´QE □QE □QE □QE □QE □QE □QE □QE □QE □QE □QE □QE □QTŽ© $□"□aþ4□vŠ£ý«
□÷dü‡øÑý«□÷dü‡øÐ□ê*•ö¬Ý"òãGö¬Ý"òã@□¨ª?Ú°□vOÈ□•-
Ú°□vOÈ□• ^¢¨ÿ jÁýÙ?!þ4□jÁýÙ?!þ4□zŠ£ý«□÷dü‡øÑý«□÷dü‡øÐ□ê*½½äw,Á□

rr*Å □QE □QE □QE □QE □QE □QE □QE □QE □QE □QE □QE □QE □QE □QE □QE □QE □Q
E □QE □QE □QE □QE □QE □QE □QE □QE □QE □QE □QE □QE □QE □QE □QE □QE □QE □
QE □QE □QE □QE □QE □QE □QE □QE □QE □QE □QE □QE □QE □QE □QE □QE □QE □W−|IøÅ−
,u¨4‹
□¯îÂ‡¹ß)Q□?ux□æ#ŸaŽ¹ãÓà—Î·Ž\cz□Ç¦Fh□J(¢€
(¢€
(¢€
(¢€
(¢€
(¢€
(¢€
(¢€
(¢€
(¢€#¸ÿ •i□Ü?Ê¹Úè®?ãÚ_÷□ò®v€
(¢€$·ÿ •˜¿ßÎ°*çmÿ ãæ/÷Çó®Š€
(¢€
(¢€
(¢€
(¢€
+˜ñ7•ôÿ □HÖqE6¥«˜Ì©§Ú□Ò□□;œô•□ÚoÃ5{ÂzøñG…´ýl[}›í‘ùžNýû9#□˚3ÓÒ€6h®KÄ−3°Ó
µ□ìm□Ã·ÚÞ¤±‰dX˜E

Js•ò· œ□
‡Ãž>}SÄ□áíoC¹Ðõ¡□•□¼Ò,©2

¥$^[illegible]Ðú[illegible] vtQYZ÷ˆô¿[illegible]Y-Ö§r"

Û"□Ai&~Ê^9cì(□VŠå<□ã9<Ys¬A.‘>˜útë□Žâ@Ò0eÜ
()ÇlŸ-ut QE□ QE□ QE□ QE□ QE□ QE□ UMKþ<›ê?□[ªš—
üy7Ô□: Ä¢Š(¢Š(¢Š(¢Š(¢Š(¢Š(□¥_°>”´‹÷GÒ¸+□^:ÕÝÍÀð×,5□ZÒÞF•®å¸KTr§□ËÞ
q•y

úŠç<ã□O□ØÜÉ□µÅ•åœÆ
»;□‰!□v>£ßØÔ-'ñÅ¾□¨[é6z}Ö¯-Ü'™□… □,tÞìxEÏs@□U□Èè>'ñ%ö-□-
µà›-&)UŠ\-äw1,□pÛq·?Î°ê (®?Ä^<þË×□@Ñ´k-sZòÄÒ[[°Æ□¡èd'¸\öÿ ëŒÃ¡xþ{¿□Gáï□
ø~çAÕgF'Ù$™fŠp¼°Y□ •9Å vÔQE □QE □QE □QE □QE □QE □QE fêÿ v/©þ•—
Zš¿Ý‹ê□¥eÐ□E□P□E□P□E□P□E□P□[Ö?ñå□ÓúÖ□oXÿ Ç"_Oë@□(¢©êÚ□¶‹¤ÝjWeþÏm□'ö)f
v u4□rŠó‹Ÿ‰-#µ´mR_‡Z¢hè¦G•î£□ª

¬aûÃŽy5Ûi°Ý†- Á-ÚM°Æh|õr

Bã'#Ôs'í@□4W□Ÿ^¾
¼€ßèß□µ[Ý(□Ââ[„,I□ûÉ□□˜-ÞµÕø[ÄÚ□ ´□u}4¿"!*ÑÈ0ñ¸ê¬;□@□4Q\G‹¾‰éþ□Žö+□Iµ‹û
(üË˜-Ž□Ý}e"□'°äûP□oEVÓ®ÿ ´4ËKÐ>>Ñ
K³9Û¹AÆ□□³@□□Q@□□Q@□□Q@□□Q@□□Q@□□Q@□□Q@□□Q@□□Q@□□Q@□□Q@□dêßëÓýßëZÕ"«□¯O
÷□ gÑE□□5¯ü}Åþø®,¹û_øû‹ýñ]□ □QE □QE □QE □QE □T□——Ú}œ·w—□ÛÛD»¤–V
ª=I5Æé□□ Ö|ce¢Ùé7bÆò &ƒQŸ÷k0N¥□Œ•?Þ8ú□ îhª÷×ÖÚe…ÅõäË□µ¼fIdnŠ dšà#ø"â
û_í=#áö§y£'¹.^ê8¤'?¼°□XƒÛ□h□Ñè¬□

x ›Oñn‡

-¦³ù.J²H0ñ8êŒ;□Z³J–ðI4–¶8Ô»□g

š }□ç□ð±|Cy ¾Ñ¾-ê–š_U¸šá ’EþòÄAf□·-u^□ñ=‡‹ô□u};Ì□HJ´rŒ<n

°õ□□µE□P□E□P□E□P□E□P□E□P□E□P□E□P□□÷ü~ËõÊ«Õ‹ïøý—
ê?•W □Š(□Š(□Š(□°Tû‹ô®j°Tû‹ô □¢Š(¢ŠÂñ?‰âðÕ-»},÷P»°‟Ê¶´³•{ÈØÉö

¤Ð□í□çƒâV£¥ê□pø³Â□z□•ä¢□o~Ô—□‡=□í&□ÏC]íåÝ¾Ÿe5åÜÉ□´□d-G8
d‟@□Q^w□Ä]□W•í~□ð□¡¨é‡˜îî.ãµóWûÈ□’Aí]¶□□6§¤ÛÞ\XOa4ªKÛ\c|d□0qôàúb€/QYúî
¨4O□êZ³Df□V²\□Ãmß±Kc=³ŠâlüqãÍBÆÞö×áŸ™oq□Ë□ÿ o@7+

ƒ,¹□-ô□èÔU=*æöïK·¸Ôl>Áy"f[_8Kå-
MëÁúŠ¹@□□Q@□□Q@□□Q@□□Q@□□Q@□□Q@□soþ±¾¦°Jæßýc}M 6Š(□Š(□Š(□Š(□Š(□□'ý
dŸAZµ•¤ÿ ¬"è+V€
+'ñ□Œîôícû□Eðåö·©,k,‹□,0Ä§8Ý+p
Áâ ðß•¤Õ<Bþ□Öô+•□Yò|ø šU•&AÔ£®□ÇÓ±ô4□ÚQEp-_□àÔ~!'†m4×'ÑÚh—
Ró°•,J□EUÇ d

ç- wtQ\Ï•¼UqáK□>K=$ê—w÷ÑØÁmö•

]Ã□ó□@û¸ç×- tÔW□□Š¼□‰ÄI7Ã_*&p-OíØ□`Ï' s•Jï(¢Š(¢Š(¢Š(¢Š(¢Š(¢Š(¢Š(
¢Š(¢Š(¢Š(¢Š(¢Š(¢Š(¢Š(¢Š(¢Š(¢Š(¢Š(¢Š(¢Š(¢Š(¢Š(¢Š(¢Š(¢Š
(¢Š(¢Š(¢Š(¢Š(¢Š(¢Š(¢Š(¢Š(¢Š(¢Š(¢Š(¢Š(¢Š(¢Š(¢Š(¢Š(¢Š(¢
Š(¢Š(¢Š(®sÇ--µð_…®µ{•¯"•-
ð“þ¶S÷WúŸ`k£¯7ø™ðçVñÆ£¥\ØkQY%€,±K□a¼□w{ô□ŸJ òï□øJëGøKý¿-î“Ä□Ö©□ÅË¸ù£R®U
=°äŽ9Àþ□_HØÿ È>Ûþ¹/ò□ó—Å?
øÛGð¬7Zÿ ‹¿µlÍäh ØF□«a¿

Î½£áþ•â□□DhüA®ÿ jÉ)W„ìÇ”›GËž¦€:Ú(¢€
(¢€
(¢€
(¢€
(¢€
(¢€
(¢€
(¢€
(¢€
(¢€#¸ÿ •i□Ü?Ê¹Úè®?ãÚ_÷□ò®v€
(¢€$·ÿ •˜¿ßÎ°*çmÿ ãæ/÷Çó®Š€
(¢€
(¢€
(¢€
(¢€2µk;X4-bæ+x’ââÙÌÒª Òa□□•S•ÀÍ`ü&ÿ ’Yáÿ úö?ú□V‡‰üM iú~£c{®i¶×†Ùÿ Ñæ»•$
åN>RsÏjç~□x‡D□□x□Ü]cO:—•Wìbå<ì,Ä□™ÝÓž”□èµç7,<Eñ»M6C|-□´˜ÝÌ>è-
eÚ±gÔ□›óïRxçÇÑXj‰ám/S°±Õ§MÓß^Ì±Åc□þ/˜•ò□~USÅ_ð•ÿ ,4□□4m'ÄšMÄòÉ-
oí□¤šêf<³a²ÌÇú□@□•A-•¬÷P]Ko□—□n□ÊÈ
G»□¶ž£8□ÅRÔ<G¤éZ¶□¥ÞÞ,7°‹2ÚÄU□˜W□ä

£®*]O[ÒtT•õ]RÊÁd$#]\$A^ê□â3@□o€?äwñÿ ý„£ÿ Ñuè5äÞ□ñg‡-<aãyî|A¥C□Î¡□Á$—
'ªÊ¡1•$üÃÜW¬□□□□ƒÐŠ Z(¢€
(¢€
(¢€
(¢€
(¢€
(¢€
©©□Ç"}Gó«uSRÿ •&ú□ç@□"QE □QE □QE □QE □QE □QE t«÷GÒ¸ß□üAo□ÞÜZ/…<G¨4H□\ZY
n·lŒãÌÏn‡Ž+±□ œ□•Ú¹□/â—ƒuKS)×-
¬dN$·¿qo,l:©V<Ÿ¦h□3áI·Ôa×<H/í'¾ÕîÄ·□[□‹P£□□□·□œ'□{z×9áÿ □j1x×Æ—
Zo…¯õ½MõF´ó□Ö(b†□±PÊÜ□œœ□èkcÀóÛë¿□¼Mâ=□□hr[ElÓ„*—W
r]}p23ïïQx□\Óüãï□hšõÔv□êW§T²¹¸m'J$8Üx□□Ž} nh¿□n'ñ□-ñ'‡n´□FéY-|É-
x§Ú2BÈ¸□±ÛüF{Šò½cW°ñ×Ä□
Ùxzd¾‹G¸këëØ

è¢□p¨□pK□Ûüqèâ=%<Hž-kÅ□«Áö…·ÚÙ1äŒç□ìxÎh□Ï4}sNð‡Å□□ÛøŠæ;□ÕZÞâÎêäìŽXÕ□+¼ð
0N9ô5Wâ'Œ<9©ë-□•GÕ-ou;mrÝÇÙ_Ì□&%\□_—"·Œ×q®xƒÀòÞÏ£ø†ûF3Û`½¾¥°□Ü¡•-g□,:W-
□Ñ<câÍKð••ªèZ5âê7÷–¶â8Zd□E□• c–
$öÅ zÍ□Q@□□Q@□□Q@□□Q@□□Q@□□Q@□□Q@□°¿Ý‹ê□¥eÖ¦¯÷búŸéYt QE□ QE□ QE□ QE□ Võ
•üyEôþµƒ[Ö?ñå□ÓúÐ□Š(¬Ï□k¶ž□Ñ'Õ¯’vµ·+æù1ïe□,îÇ ÎO°4□çž*ø-4:]Õ¬>□×l□`Ð}»V
²h-¢Ï□É]Ä□×□æ»_□évz/,4□>Âö;Ûh □nc ¬¤’Å†;dš£?ÄÏ□□&K¹<G¦ËnP–‹Ì#

tò¾÷áŠËøK□š7Ã¹nïãk□).n/`Šn>ÏlNW>ƒ ·ãš îu-F×IÓnu
é–□[hÌ’ÈÝ□•\oÂK
›□ \ê7P□wÖu õ4…†

i!□Gä þ5Í□ÂS |CÕ-}g_Ò¬</g6è4û‹Øã-þE<I2-
ÊÆ□D=zžÕê:^¹£ë"Oì□VÆøC□'Ù.□]™Î3´œg□ò
â¾$ÙÚÙü0ñ7Ù-â‡Î·’Y<´
½É□±ÇR}k¡ÑüG¤ëÒßG¦^-ÃØNmî@V-\ƒ¨ä

ýFEr□|M □ø‹L□æšu□³´□eû\~nì•—fsŸlP□aáÏù□ôŸúò‡ÿ @□§\×ƒüC¢jZ.›ec¬i÷WqYDd,
”y□□Pr ä`œWK@□□Q@□□Q@□□Q@□□Q@□□Q@□□Q@□□Q@□□Q@□□Q@□□Q@□dêßëÓýßëZÕ“«□
¯O÷□ gÑE□□5¯ü}Åþø®,¹û_øû‹ýñ]□ □QE □QE □QE □QE Cwgm□lÖ×-ñ\@Ä□ŠT

§□□□xà€□
àõ □Ç
•ÀþÍ°®îîò×O´’êöæ□khÆ^iœ"(õ$ð+Ëu□□xr_Œ~□¾□Ä□SÙÃ§Ü¤—
y□□□ô□³€O¥ kügvo□-
•b□úþÚÚL□|¥Á?ú□z□q¤1$Q HÑBª¨À t□¸O^ö£Åß□n®<=q□ü°:]Ú½³‰VF‰òÁJ““€Ã□¿□bÃâ¿
ƒ.ô□Ôæ×1íO—°[Yd□hÛ□®Ï¼H<p

{P□□ÃÔ□~:ø□¦Ä6ÛÇ¨Crª:□•
7ò□èµçß

`¸¸<Ä-,½□íF¹zn ŽQ†[d□Œ·¡#?†□zé,ü_£ê~□¹ñ

—4—ö□é#‟□M½¶□YUX□O□zÐ□5âoŠ□h2^Á„<E3[; °’Ì¥«cø¼Á‟·¾BÕÏ…v□¶~
I-µ+mEï®$¼¸ž×>_šç,ª□#□□□□ÇAV->(x&óOûbx—
O□1‟□Ò^ä-Û□□Ÿ ¬O„ñ‹‹Ÿ□ëvVïo¢jz□›`Œ…□€0Ò*ö

qù{P□¤ÑE□ QE□ QE□ QE□ QE□ QE□ QE□□ƒ}ÿ -
²ýGòªõbûþ?eú•åUè ¢Š(¢Š(¢Š(®•>âý+š®•>âý(□h¢Š (¢¹•□øÚÃÁZLw□;d»¹□*ÒÝ¤□‰
_ý¦<*ŒŒ±àP□□ÅÆ□Æ•§x>ÔyšŽ³w□T□˜¢F

òŸ@1úš>4ß
_□ØÚÈ‟Io□ªÛZÜG□–‘ãÉvU□©;1Žõ„õ
é—7□æ¹ã=□÷ÄWÊ□ó®¡□È□¨† [„™<š·ñRÞ]KÀö°Æ”□áÓ/-
õX„'p•□òTŽ£k□ŸA@□¿Ž¼Ummö^>□ê#L‰x&ò%™Pz@□=;WaáÏ□Øx«AµÖ4Çf¶¸□€ã

„□□aØ,□¬□ø¯à•Ðÿ µ□¿hÈcÞ-ÕÁœœ}ß/ïg·L{Õ□†° ·…þ□K©khl#-
Yõ□cpso□□€@□û □c<úÐ□Sâû□□OÁzå…œ~eÍÎŸ<0¦@Üí□ 2x□'½pú_ŠüiáÏ□X[j

/□ÖÊÖ8^kmF)¤!□□Â%□íœf»i|] Áá¨¼G.¢‰¤J□¥ÉFÁ

vŒŒdrqÈã½gÞ|LðU•<Ý¿‰tÙ□W>\□
$•ì□I9ü(□_Ã¾!Ó¼S¡Ûêú\¦KY□Æá†B

°ìA-Já>□i·v>□»»¼µ{FÕ5□õ□íœa¡ŽB6©□¸\ãÞ»° (¢Š (¢Š (¢Š (¢Š (¢Š (¢Š +›□ô•õ5
ÒW6ÿ ë□êh□´QE □QE □QE □QE □QE hé?ë$ú
Õ¬-'ýdŸAZ´□fâo□ÿ Â5□¼ŸØzÖ«ç□□t»O<ÇŒrüŒ□ž>†¸O

kpxçâŒzµØ□\úE¬□ÚéW!’éËýé□□□1Æ□¶:’+°¹ø‡á□?ÄWZ□§¨¦□{o´æðyQÈ¬¡ƒ#Ÿ”ŽqÉ□ƒ\‡ˆ
5}+Æ□¼%□†gŠúóM¹k›ËÛS¹!·Ç(\pwtÆ□• u-;Öï
,ÓÃ°#ã\ÖXÃ□□ùvˆ□¬œú□□=Èô®~]□ÏÃ¿□þi6
²ÚÚÊù□=Xì□cîNI÷5¡«x□Ä7>5½ñ&‘ã?ìÉnaH□6Òã¸1F |ªÎÜ□Ùc€9=ñ\†µáß□Å
ñ7Âö³øïÎ¿š
³o{ý‘
ýœ□□†ÀpÛ‡□=(□Ûëˆø—§k□¶z□Î‰¦□JçNÖa½{q2Å¹□_?3p9 wëÒ¶ïuÛ
éZhñ

¬¦iš;O´˜Jùó□×j,□$□è=iúÏ‹4□]Û[k□¥½Œ·Aš<íV
ŒüÝ□QÔÐ□%7ÄÍ[Cxåñw,¯´{□p•{□Ò]Ç□' ¾À6ŠôHäIcY#`èà2²œ,□B+Í~!xãÃúŸ„ï´□-þÛYÕ
uH□--¥Œ,rY¸ÜJä(□y=«»Ð,$Ò¼9¥éÒ¸ym-"•Ø□□D
Oé@□4QE □QE □QE □QE □QE □QE □QE □QE □QE □QE □QE □QE □QE □QE □QE □QE □QE
□QE □QE □QE □QE □QE □QE □QE □QE □QE □QE □QE □QE □QE □QE □QE □QE □QE □Q
E □QE □QE □QE □QE □QE □QE □QE □QE □QE □QE □QE PÕô]3^²û-«e□å¶ðþ\«•□t?^µy
T*…P□□€□jZ(¢Š(¢Š(¢Š(¢Š(¢Š(¢Š(¢Š(¢Š(¢Š(¢Š(□;•øö—ýÃü«□®•”2•#
Œ□P}ŠÛþy- `Ñ[ßb¶ÿ žKGØ-¿ç’Ð□-¿ü|ÅþøþuÑT
gn¬□D□□ Ôô QE□ QE□ QE□ QE□□‘□å_

ê·mw¨è:]åË

×□qÈä

œ°&’ËÂ^□Ónã»±ðö“ks-vM□”hë‘ƒ†□#,ElQ@□□þ□ðî©v×z†•¥Þ\°□¦¸³ŽG

±□ô¨-ü□ákK˜®-
¼5£C<L-9c±‰Y□□,□\,□zÜ¢€+O§Y]][Ý\YÛÍqlI,i"Vx‰ë´‘•ÏµC©èšN´‘¦«¥Ù_¬d”[«t”!=
q¸□Uú(□Ÿÿ „□Áÿ ô*h□ø.‡ÿ ‰-ð □€:□KE □QE □QE □QE □QE □QE □QE □SRÿ •&ú•çV
é²F’¦×]Ëé@□Ý□½ö+oùä´}ŠÛþy- `Ñ[ßb¶ÿ žKGØ-¿ç’Ð□□□½ö+oùä´}ŠÛþy-
 `Ñ[ßb¶ÿ žKGØ-¿ç’Ð□□□½ö+oùä´}ŠÛþy- `Ñ[ßb¶ÿ žKGØ-¿ç’Ð□ë÷GÒ³/¼7¡jw-
hÔ4]:îoùéqj’7æA5©E 2□b·…!,$Š$□TE
ª= □*¶¡¤é°¼"□KOµ½‰NB\Â²(?F□®Q@□¬të□2Ü[éövö□□Ÿ.Þ%□sô

•du□¨ ›;s|±ùBäÄ¾`Lçnìg□íVh

íGÃú.¯"É©èú}ìŠ0-sl’□ô,□\¶µ·³·K{X",□□Xâ@ª¿@8©h □Š(□Š(□Š(□Š(□Š(□Š(□Š(

Ý_îÅõ?Ò²ë¢–□æÇ˜•±Ó5Ø-¿ç’Ð□□□½ö+oùä´}ŠÛþy-
`Ñ[ßb¶ÿ žKGØ-¿ç’Ð□□□½ö+oùä´}ŠÛþy-
`Ñ[ßb¶ÿ žKGØ-¿ç’Ð□□oXÿ Ç”_OëGØ-¿ç’ÔÈ‹□□Q…□□ :,□□<Š(

„ð¯‡c¼□‰ ékt

ï8YÆ>»±šÔš□î!'□£I"'J::,¬§,□=A§Ñ@□ÿ ü ž□ÿ ¡SCÿ Át?üMhiš□'¢
F•¥XØy¸ó>Ën'oÆq• g□?□hQ@□-4ë+□□¬ìííÚâC,Æ□•

Žz³`r}ÍfÝx;Â÷·R\ÝøoGžâV-$²ØÄÎçÔ’¹&¶è

½;Ã:

•rntÍ□M±œ©C-
µ¤q1SÎ2 □p8ö-J(□Š(□Š(□Š(□Š(□Š(□Š(□Š(□Š(□Š(□Š(□Š(□²uoõéþïõ-j
ŽKx¦ È□ˆãš çh-ï±[□İ%£ìVßóÉh□-×þ>âÿ |WAP-¥°0e^□

A©¨ ¢Š(¢Š(¢Š(¢Š(□
»;]BÒKKÛhnm¤□xf@èÃÜ-□c□Â àÿ ú□4?ü□Cÿ Ä×AE W±Óì´»D´Óí-í-
“;a·ŒF‹“”… □É&©Ká□□kï·K¡é²]“»Ï{HÌ™õÝŒæµh €F□â«XéöZe¨µÓìíí-
Ô’"·ˆF€žIÀ Uš(□&□
xzêìÝÜh:\×$çÎ’Î6|úî#5ªª□B¨□@À□ ¥¢€
(¢€
(¢€
(¢€
(¢€
(¢€
(¢€
(¢€0o¿ãö_¨þU°□µ,G,ñ,Ç©¦ýŠÛþy- `Ñ[ßb¶ÿ žKGØ-¿ç’Ð□□□½ö+oùä´}ŠÛþy-
`Ñ[ßb¶ÿ žKGØ-¿ç’Ð□□t©÷□éP}ŠÛþy-
X□□□ QE□ U□OCÒu¥□u].Êýb$Æ.-ÒP„õÆàqÐUú(□Ÿÿ „□Áÿ ô*h□ø.‡ÿ ‰-È
†ÚÞ;{x’(bP‘Ç□…TP0 □€ íRQ@□Qøc@ŠûíÑèzb^nÝö…´ŒIŸ]ØÎkFx!°·’Þâ$š□T¤‘È¡•ÔðA□
,*J(□°XYÇ`¶1Ú@-j»□Ýc□0¾□qŒ{U
o
xrÎè]Zè□T□ äK□œjàý@ÍkÑ@□□Q@□□Q@□□Q@□□Q@□□Q@□□Q@□□Q@□soþ±¾¦°J€Ù[“”□æ€0(-
ï±[□Ï%£ìVßóÉh□□ŠÞû□·üòZ>Åmÿ <-€0h-ï±[□Ï%£ìVßóÉh□□ŠÞû□·üòZ>Åmÿ <-
€0h-ï±[□Ï%£ìVßóÉh□-“þ²O -ZŽ8"„“□□'®*J §¨i□f¯□Ç©iÖ—¨½□æ□•□Á•¥°Ó4ý*□□•cmg
91ÛÂ±®~Š□[¢€
,K□I¯!¼-Ö□°€2Ã3F
Æ□ï□n£=ñÖ§¢€+^iÖZŠÄ·Öv÷K□,XÄñ+„qÑ†G□zÒ_i¶□¤"□BÊÚî!ÎËˆ-
Eüˆ5jŠ ÎÓ´□□Gfm3I°²f□cml‘“õÚ□hÑE □QE □QE □QE □QE □QE □QE □QE □QE □QE □Q
E □QE □QE □QE □QE □QE □QE □QE □QE □QE □QE □QE □QE □QE □QE □QE □QE □QE □
QE □QE □QE □QE □QE □QE □QE □QE 5Ûb□ô¨~Ð□*Y¿Õ5T ¾Ð□(ûAþè¨h
¾Ð□(ûAþè¨h ¾Ð□(ûAþè¨h ¾Ð□(ûAþè¨h
¾Ð□(ûAþè¨h ¾Ð□(ûAþè¨h ¾Ð□(ûAþè¨h
¾Ð□(ûAþè¨h ¾Ð□(ûAþè¨h ¾Ð□(ûAþè¨h
¾Ð□(ûAþè¨h ¾Ð□(ûAþè¨h ¾Ð□(ûAþè¨h
¾Ð□(ûAþè¨h ¾Ð□(ûAþè¨h ¾Ð□(ûAþè¨h
¾Ð□(ûAþè¨h ¾Ð□(ûAþè¨h ¾Ð□(ûAþè¨h
¾Ð□(ûAþè¨h ¾Ð□(ûAþè¨h ¾Ð□(ûAþè¨h
¾Ð□(ûAþè¨h ¾Ð□(ûAþè¨h ¾Ð□(ûAþè¨h
¾Ð□(ûAþè¨h ¾Ð□(ûAþè¨h ¾Ð□(ûAþè¨h
¾Ð□(ûAþè¨h ¾Ð□(ûAþè¨h ¾Ð□(ûAþè¨h
¾Ð□(ûAþè¨h ¾Ð□(ûAþè¨h ¾Ð□(ûAþè¨h
¾Ð□(ûAþè¨h ¾Ð□(ûAþè¨h ¾Ð□(ûAþè¨h
¾Ð□(ûAþè¨h ¾Ð□(ûAþè¨h ¾Ð□(ûAþè¨h
¾Ð□(ûAþè¨h ¾Ð□(ûAþè¨h ¾Ð□(ûAþè¨h
¾Ð□(ûAþè¨h ¾Ð□(ûAþè¨h ¾Ð□(ûAþè¨h
¾Ð□(ûAþè¨h ¾Ð□(ûAþè¨h ¾Ð□(ûAþè¨h
¾Ð□(ûAþè¨h ¾Ð□(ûAþè¨h ¾Ð□(ûAþè¨h
¾Ð□(ûAþè¨h ¾Ð□(ûAþè¨h ¾Ð□(ûAþè¨h
¾Ð□(ûAþè¨h ¾Ð□(ûAþè¨h ¾Ð□(ûAþè¨h
¾Ð□(ûAþè¨h ¾Ð□(ûAþè¨h ¾Ð□(ûAþè¨h
¾Ð□(ûAþè¨h ¾Ð□(ûAþè¨h ¾Ð□(ûAþè¨h
¾Ð□(ûAþè¨h ¾Ð□(ûAþè¨h ¾Ð□(ûAþè¨h
¾Ð□(ûAþè¨h ¾Ð□(ûAþè¨h ¾Ð□(ûAþè¨h
¾Ð□(ûAþè¨h ¾Ð□(ûAþè¨h ¾Ð□(ûAþè¨h
¾Ð□(ûAþè¨h ¾Ð□(ûAþè¨h ¾Ð□(ûAþè¨h
¾Ð□(ûAþè¨h ¾Ð□(ûAþè¨h ¾Ð□(ûAþè¨h
¾Ð□(ûAþè¨h ¾Ð□(ûAþè¨h ¾Ð□(ûAþè¨h
¾Ð□(ûAþè¨h ¾Ð□(ûAþè¨h ¾Ð□(ûAþè¨h
¾Ð□(ûAþè¨h ¾Ð□(ûAþè¨h ¾Ð□(ûAþè¨h
¾Ð□(ûAþè¨h ¾Ð□(ûAþè¨h ¾Ð□(ûAþè¨h
¾Ð□(ûAþè¨h ¾Ð□(ûAþè¨h ¾Ð□(ûAþè¨h

¾Đ□º(ûAþè¨h ¾Đ□º(ûAþè¨h ¾Đ□º(ûAþè¨h
¾Đ□º(ûAþè¨h ¾Đ□º(ûAþè¨h ¾Đ□º(ûAþè¨h
¾Đ□º(ûAþè¨h ¾Đ□º(ûAþè¨h ¾Đ□º(ûAþè¨h
¾Đ□º(ûAþè¨h ¾Đ□º(ûAþè¨h ¾Đ□º(ûAþè¨h
¾Đ□º(ûAþè¨h ¾Đ□º(ûAþè¨h ¾Đ□º(ûAþè¨h
¾Đ□º(ûAþè¨h ¾Đ□º(ûAþè¨h ¾Đ□º(ûAþè¨h
¾Đ□º(ûAþè¨h ¾Đ□º(ûAþè¨h ¾Đ□º(ûAþè¨h
¾Đ□º(ûAþè¨h ¾Đ□º(ûAþè¨h ¾Đ□º(ûAþè¨h
¾Đ□º(ûAþè¨h ¾Đ□º(ûAþè¨h ¾Đ□º(ûAþè¨h
¾Đ□º(ûAþè¨h ¾Đ□º(ûAþè¨h ¾Đ□º(ûAþè¨h
¾Đ□º(ûAþè¨h ¾Đ□º(ûAþè¨h ¾Đ□º(ûAþè¨h
¾Đ□º*u;”-
Z¥W#ÿ V¿J u□Q@□□Q@□□Q@□□Q@□□Q@□□Q@□□Q@□□Q@□□Q@□□Q@□□Q@□□Q@□□Q@□□Q@□□Q@□□Q@□
□Q@□□Q@□□Q@□□Q@□□Q@□□Q@□□Q@□□Q@□□Q@□□Q@□□Q@□□Q@□□Q@

›ýSUJ·7ú¦ª” QE□ QE□ QE□ QE□ QE□ QE□ QE□ QE□ QE□ QE□ QE□ QE□ QE□ QE□ QE□
QE□ QE□ QE□ QE□ QE□ QE□ QE□ QE□ QE□ QE□ QE□ QE□ QE□ QE□ QE□ QE□ QE□ QE
□ QE□ QE□ QE□ QE□ QE□ QE□ QE□ QE□ QE□ QE□ QE□ QE□ QE□ QE□ QE□ QE□ QE□ Q
E□ QE□ QEW¿¼•OÓîofÿ Uo□Jøþê,Oò

¿□xÃ@ð¤HúÖ¥□±□,ïî□A8÷é\å•Æ□□ß\ˆ?µ^Ý™¶«O□*"õÆ□Ôâ¹?…þ□·ñÅÆ¡ã•□B—÷□□,-
ÐL»¢E s´ð@ÎÐ□M¿Lwú¶ƒàMZÕìïítR¸Û•1ÆéôeÁ□□ÕE,sD'Äë$n¡'Ôä0=□=Å:¼÷â-½sðïáíŒ
ž□□†Xm"ó□□□ÂŸ~N□sT<-â?‰> ÔôýRm□ÂÛÃ·o'…€'"Ç□ËdöÇ□õÆ

h□Ôh¯0Õ¼Mñ□XÖï¬ü) Cmce3@nõ□"ÌÊpJ,GÊ{`¨Î*÷ÃÏ□ê°þ§ªx□Äv□YëZv□Ä_u×8'□=
□ƒƒ»Š ØðïŽl|Gâ]oD·µ¹Šm&S□'H□×!Šœc§#¿jêk€ðO‰ïuŸ□øÃM¹ŠÕ!ÓîDq4Qm
v□™~cüG W'7Äß□Ýxß\ðÎ‡¤Úß\EtÑÛ3)
□hHf~@9ùy$

ö9□€=®Šòï
øëÅ□¾7‹Â^4°¶Šêê#%-Å·FÀ'žH í t ŒwâÇŒ¼mâ(ü_□„¼!cm6¢ÐyóMr~T_@ □¦

yëÒ€=&Šãü□□åYæ½µñ~™k□„!,æÝ†ÙsœŽ□ôÀô®Â€
(¯□ŸâOŽ/üq-ø_@Ó¬îg†éÒ □p ‰ □›œ□ü¼Ÿ □# -ÙEy~«ñ□Ä^□ð¥”zî—
□Þ*¾□àµ´·;‘À □;Iõ□□“žÜã.ûÅ?□ü;fÚÎ-¡é³iñáç,□
D™çî±?□ÍŽô□ì”W*Þ>ÒWáðñ‰/ö#

ññ—Îß/ë»□Ný+†´ñGÅÍ~Éu-+BÓ`Óä-d□JFùS±ù˜□Ç□-
=E {□8□5CIÖ´ÝvÌÝéW°Þ[«˜Ì•¶à□u¨üëÎôß□ø“Æ¾

»-´µ²ñ-Œë□õµÖvª□Ù+ž„'Œ-˜>ÕÅ|ÿ „÷û
□`□g□c□hŸ´ý£□÷a7ã¾6â€>^¢¼ÛÆ^6ñ□~/ƒÂ^□±¶›Qh<ù¦¹?*/ □•Ó□<õé[>□¿ñ¬ó^Úø¿Lµ€
Â□ÁsnÃl¹ÎG

z`zP□aE□•^HÞ(ø¥â&’÷ÃÞ□¶°ÓU□’—øY¦□Ô1□ÏÐ□s@-·Eq_□üo?Œô«¿í
E´Õ,&ò.¢L…Ïb□ät#□=+Ït□‰¿□üW%Ö›¡é63ÝÅ34—n»cŠ>Š¼œg
÷$ŽÜf€=ÞŠóŸ□xëZÔüG¨xWÅV□Úë6q‰CC÷dN:òF~`A□□{c˜|[ãí|ø¿þ□□iÐ]êqÆ$°š□□ -
P8□rNzã□ □Ï□xÒ_□ÜhqEd—?ÚW,ÝË9]ƒŽG□y®Â¾qñŽµã
•□xgJñ~—□¼ðj ,76ÿ re, ô$□=±ŒŒŠú:€(_kZf™ygi{}

□□®c¶ŽFÁ•¸à~có□~¾|øŸÿ §ü,_□ùßaÿ •ƒ□blÿ }1ægþ□ŸÆ½;K¾ñ–
™àÍnÿ ÄâÀê□°Ë5°·□\,d•Ø÷□□ÚÑ\§Ã□□_x£ÀÖ□¶¢c7S□□˜×j□®@ãè□gøOÅ°žµñ□ÅZ-
×“öM6E[}‰†□$□œó@□þ-xÒo□iw×“Y%©¶»h□QËd □'#¯5ØWÍŸ□µß□Ei©h¾□Ò`žF»iç¼¹?$@€
ä□ž=ÉçŽ3^‘àÏk²ø¶_□xÇO†ÓVòÌ-
òÃ÷f dŽ¤-2r=□ÆE z]□æþ,ñæ°|T|)à½2□ÝR(Ä·SNq□*@ã¨□ärO|`š¡¦øïÆ--ñ>•£xëM´H5'
ò-¯-O□ò□8c

€Œçš õz+Ê¾$|E×|#ã-/KÒ¬à¼Žê×p••—
’VfU Žz€p:ò;ñ□Jñ□Ž4=+YÕ|m§Y¥□-¯Ú!û3®æ□uæpO·8üM z5□ãv-*ø·ªØC¯Øèš\°tÃ|vÀ•
ì™ë÷óúþ□ìQ³4HÎ›□¨,¹Î□¦h□#Åzü^□ð¶£¬Ê□û4E‘OFsÂ¯âÄ
Ìðç^®âøumâ/□M

.mÍÔÍ□`,då8îJ•ãÔâ¹?ŽSÉs§ø{Ãñ³+jz€□Žáp1ùÈ□á]Å-*âÿ áv-c§@]Ò(Ù!ŒuTub ö
N=¨□¦™ñ^ÂóQÓ ½Ñu]2ÛTm¶□wQ□□ÄôèxÎG¯QÛšê¼Kâ+□
è7:¾¢äC□áWïHÇ¢¨îOÿ _ ¯□ÖõÝ;Žž□ð7‡t□ŒÚœs[´É□6m„iµËqÀ□œú.kbYâø£ãÃ,ò¢ø;@"□
¤`#¼¸úž□üþ_Mô□é□Ú□öµàá©iö-g}shÒÛCt□Úä□›°z-□ÐÖgÃ□□Ëã□ G}v¨—
ðÊÖ÷H«´□^àPAúæ°ÄttWFVB2
œ,+Éü□ÇGøÉã]□□ÕO‹å□—$□□Âaù
õª(¢€
(¢€
(¢€
(¢€
(¢€
(¢€
(¢€
(¢€
(¢€
(¢€
(¢€
(¢€
(¢€
(¢€
(¢€
(¢€
(¢€
(¢€
(¢€
(¢€
(¢€
(¢€
(¢€
(¢€
(¢€
(¢€
(¢€
(¢€
(¢€
(¢€
(¢€
(¢€
(¢€
(¢€
(¢€
(¢€
(¢€
(¢€
(¢€
(¢€
(¢€
(¢€
(¢€
(¢€
(¢€
(¢€
(¢€
(¢€
(¢€
(¢€
(¢€
(¢€

(¢€
¹úµúU:¹-
úµúP□¨¢Š (¢Š (¢Š (¢Š (¢Š (¢Š (¢Š (¢Š (¢Š (¢Š (¢Š (¢Š (¢Š (¢Š (¢Š (¢Š (¢
Š (¢Š (¢Š (¢Š (¢Š (¢Š (¢Š (¢Š (¢Š (¢Š (¢Š (¢Š dßêšªU¹¿Õ5T □Š(□Š(□Š(□
Š(□Š(□Š(□Š(□Š(□Š(□Š(□Š(□Š(□Š(□Š(□Š(□Š(□Š(□Š(□Š(□Š(□Š(
□Š(□Š(□Š(□Š(□Š(□Š(□Š(□Š(□Š(□Š(□Š(□Š(□Š(□Š(□Š(□Š(□Š(□Š(
 □Š(□Š(□Š(□Š(□Š(□Š(□Š(□Š(□Š(□Š(□Š(□Š(□Š(□©êÖ#TÑ¯´òÛEÕ¼•□é¹
Jÿ Z¹E x÷Á□~□□>óÁÚ›‹]ZÊêM•ÊB—□ª•R□9-„{ÖwÄŸ…>□ðï□µ]kO†æ;¸Lf=ó-
Q°UR0zðÆ»ï□ü/ðçŒn~Ùy□¶×ø ÝZ°W|p7□□?\gŽµÎÁð#Ei‛µ=kW¿Š3òÄò€¸ô<□•¦(□□âü•□
},¿ôKWµY

X[•ÓÊ_ä+•ñG•4ï□xV×ÃÞl¶Vv¯□Cäà•□¥BüÙÈÁ®š(ÄQ$kœ"…□ö □□°Öüyñ3TÕ$ðþ³k¢é□sù*
¥A•°à¯ž Î□¨–™æ«|,,òÛã/ˆàÔ5TÕnã±+-Ú□‡!ãþ]?
éõŸ,z-¥¬\ê□šŽ¡¦›¦-<6î66y8ã€OnEjø?á†‘à-vçSÓ.nßÎ¶□þTÌ□

T–È□’TNOà□Îü2ÿ ’Ÿñ□þ¿□ǒw£á—ü”ÿ ˆ?õùÿ ³½vž-
ðe§‡¼E®k□\Í,°¼¢Y#p6ÆrN□=Ú□□ø2ÓÃÞ"×5ˆ.¦–
]^Q,‵¸□c9'□-í@□gŽ□>7ø□Ž¥\gñ5¿ã/‡Ö¾*Õ!ÕtýZ}+^³@‹unÙ;y
2,□sÎG□¿□©¬x6ÓXñv□â)nfŽ},0H”□¯Ÿ^õ…âŸ„šg‰<A6¹-«¨é÷Ó…□5»□§j…□□Èà

ý¨□¯€<Uâ#ã□WÁ~&-
ÞòòÂ□:^À1¹~^□ g‡^Àðsšô^§†bâ)RB□µÂ°;O¡ô5Ëø3áî□à…¸’ÄÏqys□5ÕÃ□v□p08□çß¹8□ž
ð□•,gÕe³»¹¸:„Šî' ì
» `s÷Ï?J ëkÉ¾□□ÿ
Câ   ïöÏý□ëÖkšðÿ ƒ-<=â-sX,æieÕå□É□□¶3’p1îÔ□ÁüBš-3ãWƒu-
@„ÓÌ~X‘Î□dÜÜ“Û□Ðçµz'Œï¬ôÿ □k7□®‹□ØåB□à9e (÷$□øÒø§ÂzOŒt“§êÐ³
;£’6Úñ7L©ÿ □□jám~□èIq□¾ÕµKëHŽcµ’P□-‡□8úb€8K½:ú?ÙªÆVWòÿ ´¾ÐÀŒb"Ì£ðÜGæ+ß<
;□e□á□:öÊHÍ£Û!B¤a@QÁôÆ0GlU™´□>ãHm&[HŽžÐù□ßn□f0□□Ó□N•ærü□Ðüé□ÛZÕíì¤l½²Ê¤
lãùƒ@□~□2j_□¼wªØ□Ú|“□G^VF,Çp=ó,□âB-þÏÌ□€¯@##S“?÷î:ô

xoKð®‛-™¤Ûù6êw1'-
#-¬Ç¹8þƒŠâ□áIhQø€jVÚ†¡omö…¸6Q¸□îVÜ ã ?•½ iøËáõ¯ŠµHu]?VŸJ×¬Ð"Ý[¶NÞH

ƒÜó‛ÁïÅfx□Å^">1Õ|□âimï/, □¥ì□□—
åá°□xuì□□9«^)øI¦x‟Ä□k‛ê°ŽŸ}8Q#[¸Úv¨Q□ŒŽ ïÚµ¼□ð÷FðBÜIbg¸¼¹Àšêáƒ;

¸ □□

óïÜœ
 èõ
ÈôíбêúPÍ□´/3□êB,N?*ñ-□çâ?Äk   uËO□Yè°a•–
(#\²□ë‟Œþ$þ W¶:$±´r(d`U•†A□±¯+¸ø□ =Ü¦ÛUÕml¥mÏi□ ¯Ð□:□|Ð□□À•
Ô|b%»KÉ□Ü[îPåf9—
.□pzþ5wà'ü‹ZÏý„Ÿÿ AZë|□à□7ÀÒjgMžwŽúE□R□”□v□-§ï-OµOàÏ□Zx*ÂîÒÒêk„¹¹k,e□+
•□8úP□□ƒ´´¸ã:PÏ¿Ê*□□K□—Ç/□ÙÞ□·—¼ƒwVL†Àÿ €•8ô-ÕÝ□□Ú□-
·‹¾Ó7ÚM¯Ù¼Œ□˜õõéYÞ2øc£xÊö-BY®lu□”(¹µ`
□Óp=qØð□J å~3_Ú>¿àû□¸•®ÓPY^□ÙeRT□GlöúW°W—Ûü□ðý³Ú\-
þ¡%ô□Kp÷2¸f‟□íÆ0□yÏ'Þ½B€‹›â©
ñ□áË1ÀþÑ‹Ÿúé□z□Šãy|□-ÆŠYÞÂuP;‟□V□•|□¥øçN†ÛPi¡’ÝËÁ‹$□BzŽGC□Ÿ ¥ðg,í|□§ÜÛC
{u{%Ô¾lÓ\°%Ž þB€9ÿ ,WpIð°ÉVUÍ¼³$¼ýÃ¼·›œ□k□á□å¶¡ñOÇ7V‟$ÖòJ
H‡*Ãqä-ãÞ®^ü   ðõÆ£‹ö°†¥cipÛ¥´,@□û

Ž□×5ÑøCáÖ‟à–SQ»ÒåŸË¼TQ□‡"0¾‡©Éõ

;à□ý§ö^µ§ý¢?¶
æ›É-ól □Àw□ËÖ•â cÔ?hß

Ãfáå³³"ä§;8‛¶·§

?ï¡V□À]□`f‡SÔ¡½óÚU°•À`

0¸Æ8ç'ƒÉö®£Á□

t_□´óÙ□îo®□ÙnîX3•ë´`p23ê}zP□’GáDñ□Æ¯□éwÚÝÞ•tò4Ð□□kL¤†
×û¤□z□jëÓà□„Z□□Õ÷Šu;□"uxÒv3□

□>¸í]OŒþ□h~5š+»Ï>Öþ!µn-˜+□:□□□@Ï×Þ±ôOƒ□F—-Zj×z¶©¨ÜÚH²Â'”□

§#<dà€zÐ□□□€□h?□çþ|ÛùË^□ªZY_éw6zŠ£YÏ□ŠPí´□n:öëX°—ƒ-5/□é~(’æd¸Óâh–
□□cƒ»‟ßø□hø□@³ñF□u£_™VÚä(f‰¶°!ƒ□

□p;P□‟ëZ□‰¾□éRjþ□ñ °Ð ‛LšmòîÚ□€ÂúòÙ%všõí□R]cBÓõ5ŒÄ·–
ÑÜ□'%w¨lgñ¯7¶ø□¢,ñ
ícU½³‰ƒ%«Ê□}

;}1^§

1ÛÃ□0¢Ç□j□□F□€0 -”□äß□Ûìþ,ð□ãñ

:‹□cĐ|ðŸý”×®Wž|iĐ¤Ö>Oqn¤Üi²-â…êUr□òR[þ□]_…u¸üGá]7WŒ©ûT
Î□ ~Ž?□□~□□ÄüJÖ$□□x3ÃPÄ°î·òÏ,j□Š□÷™ˆõ úà□}+œ²ð†—'Å+?□^DgÑô□/ÎŽ□$-
ÄÍ,Ò0□’K□ã t□ßx?ÀòhZÖ-¯j×Ë¨ë:„‡3„Ú±ÇÙ□□qĐ~

ÜÏâo□ÚøƒXµÖ-õ□Ý/V¶ŒÄ—vŒ□(sò°#ž§óï@□ÇÂRÚ□̂üiáè$vÓ4ëáöXË□□†gÊ,yþ□ù-
Z-áïô□ÚGÅ□§(šz)#Ô,
•ÌÊ»•

xgJð6‘zé<Ò□□®¯o®Ÿt’• ,ÇÐsú÷$×□ðfÖmFO□xÂæ2□¬^·’□´jÄœ-
L¶?à□□êÔQE □QE □QE □QE □QE □QE □QE □QE □QE □QE □QE □QE □QE □QE □QE □QE
□QE □QE □QE □QE □QE □QE □QE □QE □QE □QE □QE □QE □QE □QE □QE □QE □QE □QE
 □QE □QE □QE □QE □QE □QE □QE □QE □QE □QE □QE □QE □QE □QE □QE □QE □QE □Q
E □QE □r?õkôªur?õkô □QE□ QE□ QE□ QE□ QE□ QE□ QE□ QE□ QE□ QE□ QE□ QE□ QE
□ QE□ QE□ QE□ QE□ QE□ QE□ QE□ QE□ QE□ QE□ QE□ QE□ QE□ QE□ QE□ É¿Õ5T«s□ª
j©@□□Q@□□Q@□□Q@□□Q@□□Q@□□Q@□□Q@□□Q@□□Q@□□Q@□□Q@□□Q@□□Q@□□Q@□□Q@□□Q@□□Q@
□□Q@□□Q@□□Q@□□Q@□□Q@□□Q@□□Q@□□Q@□□Q@□□Q@□□Q@□□Q@□□Q@□□Q@□□Q@□□Q@□□Q@□□Q
@□□Q@□□Q@□□Q@□□Q@□□Q@□□Q@□□Q@□□Q@□□Q@□□Q@□□Q@□□Q@□□Q@□□Q@□□Q@□□Q@□□Q@□□
Q@□□Q@□□Q@□□Q@□□Q@□□Q@□□Q@□□Q@□□Q@□□Q@□□Q@□□Q@□□Q@□□Q@□□Q@□□Q@□□Q@□□Q@□
□Q@□□Q@□□Q@□□Q@□’4-6ŽEWG□YXd□z,*®™¥Xh¶)e¦ÚÅkl„•Š1€ 9?-\¢€
(¢€!»´·¿³šÒê%šÞd1É□Œ†R0A¦ØØÚé-1YXÛÇom
íŽ(×
£Ú¬Q@□□Q@□□Q@□□Q@□□Q@□□Q@□□Q@□□Q@□□Q@□□Q@□□Q@□□Q@□□Q@□□Q@□□Q@□□Q@□□Q@□□
Q@□□Q@□□Q@□□Q@□□Q@□□Q@□□Q@□□Q@□□Q@□□Q@□□Q@□□Q@□□Q@□□Q@□□Q@□□Q@□□Q@□□Q@□
□Q@□□Q@□□Q@□□Q@□□Q@□□Q@□□Q@□□Q@□□Q@□□Q@□□Q@□□Q@□□Q@□□Q@□□Q@□□Q@□□Q@□□Q@
□□Q@□\•ýZý*•\•ýZý(□ÔQE □QE □QE □QE □QE □QE □QE □QE □QE □QE □QE □QE □QE
□QE □QE □QE □QE □QE □QE □QE □QE □QE □QE □QE □QE □QE □QE □QE □ÏZM«ýÑùRÑ@
µ□?*6¯÷GåKE &Õþèü¨Ú¿Ý•-□ ›Wû£ò£jÿ t~T´P□m_î•Ê•«ýÑùRÑ@
µ□?*6¯÷GåKE &Õþèü¨Ú¿Ý•-□ ›Wû£ò£jÿ t~T´P□m_î•Ê•«ýÑùRÑ@
µ□?*6¯÷GåKE &Õþèü¨Ú¿Ý•-□ ›Wû£ò£jÿ t~T´P□m_î•Ê•«ýÑùRÑ@
µ□?*6¯÷GåKE &Õþèü¨Ú¿Ý•-□ ›Wû£ò£jÿ t~T´P□m_î•Ê•«ýÑùRÑ@
µ□?*6¯÷GåKE &Õþèü¨Ú¿Ý•-□ ›Wû£ò£jÿ t~T´P□m_î•Ê•«ýÑùRÑ@
µ□?*6¯÷GåKE 5¶"-
` □$â«ý¶Óþz□ûäÿ …Mqÿ -Òÿ ¸□•s´□¹öÛOùè?ï“þ□}¶Óþz□ûäÿ …aÑ@□Ÿm´ÿ žƒþù?áGÛm
?ç ÿ ¾OøV□□□¹öÛOùè?ï“þ□}¶Óþz□ûäÿ …aÑ@□Ÿm´ÿ žƒþù?áGÛm?ç ÿ ¾OøV□□□¹öÛOùè?
ï“þ□}¶Óþz□ûäÿ …aÑ@□Ÿm´ÿ žƒþù?áGÛm?ç ÿ ¾OøV□□□¹öÛOùè?ï“þ□}¶Óþz□ûäÿ …aÑ@□
Ÿm´ÿ žƒþù?áGÛm?ç ÿ ¾OøV□□□¹öÛOùè?ï“þ□}¶Óþz□ûäÿ …aÑ@□,öÐ□□ŒŸöMYÚ¿Ý-
•s‰þ±~¢°J M«ýÑùQµ□?*Z(□6¯÷GåFÕþèü©h □Ú¿Ý•□Wû£ò¥¢€□jÿ t~Tm_î•Ê-
Š M«ýÑùQµ□?*Z(□6¯÷GåFÕþèü©h □Ú¿Ý•2FŽ$.ø
;â¤ªš—üy7Ô□ _¶Ú□ÏAÿ |Ÿð£í¶ŸóÐ□ß'ü+

Š Üûm§üô÷Éÿ
>Ûiÿ =□ýò□Â°è □Ï¶Ú□ÏAÿ |Ÿð£í¶ŸóÐ□ß'ü+

Š Üûm§üô÷Éÿ
>Ûiÿ =□ýò□Â°è □Ï¶Ú□ÏAÿ ¦Ÿð£í¶ŸóÐ□ß'ü+

Š Üûm§üô÷Éÿ
>Ûiÿ =□ýò□Â°è □Ï¶Ú□ÏAÿ |Ÿð«;TŽƒò®j°Uû£é@□Õþèü¨Ú¿Ý•–
□ ›Wû£ò£jÿ t~T´P□m_î•Ê•«ýÑùRÑ@ µ□P?*6¯÷GåKE &Õþèü¨Ú¿Ý•–
□ ›Wû£ò£jÿ t~T´P□m_î•Ê•«ýÑùRÑ@ µ□P?*6¯÷GåKE &Õþèü¨Ú¿Ý•–
□ ›Wû£ò£jÿ t~T´P□RË

□ó□\ôâ¢ûm§üô÷Éÿ
¯«ýØ¾§úV] n}¶Óþz□ûäÿ …-
m´ÿ žƒþù?áXtP□çÛm?ç ÿ ¾OøQöÛOùè?ï“þ□‡E n}¶Óþz□ûäÿ …-
m´ÿ žƒþù?áXtP□çÛm?ç ÿ ¾OøQöÛOùè?ï“þ□‡E n}¶Óþz□ûäÿ …-
m´ÿ žƒþù?áXtP□çÛm?ç ÿ ¾OøTÑ´r tÁSß□ÎÖæ•ÿ -Iõ?Î€,í_î•Ê•«ýÑùRÑ@
µ□?*6¯÷GåKE &Õþèü¨Ú¿Ý•-□ ›Wû£ò£jÿ t~T´P□m_î•Ê•«ýÑùRÑ@
µ□?*6¯÷GåKE &Õþèü¨Ú¿Ý•-□ ›Wû£ò£jÿ t~T´P□m_î•Ê•«ýÑùRÑ@
µ□?*6¯÷GåKE &Õþèü¨Ú¿Ý•-□ ›Wû£ò£jÿ t~T´P□m_î•Ê•«ýÑùRÑ@
µ□?*6¯÷GåKE &Õþèü¨Ú¿Ý•-
□ ›Wû£ò¨¥ž□□,„)#=*jÉÕ¿×§û¿Ö€.}¶Óþz□ûäÿ …-
m´ÿ žƒþù?áXtP□çÛm?ç ÿ ¾OøQöÛOùè?ï“þ□‡E n}¶Óþz□ûäÿ …-
m´ÿ žƒþù?áXtP□çÛm?ç ÿ ¾OøQöÛOùè?ï“þ□‡E n}¶Óþz□ûäÿ …-
m´ÿ žƒþù?áXtP□çÛm?ç ÿ ¾OøQöÛOùè?ï“þ□‡E n}¶Óþz□ûäÿ …-
m´ÿ žƒþù?áXtP□çÛm?ç ÿ ¾OøQöÛOùè?ï“þ□‡E n}¶Óþz□ûäÿ …-
m´ÿ žƒþù?áXtP□çÛm?ç ÿ ¾OøT±K

ùòÈluâ¹êÔÒ>ì¿Qýh□Gjÿ t~Tm_î•Ê–Š M«ýÑùQµ□?*Z(□6¯÷GåFÕþèü©h □Ú¿Ý-
•□Wû£ò¥¢€□jÿ t~Tm_î•Ê–Š M«ýÑùQµ□?*Z(□6¯÷GåFÕþèü©h
ïum□"v□‡Q´Ó~Ûiÿ =□ýò□Â²ï¿ãö_¨þU^€7>Ûiÿ =□ýò□Â•¶Ú□ÏAÿ |Ÿð¬:(□sí¶ŸóÐ□ß'ü(
ûm§üô÷Éÿ
Ã¢€7>Ûiÿ =□ýò□Â•¶Ú□ÏAÿ |Ÿð¬:(□sí¶ŸóÐ□ß'ü(ûm§üô÷Éÿ
Ã¢€7>Ûiÿ =□ýò□Â•¶Ú□ÏAÿ |Ÿð¬:(□sí¶ŸóÐ□ß'ü(ûm§üô÷Éÿ
Ã¢€7>Ûiÿ =□ýò□Â•¶Ú□ÏAÿ |Ÿð¬:(□sí¶ŸóÐ□ß'ü*h¤ŠeÝ-□g□Åsµ±¥□Ç«□¾□•
»Wû£ò£jÿ t~T´P□m_î•Ê•«ýÑùRÑ@ µ□?*6¯÷GåKE &Õþèü¨Ú¿Ý•-
□ ›Wû£ò£jÿ t~T´P□m_î•Ê•«ýÑùRÑ@ µ□?*6¯÷GåKE &Õþèü¨Ú¿Ý•-□ ›Wû£òªÆöÐ□
Œ•öMZ®mÿ Ö7ÔÐ□×Ûm?ç ÿ ¾OøQöÛOùè?ï"þ□‡E n}¶Óþz□ûäÿ …-
m´ÿ žƒþù?áXtP□çÛm?ç ÿ ¾OøQöÛOùè?ï"þ□‡E n}¶Óþz□ûäÿ …-
m´ÿ žƒþù?áXtP□çÛm?ç ÿ ¾OøQöÛOùè?ï"þ□‡E n}¶Óþz□ûäÿ …-
m´ÿ žƒþù?áXtP□çÛm?ç ÿ ¾OøQöÛOï•ûäÿ …aÐ:Ð□Kµ□?*6¯÷GåKE &Õþèü¨Ú¿Ý•-
□ ›Wû£ò£jÿ t~T´P□m_î•Ê•«ýÑùRÑ@ µ□?*6¯÷GåKE &Õþèü¨Ú¿Ý•-
□ ›Wû£ò£jÿ t~T´P□m_î•Ê•«ýÑùRÑ@
µ□?*6¯÷GåKE &Õþèü©zQE □QE □QE □QE □QE □QE □QE □QE □QE □QE □Q
E □QE □QE □QE □QE □QE □QE □QE □QE □QE □QE □QE □QE □QE □QE □QE □QE □QE □
QE □QE □QE □QE □QE □QE □QE □QE □QE □QE □QE □Ÿ-ëz□‡t‰õMRámí
□w<ý □É=«B¼wãx:ž³à•

HäYê:-'Qßæ•□þR= <~Ðz□˜%}
YK□m¢èÄ¸ÏÓ8ýkÔô-RË[Òíõ-:án,î□|R/B?¡ìAä□M»Ñì/tItym£û□•□

* *¦0 □±ÛÓ□æ³ÕÔ¯à½BÂW,-5□T□ýÕ*§□Û
ŸÆ€=vŠ(□Š(□Š(□Š(□Š(□Š(□Š(□Š(□Š(□Š(□î?ãÚ_÷□ò®v°+•øö—
ýÃü«• □Š(□Š(□Š(□Š(□Š(□Š(

O□ø³IðÊÂ/åv¸œâ
h□É,§ý•ÌñTôo□Úë□šií¤kZ|ò□Ñý¾ÈÄ¯□“ƒ’:z×□§x·J¶ñß‰u
ËkëýV;£ckogjÓI□□ðHì□6sÏjìt?-iZÞ¨t³□þ•¨í.¶°…¹†GQÔŽH?• Zñ□‹´¿□<□Ý™æ¼¸Ï“ik
□-i=ÂŽßZ©¢øûIÖ5Q¥=¾¡¦ê

¥£¶ÔmÌ/ □vòA¬Ï ¢ê?□ü_ªÌ7Ëo4v0□ÿ ,ÑWæ□êpM'ÅxÖ□

YkH6ÝiwðÏ□ƒ¨□€#èr?!@□íaø‡Åš_†¼„½i¤¹¸$Akm□’Yq×
?®+r²5ÝcEðå°êú¼‘Û¨"□¸òYØg•¿('□N(□7Cø□¥jÚÔZT¶°Ž™□'Í□□□±…¤□®ÞH¯O¯

:æ□ñ□ÆÚ□h’#[é□?kžæB□Û□Â"-
œ,q“€8¯s □Š(□Š(□Š(□Š(□Š(□Š(□ªj_ñäßQüêÝTÔ¿ãÉ¾£ùÐ□%□Q@□□Q@□□Q@□□Q@□
□Q@□□Q@□t«÷GÒ¹ªéWî•¥ -rš¿Ä-□Gñu□†¦[©¯îÞ8÷A□hág'`□’0N
À□ãšÐñgˆáð·‡çÔdŒÍ6DVÖë÷§™¸D-äþ™=«ÍuO

M ¯,¦Ôd□kZ□‰a°Ôgþô¬-ò□öT| tãÞ€=sPÔ-4□>{ûû„·µ□
Ë+œ□□ÂŒš
"ÝK¤ø†-1ˆÆ¦ús
b□}ÙÎ?
OŠJ5□ß□x~Q°ÓQÕÐÜ§i#Œn(}ŽGå^□5´□□²ZÍ□=¼ˆcxØ|¥HÁ□ôÅ %¥Ý½õ¤7v³$Öó ’9□åYHÈ
ÔÕçŸ□Ùâð}î–ÎÎšV«se□c‟µX0ÿ Ð•z□ □QE □QE □QE □QE □QE □QE □QE fêÿ v/©þ•—
Zš¿Ý‹ê□¥eÐ□E□P□E□P□E□P□E□P□E□P□Z‰}k¦hr_^Î□[@-$²¹ÀU□MeÖ½–¼□z_‘s

sBù□□Š□Xg¸<□ âŸã&ƒ□
£¤ø‡û,Ÿù   ÿ f°¶Ç®âs•Â»Û;ÛmBÆ
ÛI’kiÐI□¨r□HÈ"¼×Ä?□¼□6—y¥é·CQ¾¸□í’Ù¢hcÜÀ®□å
¡}y®£Â>□—GøocáÛ‹□Ò–›E$Ñ□€_$í=ÀÝÁö

«□‹ z ¹š=3K×u˜`b’Ýi-
□XTŽ¿6F~£Šé|5â□'Å°_ö†‘pe‰\Ç"2•xÜuVSÐ×›h-;²ø_¢Çá/□ZKm{`•öy-
TI□â□$2àü¬sÎìzçµuŸ□t[ËWÖ¼A□□VÓë×Bém"•:Ã□□\°à±É$Ž9

Ú¸kÿ Š°-µõÍ¥†Ÿ-k-jå.$ÒìLÉ□

¶@ü³]Íq:ßÄ•□x*îm&òìZÝB<Ãi□£ÿ □ÍÁ
·'9ëßš ÝðÏŠtŸ□i□Ú□DæH•Ìr#©W‰ÇUe=□l×Ÿ|/³{‡ñ□ŠJÃ

□õàž
x¥Y6",□b¤□ìI$□q^ƒ@□□Q@□□Q@□□Q@□□Q@□□Q@□□Q@□□Q@□□Q@□□Q@□□Q@□□Q@□dêßëÓýßë
ZÕ"«□O÷□ gÑE□ QE□ QE□ QE□ QE□ QE□ Žë□3»□E□fc€□©®.O‰ÚKÊãMÓ5-Z□Ø«\éö&H•
□~bF□
gÅmEl¼--òÎmàÔ/"´žaÕ"bKŸûåHüj□>#hze„qÚh□êi0
T¹□N"□Aß$ƒ□Â€;□?W´Ô´xõH™£µt.Lêc(□s¸-˜Á®VOŠz6-km7[¼±Œ•×Öö% □êw□8ü*¿Ä=f□O
á°Ë¥Ý -õYá·I£î¬ÿ 7ò ƒî+°´´,ÆÎ□Kh-8!A□h£…P0□ C¥j¶ZÖ›

¡§\,ö³

£¯ê□ìG¡«në□3»□U□$œ +ƒð□k§x□Æ:,#m-µúO□c¢y«’□ àWxè²##¨ea,¤d□é@□L¿□´a¾KM;Z
¿³Œ•÷¶–% \uù‰□~□ÞøKW±×t¡¨éÓ¬ö³ QÀÇ¨ ƒÈ ö®□Tø›á□É>š.I°´c□Ù!·e
Ã• •□□|â·>□é□i>□”Îa□{r÷ž\

"ÑU‡ N(Ð(¢Š (¢Š (¢Š (¢Š (¢Š (¢Š (¢Š Á¾ÿ •Ù~£ùUz±}ÿ -
²ýGòªô QE QE QE QE QE V/‰¼Mká{{››[Ë£qp¶ÑCg'GvÐ€#?tÖÕq_>Ñ¯‡ï
±»¼zÌÅi'MŠ¯' üâ(ÐüCó§Ž/øC¼Z›Ø.÷Ó0«"Ô•Ü
ì‰
¤' '{WÄÝí'[jv¾ŒÓ6ØßS³0£Läãñ®§RÓ-5}>[ØüÛY€&â»€ ã
ç~4‰gã½Qñ,zŒ'ÜÌèì.#LÁ"ûÀ>~côÈ÷®ûJÿ •Vÿ |ÿ !^W}m§Å•

ÛÛC□0Ç§*G□…U-€

•êšWüz·ûçù
v«©A£è÷°□Èsog□ÜH□eŠ¢–
8–¸□ÄCñj+˜#ž□□øÚXdPé"i!•”Œ,□~A□ë¦ñ´□]x□Ä6öñ<³K¦Ü$qÆ¥™ØÆÀ □R}+‰Ðþ'Ùè^□Òí
5□

ø¦Æ;KH¡–î}0ˆAT
Nsœqé@–•¥j-
Ú°]½÷ØîìüäÝö{Èü¹cöeÉÁ«•SLÔìµ•6□GN¹ŽæÒuÝ□±ž□□ž1Ú-Ð□E□P□E□P□E□P□E□P□E□P□E
□P□\Ûÿ ¬o©®’¹·ÿ XßS@□¢Š(¢Š(¢Š(¢Š(¢Š(¢Š(®oCñÆ“â□~óH°[†’ÕK™Ù ŠE□°”9
É□¸Î;□–ã=Jêvµð¾“!MOT□<«ÿ .Öãýd‡Ðÿ □÷>Õ™¡éÖ°GÅ‰4û(ÄvÖú□QÆ£ÐKßß¹4□ìÕÊEñ
E¸ñ×ü"0-Ô—Ãx3¬cÈ

‹¹“vs¸□2 ã8§øãÄW□&•□¦–,]sS“ì°t'þz-®□ÛAó□ôõ®>□

Ûø[â7€t¸ÊÉk~óÎßzyYwor L

Ô□ë□•â□é~□ÒŸRÕîD□á,/□™ØôUQÉ'Òµk^ø□¢jWñhÚÎ•n—
w:%è¼6R8Q:□ó O□‡QŸz ª¿□ôH¦^jzO^4›iX*^j□yŽ□OO›'•Â½□Y]C)□HÈ ðEy□±ñ□Nø—
¢Éá/□Û<š-¥-ÉÅè□-š□73düì1ÀLúö¯TÓl—MÒìì□ÙÒÚ□…Y°°U□'ò
TQE □QE □QE □QE □QE □QE □QE □QE □QE □QE □QE □QE □QE □QE □QE □QE □QE □QE
 □QE □QE □QE □QE □QE □QE □QE □QE □QE □QE □QE □QE □QE □QE □QE □QE □QE □Q
E □QE □QE □QE □QE □QE □QE □QE □QE □QE □ãß□□m6ëÂ-(HŒ□i:†éBõä£¯þ‹?˜¯aªÚŽ□
g«XMa¨[Gsk2í’)□*Ãüóí@□□‡Ä?
ØørMiu›) □□#HæRò□d
\çwlcŽøÅr□ ´»‹/□M}r…[Q¼yãÈÁ(P□0ß†+B□,-□,ø\•&G äC%Ã”†r□□kÐbŠ8!Ha□#Š5
^ƒ
 p □ □ÑE□ QE□ QE□ QE□ QE□ QE□ QE□ QE□ QE□ QE□□□Çü{KþáþUÎ×Eqÿ -Òÿ ¸□•s´
 QE□ QE□ QE□ QE□ QE□ QE□□çþ
¹ƒIñ—‹4+§H®§Ô□þ□r□-9□~_\□ŽO□Ïo©üEð□…ƒ¤°…□Ä—□□3‟

;FC□Ó=1þ"°}{ÂZ□‰¼¿í□6+¦Œa□’®□¦å ãÛ5&‡á•□Ãq<zF•□¨□¾Ë’Íõc’□: å<=w

…ñ3ÄÚEì‹
j/□í™s□(*C□ž¤-ÞÇÒ—âUÔ:¬zg„ídYoõ
ØŒ'!ÉŽ□;™ÛÐp>¼úWW®xkFñ$

¯§Åt¨r…²}pÃ~u

…á□□ÃLí¤i□Û;Œ4€—r=71'-Ù □9¯--
î`¶žê□§¸$C□È□¤#®Ðy8öªòëš\□°é3_A□ûF%X$m¬ÊI ŒõåO□šKí□LÔu;□FîÕe»°,ÖÒ□aå“×€
pz

¹¨µ¿
èž#E]_M,è Â»

:□@Ã□□Æ€8ï‰±Ø$š<š□′ž'7ð‹ #

□ÍósœrS-¼W¶×•è-□ðß‡/□ãJÒa,~,RYÜ□fbHü+Ñ(¢Š(¢Š(¢Š(¢Š(¢Š(¢Š(ªš—
üy7Ô□: ·U5/øòo¨þt□‰E□P□E□P□E□P□E□P□E□P□E□P□E□P□]*ýÑô®j°Uû£é@-□â?□øÃTñŽ•¬hrèr
ZiÐ"□¶¨ÓagbA"□ŽH] □xçŽõÈøãþ□Gö‡…□µ□â□ó?¶bû-
Ù~Ñ•;□·ÌÝü□sŽkÛê†¥¢éú¼¶2ß[‰žÆán-Žö]'€@n□ÏSÁÈ □9ñéÖtÝ-
Â^%×ÒÍ®´□U_P:ps□Bä©eÝóp6Ž{šô
ß□hÚ~„úÕÆ£l4å□Ì□¬€«Œdmõ'°□kFâÞ□»y-îbI •J<r(ee=A□¨®>ßá/•-
oÅì^□¶óƒn□ÝÝ□ÿ p±_Ò€+ü"²¹ƒÁ□þî&†mZòmDÆÝTHß/æ -
Æ»Ê □ è(□Š(□Š(□Š(□Š(□Š(□Š(□Š(

Ý_îÅõ?Ò²ëSWû±}Oô¬° (¢Š (¢Š (¢Š (¢Š (¢Š *ô°½†… ÿ hjwImi□□y_;T³…□ÇNHæ¨Ö¬6
-×ú;ZÞ[Åqo(+$R eaž„-□ AªËáÝKA-
}R]:çIhÉyfthŠã®îŸˆ¯:ðF¡ªéŸ□u{ý?Î□[}-ô‟(,Â□ÎÃƒ×□qÇµtÉð‡À)x.‡†íüÀsƒ$…?ï,Û
□

Wi1Ã
CiH¡U@U °

□áö‶ákÏ□Áu
ÚjRßÛ«êW7□f′iH□Ä…²xlü§¦+?áÛ[ØøóÅ□&ƒpgðÝªÅ$J²y‛ÛÎÙß□7§RGb+jûá'□5□Æ°¸ðí¸•
ŽãåI$JOû¨À~•Òé□.™ Ø–Ž‶c□□²œùp¦ÐO©õ>æ€%¶Ô¬oMÀ´¼·¸6îc˜C(s□ŽªØ<-
cÍTÒõí□Ä°oÚtëûKëV_›k□•èÊyB□.•áÍ'@–
úM.ÍmÞúsqrC3y′–§′q̄ô□□…©|)ð6-x×w~□¶31ÜÆ'xƒR□€?• sÞ□[□þ,xª?

□Æ,-â3-¿ú…»'□˜ã¦ìã½z•QÒtm7A°[□*Ê
;U9□Â›F}O©÷<Õê (¢Š (¢Š (¢Š (¢Š (¢Š (¢Š (¢Š (¢Š (¢Š (¢Š (¢Š +'Vÿ ^Ÿîÿ ZÖ
¬•[ýz□»ýh□>Š(□Š(□Š(□Š(□Š(□Š(

â¤N¾□³Ô–3"iz•½ìŠ□IE$ý
°y5Ý%tcª½ý·öq•□ź\m+•óÇZ¿$qÍ□Å*+Æà«#

†□¨#¸®R?†>

Š÷ík [ù»·a™ÊgýÂvþ□

□-.ñ¾□Íyonê"¿:•¬□`^D™□zmÜßJõ‹□iZ†Šš¼7Ð}…£□□Z@□

r□ÐŽàÖˆE□□(□□6ãŒzW+/Ã/□MznßA·óKn!YÕ3þà;□J ¡ðì□FóÄž%
Ëmª_bÙ˜c|Q□ªÿ C"ùWZu•7û&mU/ –Â□g'x\H /Þåsœ`ô«QÛÃ□²ÛÇ□$
»□4P□/L :
£¤è□^‡¥ÿ fiÖi□',LD—
□w\î$œÐ□'÷HÕôÃ:ÜY]Ø2åœ²¼d{öüë□à×"'ñZiŒN,°ˆ□□9@p|ÍŸìç□¢o…þ
¸°7□ À□œá□Ñ□ì•`¿¥w~□³¶ÓìÍ-□¼vöñ€□("*¨ç □□³E□P□E□P□E□P□E□P□E□P□E□P□E□P□□
÷ü~ËõÊ«Õ‹ïøý—ê?•W □Š(□Š(□Š(□Š(□Š(□ –öÖ
ˆíæ¹†9¥□Ç□È□8□p□'¨üêzÈ×</¢x–8ÓXÓ¢°ò³°¶C.zà,□è(□□âuþ™□,5□;ÇŽK‹¨ü»[~
¼¤ü›W©ÁÁü+¢Ð-î-<=¦[]'nb´Š9I9Ë„ þ¹¬½À>□ÐnÖëNÑ ŠáyY□šFSê
"□Â¶uM2ÏYÓgÓ¯áó-g]²G¸®áœõ□□Ò€9-[þK□‡□ìs^™c<6°l×□□¤PÆYä'F
¨ d'O Wšÿ Â¢ð7ý ÿ òn□þ.»}
Âú5¯„eðô6At©□'µ¹'ÎUùa¸•Üä÷ □"«iË§Ç¨6¡j,¤Û²äÌ¾[n8\6prO-´_jzu†Ÿ%åõå¼6j¤´²
¸ •©ëU?á□ÐÏ†ÓÃÏ¦ÂúLh□mdË(ärNsžsœ×?mðƒÀ6—Bæ?

[™□È□Ë$‰ÿ |³□ý(□ŸÁ«v‹Á×—
□Cay©Ü\ØFÃ□mØ€¸□‡□þ5ètÔE□□□B¢€□T`□è)Ô QE□ QE□ QE□ QE□ QE□ QE□
W6ÿ ë□êk¤®mÿ Ö7ÔÐ□h¢Š (¢Š (¢Š (¢Š (¢Š ó¤Ð<{aâ}cV°"Ãs›éq□—
†s$p¯ÜA´ □œu=Íc[ÿ Âyÿ
Fçoü#□Ú¿ÙI¿>□'äùœcø·çðÅzõRM&Å5§ÕÖ

_¼□Ý¦ÜyŒ□Àc8ëß□

•gÃ~<?□®<G£Ëá¹¡□ëmfš‟NZÝ0
à À,Ùç$ã□Šæu¯øYð³|/ö¯øE?µ|‹¿±ù_hò6ì□üÌüÙÆ1•Æ½¾¨\h°}Þ³e«Ïn-þÉdKyw˚Ø□a¸□□
w□€□°ŠéÚm‟k·–□·r„ŠB%Ù□ÎG+-ó‟‟œ

¸®□â5Ü3xÃÂ°□³pm¼9|Ó5Ö_bO"¨òãvþîHã¾k¶Öü9¤øŽ+Xõk5¹KYÖâ□Y—
l‹ÐðFzô<Tº¾« ¦kö□c«XÁylÇ>\Ë□□¨ô>ãš ä¼□¡xHx.{‹Èìì>ÅnÏau□Ø¤□ÀÊ□ÊàõÇÊ:Öç□¯u
□GÀ°-æ«Ÿ·Mh□)a,ÄŽ□□R0□□Ë±øIàM:ñn-ü9neS¸yÒI*ƒþë±-
¥v•(¢Š(¢Š(¢Š(¢Š(¢Š(¢Š(¢Š(¢Š(¢Š(¢Š(¢Š(¢Š(¢Š(¢Š(¢Š(¢Š(¢Š
(¢Š(¢Š(¢Š(¢Š(¢Š(¢Š(¢Š(¢Š(¢Š(¢Š(¢Š(¢Š(¢Š(¢Š(¢Š(¢Š(¢Š(¢
Š(¢Š(¢Š(¢Š(¢Š(¢Š(¢Š(¢Š(¢Š(¢Š(¢Š(¢Š(¢Š(¢Š(¢Š(¢Š(¢Š(¢Š(
¢Š(¢Š(¢Š(¢Š(¢Š(□®¡Ñ□ôaƒU?²íý_ó«´P□/ì»□WüèþË·õ□Î®Ñ@□¿²íý_ó£û.ßÕÿ :»E
 RþË·õ□Î•ì»□Wüêí□□Kû.ßÕÿ :?²íý_ó«´P□/ì»□WüèþË·õ□Î®Ñ@□¿²íý_ó£û.ßÕÿ :»E R
þË·õ□Î•ì»□Wüêí□□Kû.ßÕÿ :?²íý_ó«´P□/ì»□WüèþË·õ□Î®Ñ@□Æ™
,ü{ÕÊ(□Š(□Š(□Š(□Š(□Š(□Š(□£š□ž3□ç□Ò¤¢€)□eÛú¿çGö]¿«þuvŠ ¥ý—oêÿ •-
Ùvþ¯ùÕÚ(□—ö]¿«þt□eÛú¿çWh
_Ùvþ¯ùÑý—oêÿ •]¢€)□eÛú¿çGö]¿«þuvŠ ¥ý—oêÿ •Ùvþ¯ùÕÚ(□—ö]¿«þut

QE □QE □QE □QE □QE □QE □QE □QE □QE □QE □QE Cqm□ÈQ&~^˜5□ö]¿«þuvŠ ¥ý—
oêÿ •Ùvþ¯ùÕÚ(□—ö]¿«þt□eÛú¿çWh
_Ùvþ¯ùÑý—oêÿ •]¢€)□eÛú¿çGö]¿«þuvŠ ¥ý—oêÿ •Ùvþ¯ùÕÚ(□—ö]¿«þuf□V□„i□£Ö¤¢€
(¢€
(¢€
(¢€
(¢€
(¢€
(¢€
(¢€
(¢€
(¢€
(¢€
(¢€
(¢€
(¢€
(¢€
(¢€
¯=œW,□÷d

pjÅ□□Kû.ßÕÿ :?²íý_ó«´P□/ì»□WüèþË·õ□Î®Ñ@□¿²íý_ó£û.ßÕÿ :»E RþË·õ□Î•ì»□Wüê
í□□Kû.ßÕÿ :?²íý_ó«´P□/ì»□WüèþË·õ□Î®Ñ@□¿²íý_ó£û.ßÕÿ :»E RþË·õ□Î•ì»□Wüêí□
□Kû.ßÕÿ :?²íý_ó«´P□/ì»□Wüê{{híƒ□óóuÉ©¨ □Š(□Š(□Š(□Š(□Š(□Š(□Š(
²éðÍ#HÅ²zàÓ?²íý_ó«´P□/ì»□WüèþË·õ□Î®Ñ@□¿²íý_ó£û.ßÕÿ :»E RþË·õ□Î•ì»□Wüêí□
□Kû.ßÕÿ :?²íý_ó«´P□/ì»□WüèþË·õ□Î®Ñ@□¿²íý_ó£û.ßÕÿ :»E RþË·õ□Î•ì»□Wüêí□□K
û.ßÕÿ :±□ o□DÎ Ï5-
□ QE□ QE□ QE□ QE□ QE□ QE□ QE□ QE□ U3¦@I$¿>õrŠ ¥ý—oêÿ •Ùvþ¯ùÕÚ(□—
ö]¿«þt□eÛú¿çWh
_Ùvþ¯ùÑý—oêÿ •]¢€)□eÛú¿çGö]¿«þuvŠ ¥ý—oêÿ •Ùvþ¯ùÕÚ(□—ö]¿«þt□eÛú¿çWh
_Ùvþ¯ùÑý—oêÿ •]¢€
(¢€
(¢€
(¢€
(¢€
(¢€
(¢€
(¢€
(¢€
(¢€
(¢€
(¢€
(¢€
(¢€
(¢€
(¢€
(¢€
(¢€
(¢€
(¢€
(¢€
(¢€
(¢€
(¢€
(¢€
(¢€
(¢€
(¢€
(¢€
(¢€
(¢€
(¢€
(¢€
(¢€
(¢€
(¢€
(¢€
(¢€
(¢€
(¢€
(¢€
(¢€
(¢€
(¢€
(¢€
(¢€
(¢€
(¢€
(¢€

(¢€
(¢€
(¢€
(¢€
(¢€
(¢€
(¢€
(¢€
(¢€
(¢€
(¢€
(¢€□3□†G□UI□"ý©qþÇåZ·-
ñí/û‡ùW;@□□µ.?Øü¨þÔ¸ÿ cògTP□ßíK□ö?*?µ.?Øüª•□□wûRãý□Ê□íK□ö?*¥E oYLóÛï|g$
qV*ž™ÿ -□ð#W(¢Š(¢Š(¢Š(¢Š(¢Š(¢Š(¢Š(¢Š(¢Š(¢Š(¢Š(¢Š(¨/&h-
Ì‰Œ,:ÔõSRÿ •&ú•ç@□□µ.?Øü¨þÔ¸ÿ còªTP□ßíK□ö?*?µ.?Øüª•□□wûRãý□Ê□íK□ö?*¥E]
þÔ¸ÿ cò£ûRãý•Ê©Q@□□µ.?Øü©WS¸,□ÉÉôª4©÷×ë@□-
□Q@□□Q@□□Q@□□Q@□□Q@□□Q@□□Q@□□Q@□□Q@□□Q@□□Q@□□Q@□ïîd¶□˜ñóg9□KûRãý•Ê§Õþì_
Sý+.€.ÿ j\□±ùQý©qþÇåT¨
¿Ú—ì~T□j\□±ùU*(□ïö¥Çû•Ú—ì~UJŠ »ý©qþÇåGö¥Çû•R¢€.ÿ j\□±ùQý©qþÇåT¨
¿Ú—ì~U=•ôÓÜ¬o·□-,²êÞ›ÿ -
«ô?Ê€6è¢Š (¢Š (¢Š (¢Š (¢Š (¢Š (¢Š (¢Š (¢Š (¢Š (¢Š (¢Š (¢Š (¢Š (¢Š *…õä¶
òª¦Ü□Ï"¯ÖN-þ½?Ýþ´ ÏíK□ö?*?µ.?Øüª•□□wûRãý□Ê□íK□ö?*¥E]þÔ¸ÿ cò©í/¦žåc}»Nz
□jË«Zwü~§Ðÿ * Ü¢Š(¢Š(¢Š(¢Š(¢Š(¢Š(¢Š(¢Š(¢Š(¢Š(¢Š(¢Š(¢Š(¢Š(□
.êþhn^5Û´-2*/íK□ö?*Šûþ?eú□åUè□ïö¥Çû•Ú—ì~UJŠ »ý©qþÇåGö¥Çû-
•R¢€5ìo%¸••öà.x□~²4Ÿøø□+?¨-z (¢Š (¢Š (¢Š (¢Š (¢Š (¢Š (¢Š (¢Š (¢Š (¢Š (¢
Š (¢Š (¢Š +□µ;€ä□œ-
JØ®mÿ Ö7ÔÐ□¿íK□ö?*?µ.?Øüª•□□wûRãý□Ê□íK□ö?*¥E]þÔ¸ÿ cò£ûRãý•Ê©Q@□□µ.?Øü©
ðê3É<hva˜□ÅgÔ¶ßñõ□ûãùÐ□CE□P□E□P□E□P□E□P□E□P□E□P□E□P□E□P□E□P□E□P□E□P□E□P□E□P
□E□P□E□P□E□P□E□P□E□P□E□P□E□P□E□P□E□P□E□P□E□P□E□P□E□P□E□P□E□P□E□P□E□P□E□P□E□
P□E□P□E□P□E□P□E□P□E□P□E□P□E□P□E□P□E□P□E□P□E□P□E□P□E□P□E□P□E□P□E□P□E□P□E□P□E
□P□E□P□E□P□E□P□XÞ*Ôu]+Ã×□Z&-u-D□Hm÷`□Ä

Ÿaœž□:Žµ³E x&–¦aañßÃ–Þ&Ö^ââöÒK—¶□È†□R`#
88Ø

q×¹ê}î¼ƒÄŸòsž□ÿ °[ÿ +Šõú (¢Š (¢Š (¢Š (¢Š (¢Š (¢Š (¢Š (¢Š (¢Š (¢Š Žãþ=¥ÿ
pÿ *çk¢¸ÿ •i□Ü?Ê¹Ú (¢Š (¢Š (¢Š ÚÓ?ãÏþ□jåSÓ?ãÏþ□jå □QE □QE qZ¯„|Aâ□bê[ß
□_éÚ`lZZèì!p¸4’□I$çŽ•ŸàûÝoEñö¥à½WU—W·K□Ô,®ç□ÎT.□£Ÿâ9=}½ð;}gX°Ð4›•OS¸X-
]Î̄íú ;‟Ð

õÉø□J¿¼Õu?□k05µî¬□;[GûÖÖ«÷□¿Úo¼GÓÜP□ß□xoÄ- Ö6¯‰®t•□# E¦€—□¿9-!□h□`

½ëŸÒß[ð_Ä•/Ãw:åæµ¤k0Lð5û□žÞH—qËÿ □#ùûsè÷WPYZKuu2Co
□’I□
ª9$šà¼-□þ0ñ‹øææ'‡M,□´Ñb‘pÎ„üó‘ÛwAíø□□èu^úúÛL°¸¾¼™a¶·•¤–
Fèª□I«□åþ6×ô•oÆVþ□Ôuk□□&Ëeæ¬n®R/´-□;q¸ŒƒÃ6;c¥ fhz¿ˆõ□‰Þ□Õ/ï¯-
ì5È®å·ÒŒ„G□º ò‹/Bí□ÄûŠö:ò}wÅ~□"âÇƒîá×ô—
³·¶¼YfKÈÌq□@□3□□žÙ¯T,xn-ã¸·•%†U□□‘°eu# ,8
Žô□%□Q@□□Q@□□Q@□□Q@□□Q@□□Q@□TÔ¿ãÉ¾£ùÕ°©©□Ç"}Gó

J(¢€
(¢€
(¢€
(¢€
Tûëõ¤¥O¾¿Z éh¢Š +□¸ð7ˆµ«›‹-cÆúµ£4Œmí´i□¼P¦~PN2ç□É8®ú¸Ï□økÆ:-íÄš7Ž

—fè□Z□:7Úq,|Üîäóí@□~□êÚÄ×^"Ð5›ßí□t[Å†+â 4¨À•□Ä1ÏÖ³b:×Ä•□ëg×¯´o

éwMcös^æ°™~û□0p 'Œu«

î[G¿Ôü□¨iÐÚêöx¼–â □E¾W<Í¹òÛ³€r□-□þ

ÿ È□|-þµuk¡/ûÛ¿Ã□□VÎmsÀ^:Òt[ýnïYÐµ-ñ[Í|COm:Œ€_øƒp?-ØçÓ«Îþ&üÚç□#OõÇ_…‡®Ð

ïé^‰@-m¯\k-(ø“/„-µË•□O³°[¹^Ï
qrÌØù\ýÕ-Ýÿ J“[k~ ñ□‡--üM©k:v±tmf³Ôä□JœgÌWÀ8^ÿ ý~ãkoøL<XšO‡-Ú-
□ITiµµ˜Ä,Uù□ü¼ÈXgåè3×-RÓâÔ| ã(/¼j□°u E¥§ˆ□-
ôbÝ#h□□†çæ_Äõ □^¢Š(¢Š(¢Š(¢Š(¢Š(¢Š(¢Š(□7Wû±}Oô¬°ÔÕþì_Sý+.€
(¢€
(¢€
(¢€
(¢€
(¢€
I□P’ÚtÒe,+ö‰„□N
DlpH□qKVôßøý_¡þT□Æê^□ñ□®“s©§Ä=xêÐDÓni□mY”g□□0□□SZú7□¥“á□^/Ô`-
zX¼òF¿(‘×#•@Ä□¦k
Äž□ñî§e4mãXµKbIm:k!h“/üói"mØ#□ç[Z□ÆŸñ
á□ÆŸ□‘Òãx¤Ó¦µP□ÙdQ·□¦@àŠ ÈÑü-
âO□è¶ÚæµãmnÊúö%¸ŽÛL”C□°°Ê©\|Ø□g'óë[?□5ÝVíµŸ□ëó-
Æ-¡Üˆd¹UÛçÄãtnGb@?¥dh÷Ÿ□ü7¥[h□á;□]m#X!Ô"ÔÒ□dQ…,Œ7g g¥3áÊë□|Iñ½Þ´-
‰xVÉ%[2Æ%o,• ·$…Ú□¾h□ÔkÈ¼9á‹Ï□Þx’úóÆ>*´6úåÝ¤PØêF8’4a´□
ã®8ô¯]¯>“áL"ûP»±ño‰ôó{u%ÛCgz#ˆHç$í
Ïâz□@□/†¼5ÿ □Ý¼ðÿ më:¯œá·ê—
^{&□0§□□·+Ï¼□ªk°g□u/□ë°‡ö¡,Ño¬¯™□HÑ□ÚUÀêA=}□áè4 QE□ QE□ QE□ QE□ QE□ QE□
 QE□ QE□ QE□ QE□ QE□ VN-þ½?Ýþµ-Y:·úôÿ wúÐ□}□Q@□□Q@□ZÓ¿ãõ>‡ùUZµ§□Çê}□ò □
Ê(¢€
(¢€
(¢€
(¢€9Ï□ÿ ÂOw{□›¡
{□i#Ýqª͂‰□.HÙ□]ß¾[å□îk□á%åõç„îÿ ´/îo¦‡S¹„Ms!w*-€2□
Jï+Ï¾□□Ê-©□Øbïÿ C □ø›PÖ¼AãÈ¼□¢êo¥[ÃgöÝBö□□R¥¶¬hOÝ=óïíƒ•®Úk□

Ÿˆ ñ>-«è¢æ8u+]VQ3,nvùˆØ
'Ç¸-•øÄz¡ñ…a¶½í>Ç{§ÜIåùÈpdsÀn};ä×%ñ7UñÎ«ðûR‹RðÍ†`Æ%I~.f"™SjÆ

ìuì□ {m□□°4VÑFÇ,¨□Ÿp*J (¢Š (¢Š (¢Š (¢Š (¢Š (¢Š (¢Š Á¾ÿ •Ù~£ùUz±}ÿ -
²ýGòªô QE□ QE□□□Iÿ •‡ÿ súŠ×¬•'þ>ýÏê+^€
(¢€
(¢€
Ë×îu‹m8□aXCy}$,5□ËåÇ□9Ì□Ü□Žƒ"šÔ¢€<ÓÂ/®Ú|UÖtÍc]¸Ô¶é□ÎT□□£³såÆ8P□□ê{šÚø‰â
-KEÓtë□□Æ°¾¯z-6ÒÈ2°îë!□ð?Ÿ~•Ÿ¥ÿ Éw×¿ì□oÿ ¡ÖŸÄ□
_x"L±ŸH¸Š□_K¼KÛ6˜□ŒËü-ì□¥ `j>□ñ6›¦K©i><×®u˜□Ê"°"=´Ì□vùXÂƒÓ©Åv-□ñ
ø¯Â□f¶□#]C™□z+,UÀöÜ□sCÄ-
□'‹Èÿ „3L±"□šöçTW„z¶Ä□±íšwÁHd‡áV"d□ó-wQŒ|¦WÇøþ4□Ý]K,□sË□□q*FÌ•«□20□
àdñ"^W¬Ïã
o□øBóWÕ~È·ú˜„éV.D1Ç´œHýen™þ□ØW-WŸ|Eÿ '§À_ö□ÿ Ù□ z□□Q@□□Q@□□Q@□□Q@□□Q@□□
Q@□□Q@□soþ±¾¦°Jæßýc}M 6Š(□Š(□Š(□¥¶ÿ •¨¿ßÎ¢©m¿ãê/÷Çó

†Š (□Š (

KÄ-□ñ□ˆ5•«âk•'EHÀ□i %Ä¯ÎKHAÚ□□□¯zçô·Öü□ñ#KðÝÎ¹y-i□Ì□<□~Áç·’%Ürÿ Ä□þ~Üú=
ÕÔ□V’Ý]L□ÛÂ…ä’FÂªŽI&¸/
A?Œ<bþ9¹‰áÓ`•-4X¤\3¡?<ävÝÐ{~□€høÛ^Õmït¿□øu¢•YÕYñq*î[XPeå#¹ä □□Õ(¾□ê•É□Ò
üAñ3^©

ÆIÕ cÿ \±Œ{f±|S§êz·Ç++-?Y"I'Ãä›ˆâWr¾{nTÝÂ"òüÝ€£Å>□×ü□¡\x—Fñž¹{-Ž%šÓUœO

É□□□□·ƒÛôë@-³HÌ¨¥˜…P2I8 U}:ñu□2Òù□ª\Â“*ž 2ƒ□Ö¸/‰~"µk»-
□6«m¦□i¯™□w<ë□†Ð□0□ˆ□Ÿ□G¶h□›Ô|G®k~.ðÿ ˆ,õ+»O□Ükqi¶vÑHÈ—q□ÞdÎ□Þ

Ã
žÂ½ª¼‡Æ¾!ðšKà{]+[Ò□ÒÃY€²Ay□,□*°ÉÃp£ŽMzµ•õž¥i□Ý…Ô□VÒgdÐH□□□□

8<,?
±E□P□E□P□E□P□E□P□E□P□E□P□E□P□E□P□E□P□E□P□E□P□E□P□E□P□E□P□E□P□E□P□E□P□E
□P□E□P□E□P□E□P□E□P□E□P□E□P□E□P□E□P□E□P□E□P□E□P□E□P□E□P□E□P□E□P□E□P□
E□P□E□P□E□P□E□P□E□P□E□P□E□P□E□P□E□P□E□P□E□P□Xþ(Ô5]/Ã×7š.™ý¥□ß.×vÝù`□ä2□
Ø¢€>vÔ.>$j□tÏ□·□eK‹
cn¶á²®□™É9Ïü´?•{_„5MkXÐ–
ë_Ñÿ ²oLŒ¿gß»*:7¶yãÚ·¨ □Š(□Š(□Š(□Š(□Š(□Š(□Š(□Š(□Š(□Š(□î?ãÚ_÷□
ò®v°+€M¼

’‡ùV□‛/üò□ûäĐ□tTžD¿óÉÿ ï‟G‛/üò□ûäĐ□tTžD¿óÉÿ ï‟G‛/üò□ûäĐ□tTžD¿óÉÿ ï‟G‛/ü
ò□ûäĐ□¶™ÿ –□ð#W*¦œ¬–¸e
î<□Vè ¢Š(¢Š(□ÈµÛo□ßxòKû¿□□lé6□Ÿì»qªÁ□@□ùlêÙ,ç¶q·Ó<×]¡ø‡ÆWú¼6Ú·□?²ìŸw™w
ý¯

þ^□‘ò(ÉÉÀüs]}□□åž=³ñ-
¯â¨mãð©Ö</j□Alš”VÂê\□™wd•Sü8 ‘’OJÛÓ|Kã™oím®¾□‹+6‘RIÆµ□ˆS
□Ø□N□8–•ÜQ@□Z½×ˆáñ□•

•§ÚÏ¤ÊÎ5□å|<* Û´n□Ï=□áÖ¥¾ðŸ†õ; ·»¿ðþ•ws&7Í=”r;``e^Éà□øVÅ□□ç□¯Ã}2_-
øzîÏÃ□HÑ †ä_*ÛB¨X¨ò÷&>nsŽ

=«Ðà,[xííâHa‰BGjQ@À

□µIE □QE □QE □QE □QE □QE □QE □SRÿ •&ú•çVê® ¬Öl□□r8□€0è©<‰□ç“ÿ ß&•"_ùäÿ ÷É □è©<‰□ç“ÿ ß&•"_ùäÿ ÷É □è©<‰□ç“ÿ ß&•"_ùäÿ ÷É □è©<‰□ç“ÿ ß&•"_ùäÿ ÷É □ éSï¯ÖŸäKÿ <Ÿþù4«

»ÇîŸ¯÷M t4QE !É□□□Ö¼ò□WâŽ□¦ÒïÃZn¾W„¾µ¿K]ÃÕÑÇ_\`W¢Q@□Oƒ¼5¬Ã¯ê~*ñ3Û□Zþ4,;
kRLvÐ¯!w-¤œ□ô÷¬¦Ñ|[àŸ□ê×¾□Ó-µ--
VœÝÉ`÷BÞH'?|«7ËƒþŒdú]□□çú>•â=□Æ6ž'ñe½µ„ztnšv™□¾q□Üa¤'Ç□ã□□n˜ç£{¯□□□Åjš
}©ðá´.÷…ÿ z'Éù@ÝÓ□þÇµnÑ@-q{¢x·Â¾2Õµß

ØÚk□:¹□îl&¸□K□^1"sÆ□=}zqP_é~4ñýÎŸi®èÖž-
Ñm®'êxÅâÜÏ9C□€¨Ú□¯ù□éÔP□E□P□E□P□E□P□E□P□E□P□E□P□E□P□n¯÷búŸéYu-ª#°ÅµY°OA
ŸJÍò%ÿ žOÿ |š ŽŠ"È—þy?ýòhò%ÿ žOÿ |š ŽŠ"È—þy?ýòhò%ÿ žOÿ |š ŽŠ"È—
þy?ýòhò%ÿ žOÿ |š ŽŠ"È—þy?ýòhò%ÿ žOÿ |š ŽŠ"È—
þy?ýòhò%ÿ žOÿ |š Ž«ßÃ¬Oa*h□6öÚž□C%Ân□,
V-„□29□Í\ò%ÿ žOÿ |š³§Å"Þ)d`0y#Ú€9C¯|Q'□h¾
Ób°#oÛ›SS ?Þòþþ=³[>□ð½ß„<□-…½ÄWz¼žmÌ"K•Ž[—ç'□…Î□®□utP□ž¶½ñ:å□¬^
Ólç?/Û¦Õ□HTÿ {ËQ¿-ÕÐx;Ãð‹é2Å=Ó^ê7s5ÕõÛ

□¦n¤

Àp ö®ŠŠ ÀÐ.¼Ms&®5Í>ÒÑc°tÓÌO»Í‡øYðÇ□§§ÐW:¾"ø™n¾Lþ□°»—
§ÚmõdŽ#ïµÁlW Ñ@□g„<1«[kz□‰üK5»ëWñ¬+□®|«XW□ŠO$“É>¢»:(□Š(□Š(□Š(□Š(□Š
(□Š(□Š(□Š(□Š(□Š(□Š(□²uoõéþïõ-jËÕ#w•
£0ÛØg½ fÑRy□ÿ Ï'ÿ ¾M-D¿óÉÿ ï“@□ÑRy□ÿ Ï'ÿ ¾M-D¿óÉÿ ï“@□Õ-;þ?Sè□CäKÿ <Ÿþ
ù5gOŠE¼BÑ°□<‘í@□4QE □QE □QE □QE s^%Ö¼O¦]Ã–…áí¸]7I/ö”VÛ□?w

<sšâü□|yá¨N™uàLÛÝj/q-×ö¼¹I□□ò

–Ú=□5ë4P□1âMOÅúmüM¡xv×Y±h¾t7¢ÞU"'<·ÊW□÷Îk

□□Å^1Öl/<Ymi¥é:|Âæ□* ¼çšeû-+•—
jõ uï^‡E □QE □QE □QE □QE □QE □QE □QE □QE `ß□Çì¿Qüª½[½ŠF¼□¬nFz□íPy□ÿ Ï'ÿ
¾M GEIäKÿ <Ÿþù4y□ÿ Ï'ÿ ¾M GEIäKÿ <Ÿþù4y□ÿ Ï'ÿ ¾M [Ò□áÿ Üþ¢µë+L□Òw,Œ£o
qŽõ«@□□Q@□□Q@□ex†ÿ VÓ´Ï?EÑ□¶.÷…û7Ú’ßåç-
¹¸ãŽ=ëVŠ ñûY¾!Ûxîÿ Äßð¯7}®Î;_³□m[□›NwnïŸLW jwþ'_

Z^éz-«ê-±®tû‹ 6□§r,ƒå,

9èFk ¢€<Úø|Dñ□«é7□=§…ì'-]ÕÉ½[©Ú3Ã,a□□#ŒŸZì¤³›Bð›ÙxvÒ)'³´1ØÛÈØVe\
c'Ô□"'õ-z(□'L¹Ö[Ã□Üêz|+¬ýœ¼¶pÈ□□p~@Ä□2p3"Œõ5æ¾"›â-¿ªè7¿ð¯<□ì›Ïµlþ Ú·o7Œm
Ï□~¼×°Q@□Þ□Ôµ-NÂYµÍ□û□áe*•}±.w¦□Ý¹□□$Œ{VÍ□P□E□P□E□P□E□P□E□P□E□P□E□P□\Ûÿ
¬o©®'¹ç†_1¿týOðš ŠŠ"È—þy?ýòhò%ÿ žOÿ |š ŽŠ"È—þy?ýòhò%ÿ žOÿ |š ŽŠ"È—
þy?ýòhò%ÿ žOÿ |š Ž¥¶ÿ •¨¿ßÎ"È—þy?ýòjKxe□1□□€□s´úÐ□õ□Q@□□Q@-YãÛ?□jþ*†Þ?
•cÂö¡d□É©El.¥À9—vIU?Ã€ □$ô-½7Ä¾9-
þÖÚëáÈ²³i□$œkP8…2□m€dàs□é]Å□□ÄøßÃzÍÖ¯¤øŸÃ-nu□/z□{†Ú—
0¸ù□žÇÓëì+□V²ñç□ìÆ‰ªh¶¾□Ò%e7Ò‹å¹šd□□'í□\'ßÿ ¬}>Š çüC&¿¦Xé°ø[L´°ÅÌqO□ï´E
o,
/Ì9□-¿CW5/□h:ÅÂÜjš&›}:®Å'êÕ%`¹' °'□'□zÔ¢€<ãÅß□ôË»ß

6□á□%bƒTŽ[ÿ *Ú□Á· î

07

ãåçé]ý•□ž™g□¥…¤□¶Ñçd0F□□''
8□’□□±E □QE □QE □QE □QE □QE □QE □QE □QE □QE □QE □QE □QE □QE □QE □QE □QE
□QE □QE □QE □QE □QE □QE □QE □QE □QE □QE □QE □QE □QE □QE □QE □QE □QE □Q
E □QE □QE □QE □QE □QE □QE □QE □QE □QE □QE □QE □QE □QE □QE □QE □QE □QE □
QE □QE □QE □QE □QE □QE □PH ’p□GçÃÿ =Sþú□□%□Ÿ□üõOûèQçÃÿ =Sþú□□%□-
Ÿ□üõOûèQçÃÿ =Sþú□□%□□‘‘E □QE □QE □QE □QE □QE □QE □QE □QE □QE □QE □QE □Q
E □QE □QHÌ¨2Ä□êM -□Ÿ□üõOûèQçÃÿ =Sþú□□%□Ÿ□üõOûèQçÃÿ =Sþú□□%□-
Ÿ□üõOûèQçÃÿ =Sþú□□%□-
Ÿ□üõOûèS•ÕÆUƒ□cš u□Q@□□Q@□□Q@□□Q@□□Q@□□Q@□□Q@□□Q@□□Q@□□Q@□□Q@□□Q@□□
ÖtLoe\úœS|ø□çª□ßB€$¢£óáÿ ž©ÿ }
<ø□çª□ßB€$¢£óáÿ ž©ÿ }
<ø□çª□ßB€$¢‘YXeH#Ô□Z (¢Š (¢Š (¢Š (¢Š (¢Š (¢Š (¢Š (¢Š (¢Š (¢Š (¢Š (¢Š (¢
Š (¢Š (¢Š (¢Š (¢Š (¢Š (¢šÒ"□3ªŸsŠ u□Ÿ□üõOûèQçÃÿ =Sþú□□%□-
Ÿ□üõOûèQçÃÿ =Sþú□□%□Ÿ□üõOûèSÁ

□□□{Š Z(¢€
(¢€
(¢€
(¢€
(¢€
(¢€
(¢€
(¢€
(¢€
(¢€
(¢€
(¢€
(¢€
(¢€
)†h”á¤@GbÂ“Ï‡þz§ýô(□J*?>ùêŸ÷Ð£Ï‡þz§ýô(□J*?>-
ùêŸ÷Ð©□□d□Š (¢Š (¢Š (¢Š (¢Š (¢Š (¢Š (¢Š (¢Š (¢Š (¢Š (¢Š (¢Š (¢Š (¢Š (¢£
óâ-
òÕ?ï¡@□QQùðÿ ÏTÿ ¾…-|?óÕ?ï¡@□QQùðÿ ÏTÿ ¾…-|?óÕ?ï¡@□QQùðÿ ÏTÿ ¾…-|?óÕ?ï¡
@□QQùðÿ ÏTÿ ¾…IÖ€
(¢€
(¢€
(¢€
(¢€
(¢€
(¢€
(¢€
(¢€
(¢€
(¢€
(¢€
(¢€
(¢€
(¢€
(¢€
(¢€
(¢€
(¢€
(¢€
(¢€
(¢€
(¢€
(¢€
(¢€
(¢€
(¢€
(¢€
(¢€
(¢€
(¢€
(¢€
(¢€
(¢€
(¢€
(¢€
(¢€
(¢€
(¢€
(¢€

(¢€
(¢€
(¢€
(¢€
(¢€
(¢€
(¢€
(¢€
(¢€
(¢€
(¢€
(¢€
(ªz®–§èš| ·ú□ÜV¶‵

¼²¶ ö÷>Ã"@□(¯>³ø×àKÝ@Z.®Ñ-`«,Ð:FIÿ hŽ□¹Åz !€ ,□ Š Z(¢€
(¢€
(¢€
(¢€
(¢€
(¢€
(¢€
(¢€
(¢€
(¢€#¸ÿ •i□Ü?Ê¹Úè®?ãÚ_÷□ò®v€
(¢€
(¢€:8¿Ô§û¢ŸL‹ýJ□º)ô QE□ QE□ QE□ QTµ]_NÐôù/õKÈm-cûÒÊØAê}‡&—
IÕlµÍ.ßSÓ§óìî□|Rm+¸tè@#ñ□□rŠæ<Iñ□Â¾□¿ŽÇ\ÕE-Ì'   -?"Y2„•□È¤

Aüª-□â_„|Qª.™£jÿ i¼d.#û4©ÀêrÊ□ë@□e□V□‰¼máï□
c¯j"Ïí[¼ŸÜÉ!m¸Ý÷□ã□‡_Z ß¢¸½+âÏ,5½RßMÓõ¿:òåöE-
Ùf]Íé’€□Ä×i@□□Q@□□Q@□□Q@□□Q@□□Q@□□Q@□TÔ¿ãÉ¾£ùÕ°©©□Ç‟}Gó

J(¢€
(¢€
(¢€
×Ò□áÝÿ ßþ•‘ZúOü{¿ûÿ Ò€/ÑE□ QE□ QEr°ßÄ¯□xvù¬µ=zÞ+•8x‘ZVCèÛ□Ú~´□ÕQTô-[OÖô
øïôËÈnídû²ÄÙ□Ô{jÇñ□•¼-áiÅ¾³-[ÛNF|□□G□±*€‘øŠ é(¬-□Ä°7‰ì□æ‹¨Cy
•¬c'*}□N□üEjÐ□ECwwmai-
ÕåÄVöñ.é%•Âª•ROJ¥ ø‡Jñ6□ý¡£Ý‹«_1£ó□2□ËÔa€4□§E□P□E□P□E□P□E□P□E□P□E□P□E□P
□n¯÷búŸéYu©«ýØ¾§úV] □QE □QE nißñäŸSüêÕUÓ¿ãÉ>§ùÕª (¢Š (¢Š (¢Š (¬kï□h:n¹g
¢Ýjp¦§xÁa¶□g$ôÈ í□Ôâ´o®ÓO°¸¼•%xà□¤e‰
¹ g□G$ûP□Š+…ÿ …-¢ÿ Ð+Ä?ø*—
ü+SÃ~:Ò<S¨]ØXÇ{□Íª,’ÇulÑ□□§Z é¨¢¹Mkâ_ƒ|=|ÖZž½o□Êœ<H¯)CèÛ□Ú~´□ÕÑT´•cN×tø
ïô»Ènídû²ÄÙ□î□¡ö5v€
(¢€
(¢€
(¢€
(¢€
(¢€
(¢€
(¢€
(¢€
(¢€
(¢€
(¢€
ÉÕ¿×§û¿Öµ«'Vÿ ^Ÿîÿ Z Ï¢Š(¢Š(®,Óþ="ÿ tW?]□§üzEþè ¨¢Š (¢Š (¢Š (¢±—Åš

ž%-□□S†M[c9¶□,T

»ˆ□SìH4□³EPÖuh4=*mBâ+™b‹nRÚ□•ÎH□(äõ®Sþ□¶‹ÿ @¯□ÿ àª_ð

êŠÁð¿‹ô¿□Cw&š.Wì’ù3%Ä□&WÆqƒ[Ô Q\uÿ Å_□é·æÊçÄV¢pÛXF¯"©ô, ¨üë«´»¶¿´ŠîÒxç·
•CG,L□\-àŽ´□5□Q@□□Q@□□Q@□□Q@□□Q@□□Q@□□Q@□7ßñû/Ô□*¯V/¿ãö_¨þU^€
(¢€
è ÿ •x¿ÜÊ¹Úè ÿ •x¿ÜÊ€$¢Š(¢Š(¢Š(¢Š(¢±´Ï□h:Î±w¤é°œ7W¶j□t‹,□g-
{□IÏ□□"¶h ¢±<Câý□Â¦ÔkZŠZ5Û”…J3—#□áA r9<s[t QT5•Z□□J›P¸ŠæX¢Û”¶…¥s’□
9=k”ÿ …-¢ÿ Ð+Ä?ø*—
ü(□°¢°|/âý/ÅÐÝÉ¦‹•û$¾LÉq□‰•ñœ`Öõ □QE □QE □QE □QE □QE □QE □Í¿úÆúšé+›□ð•õ
4 Ú(¢€
(¢€
(¢€
é#ÿ V¿A\Ýt‘ÿ «_ □QE□ QE□ QXž ñ‡‡ü+□6·ªÛÙ—□DbYØz„PX•|RxwÆ--
ñdr>‡ªCyåýô\«¯¹V •ïŠ Ü¢ŠÄ-/ÐOŠ¿á□□Š□gfól□‰□nî[□AÇ8Îh□nŠ(□ŠÆÓ<Y ë:ÅÞ“¦ê
pÝ^Ù¨iÒ,°Aœ}ìm'<`□ŠÙ □Š(□Š(□Š(□Š(□Š(□Š(□Š(□Š(□Š(□Š(□Š(□Š(□Š
(□Š(□Š(□Š(□Š(□Š(□Š(□Š(□Š(□Š(□Š(□Š(□Š(□Š(□Š(□Š(□Š(□Š(□
Š(□Š(□Š(□Š(□Š(□Š(□Š(□Š(□Š(□Š(□Š(□Š(□Š(□Š(□Š(□±üGá}'Å-
0Yk6ÆâÚ□ÖácÞT□ •œ-F□ñ[□OUÕltM2}GR¹KkH□t’¹à□êO@□$Ð□›übÐ|3¦ü.¾uÒ¬-¦ŒÆ-m

□□.8R□¦ì•@k°ø|—Qü<ðúÞ□ç‹□³»¨□FÑùb¼ëN³Ô>3xš□oT·’×Á°|„ÙZH9¼p~ó

ãŽOA÷FNã^Ò □ `
(¢Š (¢Š (¢Š (¢Š (¢Š (¢Š (¢Š (¢Š (¢Š (¢Š Žãþ=¥ÿ pÿ *çk¢¸ÿ •i□Ù?Ê¹Ú (¢Š
(¢Š èâÿ RŸîŠ}2/õ)þè§Ð□E□P□E□P□E□P□)â□ i—Sê:õè–òê;G□ÑÜ>è-p„□□t

HÉc"èEWøMÿ $³Ãÿ õì□ô&®—[ÿ •

£ÿ ^²ÿ è&¹¯„ßòK<?ÿ ^Çÿ Bj ç|Aâ-□Â?□Z÷]½û%¼¾□H‘ü§| ·Ú□ã
è□vþ-ñ§‡<U¼hš½½Û ÜÑŒ«□ëµ€l{â·J)mÅAlc8æ¼ÇÇÖ6VŸ□<□w¦E□:äÚ˜I

*□äµÁóKc¨□¹õ4□éõæ_□õÝ7Ã□□¼□ªê×?f²†=@<»□ðLh□
êGjôÚBŠÄ□ ‘Đ‘Ò€9-□â□„üQsöm[·¸¸#"□□□·Ñ\□□
é«Ì>3ØÙG Xê□ĐÅˆSP•tÙcP%i
•”□É□ÉÇ°¯O □Š(□Š(□Š(□Š(□Š(□Š(□ªj_ñäßQüêÝTÔ¿ãÉ¾£ùĐ□%□Q@□□Q@□□Q@□ké
?ñîÿ ïÿ JÈ–}'þ=ßýÿ é@□è¢Š (¢Š +3Lðö•£E<z~•o •Ùæ*™i□ŽIbyn½ëN¸Ÿ□øŽûPÔŸÂ~□
`Ú³¨ûeî3-›□þ&=ä#î¯âx

¿†vð[ø»Ç‹¦ □F]F4,8Æ#Y,;hè9+Àö®’æ_□xWU¹¼½¹Ò4íFû÷ÒÉs:$²Ž™□ŽHã□□V□‡<?cá}
ÛIÓÐˆa□»□´ŒygcÜ“Í2óDðß‰[Ï½Ót½Q¢-□›,□ÌP,w&H8ÁÎGc@□_□í¡Ô~$øƒÄÚ-
³A \ÛG□Kå˜Òî`rÒ"•2 ÈÝŽI=y¯L¯-Óì-ü!ñ’ËEðó´Zf¥a-
Åîœ®Z8□~ìŠ□ÝÉ □^¥@□z·„ôÝwWµ¿Õ□×QÚ¯î¬¤|Û‡É>aNŒÜãœ□Ž•Ìü- xWR `□bïÿ C¯B¯>
ø=ÿ "¶¥ÿ a‹¿ý

€=□Š (□Š (□Š (□Š (□Š (□Š (□Š (

Ý_îÅõ?Ò²ëSWû±}Oô¬° (¢Š (¢Š ÜÓ¿ãÉ>§ùÕª«§□Ç′}Oó«T QE□ QE□ T7P□›9àY¤•¥••K□
□Ó#□”žã¨©¨ □&ñ/†t¿

ëþ□‡N€‡—ZÝ=Ä¬^iÛaù•Ï,□ÈÅzÍy÷Ä_ù□|□ÿ a•ý•× Ð□mFþßKÓnµ
·òí-¢i¥oEQ“ü«□øacq&□wâ}E6ê>!œÞ¸=R-•§Ð'#ýê-ñ&W×/´_□Û9□«L&¾*yKHÎæún
□ô"½□8Ò□’8Ô"
ª£ □Ð
 urM}à?□›«W¼Ñ´Ùœ™n!y“Îrß6X□½³ž:ûWZN□M`ÝxkÂ¾$-
Ú□:>“¨›„□»h#‘□q□‰1’1ï@□ÇÂÝ5á¹ñ6«me-Ž□©ß‰´ûy#1’€`È□ýÐÄŒ

ö¯E¯2ðD#@ø›â
iW□I¡Ak□Ê[´…ÖÎf<Æ¤ô□□q^›@□□Q@□□Q@□□Q@□□Q@□□Q@□□Q@□□Q@□□Q@□□Q@□□Q@□□Q@□d
êßëÓýßëZÕ"«□□O÷□+ gÑE□ QE□ WAiÿ -'□P+Ÿ®,Óþ="ÿ tP□ÔQE □QE □QE RÕôá«isØ□»
«U˜□i-dÙ"Œ,v·lŽ3èkÎÎ‡¦ø□â□…,4«8ím—Mº;PrÇŒ-'->çšõ□óíkþK□…ÿ ì□u@-ƒX¾-
ñ□>□ð®¡¬Ì□}š"cCür-□□□ VÕy×‰□ü%Ÿ□´o
§Ía¤□ªêC±qÄ1ŸÄî#¸>Ô□»ðûÃóx{Â□ÐÞ□Ú•Ó5åû·Þiä;››ã…ÿ €×K4I<□C*îŽE*Ã8È#□ŸE e
Ùx{DÒôs¦Zé–•éû6¼-PØÃ¾ìýïrs\oÁxÂxCP6û¿³□Wº:p$•-
÷ ¸öÈoÖ¤×µ;¿□ê□-□ðôÍ-›□yzÆª□□w,#ÝÈàžŠ□-w:vŸk¤éÖú}Œ+□-¼b8£^Š¢€,ÑE□ QE□ Q
E□ QE□ QE□ QE□ QE□□ƒ}ÿ ²ýGòªõbûþ?eú•åUè ¢Š(®Š□ø÷‹ýÁü«□®Š□ø÷‹ýÁü¨□J(¢€
(¢€
(¢€
Ë×ô+□□iÂÂîâê+S i'ÚS□™Fs□□ÎÓž@ÆqÖµ(□5ðå…ž—ñ«Y²°¶ŠÚÚ-□ÝcŠ%
ª7ú
ô;ÛË}:Æ{Û¹V□h#i%'°*□'k…Òÿ ä»ëßö□·ÿ Ðé~(Øx•V¶Ò¬4}□êÚc\yÚ•°ÞGneTÁHË9û¬rN□
û½³@□7‹ìî5Ï□êž9Õ"hç¿žÖ-6□ëmf'R¿F□¼□
÷šðÿ ‰~ ñ•ß•§¶Ô¼□4»?>

Ü^,‘"í]Š3ÉÀöÍzÇ‡5gSÓžmsBþÆ°•[□u¥ÆäÀ!· Àä‘oz Ø¬_ø,
xWPÖf¾Í1¡þ9¿‹+j¼ëÄÇþÏ‰Z7…Óæ°Ò@Õu!Ø¸âÏâwÜj Ýø}áù¼=á
ho
mJéšòýÛï4òÍŸqÂÿ Àk¨¢Š (¢Š (¢Š (¢Š (¢Š (¢Š (¢Š +›□ð•õ5ÒW6ÿ ë
êh´QE QE QE ÒGþ–~,¹°é#ÿ V¿A@

¢Š(¢Š(□7û□IþÛ}hØ@Ú"¢Æn]w8Qœ OÝëÛ□®□T´¶□ãß‡ŸL‰#°m>æMLÄ1¾,b2øÿ o¹ô-,°¯□ø
®□□ÛÃ□P=ö¯xÆ;□>#óÎÿ û*Ž¥• Uo□x^}□]êÚÄéuâ□M„—³¨ùP□»□~^£□~¾˜ µã□□

h/u□_h¿□ÖÞÆÔužwá□éÜû□^}§øløkâW,!¸—
í□•Ì:…ÍýÉë4ìŠXý□Aì+CÄ1xÍ~&□lAàÿ í-:Â□□™ÿ □8mÕ□Ôy’□l’ÝW À-ù¬□kÄ^3—
âo…î§ð'“□

□bÞËû^□û@(7□àavŽpzÐ□·Ö^¿¡[ø‹N□□w□QZ™□L-
Ò˜ÌÊ3˜ØŽvœò□3ŽµrÆ[‰ôûi®í¾Ës$JÒÛù•ü§#%w

□□##®*Å y¯‡,,ô¿□ZÍ•…´VÖÑh¶ë□Q(UQ¿ÐW¥WŸi□ð]õïû□Ûÿ èuè4 QE□ QE□ QE□ QE□ Q
E□ QE□ QE□ QE□ QE□ QE□ QE□ QE□ QE□ QE□ QE□ QE□ QE□ QE□ QE□ QE□ QE□ QE□
QE□ QE□ QE□ QE□ QE□ QE□ QE□ QE□ QE□ QE□ QE□ QE□ QE□ QE□ QE□ QE□ QE□ QE□
QE□ QE□ QE□ QE□ QE□ W)ãß□[x÷GƒN°¾¸´Hg□□„□¸àŽAú×WE xô□□’□-
8¼g-¤h□ª«` ;□šôo øwþ□m□-
/ûFëPòÙ›Ï°l±ÉÎ>•·E □QE □QE □QE □QE □QE □QE □QE □QE □QE □QE Gqÿ -Òÿ ¸□•s
µÒHždl™ÆàFk?û!□ç±ÿ ¾h□.ŠÔþÈ_ùì□ïš?²□þ{-
ûæ€2è-Oì…ÿ žÇþù£û!□ç±ÿ ¾h□ü_êSýÑO¤UÚŠ¾ƒ□´ QE□ QE□ QE□□Èx‡À)â-
F[¹<Qâk□•□5-†¡åA€0~M§¯□Ž¯á¯†-Ð□¼´-ÏÄÞ&šÞ×";□‹ðÖÄ□F

a Ç9ú×oE qšÇÃm;UÖ.uX5□{J°¹ ÌÚmûD□€□□0G@:U¿

x□EðÕôšŒ&î÷S‘v=þ¡9šb¾€ž ú□]E□□…«øRÃZñ□•-\Ít—
:K;À‘H□□° î□ç§b?□£â/ éÞ"ÕWT:Ž¯¦ß□„&}6ñ¡fPI õ□Ïjêè

?Døm¢èú´z´÷□–¯©D1□Ö«tgx¿Ýè□÷Æk°¢Š (¢Š (¢Š (¢Š (¢Š (¢Š (¢Š *¦¥ÿ -MõÎ-ÔW□
^Le¶ç¾(□ž¢µ?²□þ{ûæ□ì…ÿ žÇþù

°+Sû!□ç±ÿ ¾hþÈ_ùì□ïš Ë¢µ?²□þ{ûæ□ì…ÿ žÇþù

°×Ò□ãÝÿ ßþ”Ïì…ÿ žÇþù«v¶ÂÖ2¡·dç¥ OE□P□E□P□^omðrÆÅî□ÇÅÞ/³□□´Ò-¾¤±†sÕŽ□“îy
¯H¢€9•□ø3þ□Ëù.ÿ á%ñ-©¾#-
“©ßùñ®H;,í□n1ŸBk;QøY¢]ê×:□…þ³¢ÝÝ9’áô«Ö„JÄä’0G>Ø®âŠ ç<1à□□Â□q=ŠO5íÎ<ûÛ¹L
³Ê□bÇ·°ÅLþ□°“Æ±x¨Íuöøí□ ŒH<-™';qœóë□jÝ¢€9¯□ø;þ□K¸n?á#ñ

—å&Ï/K¾ò□ùÎXm9>õ‡£|&³Ðna-ÇÅ~+Xã¸□□oý¢□R¶A;Ô
Ü□□ú× Ñ@□□Q@□□Q@□□Q@□□Q@□□Q@□□Q@□□Q@□°¿Ý‹ê□¥eÖõÝ °

•ÛžÙª¿Ù
ÿ =•ýó@□tV§öBÿ Ïcÿ |Ñý•¿óØÿ ß4□—Ej□d/üö?÷ÍÙ
ÿ =□ýó@□4ïøòO©þuj¢·„[Â#□»□êZ (¢Š (¢Š *+¨>Ói5¿›,^j2y‘6×LŒeOb;□-
Š ó{Ÿƒ¶w’ÛËuã/□Ï%»ù□<°˜c□□yIN□¸®¿Ã~-
ÿ „rÂ[_í□[Tó%2y°¥ÏŸ"ð□Ðp0¼g-¤ÖÍ□□‹ká‹+o□ßx”Ë<·×p%°□0)

kÎÔ d□y9'šÚ¢Š Âðï„ì<56«-œ·R□Ní®æ□È□+·P¼

□®O½s□ð‡C†i□IÕuý□□[sÛi°ƒE□□×å × Q@□ž□ðž‛á+□-t›ržkù“M#—
ʼfþó1äÿ .knŠ(¢Š(¢Š(¢Š(¢Š(¢Š(¢Š(¢Š(¢Š(¢Š(¢Š(¢Š(¬•[ýz□»ýkZªÝY
§V.W□□(□

ŠÔþÈ_ùì□ïš?²□þ{ûæ€2è-Oì…ÿ žÇþù£û!□ç±ÿ ¾h□.°
Oøô‹ýÑTÿ ²□þ{ûæ¯Å•□Çœí□Í >Š(□Š(□Š(
Z¾•ý–¥\X}¶òËÎ];h²—
Ëš>s•l□–+„□f¶rßE}'Œ¼b÷□©Hî□S□DSÔ□Ù□□¥zE□□•¡é?ØzD:□ö…þ¡å□?i¿›Í™òIù›□8Î□°
□[DðÅ–‡©jú„2Ï=Þ«qçÜK;□F□□□ aTd□Éç-mQ@□u

Œ‡8`AÁÁ®{AðfŸáß ËáË□«ï²Ê%-t'ƒ*ù™ÉV Fxâ°:(□Íì~

Yi–‹iaã□Ú[&JÃo©ˆÑrrp□c-u>□ð·ü#"è□okš¯Ÿ·þB·ž□–·?s□ŒçŸ\
ß¢€
(¢€
(¢€
(¢€
(¢€
(¢€
(¢€
(¢€0o¿ãö_¨þU^¶'ÓDÓ4†R7vÅGý•¿óØÿ ß4□—Ej□d/üö?÷ÍÙ
ÿ =•ýó@□uÑAÿ -ñ□¸?•QþÈ_ùì□išÐEÙ□¦s´□@

¢Š(¢Š(¢Š(¬¯□è□ð□iŸbþÔÔôßœ?Ÿ¦Üy2ñž7`ðsÒµh □7_ƒ¶i□‰úxËÆ+y"□Þàj`HÊ:)m™
zW□§Ùÿ gé ÖÖ□h¸¹ò"Xüû—ß,˜□ÜíÝ•sVh

•□xrÏÅZ$šUûÎ-
ï$r□□€l£□□□GQéVµ}2□kF½Òî^D,î□†F‰¶°V□8>¼ÕÚ(□?BÑíü?¡Ùé□□+ÛÚD"□¦mÎ@õ8-
Êªèž□²Ðõ-_P†Yç»Õn<û‰g`HÀÂ¢à

*Œ€9<õ-ª(¢Š(¢Š(¢Š(¢Š(¢Š(¢Š(¢Š(®mÿ Ö7Ô×IYÍ¥+1>iäçîÐ□U□©ý•¿óØÿ ß4
□d/üö?÷Í eÑZŸÙ
ÿ =•ýóGöBÿ Ïcÿ |Ð□]□©ý•¿óØÿ ß4□d/üö?÷Í e×Iúµú
ÏþÈ_ùì□ìšÑQµ@ô□ □¢Š(¢Š(□‡×~□Øëž(—Ä#_ñ

•}$K :}âÄ□ (ù ã$g□æ—
Oøoö□FÚóþ□O□Üù□¬žMÆ©¾90sµ×o*z□é]½□ VEï‡,ï¼M¥ëÒ¼âïMŽXáU`□‰ □¸c'§□"µè

/øRÃÅ•XÃÔkev—qýž@„°ç ä-9ö>õcÄ:-
ü$gØ¿µ5=7ççé·-L¼gØ<ô-Z(Í×àíš_É~ž2ñŠÞH,7¸˜2ŽŠ[fH-•ßéöÛúuµŸÚ..|ˆ-
?>å÷Ë&7;wcÜÕš(¢Š(¢Š(¢Š(¢Š(¢Š(¢Š(¢Š(¢Š(¢Š(¢Š(¢Š(¢Š(¢Š(¢Š(¢Š(
 ¢Š(¢Š(¢Š(¢Š(¢Š(¢Š(¢Š(¢Š(¢Š(¢Š(¢Š(¢Š(¢Š(¢Š(¢Š(¢Š(¢Š(¢Š
(¢Š(¢Š(¢Š(¢Š(¢Š(¢Š(¢Š(¢Š(¢Š(¢Š(¢Š(¢Š(¢Š(¢Š(¢Š(¢Š(¢Š(¢
Š(¢Š(¢Š(¢Š(¢Š(¢Š(¢Š(¢Š(¢›#ùq;ã;TœTwFéX”
´ã- Y¢Š(¢Š(¢Š(¢Š(¢Š(¢Š(¢Š(¢Š(¢Š(¢Š(¢Š(¢Š(¢Š(¢Š(¢Š(¢Š(¢Š
†êco•.ìÅ MEeÿ k7üñ-
÷ÕIo¨´Ó¬f07wÍ hQE QE QE QE QE QE QE QE QE QE QE QE QE QE
QE QE QUo.Í¨B6ì÷ª¿ÚÍÿ <Gýõ@”S!6|cpú (¢Š (¢Š (¢Š (¢Š (¢Š (¢Š (¢Š
 (¢Š (¢Š (¢Š (¢Š (¢Š (¢Š (¢Š (¢Š (¢Š (¢Š (¢Š (¢Š (¢Š (¢©Þ^›YBÈÏZ ¹ET³
¼7Là]£Ö-ÐEPEPEPEPEPEPEPEPEPEPEPEPEPEPEPEPEPEPEPEPEPEPEP
EPEgÜj-

í□Œ□½óQÿ k7üñ-
÷Õ jQY□ŰÍÿ <GýõZ0ÉæÂ’□•Ã8 □ÑE□ QE□ QE□ QE□ QE□ QE□ QE□ QE□ QE□ QE□ QE□
QE□ QE□ QE□ QE□ QEf6ªÊÄyCƒŽ´□§ER³¾72”(□
œæ®Ð□E□P□E□P□E□P□E□P□E□P□E□P□E□P□E□P□E□P□E□P□E□P□E□P□E□P□E□P□E□P□E□P□E□P□E□
P□E□P□E□P□E□P□E□P□E□P□E□P□E□P□E□P□E□P□E□P□E□P□E□P□E□P□E□P□E□P□E□P□E□P□E□P□E
□P□E□P□E□P□E□P□E□P□E□P□E□P□E□P□E□P□E□P□E□P□E□P□E□P□E□P□E□P□E□P□E□P□E□P□E□P□
E□P□E□P□E□ÍxâïÄÖš□• YGu©Ï:Â

„m…□9□ä€q€?-ô□ÒÑ^□â□~,ø#F‟Ä×~*³¿ŠÝ‛®m|¼®
□À□G□#8Áô¯cðö¬5ï

iÚ°•ÊûeºLcÎv□ ‘žø4□¥E□P□E□P□E□P□E□P□E□P□E□P□E□P□E□P□E□P□E□P□wñí/û‡ùU-
'ý\¿QWn?ãÚ_÷□òªZOú¹~¢€4h¢Š (¢Š (¢Š (¢Š (¢Š (¢¹Ï□êúÜ7±i^-
Ò□ÅäÑù□{s•µ¶\‘-#—n>âóß P□GEq□

5½[]ðÅÌúÍÚÝ]Ã¨Ooæ¬K□*□□ÂŒWi@□□ÍxƒÃ□†³~—6ž)Õt¨Ö0†
M›   □•Ü©9ç…p¾6Ò<Aá½.×ì-9×nuKû¸í,-ä1…wcÉ8\à

’~”□ëôTvñ¼VÑG$¦Y□□´„`¹□“øÔ” QE□ QE□ QE□ QE□ QE□ QE□ UMKþ< ›ê?□[ªš—
üy7Ô□ Ä«6-
ñû□Ôÿ *-Vl?ãö/©þT□»E□P□E□P□E□P□E□P□E□P□E□P□EyåÎ»âŸ□ëú†›á+‹M3LÓe6÷:¬ðùÍ$
ãï$HxùsÉ5»á-3Å°mäË¯xŠ×Y´hÿ vâÈ[Ê¯ŸE;Jã>ý(□¦¢Šâµ[?ë□ÅÔz~«aáý.□Û□¢Ùn§Ÿ•-
!ŽÕ□è:ñ@□-□ÁøCÄ> ‹ÅÚ‡ƒüPö×WÖÖË{m}n›□Ä%,’ËÐ0$

=ý2{Ê (¢Š (¢Š (¢Š (¢Š (¢Š (¢Š (¢Š ÍÕþì_Sý+.µ5□»□Ôÿ JË

,×þ="ÿ pTÕ□¯üzEþà©¨ ¢Š(¢Š(¢Š(¢Š(¢Š(¢Šã|uâ=BÄZh^t□ö¢KG#(qk
ó$Ì□□

=IöÅ vTW+ðßY¿ñ□Ãí#TÔæ□^\FÆY□□ÜC°è □À□□u□H±FÒ;□E□˜žÀP□¨¯/Òu□□|Aµ‟[Ñõ»_

è¯#-Š□%¹štV+½÷œ.H=?úçgÁ~$Öf×5O
øœ[¶±§¢M□Í°íK¨□£ã±□ ý}¨□·¢Š(¢Š(¢Š(¢Š(¢Š(¢Š(¢Š(¢Š(¢Š(¢Š(¢Š(¢Š
(¬•[ýz□»ýkZ²uoõéþïõ □i?~_ -JËÒ~ü¿AZ" QE□ QE□ QE□ QE□ QE□ QESÕ¦¾·Òn¥Ó-
Rêùc&□]Â+¿`Oa@□(¯5¾Ó>)ZéÓjÃÅZ[ÜÃ□Jt´Ó‡'Ø□Ø$??·o-uÞ

ñ□ñW„tÝoÉòZî-Ï□9
À•`=²

(□rŠ+□ñ×ˆõ
□i¡x}ÐkÚ‰-□Œ¡Å¬+Ì"0<p8 õ'Û□□ÙQ\¯Ã}fÿ Ä□´□SS˜Myq□□d□□q

Ã¢€□ t□ÕP□E□P□E□P□E□P□E□P□E□P□E□P□E□P□□÷ü~ËõÊ«Õ‹ïøý—ê?•W □°
Oøô‹ýÑ\ýt□Ÿñé□û¢€&¢Š(¢Š(¢Š(¢Š(¢Š(¢¹□-x›QÐâÒ´ý□(-
VÕîÅ-»ÜçÊ‹Œ³¶98ôþxÁæõÕø‘àý-□□Kâ›□f
Eón,eÓÒ P}íŽ¼ä□_×¥ z□□[M½MKK´¿□Y□æ□™U°€Ê□□ó¬/-x¢_
xZ]FÚÝ./-T·¶•Î□Èç ±ô□ŸÃ·Z é¨¯5¼Ò~(ØéòêkãC6ââ□Ì-§6š«
`d¨"ï~'-
…uþ□ñ□ü%>□Óu¿#Èk¸·´yÈV□,□¶AÇµ mÑE□ QE□ QE□ QE□ QE□ QE□ QE□ W6ÿ ë□êk¤®mÿ
Ö7ÔÐ□Ý+þ>_ýÏê+^²4¯øù□÷?¨-z (¢Š (¢Š (¢Š (¢Š (¢Š (¢™,±Á
Í3¬qF¥□Øà(□’O¥ >Šò}□ÆÞ"Ö~$híç□<5«-×Ø-Z□□$p Ä¬Än□›$

ô□Ö½b€
+Ÿñ—‰Ç…tAu□³^_\L-¶VŠpg™øUÏaÔŸa\êh_□î£□sxËM²□†ï°Ã¦,□¯û>c□ß□ z□□□¨¸□p
³□\^×Í1□□¾>m¹ç□Î*j (¢Š (¢Š (¢Š (¢Š (¢Š (¢Š (¢Š (¢Š (¢Š (¢Š (¢Š (¢Š (¢Š
(¢Š (¢Š (¢Š (¢Š (¢Š (¢Š (¢Š (¢Š (¢Š (¢Š (¢Š (¢Š (¢Š (¢Š (¢Š (¢Š (¢Š (¢Š (¢Š
_(¢Š (¢Š (¢Š (¢Š (¢Š (¢Š (¢Š (¢Š (¢Š (¢Š (¢Š (¢Š (¢Š (¢Š *-
¯ý¡ý□yý"äÿ hù-
öo;îy˜ùsíš»\‡•õ□□hv□WÞ□Ò"TXæ?l¶ÚZF□□l ç9ôÏn1š ò□ˆ□¿□î4]Þ(…'Ð!u-î-1ãRÊ

yà·¾p@À$q^åàÝOJÕü¥Ðh^ÑéÆ□HcaÌa>]‡Ü□□Â¼³Zø¡â?□hWš

“à-R;ëèZÚF˜1Hƒ•¬~èìO' u5èÿ

ü37„|□§h÷.¯s□³ÌW vbÄ□¦qžøÍ u□QE □QE □QE □QE □QE □QE □QE □QE □QE □QE Gqÿ
-Òÿ ¸□•RÒ□ÕËõ□vãþ=¥ÿ pÿ *¥¤ÿ «—
ê(□FŠ(□Š(□Š(□Š(□Š(□Š(□Š+šñ/Œ□á□»†ßþ□Ï□êžjoó4»-
=□œaŽáƒí@□□□¿äVÔ¿ì1wÿ ¡× ×Žü6ñ5æ‡húE÷ƒ¼V’^j’Ê³ÿ f□□k+Œ□bF ïÁÇ½z.¯®ßéÞ
Ñ´ëm

êöÚý□g¼ˆ□–
•@Án□\÷#§z Ý¯<Ó•ü%ß□¯5□óéž□ŒÙÛz5Û□Þ°÷UÂþUÕø«S»Ñ¼/¨ßØYÏy{□'ìðA□‘žCÂü dŒ•
O°5KÀ^□o
ø:ÇOœî½`g¼□œ—•þg$÷Á8Ï □□ÒÑE□ QE□ QE□ QE□ QE□ QE□ QE□ UMKþ< ›ê?□[ªš—
üy7Ô□: Ä«6-
ñû□Ôÿ *-Vl?ãö/©þT□»E□P□E□P□E□P□E□P□E□P□E□P□‡ü0Óüs¬x□Þm7Ä□~•e,óÉæ-
•¹žW26âáÈQÏ□□…uÞ-×¼K¤xå|#â›«mDÝZµÕŽ¡□>Q}§

Žƒ€q“Çëž2´k½oá•Ö££Má□WWÑ‰»’çO¹Ò¡□2+œ˜Ýr1ƒßëZž□°Ö¼Kã±ã□gL“I³´´k]:Êv□c¸å¤
p>ï□cü2@=

¡»»·±´šîîd†Þ□/$Žpª£’I¬•;]¿½ñV«¤M¡Ý[YÙ,m

¡!>]É`
Æ8ö'§8¯7ñ^½¨k-1›OÖ¼'â›•□iòþîÛOÓšD¿‘Oß‘‰ Æ1□£ õ>”□Òx□ÞãÄ-'Õ¼ys
Áo{□Ùéq8Ã□U9ó□í½¹□Óê+Ðk□Ð|x°Î«o¦¯„|S§,€□=ö›äÁ□U'□·□g□□u"»
 (¢Š (¢Š (¢Š (¢Š (¢Š (¢Š (¢Š ÍÕþì_Sý+.µ5□»□Ôÿ JË

,×þ="ÿ pTÕ□¯üzEþà©¨ ¢Š(¢Š(¢Š(¢Š(¢Š(□-
¯ªÙèzEÖ©□(ŠÖÖ3$□ì;□Rz□Üšä|!¥^Oe©ø»Z^¦¯¬DJDßòéj□òâ-‡-
3z"ÏJêõ½□Nñ-'>•«[}¢Ê}¾d[Ù7m`Ã•
Ž@ï\]ÇÁ?‡ëm)‹ÃÇÌ□JâòàóŽ?Ž€.|-ÿ 'O □k)?ôk×gun—
v"[>BK□FØë,1^uð□ÀÚ□‡ü1a«Ë¤Íe¯Ü[¼Wm3H¯·Ì$□Œp¼*ö-u¾□×oõ©õXït;--
lîÚ□^rqrf¤‹•8>Ù-æ€8
ëšÿ ÃÝ□?

k-□Öõ□²gKkÝ&□<sFX°''´óÞ—@Ôõ-kãªÞÞè"éñO²¬3È-#ÇçŒ3□û¤±#□ž+i¾$j±-·"áï‰þÜ
□c•^□□ë¶q•|Uï□hZ²jz—
Š<H'E«êA#KXŸzÚ@Ÿv=ÝÉ'$Ž3@□•□Q@□□Q@□□Q@□□Q@□□Q@□□Q@□□Q@□□Q@□□Q@□□Q@□□Q@□
□Q@□dêßëÓýßëZÕ"«□¯O÷□ ;Iûòý□jV^"÷åú
Ô □Š(□Š(□Š(□Š(□Š(□Š+3Ä7□½®‰q>…g□î£-ÖŽÞgØ²□ÃpÝØíÎ3Æq@-{â‹o‹□šmÄ

š□–

□f‹G–Hî¤ˆõUiAPHô□ô®ÃÀ:–‹©ø3O}□',Â□ò□ □-□^
·ûYïß9ïX□âN´ð□"øwâ_í20#xT[î÷›8Ç¾+[áï‡/|-á6‹Q)&¥uq-
íÒD~Q#œí_ }h□¡Õõ[=□H°Õ/å□ZÚÆd‘½‡aêO@;"\•„4«Éìµ?□kQ□Õõˆ‰H›þ]-
@>\CÐãæoRyéWôýŸ□<□ðø›Ã—:|W2□—O¹‘ÕðŽ
¶@V□€{~"±î> ü?[iL^->`BW□—
□œqüt□sà÷ü’}□þ¹Iÿ £^»Šó_ƒþ□Óü?á‹□^]&k-~âÝâ»išE}¾a
□c…áW°ýkÒ¨ ¢Š(¢Š(¢Š(¢Š(¢Š(¢Š(¢Š(□□ûþ?eú•åUêÅ÷ü~ËõÊ«Ð□]□§üzEþè®~°
Oøô‹ýÑ@□QE□ QE□ QE□ QE□ QE□□Ä|M]□m

ÖÛS7†þK•m-tñ›¯´/CÓ¹<cß□Âê¿ðšÅccÿ
-          n|.€=çö^Ýù□•ûH^JŽ§Ëãë]—Žô½nßÅ--
ñn‹§ÿ j□,M□ÅŠ¸Wd‘pY3ÜsÇSÇ½RÕ|u-kÚEÞ•¤x
ÄK{u
A¿R¶X MÀ,Å‹□□žœf€=□ÊâÚîÆÞâÊHäµ-5x^?°ÈFA-Ø¬□-
Ÿ□Ÿ□^Eâ]æÂm±…ˆ□+HOÈ#□’ù□}9ã5J□ïþ-|0³·´Óg×/tø#ŒÛ[□°V,□□ùIÀÉ=:
¯ñ□GÖu-#BÕô{1.¡¥^Å¨□gÌá|Ü

S=□

ÿ : â.¢ø"‡R□f;û•

–ap–æl ª ›lp$ÆAã®ß ›®Mzß…îôkß

éóx}£:W’□ÜF0□G□py□c□<æ¹3ñ/U’□□·ÃÏ□□@Œ*Ml□□Þò‟Œ{âµ¾□xnóÂþ□ŠËQ1ý°i¤¹•"9HÙ
Û;WØ

~9

¶Š(□Š(□Š(□Š(□Š(□Š(□Š(□¹·ÿ XßS]‰soþ±¾¦€.é_ñòÿ î□QZõ'¥□ÇËÿ ¹ýEkÐ□E
□P□E□P□E□P□E□P□E□P□\□Œ§—Å:í¿•lde•Ô\ëS!ÁŽÛ<D□f•ñþîO
×{\~·ð·Áž"ÕçÕu]□í□³íó‰ûLÉ»
□p®□@;P□f¹□Vß□ü
□□¬pÅgz^Š0□Dj □Ó□èuã□¯Áï

Åãÿ □ZÙørs¢Í□É¾ešv@ÁG—¹÷eyÎ9□÷¯BÖu ü!¤i□š/‡îµ(<è¬„6ìÄÛÅŒ□'

H □Ïâh□”øžú«øçÀvÚ?Ù>ÖóÝ¼_lÝå□X×

Ûyà□#Þ¥Õ4ï‰°.›q«Åâë□Eí£iŸN“LH£uQ’ªàîÎ□□>µ«ñ□BÕu□4□kA…gÕ´K±s□»6ß>20ñç±#-
•cê~5ñ'ˆ´™ô}□Á□åž£u□…®5(„6öá†
‡ÏÍ€N8üúP□qá}v/□xcNÖ¢ŒÆ·□‰

dçctažø ŠÖ¬•
èqøkÂún□□ù,Î□Œ¾1½°±Çl’MkÐ□E□P□E□P□E□P□E□P□E□P□E□P□E□P□E□P□E□P□E□P□E□P□E□P□E□P□E
□P□E□P□E□P□E□P□E□P□E□P□E□P□E□P□E□P□E□P□E□P□E□P□E□P□E□P□E□P□E□P□E□P□E□P□E□P□
E□P□E□P□E□P□E□P□E□P□E□P□E□P□E□P□E□P□E□P□E□P□E□P□E□P□E□P□E□P□E□P□E□P□E□P□E□P
□E□P□E□P□E□P□E□P□E□P□E□P□E□P□E□P□E□P□E□P□wñí/û‡ùU-
'ý\¿QZ4 □AŠ (¢Š (¢Š (¢Š (¢Š (¢Š (¢Š (¢Š (¢Š (¢Š (¢Š (¢Š (¢Š (¢Š (¢Š (¢Š
(¢Š *¦¥ÿ -Mõ-
Î-ÐFzÐ□3Vl?ãö/©þU¹µ□?*6•ØP□ÑE□ QE□ QE□ QE□ QE□ QE□ QE□ QE□ QE□ QE□ QE□
QE□ QE□ QE□ QE□ QE□□›«ýØ¾§úV]t¤□Ôf□«ýÑùP□V¿ñé□û,¦¢Š (¢Š (¢Š (¢Š (¢Š (¢
Š (¢Š (¢Š (¢Š (¢Š (¢Š (¢Š (¢Š (¢Š (¢Š (¢Š (¢Š (¢Š (¢Š (¢Š (¢Š +'Vÿ ^Ÿîÿ
ZÖ¤ - □ ÌÒ~ü¿AZ”€□Ð□K@□□Q@□□Q@□□Q@□□Q@□□Q@□□Q@□□Q@□□Q@□□Q@□□Q@□□Q@□□Q@□□Q@
□□Q@□□Q@□□Q@□□Q@□7ßñû/Ô□*¯].Ð{
6¯÷Gå@□Õt□Ÿñé□û¢¥Ú¿Ý•-
□QE □QE □QE □QE □QE □QE □QE □QE □QE □QE □QE □QE □QE □QE □QE □Í¿úÆúšé)6
¯ ü¨□'Jÿ •—ÿ súŠ×¤

€
Z (¢Š (¢Š (¢Š (¢Š (¢Š (¢Š (¢Š (¢Š (¢Š (¢Š (¢Š (¢Š (¢Š (¢Š (¢Š (¢Š (¢Š (
¢Š (¢Š (¢Š (¢Š (¢Š (¢Š (¢Š (¢Š (¢Š (¢Š (¢Š (¢Š (¢Š (¢Š (¢Š (¢Š (¢Š (¢Š
(¢Š (¢Š (¢Š (¢Š (¢Š (¢Š (¢Š (¢Š (¢Š (¢Š (¢Š (¢Š (¢Š (¢Š (¢Š (¢Š (¢Š (¢Š
(¢Š (¢Š (¢Š (¢Š (¢Š (¢Š (¢Š (¢Š (¢Š (¢Š (¢Š (¢Š (¢Š (¢Š (¢Š (¢Š (¢Š (¢
Š (¢Š (¢Š (¢Š (¢Š (¢Š (¢Š (¢Š (¢Š (¢Š (¢Š (¢Š (¢Š (¢Š (¢Š (¢Š (¢Š (¢Š (
¢Š (¢Š (¢Š (¢Š (¢Š (¢Š (¢Š (¢Š (¢Š (¢Š (¢Š (¢Š (¢Š (¢Š (¢Š (¢Š (¢Š (¢Š
(¢Š (¢Š (¢Š (¢Š (¢Š (¢Š (¢Š (¢Š (¢Š (¢Š (¢Š (¢Š (¢Š (¢Š (¢Š (¢Š (¢Š (¢Š
(¢Š (¢Š (¢Š (¢Š (¢Š (¢Š (¢Š (¢Š (¢Š (¢Š (¢Š (¢Š (¢Š (¢Š (¢Š (¢Š (¢Š (¬
+â~Û''¨þU_qõ4□ÒÑPÚ□Ç¤_îŠš€
(¢€
(¢€
(¢€
(¢€
(¢€
(¢€
(¢€
(¢€
(¢€
(¢€
(¢€
(¢€
(¢€
(¢€
(¢€
(¢€
(¢€
(¢€
(¢€
(¢€
(¢€
(¢€
(¢€
(¢€
(¢€
(¢€
(¢€
(¢€
(¢€
(¢€
(¢€
(¢€
(¢€
(¢€
(¢€
(¢€
(¢€
(¢€
(¢€
(¢€
(¢€
(¢€
(¢€
(¢€
(¢€
(¢€
(¢€
(¢€

(¢€
(¢€
(¢€
(¢€
(¢€
(¢€
(¢€
(¢€
(¢€
(¢€
(¢€
(¢€
(¢€
(¢€
(¢€
(¢€
(¢€
(¢€
(¢€
(¢€
(¢€
(¢€
(¢€
(¨o.íì,§¼»•b·,6’Y□ðª□I?… PÖ¼M¢xt@u□RÚËÏ$Eç8RøÆqôÈüëQX2†R□# ŽõòŸ□-
Qñ¦™wñ□óÌ‡O7ÑØivì:Ä7□oÌ{ò[Ò¾¥±ÿ •}·ýr_ä(□Å□Q@□□Q@□□Q@□□Q@□□Q@□□Q@□□Q@□□Q@□□
Q@□□Q@□□Q@□□Q@□□Q@□□Q@□□Q@□□Q@□□Q@□□Q@□□Q@□eÜø—
A³¼û□Ö·¦Áuœy□ÝÆ¯Ÿ÷IÍa|O×nô□□ÞÏ§9Žþá’ÖÝÁÁW‘,ä{□’=À¨ôï…>

³Ñ□N¸Ñ-
/$)‰®ç□tÒ¹ûÍ¿ï□NO□b€;PA ƒ□z□+Îþ□Ëq§Ÿ□xNyÞtÐ¯ü«W□å…»□Ñ©>Ø?Ÿµz% □QE □QE □
QE □QE □QE □QE □QE □QE □QE □QE □QE □QE □QE □QE □QE □Q\□Å+Û¶Ó´□

ØÜ½´°ö¡□”³FpÉ□Y□>¸Àú□@□Bø›@{ï°®¹¦µæí¿g□q™3é·9Íj×□sðŸÁSèm¥¦ƒi□òö%Äq□:œpÞ
gÞ'¿&£øOªÞê~□H5)L×°mÌ¶□JÇ%Ìg Ÿ}¤□Â€;Š(¢€
(¢€
(¢€
(¢€
(¢€
(¢€
(¢€
(¢€
(¢€
(¢€
(¢€
(¢€
(¢€
(¢€
(¢€
(¢€#žxm y§•"‰□Yä`ª£Ô"Ò©iúþ□«ÈÑé°½…ì^2Ëmr'□ú…&¸O□ÙÇãoŠÐxkP,ú&"b/®-
ƒ□·□³a□ã¨□ŸÏÖ¡ø□à□#Gð¼Þ$ðÞŸm¤ë□>Û¨&³ŒE¹T□ÊÁp□□Ï_äM z•□SJ¾]OH²Ô□an I€ô

¡¿-[□Š(□Š(□Š(□Š(□Š(□Š(□Š(□Š(□Š(□Š(□Š(□Š(□Š(□Š(□Š(□Š(□Š
(□Š(□Š(□Š(□ ¼½´Óíšâöê□h□ïK4□□}Iâ§¯/ƒKµø□ñ?]}j1w¥x□Êµ´³‟˜šf]Ò;/BGN{c
Ò€=□NÖt½a□ôÍJÎõSï5´ë]¤ÕÚòo-ø{Lð4úGŒ¼;g

›5¥ìPÞÇj¾\sÛ¹ÚÁ”qžœã¿°¯Y □Š(□Š(□Š(□Š(□Š(□Š(□Š(□Š(

□ïøý—
ê?•W«□ßñû/Ô□*¯@□□§üzEþè©ª□Oøô‹ýÑSP□E□P□E□P□E□P□E□P□E□P□MGUÓ´{as©ßÚÙ@X –
æe•K-ƒ,@Ï□ò¬Èüoá)dXãñFˆîÄ*ªê□□Iè Ý\ÇÅÁdm¼&5/³ý‡þ□+o´}§o•³d›·nãn:çŠÕÒô¯†
ú•ÈþÈ°ð¥Ôèw□²Cnì¤wùFE v□t¢¼óâÄóÏkáÿ □¥Ä–
ÖÚÖ«□¥Ü±¶Ö1–¨□mÜ□* ë ñ6□s|,mõÍ6[²qäGw□IŸM æµk‡Õþ□xFûÃÓiöz%□œâ"–î Œ,±¸–
+oûÇœu'5káŽ¹qâ/‡ZF¡xåîŒm□ÌÝY£b™>çnOÖ€:ê(¢€
(¢€
(¢€
(¢€
(¢€
(¢€
(¢€
(¢€
(¢€
(¢€
(¢€
(¢€
(¢€
(¢€
(¢€
Í¿ñ

‰¥N°j:ÆŸg3r#¸¹HØþ

EWñv´Þ□ð†-« □KKW'0z□ÇËŸlâ¹_□ü8ÐÂö—°æ™mªêÚŒ+uyu{□•ÙÜn
□è□qÆ:P□ Å,sÄ²Å"É□Œ«¡È#Ô□}y§•-
ÿ á□øƒâ/□Û»•(C-£a□±o!Xíu□öÜGåêMz] □QE □QE □QE □QE □QE □QE □QE □QE □QE □
QE □QE □QE □QE □QE □QE □QE □QE □QE □QE □QE □QE □QE □QE □QE □QE □QE □QE
□QE □QE □QE □QE □QE □QE □QE □QE □QE □QE □QE □QE □QE □QE □QE □QE □QE □QE
□QE □ã□-u}PYé¾´±½'Âñ¼Ûém¢,Y□†□-™ïÏ¢û×²Q@0|Añõ®¯à;□

Ùx[RÒ-mn"15Âávª°ÛÓ–9Ï¿5î_□ü_ÿ ~ˆÓÿ dÞéÿ f+□.W□O”□©î?úÔß‰-

›Ç^□M&
Èí$K”œI"□□h#□□½úWW□^M¼Qg;□.}p1@□QE□ QE□ QE□ QE□ QE□ QE□ QE□ QE□ QE□ QE□
 QE□ QE□ QE□ QE□ QE□ QE□ QE□ QE□□çŸ□□Gà4½ÚLvZ…µÄ˜-
Â□□ý˜W Ç"K□É□□G□•”ä□z□¯©i¶š¾™s§_B&µ¹ŒÇ,g°Ÿåõ®□□†Þ"±³□^Ÿñ□S·Ñ”lKsk□Ì‰ýÕ›
;†□□□Š “áã
ß□øÿ U^î·—QŠÙ□t-
□l□ßB½□²¼9áÝ;ÂÚ$□N—
□KhrrÇ,ìy,Ç¹&µh ¢Š(¢Š(¢Š(¢Š(¢Š(¢Š(¢Š(¢Š(¢Š(¢Š(¢Š(¢Š(¢Š(¢Š(
 ¢Š(¯:ø˜ÂÏÄ^□Ô¥;máÖD.ç¢™□ ŸÈ×¢ÖO‰<;§ø«B¸Ò58Ù-¦□”8daÈe=ˆ4□ªÌ¨¥˜…P2I8 Wž
ü□ÌÞ□Ôõ%□ÉÔµ›»¸Oª□
□æ¦£□†þ"»³þËÔ~!êw:1□-ÝmcI]?°Ód±Èàç-w°n□i¤i¶Úu„+□-´b8£^Š£üõ
TQE □QE □QE □QE □QE □QE □QE □QE □QE □QE □QE □QE □QE □QE □QE □QE yÖœÂÏãî
³

§k_èÐÍ

□ ̂#m
~¿•jüT½ŠÇá‡ˆ$•€□Z´+žìøP□âjÇ‹¼□oâ‰l¯b¾¹Ó5{□&ÖþÛ□Ó=T•âSéþ'9□|9¿¿Ô¬îü[â«-~
+)□¶ö†Ù-¡Þ:3ªçy-ÿ Ë"€:¿

ÚI§øSG²˜□-
½Œ18=Š □ùV¥□P□E□P□E□P□E□P□E□P□E□P□E□P□E□P□E□P□E□P□E□P□E□P□E□P□E□P□E□P□E
□P□E□P□E□P□E□P□E□P□E□P□^uà□□¿□<□$HvÏöø®,ž¥$L,=°~uèµÇø›À□Û:Ü:þ'¬\èšÜQù&ê
Ý□¬±õŰèxl□æà`□/ãC ü□□”‡7□–£mm□

¬ÅÃqÿ |×¢W□£ü?ž=~ß]ñ/ˆnµýFÐ□iæB˚C□=Yc^7{ÿ €Ço@□□Q@□□Q@□□Q@□□Q@□□Q@□□Q@□□Q@□
□Q@□□Q@□7ßñû/Ô□*¯V/¿ãö_¨þU^€:
Oøô‹ýÑST6Ÿñé□û¢¦ □Š(□Š(□Š(□Š(□Š(
Z¦□¦kvËmªéö×Ð#‰□;˜−E

 àŽ¸'ó¯=ø•àO

Xø7QÖ´í:×HÔ´è□ÍµÕŠ□□d^@ùp

O□ú×Yâ□

êšÔ¶³é>&½Ñg·

?r‹$rg}□,F8úšÁÿ …o©jóB<_âûÍvÎ'□
$µKXd#‘æ*gpö

¿Ã·w□‡††«ÛµÛsqg

3

C

È
qõ&ªx³Â––0ÑN›~ÒÇ¶Eš ám²C"ôu>¼ŸÎ¤ñ>□w®øvãLÓµyô{‰6ì»¶□|`0$

□y□□□Ö™-èwúž‡oeg¯Þi×□20½…C3•R

õ<09É- P□#{á?□Øiw□k?□u□¤ÛDÒKäYÅ□Û□dþødç□ëGàþ□. ›ð»FŽee’d{Œ7\HìËÿ Ž‘Uÿ á[
êz¼‘¯‹üa{®YFáþÄ-Ék

„□•0'ß-Õè
ª^¨Š□T` 0 □¢Š(¢Š(¢Š(¢Š(¢Š(¢Š(¢Š(¢Š(¢Š(¢Š(¢Š(¢Š(¢Š(¢Š(¢Š(
□‘ø£k%çÃ□Å□%…£I□è¸cú□ZÞ□¼‹Pð†□w
□Ž[(Xc·È8ü:V´‘¤Ñ<r"¼n
²°È õ□¼ê□†šÆŠ%´ðÇ□ï´□%Ý™lžÒ;□□NHGc•□ÿ $Ð□ô-
□ß-uë˜Nèì4xlåaÐ;¿˜□× ×¢V□„ü%aá□6KkI&¸žâC5ÕÝÃn-
âCÕ˜ÿ Op¹-ê (¢Š (¢Š (¢Š (¢Š (¢Š (¢Š (¢Š (¢Š (¢Š (¢Š (¢Š (¢Š (¢Š (¢Š (¢Š
 (¢Š (¢Š (¢Š (¢Š (¢Š (¢Š (¢Š (¢Š (¢Š (¢Š (¢Š (¢Š (¢Š (¢Š (¢Š (¢Š (¢Š (¢
Š (¢Š (¢Š (¢Š (¤$□’p)<Äþð □QMó□ûÂ•1?¼(□ÔS|Äþð£ÌOï
u□ß1?¼(ó□ûÂ€□E7ÌOï
<Äþð □QMó□ûÂ•1?¼(□ÔS|Äþð£ÌOï
u□ß1?¼(ó□ûÂ€□E7ÌOï
<Äþð □QMó□ûÂ•1?¼(□ÔS|Äþð£ÌOï
u□ß1?¼(ó□ûÂ€□E7ÌOï
<Äþð □QMó□ûÂ•1?¼(□ÔS|Äþð£ÌOï
u□ß1?¼(ó□ûÂ€□E7ÌOï
<Äþð □QMó□ûÂ•1?¼(□ÔS|Äþð£ÌOï
u□ß1?¼(ó□ûÂ€□E7ÌOï
<Äþð □QMó□ûÂ•1?¼(□ÔS|Äþð£ÌOï
u□ß1?¼(ó□ûÂ€□E7ÌOï
<Äþð □QMó□ûÂ•1?¼(□ÔS|Äþð£ÌOï
u□ß1?¼(ó□ûÂ€□E7ÌOï
<Äþð □QMó□ûÂ•1?¼(□ÔS|Äþð£ÌOï
u□ß1?¼(ó□ûÂ€□E7ÌOï
<Äþð □QMó□ûÂ•1?¼(□ÔS|Äþð£ÌOï
u□ß1?¼(ó□ûÂ€□E7ÌOï
<Äþð □QMó□ûÂ•1?¼(□ÔS|Äþð£ÌOï
u□ß1?¼(ó□ûÂ€□E7ÌOï
<Äþð □QMó□ûÂ•1?¼(□ÔS|Äþð£ÌOï
u□ß1?¼(ó□ûÂ€□E7ÌOï
<Äþð □QMó□ûÂ•1?¼(□ÔS|Äþð£ÌOï
u□ß1?¼(ó□ûÂ€□E7ÌOï
<Äþð □QMó□ûÂ•1?¼(□ÔS|Äþð£ÌOï
u□ß1?¼(ó□ûÂ€□E7ÌOï
<Äþð □QMó□ûÂ•1?¼(□ÔS|Äþð£ÌOï
u□ß1?¼(ó□ûÂ€□E7ÌOï
<Äþð □QMó□ûÂ•1?¼(□ÔS|Äþð£ÌOï
u□ß1?¼(ó□ûÂ€□E7ÌOï
<Äþð □QMó□ûÂ•1?¼(□ÔS|Äþð£ÌOï
u□ß1?¼(ó□ûÂ€□E7ÌOï
<Äþð □QMó□ûÂ•1?¼(□ÔS|Äþð£ÌOï
u□ß1?¼(ó□ûÂ€□E7ÌOï
<Äþð □QMó□ûÂ•1?¼(□ÔS|Äþð£ÌOï
u□ß1?¼(ó□ûÂ€□E7ÌOï
<Äþð □QMó□ûÂ•1?¼(□ÔS|Äþð£ÌOï
u□ß1?¼(ó□ûÂ€□E7ÌOï
<Äþð □QMó□ûÂ•1?¼(□ÔS|Äþð£ÌOï
u□ß1?¼(ó□ûÂ€□E7ÌOï
<Äþð □QMó□ûÂ•1?¼(□ÔS|Äþð£ÌOï
u□ß1?¼(ó□ûÂ€□E7ÌOï
<Äþð □QMó□ûÂ•1?¼(□ÔS|Äþð£ÌOï
u□ß1?¼(ó□ûÂ€□E7ÌOï
<Äþð □QMó□ûÂ•1?¼(□ÔS|Äþð£ÌOï
u□ß1?¼(ó□ûÂ€□E7ÌOï
<Äþð □QMó□ûÂ•1?¼(□ÔS|Äþð£ÌOï
u□ß1?¼(ó□ûÂ€□E7ÌOï
<Äþð

û•:I®-Eu□»□□û&_ùèŸ-jy‰ýáG˜ŸÞ□ Ø#1@ˆH%F8©)¾b□xQæ'÷… :Šo˜ŸÞ□y‰ýá@

¢ ›æ'÷…–b□xP□¨¦ù‰ýáG˜ŸÞ□ ê)¾b□xQæ'÷… :Šo˜ŸÞ□y‰ýá@

¢ ›æ'÷…–b□xP□¨¦ù‰ýáG˜ŸÞ□ ê)¾b□xQæ'÷… :Šo˜ŸÞ□y‰ýá@

¢ ›æ'÷…–b□xP□¨¦ù‰ýáG˜ŸÞ□ ê)¾b□xQæ'÷… :Šo˜ŸÞ□y‰ýá@

¢ ›æ'÷…–b□xP□¨¦ù‰ýáG˜ŸÞ□ ê)¾b□xQæ'÷… :Šo˜ŸÞ□y‰ýá@

¢ ›æ'÷…–b□xP□¨¦ù‰ýáG˜ŸÞ□ ê)¾b□xQæ'÷… :Šo˜ŸÞ□y‰ýá@

¢ ›æ'÷…–b□xP□¨¦ù‰ýáG˜ŸÞ□ ê)¾b□xQæ'÷… :Šo˜ŸÞ□y‰ýá@

¢ ›æ'÷…–b□xP□¨¦ù‰ýáG˜ŸÞ□ ê)¾b□xQæ'÷… :Šo˜ŸÞ□y‰ýá@

¢›æ'÷…–b□xP□¨¦ù‰ýáG˜ŸÞ□ ê)¾b□xQæ'÷… :Šo˜ŸÞ□àA□□(¢Š(¢Š(¢Š(¢Š(¢Š(¢Š
(¢Š(¢Š(¢Š(¢Š(¢Š(¢Š(¢Š(¢Š(¢Š(¢Š(¢Š(¢Š(¢Š(¢Š(¢Š(¢Š(¢Š(¢
Š(¢Š(¢Š(¢Š(¢Š(□"□ªj©Væÿ TÕR€
(¢€
(¢€
(¢€
(¢€
(¢€
(¢€
(¢€
(¢€
(¢€
(¢€
(¢€
(¢€
(¢€
(¢€
(¢€
(¢€
(¢€
(¢€
(¢€
(¢€
(¢€
(¢€
(¢€
(¢€
(¢€
(¢€
(¢€
(¢€
(¢€
(¢€
(¢€
(¢€
(¢€
(¢€
(¢€
(¢€
(¢€
(¢€
(¢€
(¢€
(¢€
(¢€
(¢€
(¢€
(¢€
(¢€
(¢€
(¢€
(¢€
(¢€
(¢€
(¢€
(¢€
(¢€
(¢€

(¢€
(¢€
(¢€
(¢€
*9'Š□,YQ
¶Ô

Àn>ƒÔÕMCZÓ4™-!Ô/¡¶’îO*Ýdl□□Ž□æ?: ¿EPÔ5-3IšÒ□Bú□i.äò-ÖFÁ‘¸à~có«ô QE□ QE□ QE□ QE□ QT5□kLÒ□Ùu□ØmšêA□□VÇ˜ç°«ô QE□ QE□ QE□ QEEus□•¤×W2,pA□I#·EP2Iü□ KEfx□_±ñ6•

«¦´•i1`□"□'i*x>àÕèî-æ•âŠxžHþú+,Wê;P□´R3□RÌ@P2I<□Uo5;[□&ãT’Mö□BÓ»Ä7å□d‘Ž
¼
·EgèšÕ□^t{mWM—Í´¸RQ^Áà□A□^
ŠÐ □Š(□Š(□Š(□Š(□Š(□Š(□Š(□Š(□Š(□Š(□Š(□Š(□Š(□Š(□Š(□Š(□Š(
□Š(□Š(□Š(□Š(□Š(□Š(□Š(□Š(□Š(□Š(□Š(□Š(□Š(□Š(□Š(□Š(□Š(□Š(
□Š(□Š(□Š(□Š(□Š(□Š(□Š(□Š(□Š(□Š(□Š(□Š(□Š(□Š(□Š(□Š(□Š(□Š
(□Š(□Š(□Š(□Š(□®Gþ-~•N®Gþ-~” ê(¢€
(¢€
(¢€
(¢€
(¢€
(¢€
(¢€
(¢€
(¢€
(¢€
(¢€
(¢€
(¢€
(¢€
(¢€
(¢€
(¢€
(¢€
(¢€
(¢€
(¢€
(¢€
(¢€
(¢€
(¢€
(¢€
(¢€
(¢€□7ú¦ª•noõMU(¢Š(¢Š(¢Š(¢Š(¢Š(¢Š(¢Š(¢Š(¢Š(¢Š(¢Š(¢Š(¢Š(¢Š(
¢Š(¢Š(¢Š(¢Š(¢Š(¢Š(¢Š(¢Š(¢Š(¢Š(¢Š(¢Š(¢Š(¢Š(¢Š(¢Š(¢Š(¢Š
(¢Š(¢Š(¢Š(¢Š(¢Š(¢Š(¢Š(¢Š(¢Š(¢Š(¢Š(¢Š(¢Š(¢Š(¢Š(¢Š(¢Š(¢Š(¢
Š(¢Š(¢Š(¢Š(¢Š(¢Š(¢Š(¢Š(¯™t□□êú□Å
•JöúêM-]NK;¡$¬cPÌpH<

u-ÊE}5_=x{Ã?ð•ø□â.ž‰°é5□=¯□ù¨d □¨Êÿ À¨□èZù¯âï‹õmOÅW#J½¹ƒLÑæKFx%(

í¸’qŽ~Fð□zîôO‰k□Á'Ö¥”□NÅ>Ãµ¹-8□CÏ^□côjâ<Yá§ðïÀ-!®Tý¿PÕc¼¹fûÙh¤Ú

yáqÇ©4□éŸ□,<;}«xMµÝBòÖu¼"ÑmÓ"F%2□ãŽBóî□

¯Œ?ò2ø□þÂgÿ BŠ›ñ{þF-
ÿ ØGÿ fŠ□ñ‡þF_□ÿ ØLÿ èQP□ñ‡þF_□ÿ ØLÿ èQWqâ_□h~□khõK-Yî[□A

fI□¶vŽqŸþµyçÇ[‹‹Kÿ □ÜZAçÜÅy#Å□ ÞÀÆ@ãÔÑð†ÊÃÅ7·¾/ÕïF¡â5˜£C À³-
Ãµ}ÇCÐ`□È4□ì•Ïk>7Ð<=-[éZ-ð¶¸¸…§Vu!□.z·@~S□ßñ□Ð×‹üDÑ¬µÿ •Þ□Óu□Ì-
"Yþñ□cp□#c#±Å uúoÅÿ □jššXAª”-FÙ□Í
"9íó□ÇãŠÜñ□‹ô_
Ë`š½Ñ·ût¦(˜©*¯□ÆK-Š□G&¸Ï‹¾□Ð¢øi}sm¥ÙÚÏaå½¼□Ä±•ùÕHÈ□,□ñëƒ\w□□¼EáŸ†1_I!7˜Š
i3ó□ˆ”¶OsÖ€=S@ø—á□□ëM¤éWÏ-ÐRÊ□□Up:□Hþx©¼Mñ□Ã^□•`Õµ□Žå—p,5.øõ
tüqšÓÓ<7¢hë Óô«;v,?*9□□

□¸ÝŒœ÷çžõå_□´«□j~"ñV³m□åô·¥#□
□B:ð□N □@¸□è□Ð¼3ñ□Ã^.• Òu□%Ê.æ,D(øõ õü3ŠŸÄþ6ðÿ „#Œë7ë

’ŒÇ
©waë□Ð{œ
ó_Š:M□…üaá-
□hÖ±YÝ½ï•2Û®Á(Êõ□Ž…□õ□¥ðÞŸiâ¿Ž-)½ÕáŽétÌAm□Ë¹W□hm§□á'êÙë@□Ÿ□<k øÂëÂ/£^‰š
□Q|Ø™
:d®2□oqÅzö¹â-+Ã·°e¦£+¤Ú”ÞE°XËe²□8è>aù×•üYðÖ□¥x›ÂšžŸc□-ÅÆ ±Íä¨Ep□H%G□äóß
<Ö¯Åÿ ù□>ÿ ØLÿ èqP□¥ë□½¦ƒ¤\ê—
îÉkl›äeRÇ-ÀTPx‡KŸÃ±ëÆé"Ó^□7□7È□Ÿ\ôô¬/ŠŸòL5ïú÷ú□×-ø¦Inþ□ü9Ð<ÖŠÛS‘□fSŽ□h-
ú□?€ □D¶øÍàk›ñj°³&â□e’□XÉ>äqõ8®KÇ□□íì|k£ZiZ•Äv-
□Œš²¤_,ˆ□A□?{€ý?Â½-÷À-□¾Ñ†•.‡f¶ª»SËˆ#§¸qÎ}óÏ|×Ÿ|IÒl,¼sðæÖ
HV/¶¬L6□½Uá 1þ.=h□Ðü3ã}□ÅšmÖ¡¦Ü8·µm³4éåìã99íŽõ„~4x□_ý—
ûUñ•¾p·□/?\gñÅt>$ðòê~□Õtm5a³’î□D(¡□q□g□¡Æ□µy&…-èÞ□Òí¼-ãÿ □%-
ÌÆ/□ªÉ□Ç?x¶3žŸ2-ü(□Þ□•Ð:□ÊÃ Ž„W›ümÕå°ð'Ø-‰ûF©p-
)ÃmûÍ•®□ÿ À«Ñ`x¤·•íÙ□□@cd ©\qŒvÅy?Æ $ñ•ÃèýSê-¸vûð□êh□oÇ+7ƒ>

][iR□^ÒÖ□e•8 □Tfö''ŸSšàõÍ□Oðo†ü
â=□&‡Ty-ÄÏ□¶nD‰¹·sÈ'#èØ¯pÕ´«=sI¹Ó/â□ÚÜ¡I□ÛÔz□y□ÔW”x—
Ã:WÃí"ÓXÔµ•OWþÍm°6•w 1¬ßÁÀ •¸□·
•A@□Ÿ□5‹Ï□ë□ß□4□JÝ^aõ)×‛o□R

=G$dd`□□z

"£YhÚ%¶'i□□vñyJ□ÎG|ú"É?S^+áxüK¤ø□hÖdÿ „«U€ê:µýò—û:□•ˆ(Ç<,}Ø□áÍz-
Ãÿ □ê-!"YÒµ˜ □VÑî|‰Úß"9A,□.yþ□úzâ€9ÿ „36□¬ø«ÁÒ1Ù§^™mC□±□‰—
□□âF½Z¼"@ýßí)âuAò¶œ™ð□

□Þç^·@□□Q@□□Q@□□Q@□□Q@□□Q@□□Q@□□Q@□□Q@□□Q@□□Q@□□Q@□□Q@□□Q@□□Q@□□Q@□□Q@□
□Q@□□Q@□□Q@□□Q@□□Q@□□Q@□□Q@□□Q@□□Q@□□Q@□□Q@□□Q@□□Q@□□Q@□□Q@□□Q@□□Q@□□Q@
□□Q@□□Q@□□Q@□□Q@□□Q@□□Q@□□Q@□□Q@□□Q@□□Q@□□Q@□□Q@□□Q@□□Q@□□Q@□□Q@□□Q@□□Q
@□□Q@□□Q@□□Q@□□Q@□□Q@□\•ýZý*•\•ýZý(□ÔQE □QE □QE □QE □QE □QE □QE □QE □QE
□QE □QE □QE □QE □QE □QE □QE □QE □QE □QE □QE □QE □QE □QE □QE □QE □QE □Q
E □QE 2oõMU*ÜßêšªP□E□P□E□P□E□P□E□P□E□P□E□P□E□P□E□P□E□P□E□P□E□P□E□P□E□P□
E□P□E□P□E□P□E□P□E□P□E□P□E□P□E□P□E□P□E□P□E□P□E□P□E□P□E□P□E□P□E□P□E□P□E□P
□E□P□E□P□E□P□E□P□E□P□E□P□E□P□E□P□E□P□E□P□E□P□E□P□E□P□E□P□E□P□E□P□E□P□E□
P□E□P□E□P□E□P□E□P□E□P□E□P□E□P□E□P□^qð·Ãú®‡©x®]JÉí’ïPß□r?x ¿#□§"½-Š ð«Ï…
□”ß□Þ%•ÿ á□¸°]Bo˜ye€'a_]Å”z+W_ñ£Ãú¯^ü□ig¤Y=ÝÂj□ÊÑ¡ …
Ï'Õ‡ç^•E y¿Ä¿□jÚÎµàÙ´ë'¸ŽÊÿ }Ã)-
»\ÇÉÉéòŸÊ□ñ7Ãú®µ®ø>}:Éî"³Ô<Ë†R?v¥□ääôùOå^□E yÏÄß□ê°Ö»àùôë'¸ŠÏPó.□HýÚ-
C““Óå?•gø»ÁúÏ‡|a□•<□içO+íÔtô!DÀõl{÷ô8o\zµ□□□□4¶ñHñ4Nè□£b
BGCŽ2=«Ä¾*C¬Ïñ□Ã©áù--TX-·f
□Á¤8çŽ@#ž9çŠ÷□ãõ□□Í©üHÑ|R·©□Z|□□ÀP-|îÁ□?í~”□Ák□ÿ □þ!ZEáíKEµÑì□E7—
!¸p§<□Ä‘œ□

àr□nøóÂ□ó\ø□ÛE±’{=&éRB□ýÜkå€NO¢žkÔ(□¼jO

ø×áßŠ5Kÿ iÐjÚ6¥'šö¬À4M’p□AÈÉ Œ,:óŠöZ(□Ètï□xÇÇ-1Ó5ß□ÙÁ¦iÚSy-ÖQ¶Yß
äòqÈ\ç®Ð äš□‰<7âÏ
üA¹ñ□,l¢Ôa¿‹eÝ›0□-3ÆA9*□#□sÆ:úÝ□□àÚî‹ñ+ÅÚÎ‡«júTvö-×©åØÀà˜—
´□ÉôÇ'·Až{¿Šž□Ô|Q£ØÜè¬£VÓ.<ûuf
¿Ô□x□!HÏ□W}E xo^ßâÇ•<;s¦Í [éÖË-n68□rW□‹-'□¯-•{Væ¯ð÷Q×þ□è:|`Zëš\i,+#c

□(Hèzsê□Jõj(□Ç[\øÇ©Z.•‡í,.H &¢Yp□v1\úàaZ_□ü+âKëo
êúRÇ¨jÚ□,IPá|öù `28Ü•□?5z…□□ÁYjÿ □µ□ê—
•¡ÙXëK"ý,ÕÛ‡L®íÙlg□±œ□$Æëò|OñÖŽþ□¼ð□•Œs:y×løUÚÀårÇ□;dã5íôP□□□M□>…§é,C
³¶ŽÜ9□Ý±Bç…y·Ç[I£Ðt}zÝI—
J¿VÏ÷U»ŸmÊ£ñ¯W¬ß□h¶Þ"Ð/´‹¿õ7q□ÉÆvžÌ=ÁÁü(□å¥Ô7ÖP]Û¸x'□e□‡ua□□#^]¢é×¿□~!Ë
â]^Ò{}□Gs□™kq□C$€ó!Sî□úí□í5Ýx7D¼ðç…,t‹ëÄ¼–ÕLbeR¹L□£ŸA□øVí y§ˆ´□wAø›-
Œt}!õk[‹?²ÝÛÃ"¬ŠGB3Ôp¿‘éÅ\øqáÍWO¾ñ□ˆ5«qiy-]y«f□9,0X€XpIÝú□¥wõST,êçI¼·²¸
□÷rÂé

Ì¹□¹□□Ç±æ€<»á¨þÜø£ãO□/Í□Éö(□tuÝÔ¤iùŠõÊæ¼
á(|□áxt”‘f›sK<Ávù’7S•` ü+¥ □Š(□Š(□Š(□Š(□Š(□Š(□Š(□Š(□Š
(□Š(□Š(□Š(□Š(□Š(□Š(□Š(□Š(□Š(□Š(□Š(□Š(□Š(□Š(□Š(□Š(□Š(□
Š(□Š(□Š(□Š(□Š(□Š(□Š(□Š(□Š(□Š(□Š(□Š(□Š(□Š(□Š(□Š(□Š(□Š(
□Š(□Š(□Š(□Š(□Š(□Š(□Š(□Š(□Š(□Š(□Š(□Š(□Š(□®Gþ-~•N®Gþ-~” ê(¢€
(¢€
(¢€
(¢€
(¢€
(¢€
(¢€
(¢€
(¢€
(¢€
(¢€
(¢€
(¢€
(¢€
(¢€
(¢€
(¢€
(¢€
(¢€
(¢€
(¢€
(¢€
(¢€
(¢€
(¢€
(¢€
(¢€
(¢€□7ú¦ª•q×z•Î3Qýœ□{ô
ôU□³□ï~”}œ□{ô
ôU□³□ï~”}œ□{ô
ôU□³□ï~”}œ□{ô
ôU□³□ï~”}œ□{ô
ôU□³□ï~”}œ□{ô
ôU□³□ï~”}œ□{ô
ôU□³□ï~”}œ□{ô
ôU□³□ï~”}œ□{ô
ôU□³□ï~”}œ□{ô
ôU□³□ï~”}œ□{ô
ôU□³□ï~”}œ□{ô
ôU□³□ï~”}œ□{ô
ôU□³□ï~”}œ□{ô
ôU□³□ï~”}œ□{ô
ôU□³□ï~”}œ□{ô
ôU□³□ï~”}œ□{ô
ôU□³□ï~”}œ□{ô
ôU□³□ï~”}œ□{ô
ôU□³□ï~”}œ□{ô
ôU□³□ï~”}œ□{ô
ôU□³□ï~”}œ□{ô
ôU□³□ï~”}œ□{ô
ôU□³□ï~”}œ□{ô
ôU□³□ï~”}œ□{ô
ôU□³□ï~”}œ□{ô
ôU□³□ï~”}œ□{ô

ôU□³□ï~″}œ□{ô
ôU□³□ï~″}œ□{ô
ôU□³□ï~″}œ□{ô
ôU□³□ï~″}œ□{ô
ôU□³□ï~″}œ□{ô
ôU□³□ï~″}œ□{ô
ôU□³□ï~″}œ□{ô
ôU□³□ï~″}œ□{ô
ôU□³□ï~″}œ□{ô
ôU□³□ï~″}œ□{ô
ôU□³□ï~″}œ□{ô
ôU□³□ï~″}œ□{ô
ôU□³□ï~″}œ□{ô
ôU□³□ï~″}œ□{ô
ôU□³□ï~″}œ□{ô
ôU□³□ï~″}œ□{ô
ôU□³□ï~″}œ□{ô
ôU□³□ï~″}œ□{ô
ôU□³□ï~″}œ□{ô
ôU□³□ï~″}œ□{ô
ôU□³□ï~″}œ□{ô
ôU□³□ï~″}œ□{ô
ôU□³□ï~″}œ□{ô
ôU□³□ï~″}œ□{ô
ôU□³□ï~″}œ□{ô
ôU□³□ï~″}œ□{ô
ôU□³□ï~″}œ□{ô
ôU□³□ï~″}œ□{ô
ôU□³□ï~″}œ□{ô
ôU□³□ï~″}œ□{ô
ôU□³□ï~″}œ□{ô
ôU□³□ï~″}œ□{ô
ôU□³□ï~″}œ□{ô
ôU□³□ï~″}œ□{ô
ôU□³□ï~″}œ□{ô
ôU□³□ï~″}œ□{ô
ôU□³□ï~″}œ□{ô
ôU□³□ï~″}œ□{ô
ôU□³□ï~″}œ□{ô
ôU□³□ï~″}œ□{ô
ôU□³□ï~″}œ□{ô
ôU□³□ï~″}œ□{ô
ôU□³□ï~″}œ□{ô
ôU□³□ï~″}œ□{ô
ôU□³□ï~″}œ□{ô
ôU□³□ï~″}œ□{ô
ôU□³□ï~″}œ□{ô
ôU□³□ï~″}œ□{ô
ôU□³□ï~″}œ□{ô
ôU□³□ï~″}œ□{ô
ôU□³□ï~″}œ□{ô
ôU□³□ï~″}œ□{ô
ôU□³□ï~″}œ□{ô
ôU□³□ï~″}œ□{ô
ôU□³□ï~″}œ□{ô
ôU□³□ï~″}œ□{ô
ôU□³□ï~″}œ□{ô
ôU□³□ï~″}œ□{ô

ôU□³□ï~”}œ□{ô
ôU□³□ï~”}œ□{ô
ôU□³□ï~”}œ□{ô
ôU□³□ï~”}œ□{ô
ôU□³□ï~”}œ□{ô
ôU□³□ï~”}œ□{ô
ôU□³□ï~”}œ□{ô
ôU□³□ï~”}œ□{ô
ôU□³□ï~”}œ□{ô
ôU□³□ï~”}œ□{ô
ôU□³□ï~”}œ□{ô
ôU□³□ï~”}œ□{ô
ôU□³□ï~”}œ□{ô
ôU□³□ï~”}œ□{ô
ôU□³□ï~”}œ□{ô
ôU□³□ï~”}œ□{ô
ôU□³□ï~”}œ□{ô
ôU□³□ï~”}œ□{ô
ôU□³□ï~”}œ□{ô
ôU□³□ï~”}œ□{ô
ôU□³□ï~”}œ□{ô
ôU□³□ï~”}œ□{ô
ôU□³□ï~”}œ□{ô
ôU□³□ï~”}œ□{ô
ôU□³□ï~”}œ□{ô
ôU□³□ï~”}œ□{ô
ôU□³□ï~”}œ□{ô
ôU□³□ï~”}œ□{ô
ôU□³□ï~”}œ□{ô
ôU□³□ï~”}œ□{ô
ôU□³□ï~”}œ□{ô
ôU□³□ï~”}œ□{ô
ôU□³□ï~”}œ□{ô
ôU□³□ï~”}œ□{ô
ôU□³□ï~”}œ□{ô
ôU□³□ï~”}œ□{ô
ôU□³□ï~”}œ□{ô
ôU□³□ï~”}œ□{ô
ôU□³□ï~”}œ□{ô
ôU□³□ï~”}œ□{ô
ôU□³□ï~”}œ□{ô
ôU□³□ï~”}œ□{ô
ôU□³□ï~”}œ□{ô
ôU□³□ï~”}œ□{ô
ôU□³□ï~”}œ□{ô
ôU□³□ï~”}œ□{ô
õr?õkô¨þÎ?½úTª6¨–” ´QE □QE □QE □QE □QE □QE □QE □QE □QE □QE □QE □QE □QE
□QE □QE □QE □QE □QE □QE □QE □QE □QE □QE □QE □QE □QE □QE □QE □QE □QE □QE
□QE □QE □QE □QE □QE □QE □QE □QE □T□úÐÜ›au□œ□Ë!
Ýùu©è ¢Š(¢Š(¢Š(¢Š(¢Š(¢Š(¢Š(¢Š(¢Š(¢Š(□9É[y□8!
□ð¬?µ\□Ïgÿ ¾«rãþ=¥ÿ pÿ *çh□oµ\□Ïgÿ ¾¨ûUÇüö□ûê¡¢€&ûUÇüö□ûê•µ\□
Ïgÿ ¾ª□(□oµ\□Ïgÿ ¾¨ûUÇüö□ûê¡¢€&ûUÇüö□ûê•µ\□Ïgÿ ¾ª□(□oµ\□Ïgÿ ¾¨ûUÇüö□ûê¡
¢€&ûUÇüö□ûê•µ\□Ïgÿ ¾ª□(□oµ\□Ïgÿ ¾¨ûUÇüö□ûê¡¢€&ûUÇüö□ûê•µ\□Ïgÿ ¾ª□(□oµ\□
Ïgÿ ¾¨ûUÇüö□ûê¡¢€&ûUÇüö□ûê•µ\□Ïgÿ ¾ª□(□oµ\□Ïgÿ ¾©ð\Î×□ƒ+•X□3ïUªK□øù‹ýñü
è□¢¢Š(¢Š(¢Š(¢Š(¢Š(ª×îÑÚ3#□9□Š³U5/øòo¨þt□•ö«•ùìÿ ÷Õ–
j¸ÿ žÏÿ }T4P□ßj¸ÿ žÏÿ }Qö«•ùìÿ ÷ÕCE Mö«•ùìÿ ÷Õ–
j¸ÿ žÏÿ }T4P□ßj¸ÿ žÏÿ }Qö«•ùìÿ ÷ÕCE Mö«•ùìÿ ÷Õ–
j¸ÿ žÏÿ }T4P□ßj¸ÿ žÏÿ }Qö«•ùìÿ ÷ÕCE Mö«•ùìÿ ÷Õ–

j¸ÿ žÏÿ }T4P□ßj¸ÿ žÏÿ }Qö«•ùìÿ ÷ÕCE Mö«•ùìÿ ÷Õ-
j¸ÿ žÏÿ }T4P□ßj¸ÿ žÏÿ }Qö«•ùìÿ ÷ÕCE oÙ³=¤1Ä’G$Ôõ^Çþ<¢ú□Z±@□□Q@□□Q@□□Q@□
□Q@□□Q@□□Q@□59d‰bØì¹'88¬ïµ\□Ïgÿ ¾ªö¯÷búŸéYt□7Ú®?ç³ÿ ßT}ªãþ{?ýõPÑ@□}ªãþ{
?ýõGÚ®?ç³ÿ ßU□□□7Ú®?ç³ÿ ßT}ªãþ{?ýõPÑ@□}ªãþ{?ýõGÚ®?ç³ÿ ßU□□□7Ú®?ç³ÿ ßT}ª
ãþ{?ýõPÑ@□}ªãþ{?ýõGÚ®?ç³ÿ ßU□□□7Ú®?ç³ÿ ßT}ªãþ{?ýõPÑ@□□³□¶‰˜’JŒ"RÔ6¿ñé□û
,¦ □Š(□Š(□Š(□Š(□Š(□Š(□Š(□Š(□Š(□Š(□Š(□Š(□Š(□³5)¥Žd
#(+ØûÖ•dêßëÓýßë@□~Õqÿ =Ÿþú£íWóÙÿ ïª†Š ›íWóÙÿ ïª>Õqÿ =Ÿþú¨h
¾Õqÿ =Ÿþú£íWóÙÿ ïª†Š ›íWóÙÿ ïª>Õqÿ =Ÿþú¨h
¾Õqÿ =Ÿþú£íWóÙÿ ïª†Š ›íWóÙÿ ïª>Õqÿ =Ÿþú¨h
¾Õqÿ =Ÿþú£íWóÙÿ ïª†Š ›íWóÙÿ ïª>Õqÿ =Ÿþú¨h
¾Õqÿ =Ÿþú£íWóÙÿ ïª†Š ›íWóÙÿ ïª>Õqÿ =Ÿþú¨h
¾Õqÿ =Ÿþú£íW-
óÙÿ ïª†Š ÙÓ$y!rîX†îjígé?ê$ÿ {úV… □QE □QE □QE □QE □QE c^\L—
r*Êà□À□Ú ûUÇüö□ïê•}ÿ -
²ýGòªô□7Ú®?ç³ÿ ßT}ªãþ{?ýõPÑ@□}ªãþ{?ýõGÚ®?ç³ÿ ßU□□□7Ú®?ç³ÿ ßT}ªãþ{?ýõPÑ@
□}ªãþ{?ýõGÚ®?ç³ÿ ßU□□□7Ú®?ç³ÿ ßT}ªãþ{?ýõPÑ@□}ªãþ{?ýõGÚ®?ç³ÿ ßU□□□7Ú®?ç³
ÿ ßT}ªãþ{?ýõPÑ@□□d•$N]Ë□ÝÍ^¬ý'ýLŸïV… □QE □QE □QE □QE □QE □QE □QE □QE □€
÷S‡`&~§ø«~¹·ÿ XßS@□}ªãþ{?ýõGÚ®?ç³ÿ ßU□□□7Ú®?ç³ÿ ßT}ªãþ{?ýõPÑ@□}ªãþ{?ýõG
Ú®?ç³ÿ ßU□□□7Ú®?ç³ÿ ßT}ªãþ{?ýõPÑ@□}ªãþ{?ýõGÚ®?ç³ÿ ßU□□□7Ú®?ç³ÿ ßT}ªãþ{?
ýõPÑ@□}ªãþ{?ýõGÚ®?ç³ÿ ßU□□□7Ú®?ç³ÿ ßU¡¦K$¢]îÍŒc'>µ"ZzGI¿□ë@□tQE □QE □QE
□QE □QE □QE □QE □QE □QE □QE □QE □QE □QE □QE □QE □QE □QE □QE □QE □QE □Q
E □QE □QE □QE □QE □QE □QE □QE □QE □QE □QE □QE □QE □QE □QE □QE □QE □QE □
QE □QE □QE □QE □QE □QE □QE □QE □QE □æ_□üG©éš>—¡h²´:-¹söt‘N□A€À-Ä—
AŸBkÓkÇ~1ºÛxïáÕÜ¬□□õ"ÎÇ¢□$$þŸÊ€□wû=èqø}ÖÊþüëI□èîZP□å□ □Ç
O¾G©®‹à¿Š/<Oà$}FVšòÆvµ’W9i «□ë†Æz□¹5è.ë□3»□U□$ô□¼föxFo
ë7 □□°"□-˜U?û0 □a¢Š(¢Š(¢Š(¢Š(¢Š(¢Š(¢Š(¢Š(¢Š(¢Š(□;•øö—
ýÃü«□®Šãþ=¥ÿ pÿ *çh ¢Š(¢Š(¢Š(¢Š(¢Š(¢Š(¬í□N‹AÐ/µY—
rZÂÒmþñ□□âp?□Ñ®3âÂ»ü1ÖBg;b'-,T'ôÍ PÓ>-Ûø,Â-SÆ3]jZ□Ò‰Z□;Ç□¾y□Š¤c-
Î¶® ·ð□,59tï>Híb’xc¸"Éµ±Â,yÛœqîk.□…þ□Ô,â¹¾kýFâHÃ‹»‹Ù
ò3‘, üª□ÛË¨Xx£Â:¥Ô×¶vW□i□Ò¶d1:œ)oUõ □´o†úV¹¡Újž"žóQÕo!YÐá®]|²Ã
R □8éþ□£à□»ÛkÝsÃ□…Ü—rhó ,yN]à‘w
cÜ€:ûÕ]?Cøƒ¡YÇ¥éÚ¶‡wa□ùpÍ{□¢dAÐax8-¦¢ð□¥úxßÅ÷□…úßÌ-m□—)□•YÂ□Ê□□w!□
ôZ(¢€
’ßþ>bÿ |□Ž¤·ÿ •˜¿ßÎ€:*(¢€
(¢€
(¢€
(¢€
(¢€
©©□Ç"}Gó«uSRÿ •&ú□ç@□”QE □QE □QE □QE □QE □QE □ç6Z[|G¿¿Ôu[»‘ ÁpöÖV0JcI,□
4ŽW""œzW£W□ü:ð&‘â□□ÛÏ-I{xË,±›V°tŠ□□ç
¤rzœúÐ□ h-
Ñü1u,ÚB\@’¦Æ€Ü;Ç×9Ã□Ï¿¹ª^'Ñõm]oÅÆ§ö]□(□’ÐÐ•-
á,äù□ÙsÆÕê:šÅÒ,□ƒ~%ZèZmÍÃhú•"ÍöY¥2□$CÕIä□8ü}†;½_þ@·ÿ õï'þ,h□□á¡ÏÃ•□?óÃÿ
f5Õ×)ðÏþIÆ‰ÿ \?öc]] y¾¹áÍ+Å□□ž³jn-âÐÖTO5Ó□çºÏÊGbk£Ñ>-
ø_ÃºŠê□V™ö{¥R,O>Wàõá˜Šv½àO

xŸPKí^ÀÜN‛□C   äA´□@Â°îMr·š8ø{â]□]
îétÝJõln4ùf2GóôuÏ •ð÷ □k±ÿ •(¾ŸÖ¬U{-
øò‹éýjÅ □QE □QE □QE □QE □QE □QE fêÿ v/©þ•—
Zš¿Ý‹ê□¥eÐ□E□P□E□P□E□P□E□P□E□P□E□Ê|Bñ9ð¯…žê&
sq"ÛA#□V6`~s€N□□z□□(□•ø□<¾+·ÖímäeÑô+i$¸t8□Ý…;cÏp□O¾□wš□Â[x/L¹
•±□z|R;-ÀF5æ·ž3ð5‡Ã[ï□ézÉžáìä@ZÚ`ÓÊÀå˜•ÆI=Ít□w^ô¿□ü+Ô¬ô›³=Í-Šb□□n»□ÀÀ□˜

ò§¦zP□□†ü-¿□t¨üGã   îî-
ï2Ö:Z\4PÛÁœ.B□Y^□'=ëºðÏ„4¿□Ås□‘ö˜í§eo"[†‘##?wq$g<óØWŸø+á□†|Càm□PÖ□PÕ$šÎ
2¢{Ù□CÆ6"© □ŒwéZ~□ŽãÃ□□5ÿ □G{sw¤Ám□å˜¸“{[îá“w§<□os•□E°¸[K9î\□°ÆÒ□:•□kÉü
#à«^Ú□~*ñŒ·:□Ö¢Ï$6âåã†Ò0ÅUQT□LóüòO®:,^Èê□X□AèEy¦™á□□ø:)4Ï
jÚ-Î‹æ3ÛÃ«G/™n□ä¨1ýá’zþT□/,Å×…|}©ø"KÛ‹½4Ù¦£¦›—
ÞñG»cÇ»¸ÝÓéï^‘^Má»□sþ□|òëz¬□…Õ¶‡‰M´-Tp□—+□îx□²yæ½f€
(¢€
(¢€
(¢€
(¢€
(¢€
(¢€
(¢€
(¢€
(¢€
(¢€
(¢€
ÉÕ¿×§û¿Öµ«'Vÿ ^Ÿîÿ Z Ï¢Š(¢Š(¢Š(¢Š(¢Š(¢Š(□‡ñ□ÞøŸÅËákKéì´û[qs©Mn
Ûd“qÂD□¶@$ûU-?á¯†ô•BÞÿ O†êÚê□□æ%Ü‡ÌÇf□□Aî+š-□¶ñ□Å□□Ûê□W±Û^-$□ðNbYÇ-
□ÏŽH□ß½'‰¼3eðý4ý{Ã/sfÉ{□W□¾{¼w□¹Á□1<Ð□¦¿□þ,ø,¾□-
ò{m&ÒÈ]ÝGnå□å™°□‘ÎÐ0□?b3|Iá»O‡VÐxŸÃMqk□¬ñ‹ëO=Þ9áf
r□Ÿ›$`×Gâ?
êw- ·ñ‡u□lõXàû<©p…¡ž<ä□Ç#□¸öô®Gâ□¯•§ðuÊëZ-
‘□RÉ□)i§BäÜ;H¸RÏÈÇÞãû´□ë€† ƒ•y□—™

~T□ÇœìP¹ú
} □QE ki?ê$ÿ {úV…gé?ê$ÿ {úV… □QE □QE □QE □QE □QE `ß□Çì¿Qüª½X¾ÿ •Ù~£ùUz
(¢Š (¢Š (¢Š (¢Š (¢Š *9çŠÖÞ[‰äXá‰
»±ÀU□$š’¼×âO‰´Èµ};Ã:-ëYiÓ¨º¿”#±’ ÇlCh'æe9=€÷

Kÿ µë~"ð×‹nŒ±Ew¬Ã□•nÄ•–ÃqÞG÷œóôÅvÿ □µ+ý/ÁW7□□Iní$qËq□ËC□0

ãß¶}ëŒñgÄ/□ê□^□þÏÔÃCaªE<Ám¥Q□J□È□FqÇ□»-gÆ-□øfÊëIŠ=Pë□}ŽÊ)T¤r±%NðÃ!F

xçñÍ PŸá¯,í¼'y®G}wmq

□hõ…¿rùÛ•lçiÉÇAÎx¯Að□æ§¨x□E»ÖC}¾[eiKŒ3□u•¹\Æ¼ÖËá

¡mg-¡§ê-óß[NnWK°„□9œ□°±‶•ön{t¯Mð_Š□ÅÞ□‡T□Ík6ö†âÝŽ|©Pá—
=Çqìh ñã□ø{âFRA□]É□-Ÿ°jâü5ðwÀz‡…t‹Û-
Ì¸¸²†Y_ís•ÌÈ 8□•Éí^—¨XÛêšmÖŸy-
™mu□C2d□ÈÀ,29□-ÕÂIðOÁk□:}-æ•t□Éum{/˜‡±□˜□Ò€;□+J²Ñ4»}3N‡É³·M‘G¸¶Ñé-
$ŸÄÕÊâ>□ë:-«á‹¨5kƒuw¥ê□iítzÎ##

}ðqŸjíè ¢Š(¢Š(¢Š(¢Š(¢Š(¢Š(®mÿ Ö7Ô×I\Ûÿ ¬o© □ÑE□ QE□ QE□ QE□ QE□ Q
E□□•â}z?

h’Þ´fiØˆ- _½4ÍÂ úŸÐ□á¼□¤]hß□¯#¿¸iõ
•□n¯$' ÊÓr□°áGºª÷>:ðÜÿ □.n5íGìÖú+µ½□±,GÝ7I&;TŽ1µ□>*(>"øQ~(\êçUÿ @“JKe—
ìòó “;q·=;ã□□½¯Ç?‹> ¯…å¼žÛI´²□wQÛ¹F¹fl□$s´

ÏØŠúÏ‡-þ□ÙÇâï

Isi□•Ì+¨Yùîñ\@íµ²□Ÿ›$`öÍlø□Â°□Ï^-üGáÍB□=V8>Ï"\!hg□9□±ÈÁî=½+™ñÕ§Ž.<
¨&³ªèñC4°@–Zt

ÆêF•m]Òr¸ûÜ□v€=è□Ê□9□•j;›ˆlíe¹¸‘b,□2I#œ□P2IöÅ,□ù6ñÅœìP¹õÀ¯,ø-âÝ&□kKð-
±~Ö□]Ê‹ÍJa□±’□Çl+°□ó2œž0□¾(□ŸÔ•î¿â•
xÖðË□7°ì□Ú]³□<»@□ï#ûÒ□»éŽÕîõáÞ4ø□à□NïÂGLÕ•áÓµxn'
i2□¡U`H□□q‘ÀçÚ½‡DÖôÿ □i□j°UÇÚ,§ÝåË±“v□©á€#□{P□…□Q@□□Q@□□Q@□□Q@□□Q@□□Q@□
□Q@□□Q@□□Q@□□Q@□□Q@□□Q@□□Q@□□Q@□□Q@□□Q@□□Q@□□Q@□□Q@□□Q@□□Q@□□Q@□□Q@
□□Q@□□Q@□□Q@□□Q@□□Q@□□Q@□□Q@□□Q@□□Q@□□Q@□□Q@□□Q@□□Q@□□Q@□□Q@□□Q@□□Q
@□□Q@□□Q@□□Q@□r_□<□□•<4tÖ˜[ÝE šÚr2□À#□Ø,GäyÅu´P□ŠMá_Œšžšt
í{L]=ÓÉšè□É$}□È]Ç•¡=Ï&½CÂ^□³ð□†-4["^8□/#

□□ò=h□bŠ(□Š(□Š(□Š(□Š(□ªj_ñäßQüêÝW½‰æ¶dA-
$P□□□oû6çûƒþú□□ƒÜÿ p□ßB€*QVÿ ³n□¸?ï¡GömÏ÷□ýô(□¥□oû6çûƒþú□□ƒÜÿ p□ßB€*QVÿ
 ³n□¸?ï¡GömÏ÷□ýô(□¥□oû6çûƒþú□□ƒÜÿ p□ßB€*QVÿ ³n□¸?ï¡GömÏ÷□ýô(□¥pgÂÞ'Ð5kû
•   j□oØoæ7□Yj(ûb•ýâ…9çÓŠô□iÛŸî□ûèQý›sýÁÿ }
 â|;ákÛMfãÄ□þ —ÚÄÑ□□ÊM±[Åœì@yëÜÿ ŽO□Aã{«‹‹}□øxiÒÅ°□Ï;Îäa¾ï-
Jí¿³n□¸?ï¡GömÏ÷□ýô(□Î¼□£øÛÃðiúUóø}ô‹e(Í□˜ÎG$`•□9#·Jè^Û^>-Žá/-Æ,-
Š½©OÞ□²~lã¦1ßðï]'ömÏ÷□ýô(þÍ¹þàÿ ¾… q:¥¯Ž£Õg›FÔtYl¤ Ç□ü.¦.
2ryÉçÖ Ó|+¬^ëÖ°ßŠõ+{©ìòm,ìã+□,F
å¹céž•Þÿ fÜÿ p□ßB□iÛŸî□ûèP□□□üyEôþµb¡µ□¢¶Dq†□šš€
(¢€
(¢€
(¢€
(¢€
(¢€
(¢€3u□›□Ôÿ JË–•BÞK•-
□ÆsÎ*•ömÏ÷□ýô(□¥□oû6çûƒþú□□ƒ
Üÿ p□ßB€*QVÿ ³n□¸?ï¡GömÏ÷□ýô(□¥□oû6çûƒþú□□ƒÜÿ p□ßB€*QVÿ ³n□¸?ï¡GömÏ÷□ýô
(□□Ä°dÚÏ†u=2ÝÑ&°¶x‛¤$($`g œTÚU‰²Ðl´û□’46±Á&9V!Bž½¸-ŸìÛŸî□ûèQý›sýÁÿ }
 â´•   øóÂ–‡Nð¶¯¢Ï¢³□,-V9|Ë`Ç%T$Þ□'¯å]7ƒ¼!'‡dÔ5-
KPmK\ÔÝ^òì¦Á…□TEì □ž€tÐ!ŽÞ4n¡@5% axv×Ä–Óê§_Ô-
nâ’ížÁ`M¦(;+|£'óúšç□Çâ°□¶□XðÄ□d□y%´¢p=v□“5è□P□9á   Çá‹k§-
ò]CT¾“Î¾¿˜ Ó>00□ÝP8
Ú°:(□Š(□Š(□Š(□Š(□Š(□Š(□Š(□Š(□Š(□Š(□Š(□²uoõéþïõ-jÏÔ-
e¸•Z5□□ÇZ É¢-ÿ fÜÿ p□ßB□iÛŸî□ûèP□J*ßömÏ÷□ýô(þÍ¹þàÿ ¾… T¢-ÿ fÜÿ p□ßB□iÛŸ
î□ûèP□J*ßömÏ÷□ýô(þÍ¹þàÿ ¾… T¢-ÿ fÜÿ p□ßB□iÛŸî□ûèP□J*ßömÏ÷□ýô(þÍ¹þàÿ ¾…
T¢-ÿ fÜÿ p□ßB□iÛŸî□ûèP□□â?
jw□ý¿^¼9□□ž«□?g•.□´7□ç 69□=þž•V/
ø‡\Õ¬¯<]¨X=µŒ¢x,4äq□J:;³òqéÿ ×Ï¡ÿ fÜÿ p□ßB□iÛŸî□ûèP□%¯Áâã{□þ□½Ò„>^Ù-
µ□Ÿ□²~`ÉÏB□=«>ÃÂ°Î¡¬ÚêÞ,ÔíîžÍ¼Ë[□8ÊÁ□Ÿß%¹f□³Ò»ßìÛŸî□ûèQý›sýÁÿ }
 ©E[þÍ¹þàÿ ¾…Ù·?Ü÷Ð
”U¿ìÛŸî□ûèQý›sýÁÿ }
 ·¤ÿ ¨“ýïéZ□SO,Kx•d□%³Ö-Ð□E□P□E□P□E□P□E□P□E□P□□÷ü~Ëõ-
Ê«Ö□ÕŒòÜ»¢,¤ñÍCý›sýÁÿ }
 ©E[þÍ¹þàÿ ¾…Ù·?Ü÷Ð
”U¿ìÛŸî□ûèQý›sýÁÿ }
 ©E[þÍ¹þàÿ ¾…Ù·?Ü÷Ð
”U¿ìÛŸî□ûèQý›sýÁÿ }
 ©E[þÍ¹þàÿ ¾…Ù·?Ü÷Ð
”U¿ìÛŸî□ûèQý›sýÁÿ }
 äüO Üëwz

¶òB‹§ê1ÝÊ$$□E□□¸□ž{â—ÅÞ□-&ÓíÒ+Ç²¾³□nm.Qwyr/LŽâº¿ìÛŸî□ûèQý›sýÁÿ }
âì,~*Ok%ªêÞ□·‰ŽÓy□□´ãŽ¡OÉšéôŸ
Má□□K¢h□€j□)□;Ë•Îë†Éó□`ñ¸ôÁàw-Í>   -ãq Á'#š¹@□ãXx©ü□□¨Ö-bñ□¢y—
¢ ñ;,
|¸□□Æp:ç□•&Ÿñ^ö3i>±á‹□˜mk»8&yÀõ
ÿ .kÐ¨

o
xnÏÂz□¾"d]Ò<³Ë!ËÊìrÎÇÔšÙ¢Š (¢Š (¢Š (¢Š (¢Š (¢Š (¢Š +›□ð□õ5ÒV+é×%Ø„□'ûÂ€
)ÑVÿ ³n□¸?ï¡GömÏ÷□ýô(□¥□oû6çûƒþú□□fÜÿ p□ßB€*QVÿ ³n□¸?ï¡GömÏ÷□ýô(□¥□oû6ç
ûƒþú□□fÜÿ p□ßB€*QVÿ ³n□¸?ï¡GömÏ÷□ýô(□¥□oû6çûƒþú□□fÜÿ p□ßB€*W=□ƒs-
Ä□□□□!û,šrÚ□□ïÞvqŒc-õÖ□fÜÿ p□ßB□ìÛŸî□ûèP□%¯Áâã{□þ□½Ò„>^Ù-
µ□Ÿ□²~`ÉÏB□=©<;àý_T×-õ•□jv÷o`âKK
8ÊÛÇ'8□îå˜vÏJë¿³n□¸?ï¡W´ûimÄž`□v1Î}h□?Äö¾$°,ÄxoPµ²•.Ñîšá7□€gr□"òxôúŠÝ¢Š
æ<]á»½~÷Ã"ZË□.™ªG{0"•Y□□Bà-y□qõ®žŠ(¢Š(¢Š(¢Š(¢Š(¢Š(¢Š(¢Š(¢Š(¢Š
(¢Š(¢Š(¢Š(¢Š(¢Š(¢Š(¢Š(¢Š(¢Š(¢Š(¢Š(¢Š(¢Š(¢Š(¢Š(¢Š(¢Š(¢
Š(¢Š(¢Š(¢Š(¢Š(¢Š(¢Š(¢Š(¢Š(¢Š(¢Š(¢Š(¢Š(¢Š(¢Š(¢Š(¢Š(¢Š(
¢Š(¢Š(¢Š(¢Š(¢Š(¢Š(¢Š(¢Š(¢Š(¢Š(¢Š(¢Š(¢Š(¢'˜"37@2j¯ö•·÷Ïýòh□
Ý□SûJÛûçþù4□i[□|ÿ ß&€-ÑU?´-¿¾□i"Gö•·÷Ïýòh□Ý□SûJÛûçþù4□i[□|ÿ ß&€-
ÑQÃ2N›ÐåsŽ•% □QE □QE □QE □QE □QE □QE □QE □QE □QE □QE □QE □QE □QL-
T†2îp¢€ETþÒ¶þùÿ ¾MÚVßß?÷É
tUOí+oïŸûäÑý¥mýóÿ |š ·ETþÒ¶þùÿ ¾MÚVßß?÷É
tUOí+oïŸûäÑý¥mýóÿ |š ·EE□Äw ˜É8ëÅK@□□Q@□□Q@□□Q@□□Q@□□Q@□□Q@□□Q@□□Q@□□Q@
□□Q@□□Q@□□Q@□□□÷□Û…ó □°qPÿ i[□|ÿ ß&€-
ÑU?´-¿¾□i"Gö•·÷Ïýòh□Ý□SûJÛûçþù4□i[□|ÿ ß&€-
ÑU?´-¿¾□i"Gö•·÷Ïýòh□Ý□SûJÛûçþù4□i[□|ÿ ß&€-ÑU?´-¿¾□i"Vc'eŒ:□©é@

¢Š(¢Š(¢Š(¢Š(¢Š(¢Š(¢Š(¢Š(¢Š(¢Š(¢Š(¢Š(¢Š(¢Š(¢Š(¢Š(¢Š†k¸-Ø
,ŒA#=(□j*§ö• ·÷Ïýòhþ Ò¶þùÿ ¾M [¢ª□i[□|ÿ ß&□í+oïŸûäÐ□°*§ö• ·÷ÏýòjÊ:ÈŠëÐŒŠ u
□Q@□□Q@□□Q@□□Q@□□Q@□□Q@□□Q@□□Q@□□Q@□□Q@□□Q@□□Q@□□Q@□□Q@□□Q@□□ZKè"•£±

:ñMþÒ¶þùÿ ¾M [¢ª□ì[□|ÿ ß&□í+oïŸûäÐ□º*§ö• ·÷Ïýòhþò¶þùÿ ¾M [¢ª□ì[□|ÿ ß&•í+
oïŸûäÐ□º*§ö• ·÷ÏýòjXn¢¸$FIÇ^(□j(¢€
(¢€
(¢€
(¢€
(¢€
(¢€
(¢€
(¢€
(¢€
(¢€
(¢€
(¢€
(ª§Q¶□,Ç#ý"@□¨ªŸÚVßß?÷É£ûJÛûçþù4□nŠ©ý¥mýóÿ |š?´-¿¾□ì"@□èªŸÚVßß?÷É£ûJÛûç
þù4□nŠ©ý¥mýóÿ |š?´-¿¾□ì"@□èªŸÚVßß?÷É§G}□²□V%□N(□Í□Q@□□Q@□□Q@□□Q@□□Q@□□Q
@□□Q@□□Q@□□Q@□□Q@□□Q@□□Q@□□Q@□□Q@□□Q@□□Q@□□Q@□□Q@□□Q@□□Q@□□Q@□□Q@□□Q@□□Q@□□Q@□□
Q@□□Q@□□Q@□□Q@□□Q@□□Q@□□Q@□□Q@□□Q@□□Q@□□Q@□□Q@□□Q@□□Q@□□Q@□□Q@□□Q@□□Q@□□Q@□□Q@□
□Q@□□Q@□□Q@□□Q@□□Q@□□Q@□□Q@□□Q@□□Q@□□U=WV°ÐôÙµ□Nê;[HFé%□ð?ÄŽ€

M \¢¼«CøÊž#ø…§è□□<©¦Þ,†;Ë,Uä
®w*ã□r„uüºWªÐ□E□P□E□P□E□P□E□P□E□P□E□P□E□P□E□P□E□P□E□P□E□P□w-
ñí/û‡ùW;]□Çü{KþáþUÎÐ□E□P□E□P□E□P□Ö™ÿ -□ð#W*ž™ÿ -□ð#W(¢Š(¢Š(¢¸-WÇ:'ë□
Z_‡<'□ÍhÛ.&2-´
Ø□h'þñ□□ U¯ xâ/□]ßi—ZmÎ"¬Ø`ÜØÜ□HSÑ•‡

¾þãÔP□WEr^ ñ•Ö•¬□ch¾□¾Öõ%ŒK"ÄÂ(bSœo•¸□ààT>□ñóêž
□□kz□Î‡-□¼èíæ‘eI□u)"ðØþ‡Ðà□³¢Š(¢¸K/‰pj?□"Ã6škÉhí4K©yØG–
%□"ªã•2□sÖ»º (¢Š (¢Š (¢Š (¢Š (¢Š (¢Š *¦¥ÿ -Mõ-
Î-ÕMKþ<›ê?• bQE□ QE□ QE□ QE□□©¤ýÉ~¢´«7Iû'ýEiP□E□P□E□ÁOñ□Y¼¹¸_□x'PÕ--
ähÞîYÒÕ□©Áò÷òã Ð□{Esž□ñ□ŸŒ,.%†ÞâÎîÒS□Ý□ÊâH$□□·¡ö5□‰üqo j□úMžŸu«ëw
æEah□à•7»-□sÜÐ□UEr:□‰üI}«Ge-x&ëIŠUb—+y□Ì`□œ6ÜmÏó®°€
+'ñ□Œî´ícû□Eðíö·©,bY□&□C□œã|-À'□□¡ðç□ŸTñ□ø{[Ðît=hEçGo4‹*Lƒ©I□†Çô>‡ □•□Q
@□□Q@□□Q@□□Q@□□Q@□□Q@□□Q@□º¿Ý‹ê□¥eÖ¦¯÷búŸéYt QE□ QE□ QE□ QE□ Võ□üyEôþµƒ
[Ö?ñå□ÓúÐ□Š(ªz¶§m¢é7Z•Ù□ªÛFd}ŠY^□€□M \¢¼âçâG^ím□T—
áÖ¨š:)'ç{¨Äêƒ«□~ðãžMvÚn·a«h0kv"n±š=\ƒ□¸ÉÈõ□ä{P□□□çgâ/^/ 7ú7ÃíV÷J□p¸–á
'EþòDAf□·-u~□ñ6Ÿâí□□_M/äÈJ´r

<n:«

ÄP□Í□W□âï‰z□†ã½ŠÆÒmbþÊ?2æ
c„·_YdÁ
ì9>Ô□ÛÑU´ë¿í□2Òô&Ï´B'ìÎvîPqŸÆ¬Ð□E□P□E□P□E□P□E□P□E□P□E□P□E□P□E□P□E
□P□E□P□E□P□E□P□Y:·úôÿ wúÖµdêßëÓýßë@□ôQE □QE □ÐZ□Ç¤_îŠçë ´ÿ •H¿Ý□□5□Q@□□
Q@□□Q@□□□åå¶Ÿg-
ÝåÄvöÑ.é%•,ª□RMqº_Ä¨5Ÿ□Ùh¶zMØ±¼,I ÔgýÚÌ□©D#%O÷Ž>†€;š*†³&©

•3èÖö×□ão—□Ì…□ò3’□=3\§ö•Äßú x{ÿ □äÿ âh□°¢¹□ x§SñU-¥>£cmmöKÇ´G¶”°JSï□$

ŒñžõÖM*A“JÛc•K1Æp É Ñ^v~"ø,ò£|>Õot 7
‰n $_ï$D`{z×Wáoiþ.ÐaÕôÒþL„«G
ÃÆãª°ìE lÑE QE QE QE QE QE QEƒ}ÿ -
²ýGòªõbûþ?eú•åUè ¢Š(¢Š(¢Š(--'ïËô›ZZOß—è(RŠ(Š+
Äþ'‹ÃV¶íö
ÝBîêO*ÚÒÎ=ï#c'Ø :“@´Wž‰ZŽ—¨YÃâÏ]èvw’ˆa½ûR\Fô´|™ÿ =u¾
Ÿ^,Æ6ðý•ÕÑK¹š5
ƒÈ -sŠ Ö¢¸9uo‰PDòË¡xu#E,Ì×ò rIùk_À^"½ñ_„mu«ë(í-áŸbFÄ†@Äçžq@
-OVÕl´=&çSÔfÚ[!’W=‡Ó¹= îMpð²|G5—ö-¯Ã½R]¯˜.ê5™£ë¸C,ÇŽzÐ¤QY°»aâ]

×WÓ%2Z\®ä$`,

□#±□□~•¥@□□Q@□□Q@□□Q@□□Q@□□Q@□□Q@□soþ±¾¦°Jæßýc}M 6Š(□Š(□Š(□Š(□¬Y□Çä
_Z¯V,¿ãò/- oQE□ Q\—ˆ<gw§k-
ØÚ/‡/µ½IcYdX™a†%9Æé[€N□□□†ü}&©â□ðî·¡\èzÏ“çÅ□Ò¬©2

¥□p

>•• ¡

ÒŠæ¼OâÖÐnml,´{í_T°Vx--T □□
;ž□dã5“¥üC»-"´ÐüOá«-□îû"ÍÞá.!™‡ðï\ ÞÞãÔd□»¢ŠÂñ?‰âðÕ-»},÷P»°“Ê¶´³•{ÈØÉö

¤Ð□í□çƒâV£¥ê□pø³Â□z□•ä¢□o~Ô—□‡=□í-
&□ÏC^‡@□□Q@□□Q@□□Q@□□Q@□□Q@□□Q@□□Q@□□Q@□□Q@□□Q@□□Q@□□Q@□□Q@□□Q@□□Q@□□Q@
□□Q@□□Q@□□Q@□□Q@□□Q@□□Q@□□Q@□□Q@□□Q@□□Q@□□Q@□□Q@□□Q@□□Q@□□Q@□□Q@□□Q@□□Q
@□□Q@□□Q@□□Q@□□Q@□□Q@□□Q@□□Q@□□Q@□□Q@□□Q@□□Q@□RÕt•?[±k-
NÒ;«Ves□ƒ*Jœ□ÔUÚ§ªj¶□.Ÿ-þ¥u□-¤Xß,§
2p?S@-S¯Å□´·„"Š5Ž4ÒYQ□`(□ã

Â½Š¼

]ñ§†î>?øo[‡X¶}2ßOh¦¹□ìF"~□ýô¿□{v‘-išý€¾Òoa¼¶,SÌ‰²7

£ë@□è¢Š (¢Š (¢Š (¢Š (¢Š (¢Š (¢Š (¢Š (¢Š (¢Š Žãþ=¥ÿ pÿ *çk¢¸ÿ •i□Ü?Ê¹Ú (
¢Š (¢Š (¢Š ÚÓ?ãÏþ□jåSÓ?ãÏþ□jå □QE □QE □ç>□aâ□‹°ÿ ˆí□të+%Ò□a÷g”8w*{í#□úU
_□øæÓV×gð…‡ˆ,txbùuMNâå"hÇx¡

Fd<,Ý□ë]_†µ_□ZZÙè--Ö4‰□$+□-µär;
' ’OROÔÐ□K^srÃÄ_□´Ód7Áá«I□ÜÃî‰f]«□}@ù¿>õ'Ž|}□†¨ž□Òõ;
□ZtÝ=õìË□V1Ÿâùˆß!□åQõ<Uÿ _ø#A°ƒFÒ|I¤ÜO,™fþÐŠI®¦cË6□,Ì□ □□Ù×ã½nò□-
<;¢>5ÍeŒ08ÿ —h‡úÉÏ QÓÜ□JÜÔ<G¤éZ¶□¥ÞÞ,7°‹2ÚÄU□˜W□ä

£®+‟Õ¼□â□Ÿ□Þø‟HñŸöd·0¤
□iqÜ□£P>Ugn□l±À□žø

ùtK?

üKø}¤Ø&Ëkk+ä\õc°e□¹9'Ü×¨W^k^□ñœ_□|/k?Žüëù »6÷¿Ù□¯ÙÀA¸l□□¸q"Ò½žÆ+^4ûhnî~
Õs□J²Üya<×□□¶Ž□''□¦h□Å□Q@□□Q@□□Q@□□Q@□□Q@□□Q@□TÔ¿ãÉ¾£ùÕ°©©□Ç"}Gó

J(¢€
(¢€
(¢€
(¢€54Ÿ¹/ÔV•fé?r_¨-* (¢Š)²H‘FÒHê^€³3□ □RM:¼‹Xñ–•ã½Z}□¼E§é~□µ“eì³^¤3j

:Å□$□□«□□AÆh□gá¢¶©-x³Å'¡K□bõ□Ï#-dp©O0□F$þUÎx□Å□Œ^5ñ¥Ö›ákýoS}Q-<ÄuŠ□¡„lT
2· ç' z□ôý□\ðÝê¦□¡j°]È·ˆm·²¹□ü¸Æ ùTœ□□?*àü?®iþ□ñ÷Š´Mzê;□u+ÓªY\Ü6È¥□□œn<
□□Ç>†€74_ˆ7□øŽ□□ø"Ã·Z

£t¬Ö¾dË<Sí□!d\□Øíþ#=Åy^±«Øxëâ?…ì¼=2_E£Ü5õõì□tQ□¸T

8%^íþ8ô□ñ-'ž$O□5â□UàûBÛíl˜òFsŒv<g4□©^srÃÄ_□´Ód7Áá«I□ÜÃî‰f]«□}@ù¿>õ'Ž|}□
†¨ž□Òõ;
□ZtÝ=õìË□V1Ÿâù^ß!□åQõ<Uÿ
_ø#A°ƒFÒ|I¤ÜO,™fþÐŠI®¦cË6□,Ì□ □□ÙÑE□ QE□ QE□ QE□ QE□ QE□ QE□□
›«ýØ¾§úV]jjÿ v/©þ•—
@□□Q@□□Q@□□Q@□□Q@□oXÿ Ç"_OëX5½cÿ -Q}?- X¢ŠÌñ□»iá-□}Zù'k[r¾o‟-öPX.ìz

äû□@-yâ¯ŠÓC¥ÝZÃá]vÁ¦□□Ûµk&ŠÚ,ñ¼•ÜH□qŽkµð.—g¢ø#HÓì/c½¶Š□¶æ2
ÊI,Xc¶Iª3üLð1Òd»“Äzl¶å h¼ÀÒ0ÇO+ï~□¬¿„°I£|;-îþ6±²’æâö□¦ãìöÄåsè0
~9

çRÔmt□6çP¾™aµ¶ŒÉ,□Đ(□Æü$°¹·ð•Î£u□·}gPŸSHX`Æ’□´~J□ã\×ü%:□Ä=YgÖuý*ÃÂösnƒO
¸½Ž9oäSÄ")l¬`ôC×©í^£¥ëš>²$þÉÕlo„8□}'á%Ùœã;IÆp□* Ð®+âM□-ŸÃ□□}šÞ(|ëy%"Ë@»
Ü•K□u'Ö°□ÄzN½-ôzeâÜ=„æÞä□aåÈ:Ž@ÏÔdW!ñGÄÚ□ð-
^´Á®i§Pû;Gö_µÇæîÈùvg9öÅ v-□ÿ '_Iÿ ¯(□ô□ZuÍx?Ä:&¥¢é¶V:ÆŸuw□"FH ¹G' U□*

F

Åt´ QE□ QE□ QE□ QE□ QE□ QE□ QE□ QE□ QE□ QE□ QE□ VN-þ½?Ýþµ-Y:·
úôÿ wúÐ□}□Q@□□Q@□t□Ÿñé□û¢¹úè-?ãÒ/÷E ME□P□E□P□E□P□7vv×öÍmyo□Ä

Ah¥@ÊpA□□Ž□□ð®□Z |qð¸□□iÛªîîï-
tûI.¯na¶¶Œeæ™Â"□RO□¼·Wñg‡%øÇáËèüA¥=œ:}ÊIp·'˜Ñ•@[8□úP□¬×)ñ□Ä□x□Â□□c-
Õ/]l¬□~óO'Ê¸÷□·á]□Ž¡e©Ú%ÞŸw□Ý³çdÖò
□°ppÀ□y□W□ŸøKþ0□□Kð¬_ð□¼"~»T~□{Ð□[á]□
ø_NÑ Á[XB³□ãsË7âÄŸÆ¶)□•□□ˆ
£$žÂ²tŸ□h°Þ„úÞŸ~"i±ï/9V@»>öC F(□î¥¨Úé:mÎ¡}2Ãkm□'Y□ P+•øIasoá+•Fê□núÎ¡>¦
•°Á□$#hü"-
Æ¹¯øJt□ˆz²Ï¬ëúU‡…ìæÝ□Ÿq{□rßÈ§‰&RÙXÁè‡¯SÚ½GK×4}dIý"ªØß□p$û%ÂK³9Æv"ŒàþT□¡
E□P□E□P□E□P□E□P□E□P□E□P□E□P□□÷ü~ËõÊ«Õ‹ïøý—
ê?•W □Š(□Š(□Š(□´´Ÿ¿/ÐVmii?~_ □J(¢€
(®cÆþ6°ðV"□ÅÎÙ.î_Ê´·i□bWÿ i•
£#,x□□□ñq†±¥iÞ□µ-f£¬ÝÆ□□&('ƒ¼§Ð

~¦½□¼×Âz□…ôË›□s\ñž□{â+å□y×P‹d ÔC□-Â□ÌžMz4□Ãuo□Å¼©,2¨xä□ƒ+©□□□Á□w

#â}õÄú]□…4ù
ê-!¸û eë□□™Ÿè□□ø□vv6VúnŸmck□ŽÞÞ%Š$Âª0□ä+,ð‘ÿ „³â-·âÆù¬tüé:aìvœÍ
ú· úqŰ»ÍBþ×JÓ®/ïe□ÚÛFÒË!□íP2N□'ð

/ã%•Õ÷Ã[ñi
Na’)å…FKÆŽ

{

□
¿kñ;Á7□\wËâM:(Ù□yRL«*û□þö□
Ö>)Ñ□ÃIâ6¿EÒ□□ÅÓ+□ÚÌ□□1‘É□‘År□¯‰¾□é¨Úœqø~þýŽbŽÆ□¦¸•Ï@6,A'¹Å CðbòÚïMñ0°b
Úrë·□hv•ÄL□” y□v÷¯M®GáÎ‹{¤øj[•R!

§ªÞK¨ÝD?å›Êr□ðP£ëšë¨ ¢Š(¢Š(¢Š(¢Š(¢Š(¢Š(®mÿ Ö7Ô×I\Ûÿ ¬o© □ÑE□ QE□
QE□ QE□ U‹/øü‹ëUêÅ—ü~Eõ □ê(¢€0|Mâ□øF¢·"û□ZÕ|âÃn—iç˜ñŽ_'€sÇÐ×
á□n□□üQ□V»□KŸHµ'□]*ä2]9□½#, Æ8Â–
ÇREv□?□ü3§øŠëBÔõ□Ó¯möœÞ□*9□"0dsò'Î9#□k□ñ□¯¥xÃâW„ ðÌñ_^i·-sy{jw$6øå
Ž

î˜Ïó □X¯9ñ»□□Çþ□ðõ˜ó'°½]^ñ×¤□G÷C–Û‰Æ>•£ãÏ–ÛøT[i–òÚ□m_Œ[‹¹„PÂ½

²¹ □□<ub0*Ÿ„µ□□ønÖg"ÆZ-ö-zþmõôš„;ç"þúáGEQÀ□□è4V^·â='Ã'ZË«^-
²]N¶ð'¬Û¤nƒ€qÓ©â³<oãk□□i1Ü\í'îåü«Kv•F%□ò˜ðª22Ç□@□-
□□k□V□àûQæj:ÍÜaPrb‰□;Ê} ÇêkÑ«Í|'¨ø_L¹¸×5Ï□è□¾"¾P'•u□¶@•D1□Ü
üÉä×£A<7VñÜ[Ê'Ã*‡ŽHØ2°'•A□□Gz 'Š(□Š(□Š(□Š(□Š(□Š(□Š(□Š(□Š(□Š(□
Š(□Š(□Š(□Š(□Š(□Š(□Š(□Š(□Š(□Š(□Š(□Š(□Š(□Š(□Š(□Š(□Š(□Š(
□Š(□Š(□Š(□Š(□Š(□Š(□Š(□Š(□Š(□Š(□Š(□Š(□Š(□Š(□Š(□Š(□Š(□©ê
šU†·§Éa©ÚÅui.7Å È89¨«"P□!ÿ
³Àßô,Ø□ß'ük ÑôM3@°□:M"6vÁ‹ùq.□ãÔýjý□ QE□ QE□ QE□ QE□ QE□ QE□ QE□ QE□ QE
□ QE□□□Çü{KþáþUÎ×JÊ□
·B0j¯öu·÷□ýôh□□ŠÛþÎ¶þáÿ ¾•ÙÖßÜ?÷Ñ

J+oû:Ûû‡þú4□g[□þÿ ßF€1(-¿ìëoîûèÑý•mýÃÿ }□ M3þ<ÿ àF®S"…
M‘Œ.sÖŸ@□□Q@□□Q@□—>□ð½åÌ—7^□Ñç¸•‹É,¶13;-¤’¹&Ÿcá?

i—iw§ø□J´¹LìšÞÎ8Ýr0pÀ□8$VÅ□□‘□á_

ê—mw¨h□]åË □k‹8är ÀË□OJŠßÁž□´¹ŠâÛÃZ43ÄÁã–;□•‘□È …È
÷-Ê(□´úu•ÕÕ½ÕÅ□¼×□Ä˜&'%g^ž»I□\ûUš(□$±´šò□Ém`{¨□,34`¼a¾ðVê3ß□jz(□Š(□Š
(□Š(□Š(□Š(□Š(□ªj_ñäßQüêÝ2X'hÊ8Êš ç(-¿ìëoî-
ûèÑý•mýÃÿ }□ Ä¢¶ÿ ³-¿¸□ì£Göu·÷□ýôh□□ŠÛþÎ¶þáÿ ¾•ÙÖßÜ?÷Ñ

J+oû:Ûû‡þú4□g[□þÿ ßF€ Ò~ä¿QZU□6ñÛ,#□Ï^sRÐ□E□P□X□ào□»□o
è…‰É'O‹$ÿ ß5¿E eé¾□Ð´k†¸Òô]:Æf]•%-ªDÅr

P

2-ÕcPÒtÝ^

¥§ÚÞÄ§!.aY□£□W(
Ö:uŽ™n-ôû;{H□Ï—o□Æ¹ú □□N²:^ÔM□¹¾Xü¡rb_0&s·v3Œö«4P□Eÿ …|;ª]µÞ¡ iw—
, i®,ã'È□□,A=*+□□xZÒæ+‹o□hÐÏ□□ŽXìbVF□ ,□
ƒÞ·(□Š(□Š(□Š(□Š(□Š(□Š(□Š(

Ý_îÅõ?Ò²ë¡šÞ;€<Áœtç□□öu·÷□ýôh□□ŠÛþÎ¶þáÿ ¾•ÙÖßÜ?÷Ñ

J+oû:Ûû‡þú4□g[□þÿ ßF€1(-¿ìëoî-
ûèÑý•mýÃÿ }□ Ä¢¶ÿ ³-¿¸□ï£Göu·÷□ýôh□□·¬□åÊ/§õ¦□g[□þÿ ßF¬Ç□Å□D□QÒ€□A Œ-E□
P□BxWÃ±Þ
ÄÐtµ°□wœ,ã□Ÿ]ØÍjM

w□É

Ñ¤'H¥□□AVSÁ□- Óè

□þ□O□ÿ Ð©¡ÿ à°þ&´4Í
HÑ□£JÒ¬l<ÜyŸe·H·ã8ÎÐ3ŒŸÎ´(
Öšu•ƒNÖvvöíq!-c

J†G=Y°9>æ³n¼□á{Û©.nü7£Ïq+□’YlbgsêI\“[tP□^•á•□G¹7:f‰¦ØÎT¡–ÚÒ8˜©ç□P

8□{V¥□P□E□P□E□P□E□P□E□P□E□P□E□P□E□P□E□P□E□P□E□P□E□P□Y:·úôÿ wúÖµC5¬3°i□'
□:Ð□?Emÿ g[□þÿ ßF•ìëoî-
ûèÐ□%□·ý•mýÃÿ }□?³-¿¸□ï£@□•ÐZ□Ç¤_îŠ‹û:Ûû‡þú5e□c@Š0 `P□¨¢Š (¢Š (¢Š ,îÎ×P
´'ÒöÚ□›i□-□•:0÷□ƒXßð,x?þ…M□ÿ □Ðÿ ñ5ÐQ@□ìtû-.Ñ-4ûK{KdÎØmã□¢äää@ rI¤´Óì¬

æÎÒ
□΄JÓMäÆ□Ì•õvÇV>§š³E □□0y□NßJÓ¬ôöÓí´ûX,˜0kx¡UŒ†ûÀ¨□ç'>µrŠ çÿ á□ð□ý
šþ
¡ÿ âkCLÐ´□□J4-*ÆÃÍÇ™ö[t‹~3Œí□8ÉüëBŠ (¢Š (¢Š (¢Š (¢Š (¢Š (¢Š (¢Š Á¾ÿ •Ù~
£ùUzÞ’Æ d.êK-¼šgöu·÷□ýôh□□ŠÛþÎ¶þáÿ ¾•ÙÖßÜ?÷Ñ

J+oû:Ûû‡þú4□g[□þÿ ßF€1(-¿ìëoîûèÑý•mýÃÿ }□ Ä--
'ïËô□gû:Ûû‡þú5,6Ñ['cR3×œÐ□ÔQE □CSÐô□ic]WK²¿X‰1‹«t"!=q¸□t□~Š çÿ á□ð□ý
šþ
¡ÿ âkr□!¶·ŽÞ□'(#@'Ç□…TP0 □€ íRQ@□¬tû-.Í-4ûH--";!,0^¹98□Ž¤š-
x!°·'Þâ$š□T¤'È¡•ÔðA□,*J(□¼v□qX-Œv□%š§--Ö0#
ýÝ½1íTì|5 é—
?h°Ñ4ÛIÏüµ·µHÛó□5©E □QE □QE □QE □QE □QE □QE □QE □Í¿úÆúšé*©Ó-‰$¡Éÿ hÐ□□□
·ý•mýÃÿ }□?³-¿¸□ï£@□"Vßöu·÷□ýôhþÎ¶þáÿ ¾• bQ[□ÛÖßÜ?÷Ñ£û:Ûû‡þú4□‰Emÿ g[□þ
ÿ ßF•ìëoîûèÐ□%X²ÿ •È¾µ§ý•mýÃÿ }□tv0DáÕHaÓ"@□h¢Š §¨i□f¯□Ç©iÖ—
¨½□æ□•□Á•¥°Ó4ý*□□•cmg 91ÛÂ±®~Š□[¢€35/

hzÌÉ6©£i×Ò¢ìWºµIYW®□`p9ªCÀ¾□□#Âº-□i□□ÿ □]□□□ZóN²ÔV%¾³·¹X¤□Æ'‰\#ŽŒ28#Ö¡Ô
ô='ZX×UÒì¯Ö"LbêÝ%□O\n□□□_¢€9ÿ øA<ÿ B¦‡ÿ ,è□øšÜ,□m-ã··‰"†%
□q¨UE□ 8

Õ%□ QE□ QE□ QE□ QE□ QE□ QE□ QE□ QE□ QE□ QE□ QE□ QE□ QE□ QE□ QE□ QE□ QE□
 QE□ QE□ QE□ QE□ QE□ QE□ QE□ QE□ QE□ QE□ QE□ QE□ QE□ QE□ QE□ QE□ QE□ QE
□ QE□ QE□ QE□ QE□ QE□ QE□ QE□ QE□ QE□ QE□ QE□ QE□ QE□ QE□ QE□ QE□ QE□ Q
E□ QE□ QE□ QE□ QE□ QL™ŠA#/P¤•Ê±¿´n□ç ÿ ¾E nQX□Ú7?óÐ□ß"§´½ž[¤GpTç#□Ò€5h¢
Š (¢Š (¢Š (¢Š (¢Š (¢Š (¢Š (¢Š (¢Š (¢Š (¢Š (¢Š (¢Š (¢Š (¢Š (¢«ÞÊðÛ3¡Ã□;P
□Š+□ûFçþz□ûäQý£sÿ =□ýò(□rŠÊ´½ž[¤GpTõàzV- □QE □QE □QE □QE □QE □QE □QE □Q
E □QE □QE □QE □QE □QE □QE □QE □U-Bâ[q-ØÎsÆj‡ö•Ïüô-
÷È □Ê+□ûFçþz□ûäQý£sÿ =□ýò(□rŠÃþÑ¹ÿ žƒþù□-½õÄ—□£8*X□À □z(¢€
(¢€
(¢€
(¢€
(¢€
(¢€
(¢€
(¢€
(¢€
(¢€
(¢€
(¢€
(¢€
(¢€
(¢€
(¢€
(¢€
(¢€
(¢€
(¬ýBêh%U•°
ç¦h□BŠÃþÑ¹ÿ žƒþù□¯ní%¼nÇ,W&€%¢Š(¢Š(¢Š(¢Š(¢Š(¢Š(¢Š(¢Š(¢Š(¢Š(¢Š
(¢Š(¢Š(¢Š(¢Š(¢Š(¢²nïgŠéÑ□□□Ž□Cý£sÿ =□ýò(□rŠÃþÑ¹ÿ žƒþù□□hÜÿ ÏAÿ |Š
 Ü¢°ÿ ´n□ç ÿ ¾E-
Ú7?óÐ□ß"€7(¬í>êiæe‘²□ç¥hÐ□E□P□E□P□E□P□E□P□E□P□E□P□E□P□E□P□E□P□E□P□E□P□E
□P□E□P□E□Šú…Èv□Æ□þè □ª+□ûFçþz□ûäQý£sÿ =□ýò(□rŠd,^□Ù°•□þTú (¢Š (¢Š (¢Š (
¢Š (¢Š (¢Š (¢Š (¢Š (¢Š (¢Š (¢Š (¢Š (¢Š (¢Š (¢Š (¢Š (¢Š (¢Š (¢Š (¢Š (¢Š
(¢Š (¢Š (¢Š (¢Š (¢Š (¢Š (¢Š (¢Š (¢Š (¢Š (¢Š (¢Š (¢Š (¢Š (¢Š (¢Š (¢Š (¢Š
 (¢Š (¢Š (¢Š (¢Š (¢Š (¢Š (¢Š (¢Š (¢Š (¢Š (¢Š (¢Š (¢Š (¢Š (¢¹¿□Yx›QÐ□ÓÂ·
°Y^Ë:¬·□œ□áÁÜWƒógoáœs@□%□à¾+ð□‰|□áÛ□□ØxïT¹½³Û$ë1!e□€îÇ=z6s^Ãá-
fO□øGJÕå@’ÝÛ$’*Œ Øç-ÙÎ(□fŠ(□Š(□Š(□Š(□Š(□Š(□Š(□Š(□Š(□Š(□î?ãÚ_÷
□ò®v°+•øö—
ýÃü«• □¬Ø□Çô□•ò5Z¬Ø□Çô□□ò4□»E□P□E□P□E□P□E□P□E□P□E□æÚõÆ±â□‰2øJÛ\¹Ñ4û;□»•
ìð·□,Í□•ÏÝQíßô ôš+Êæ¶Öü□ã

[[ø›RÖtíbèÚÍg©È&•8Ï˜¯€p½ÿ úüz¥ □W□ãm{U·½Òü7áÖŠ=gUgÅÄ«¹maA—
″Žç• <□T¢øsªC$wKñ□ÄÍz¤3□'V□□ýrÆ1íš ïè¢Š (¢Š (¢Š (¢Š (¢Š (¢Š (¢Š *¦¥ÿ -M
õÎ-ÕMKþ<›ê?• bQE□□fÃþ?búŸå[µ…aÿ ±}Oò-Ú (¢Š (¢Š (¢Š (¢Š (¢£žd¶·’yN#•
±ô dÐ□″W″xwKñ□Ä□/þ□]KÅ:¾‘ivîll4™„>TAŠ,í,Xœgüàkø+SÖtÏ□êž
×¯ÛQ’ÚÝ/l/dP$-□;H□R-□žüûP□ QEqZ¯„|Aâ□bê[ß□_éÚ`lZZèì!p¸-
4’□I$çŽ″□ÚÑ^uàûÝoEñö¥à½WU—W·K□Ô,®ç□ÎT.□£Ÿâ9=}½ð=□€
(¢€
(¢€
(¢€
(¢€
(¢€
(¢€
(¢€3u□»□Ôÿ JË-M_îÅõ?Ò²è ¢Š(©-?ãî/÷…CSZ□ÇÜ_ï
 è(¢Š (¢Š (¢Š (¢Š (¢°üG7ˆB[ÚøzÖØÍpXKytÿ »µQ□›`åØç€8ã“@□″W□ü5›T]wÆ□~§¬]ê
mg}□i-ÃtÊdíQÂ

ö□VçŽ|Iwáý&Ú-*□çÖu+•³±ŽO»æ7VoöT□Oá@□E□ç©ðãZž1q□ħ
Ä‡Q#,Ö³,Vá½¢Æ1ø×yk□°ÙÁ□ó™åHÕ^b¡LŒ□
`p2yÅ ME□P□E□P□E□P□E□P□E□P□E□P□E□P□E□P□E□P□E□P□E□P□E□P□Y:·úôÿ wúÖµdêßëÓ
ýßë@□õÐZ□Ç¤_îŠçë ´ÿ •H¿Ý□□5□Q@□□Q@□□Q@□□Q@□□Q@□□V-
•.g²ð/ˆ.-¥xn!Ó®$ŽD8da□□AìA □Ê+Ëô□‡Rê¾□Ó5□üwãešêÒ)ÝSW!C2□8Êž2kÑ4-?û+K·±û
]Ýç’›~Ñy/™,žìØ□4□rŠdÞo‘''³ÍÚvoÎÝØã8íšóãðûÄz„□ëSø□-Gª0Ü□žÂ□XÏ§—•˜□r
□D¢¸Ï†~ Ôõï□Ý&²É&£¦_Í§Ï<c
3G□œ□pÃò®Î€
(¢€
(¢€
(¢€
(¢€
(¢€
(¢€
(¢€0o¿ãö_¨þU^¬_□Çì¿Qüª½ □QE □QE _Ò□åáÿ Üþ¢µë#Iÿ •‡ÿ súŠ× □Š(□Š(□Š+/_¹
Ö-´áý…a□åô’□ÔO/—□@ç2?r□:

Nh□RŠóO□¾»iñWYÓ5□vãRÛ¦C9R6BŽÍÏ—□á@□g©îkkâ'ˆµ-□MÓ¬tC□êú½êXÛK
ÊÃ»¬„wÀþ}úP□cEy–£à□i°d°–"ãÍzçY•

¢+©CÛLÀgo•Œ(=:œWaàß□¯Šü!¦ka□5Ô9‘□¢¸%\□mÀÐ□í□□Ô²Ág<°@×□¤lÉ
°S#□• ž□O□5åzÌþ0¶ñ_„/5}Wì‹□Ⓒˆ N•bäC□{IÄ□ÖVéŸá□… zÕ□Q@□□Q@□□Q@□□Q@□□Q@□□Q
@□□Q@□soþ±¾¦°Jæßýc}M 6Š(

Šßþ=¢ÿ p□*’£·ÿ •h¿ÜÊ¤ □Š(□Š(□Š(□Š(□Š+Ï<M¨k^ ñä^

Ñu7Ò-á³ûn¡{
ƒ)RÛV4'îžù÷öÁ ô:+ÉõÛMoá•³ñ□-'Õµ}□\Ç

¥kªÊ&e•Îß1□□□□8÷□ë□ QX^'ÓµíRÖÞÓDÕãÒƒÉþ•såy’ˆñÒ0x□žç§jóÿ □éÚïÃM>/□Øx³XÕ-
`ž5¾±Õf□,±³□;

□Ó’:~|`€zí□Q@□□Q@□□Q@□□Q@□□Q@□□Q@□□Q@□□Q@□□Q@□□Q@□□Q@□□Q@□□Q@□□Q@□□Q@□□
Q@□□Q@□□Q@□□Q@□□Q@□□Q@□□Q@□□Q@□□Q@□□Q@□□Q@□□Q@□□Q@□□Q@□□Q@□□Q@□□Q@□□Q@□
□Q@□□Q@□□Q@□□Q@□□Q@□□Q@□□Q@□□Q@□□Q@□□Q@□□Q@□□Q@□□Q@□□W□ñJãÅqxSÉð•¬³^ÜIå
K$8ó"Œƒ’¼ðO□=³ë, 9O‰ZÜþ8Ö"øqá¦□É$□õ[¥□Ž□R

Ò}Ž ÷□z′□«iZm¾•¤Yé– ˆ-
!HcÏ\(À'ßŠð□□ÜøïÀúI³Ó¾□3Í!Ýqu$‡Ì™½Ï ì;}I'Ú<'©ë□¶•□Þ»¤ÿ eß30kmû°□àûf€6è¢
Š (¢Š (¢Š (¢Š (¢Š (¢Š (¢Š (¢Š (¢Š Žãþ=¥ÿ pÿ *çk¢¸ÿ •i□Ü?Ê¹Ú *Í‡ü□Gø
ÿ #UªÍ‡ü□Gøÿ #@□´QE □QE □QE □QE □QE □åÞ6¶ÿ „ÃÅ‰¤ørÝ¢×ô•F›[YŒBÅ_'-
ËÌ…†~^ƒ=z×¨×œ^èž–ð¯ŒµmwÃ66šÅŽ®c{› ®□□Ç"

e□ñƒÏ_^œP□FŸ□£àO□A}ãP5ƒ¨J--<@□ÿ £□é□DxŒ7?2þ'-zõy•þ—ãOÜéöšî□iáý□Úé.§Œ^-
Ìó”9□
• zÿ ‘]Ž¯uâ8|A£C¥iö³é2³•Fy_□
€6í□†sÏcøu

□Å:~§«|r²²Óõ™4’|>I¸Ž%w+ç¶åMÜ)?/ÍØ
<SáÍ□ÀÚ□Ç‰to□ë—²ØâY-5YÄðÌ™□€□□x=¿NµÑøßÃzÍÖ¯¤øŸÃ-nu□/z□{†Ú—
0¸ù□žÇÓëì+□V²ñç□ìÆ‰ªh¶¾□Ò%e7Ò‹å¹šd□□‘í□\‘ßÿ ¬@=□N¼]GL´¾E*—0¤Ê§¨

ãõ«4ÈaŽÞ□à…□E□„E□ □ Sè ¢Š(¢Š(¢Š(¢Š(¢Š(¢Š(ªš—
üy7Ô□·U5/øòo¨þt□‰E□P□›□øý‹ê□•nÖ□‡ü~Åõ?Ê·h ¢Š(¢Š(¢Š(¢Š(¦M
\A$2□ÑÈ¥□z,0iôP□•è□Ÿ□<□§ÿ Â?cáëO□ip;ýŽåoÒÚEFbØpã'
=?:‹Ãíâ□ßŽ_i×í¬mn#Ð□î,ä2□£3|ªìz¶w-8Æ+eµÿ ‰'-¶ÿ „'N-
\•/□UU‡ë°•øö-_□x^÷G"PÕõ»¸ï5íMÕ®¥‰HŽ5Q„Š0yÚ?3Þ€:°£¬ë□□□"q©êw
□¤
¹Ý¿@□rz□Þ¨i×^#"ÅZ-½þŸk□‡□Æl.Qó$¬@Ý¸n8ÁÏaø×Ÿë¶Þ9¾ñä—÷~
þÙÒl%?ÙvãU,□•òÙÕ²YÏlão¦y

•À°Uýæ«©øÓY□-¯u`±ÚÚ?Þ¶µ_¸-þÓ}â>žâ»ªä4?□øÊÿ W†ÛVð'ö]“îó.ÿ µáŸËÂ’>E□98-
Žk¯ □Š(□Š(□Š(□Š(□Š(□Š(□Š(

Ý_îÅõ?Ò²ëSWû±}Oô¬° (¢Š *kOøû‹ýáPÔÖŸñ÷□ûÂ€:
(¢€
(¢€
(¢€
(¢€
(¬/□j°þ—□»h>□þÛwb%O·Gmå□Œ□¸9Ï==(□›ð□üŽþ?ÿ °”□ı.³þ'[ê□ž<ð□¦□¨□gÍ,·•n¼¡'-
ÞZò□ðN7□žæ¨x~o^z-¹¯ê_ð¯<ÿ í{•¸òÿ ¶-×ÊÂíÆyÝõÀ®ËÇž□¿ñ-™au¤Í□¶·¥Ü-å›KÊ□□Q±
Ø□ä(□□Xð□½¥éWZ¦•ãß□>§m□L©w:Éo!Q□¦<`□ŒwÅv-

×_ÄÞ□Òµ™cXå°€4Š½□Ž□-Ù□□È_Ü|Lñ6™.ŠÞ□°Ð–å

7□"ß¬à!□c□kÈ$g□?—Zï4--ÛÃú□Ž‘iŸ"Ò□‰
ul

§Üž□□ Ð¢Š(¢Š(¢Š(¢Š(¢Š(¢Š(¢Š(¢Š(¢Š(¢Š(¢Š(¢Š(¬•[ýz□»ýkZ²uoõéþ
ïõ

úè-
?ãÒ/÷EsõÐZ□Ç¤_îŠ šŠ(□Š(□Š(□Š(□Š(□©k□d:Ö‹}¥Ü3¤7¶ò[ÈÑ□□+©RF{óWk3_“Y‡
G-M□
Iõ□*R+¦*Œ27

Ž‡□Ç½ qkð²öÂÍ□Gñ÷Š–æ…□Â·□Bh□□ yx□□8-□‡-$¿ñ'†æmY#□žŸy-
…ÛF0¯$xË□î□üsY’kÿ □o#6¶þ ˚Óça´^\ê©,H□⅟±Fã[þ

ðÇü">□□xŸíwŽïqspÃo□;œ³{

ƒè(□£®kÆ^*-□Óã,Î/µëwÄÃ§Y/-,ŸÞ>ˆ½IéŠvƒ}â©¼'-Ö¹¤ÚÁ®¨”Çg□£c‘□ƒvæ□<wü«Ï|<>
éZ□Þµ©|=:¦¹u•{ÇÖ-ÐE-xŠ$çb•©$òM z□□ü0|'áx4éfóï□šâò□ùé3œ±úv-ÀWGX-□Õ¼Cª
¯íï

□a˜öù#íñÜùÙÎ~à□q□×®}«~€
(¢€
(¢€
(¢€
(¢€
(¢€
(¢€
(¢€0o¿ãö_¨þU^¬_□Çì¿Qüª½ □QE □QE _Ò□ãáÿ Üþ¢µë#Iÿ •‡ÿ súŠ× □Š(□Š(□Š++Ä7
ú¶□¦yú.‹ý±w¼/Ù¾Ô–ÿ /9mÍÇ□qï@□ž–ÿ %ß^ÿ °=¿þ‡Z□□<–
}âM2Æ}"â(5}.ñ/lÚ`v3/ð·±þ•ÆÚÍñ

ÛÇwþ&ÿ …y»ívqÚý›ûjÜlÚs»w|úb½□S¿ñ:ørÒ÷KÑm_Um□s§Ü]□°□;‛d)`qÏB3@□èñ□ÄÉâò?á

Ó,e□æ½¹Õ□á--±□ì{f•ðR□!øU¥□□<Ç•Ôc)•ñþ?•V¾□<cjúMÆ•iá{ Ç—
uroVêvŒðË□A€Hã'Ö»),æÐ¼&ö^□´ŠIìí

v6ò6□™W□□äu dä}h□^¼ûâ/ü•>□ÿ °ÇþÈk«Ó.u-ðÄ7:žŸ
ë?g/-œ2 †\•1$

œ

äã=My¯ˆ¦ø‡¯ê°□ïü+Ï#û&óí[?¶-ÛÍã□sÆß¯4□ì□V7†õ-kS°–ms@þÅ¸YJ¤-
lK□é€wnA□É#-Õ³@□□Q@□□Q@□□Q@□□Q@□□Q@□□Q@□soþ±¾¦°Jæßýc}M 6Š(

Šßþ=¢ÿ p□*’£·ÿ •h¿ÜÊ¤ □Š(□Š(□Š(□Š(□¼ÿ Äþ-
ñ-Ÿãh|cáXm¯g{O±Þé÷□y~r□Ü□□ð□§_Nù5è□Ìx“Sñ~›□□h^□µÖlZ/•□è··dÉÏ-
ò•Æ=óš óŸ‰°¯Žu_‡Ú”Z—†l4k□1,,Kñs4¤Ê›V0€ wc¯`kÚmÑ¢¶Š69e@¤û•\□:-
Š¼c¬Ø^x²ÚÓKÒtù…Ì:T□yÏ4Ë÷ZW.Õê ëÞ°o□ÝxŽÖ
□á½>ÖöW»D°□□´G□Îæ0äqëô4□»^o¯?ü,_□[øvÄïÐt›•ŸV°rYW•·SÜç–
ôã¿]/‰CÅóèöö^□°{□´¹[Ù"°Ž
c‹Ž□œà□É□°qŽ•□£jž4Ð4¨4Í3á:[Ú@¸D]~ßñ$ã’z’zÐ□§QPXËq=…´×vße¹’%im
üÀþS•]Ã†ÁÈÈëŠž€
(¢€
(¢€
(¢€
(¢€
(¢€
(¢€
(¢€
(¢€
(¢€
(¢€
(¢€
(¢€
(¢€
(¢€
(¢€
(¢€
(¢€
(¢€
(¢€
(¢€
(¢€
(¢€
(¢€
(¢€
(¢€
(¢€
(¢€
(¢€
(¢€
(¢€
(¢€
(¢€
(¢€
(¢€
(¢€
(¢€
(¢€
(¢€
(¢€
(¢€
(¢€
(¢€
(¢€
(¢€
(¢€
(¢€
(¢€
(¢€
(¢€
(¢€

(¢€
(¢€
(¢€
(¢€
(¢€
(¢€
(¢€#œ□·□□’P€?
Ãû%Çüñ□Ê°
(□Ÿû%Çüñ□Ê¬Y[Ì—
q³DÁFrHö–Š(¢Š(¢Š(¢Š(¢Š(¢Š(¢Š(¢Š(¢Š(¢Š(¢Š(¢Š(¢Š(¢Š(¢Š(¢Š(¢Š(
ª×èÒZ2¢–9□
³E sÿ d¸ÿ ž/ùQöK•ùâÿ •t□P□5•¼Éw□4L□-I-Õ³E□ QE□ QE□ QE□ QE□ QE□ QE□ QE□
QE□ QE□ QE□ QE□ QE□ QE□ QE□ QE□□CSŠIV?-
`œàVwÙ.?ç‹þUÐQ@□ÿ Ù.?ç‹þT}’ãþx¿å]□□□Ïý’ãþx¿åRÛ[N·1³Dà□□$VÝ□ QE□ QE□ QE□
QE□ QE□ QE□ QE□ QE□ QE□ QE□ QE□ QE□ QE□ QE□ QE□ QE□ QE□ QE□ QE□ Vf¥

²Ì…#f□{
Ó¢€9ÿ ²\□Ï□ü«nÙJÛF¬□!FA©h □Š(□Š(□Š(□Š(□Š(□Š(□Š(□Š(□Š(□Š(□Š(□
Š(□Š(□Š(□Š(□Š(

kËyžîFX˜,x {Td¸ÿ ž/ùWAE sÿ d¸ÿ ž/ùQöK•ùâÿ •t□P□?öK•ùâÿ •-
d¸ÿ ž/ùWAE fi°Ë□î^6PW□#Þ´è¢€
(¢€
(¢€
(¢€
(¢€
(¢€
(¢€
(¢€
(¢€
(¢€
(¢€
(¢€
(¢€
(¢€
Á{YË±□¿SÚ·¨

□i——
óÅÿ *>Éqÿ <_ò®,Š Ž VÞ0F□P□ü*J(□Š(□Š(□Š(□Š(□Š(□Š(□Š(□Š(□Š(□Š(
□Š(□Š(□Š(□Š(□Š(□Š(□Š(□Š(□Š(□Š(□Š(□Š(□Š(□Š(□Š(□Š(□Š(□Š(
 □Š(□Š(□Š(□Š(□Š(□Š(□Š(□Š(□Š(□Š(□Š(□Š(□Š(□Š(□Š(□Š(□Š(□Š
(□Š(□Š(□Š(□Š(□Š(□Š(□Š(□Š(□Š(□Š(□Š(□Š(□Š(□Š(□Š(□Š(□Š(□
Š(□Š(□Š(□Š(□Š(□Š(□Š(□Š(□Š(□Š(□Š(□Š(□Š(□Š(□Š(□Š(□Š(□Š(
□Š(□šî±®ç`£ÔÓª¦¥ÿ -MõÎ€-++¨e
ƒÐŠZ¯cÿ -Q}?-X □Š(□Š(□Š(□Š(□Š(□Š(□Š(□Š(□Š(□Š(□Š(□Š(□Š(□Š(
□Š(□Š(□I,qcÌp¹é“M[˜],¬ªXô Õ-
_îÅõ?ÒªX□Çì_Sü¨□vŠ(□Š(□Š(□Š(□Š(□Š(□Š(□Š(□Š(□Š(□Š(□Š(□Š(□Š(
 □Š(□Š(□Š(□Š(□Š(□Š(□Š(□£’x¢
<Š¤úš’²uoõéþïõ □□µÛÿ ÏdüéRâ□□jH¬Ç°5ÏU-;þ?Sè□• nQE□ QE□ QE□ QE□ QE□ QE□
QE□ QE□ QE□ QE□ QE□ QE□ QE□ QE□ QE□ QE□□□\ÂŒU¥PÃ¨&•'ŠFÚ’+-
@k□ûþ?eú•åRé□ñ÷ÿ □4□³E□P□E□P□E□P□E□P□E□P□E□P□E□P□E□P□E□P□E□P□E□P□E□
P□E□P□E□P□E□P□P›»ppfOÎ¦®mÿ Ö7ÔÐ□ð°€□□©“ïSW9□úäÿ xWG@□□Q@□□Q@□□Q@□□Q@□□Q
@□□Q@□□Q@□□Q@□□Q@□□Q@□□Q@□□Q@□□Q@□□Q@□□Q@□□Q@□□Q@□□Q@□□Q@□□Q@□□Q@□□Q@□□
Q@□□Q@□□Q@□□Q@□□Q@□□Q@□□Q@□□Q@□□Q@□□Q@□□Q@□□Q@□□Q@□□Q@□□Q@□□Q@□□Q@□□Q@□
□Q@□□Q@□□Q@□□Q@□□Q@□□Q@□□Q@□□Q@□□Q@□□Q@□□Q@□□Q@□□Q@□□Q@□cøŸÅ□W„tgÕuyÌVê
Á□*îi□ç
£¹8?•lV?‰£Ñ□‡e½×-!¹±°ÿ K"X¼À¥□ù,÷8'ó □;ƒö•Ð□Ì_lÑõ{;)[□ÝI□*G© ô-Ù¯X·¸†êÚ
+‹y□Xe@ñÈ‡!”Œ,□¡□à_□>-øwÅþ□“ÃZ%¼Ó\ÞÉ□,×*°Ç

□□å•□Œdà I&½¯ÂÚcè¾□ÒtÉ%Ydµ´Ž&u9V!@8öôö □j(¢€
(¢€
(¢€
(¢€
(¢€
(¢€
(¢€
(¢€
(¢€
(¢€
(¢€
(¢€
(¢€
(¢€
(¢€
(¢€
(¢€
(¢€□,±Á
Í+¬q¢-wc€ rI=…p□|bÐq$öšV¿}§ÆÄ>£k§³[Œu;‰□□¥?ã%ÌÑü>-Î□hÛQº,Ì²õ
î7~`□ø×qego§ØÁei□Åm□b8ãQ€ª□ □4}cO×ô¨5=.å.lç\¤‹ß±□-A□,□^¯8øk□é~)ñÎƒ Ùii
©%Ä1Ž^&BÄ□AòŽ+Ñè ¢Š(¢Š(¢Š(¢Š(¢Š(¢Š(¢Š(ªš—üy7Ô□·U5/øòo¨þt û-
øò‹éýjÅW±ÿ •(¾ŸÖ¬P□E□P□E□P□E□P□E□P□E□P□E□P□E□P□3âÏ-è^

€¶£<’\l2-¥ªù“□□[nx_v {Öý•ÔwÖ6÷‘□□Ï□Ê□†

dgó®â

•¥é_□<]yeg□wW°<·3òÏ#□:±ç□°è;
ë|9ÿ "¾"ÿ ^Pÿ è□€4ë^Õ¾&Yéž!¾Ñ`ðï^õK›,\>›b&D.¡"□¸□Áî;□íëÊ□Ä□-□ø—
ã□çðÏ^µ□/ÞÍ¡›N°3&□ □–•:œqž†€;?□x¿þ□K™áÿ „wÄ□W"•·ê–^B¾N0§qÉ®'¹¯
øïCñl³ÛXI47Öã3Ù]Äbš1êTöätÏQ]-
□QE □QE □QE □QE □QE □QE □QE fêÿ v/©þ•RÃþ?búŸåVõ□»□Ôÿ J©aÿ ±}Oò □Ú(¢€
(¢€
(¢€
(¢€
(¢€
(¢€□î±£;°TPK18 z×□7Æ□□y'Ùizö¥c□!ïì´ö{uÇ_˜'ÀúW~è²##¨d`C+

,=□p:¿Å¿□xfK□(]‟wdÆ□±AlÈ□—•¡˜□□ß8

ÃDÖôï□i□jšUÊÜYÎ2Ž□□8 ƒÈ ñƒWÉ!I□'Ðw®+áf‰>‹àÐn

□Kû™o¼«y□'Â$#□¬8 :q]µ p¿ð›ø"?òNõ□ü□‡ÿ Š©t?\êž-_

ßxjûL»kV°ÌÒÆàF

Ü•¤ã'Šì¦š;x$žgTŠ5.îÇT

’kƒøi□šÃêþ6»FYu¹ñj¬9ŽÒ<¬cÛ8$úðh□¿¢Š(¢Š(¢Š(¢Š(¢Š(¢Š(¢Š(¢Š(¢Š(¢Š
(¢Š(¢Š(¬•[ýz□»ýkZ²uoõéþïõ

úµ§□Çê}□òªµkNÿ •Ôú-
å@□”QE □QE □QE □QE □QE □Q\ŸÄÍbm□áÆ¹¨[9IÒßË□ÇUg`€□q»4□OPø§¢[j3ØéÖ:Æ¹5»lŸ
û"ÈÎ±7¡l•ùf°/

ø†ÓÄ°a¾´†î□Y

O□Ü&)□†2
Ÿ¨¯?ðç•¼9áÏ□Ùé°-•â□BÂÖ
¯ya¦3DÍ□™Ë□3“’N+Ð|;âM/ÅZBjzEÈžÙ‰SÁVF□U□ä□@□„…□’ □’{W□?ÅÝ□M0Ó´ÍwWµ□ŠËy§X
□`B:üäŒ•ê+oâ
Ý7ÃÏ□-•cpl%Ú□®6œãß□¨>-_i□ü=ÑN™=¿Ù¡³•d□Ã÷n□o□èÛ³œ÷æ€64□□iž'Ò"Õ4›•¸µ—
0□□#ª°<,=+N¼ËáåÍ”□□¼y§ésÃ6šg·»□ÈpÈ²:7□ãï

~□é´ QE□ QE□ QE□ QE□ QE□ QE□ QE□□ƒ}ÿ -
²ýGò©t¿øûÿ €šŠûþ?eú•åRé□ñ÷ÿ □4□³E□P□E□P□E□P□E□P□E□P□E□P□\¦¯ñ
EÑü]cá©–êkû·Ž=ÐF□8YÉØ$$Œ□,p□8æ´<Yâ8|-
áùõ□#3M‘□µ°ýéæn□□¹?¦Ojó]SÃ"h+à©µ□□ÚÖ£âXnµ□ÿ ½++|£ý•(□8÷ □e¢Š£¬êöz

•wªê□ùv¶±™$nø□‡©' □S@□^/ø…¢ø.{H5□°š{žDv±‡1¦à»ß$aw□3Ü×W^□â½"ñ¾□ê¾*ÖâÙ¬ë□
V□å7ü°Û‰“Ë„z`rÞ¤óÒ½Ê€
(¢€
(¢€
(¢€
(¢€
(¢€
(¢€
(¢€
æßýc}Mt•Í¿úÆúš X¿×'ûÂ°:ç"ÿ \Ÿï
èè ¢Š(¢Š(¢Š(¢Š(¢Š(®cÅ~7³ð□Ö□i6™ªê7Z‡˜`ƒM·□9□€X‘¸vaÓ=ë§¯9ñýÝÎ‘ãÏ□kQ
èú¦¥mh—Ë*iÖ¦gRèŠ¼

□Ï©ìh□KJø‰ý©ª[Øÿ Â□âëO=öý¢ïLòâ□Ý›qÀ®Ò¸-#â†…©êðé7v°¦□□9Ä□j¶†□)ôS’3øó]- a
x•\Ôôcn4ÿ

^k-níæÚD_+□Æw□×'ò®róâ-»ae=åß€uhmà•¤–F¹‡
d"ózW WŸüI•õ«•□Àö®Cë□ù—¥O1ÚFw?Óq □^E uÞ-
ÕÆ¿áû□Ym¥¶[ÈVeŠ_¼¡†FqíÍiSb□!‰"‰□FŠ□UF □ □ê (¢Š (¢Š (¢Š (¢Š (¢Š (¢Š (¢Š
(¢Š (¢Š (¢Š (¢Š (¢Š (¢Š (¢Š (¢Š (¢Š (¢Š (¢Š (¢Š (¢Š (¢Š (¢Š (¢Š (¢Š (¢Š
(¢Š (¢Š (¢Š (¢Š (¢Š (¢Š (¢Š (¢Š (¢Š (¢Š (¢Š (¢Š (¢Š (¢Š (¢Š (¢Š (¢Š (¢
Š (¢Š (¢Š +
Äž+Ð¼2,ã×n–Þ+÷0ÆÎ…□œs»□□Ï~9-ÚË×¼;¤xŸN6□Í„W-ÙÜ□ò
ŸU#□O¸4□ÀüGÿ …ysàMJy_DyÍ³›)-š3)——
&Âœ'»□íŽ¼VÇÁÃ|~□h¦üÈ_cù~fwy{Ûg^ØÆ=±U¬þ□ø□Îð\•)æ*Á–
9§v@~™ä{□× Ç□E□Ç□*"€ªª0 □ □ ê(¢€
(¢€
(¢€
(¢€
(¢€
(¢€
(¢€
(¢€
(¢€
(¢€
(¢€
(¢€
(¢€
(¢€
(¢€
(¢€
(¢€
(¢€8?Œ677□□.®-#2O§Í□ê î#`[òROá]6Ÿâ]RÐ#ÖàÔ-ÿ ³Ú1#LÒ #□É

sò'Ü–•ª@`A ƒÁ□¸É¾□ø

{ózþ□¶ó‹n!]Õ3þàm¿†(□;áq:-÷Š¼T¨Ëk«ê8´f□ó!^lWú□ŸÈ×¢ÔpA□´□Á□I□1¨TŽ5
ª□@ è*J (¢Š (¢Š (¢Š (¢Š (¢Š (¢Š (¢Š *¦¥ÿ -Mõ-
Î-ÕMKþ<›ê?• >Çþ<¢ú□Ž±Uì□åÊ/§õ«□ QE□ QE□ QE□ QE□ QE□ QE□□ÈüRÿ ’aâ-
úóoæ+oÃŸò+é?õå□þ€+•Ôþ□xXÔîu□ý□Î»¹s$²}®uÜÇ©Àp□à*ç‡>□øSÂz‹ê□&•ö[§^ÄÒ}¢WÊ□
□f#¨□□ÐE¨ÙO{=”7–ò]Û€f□%S$`ò7(9□íš-µ
+Ï3ì·–óùlQü©Cl`pAÁàƒU,ü9¤Øk·ÚÝ-šÇ¨ßª-Ìá˜—
08'□ è□k□SøQà}^ú[Ûß□À×□¹y-9d‹s□’HF□$Ð□%ÜözÏÇ}□M-Hæ“M°ŸûRhH*□†#□˜q□Ç8¯M¬
Í□ÃÚG†ì□¦□§Áe□9e‰pXú±êO¹-: (¢Š (¢Š (¢Š (¢Š (¢Š (¢Š (¢Š ÍÕþì_Sý*¥‡ü~Åõ?Ê
-êÿ v/©þ•RÃþ?búŸå@□´QE □QE □QE □QE □QE □QE W¿¾¶Ó4û‹ûÉDV¶Ñ4²ÈA;QFIÀç ª‰¨
hšÞ□×+sa}§:åœ°I□-ùãó-□#IchäEtpU•†A□¨"¸»□„^□¹»7RxnØHNq□’"□ß
Á□J ÉøGä-×‹□IbÞ□ML•;□(

?y³ýœíÆ8¯C´Ôl¯Úu³¼·¸ky

S□eW1¸ê-ƒÁö4¶V6šmœVv6Ñ[[D6Ç□H□T{□Tô□

i:□·Òivkn÷Ó›‹’□›Ì□õ<“• À

Wâ□ì÷ZvŸá□>B·þ!¸û1eë□¸ægú□ãèMvöVpiÖ□öV±ˆííãX¢Aü*£ ~Bª>ƒ¦Éâ□µç¶Ý©Enm£˜»□
'"'□çh$÷Æ{f´¨ ¢Š(¢Š(¢Š(¢Š(¢Š(¢Š(¢Š(¢Š(¢Š(¢Š(¢Š(¢Š(¬•[ýz□»ýk
Z²uoõéþïõ

úµ§□Çê}□òªµkNÿ •Ôúå@□”QE □QE □QE □QE □QE □Ç|UÒæÖ>□kÖvè^_ Jª:Ÿ-
ÖB□¾□»□(□□Ã~%ÒµŸ
Ùêð^[-±·V”™ □•>eoîàäs\§Â£□Ý÷Œ5›%Û¤_êÌÖ□Œ,›F□×Ø“×ÛÚµ®¾□x□óPké¼
9lgfÜÛ□Ñ
ÿ qX/é]l□vÖÖigoo□VÈ›□□Ô*ªú 8□€!·ÕtëÍ=¯í¯íg²PÅ®#™Z0□ïe•Ç□9ô®/U
øwðÉ„šÞ¡¥éÑB□kÓ‹–Š#ß8V
úWS¤ø_EÐô7Ñtë□âÓ_~ûv,êÛþð;‰$zÀƒá□€-ï□ÒxnØÈ

q$':□ß

Å□J ÍøU¦Ú=×^|I§X%Ž—©Ï□Z|)–

□©Q ^Û‰cÏ5é□ÔDŽ5Ž5TE *¨À v□•@□□Q@□□Q@□□Q@□□Q@□□Q@□□Q@□□Q@□7ßñû/Ô□*—
Kÿ •¿ø   ¨¯¿ãö_¨þU.—ÿ -
□ð□@□4QE □QE □QE □QE □QE □QE y÷Œü7ã□SÆ:V±¡Ë¡Éi§BLVÚ£M…□‰□LF9!v€Iãž;×#ã□
øYÚ-□þÕÿ „SÌþÙ‹ì□eûF<ì6ß3wðuÎ9¯oª□-
‹§êòØË}n&{□…°¶;ÙvJ□□¸#=O□"€+Øß^i¾□ŽïÅ—
ZU-Ògí3A!KeË□¸i9□□ëÞ¹Ï^^□ñ□‰¿±Nƒ6•ö-
³ýª{}E¤ò§a□+>X%"|Ç□ ñÖº•sBÓ|I¤Ë¥êÖ¢æÊR¥â,Ë'□#• Ž@ïW¡†;x#,□   □j□□t

□@-'ñ7þ□Gü!□Û¿ðŠ}ƒÏƒwØ~Ñæîó□n7ñŒã>Ù¯Wõçü$□ÛÏÿ ?öWÛ¼Ó³û3Ìòü¼

gÌçvw{c□gYÑtÿ □i¯§ê-
ââÕÙ]£ÞË’¬□r¤- Uú (¢Š (¢Š (¢Š (¢Š (¢Š (¢Š (¢Š +›□õ•õ5ÒW6ÿ ë□êh□bÿ \Ÿï
èëœ‹ ýr□¼+£ □Š(□Š(□Š(□Š(□Š(□«›û1{ö#w »
Èó□ý§8;sœpyö«□Îx‡À~□ñ\ëq-èðÝN«°K¹‘öõ□r•qÉ

Sã%Å□þ…c ÛI□°ýÕü`‰□2ÆÁ²_□•□sÏ½z-
æ£e§,M}yoj²È"ŒÏ* w=□dòO¥cx{À~□ð¬-6‹£[ÚÌFß;,òcÐ3□@üjö·áÍ'ÄqZÇ«Y-ÊZÎ·□,Ì»
d^‡,3×¡â€4É
¥˜€ É'µyçÃà|Kâ□wÇR,a°“ì□f{ZÄyaþûäýA®öòÒ□BÆâÊåY ¸□¢‘UŠ’¬0@#□pzŠ□LÓlôm2ÛN
ÓàX--
•G□`“µG¹äýM [¢Š(¢Š(¢Š(¢Š(¢Š(¢Š(¢Š(¢Š(¢Š(¢Š(¢Š(¢Š(¢Š(¢Š(¢Š
(¢Š(¢Š(¢Š(¢Š(¢Š(¢Š(¢Š(¢Š(¢Š(¢Š(¢Š(¢Š(¢Š(¢Š(¢Š(¢Š(¢Š(¢
Š(¢Š(¢Š(¢Š(¢Š(¢Š(¢Š(¢Š(¢Š(¢Š(¢Š(¢Š(¢Š(¢Š(¢Š(¢Š(¢Š(¢Š(
¢Š(¢Š(¢Š(¢Š(¢Š(¢Š(¢Š(¢Š(¢Š(¢Š(¢Š(¢Š(¢Š(¢Š(¢Š(¢Š(¢Š(¢Š(
 ¢Š(¢Š(¢Š(¢Š(¢Š(¢Š(¢Š(¨ç…n"1± -
J’Š d1□bXÔ’□ÖŸE□ QE□ QE□ QE□ QE□ QE□ QE□ QE□ QE□ QE□ QE□ QE□ QE□ QE□ QE
□ QE□ QE□□□Íª]□

Ì6úTPéÑÃ*È®ä¯cW(□Š(□Š(□Š(□Š(□Š(□Š(□Š(□Š(□Š(□Š(□Š(□Š(□Š(□Š
(□Š(□Š(□Š(□Š(□Š(□Š(□Š(□«\Ù%Ë†f`@Ç□fŠ ¡ý"□üô□Ô¤ƒOŽ
DŠìHìjÝ□ QE□ QE□ QE□ QE□ QE□ QE□ QE□ QE□ QE□ QE□ QE□ QE□ QE□
QE□ QE□ QE□□Nm:9¥i□Ü□ì)Öö1ÛË½Y‰Æ9«TP□E□P□E□P□E□P□E□P□E□P□E□P□E□P□E□P□E□
P□E□P□E□P□E□P□E□P□E□P□E□P□E□P□T

•□bw¿'Ú¯Ñ@□WK‰X0wàçµ^¢Š (¢Š (¢Š (¢Š (¢Š (¢Š (¢Š (¢Š (¢Š (¢Š (¢Š (¢Š (¢Š
(¢Š (¢Š (¢Š (¢Š (¢Š (¢Š (¢Š (¢Š (¢Š (¢Š (¢Š (¢Š (¢Š (¢Š (¢Š (¢Š (¢Š (¢
Š (¢Š (¢Š (¢Š (¢Š (¢Š (¢Š (¢Š (¢Š (¢Š (¢Š (¢Š (¢Š (¢Š (¢Š (¢Š (¢Š (¢Š (
¢Š (¢Š (¢Š (¢Š (¢Š (¢Š (¢Š (¢Š (¢Š (¢Š (¢Š (¢Š (¢Š (¢Š (¢Š (¢Š (¢Š (¢Š
(¢Š (¢Š (¢Š (¢Š (¢Š (¢Š (¢Š (¢Š (¢Š (¢Š (¢Š (¢Š (¢Š (¢Š (¢Š (¢Š (¢Š (¢Š
*
¹šÞÜÈ □□ëSÕMKþ<›ê?• Mo)šÝ$`□aÚ¥ªö?ñå□ÓúÕŠ (¢Š (¢Š (¢Š (¢Š (¢Š (¢Š (¢Š (
¢Š (¢Š (¢Š (¢Š (¢Š (¢Š (¢Š (¢Š (¢Š -spÐI
¨□{`æ¬Õ□Cýu¯ûÿ áW¨ ¢Š(¢Š(¢Š(¢Š(¢Š(¢Š(¢Š(¢Š(¢Š(¢Š(¢Š(¢Š(¢Š(
¢Š(¢Š(¢Š(¢Š(¢Š(¢Š(¢Š(¢Š(ªW—
•m2"ª□Fy«µ"ª□ÇÌ□îÿ Z Ö¢Š(¢Š(¢Š(¢Š(¢Š(¢Š(¢Š(¢Š(¢Š(¢Š(¢Š(¢Š(¢
Š(¢Š(¢Š(¢Š(¢Š(¢Š(¢Š(¢Š(¢Š(¢Š(¢Š(¢Š(¢Š(¢Š(¢Š(¢Š(¢Š(¢Š(
¢Š(¢Š(¢Š(¢Š(¬–Õ%

FÄàûÖµsoþ±¾¦€:□$2À’□□až*J,Ïþ<âÿ v§ □Š(□Š(□Š(□Š(□Š(□Š(□Š(□Š(□Š(
□Š(□Š(□Š(□Š(□Š(□Š(□Š(□Š(□Š(□Š(□Š(□Š(□Š(□Š(□Š(□Š(□Š(□Š(
□Š(□Š(□Š(□Š(□Š(□Š(□Š(□Š(□Š(□Š(□Š(□Š(□Š(□Š(□Š(□Š(□Š(□Š
(□Š(□Š(□Š(□Š(□Š(□Š(□Š(□Š(□Š(□Š+□ø¯ãWð§‡□ÓOoø□j□ö{5□”Ï□ÿ

€=Èô4□ç□□>(kÃ^šÓÂ·'ÛiÚT‹□åÔAHyØŸ—$-□Ö□îCu□¯|´v-Î
□åš5b}È¯žüw é>□ø)§é□·Ö—7ï¨Å5ÛÅ2¹'R¯»§aÐ{

õ&½÷H»¶»ÒíÞÚâ)•F ´N□□´qÅ]¢Š(¢Š(¢Š(¢Š(¢Š(¢Š(¢Š(¢Š(¢Š(¢Š(¢Š(¢
Š(¢Š(¢Š(¢Š(¢Š(¢Š(□#Å- ¶ð¯†¯µ»µ-□¬{¶□,ìH
£êÄ□Æ¸Û}7â–«`š£x›NÒ®%_14¡§¬ˆ€ò□ä9`}qš—ã?>

´□¿ÕIªÚ¬¾›w÷ý+Ñ(□”ð□Šî<S£\□hÛ-
®–§Ü½□ô□~U•{¯±ÿ -µÕ×□ø□mø‘ñ□SýWÚíHÇMÆ6Ýú×¢P□E□P□E□P□E□P□E□P□E□P□E□P□E□P
□U5/øòo¨þunªj_ñäßQüè□ö?ñå□ÓúÕŠ¯cÿ -Q}?-X □Š(□Š(□Š(□Š(□Š(□Š(□6ñ×Š¼
Y□•-\è6#L³ÒÑŒš□ì`¼ì§□†3Á_öÛƒØ□îô[‰o4->ævÝ4ÖÑÈíŒeŠ,N□¹®□â—
ü“□□ÿ ×›□l[~□ÿ ‘_Iÿ ¯(□ô□@□uæR¿Œ¼Iñ□Äún“âñ£YiMl±Ä4Èn7y‘□'-ƒ×>½kÓkÏïü
âQâÝg[Ð<b°Jj†□-□¦$üÇ□Aó;{□À□h□sÃZ/‰´»™ß]ñoöÜN€GötvÞ[g“”'?Jé+Ï´□x—
Gñ¥¿„üZö—
-}□Í§ê6±ù~iNY□:□□<{uÏ-ƒ@□□Q@□□Q@□□Q@□□Q@□□Q@□□Q@□□Q@□u□õÖ¿ïÿ …^ª:‡úë_÷ÿ
Â¯P□E□P□E□P□E□P□E□P□E□P□E□P□^Qã□xÆ-2=[N³□&”·°À¦ò ×WAœ);

Di×¯Ìxé^¯^}ñ�означ

Û¬Œ-í¢³[·t□ƒ#1À'¯ËëV|□âm[VŸYÑ<A□
¬è³¤SËo'□Èà"p;d□ÇÓ§AÒkzÕ‡‡´{□SRœCkn›□□Sè îIà□Zæ>□é□ñE«x"V„Ûê:ýÈ¹ksÖ□Tb$
?í□<ýh□·¢Š(¢Š(¢Š(¢Š(¢Š(¢Š(¢Š(¢Š(¢Š(¢Š(¢Š(¢Š(¬•Sþ>cÿ wúÖµdêŸ
ñóû¿Ö€5¨¢Š (¢Š (¢Š (¢Š (¢Š (¢Š *□«□ig=ËG$‹

m!H•³¶□p u>,¦¢€<»QñWŒ—ÄÞ□-{x´}'SÔ□¸°uY.dM¤î•¹□z|«ÈîkÔkÏ¾"ÿ ÈÓà/û

□ì†½□€+ß[½Ý…Å´W2ÚÉ,l‹ <Xß□#□†r2:×□ÿ □

µÿ E□Ä?ù
ÿ ‰®ê¹oˆ^!—Ãž□°¸´□µ+’¶–(¿y§"å\{Ž[þ□@□?□dÕîµß□I>»{ªhö—□ÊÒK½¤¼ˆ?zÃ p
z×£Ö'„|?□…¼)§hÑ□ÆÞ
$qür-]¿□$ÖÝ □QE □QE □QE □QE □QE □QE □QE □QE □QE □QE □QE □QE □QE □QE □QE
yÌúßŠ¼câMSMð®¡m£éZTßf¸Ôe·□É4ãï"!ùp½

□+.•¯øŸÃ~4±ð×‹.íµ+}Q□ØjPÀ!c"

´nƒ□ÇLzŽ¹á~

üÞ□¾‛¿ÖÉ«]4¾»·wý(øš1-ø□Óýh×á□×i□wô □D¢Š(¢Š(¢Š(¢Š(¢Š(¢Š(¢Š(¢Š(®m
ÿ Ö7Ô×I\Ûÿ ¬o© □Û?øó‹ýÚž ³ÿ •8¿Ý©è ¢Š(¢Š(¢Š(¢Š(¢Š(®kÄëâë››[/□=□”

¬×:…Òù•#
'÷=NO□ÒÖO^□á þÌð•□f}¿Ì□þÒó<-œçîsžŸ- qÚ¾2ðgŠ´K/□jöÚî•¬\}'g[E·-
Hùx^□?□~□ý6¼nâã^Ò|o¤jŸ□mâ-
Ñ&òtë□6Oô;ißŒÈŒ7äöbH-•Ç²P□^Iªxï^¼ñ¶•&•r•øeµˆ´-
>R±½~L¬¬FB®□,:œœ×QãÍZòO²xOE"¦±¬eL«ÿ .¶ÃýdÇÓŽ□Ôž:V/Œ´›=
>-iv□□-muÛxã_`-Éõ$òOrh□Óh¢Š (¢Š (¢Š (¢Š (¢Š (¢Š (¢Š (¢Š (¢Š (
¢Š (¢Š (¢Š (¢Š (¢Š (¢Š (¢Š (¢Š (¢Š (¢Š (¢Š (¢Š (¢Š (¢Š (¢Š (¢Š (¢Š (¢Š
(¢Š (¢Š (¢Š (¢Š (¢Š (¢Š (¢Š (¢Š (¢Š (¢Š (¢Š (¢Š (¢Š (¢Š (¢Š (¢Š (¢Š (¢Š
(¢Š +"ñwÃ•

øÚêÚçY†w–Ý

hÑLSå'8?□u”P□Ï□□~□è~□ð¤wÞ□±¿-óíqÆÊ□¥ù□lð□®9¯]ðO,ô□□éM□•

Ñ›•²MæÈX–
ÛŽý;×OE □QE □QE □QE □QE □QE □QE □QE □QE □QE □QE □QE □QE □QE □QE □QE □QE
□QE □QE sž;ðÉñwƒu□-9□W□¨x$=□E!—è20}‰®b×â–
‰mtô²Ô|□¯M–Æ›□-âSk+Ž7y¹Âƒ×¡Ç½zU□□È|=ðÝþ…¥ÞÞkM□k:µÛÞÞ□ÎV6n‘ƒÜ(þg-uôQ@□□Q
@□□Q@□□Q@□□Q@□□Q@□□Q@□□Q@□TÔ¿ãÉ¾£ùÕ°©¨,lÛ-£ùÐ□ì□âÊ/§õ«□^ÄbÎ,ú□Ž±@□□Q@□□
Q@□□Q@□□Q@□□Q@□□Q@–[ñ□Åw:†ƒ¯xjÓÂ–*žâHÚÞ;˜´ÒÖîs÷ƒ□’¾ø–ß□ø®MNÐÓG—
Ã^"Ód¶³@×□…‡�much
□
¤+däž c 5ÚÑ@□Zv»□{â–WH›C°¶³²XÚ□BB|»’À□□ŒqìONqX7_□õ–
6úæÖ÷À~%□.VH¥°·[˜åPp□ Œd`ã¶k»¢€<ïD±×<Uã»O□k□TšE†›o$:u¤ì

îòpò8tmã□kÑ(¢€
(¢€
(¢€
(¢€
(¢€
(¢€
(¢€
(¢€(êë-□ßÿ
½ToÁ3[`□-
øUê (¢Š (¢Š (¢Š (¢Š (¢Š (¢Š +Æ~"x®óÄ°□Òì|□âñ4W±Jd"J;□c|œ□NsŽ8¯f¢€9ï

ø¯þ□V¹Ø□î•ä□9Õlü□&s÷99Æ9úŠ"Ã°íþµ>«□î‡u¥-□ÛA
ÎN.Pt‛r□□Û#ÜÖí□□â/âký_Å□ªxŸÁ^/žÒÂSý™§ÛiLð¡-
òÚBHß' Æ□¶z×£øoÆ□ð‛ßÉiÿ □×ˆô½‛□<íNÃÈ□°@Ú□qËsœz□]=□ QE□ QE□ QE□ QE□ QE□
QE□ QE□ QE□ QE□ QE□ QE□ QE□ VN©ÿ -
1ÿ »ýkZ²µ0MÌx□ü¿Ö€5h¢Š (¢Š (¢Š (¢Š (¢Š (¢Š *+©þÍi5Ç•,¾R3ùq.ç|

áGr{
–Š ño□ø¾ó]Ö¼3ykà□□¤z]ÿ Úg□é$□]¸ÂáŽO×□éþ-ñ□ñ

›5àÑµ}4Äå□□□·'#à□•□9□ã> ÖÕ□□‰á=nûÄ:□j□†‰s£\4Œ¦Òä□à□€y óô®vúÂ÷ÄŸ□lÍÍ¤ñèÞ
–ƒÏŽI#*—□r

“Ã□^r:-
-w´P□E□P□E□P□E□P□E□P□E□P□E□P□E□P□E□P□E□P□E□P□E□P□E□P□E□P□E□P□E□P□E□P□E□P□-
Z¾·ð×Ä:ÔIáíGYðî§v×Öï¦F%-ÞGûèS ã□ƒíß'□¬-
µ¿□øßL×µ=-çGÑ4`ïioy□=Äî1½□}Ð£§¿®N=&Š (¢Š (¢Š (¢Š (¢Š (¢Š (¢Š (¢Š (¢Š +›
□ð•õ5ÒW8êÞc|§©í@□-
□ñç□ûµ=AgÅ¤_îÔô QE□ QE□ QE□ QE□ QE□ W%â_□ø‹Ãú¼Omá‰õ•□á□ä±`n"—
'?»?yq·□Ç~k-¢€<£]¹Öþ'-ž‰má□SHÒ□Ôsß^jÑ□_b□ìDÉ$ŸZî¼O®ßèPXÉa¡ÝjÍsv□H-äƒ
□æC€x□ö□õ□»E rþ#øuá_□ê)□®i_j°H„+'Ú%□□ a□

¬□à<]ð{Ã6·¾□]□Ã"´Sj'Ç□âM<€[□w□%ŽÑÓæãë^ÏE gèš&ŸáÝ"□+J·û="□¼¸·³íË□<±$òOz
Ð¢Š (¢Š (¢Š (¢Š (¢Š (¢Š (¢Š (¢Š (¢Š (¢Š (¢Š (¢Š (¢Š (¢Š (¢Š (¢Š (¢Š (¢Š
 (¢Š (¢Š (¢Š (¢Š (¢Š (¢Š (¢Š (¢Š (¢Š (¢Š (¢Š (¢Š (¢Š (¢Š (¢Š (¢Š (¢Š (¢
Š (¦JJÆH85_Í□ï□ ·ETó_ûÆ•5ÿ ¼h□Ý□SÍ□ï□<×þñ
tUO5ÿ ¼hó_ûÆ€-ÑU<×þñ£Í□ï□ ·ETó_ûÆ•5ÿ ¼h□Ý□SÍ□ï□<×þñ
tUO5ÿ ¼hó_ûÆ€-ÑU<×þñ£Í□ï□ ·ETó_ûÆ•5ÿ ¼h□Ý□SÍ□ï□<×þñ
tUO5ÿ ¼hó_ûÆ€-ÑU<×þñ£Í□ï□ ·ETó_ûÆ•5ÿ ¼h□Ý□SÍ□ï□<×þñ
tUO5ÿ ¼hó_ûÆ€-ÑU<×þñ£Í□ï□ ·ETó_ûÆ•5ÿ ¼h□Ý□SÍ□ï□<×þñ
tUO5ÿ ¼hó_ûÆ€-ÑU<×þñ£Í□ï□ ·ETó_ûÆ•5ÿ ¼h□Ý□SÍ□ï□<×þñ
tUO5ÿ ¼hó_ûÆ€-ÑU<×þñ£Í□ï□ ·ETó_ûÆ•5ÿ ¼h□Ý□SÍ□ï□<×þñ
tUO5ÿ ¼hó_ûÆ€-ÑU<×þñ£Í□ï□ ·ETó_ûÆ•5ÿ ¼h□Ý□SÍ□ï□<×þñ
tUO5ÿ ¼hó_ûÆ€-ÑU<×þñ£Í□ï□ ·ETó_ûÆ•5ÿ ¼h□Ý□SÍ□ï□<×þñ
tUO5ÿ ¼hó_ûÆ€-ÑU<×þñ£Í□ï□ ·ETó_ûÆ•5ÿ ¼h□Ý□SÍ□ï□<×þñ
tUO5ÿ ¼hó_ûÆ€-ÑU<×þñ£Í□ï□ ·ETó_ûÆ•5ÿ ¼h□Ý□SÍ□ï□<×þñ
tUO5ÿ ¼hó_ûÆ€-ÑU<×þñ£Í□ï□ ·ETó_ûÆ•5ÿ ¼h□Ý□SÍ□ï□<×þñ
tUO5ÿ ¼hó_ûÆ€-ÑU<×þñ£Í□ï□ ·ETó_ûÆ•5ÿ ¼h□Ý□SÍ□ï□<×þñ
tUO5ÿ ¼hó_ûÆ€-ÑU<×þñ£Í□ï□ ·ETó_ûÆ•5ÿ ¼h□Ý□SÍ□ï□<×þñ
tUO5ÿ ¼hó_ûÆ€-ÑU<×þñ£Í□ï□ ·ETó_ûÆ•5ÿ ¼h□Ý□SÍ□ï□<×þñ
tUO5ÿ ¼hó_ûÆ€-ÑU<×þñ£Í□ï□ ·ETó_ûÆ•5ÿ ¼h□Ý□SÍ□ï□<×þñ
tUO5ÿ ¼hó_ûÆ€-ÑU<×þñ£Í□ï□ ·ETó_ûÆ•5ÿ ¼h□Ý□SÍ□ï□<×þñ
tUO5ÿ ¼hó_ûÆ€-ÑU<×þñ£Í□ï□ ·ETó_ûÆ•5ÿ ¼h□Ý□SÍ□ï□<×þñ
tUO5ÿ ¼hó_ûÆ€-ÑU<×þñ£Í□ï□ ·ETó_ûÆ•5ÿ ¼h□Ý□SÍ□ï□<×þñ
tUO5ÿ ¼hó_ûÆ€-ÑU<×þñ£Í□ï□ ·ETó_ûÆ•5ÿ ¼h□Ý□SÍ□ï□<×þñ
tUO5ÿ ¼hó_ûÆ€-ÑU<×þñ£Í□ï□ ·ETó_ûÆ•5ÿ ¼h□Ý□SÍ□ï□<×þñ
tUO5ÿ ¼hó_ûÆ€-ÑU<×þñ£Í□ï□ ·ETó_ûÆ•5ÿ ¼h□Ý□SÍ□ï□<×þñ
tUO5ÿ ¼hó_ûÆ€-ÑU<×þñ£Í□ï□ ·ETó_ûÆ•5ÿ ¼h□Ý□SÍ□ï□<×þñ
tUO5ÿ ¼hó_ûÆ€-ÑU<×þñ£Í□ï□ ·ETó_ûÆ•5ÿ ¼h□Ý□SÍ□ï□<×þñ
tUO5ÿ ¼hó_ûÆ€-ÑU<×þñ£Í□ï□ ·ETó_ûÆ•5ÿ ¼h□Ý□SÍ□ï□<×þñ
tUO5ÿ ¼hó_ûÆ€-ÑU<×þñ£Í□ï□ ·ETó_ûÆ•5ÿ ¼h□Ý□SÍ□ï□<×þñ
tUO5ÿ ¼hó_ûÆ€-ÑU<×þñ£Í□ï□ ·ETó_ûÆ•5ÿ ¼h□Ý□SÍ□ï□<×þñ
tUO5ÿ ¼hó_ûÆ€-ÑU<×þñ£Í□ï□ ·ETó_ûÆ•5ÿ ¼h□Ý□SÍ□ï□<×þñ
tUO5ÿ ¼hó_ûÆ€-ÑU<×þñ£Í□ï□ ·ETó_ûÆ•5ÿ ¼h□Ý□SÍ□ï□<×þñ
tUO5ÿ ¼hó_ûÆ€-ÑU<×þñ£Í□ï□ ·ETó_ûÆ•5ÿ ¼h□Ý□SÍ□ï□<×þñ
tUO5ÿ ¼hó_ûÆ€-ÑU<×þñ£Í□ï□ ·ETó_ûÆ•5ÿ ¼h□Ý□SÍ□ï□<×þñ
tUO5ÿ ¼hó_ûÆ€-ÑU<×þñ£Í□ï□ ·ETó_ûÆ•5ÿ ¼h□Ý□SÍ□ï□<×þñ
tUO5ÿ ¼hó_ûÆ€-ÑU<×þñ£Í□ï□ ·ETó_ûÆ•5ÿ ¼h□Ý□SÍ□ï□<×þñ
tUO5ÿ ¼hó_ûÆ€-ÑU<×þñ£Í□ï□ ·ETó_ûÆ•5ÿ ¼h□Ý□SÍ□ï□<×þñ
tUO5ÿ ¼hó_ûÆ€-ÑU<×þñ£Í□ï□ ·ETó_ûÆ•5ÿ ¼h□Ý□SÍ□ï□<×þñ
tUO5ÿ ¼hó_ûÆ€-ÑU<×þñ£Í□ï□ ·ETó_ûÆ•5ÿ ¼h□Ý□SÍ□ï□<×þñ
tUO5ÿ ¼jÌd"□õÅ :Š(□Š(□Š(□Š(□Š(□Š(□Š(□Š(□Š(□Š(□Š(□Š(□Š(□Š(
□Š(□Š(□Š(□Š(□Š(□Š(□Š(□Š(□Š(□Š(□Š(□Š(□Š(□Š(□Mþ©ª¥[›ýSUJ (¢
Š (¢Š (¢Š (¢Š (¢Š (¢Š (¢Š (¢Š (¢Š (¢Š (¢Š (¢Š (¢Š (¢Š (¢Š (¢Š (¢Š (
¢Š (¢Š (¢Š (¢Š (¢Š (¢Š (¢Š (¢Š (¢Š (¢Š (¢Š (¢Š (¢Š (¢Š (¢Š (¢Š (¢Š (¢Š
 (¢Š (¢Š (¢Š (¢Š (¢Š (¢Š (¢Š (¢Š (¢Š (¢Š (¢Š (¢Š (¢Š (¢Š (¢Š (¢Š (®□Æ□□ì
ü/©Å¢ØióëËÜ □goü□än □'9 □qé'žro‹ >+ÑT]øƒÀWvÚ~Fé£fýØ'□äc>Ç□□ëôVn‰®éþ!Ñ
Õ´Ù¼ÛY"°8Á□u□v ñY¾ ñ□¯Ž4IuKKi-Ò;†·)1□ä□9ãÙ… t"WœØøƒU—ã¾©¡½ë•2-
=dKl□ªÛPç¦s'□ôj (¢Š (¢Š (¢¼"ÄÚÏ‹¼Kñ:ëÁþ□ÕcÒaÓíVy¦eÉ□□‡®   ÿ –
Š1Çs@-·EdøjÛY´ðõ¥¾¿w□Þ¨□„ÓÂ0¯ó□½‡ðíÏ□sZÔ QE□ Q\—€ïü[□azþ.Óâ³¸[,°,`

¦=‰ã=□z±áŸ□ÚxŸV×tû{i¡"H¹û<□!□□å†F?Ü=}¨□¥¢Š(¢Š(¢Š(¢¨ëSÉk¡j□□¶Ùb¶'Ñ±Ñ,
'□r¿ u•C^ø□k}ª]=ÍÓK"™¯□
7□(□¸¢Š(¢Š(¢Š(¢Š(¢Šæ¼3ã;O□êÚîŸom42i□?g'¤#□□°ÈÇû‡¯µ t´QE □Wœø‹Ä□–§Æ•

èÖ÷¯-•sjÏ4 □®ß¼äñŸá•z5 □WŸ|c×u?□x*+½&ñín-ö8Ì^□v□ÄŽG°®òÙÚKH]ŽY‘I>ø
h¢Š (¢Š (¯=ø¿¯]é~□‡LÓ$uÔõ<…´ƒËm®□?1¢ÿ À©|Q¯ßø'Hð×†ô•—
zÆ Ée□÷ŒÌ£hUi□žX~¾˜ □A¢¼ÿ Ã~*×í¼u7ƒüQö)nZÛí6·vŠPH;,§ñôû§®iÞ)ñ-¦<e¦xGÃ
□š”Œ&¾šTÞ–Ðõä□9#ž£¶9a@□õ□Ä|X²¾¸ð%Íæ™uqoy¦°Þ¡†B›,}àqØ)'þ□+ ð¶¹-
‰</¦ë□ãý*□v
Ñ_£□Á•-
… kÑE□ QE□ QE□ QE□ QE□ QE□ QE□ QE□ QE□ QE□ QE□ QE□ QE□ QE□ QE□ QE□ QE□
QE□ QE□ QE□ QE□ QE□ QE□ QE□ QE□ QE□ QE□ QE□ QE□ QE□ QE□ QE□ QE□ QE□ QE□
QE□ QE□ QE□ QE□ QE□ QE□ QE□ QE□ QE□ QE□ QE□ QE□ QE□ QE□ QE□ QE□ QE□ UÈ
ÿ Õ¯Ò©ÕÈÿ Õ¯Ò€□E□P□E□P□E□P□E□P□E□P□E□P□E□P□E□P□E□P□E□P□E□P□E□P□E□P□E□P□
E□P□E□P□E□P□E□P□E□P□E□P□E□P□E□P□E□P□E□P□E□P□E□P□E□P□E□P□E□P□&ÿ TÕR–Íþ©ª¥ □Q
E □QE □QE □QE □QE □QE □QE □QE □QE □QE □QE □QE □QE □QE □QE □QE □QE □QE □
QE □QE □QE □QE □QE □QE □QE □QE □QE □QE □QE □QE □QE □QE □QE □QE □QE □QE
□QE □QE □QE □QE □QE □QE □QE □QE □QE □QE □QE □QE □QE □QE □QE □QE □QE xïÁ¸“Xñ
?<üKr¡îä¼1FÍÉ•If z□°?à5ë×□ðÝÛKmq□Ë

¨RHÜd2‘,□ôÅx~‹¬Åð›âF»§k‘É

•«Kö‹ kµBÊ¼’8□ž□©Ç9□ŒWc¯|fðŽ—
¥K=Ž¢š□æÂa·…[ænÛ‰□QëßØÐ□□ðûÃo®hÞ.ð□Õo,-ôíMeŽ[fÃ7□)□Ô□ƒñ□«|-
ð□öþ□5¯íÝFÏìÚ‰_³Û¶Ô}□□'Üç…wÿ □|3□ŁøVóPÕ#hõ□^cpêã

~×ñw[ðßü$□Œ□c³Y¾Ø-ûÙ2";Xç§Ïÿ ŽŠúJ¼›Ãßòr~*ÿ °d□ů□½ aüAƒÄV¾?ð§‡|=¬]¥ÉÓ□Ýf
yˆÏ.□#{í□'□ãŽq]UŸ,µ□□xkÄ·mâ□CQ2é"□I²<¹□1Þ§qÁþ^¦¨ø¯þN□Â?õäßûV½#Ä□rê>□Õ,a
□–æÒXP□ÉÈ@þt□Êü□¹žïáŽ—-ÌòM)iAy□³-
Þ7sY>□¾»¸ø¯ã˜&°šHc•Dq¼„ªrG ô¬_…¿□¼7áß□.•-ÞýŠöÂY□¡'6,à±#

¹$c¨Å;àþ±□¿ñ□Æ:´□¼pÝ2È^ÿ x)cŒã½ Ið¾ÖïÅ¿□üG§]ê×Ñ¼úƒ(¹I‰′?°x'±Ç?S\Ÿ•>□□ox
‹ÅV□ð□j6ŸÙ—,2□ÃOóH77=~_Ô×qð□þE-gþÂoÿ -cø'ÅZ/ƒüuãèõûÑbÓj□H¼Äc½CËœ

xe#×4□í°Eä[Ç□÷}Š□sœ³`c$ú×+ã•#Å□äv6□□--•hìæþègÍU□Ú□ð,ò:

z×W

©<1Í□nŽE

¬;,2□xßÆ‹ço□x{FÕ/î4ÿ □]□÷s@

]ƒr\àmì~öph\¶Ô¾êZV«¦øÎãV,K¥ŠîÎi·nNWq##8Èãžk¤øß}¬YÝxQ4K©¡¼'íÄb7ÚÆ
Í¹ƒÉïÇ&¼¿Ç_´ô°ƒÂWw7—Æu3LÎZ0ž™ |Äã§¡¯TøÃÿ #/€ÿ ì&ô(¨
Äžño…|57Šÿ ā5Ô'ÕíBË<a˜ÆAa•   <žã

,°/□üBÔm¾□è—ú`   ¬k‹□Q”êØ¯ÎT-
~□×=«§ø¡ÿ $Ï_ÿ ¯SüÅyg‰ìî“à÷□µëhŒ¿Ù.’H p□±ÉöÊ•ÿ □ □=Wá□4□□\ê¶¾5½¸Ô’Ýžê
•Œr®Ó¹A$çŒãpçŽ•Óü

ÿ ’cgÿ]åÿ Ðª·ˆþ0xFO□]□;æ°»»¶xã´HØH□—□lŒ

gž~™«?□ÿ ä˜Ùÿ ×y□ð* Ôø¥¦Þj_□õ□§ÜO□Ýª‹¨Ì2□-³-
□uùwqëŠo…üi□ïÂÈ<MvûÚÚÍ□ÎHËÏ□!¿□##ýá]›(e*À□#□-„WÌ□¢êZ□æ·ð¾ÍXÇ©j°µ±=□lsÉ÷
ýÖOû-@□‡†üGª⌀_á□-ã+û™nu□Vè›D□ÉU$•□iéÎó•Ô(®~Ú
-SO•TÔ¾.M□µ2y»□ßd,yÙÁ□ÇN0□`q^•ñKÂ•7ÂQ¦iq3•(E$q•-dŒm?S´“øW-
áÍKàíß‡m&Ôl´ëKÔ…EÌSDûƒ□†#□Ü
ç•_Z Ùð_Ä}BóáF³«^²ÜêZ8t□□Ä¿.Q›□îp}@õ5ÍØx□Ä>-
ð;x²÷ÆwÂþxd¹Ž□ÇËP3ò’□c8ì03ÓŠêï/<97Áï□Üø□Æ□²ì’)@„ 'jïo›□á□púW•i:• ÐâÒ□â□
£a§L˜¸³6nU ûê»[□rO¦{ŽÔ□é
/ÿ °>□k>/»¿½¼‘U• ¸””□-ví<ýíÊ3\ÖŸs□‹mN³âOŠ□i·ó34vp-
oƒ•À

•□ç"^‹–‛¡kŸ□o´?□]
Ûu·eŒŒù□*□ø`@!˜Ž„

£µq>□½ø['†`¶ñ-••®±m°;•´ÄàÈA8`ztÇ□r□□(□oáþ-{ã]□Ä~

Ô5éî□©Qm«[¹ó$‹w\žHùG^Hb+•ð'Ã•íï□xªÏþ□□FÓû2ðCæ@Øiþi□æç¯Ëúšõ¿]x□î]Hø2Þ□0
ùks$P2nÎí¸,2G□¸O□ø«Eð□Ž¼}-¿z,Zm@É□˜Œw¨ys€□Ï

¤zæ€;O□øgÅ□íí¥…—ˆŽ‟ Al>Ñ:1ûD²□A□Œq´

àdœfÅpö'Qø{ñAÓ-|U.µ¤êÒy□C,»ÌlH^FH□$□Œg□T_□ï¬µ¯Š□šW‰õk›□
ý''·hr□RË□ÇfŽr2G□íœ×8Öþ
µø£á8<□5ÅÂ-ü_išF%□¼ÅÀR@<sž1È

³â□¾¯wñƒÃ–ú□ÂÛêRÙlŠf#□å¤Üz-‹Ÿ□Nj?□x?Å-
□4□øJ´ï□ßßOk"5ÜW□¶H□,ç□ŽFHàúç<Tÿ □õè<3ñ—Ã:½ÔRI□½ždXþö
H   Læ¬üLø•áÝwÀóhÚ□éÔ/õ6Ž8âŠ&Ü£x' •Ï□□®M ?ã□¨°ßÂ□TDØ.îm¦Ùœí-□□?

• ¹ ñ7Æw>□ðM'ÓÝ#Ô/ŠÃ

Œ2"]¹gÁêG□ñö®oâŽ™.•ðGAÓg Mm5´r€r□ˆßwëšµñ³GžïÁú&¯□±¹‹L•Zx»□ÝFIöÊ•øÐ□□woe
c¦ÉªZ|]ž}n(Ì»<ÇÛ+□□€ç<ôÉëè+³½øƒ¬Þü□ÿ „ŽÐùz¦E´óF£÷g~Òàv$cè[Ž•^ßQø#>œ—
om§C•□¡’ <Ä>…@9ü2+-‹ÄÞ□Ñ<□e}□q[øruV·□>ÊÅX’ûƒ& ÇÈÙÈ

KÂ>□¼¾]7Ä□?ÄkËÉ7Ç-Ò□.®8,Œ7ä□da‡å^Í_7ø²o
i□¦™ª|5Ôå]nk¥O²Z□hÝ[=A□ÛhÛœ□zq_H

ãž´□äž6'Røíà½.Nb·ˆÞ(ÿ ksŸý¤µµñ+Ãš¶¡}áß□h-
ÂòïE°óšÓxC2□¤€O□ù□ŽÃñ©:oÇŸ□jrq□Ä?cSþÑg_ýªµëSM□¼□O4‹□Q©wv8
 d’{
 ñMgVÔô¯□\üD×t†Ó|‹?°éV□H-,ó6y!z(Ë□ÓŒbŸàÏ□øwÀÆòãÄ—òÍâk÷□jr¤□
µÜr#fQ€Fy□¿□…_ðä2üOñãø®ò6--Ò□Å¥Bã□i□&B□à~»G;McxGPÓ´ï
          |D³Ö®`‹R3Ýyé3…yrŒ □ûß6ìc¹÷ □g”Zk°□‹

©=¥õ»*°œ«£®3ôÁ¯:ø□y%ÇÃé rH¶¿'8Áì¥U¿›5iü0-]#àÞŸuz□□□ó\aûG½Ø-
¡□•b+3à□£ÁðþyÝH□7ò<d÷P¨¿Ì5 z•□Q@□□Q@□□Q@□□Q@□□Q@□□Q@□□Q@□□Q@□□Q@□□Q@□□Q
@□□Q@□□Q@□□Q@□□Q@□□Q@□□Q@□□Q@□□Q@□□Q@□□Q@□□Q@□□Q@□□Q@□□Q@□□Q@□□Q@□□Q@□□
Q@□□Q@□□Q@□□Q@□□Q@□□Q@□□Q@□□Q@□□Q@□□Q@□□Q@□□Q@□□Q@□□Q@□□Q@□□Q@□□Q@□□Q@□
□Q@□□Q@□□Q@□□Q@□□Q@□□Q@□\•ýZý*•\•ýZý(□ÔQE □QE □QE □QE □QE □QE □QE □QE □
QE □QE □QE □QE □QE □QE □QE □QE □QE □QE □QE □QE □QE □QE □QE □QE □QE □QE
□QE □QE 2oõMU*ÜßêšªP□E□P□E□P□E□P□E□P□E□P□E□P□E□P□E□P□E□P□E□P□E□P□E□P□E□
P□E□P□E□P□E□P□E□P□E□P□E□P□E□P□E□P□E□P□E□P□E□P□E□P□E□P□E□P□E□P□E□P□E□P□E
□P□E□P□E□P□E□P□E□P□E□P□E□P□E□P□E□P□E□P□E□P□E□P□E□P□E□P□E□P□E□P□E□P□E□P□
E□P□E□P□E□P□E□P□=GJÓõ‹o³jV6÷□ç!'Œ8□Ôg¡÷¬½?ÀÞ□ÒîVæË@°Šu;-A□,§Ô□Ðý+ ¢€
Ä—ÁÞ□›W□´š%‹_‡ó<ó□Ü[®ãêsß-mÑ@□□EÓ#Ö¤Ö□Æ□Ô¤ŒD÷!~v^8'ð•□-
‹¦ë□Øê6PÜ›iD¯ù«□Ž;Š¿E Eqo□Ý¼-÷0Ç4□□¯□Š□Xz□x"¹ø¾x>□ƒ:xoMÞN~h□‡äx®-
Š ¡ªhšfµb,µ+□.-Uƒ□¤\¨#¡□yT*…P □

Ô´P□T!ÑtÈ5›□b+□SQ¸A□×!~wQŒ□□à#ò-•~Š ¡>‹¦]jÖÚ¬ö0É□1¥!¸eË 9È□ñ?□_¢Š Â½ð_†
u□DßÞh:|÷Då¤x□->-ýïÆ¯Yèš^□}u{ga□□7[|ù#L□ÇLÕú(□†•¢éš□2Ã¥ØÃi□²□dX—
hf=OéU5?□xwY¾[ÝKE²º¹□ýì°,Ç□3ëøÖÕ□ €□Pª `□ÚªjzN•¬Ú}—S²·»ƒ;¼¹ã

□õ□è}êå□□Î§€¼'□ ¶O□iâ□"É•
d²ç□ž§□?™-=GEÓ5i-&Ô,a¹’ÒO6Ý¤\˜ÛŽGä?*¿E W½²µÔ¬¦³½□'¶™JI□Œ†-†›o§YZé©§Ck□Y
$~RÀ□lÙŒc-•jŠ Á²ðO…ôë¶°³Ðtøgl□ë□äd`ãÓŽ8--/J°ÑlRÇM´ŠÖÙ
+□c □rjå□ VtÚ□“q-C¬Í§Û¾¥
ìŽå“çQÏ□Äþu£E □ÎÝx
Âw·fêãÃÚ{ÎÇs7’□âz’□□×EE q_□</=÷ÃmCEðÝ¤6îB²[@«□¸

□”□€ Çãø×□i~3ƒJÑ-t{¯…7□wo
Å°Ú,$`0IÝ□ “Ï~½ëÞ¨ □1øAá•OG]sXÔtõÓ□-p²A§•·È@Xã-
Ã÷ð□□m®ÃSðW†u‹³u¨hv3Ü1ËJÑ Ì}ÈëøÖõ□□OMÒtí-ÜÛé-□Öp“’•D□□êqÔûÕ□OÂ-□Öo-
÷RÑl®®F?{, ±ÇLúþ5µE fjÞ□ÑµØ#‡TÓ-nã•ýX-0v□ºz□ÂªÛø3Ã6-höú□„Mg'›□$ □~>l÷<

O ô-Ú(□:ó@Òu□F-BóN¶žî(Ú$-
HÃ□Œ□eç±□þgÖªiÞ□ðÖ'{öÝ?C±·¹É"T„□_÷Oðþ□¹E QÕ´m7]³û-©e□Ý¸pâ9W 0èjá•

~YE)□»qÆ=1N¢€9£ð÷Áísöƒá½7Ìÿ ¯uÛÿ |ôý+^çFÒï4ÕÓ®tëIlT ¶ï
˜×□0¸ÀÇµ^¢€0ô¿□xoD°ûV›¢Y[\

âT^n_¡<□Â·(¢€<ëã□‡®õ_
Ûêšb;jz=À¹‡Ë\¹^7□÷á[þ□]K[ÚøÏÁ±Ç□□Ä6ú•²4±□hÝ2□□ÏQÏ-ÿ JÜ¢€)éZ]ž‰¥Ûi°|
□¥°□Ž5ì?©=IîMPÔ|□á½_P□ú†‰cst1™e„□ØéŸ_Æ¶è

□â½Ýí§€n,4«Iç°Ô]l£H#-µ[ïd

ƒh+øŠè<%¡'†|)¦èé,ma
åz3ž\•«□kfŠ (¢Š (¢Š (¢Š (¢Š (¢Š (¢Š (¢Š (¢Š (¢Š (¢Š (¢Š (¢Š (¢Š (¢Š (¢Š
(¢Š (¢Š (¢Š (¢Š (¢Š (¢Š (¢Š (¢Š (¢Š (¢Š (¢Š (¢Š (¢Š (¢Š (¢Š (¢Š (¢Š (¢
Š (¢Š (¢Š (¢Š (¢Š (¢Š (¢Š (¢Š (¢Š (¢Š (¢Š (¢Š (¢Š (¢Š (¢Š (¢Š (¢Š (¢Š (
¢Š (¢Š *ä□ê×éTêä□ê×é@

¢Š(¢Š(□’,c

š-å¿÷OåW(
~[ÿ tþTyoýÓùUÊ(□Ÿ-ÿ Ý?•-[ÿ tþUrŠ §å¿÷OåG-ÿ Ý?•\¢€)ùoýÓùQå¿÷OåW(
~[ÿ tþTyoýÓùUÊ(□Ÿ-ÿ Ý?•-[ÿ tþUrŠ §å¿÷OåG-ÿ Ý?•\¢€)ùoýÓùQå¿÷OåW(
~[ÿ tþTyoýÓùUÊ(□Ÿ-ÿ Ý?•-[ÿ tþUrŠ §å¿÷OåG-ÿ Ý?•\¢€)ùoýÓùQå¿÷OåW(
~[ÿ tþTyoýÓùUÊ(□Ÿ-ÿ Ý?•-[ÿ tþUrŠ §å¿÷OåG-ÿ Ý?•\¢€)ùoýÓùQå¿÷OåW(
~[ÿ tþTyoýÓùUÊ(□Ÿ-ÿ Ý?•-[ÿ tþUrŠ §å¿÷OåG-ÿ Ý?•\¢€)ùoýÓùQå¿÷OåW(
~[ÿ tþTyoýÓùUÊ(□Ÿ-
ÿ Ý?•-[ÿ tþUjGòãgÆv,qYÿ ÚËÿ <Oýõ@□yoýÓùQå¿÷OåPÿ k/üñ?÷Õ-
ÚËÿ <Oýõ@□yoýÓùQå¿÷OåPÿ k/üñ?÷ÕÚËÿ <Oýõ@□yoýÓùQå¿÷OåPÿ k/üñ?÷Õ-
ÚËÿ <Oýõ@□yoýÓùQå¿÷OåPÿ k/üñ?÷ÕÚËÿ <Oýõ@□yoýÓùQå¿÷OåPÿ k/üñ?÷Õ-
ÚËÿ <Oýõ@□yoýÓùQå¿÷OåPÿ k/üñ?÷ÕÚËÿ <Oýõ@□yoýÓùQå¿÷OåPÿ k/üñ?÷Õ-
ÚËÿ <Oýõ@□yoýÓùQå¿÷OåPÿ k/üñ?÷ÕÚËÿ <Oýõ@□yoýÓùQå¿÷OåPÿ k/üñ?÷Õ-
ÚËÿ <Oýõ@□yoýÓùQå¿÷OåPÿ k/üñ?÷ÕÚËÿ <Oýõ@□yoýÓùQå¿÷OåPÿ k/üñ?÷Õ-
ÚËÿ <Oýõ@□yoýÓùQå¿÷OåPÿ k/üñ?÷ÕÚËÿ <Oýõ@□yoýÓùQå¿÷OåV!—Î…d□□†qRP□?-
ÿ °□*<·þéüªå□□OË□ìŸÊ□-
ÿ °□*¹E SòßûŠò£Ë□ìŸÊ®Q@□ü·þéü¨òßû§ò«•□ÄâÞ□!]Øí@□yoýÓùQå¿÷OåPÿ k/üñ?÷Õ-
ÚËÿ <Oýõ@□yoýÓùQå¿÷OåPÿ k/üñ?÷ÕÚËÿ <Oýõ@□yoýÓùQå¿÷OåPÿ k/üñ?÷Õ-
ÚËÿ <Oýõ@□yoýÓùQå¿÷OåPÿ k/üñ?÷ÕÚËÿ <Oýõ@□yoýÓùQå¿÷OåPÿ k/üñ?÷Õ-
ÚËÿ <Oýõ@□yoýÓùQå¿÷OåPÿ k/üñ?÷ÕÚËÿ <Oýõ@□yoýÓùQå¿÷OåPÿ k/üñ?÷Õ-
ÚËÿ <Oýõ@□yoýÓùQå¿÷OåPÿ k/üñ?÷ÕÚËÿ <Oýõ@□yoýÓùQå¿÷OåPÿ k/üñ?÷Õ-
ÚËÿ <Oýõ@□yoýÓùQå¿÷OåPÿ k/üñ?÷ÕKo¨
‰„b2¹ïš _-ÿ °□*<·þéüªå□□OË□ìŸÊ□-
ÿ °□*¹E Sòßû§ò£Ë□ìŸÊ®Q@□ü·þéü¨òßû§ò«”P□?-ÿ °□*<·þéüªå□□OË□ìŸÊ□-
ÿ °□*¹E Sòßû§ò£Ë□ìŸÊ®Q@□ü·þéü¨òßû§ò©.îÅ¨RP¶ì÷ª¿ÚËÿ <Oýõ@□yoýÓùQå¿÷OåPÿ
k/üñ?÷ÕÚËÿ <Oýõ@□yoýÓùQå¿÷OåPÿ k/üñ?÷ÕÚËÿ <Oýõ@□yoýÓùQå¿÷OåPÿ k/üñ?÷Õ-
ÚËÿ <Oýõ@□yoýÓùQå¿÷OåPÿ k/üñ?÷ÕÚËÿ <Oýõ@□yoýÓùQå¿÷OåPÿ k/üñ?÷Õ-
ÚËÿ <Oýõ@□yoýÓùQå¿÷OåPÿ k/üñ?÷ÕÚËÿ <Oýõ@□yoýÓùQå¿÷OåPÿ k/üñ?÷Õ-
ÚËÿ <Oýõ@□yoýÓùQå¿÷OåPÿ k/üñ?÷ÕÚËÿ <Oýõ@□yoýÓùQå¿÷OåV —
Î…d□□»T”□OË□ìŸÊ□-ÿ °□*¹E Sòßû§ò£Ë□ìŸÊ®Q@□ü·þéü¨òßû§ò«”P□?-
ÿ °□*<·þéüªå□□OË□ìŸÊ□-ÿ °□*¹E Sòßû§ò£Ë□ìŸÊ®Q@□ü·þéü¨òßû§ò«”P□?-
ÿ °□*<·þéüªå□□OË□ìŸÊ□-ÿ °□*¹E Sòßû§ò£Ë□ìŸÊ®Q@□ü·þéü¨òßû§ò«”P□?-
ÿ °□*<·þéüªå□□OË□ìŸÊ□-ÿ °□*¹Un¯E«ª”-‘ž´ ß-ÿ °□*<·þéüª-
íeÿ ž'þú£ûY□ç‰ÿ ¾¨□o-ÿ °□*<·þéüªíeÿ ž'þú£ûY□ç‰ÿ ¾¨□o-ÿ °□*<·þéüª-
íeÿ ž'þú£ûY□ç‰ÿ ¾¨□o-ÿ °□*<·þéüªíeÿ ž'þú£ûY□ç‰ÿ ¾¨□o-ÿ °□*<·þéüª-
íeÿ ž'þú£ûY□ç‰ÿ ¾¨□o-ÿ °□*<·þéüªíeÿ ž'þú£ûY□ç‰ÿ ¾¨□o-ÿ °□*<·þéüª-
íeÿ ž'þú£ûY□ç‰ÿ ¾¨□o-ÿ °□*<·þéüªíeÿ ž'þú£ûY□ç‰ÿ ¾¨□o-ÿ °□*<·þéüª-
íeÿ ž'þú£ûY□ç‰ÿ ¾¨□o-ÿ °□*<·þéüªíeÿ ž'þú£ûY□ç‰ÿ ¾¨□o-ÿ °□*<·þéüª-
íeÿ ž'þú£ûY□ç‰ÿ ¾¨□o-ÿ °□*<·þéüª!«)8òOýõZ4□OË□ìŸÊ•-
ÿ °□*¹E Sòßû§ò£Ë□ìŸÊ®Q@□ü·þéü¨òßû§ò«”P□?-ÿ °□*<·þéüªå□□OË□ìŸÊ□-
ÿ °□*¹E Sòßû§ò£Ë□ìŸÊ>>¤°ÌÑ~ÉÛß5-
ö²ÿ Ï□ÿ }P□Þ[ÿ tþTyoýÓùT?ÚËÿ <OýõGö²ÿ Ï□ÿ }P□Þ[ÿ tþTyoýÓùT?ÚËÿ <OýõGö²ÿ
 Ï□ÿ }P□Þ[ÿ tþTyoýÓùT?ÚËÿ <OýõGö²ÿ Ï□ÿ }P□Þ[ÿ tþTyoýÓùT?ÚËÿ <OýõGö²ÿ Ï□
ÿ }P□Þ[ÿ tþTyoýÓùT?ÚËÿ <OýõGö²ÿ Ï□ÿ }P□Þ[ÿ tþTyoýÓùT?ÚËÿ <OýõGö²ÿ Ï□ÿ }
P□Þ[ÿ tþTyoýÓùT?ÚËÿ <OýõGö²ÿ Ï□ÿ }P□Þ[ÿ tþTyoýÓùT?ÚËÿ <OýõGö²ÿ Ï□ÿ }P□Þ
[ÿ tþTyoýÓùT?ÚËÿ <OýõGö²ÿ Ï□ÿ }P□Þ[ÿ tþTyoýÓùSaÔ-i-
1□□Ž3š½@□ü·þéü¨òßû§ò«”P□?-ÿ °□*<·þéüªå□□OË□ìŸÊ□-
ÿ °□*¹E Sòßû§ò£Ë□ìŸÊ®Q@□ü·þéü¨òßû§ò«”P□?-ÿ °□*<·þéüªå□□OË□ìŸÊ□-
ÿ °□*¹YÍªª±-QàãïP□¾[ÿ tþTyoýÓùT?ÚËÿ <OýõGö²ÿ Ï□ÿ }P□Þ[ÿ tþTyoýÓùT?ÚËÿ <
OýõGö²ÿ Ï□ÿ }P□Þ[ÿ tþTyoýÓùT?ÚËÿ <OýõGö²ÿ Ï□ÿ }P□Þ[ÿ tþTyoýÓùT?ÚËÿ <Oýõ
Gö²ÿ Ï□ÿ }P□Þ[ÿ tþTyoýÓùT?ÚËÿ <OýõGö²ÿ Ï□ÿ }P□Þ[ÿ tþTyoýÓùT?ÚËÿ <OýõGö²
ÿ Ï□ÿ }P□Þ[ÿ tþTyoýÓùT?ÚËÿ <OýõGö²ÿ Ï□ÿ }P□Þ[ÿ tþTyoýÓùT?ÚËÿ <OýõGö²ÿ Ï
□ÿ }P□Þ[ÿ tþTyoýÓùT?ÚËÿ <OýõGö²ÿ Ï□ÿ }P□Þ[ÿ tþTyoýÓùU˜dóbY1•Ã8§Ð□?-
ÿ °□*<·þéüªå□□OË□ìŸÊ□-ÿ °□*¹E Sòßû§ò£Ë□ìŸÊ®Q@□ü·þéü¨òßû§ò«”P□?-
ÿ °□*<·þéüªå□□OË□ìŸÊ□-
ÿ °□*¹E Sòßû§ò«Q,#P})ÔP□E□P□E□P□E□P□E□P□E□P□E□P□E□P□E□P□E□P□E□P□E□P□E□P
□E□P□E□P□E□P□E□P□E□P□E□P□E□P□E□P□E□P□E□P□E□P□E□P□E□P□E□P□E□P□E□P□E□P□E□
P□E□P□E□P□E□P□E□P□E□P□E□P□E□P□E□P□E□Ëø÷Æ-¾□ðÓê³Äg™œEo 8ó$ •
ì □“þ4□ÔQ^'?Š~2Ùimâ

•□L□)•%®ß□#ÆI+¿pã¶r;Šôß□x®×Æž□µÖ-PÅæåe„¶LR

□sßÔ{□Ò€7è¢Š (¢Š (¢Š (¢Š (¢Š (¢Š (¢Š (¢Š (¢Š (¢Š Žãþ=¥ÿ pÿ *çk¢¸ÿ •i□Ü?
Ê¹Ú (¢Š (¢Š (¢Š (¢Š (¢Š (¢Š (®NïMñ»ÞLö¾!Óc·i□Ä•`IUÏ •Üœw¬˜µ□ÙxëIÐîµk
ä¸•î.V+O,Å
ð 9=[•@-…EqZî»âƒãEðÿ ‡cÑþ]<^Èú^—¼…06-
aÛÖ´4OøN?´Wû{þ□ï°í9û□•æîí÷øÅ t´Q\¯‰<S{c¬Zè□□ž—
úÍÌfb²³¾È ^□os×□ã□ü□ ê¨®□ø«Äþ-Ô¬£ñnŸ¦ÿ gÞÌ [Ý5ßl27Ý

œ^+½ □Š(□Š(□ë/øó‹éV*½—üyÅô«□ QE□ QE□ QE□ UMKþ<›ê?□[ªš—
üy7Ô□: Ä¢Š(¢Š(¢Š(¢Š(¢Š(¢Š(¢Š(¢²¼E¯ÚxkF—Q»

ê¤$qF2ò¹8TQêMsi□ħ&å□ÜZ>ƒm
|ÂÒây□lz□_—4□ÜÑXzï‰#ðç††«¨Û°œª(µ‰·3ÌÝ#Sßœóè
®vmcâEµ™ÔäÐ´g¶Uó□Æ9ä7!z‵»î‶•Lý(□¾«zoü~¯Ðÿ *ÂÐ5»_□èVšµ‵>EÂn
ÝTƒ,§Ü□Ené¿ñú¿Cü¨□nŠ(□Š(□Š(□Š(□Š(□Š(□Š(

Ý_îÅõ?Ò²ëSWû±}Oô¬° (¢Š (¢Š (¢Š (¢Š (¢Š (¢³µýBM'ÃšžÂ¥
+Ëik,è¯÷IU$□ŽÜP□•□Ài÷¿□u-6Öú□ðŠÅs
L□ÅÈ`□□3Žü×ee%üZDrj‰□ß$e¦[Å

È□ŸÎ€.Ñ\/ö¯Ä[Ø•í–ƒ¤Z[žRÒöw7

;r¸U'Ðô-ß□ø•<U ® -
ÚŮe‘¡¸□ŽLr/Qžý□ã@□Ý□üyEôþµb«Øÿ Ç”_Oë\'Œ>"ÞéÖ:³xkI7Í¥□ûeíÈ)m
/T□

•Û
Àîh□Ðèªz UÛßèö7'*¬–□ñÊÁz□Ê
ÇçW(¢Š(¢Š(¢Š(¢Š(¢Š(¢Š(¢Š(¢Š(¢Š(¢Š(¢Š(¬•[ýz□»ýkZ²u
oõéþïõ

ú(¢€
(¢€
(¢€
(¢€
(¢€
(¢€
+3\·Ö.lÑ4[û{;•
,óÁæ,¸<□‘ƒœs\ÝÕ§Ž,¬æ°¸ñN•□□ÆÒHçN8U□$ýïJ íè®?Ã~&»O‡iâO□:!òÚrc•i1çäã=HÆ>¢
¨Ã-üDÔì×S°Ðôx,ä_2+K©Ÿí

‡'ÈÂ,G- wÔV□„üK□Što¶$□mq□-□Í³œ´2¯U?§çZ×‰q%"éi*CrÑ°ŠGMÊ-Ž
□Æ{P□ôW□ý•ãïú□t¿ü□þ*£ð^-¯ê:æ¹k©ÞÚÞYéò-°Ï□-Xi±—
□"÷x□Þ€;Z(¢€□~ðú×K\Òýáõ®–€
(¢€
(¢€
(¢€
(¢€
(¢€0o¿ãö_¨þU^¬_□Çì¿Qüª½ □QE □QE □QE □QE □QE □QY^"×í<5£K¨Ý‡uR□8£□y\œ*(õ&
€5h®□/þ$Ü »‹GÐm¡o˜Z\O#M□BËòæ°›-V-/D:-ª>Ì±Æ-2®dØÇ□hÀù¹8□ë@□□W□aã-
bóÆ°~™>Ž¶□uí¼³D. ûA Ð°□ ŸîœŸ¥w□ QTu•VßCÑ®õ;½ÞE¬FF
98ì=ÏJãàÖ>#j6i©YèÚ□6² ';K‰ä3²''ó

($zâ€=□Ëþ?"úÖõpž□ñ"x£O†ÿ ìÏk2Lð\[¹ÉŽEá†{öüë» □Š(□Š(□Š(□Š(□Š(□Š(□¹
·ÿ XßS]%soþ±¾¦€□E□P□E□P□E□P□E□P□E□P□E□P□EqZî»âƒãEðÿ ‡cÑþ]<^Èú^—¼…06-
aÛÖ´4OøN?´Wû{þ□ï°í9û□•æîí÷øÅ t´V□·eâ{‹Õ}□X²³¶□€ÑÏhdbÙ99Ü8Æ8ö®SÄ×ž9ðÞ”·m
¯i·3K2[ÛÛ¥†□i□à(;¾§ð □J¢£€J-ã□•3m□ÊŽ
cœ{f¹Ï□kº¦‰g¦.□□›Þ_ê□Ù!¼

c]áŽNÒ□U□□èÖ□ñç□ûµ=yöŸÿ
_□þoü!□fÊïÛö-ûsÎ;g□è4 QE□ QE□ QE□ QE□ QE□ QE□ QE□ QE□ QE□ QE□ QE□ QE□ Q
E□ QE□ QE□ QE□ QE□ QE□ QE□ QE□ QE□ QE□ QE□ QE□ QE□ QE□ QE□ QE□ QE□ QE□
QE□ QE□ QE□ QE□ QE□ QE□ QE□ QE□ QE□ QE□ QE□ QE□ QE□ QE□ QE□ WŽücQsãŸ‡VR
•ÖòêXt=□2B?'?•{□ygÆÝ□R½Ò4Ÿ□èñ<×°□×ž#EÜv□¤¶;à¢þ□=¨□Ô•□Ddu

¬0AèEx÷iðÌž□Ö-Á&8õ&*}IE□ù
eçí□¢Ï °Ó4ýAµ¹£Ù□³D
¤¤`e•äg¦□O Ï□7Áï
]øOÀ‘C¨FÑß^L×SDÝcÜ
}ð ‘Ø"@□ý□Q@□□Q@□□Q@□□Q@□□Q@□□Q@□□Q@□□Q@□□Q@□□Q@□Ü□Ç´¿îå\ítW-
ñí/û‡ùW;@□□Q@□□Q@□□Q@□□Q@□□Q@□□Q@□’DŠ''F□^
3□€ êkŠð
>±sªøÂáHmRo.Ð0å-c;Sé’ ?•©>#^Ï&—
iáË□+}®MöU#ªEÖWú□àýk«±³ƒN°·²¶M□[Æ±F¾Š£□€9□wDñRøÜkÞ□:A□§-
›® Òq‰□É□□¸ïëIcâÝwM×ì´□□iv¶æýŠZÞØÈZ□qü□7
žßç□5O^š□‡ªÏ§êÍyfñ□□²Z»G&@9R äs□¨5…q©□ÂÃñ-†°E-Ïö>—
v/§¿š#□;¨ù□7ryë@-‘^u¯]¿„>$ÿ ÂG{kq.□{§‹Y.!Œ¿ÙÝ[?0□€@-
Ÿµuš§‰l´□sJÒgŠá®56u…£@Qv€Nãž:öÍV×ük£xbö;}]®mÒHüÅ¸□îñ-HÛ•□ž:c¸

□â'□t/□ø□P´Ò□åëf6ûDv®±ÀDŠrÌÀcÓêkÖ fkx™þù@[ëŠóÝCU?□&µÒ4{KŸì!:M□□<F4'□î□
nX'□Ojôj (¢Š (¢Š Þ²ÿ •8¾•b«Ù□Çœ_J±@□□Q@□□Q@□□Q@□TÔ¿ãÉ¾£ùÕ°©©□Ç"}Gó

J(¢€
(¢€
(¢€
(¢€
(¢€
(¢€
(¢€<ïâ5Ýä^)ðu½••Ûå73Î–¦A□y#A´–<

n'ð©¯µŸˆšEœ°□Þ‘¡\ÙÂ¦Im-f—Ï□98-ò’□ 5gâ□•ìO¢ø‹Oµ’îm□èÉ$□

»Âã□m□Î ÿ "¨j□□4-_H¸°ðô□šŽ-s□C□ªÚºùlÃ□r@

äóþ4 Ï□ê-þ ¼ø□ıo“gy¨
•-Øªä□î "½-¼×Wð-¥¥x□Ã£O‹íZŸ‡æŠèÂœùØÉ‘□×¯â□-\“âÎ‡-
™M:ÞþëWeÄzp´q&þÁŽ0□z•M Ið°□ôMbÝ8ŠßY°Š!Ø(*xüÍz.›ÿ «ô?Ê¸ÿ □èW>-
ð¤□×Ä□ù•în±ÚG9#ð□-
…v□oü~¯Ðÿ * Û¢Š(¢Š(¢Š(¢Š(¢Š(¢Š(¢Š(□7Wû±}Oô¬°ÔÕþì_Sý+.€
(¢€
(¢€
(¢€
(¢€
(¢€
Íñ

Ÿ.«á-SN□'f°´-□Ëœ(fB□}²kJ³õ-^-□K—Pž
™âŒ¨e¶•Ìp □8ô□Éö

6Í~%hzM´
aáÛè-!H–□%•fuU□ï7Ëœ
ê|/â;□□èqêVñ<-¹£š   >ôR/ÞSþz□X□ñcÃ□□K
/oï1òYÁg/˜ÇÓ•□õ©|□¦ê~□ðv¡yuddÕ.çŸQ{(Ø□½†D`úá@úš ‹Ä-$ñ-›ÝÇ¦ø7Í
†6+□à¼IK/f□Œ6}³V~□□e•
□¦ÝËu!□Þñç□Ë‶í□åÃ'ðžœsÇsU□âÏ†-=—£P²½□□)¬äóséÀ#õ©>-
XÞ›□{^»²’Åu{±,□²Œ:Æ €Ì;□ÎH □T±ÿ •(¾ŸÖ¹□Š
□…þ"Çüú7ó□oÂ>)±ñ$z„□q\ÆÚeËZLgŒ(g□×‟‘õÁö®/â‡Ä□
·…üEá±ªÿ ÄÛÊk□³ýž_õ™é»nßÇ8 □EðçüŠúOýyCÿ
Ó®#À^>ðÏ^lìtm/SûF¡ob□,>D‰´(UnY@8$t5ÛÐ□E□P□E□P□E□P□E□P□E□P□E□P□E□P□E□P□E
□P□E□P□E□P□Y:·úôÿ wúÖµdêßëÓýßë@□ôQE □QE □QE □QE □QE □QE □Äü@šMM´¿□Ú¹□êó
□¤2õKdù¤>ÙÀ□×‘]µpž
Ï^|G¬øÂA˜$o°iÄÿ Ï□ÏÌÃÙ›ŸÀÐ□~)Ä‘x&ÖÆ5   m-õµ» è□wO§□»Ð00:W5ãí
ãÄ^

½²³ÿ •ÅÛ5¿8Ë£ñÁ•bÛ|YÑÅSQ,þÛXUÄšwÙÈdîŒué’(_Ž¼snœD.à”Ós¡-
ú×u\w€ôÛëK-S[Õàk{íZé®ÞÜòÐÇŒ"-
p3ùúÖ×†¼Ggâ-5Kî#•••,è²§€Hýh/ëÃÃ~½Ô€Ý:&ËtÆKÊÜ
Ç~Hü¦ø7B>ð½Œ§uÑS-Ë“’ó7Ìäžüœ} ¬-[þ*‰V@ù¬4%÷~;ªSîÍù×w@Q@
¿x}k¥®i~ðú×K@Q@Q@Q@Q@Q@Q@Q@7ßñû/Ô*¯V/¿ãö_¨þU^€
(¢€
(¢€
(¢€
(¢€
(¢€
ó¿ˆ×w‘x§ÁÖöv?o”ÜÏ:Z™aäÒXð1¸ŸÂ½¸¯ˆ6w±>‹â->ÖK¹´k£$•D2ï
ŒI´w8üŠ -}¬üDÒ,åÔîô•
æÎ2Kmk4¾xAÉÁo”•=®ÃHÕ-µÍ-ÓSµÉ,æ%•

F{pxü+‰Ôþ&iZ¾‘qaáè/5□Zæ&†;UµuòÙ†2ä€ □Éçük Ñ¢¶ð7€¬ãÕ.□pØ[¯Ú%
X□'œ   #qô

½[þK□‡□ìs]Åxþ£ñ□Â“üIÑuhõ]Ö6ÖsÅ,¿g”mfèl·'ð□èñø§J¹ð¼Þ"´–K•:(¤—tq°f
•À+`ç õÅ Y×l¬u□
úÏRp–RÂË3–
±qËdôÇ\ûW–Úx³Å°n,öúu£êz=¹X`×E«îX‡□ü£Ì›Gñ

8ç5Ôø¼ÜøËá{Üè•Lÿ iXî□ÙÆ×•□□P€zàt□qïMƒâ¿…#¶H¦k»;¥P¿a{)<Å#ø@
•n´□Ð|:¶Òmt+!£^ý°ÞWi^èŸšY□å™‡cžÝ°W ×•|,±»ŠãVÔæ²"O¶Ôõ□¹¶³•v²&1¸¯ð-
ôö¯U □Š(□Š(□Š(□Š(□Š(□Š(□¹·ÿ XßS]%soþ±¾¦€□E□P□E□P□E□P□E□P□E□P□E□P□
□®èž*_□•{Ã§H!´å³uÔ□N1#9 ÷□ýi,|[®é°ý–•âÍ.ÖÜß±K[Û□
Bî?€†ä□Ûüâæ©ñ□@ÐõYôýY¯,Þ" -KWhäÈ□*T□Žqõ□°®5/øX~#Ð×Hµ¹þÇÒîÅô÷óDcGu-
"&îO=h□Ò+†cÿ   ?Å□O½§ør=Íè÷r

>»Wò5Òø□Z‡Ã¾□¾Õ¦Á[h‹ *ŸânŠ¿‹ □?□Ìð□‹ 6□áhMîN£|íyxÍÔË'$-
Àü(□§®[Ç□&«-ZiO£5 ¼°Ôc¼QvÌ#;U†

ÐOR?ZÐñ□‰l¼5□œ·±\H·w)m□□□□Íœ□Èã•¯µEâ-
□éÞ□’ØjqÝ¬3†?hŠÝ¤□1Ž□ŽA9ãŽÆ€)OâÏ-øGOMKÄz&‘}£G•>m"Y□•)8ÜVO½•oÌW¦[\Ãyi□Õ»
‰ ™□HÜte# þF¼£]ñõ—
Œü/qáŸ□ÛÝê×ú„FÙ¤□Ï□6êÜ3»¸□ÀÏã^□£iË¤hZ~˜¯½lí£·□ýàŠ□?¥ ^¢Š(¢Š(□°XÚGpn□Ö□œõ‘c□•ãÖ¬QE □QE □QE □QE □QE □QE □QE □QE □QE □QE □QE Gqÿ -Òÿ ¸□•sµÓ□□
ŒƒQù□ÿ Ï$ÿ ¾E s´WEäCÿ <“þù□y□ÿ Ï$ÿ ¾E s´WEäCÿ <“þù□y□ÿ Ï$ÿ ¾E s´WEäCÿ <“þù□y□ÿ Ï$ÿ ¾E s´WEäCÿ <“þù□y□ÿ Ï$ÿ ¾E s´WEäCÿ <“þù□y□ÿ Ï$ÿ ¾E s´WEäCÿ <“þù□y□ÿ Ï$ÿ ¾E s¸□Í□Ñy□ÿ Ï$ÿ ¾E-D?óÉ?ï‘@□í□Ñy□ÿ Ï$ÿ ¾E-D?óÉ?ï‘@□í□Ñy□ÿ Ï$ÿ ¾E-D?óÉ?ï‘@□í□Ñy□ÿ Ï$ÿ ¾E-D?óÉ?ï‘@□í□Ñy□ÿ Ï$ÿ ¾E-D?óÉ?ï‘@□í□Ñy□ÿ Ï$ÿ ¾E-D?óÉ?ï‘@□Ù□Çœ_J±H Q… □ØRÐ□E□P□E□P□E□P□U5/øòo¨þun‘•\a€#ÐŠ æ¨®‹È‡þy'ýò(ò!ÿ žIÿ |Š çh®‹È‡þy'ýò(ò!ÿ žIÿ |Š çh®‹È‡þy'ýò(ò!ÿ žIÿ |Š çh®‹È‡þy'ýò(ò!ÿ žIÿ |Š çh®‹È‡þy'ýò(ò!ÿ žIÿ |Š çh®‹È‡þy'ýò(ò!ÿ žIÿ |Š çh®‹È‡þy'ýò(ò!ÿ žIÿ |Š çh®‹È‡þy'ýò(ò!ÿ žIÿ |Š çjÞ›ÿ -
«ô?Êµü^□ç’□ß"•b□

V5□Ô

}□Q@□□Q@□□Q@□□Q@□□Q@□□Q@□□Q@□°¿Ý‹ê□¥e×HÈ□□ê–□Qšo‘□üòOûäP□;Et^D?óÉ?ï‘G‘□üòOûäP□;Et^D?óÉ?ï‘G‘□üòOûäP□;Et^D?óÉ?ï‘G‘□üòOûäP□;Et^D?óÉ?ï‘G‘□üòOûäP□;Et^D?óÉ?ï‘G‘□üòOûäP□;Et^D?óÉ?ï‘G‘□üòOûäP□;Et^D?óÉ?ï‘G‘□üòOûäP□;Et^D?óÉ?ï‘G‘□üòOûäP□v?ñå□ÓúÕŠ@¡F□ □aK@□□Q@□□Q@□□Q@□□Q@□□Q@□□Q@□□Q@□□Q@□□Q@□□Q@□□Q@□□Q@□dêßëÓýßëZÔÖ□□å‘XûŒÐ□7Et^D?óÉ?ï‘G‘□üòOûäP□;Et^D?óÉ?ï‘G‘□üòOûäP□;Et^D?óÉ?ï‘G‘□üòOûäP□;Et^D?óÉ?ï‘G‘□üòOûäP□;Et^D?óÉ?ï‘G‘□üòOûäP□;Et^D?óÉ?ï‘G‘□üòOûäP□;@

è¼^□ç′□ß"•"ùäŸ÷È

vŠè¼ˆ□ç′□ß"•"ùäŸ÷È

vŠè¼ˆ□ç’□ß"•"ùäŸ÷È

w□Šè¼ˆ□ç’□ß"•"ùäŸ÷È

vŠè¼ˆ□ç′□ß"•"ùäŸ÷È

y~ðú×KQù□ÿ Ï$ÿ ¾EI@□□Q@□□Q@□□Q@□□Q@□□Q@□7ßñû/Ô□*¯]□†&9hÐ"Ü¨¤ò!ÿ žIÿ |Š çh®‹È‡þy'ýò(ò!ÿ žIÿ |Š çh®‹È‡þy'ýò(ò!ÿ žIÿ |Š çh®‹È‡þy'ýò(ò!ÿ žIÿ |Š çh ®‹È‡þy'ýò(ò!ÿ žIÿ |Š çh®‹È‡þy'ýò(ò!ÿ žIÿ |Š çh®‹È‡þy'ýò(ò!ÿ žIÿ |Š çh®‹ È‡þy'ýò(ò!ÿ žIÿ |Š çh®‹È‡þy'ýò(ò!ÿ žIÿ |Š çh®‹È‡þy'ýò(ò!ÿ žIÿ |Š Å²ÿ •È ¾µ½L□Ä§+□□;…□ú (¢Š (¢Š (¢Š (¢Š (¢Š +›□ð•õ5ÒT~DGþY'ýò(□•¢°/"- ùäŸ÷È£È‡þy'ýò(□•¢°/"ùäŸ÷È£È‡þy'ýò(□•¢°/"ùäŸ÷È£È‡þy'ýò(□•¢°/"- ùäŸ÷È£È‡þy'ýò(□•¢°/"ùäŸ÷È£È‡þy'ýò(□•¢°/"ùäŸ÷È£È‡þy'ýò(□•¢°/"- ùäŸ÷È£È‡þy'ýò(□• -´WEäCÿ <"þù□y□ÿ Ï$ÿ ¾E s´WEäCÿ <"þù□y□ÿ Ï$ÿ ¾E 2Ïþ<âÿ v§¤ (ì)h ¢Š(¢Š(¢Š(¢Š(¢Š(¢Š(¢Š(¢Š(¢Š(¢Š(¢Š(¢Š(¢Š(¢Š(¢Š (¢Š(¢Š(¢Š(¢Š(¢Š(¢Š(¢Š(¢Š(¢Š(¢Š(¢Š(¢Š(¢Š(¢Š(¢Š(¢Š(¢Š(¢ Š(¢£¸$[ÊAÁ□□•`úó□ÏWÿ ¾• tTW;çÍÿ =_þú4yó□ÏWÿ ¾ • tTW;çÍÿ =_þú4yó□ÏWÿ ¾• tTS"9‰

þè§Ð□E□P□E□P□E□P□E□P□E□P□E□P□E□P□E□P□E□P□E□P□E□P□E□P□E□P□E□WP f[F*H9□ƒ@□¨®wÏ›þz¿ýôhóæÿ ž¯ÿ }□ è¨®wÏ›þz¿ýôhóæÿ ž¯ÿ }□ è¨®wÏ›þz¿ýôhóæÿ ž¯ÿ }□ è¨®wÏ›þz¿ýôhóæÿ ž¯ÿ }□ è¨®wÏ›þz¿ýôiVyw•Þ¿_ï□ èh¢Š (¢Š (¢Š (¢Š (¢ Š (¢Š (¢Š (¢Š (¢Š (¢Š (¢Š (¢Š (¬ýQÝ□- ŒË'z□zVoŸ7üõ□ûèÐ□EEs¾|ßóÕÿ ï£GŸ7üõ□ûèÐ□EEs¾|ßóÕÿ ï£GŸ7üõ□ûèÐ□EEs¾|ßóÕÿ ï£GŸ7üõ□ûèÐ□EEs¾|ßóÕÿ ï£GŸ7üõ□ûèÐ□EEs¾|ßóÕÿ ï£[vDµœe‰$Ž§ë@□ÑE□ QE□ QE□ QE□ QE□ QE□ QE□ QE□ QE□ QE□ QE□ QE□ QE□ QE□ QE□ QE□ QEeê':N□]"mìqÞ€5(®w Ï›þz¿ýôhóæÿ ž¯ÿ }□ è¨®wÏ›þz¿ýôhóæÿ ž¯ÿ }□ è¨®wÏ›þz¿ýôkrÔ- µˆ'I*94□5□Q@□□Q@□□Q@□□Q@□□Q@□□Q@□□Q@□□Q@□□Q@□□Q@□□Q@□□Q@□□Q@□□Q@□□‰{,‹y Y□ z□>•□Ÿ7üõ□ûèÐ□EEs¾|ßóÕÿ ï£GŸ7üõ□ûèÐ□EEs¾|ßóÕÿ ï£GŸ7üõ□ûèÐ□EEs¾|ßóÕÿ ï£[ð'`Œ""´□* }□Q@□□Q@□□Q@□□Q@□□Q@□□Q@□□Q@□□Q@□□Q@□□Q@□□Q@□□Q@□□Q@□□W<óË æ7ï_©þ#@□□□Îùó□ÏWÿ ¾•-|ßóÕÿ ï£@□□□Îùó□ÏWÿ ¾•-|ßóÕÿ ï£@□□□Îùó□ÏWÿ ¾•-|ßó Õÿ ï£@□□□Îùó□ÏWÿ ¾•t

þ-~,€□E□P□E□P□E□P□E□P□E□P□E□P□E□P□E□P□E□P□E□P□E□P□E□P□E□P□E□P□E□P □E□P□E□P□E□P□E□P□E□P□E□P□E□P□E□P□E□P□E□P□E□P□E□P□E□P□E□P□E□P□E□P□E□P□E□P□E□ P□E□P□E□P□E□P□E□P□E□P□E□P□E□P□E□P□E□P□E□P□E□P□E□P□E□P□E□P□E□P□E□P□E□P□E□P□E □P□E□P□E□□â-;VÕ|=qc¢êcM½˜ª‹-¹(¹□±èqœåÖ€&□□hÉ®Ç¢6§ký© ,¶ž`ó□□=;p3ô-:ùþËÂ6~

ý |+§ÚO=ÃËc%ÅåÄí——
VYÃ1ôè8öîy¯ (¢Š(¢Š(¢Š(¢Š(¢Š(¢Š(¢Š(¢Š(¢Š(¢Š(□;•øö—
ýÃü«□®Šãþ=¥ÿ pÿ *çh ¢Š(¢Š(□£‹ýJ□º)ôÈ¿Ô§û¢Ÿ@□□Q@□□Q@□□Q@

šh-áy§•"Š5,îì□T

¤"ÐS-.í¯íc°³¸ŠâÞA°9apèãÔ□Á®KÅž□]lê□ÚÆ¡=ÖŸ□»5®˜□ÈQÂ}ù1Ì‡<Œð=□ ¿ä-
x□þ½□þ„Ô□Ðj^#Ð´iÒ□SZÓ¬euÞ±Ý]$LËœd□#ŒƒLÓüSáíZèZéÚö—
ypAa□µär9□©Â'k€ñ ðÑøÚßð"□'ì□ðŽ§—ý§åù{þÐÝ<Î7c>øÍvš□•àá'ö‡‡l4-ê
ý§N†□€z□È(□¡ª□ž¹¤h«□j°¥•€"'□°¸H·ã®7□œd~u~¼Ëâ□Ñ•Ä□□□o‹□ìß/Pó~ß³ÉÏ-
˜Ý¿åëŒg½ vv¾0ðÅõÔv¶~#Ò..%;cŠ□è•Üú □&¶«—
Ðô¯□ÜÜ-Þ□aá¹g€îY¬!□š3ê□□Eu□ QE□ QE□ QE□ QE□ QE□ QE□ UMKþ<›ê?□[ªš—
üy7Ô□: Ä¢Š(¢Š(¢Š(¢Š(¥O¾¿ZJTûëõ

–Š(□³µ□□FÒ$Xõ-
^ÂÉÛ•[›”ŒŸ b+F¸ôøuàÛ!yy¨i¶·³\;Mqw©âV$œýæá@éÆ8□□ÖÃ4W0¤ÐJ’Äã*èÁ•‡¨#-WÔ5M;
I„M©_ÚÙÄN□ÜÌ±©?V"¼ûá□□ÛËâ„Ò™ÛÃ_Ú'û,’J-?y°ž©œ {ãëU¼#¢iþ>×5ß□x,Ö=E□þK□:Þå
wÅ

□àd!ã,NN}=è□Òì5+□Rßí□}íµÜ9Ç™o*È¹ú,EZ¯*Õt[/ üHðÖ¥ À¶6:Ìí§_ÚCòÄìFc`½□□ÓÓÜ
×ªÐ□{ÝBËM·7□÷pZÀ

"È□GâN*□;ZÒµ…fÓ5;+åO¼m§Y@úí&¼ÓÄ¶úLß□à_□$o¤I§…Ò~Ö□Ñ¼íß8lü»þ½±íLÔôm
Ûâ—†ãð]-½¶§o3>¨4õ
□v¸äJ□å□ž ëúP□®QE□ QE□ QE□ QE□ QE□ QE□ QE□□›«ýØ¾§úV]jjÿ v/©þ•—
@□□Q@□□Q@□□Q@□□Q@□oXÿ Ç"_OëX5½cÿ -Q}?- X¢Š§«i±êúMÖ•4ÓÃ□Ìf7xc€zàö
íâm□oþÂÚÞš/7mû9»□ÌÏ¦Üç5©^oâŸ□ü:Ð¼□z·šN□g

vî#œ¨ó÷ãåÚçæfÎ02s[¿□àÔÛá®□□°óÇ|Ö›]˜‘"ƒ□¹'□Ávõï@□□~#Ðôû±i{¬éÖ×'¤3]"9ÿ €‟
šÒ□0□□Aä□\}¯ÂÏ□Ûi-dú□-×˜□™qt¾dîOV2˜□ìEfü□’Qá+û3<“ÙXê·6¶2HrMº‘·žü–
□C¨//m4ëWº¾º†ÖÝ>ü³È□□êO□§¯,ø™á□›Â~$Öõ□B}Fh¡‘¬ a²
5Ï□PpÏŽ®r}1@-£□‰,k$n¯□€ÊÊr□-„□ufxsþE}'þ¼¡ÿ Ð□iÐ□E□P□E□P□E□P□E□P□E□P□E□P□E□P
□E□P□E□P□E□P□E□P□E□P□Y:·úôÿ wúÖµdêßëÓýßë@□ôQE □QE □ÐZ□Ç¤_îŠçë ´ÿ •H¿Ý□□
5□Q@□□Q@□□Q@□VmFÅ5□ÓÚöÝo]

‰leQ#(êÁs’=ê=^Öö÷KžÛO¿û□Ô€*\ˆ„†>FHSÁ8Î3ßšó8|7aá¿Œþ□ŠĐÍ,Óé÷R\Ý\Èdšáø□••S
ì0□a@-›ªêÖ□&□&¡©ÝGki□□Ë!ÂŒ□□êEsŸð´ü□ÿ C5‡ýô□Â°É"ŽhÌr¢°-ªÃ
þ□□¯Í£øwÃ÷Ú½Ý•¯“i
JÃÊQ¸ŽŠ8êN úĐ□□□Äš7‰ –m□Q,ö8˜$□□ÎÓŒàÖ¥q¿

´9´•□¥Õê□ÔµY[P¼Âã□'!qØ*í□ìs]|ÑùÐI□÷MêWr□2äu□Ö€3îüG¡é÷bÒ÷YÓ-®OHfºDsÿ □'5
¤□`□ ƒÈ"¸û_…ž
¶ÓZÉô□[¯02ãé|ÉÜž¬d?0'ØŠÍø7$£ÂWöfy'²±Õnmld□ä›u#o=ù-
@–‡E□P□E□P□E□P□E□P□E□P□E□P□E□P□□÷ü~ËõÊ«Õ‹ïøý—
ê?•W □Š(□Š(□°(?ãÞ/÷□ò®v°(?ãÞ/÷□ò (¢Š (¢Š)²I□1´'°¤j2ÌÇ □RiÕ‰â?
éž+†ÖßVY¥µ,_4Û¬¥c"ã□xx

¸Í Z°ñ□‹ ªÌðéÚ½…ä©Ë%½ÊHËõ
N+F¼‹ âŸ…t?□øn×^ðþ ›m¦kVw•
7²ŒDdfp
0\nÈÉçž=3]□Å+Û¶Ó´□

ØÜ½´°ö¡□”³FpÉ□Y□>¸Àú□@□Bø›@{ï°®¹¦µæí¿g□q™3é·9Íj×□sðŸÁSèm¥¦ƒi□òö%Äq□:œpÞ
gÞ'¿&£øOªÞê~□H5)L×°mÌ¶□JÇ%Ìg Ÿ}¤□Â€;ŠÏÔuí-
Ge]OV±²fåEÍÊFOÓq□OÆ:÷ü#-□Õu□¡žÖ□Ñ«t.xP}·□\N‡á¯□i6‘IâëÍ□ÿ ÄwŠ%½—
Vž'•ÈÃ%U\ü t□□¨□Ó-ní¯-ÒâÒâ+ˆîÉ□‡Vú□Á©«7DÑô□□ÀÃ¢ÚÁmi+™¶A÷
`9□¹ tâ´¨ ¢Š(¢Š(¢Š(¢Š(¢Š(¢Š(®mÿ Ö7Ô×I\Ûÿ ¬o© □ÑE□ QE□ Q
E□ WIúµú
æë¤•ýZý□ :Š(□Š(
×Ú□Ž™oö□BòÞÒ□ãÌ¸•c_Ì□),5;□Vßí□uõµä9Ç™o*È¹ú©"±µ/□è□Ö¿ý³«Ùÿ hN±□¢Šèù•Ä□s¶
3ÆNy'=+Žm□ÃÂß□t□|;□YCªY\□FÒ□¶-¨¹•ö

ÝÇ-žç -©TÎ-¦PigP´ƒ.ñhf_4¯®Ìç-øª~'ñ·…ü?sªÜ,þPÛ+÷¦ð^¾äà~µåúNu¤|Sð…
ö¬ÂMsUŠúëPqÑXÆ»cì¢áGã@-ÑE—¯é—°¾œ,ìµi´Íò:x

|åPŸ¸O78
qê63_Íc□í¼—•¨imÖU2 =
.r□Ö¬×-øGD°ðÿ ÆMoOÓ¡1@°D

w1fv/’ÌÇ’Äõ&½J€
(¢€
(¢€
(¢€
(¢€
(¢€
(¢€
(¢€
(¢€
(¢€
(¢€
(¢€
(¢€
(¢€
(¢€
(¢€
(¢€
(¢€
(¢€
(¢€
(¢€
(¢€
(¢€
(¢€
(¢€
(¢€
(¢€
(¢€
(¢€
(¢€
(¢€
(¢€
(¢€
(¢€
(¢€
(¢€
(¢€
(¢€
(¢€
(¢€
(¢€
(¢€
(¢€
(¢€
(¢€
(¢€
(¬□□iú®©áë›=□SþÍ¿“o—u·vÌ0'ó□• yÇ‰?äç<'ÿ `·þW□ëõáÓü-
ñÝÎ½o®Mã^ŸS¶□Ê†äÄÛ‘~n□ýôß□zŸ„4½kGÐ–×¯Ö?µoDŒßhÙ·
z/¾9çÞ€7¨¢Š (¢Š (¢Š (¢Š (¢Š (¢Š (¢Š ¯(¢Š (¢Š (¢Š Žãþ=¥ÿ pÿ *çk¢¸ÿ •i□Ü?Ê
¹Ú (¢Š (¢Š èâÿ RŸîŠ}2/õ)þè§Ð□E□P□E□P□E□P□□oþ@:•ýzËÿ šæ¾□□É,ðÿ ý{ý
©Þ%ðÏ‹5›ëƒ¦xÛû/Nš?,Ùÿ eE>8Ã|ìAçôª~□ð?‰¼*Ö□¯ão¶hÖŠWì-
ÙQÇ¹H8-fâÃ□çðÅ u□—†4□fénµM□O¾□SËY.m’F
’q–□Œ“ù×□ø›ÃÚo,üqá□SÃVé§Üj□ŠØÝZÛ|±Ï
□˜”□|½x□Ç ®›Xð—
‰nu‹›ý□ÇWÚbNA6ÒÚGu□`□ò□ÆÑÆ~¤Òè^□6:âkÚî·w¯jñ!H&¸UŽ8□ëåÆ¼)=Í vU•ªø□F×

'VÒ¬¯üŒù_j□dÙœg□□Æp?!Tõ}
ÿ Qñ□•¨Ûk·VVÖ

í=œ@ì»

□□r:c¸={U□x_\ÔõUÔ4_□_hïå□Ú□□\BpIÝ±¸□žO°

7âo…ôo   é□¾)ðÝŒ:^³gy
Ãö5ò-pÌ□Œ à,3Û±¯]®□ÃáåÌÚÅ¦©â¯□]ø,{'ó-
axR□#~Ïå¯□‡c]Õ □QE □QE □QE □QE □QE □QE □SRÿ •&ú•çVê¦¥ÿ -Mõ-
Î€1(¢Š (¢Š (¢Š (¢Š)Sï¯Ö’•>úýh□¥¢Š(®?Ä?
ü!â□J]KUÒÚ[éUU§[™Pà

□mè=+¯ □AèkÏ!øsâ□!M·‡|□©XißÁmuk□ß-
?°Œä□-” ß□Ï□ øëVðD·ój□m¥¤wvRÎCK□± ÄÍß®G°¥ø8Â

êúkœ\Xk7PÊ‡¨;•□õý+ ðŸƒ-<,/.>×s¨jwÌ-îþé³$¤t-Ê;
ÌÖ>-ÜKâ+•□Ã^"¹Ð5
°□ß—□Ï

ätc□`n÷ÿ □•
□□˜]ø³ÀZ\g7□¬
Í£¨HW,~œ×¢W!á¿□•Y—^Õµ{□o['?$]Ü(EŠ?îÆƒ…□¿ÿ \çIô+÷ñ¬Zà×n–Á-

□K ùLù'Ì<ã<úgŽ½¨□'Ô£"âWŠµ¯□\Oöo□hí□]G□©-
îV□€ÜAØƒ□ã"ëéBûHoƒóÚj:□Ã¿‡¯o£,óKŸç(_•$O÷²1÷I9þ]-¹ðúK¿□Ëâ□□ë÷z

«pŠ–/

k,S…□□ãn □ŒÔ□Ÿ

ïnõk=CÅž)»×ÍŒ¢k{sn-
Ð,ƒ£□O¼Gj ïh¢Š (¢Š (¢Š (¢Š (¢Š (¢Š (¢Š ÍÕþì_Sý+.µ5□»□Ôÿ JË □Š(□Š(□Š(
□Š(□·¬□âÊ/§õ¬□Þ±ÿ •(¾ŸÖ€,QEfx‡C·ñ&‰q¥]Mq

sm"[y6HŒ¬□YOb□□€8û¿,Þ

wk•:ÚëKÔ□□#¼µ»—z?\€ÌGZÐøaâ□GÄ-□y5Y□{Ë;¹lžá□□û□ÃàqÈ?¥g-
x¶H□”ß□µ&Ó^ÚÈ¶Q¬å}<ìîÏ½uZo†ít/
ÿ `èŽÖ1¤□□3c{#°?¼9ûÇ'4□•âÍ~÷U¿□□x^Lê’®/¯G)§Bz±?óÐ•º½{ñ]>•¡ÙxoB´Ò4èöZÚÇ±
sÕ□RÇÜ’I÷5çú/Ã?□xzÑí´¿^¢□ä‘¥□□

□y□ŽK3³□cîI®ÏÃ:O^t±uý½â□ıÃ&ß$ý,;o'□Ý÷ ÝœŽ½1ï@□õÈüRÿ ’aâ-
úóoæ+GÃº□þ‹>«%î»uª-íÛO
N□□ÈzF¼ž□¶□°®oÄþ□ñg‰□£h|yö}&ð•û□öDO²3ü>fàÇëÖ€:ï

□È¯¤ÿ ×”?ú -:ä|)á¯□h3¢jž0þÖÓâ·òb´þÌŽ□„ckoRIÀ□`ú×]@□□Q@□□Q@□□Q@□□Q@□□Q@□
□Q@□□Q@□□Q@□□Q@□□Q@□□Q@□dêßëÓýßëZÕ“«□¯O÷□+ gÑE□ QE□ WAiÿ -‘□P+Ÿ®,Óþ="ÿ
tP□ÔQE □QE □QE □çÚ×ü—
ÿ Ø6ê»M^ÚúóJ¸·Óu□ìëÇ\Euä¬ÐQÏ]□Áã#ŸZóÉþ□xÊã\µÖ¥ø□»Pµ‰â†oì8FÔo¼6îÁÏ¸ □P¯;
ñÁÿ „£Æ:□,cù-C□iê tòc?"f□ä□vz□ž¥a¤Cm«j¿Ú—
ª[Ì»û:Á¿$‘ò/□□□ð¬Ý□Â¿Ù- ×u»›Ï¶^ê³©□åì□Âƒ □äç□©ã<q@□□□×RÈÊ□©
€Ã¨÷®{Aðî©£øN]&ãÄwZ…û‰vjS¦]
giÁc□¾„þT□“âÍ~÷U¿□□x^Lê’®/¯G)§Bz±?óÐ•°½{ñ]>•¡ÙxoB´Ò4èöZÚÇ±sÕ•RÇÜ’I÷5çú/
Ã?□xzÑí´¿^¢□ä‘¥••

□y□ŽK3³□cîI®ÏÃ:O^t±uý½â□iÃ&ß$ý,;o'□Ý÷
ÝœŽ½1ï@□ôQE □QE □QE □QE □QE □QE □QE `ß□Çì¿Qüª½X¾ÿ •Ù~£ùUz (¢Š
(¢Š +¢ƒþ=âÿ p□*çk¢ƒþ=âÿ p□* 'Š(□Š(□¡°°·±´–
êêd†Þ□/$'6□Tu$ÔÕÇü@ð]ï•¬¬--µÓ¦Co7□,fÔN"'□¡"°□□
ÁÈ9éÅ eé•\|Dñ□Ÿˆ¯!'□é¯æiVò©V»—þ~□-Š?€-{ýSâc
?□x□R"í·‡Y□»žŠd\□□#R/„>!*…_‰À(□ h□ø-tš·…í¼Gá?ì-zV¼ß
,·(¢&i□□Þ(□)ÈÎ:sŽE n3*)f!T

’N □ç¿□37…u=IAòu-fîî□ê…,ƒù©¨ßá¿ˆ®ìÿ ²õˆz□ÎŒFÇ·[XÒWOî´Ù,r89ë]„°

pøRM□H™´¸Å©··š

Z□—□‡ ’:õÎ{Ð□9ñ’ .~□kÉ□%„q9Ç¢ÊŒ□@jm□áï□¤Ð-ž□

éÓÃs
È%š!+¸`

w¶[œõÍoi°!ƒÂÑhš-Üš¯ú9·¸žà□× , nIäSõ®6Ûá®¿¥[□7Fø□¨ÙhÃ";V´ŽY#Sü+)9_l

(□>□Gý•yâÝ
ÖG}+MÕ□;0Ì[Ë□e£□öS□ÄŸZôšÇðÏ†tï è©¥é¨þPc$’JÛ¤-C÷□Ïrkb€
(¢€
(¢€
(¢€
(¢€
(¢€
(¢€
æßýc}Mt•Í¿úÆúš m□Q@□□Q@□□Q@□t‘ÿ «_ ®n°Hÿ Õ¯ÐP□¨¢Š (¢Š Çñ'‰4ÿ
éM}|ÌÅ˜G□□□ÒÜH~ê"÷cX¾□ðõúê□ž+ñ□¨×u□□°)ÊÙ[ŽV□=Ïv=ÏëCÄ□□µÍcÆ‹âM?Åÿ ÙòC□ŠÚ
□4Ô¹□ã□q]í€Xóœgœg□kOðÇŽ-õ□i¯>"ý®Ö9U¦·þÄ,?5□åw□•Èã#¥ s¾*Öï□Äè□Qð·‰5□-
FE’ÈiŰyš9®YA2±$□•□□Î

O□‶-xÿ í?□|/©□Â#â¸¾É□Úý–
]7lón@3□nù€ïÏ□íõ•¨ødj-2Ñ|Cö³□Ó"ž?#ËÏ™æ¨□Ýž1□CŸj ×±°ûvŸmwäOoçÄ²y7
²H÷

íeìÃ8#Ö¬V□‰ô+ýv
□ì5Û-%-îÒy-Ü□fEÎc8#ƒŸqÇCV<Ca«j:g‘¢ë_Ø÷{Ã}§ì©qòó•ÚÇ□ñÏµ rz_ü—
}{þÀöÿ ú□z□y|_□¼e

½q-Çñn£q
Á,ßØp•È§ mÝ•õ□5èú|76Úu´□·□1°Ž%Yn|±šàrÛG
‟Î□J ³E□P□E□P□E□P□E□P□E□P□E□P□E□P□E□P□E□P□E□P□E□P□E□P□E□P□E□P□E□P□E□P□E
□P□E□P□E□P□E□P□E□P□E□P□E□P□E□P□E□P□E□P□E□P□E□P□E□P□E□P□E□P□E□P□E□P□
E□P□E□P□E□P□E□P□E□P□E□P□E□P□E□P□E□P□E□P□E□P□E□P□E□P□E□P□E□P□E□P□E□P
□E□P□E□P□E□P□E□P□E□P□&RðÈƒ«)□²□²î?ØüëfŠ ÆþË¸ÿ cóƒû.ãý•Î¶h

oì»□ö?:?²î?ØüëfŠ ljV5SÔ)ÔQ@□□Q@□□Q@□□Q@□□Q@□□Q@□□Q@□□Q@□□Q@□□Q@□□Q@□□Q
@□□Q@□□Q@□Ay
OncLd'Ö§¢€1¿²î?ØüèþË¸ÿ có-š(□□û.ãý□Î□ì»□ö?:Ù¢€1¿²î?ØüèþË¸ÿ có-š(□□û.ãý•
Î□ì»□ö?:Ù¢€1¿²î?ØüéWL¸

□ÉÁõ-Š(¢Š(¢Š(¢Š(¢Š(¢Š(¢Š(¢Š(¢Š(¢Š(¢Š(¢Š(¢Š(□•ý´—
"1->\ç&©□eÜ□±ùÖÍ□□•ý—qþÇçGö]Çû•lÑ@□ßÙwì~t□eÜ□±ùÖÍ□□•ý—qþÇçGö]Çû-
•lÑ@□ßÙwì~t□eÜ□±ùÖÍ□□•ý—qþÇçZ-Ñ´VÉ□c sŠ-
Š (¢Š (¢Š (¢Š (¢Š (¢Š (¢Š (¢Š (¢Š (¢Š (¢Š (¢Š (¢Š (¢Š (¢Š (¢Š (¢Š *…õœ·
□«&Ü□Ç&¯Ñ@□ßÙwì~t□eÜ□±ùÖÍ□□•ý—qþÇçGö]Çû•lÑ@□ßÙw-
ì~u«□□àDnª0qRQ@□□Q@□□Q@□□Q@□□Q@□□Q@□□Q@□□Q@□□Q@□□Q@□□Q@□□Q@□□Q@□□Q@□□Q@
□wV□Mrò.Ý¤ñ‟Q□eÜ□±ùÖÍ□□•ý—qþÇçGö]Çû•lÑ@□ßÙwì~t□eÜ□±ùÖÍ□□•ý—
qþÇçZÑ)H‛OP □}□ QE□ QE□ QE□ QE□ QE□ QE□ QE□ QE□ QE□ QE□ QE□ QE□ QE□ V;i
—□É□9>µ±E c□eÜ□±ùÑý—qþÇç[4P□7ö]Çû•Ùwì~u³E c□eÜ□±ùÑý—qþÇç[4P□7ö]Çû-
•l(Â(=…-
□ QE□ QE□ QE□ QE□ QE□ QE□ QE□ QE□ QE□ QE□ QE□ QE□ QE□ QE□ QE□ QE□ QE□ Q
E□ QE□ QE□ QE□ QE□ QE□ QE□ QE□ QE□ QE□ QE□ QE□ QE□ QE□ QE□ QE□ QE□ QE□
QE□ QE□ QE□ QE□ QE□ QE□ QE□ QE□ QE□ QE□ QE□ QE□ QE□ QE□ QE□ QE□ QE□ QE□
QE□ QE□ QE□ QE□ QE□ QE□ QE□ QE□ QE□ QE□ ×`ˆÎz(ÉªŸÚ-
ÿ íþUfãþ=¥ÿ pÿ *çh□gûRßý¿ÊŸ□ü3J±®íÇ¦EaÕ›□øþ□ñþF€7h¢Š (¢Š (¢Š (¢Š (¢Š (¢
Š (¢Š (¢Š (¢Š (¢Š (¢Š (¢Š (¢Š (¢Š *9¦X#2>p=*J©©□Ç‟}Gó □ÿ j[ÿ ·ùSâ¿†
i□5Ý¸ôÈ¬:³aÿ ±}Oò □Ú(¢€
(¢€
(¢€
(¢€
(¢€
(¢€
(¢€
(¢€
(¢€
(¢€
(¢€
(¢€
(¢€
(¢€
(¢€!¸¹ŽØ)‟?7L
ƒûRßý¿Ê¢Õþì_Sý+.€6□µ-
ÿ Ûü¨þÔ·ÿ oò¬j(□gûRßý¿Ê®ƒ‛šæk¥_°>” ´QE □QE □QE □QE □QE □QE □QE □QE □QE
□QE □QE □QE □QE □QE □QE □QE □QE □QE □QE □^{È-Ø+îÉ□àUŠÉÕ¿×§û¿Ö€,ÿ j[ÿ ·ù
Qý©oþßåXÔP□E

Ë<bDÎ□-IU4ßøò_©þun€
(¢€
(¢€
(¢€
(¢€
(¢€
(¢€
(¢€
(¢€
(¢€
(¢€
(¢€
(¢€
(¢€
(¢€
(¢€*Ë□

24m»#®3ûRßý¿Ê³¯¿ãö_¨þU^€6µ-ÿ Ûü¨þÔ·ÿ oò¬j(z
È®ªnÈäUŠÈÒáÿ Üþ¢µè ¢Š(¢Š(¢Š(¢Š(¢Š(¢Š(¢Š(¢Š(¢Š(¢Š(¢Š(¢Š(
¢Š(¢Š(ªgS·ƒ¿•j¹\Ûÿ ¬o© iKöÿ *?µ-
ÿ Ûü«Š ÙþÔ·ÿ oò«QJ³D$\í=3\ånéÿ ñã-
ãüÍ Y¢Š(¢Š(¢Š(¢Š(¢Š(¢Š(¢Š(¢Š(¢Š(¢Š(¢Š(¢Š(¢Š(¢Š(¢Š(¢Š(¢Š(¢Š
(¢Š(¢Š(¢Š(¢Š(¢Š(¢Š(¢Š(¢Š(¢Š(¢Š(¢Š(¢Š(¢Š(¢Š(¢Š(¢Š(¢Š(¢
Š(¢Š(¢Š(¢Š(¢Š(¢Š(¢Š(¢Š(¢Š(¢Š(¢Š(¢Š(¢Š(¢Š(¢Š(¢Š(¢Š(¢Š(
¢Š(®wÆ¾0³ðNý©w×åX ,–'FžÃ
yöïÒº*kÆ'mÞŠÛNåÜ3ƒê=èÇ&øÉâm G¯øöËH'€3nmÑƒë¹@Ï ;s^»a}o©éÖ×ör-
®bYbp1¹XdÖ¼óã†µoeà ôŒy·ú³¤Ð(ÜÍ‡V'-ØêEuÞ

ÒfÐ¼□£éw?ëí-Q%□Î□ažø$Š Ý¢Š(¢Š(¢Š(¢Š(¢Š(¢Š(¢Š(¢Š(¢Š(¢Š(□;•øö—
ýÃü«□®Šãþ=¥ÿ pÿ *çh «6ñýãü•V«6ñýãü• nÑE□ QE□ QE□ QE□ QE□ W#â_□•-
Y‹BÒtk-oZ’/<ÚÛ°EŠ<à3»p¹?ç‘ž°¼¿ÆóÏà_□'□,žÚéo [+-.I6M>Óò´□w0ã+•çÀ□®—ñ

óþ□
=□Äþ□°Ðn¯‰[I□t¸†V-
Ã½@□½¿ÄWw^O¥kQüMñµ“\ªéVú□æâ=2èí½šlpì‡î¢ç±<õë^±@□Þ&ñF›á=+íú‹HC8Š□a]òÏ!è^
½É®f/□x¹¤Ži¾□ê1Ø9-
¼[èš`□s□_Ã5•ãmmí>1hP□dÐêÍe¥Éukih□□□#”ÜÙá@U?1éÅhÜüKÖt3□ÏŠ|□{¤éNá□ö;ÈîDY8
□Õ□Tgüš ōz)□ÕÑ]□2°È ä□K@□□Q@□□Q@□□Q@□□Q@□□Q@□□Q@□TÔ¿ãÉ¾£ùÕ°©©□Ç“}Gó

J³aÿ -
±}Oòªõfãþ?búŸå@□´QE □QE □QE □QE □QE □QE q°ï□¾Ã–¾…¡h·zö±□‡ž□vXã€–žd□Â“ØU
Ï□x‡]Õo&µÖü'u¢°Gæ$†å."~q•ëÑ¹Î>µæß

<[¬ÿ Â?sw£x2ÿ X¾Ô/g»»°y'Ú□bä□Y-
ïa@□□Žk¿ð¿Žÿ ¶õ«•□UÑ®´Mn□¼ÿ ²ÎáÖHó□Èã†□ÿ '□ □…qZ¯Žu%Ö.´¿

xNÿ [šÑ¶\Le[h□°

Ñ#ýâ22 ®ÖŠ å<%ã^¼Mw}¦]i·:N³aƒscrA!OFV□2ûû□Q]]yÏ†Xxƒâî¿â;A□:ÊÉt„˜}Ùå

□ÊžûHÆ~•èÔ QE□ QE□ QE□ QE□ QE□ QE□ QE□□›«ýØ¾§úV]jjÿ v/©þ•—
@□□Q@□t«÷GÒ¹ªéWî•¥ -□Q@□□Q@□□Q@□6I□(ÚI□Q□□fc€ êI§TsÁ□Í¼-
óÆ’Ã*”’7□VR0A□Á□□ÂËñJÆ_□éZ~—§_XßÝý“ûO>\□ñÈŒ‘ûÌc’0=ÍwsJ-ðI4-¶8Ô»□g

šóïÃ□¿‰>Ã

i□I«…DE□T□8

,½□€<ïþ□/ˆo!7Ú7ÃÝRóKê·□\$□H¿ÞXˆ,Àöõ®«Â¾'°ñ~•

¯§y,) VŽQ‡•ÁÁV–¢¹□□|P‶A′ö□ü!â)šÙÙ□Ô–e–[Åæ

•½ò□®|+°µ³ðZMm©[j/}q%åÄö¹òü×9ePy□À□ -:
í«•×|}ö□mô-
E»×µˆ”<ðÛ²Ç□ ôó$n□žÂ»*ñ□‡--Ö□á¹»Ñ¼□□¬_j□³ÝÝÝ<Ém
1r□¬•÷°

□Ç4□é>□ñ

»ªÞMk-øNëEt•ÌI□Ê\Düã□×£sœ}k¦®?Âþ;þÛÖ®4□WF°Ñ5¸"óþË;‡Y#Î7#Ž□güœ□v□ QE□ QE
□ QE□ QE□ QE□ QE□ QE□ QE□ QE□ QE□ QE□ VN-þ½?Ýþµ-Y:·úôÿ wúÐ□}□Q@□zoüy/Ôÿ
:·U4ßøò_©þun€
(¢€
(¢€
(¢€
(¢€
«©j6°F™s¨ßJ"µ¶□¥•Ïe□'ëô«U•âm□□xkQÑ§”Å□Ü-˜¼”=•¾

(□□□â/‰¯mWQÓ>□jW:S®øç{Èã•Ó¨a□ 9□Œ-k®ð¿‰¬<[¡C«iÞ`‰ÉGŽUÚñ88eaê+Ë->-
ø{Gm□ëN,úæÁ'É5‹iKiäã□¤p2¤q•□Î□Jô è0è-□Š8ïâ¿'îW¼žî□<¹d□ä"Çðô□é@□=p·ÿ □
.&Õî´¿
ønïÄ□Ù¿—s,s,□ñ¿tó□,Ã¸®›Ä·òi^□Ö5□Ž$´²štúª□-
Ê¼ÃÀ^%×tÿ □iV¾-ð□¡¨F°‰'°¸°ŽÔM#|ÎË¿%ÆIÁï@-‹á□sTÖ"¹□¿‡nt[˜□FÉeYR@sÊ°ðqŽ~¢
·«—ð‡•`ñS_ZIas¦j°{□»±¹ÆèóÈ
•¼§×üFzŠ (¢Š (¢Š (¢Š (¢Š (¢Š (¢Š (¢Š Á¾ÿ •Ù~£ùUz±}ÿ -
²ýGòªô QE□□□Iÿ •‡ÿ súŠ×¬•'þ>ýÏê+^€
(¢€
(¢€
(¢€
£«k□vƒ§I¨j—qZÚÇ÷¤□ãžÀ

¤žÀrjõAsgkyå}ªÞ)¼™±y^cŒ€Ã=ÉçÞ€9O

øûþ□_□ßhãE»±ŽÐÕ.¢−èíyQŽ□òñ•□¨ÉÎ;
Ýñ□½oá½!õ
ˆ.n eŽ8-
c2I+±Âª□Rk‟Òÿ ä»ëßö□·ÿ Ðë»»Ÿì¶sÜyRÍåFÏåB»□ð3…□Éè□ yíßÄÍsF‰u□□Àz†Ÿ£□□ï□î
9š N□xÔezŽ¦½□   ¢¹·Žx\<R¨tuèÊFA□ã¾5øˆuí!ü<ú&©áë}O□K©kÖ•

Q!#8□vXŽ™ □Zõ½*Ö
□"ÊÒÕüËx Ž(Ÿ9ÜŠ □žü
 MWS¶Ñ´«-Jð¸·¶ŒÈû□³□; :šá.~$x†ÖÑµI~□ê©¢ Þ÷□s□□S»□>ðÀç"^□Xž/×m|7á]CS»
¬q□Ž>òÈF□ îI P□í#V³×t‹]SO—Í´°ŒI□ã□-
QØŽ„{UÚå~□èw>□øw¢éwŠVæ(KÊ‡ª3³9Sî7cð®ª€
(¢€
(¢€
(¢€
(¢€
(¢€
(¢€
æßýc}Mt•Í¿úÆúš m□Q@□néÿ ñãäüÍaVîŸÿ -1þ?ÌÐ□š(¢€
(¢€
(¢€
(¢€0üCâ½3Ã,□®ZIï®8¶°¶O2yÏû*;{œ□ëUü□â¶ñ†‡&¢ú{X<wR[4□(□,‡□•□ùîkwìv¿nûoÙâû
_—åyû□ý™ÎÝÝq'N+…ø=ÿ "¶¥ÿ a‹¿ý

€7<OâÖĐnml,´{í_T°Vx--T □□
;ž□dã5“¥üC»-"´ĐüOá«-□îû"ÍÞá.!™‡ðï\ ÞÞãÔg»¯9ñ»□□Çþ□ðõ˜ó'°½]^ñ×¤□G÷C-Û‰Æ>
”□èÕŸ-ëz□‡t{□WS¸□Z[®çsÉô

äœ +B¼¿âÆ¨¶¾
ð]"öWWöÒ^Ëtöv±ù'Lð (¡{□Ï"ž03@□"ÇÞ+»Œ^X|8Ô¥Ó˜nYf½Š□ŠúùGŸÃ5ÞZÎnlà¸hd„Ë□¹Š
Q‡LŒíaê:□óûÏˆ¾%ÒmÛPÕ¾-j□ÚLct·□ÞÅ,'§v1

F;ä×{§j□°®›m¨YJ%µ¹□e‰Çñ)□□□fŠ(□Š(□Š(□Š(□Š(□Š(□Š(□Š(□Š(□Š(□Š(
□Š(□Š(□Š(□Š(□Š(□Š(□Š(□Š(□Š(□Š(□Š(□Š(□Š(□Š(□Š(□Š(□Š(□Š(
□Š(□Š(□Š(□Š(□Š(□Š(□Š(□Š(□Š(□Š(□Š(□Š(□Š(□Š(□Š(□Š(□¹¿□xÓ
Oð7‡ŸT¾V‘Ù¼»x□¬²□H□ì8É=½Î□é+7Zðþ‘â;TµÖ4ø/aGÞ©*çkc□• x□,µÏ

j^ >8ñÇ‰´÷Ö□þ<¬w□-iÛ• _AÛ99cÇ¹i:Æ•®Ø-ö—
y□Ý«’¢X›##,+žÿ …Yàoú□l?ï“þ5Ðé:>•¡X-†—g□¥ª□Â(†□O$Ð□ê(¢€
(¢€
(¢€
(¢€
(¢€
(¢€
(¢€
(¢€
(¢€
(¢€#¸ÿ •i□Ú?Ê¹Úè®?ãÚ_÷□ò®v€
³aÿ Ñþ?ÈÕj³aÿ -
Ñþ?ÈÐ□í□Q@□□Q@□□Q@□□Q@□□Q@□y=¦·¥h□□üE/‹gŽÒæHá□EÕßË□·Úw*1áNãÏ©ÍzÅT¿ÒôýV□

£cky□9 q
È¿"□@-YãýoAñ□¯áØ</ym□âxõ□š ì□Hb„□æouãf:,□+zV¡â='JÕ´í.öña½ÔY-Ö"¬|Â¸Ï
`u□qRéÚ&"£†□f—ed□ï
kt•?] TÓéÖWWV÷W□vó\[□`šH•ž"zí$esí@-yã;Èü%ñCAñeðdÒn,äÒî®□' %·¡lv$þ†¢ø•ã=
[ð•Ï‡t+ëmgVÕ,ÛÛ[YH%Á,
f+• □ž□Ç-›=¼7P<□□Ç4.0ñÈ¡•‡¡□-RÓ´□□Gv}3I°²g□f¶¶HË}v•@□iVm§é□VL
ûÚÞ□â-ýâª□□J·E□ QE□ QE□ QE□ QE□ QE□ QE□ UMKþ<›ê?□[ªš—üy7Ô□: Ä«6-
ñû□Ôÿ *-Vl?ãö/©þT□»E□P□E□P□E□P□E□P□E□P□E□P□"|9ñ&"àÛ=CÁ¾ ¿·Ó.ôËÉ|"w
‰&□Ø²º³`□äñô«Zn¡oãOŒVúÆŠLúV‹a%¼·Ê□—4Î~âŸâÀ9Ïÿ [>□¨èZF°PêzU□ñO¹ö›t—
oÓp8«VÖ¶öVéok□P@ƒ
□H□Wè□□€(Zx□I¿×o´KkÅ"Q°Uk˜□°(□drF□QÐœW□âß□ÚjÚìþ□°ñ□Ž•

_.©©Ü\¤M□ï□!ˆÌ‡•[¢ýkÑâÓ¬ ½žö□;xîî □N‘(’@8□˜

œvÍfÜø7Â÷—2\ÝxoGžâV/$²ØÄÌìz’Jäš «á-WÁÖ–¶z□‡µ□"E‰
Ákmy□ŽÀ□IÀ$“Ô“õ5ÒÖ=□„ü9¦]¥ÞŸáý*Òå3²k{8ãuÈÁÃ □à‘[□ QE□ QE□ QE□ QE□ QE□ Q
E□ QE□□›«ýØ¾§úV]jjÿ v/©þ•—@□□Q@□t«÷GÒ¹ªéWî•¥ –□Q@□□Q@□□Q@□2Yc,'-
WXãE,îç□@ä’{
}2X£ž'ŠXÖHÝJº8È`x Žâ€<§Çž,ðåç‰<□-¯ˆ4©ã·Õ|ÉÞ+ÈØD»OÌÄ-□¹¯Cÿ „—I—
D½Õì/!Ôílãw“û>Eœ’«¸¨Úq»□³ÜU_øA<ÿ B¦‡ÿ ,è□øšÔÓt□3F□ ÒôëK□]·´v°,J[□É
 Î çÚ€9ÛOŠ-   ¼ÓþØž%ÕãLdÇ4¢9□¶ÆÃgè+□á<bâçÅ:Ý•»Ûèšž£æØ#!@à

4Š½ƒ□~^ÕÙÏáo□]]›»•□KšäœùÒYÆÏŸ]ÄfµUB¨U (□ t□□™£ø□I×¥¾□L¼[‡°œÛÜ€¬<¹□QÈ□úŒ
Šó¯‡>$Ò|□g¨x7Ä□öúeÞ™y/’nä□$Ð;□WVl□œž>•ê□šu•ƒNÖvvöíq!-c

J†G=Y°9>æ¡Ôt-#X(u=*Æø§ÜûM°K·é¸□P□Ÿé°…¿•>1[ë□)3éZ-„-
òß(>\Ó9ûŠ□‹ ç?ýlú}Emkoen-
ö°E□□0±Ä□U~€p*Z (¢Š (¢Š (¢Š (¢Š (¢Š (¢Š (¢Š (¢Š (¢Š (¢Š (¢Š +'Vÿ ^Ÿîÿ Z
Ö¬•[ýz□»ýh□>Š(□½7þ<—ê□□[ªšoüy/Ôÿ :·@□□Q@□□Q@□□Q@□□Q@□q-
□Æ¤~□jƒMY™¾O=`ûæ□ÃÌ□þ□œûf»z(□ˆÓ<sðí<9

6šÖ□o§,;E¤’"□\r¦3ÏÔcšÌøEµtÿ □ÝÙ$□xr]JI4´‘J□□>f@z!=□±÷®ÊO
xnk£u/‡ô§¸'&V²Œ¾}sŒÖ¸ET□□□□□@à□J ç—
PÒü{àÍ@h÷«qi{□Ö,]Œ 1R§† ÷ô®Káß□t+□□ÚhÚî§k¥jºJ}ŽæÚöQ□□œ□7cv@□+Ñ¬tû-
2ÔZéövö–êI□[Ä#@O$à *-ÿ ‡t=Rán5□□N»□F□·□©#□Ä,h□ˆð<ëâ_ˆÞ#ñuŒn4y ŠÆÚvR¢é“-
p□`F3ÿ ×Ç¥Sc□!□c‰□#Q…U□ z□N □Š(□Š(□Š(□Š(□Š(□Š(□Š(

□ïøý—
ê?•W«□ßñû/Ô□*¯@□□Q@□ôŸøø□=?¨-zÈÒ□áý Üþ¢µè ¢Š(¢Š(¢Š(ªš†©§é□¿iÔ¯-¬-÷□
ónfXÓ' Ë□3Vê¦¡¥éú½¯Ùµ+□kÛ}Á¼«˜VDÈèpÀŒÐ□˜éÞ,ðâ|hÖ¯ßÄ□RÙÉ¥A□\□ÈÄlÁ¹PÙÁ#Ò»
-{ÆZ7‡t›=Zöá›M°•c[»uócPÊJ¹+Ÿ”ã□□ê)?á□ð□ý
šþ
¡ÿ âkf;;Xl-Ê+hRÕ□F°* Š€`(^˜Çj â<Wñ
Á□øJþ7Ö4ýH\Û´ig□«,’³

*ì□ ç□œb®ø!eð§Â½1¼A#[›□#%Á•□bA–Á□œªàcÛ□¹iá•□ÂëíVz–™ms×Î†Ò4□Ì

ÖŒðCuo%½ÄI42©I#‘C+©à,[illegible]P[illegible]Tž*Ñcð©ñ1½S¤[illegible]|áq´Œ¯AÁ[illegible]É<c[illegible]Íyæ‘â[illegible]Å°Õ¿‰¼Oâ-
[illegible]ÖÖÕ·éZ<š„Y„öša»™}[illegible]ø~µê[illegible]¤é²i[illegible]Ù o§Ú6Ÿ´'Ù[illegible]1m[illegible]Ìc[illegible]tÅeÿ Â
àÿ ú[illegible]4?ü[illegible]Cÿ ÄÐ[illegible]¦•«iÚÅ»\iz…-ô
Å

¶³,ª q•$g qïW*ž ¤éÚ=³[éz}-Œ

ÅÌV°¬JX€3… g síW(¢Š(¢Š(¢Š(¢Š(¢Š(¢Š(®mÿ Ö7Ô×I\Ûÿ ¬o© □ÑE□ VîŸÿ -1
þ?ÌÖ□néÿ ñã-
ãüÍ Y¢Š(¢Š(¢Š(¢Š(□3Rñ□‡£J‘j°ÎŸc#®äK«¤ˆ°é□□ŒŠóÏ„ž%Ðmô+Ë9µ½6;©õ{‟

v•ä

ãnÕ''=±Ö½□Rðî‡¬Ì’êš6□}".Ô{«T”¨ë€X□
-□,ü+m<sÁá□□)£`ñÉ□„JÈÀä□Bä□{Ð□7□<{oáQm¦[Ëiýµ~1n.æ□C
ô2Êä€□`ñÕˆÀª~□Ô<□á»YžO□h·Úµëù·×Òj□ïžOûë…□□G W]©xsCÖfIµM□N¾•□b½ÕªJÊ½p
□•ÍR-□ð€9-□Ðóÿ `è¿øš ¹-ø•IðäV²ê×‹l—S-¼$«6é□ à□têx®;â□“HÔ|-âï*I-tk×□~Z-)

Ê□Ÿ-Ø˜®òóN²ÔV%¾³·¹X¤□Æ'‰\#ŽŒ28#Ö¬2«£#¨ea,□È"€8□|Jð ð-à²Õ-5;«¸□□k+Y□²Êî
ª”□ŽO9□úVß€4{□□ÀZ.™x1s□°óW9ÚÇ,WðÎ?
Ð²ðÞ…¦Ý□«□□Nµ¸9Ì°Z¢?æ□kR€
(¢€
(¢€
(¢€
(¢€
(¢€
(¢€
(¢€
(¢€
(¢€
(¢€
(¢€
(¢€
(¢€
(¢€
(¢€
(¢€
(¢€
(¢€
(¢€
(¢€
(¢€
(¢€
(¢€
(¢€
(¢€
(¢€
(¢€
(¢€
(¢€
(¢€
(¢€
(¢€
(¢€
(¢€
(¢€
(¢€
(¢€
(¢€
(¢€
(¢€
(¢€
(¢€
(¢€
(¢€
(¢€
(¢€
(¢€
(¢€
(¢€
(¢€
(¢€
(¢€
(¢€
(¢€
(¢€

(¢€
(¢€□”2•a•F□ªÿ `¶ÿ žCó5fŠ -ö
oùä?3NŽÒ□œ:F□

‡&§¢€
(¢€
(¢€
(¢€
(¢€
(¢€
(¢€
(¢€
(¢€
(¢€
(¢€
(¢€
(¢€
(¢€
(¢€
(¢€
l'¤¨Q×*{S¨
ß`¶ÿ žCó4ä³,7

'€Ã¡É©è □Š(□Š(□Š(□Š(□Š(□Š(□Š(□Š(□Š(□Š(□Š(□Š(□Š(□Š(□Š(□Š(
□å,)ñæ.ìtæ¢û□·üò-
™«4P□o°[□Ï!ùš>Ámÿ <‡æjÍ□□[ì□ßóÈ~f¬ô□Q@□□Q@□□Q@□□Q@□□Q@□□Q@□□Q@□□Q@□□Q@□
□Q@□□Q@□□Q@□□Q@□□Q@□□Q@□□Q@□□Q@□□Q@□□Q@□□Q@□E-´30i□q□□jZ(□·Ø-¿ç•üÍ-
`¶ÿ žCó5fŠ lq¤H□□
;S¨¢€
(¢€
(¢€
(¢€
(¢€
(¢€
(¢€
(¢€
(¢€
(¢€
(¢€
(¢€
(¢€
(¢€
(¢€
(¢€ {8$rï□,zœšoØ-¿ç□üÍY¢€+},Ûþy□ÌÑö
oùä?3Vh □¢¶†□-
□m$c-KE□ QE□ QE□ QE□ QE□ QE□ QE□ QE□ QE□ QE□ QE□ QE□ QE□ QE□ QE□ UsclI&
!"îjÅ□□[ì□ßóÈ~f•°[□Ï!ùš³E Vû□·üò-
™©ã□b@ˆ0£ §Q@□□Q@□□Q@□□Q@□□Q@□□Q@□□Q@□□Q@□□Q@□□Q@□□Q@□□Q@□□Q@□□Q@□□Q@□□
Q@□□Q@□□Q@□□Q@□□Q@□□Q@□□Q@□□Q@□□Q@□□Q@□□Q@□□Q@□□Q@□□Q@□□Q@□□Q@□□Q@□□Q@□
□Q@□□Q@□□Q@□□Q@□□Q@□□Q@□□Q@□□Q@□□Q@□□Q@□□Q@□□Q@□□Q@□□Q@□□Q@□□Q@□□Q@□□Q@
□□Q@□□Q@□□Q@□□Q@□□□w¶'ÎÐGu
Ì¿z5•□-
QSÐ□E□P□E□P□E□P□E□P□E□P□E□P□E□P□E□P□E□P□E□P□E□P□E□P□E□P□E□P□E□P□E□P□E□P
□E□P□E□P□E□P□E□P□E□P□E□P□E□P□E□P□E□P□E□P□U¯¤x-YÑ°ÀŽjÍTÔ¿ãÉ¾£ùÐ□;K¹äº•-B
TžF□¥kÖ□‡ü~Åõ?Ê·h ¢Š(¢Š(¢Š(¢Š(¢Š(¢Š(¢Š(¢Š(¢Š(¢Š(¢Š(¢Š(¢Š(¢
Š(¢Š(¢Š(□¥äÒE-
¸FÀfÁ÷éVêŽ¡þº×ýÿ ð«Ô QE□ QE□ QE□ QE□ QE□ QE□ QE□ QE□ QE□ QE□ QE□ QE□ QE
□ QE□ QE□ QE□ QE□ QE□ QE□ QE□ QE□ Vv£s43"ÆåA\ô-□ÉÕ¿×§û¿Ö€&Ó®%□¤□>ì□Ž+B²
ôŸ¿/ÐV¥ □QE □QE □QE □QE □QE □QE □QE □QE □QE □QE □QE □QE □QE □QE □QE □QE
d]ÝÏ□Ôˆ'□ ð0=+JÙ™í£f9b "X×ßñû/Ô□*Ø´ÿ •H¿Ý□□5□Q@□□Q@□□Q@□□Q@□□Q@□□Q@□□Q
@□□Q@□□Q@□□Q@□□Q@□□Q@□□Q@□□Q@□□Q@□□Q@□a½õÈv□S€}□nW6ÿ ë□êh□KN¹šiÙdrÀ.q•q
ZU'¥□ÇËÿ ¹ýEkÐ□E□P□E□P□E□P□E□P□E□P□E□P□E□P□E□P□E□P□E□P□E□P□E□P□E□P□E□P□
E□P□E□P□E□P□E□P□E□P□E□P□E□P□E□P□E□P□E□P□E□P□E□P□E□P□E□P□E□P□E□P□E□P□E□P
□E□P□E□P□E□P□E□P□E□P□E□P□E□P□E□P□E□P□E□P□E□P□E□P□E□P□E□P□E□P□E□P□E□P□E□
P□E□P□E□P□E□P□E□P□\×Ž<7□â½□t«□bM-d•MÄ±©-
$X;□`Ž¤□Ë□ë¥ªZ¾Ÿý-£Þiÿ hšÛí0´^t-‡LŒdZ ð¯ˆŸ
¼7ào□>¹¥êWöÚ¥¬'ù□%ÈÌÎX

□ó□-ãÒ½§Â7—š□ƒ´kÛü›¹ì¢’RF
1PIÇlõükÅü]ðFëIÒg×ì¼G6¡>›□¹0j1 □•>cÉ$-□ùH
ô5ë_□|M'‹ü□§k□B□Ï*²J‰÷C«□${□g□³Š éè¢Š (¢Š (¢Š (¢Š (¢Š (¢Š (¢Š (¢Š (¢Š (
¢Š (¢Š (¢Š (¢Š (¢Š (¢Š (¢Š (¢Š (¢Š åþ!øŽ□
ø&ÿ R³P×¸XmTŒþõØ*œwÆs•jç-þ

èW6 .¹s¨ßkŽ»¦Ô□ì□Ä‡©A□ □Ó Ô¿□?wà‹{—
ÿ So©ÚË)ì□>9üÅz'Z àþ□ê°”¶ºÇ‡õ‹¦»¾Ð¯M¯Ú_ïM□□□›ß□ý+¼¯;ð□ïþ!|B»□˜ZúÞ GMñÆC
□Ì×¢P□E□P□E□P□E□P□E□P□E□P□E□P□E□P□E□P□U5/øòo¨þunªj_ñäßQüè□6Ãþ?búŸå[µ…aÿ -
±}Oò-Ú (¢Š (¢Š (¢Š (¢Š (¢Š (¢Š àn>□hºÅÍÅç‰o5□fîì□Õå¹x’ O
□!□@□õªÿ
åº·¹ñ>‰öù¯ô-+Pò,n&}ì□□h÷wÛÀükOÄÿ □□Ä×·□-âŸ□Ø¬È□ÚÚ^í·□□ÿ W·¿~y¬Ï‡×7¾ñ□¡à
åµ‘tûu»³º¶€CæÄÇ□z

□□zŽ¼ýh□Ñ«Šñv‡-kk¨‹-[ìz

6ÌÉmdJÍrÁ2|Ù?…sÆÕê:žÕÚÕ□oþ@:•ýzËÿ š æ¾□’~□x□Póíÿ ³5vuÆ|&ÿ ’Yáÿ úö?ú□Wg
@□□Q@□□Q@□□Q@□□Q@□□Q@□□Q@□□Q@□u□õÖ¿ïÿ …^ª:‡úë_÷ÿ Â¯P□E□P□E□P□E□P□E□P□E□
P□E□P□^1ñ+CÖ¿á–‡Yñ□¬f°□□°ÁcfJZÛ©□v<Èøþ&é“€+ÙëÏ¾2□È‘□ý„–□ð` □A¢Š(□,ŸáF•¬
\Ü]ø–óPÖnå‘™□[—‰ □ð±¢□□□□Z¯ðÎKÝ?ZñW…¦¿žúËF¹„YÏpÛ¤   "□òÉï· ~'é]□‹¼W–
†ìâŠ□Mî±xÆ+
□þüòzŸD□Y°□Lð?…äðÎ•/Ûg□:½üÍw¨\

•3u□ý‘Ð~|f€:j(¢€
(¢€
(¢€
(¢€
(¢€
(¢€
(¢€
(¢€
(¢€
(¢€
(¢€
(¢€
ÉÕ¿×§û¿Öµ«'Vÿ ^Ÿîÿ Z v"÷åú
Ô¬½'ïËô□©@□□Q@□□Q@□□Q@□□Q@□□Q@□SÕ-.oô›«KK×±¸š2‰rŠ□¢'ø€=êå□□åÚßÂO
Xh□°"^êQjvÐ<ßŰó_Èe

£;›•½G`+ªøw©j□¿Ãí□ÿ U,of¶□Fa,ã$+ª€□□æ5¿„□¨Æd‹ÅúõÄÊþlpê³-Ý¶ìä□□(□~uÑü<ñ-
Ç‰ü/ö‹Ûh-ï-.$²¹H□Ðù'œ□¾Ø"€:°óÏ□ÜMâ-cþ□»□]-b^]ëSÆpR.© =™ÈÉÿ d{×Mã/□Gá-
□ê:ä'□~Ë□)□þ'b□AöÜF}«Ìü)ñ□À:□†®□ïÄ,ãY¿/s□qö9ÿ y;Ž@>_Ý_°;`{Ð□cð|"ð£@$'|§
ëÿ]-»Šòß,^+Ñ/¼□¦xvÚ÷~-glòOoå8Ø¾içq□Oß^‡½z• □QE □QE □QE □QE □QE □QE □QE
`ß□Çì¿Qü«bÓþ="ÿ tV=÷ü~ËõÊ¶-
?ãÒ/÷E ME□P□E□P□E□P□E□P□E□P□E□P□□‰ü;/‰mmìÿ µïtûA&ë•³m□:ã„ßÕFzã-y¿•ü□§|7
Ñâñ_„¤»°½´¹^K□Ú^Hï□œ)G

Nzçóö#Ù□,©f (□$ô□ç

Çâo‰í^□O„´k•;Îí¨]/ /¬hsÏBxç¨ ôŠá~&êÚ□µŽ□¢éWmeu-ê□Ù□´ûÐÆyf_ö°□ø÷®ê¹Ÿ□øHx
»H,□¯^ÆþÎá.ì®Ñw□¥^„ŽãŸåé@□Æ£ðkD·Ó%¸Ð¦Ôlõø□¼□€½‘¤y Èß“Œ□× u®£áÿ ˆ¥ñW•t-b
|}¢hŠÍ□ŒÈŒQŽ;d©8÷®~KoŠBÒE¿×|1ck□□’þÞÚW˜(□-µ°™Ç>•càÅ¤–Ÿ
ô•4□Óy³

ÿ u¤b§ñ□?• w´QE □QE □QE □QE □QE □QE □QE □Í¿úÆúšé+›□ð•õ4□wJÿ •—
ÿ súŠ×¬□+þ>_ýÏê+^€
(¢€
(¢€
(¢€
(¢€
(¢€
¡-ë6~ÑnõmBO.ÖÖ3#žçÐ□RN -¦¯×•xßÆÞ-"âE¾‡â=CìzF‹²îXš
$û]É□£R□OÈ€†ç©8ç□□SFÓõ4øµám{Z.š¦µ□ìò[-;m¢□•* ?Ù□'ÜšöÊñ□kâ—
ƒ.þ&ø_WƒYßac□Ú\KöY†Âè□Œ□ÉÉ□□¯g±½·Ô´ûkëI<Ëk˜-hŸ□nF□□□'Á□h□—
ø□-ê6□:v'¢J±júÕÐ´·™†D
ŒÉ&;íQúÖj|□ð£Æ$¿þÒ¾Ôq-Ô&¿—Î-ëÃ ?*ÏøŸ§□jxïÀV□{ue□Ó^D÷□'yr)1¦□·bpGâiúçÂ-
□Ùh·°†•×ú~¯o
Í□ú_J_z,Al¶1'ÏJ ô›[qig□²É$,□Ö0ò¶çl

e□sêjjç<□¬ÝxƒÀz6©{ÍÔöàÊØÆæ□©l{ã?□tt QE□ QE□ QE□ QE□ QE□ QE□ QE□ QE□ QE□
QE□ QE□ QE□ QE□ QE□ QE□ QE□ QE□ QE□ QE□ QE□ QE□ QE□ QE□ QE□ QE□ QE□ QE
□ QE□ QE□ QE□ QE□ QE□ QE□ QE□ QE□ QE□ QE□ QE□ QE□ QE□ QE□ QE□ QE□ QE□ Q
E□ W!ãÿ

ëž"°²‶ÃÚãéZ□”ÆXÎæ□Ë‛□¯Žß□□F9®¾Š ñ{ß
üañ%‶èÚ¾¹¥Ûiò•.âhT□•;ýÕÏ>Ÿ(=□□ê^□ðõ§…|9e¢Ù□0Z¦ÐÍÕØ’Y□Ô’kZŠ (¢Š (¢Š (¢Š
(¢Š (¢Š (¢Š (¢Š (¢Š (¢Š (¢Š (¢Š (¢Š (¢Š (¢Š (¢Š (¢Š (¢Š (¢Š Í×ôK?□h7°=ú
‶mw□GÛÕ{†-à€G¸®-×Aø¥¦Ø&□iâ□□k8×ÊŠþâÞOµ*

□Û÷ □×>õéTP□□„<-má□ tè&’âW‘§¹¹—
ïÏ+}ç?^? :Öõ□P□E□P□E□P□E□P□E□P□E□P□E□P□E□P□E□P□U5/øòo¨þun«_FòŰ²"å²8

»□øý‹ê□•nÖE□¤ñÝFï□
□'ð-z (¢Š (¢Š (¢Š (¢Š (¢Š)□È#$g¸¥¢€<î□□â†Œ¦ÓNñ

‰ªÚ

›W†E•WÐ˜þñ÷=k_Â-□°Ñ/õ□oZÔ¿´µÝGjÏ:Ç²8Ñ~ìh½€õï□]m□□…§Zø’?□j·□ú…¬°□‹□°¶□1
$L ÜXí□ÉÏsøVWŠ‾þ ÝÝ][h'Ã#Kš/,□ÿ ?Ï□\7Üù~•ÙQ@-wàM□Çþ□¶Ó4mBO

É¢Z##µ¹œÜ‛,F □s¸ŽÝ+Ñ(¢€
(¢€
(¢€
(¢€
(¢€
(¢€
(¢€
(¢€(êë–□ßÿ
½U/"y%·(¤…lŸn•n€
(¢€
(¢€
(¢€
(¢€
(¢€
(¢€
òŸ□øoâ□Š¬

Ÿq/„#µ[”ž3[illegible]\‡ù[illegible]*

AZõj(□žðÏü&;®□á+þÂÛ…û?öW□žû·ùŸð□cÞ¤ðí¯‰-
§ÕN¿¨ZÝÅ%Û=,À›LPvVùFOçõ5»E y&ŸáO‰Úo^oõ±/„/u
³´\Þ5Ë<Qv□□€□}€äõ&»O□ÿ Âwöù?á(ÿ „sì^Qòÿ ³<ÿ 3ÌÈÆ|Î6ãw¾q]=□ QE□ QE□ QE□
QE□ QE□ QE□ QE□ QE□ QE□ QE□ QE□ QE□ VN-þ½?Ýþµ-Y°□¼³L†4,□â€□¤ýù~,µ+?M,X^
C"□È□-
 (¢Š (¢Š (¢Š (¢Š (¢Š +3Ä:mÐ¯¢\YXj“i—
oµ¢»„e£e`Ý;ƒŒ□Ü□ZtP□••7âÌ•□'×<5□Dm7ém/Ú þðO¹šéü+á‹□xj-
"ÆW•“s½Ä¼´²±Ë;~? □»E bxN×Ä6š□Eâ‹ûkíLHÅ¦¶M¨W?(ÆÕè=¿Æµî#3[K□
□B ŸqRQ@□ç€ü?uáo□éš-
ì□ÉsjŒ®ð’P’ìÜ□□ïé]□□P□E□P□E□P□E□P□E□P□E□P□E□P□E□P□□÷ü~ËõÊ¶-
?ãÒ/÷Eg]ÚO%ÔŽ‘’¤ð□
Ò¶VKhÕ†□P□ h¢Š (¢Š (¢Š (¢Š (¢Š (¢Š â¾$x{ÄÞ&Òm´ï□ÞXAnÒ□}□Ü’
•8ÂfONÓÎà□'ŽzÖu-¯Å{□H-m!ð□6ð¨Hâ□nÂªŽ€
ôj(□ŸÖíüSwàÿ 'I½°²ñ□G□éÊ-•_#ÌÚ□Xãï□□OJ‟[µñ$Ú□°h°□□¾«□#H÷O—Šl)

¤

T□AÈçŠÜ¢€<æçÂÞ;ñT_`ñV·¥Yé.□Ò-ôXä□r¿Üg"•□¾>•è6ÖÐÙÚÃkm□Å□(#Ž5□
`□ì□KE □QE □QE □QE □QE □QE □QE □QE □Í¿úÆúšé+
ì®K±□□gÔP□ÚWü|¿ûŸÔV½féÖÓC;4ˆT□Æ□□ZT QE□ QE□ QE□ QE□ QE□ QE□□Ì
j¾□»¿ñÿ ‡µø¥□mtØnc™□□ìdP□hÆ;s'*×‰í|Iu□ˆðÞ¡ke*]£Ý5Ân□ Îå-
)äñéõ□»E s^6ð˜ñn'

□^5Ž¡g:ÝY]¢äÃ*ô8î=¾ž•Í]øoâGˆ¬[G×5Ý□ÓL”yw3i•Èn&•°üÿ *äpqúô¯J¢€*é°}¶‶¦ZéÖ
Qˆí-¢X¢OEQ□V¨¢€
(¢€
(¢€
(¢€
(¢€
(¢€
(¢€
(¢€
(¢€
(¢€
(¢€
(¢€
(¢€
(¢€
(¢€
(¢€
(¢€
(¢€
(¢€
(¢€
(¢€
(¢€
(¢€
(¢€
(¢€
(¢€
(¢€
(¢€
(¢€
(¢€
(¢€
(¢€
(¢€
(¢€
(¢€
(¢€
(¢€
(¢€
(¢€
(¢€
(¢€
(¢€
(¢€
(¢€
(¢€
(¢€
(¢€
(¢€
(¢€
(¢€
(¢€
(¢€
(¢€
(¢€
(¢€
(¢€
(¢€

(¢€
(¢€
(¢€
(¢€
(¢€
(¢€
(¢€
(¢€
(¢€
(¢€
(¢€
(¢€
(¢€
(¢€
(¢€
(¢€
(¢€
(¢€
(¢€
(¢€
(¢€
(¢€
(¢€
(¢€
(¢€
(¢€
(¢€
(¢€
(¢€
(¢€
(¢€
(¢€
(¢€
(¢€
(¢€
(¢€
(¢€
(¢€
(¢€
(¢€
(¢€
(¢€
(¢€
(¢€
(¢€
(¢€
(¢€
(¢€
(¢€
(¢€
(¢€
(¢€
(¢€
(¢€
(¢€
(¢€
(¢€
(¢€

(¢€
(¢€
(¢€
(¢€
(¢€
(¢€
(¢€
(¢€
(¢€
(¢€
(¢€
(¢€
(¢€
(¢€
(¢€
(¢€
(¢€
(¢€
*1<Fo+xßéRPEPEPEPEPEPEPEPEPEPEPEPEPEPEPEPEPEPEPEP
EPEÈåIs±·`àÐè¢Š (¢Š (¢Š (¢Š (¢Š (¢Š (¢Š (¢Š (¢Š (¢Š (¢Š (¢Š (¢
Š (¢Š (¢Š (¢Š (¢Š (¢Š (¢Š (¢Š (¢Š (¢Š (¢Š (¢Š (¢Š (¢Š (¢Š (¢Š (¢Š (¢Š (
¢Š (¢Š (¢Š (¢Š (¢Š (¢Š (¢Š (¢Š (¢Š (¢Š (¢Š (¢Š (¢Š (¢Š (¢Š (¢Š (¢Š (¢Š
(¢Š (¢Š (¢Š (¢Š (¢Š (¢Š (¢Š (®ÇŸ´oßÚØ][Ïyw:ù•G–
™À''¿8-Ý¸Ïs‹4)*çk¨aŸC@¢Š(¢Š(¢Š(¢Š(¢Š(¢Š(¢Š(¢Š(¢Š(¢Š(¢Š(¢Š(
 ¢Š(¢Š(¢Š(¢Š(¢Š(¢Š(¢™,±Á
Í+¬q¢-wc€ rI=…p|bÐq$öšV¿}§ÆÄ>£k§³[Œu;‰¥ zGGÖ4ýJƒSÒîRæÎuÊH½ûAäx
Õê (¢Š (¢Š (¢Š (¢Š (¢Š (¢Š (¢Š (¢Š (¢Š (¢Š (¢Š (¢Š (¢Š (¢Š (¢Š (¢«j…¦“
§Ïþ-ö°!yes€¢€,Ñ^v~2h(‹u.“â´Æ#›éÌ-
ˆ=÷g8ü+¾´»·¾´†îÖdšÞdG"«) šŠ(Š(Š(Š(Š(Š(Š(Š(Š(Š(Š(
Š(Š(Š(Š(Š(Š(ŠÄñ7Šô•XÇuªÎËæ¿—1!yfî¢ŽIý+
Nø©¢^jÖÚeõ†±¢Ü]6Ûoí[3ÌÝ,œ'Ÿ®(¸¢Š(¢Š(¢Š(¢Š(¢Š(¢Š(¢Š(¢Š(¢Š(¢Š(
 ¢Š(¢Š(¢Š(¢Š(¢Š(¢Š(¢Š(¢Š(¢Š(¢Š(¢Š(¢Šç<OãmÂoé¸¸¾¹Ï'eg-
y@êBŽÐçÑÑ\~ƒñ'F×5•Ñä¶Ô´-MÔ´vš¥©•ä®ÞH?Læ»
 (¢Š (¢Š (¢Š (¢Š (¢Š (¢Š (¢Š (¢Š ÌOù7ãü«N³þCMøÿ *Ó Š(Š(Š(Š(Š
(Š(ŠŽ{^mmä¸¸-8aK<'0UP:'OA\×‡¼¢x£]¼Ò´£s+[B&ûCE¶)Tœe
9ažøÁìM u4QY-'ñŸ…<=u-_Ç<-Öû$
Ø³ @êÃ½ kÑ^ý
Oþ¤/ÿ àŸÿ ³®òÞ_>Ú)¼¹#ó6Ér3,; 'Š(Š(Š(Š(Š(Š(Š(¨é¿vo÷êõ
QÓ~ìßïÐê(¢€
(¢€
(¢€
(¢€
(¢€
(¢€
(ªºŽ¥e¤XË}¨ÝEkkËË+QøÐª+œð¯•t¿¶ 4ÈîÕleXÝ®"ò÷î

œà•P□µtt QQÏq□-¼—□□Ç

1©g’F
ª□RIè+šð÷□ôO□k·šV”nekhDßhh¶Å*‟Œ¡',3ß□=‰

¦Š(□Š(□Š(□Š(□Š(□Š(□Š(□Š(□Š(□Š(□Š(□Š(□Š(□Š(□Š(□Š(□Š(□Š(□Š(
 □Š(□Š(□Š(□Š(□Š(□Š(□Š(□Š(□Š(□Š(□Š(□Š(□Š(□Š(□Š(□Š(□Š(□Š(□Š
(□Š(□Š(□Š(□Š(□Š(□Š(□Š(□Š(□Š(□Š(□²<Oâ+/
øvóY¿oÜÛ¦B□Ì□ÑT{‟□úÖ½y_Åï□x¯Å÷:Dz(´—
O´c4¶÷□m□.x,;Œd~'Ö€<¿Å~-½›áÔ¾:×Æíg[Ô¡xÁÿ -6û_h-™□}~P¾õôå□üƒí¿ë’ÿ !_8üR-
□×Âp/Š†-4Ñw□Qk·p‟kméÛ□ý+Ú~ÿ Â]ý^ßð-
ý‹ÍÊý›ìÝ|½£ïcŒÿ õè□-¢Š(¢Š(¢Š(¢Š(¢Š(¢Š(¢Š(¢Š(¢Š(¢Š(¢Š(¢Š(¢Š(
 ¢Š(¢Š(¢Š(¢Š(¢Š(□€øÉs4□□¥³…Ú6Ôn ³,½B»□ß˜□~5ÜYYÛéö0YZD±[A□Ž8Ô`*□€+Šø
Ãcswðòêâ Ò3$ú|Ñª

â6□¿%$þ□Óiþ%Ñõ-□=n□Bßû=£□4Í □1Œ•Ç?)□Áé@□wÃX—KñOŽt□□ËKMI.!ŒtA2□
z□”q^•^uð¸•VûÅ^*TeµÕõ□Z3

y•Ä6+ý

OäkÑh ¢Š(¢Š(¢Š(¢Š(¢Š(¢Š(¢Š(¢Š(¢Š(¢Š(¢Š(¢Š(¢Š(¢Š(¢Š(¯:ø¤£
Q½ð‡‡å□-5□]□Êv’8Æâ‡Øä~Uèµç?□
i£Ã>%(Ím¤j±½ÑQ’•¿ÊÍøp?□ ô□- ¸µ’Öh‘íäC□ÆÃå*F□Ç¦+□ø6Ï□ƒït¶vtÒµ[›(Ë□□ªÁ‡þ„
k®½ñ&•§èO-\j6ÃNXüÁ:È
¸ÆFßR{□Ö¹□„VW0x
ßÝÄÐÍ«^M¨˜Ûª‰□åüÔ□øÐ□yE□P□E□P□E□P□E□P□E□P□E□P□E□P□E□P□E□P□E□P□E□P□E□P□E
□P□E□P□E□P□E□P□œÄ‹«ü{°k•

š.□,ÝO!$•²\{í8-‹:d:—Ã=kÌQæZÃö¨_°<□0 ö8□} ¬›«È|3ñÓí□„‹□-»¥¬0Ï!Úž|m÷
<□—‰□®üZÖíí|□w¤A"Íªêà̄YÙÚ#□ò— □□ □óÓ ï@□g‡o_RðÎ"□)Ì—
6pÌÇÝ•□üëJ©hÖÙz-Ÿ§d-
²ÛG□G}ª□úUÚ (¢Š +Î|□‹ª|Nñ¾³p¡æµž-
:ÜŸùe□®X□Lœ□^□^gá‹Ø<;ñgÅº%ü©□j'E¨X™

Ñ0*C€OR□océ@□üf‰m¼-a¯F6Ði□•½Ä2

F}

FG°¯G¯4ø¯y□±□"àËIRmGS¿„É
□´P#nw`:□□×¯>•ét QE□ QE□ QE□ QE□ QE□ QE□ QE□ QE□□˜Ÿò□oÇùV□f'ü†›ñþU§@□□Q
@□□Q@□□Q@□□Q@□□Q@□□Q@□úÆ‡¦ëöÑ[j¶©uo□«0‰ÉØYs•Ã£

z□Šã4„Xþ:k¨Š□WF·□@À□wJô:óí/þK¾½ÿ `{□ý

€=□¸□‹ ––W¿

õH,íg°œ½»,0F]Ø ã' rx□×_{}i¦Ú=ÝõÔ□¶É•óO D\œ

±àri'Ôl-V□¸¼·…g`±□%U□□3…Éäà□Å pçâö□léý¯¡ø"F·f
ö•GM1Ä3êA'ô®ö â°·Žâ RXePñÈ‡*ÊFA□¸Åbø¿VÑt¿
j□ëSÀ,ÞÝÕ£'‡ïr§åQÜž˜□›ð®Ê÷Nøc Û_«-À€±Wêª ÎÌ úaJŒP□aE□P□E□P□E□P□E□P□E□P□E
□P□E□P□Ttß»7ûõz¨é¿vo÷è□õ□Q@□□Q@□□Q@□□Q@□□Q@□□Q@□fêZ□—
¬]Ù\ê6qÜÉdÌöâ\•F8ù¶ô'Ž □v-*(□Ï¼□ÿ #¿•ÿ ì%-
þ‹¯A¯>ð□üŽþ?ÿ °"□ú.»‹ ÍFËNXšúòÞÕe□E□žU@îz(ÉäŸJ ƒXÐôÝ~Ú+mVÕ.-ã•f□9;
.q¸taÏC'\f□‹-
ÇMu□BªèÖà(□ né^‡^}¥ÿ Éw×¿ì□oÿ ¡Ð□ ÑE□ QE□ QE□ QE□ QE□ QE□ QE□ QE□ QE□ QE□ Q
E□ QE□ QE□ QE□ QE□ QE□ QE□ QE□ QE□ QE□ QE□ QE□ QE□ QE□ QE□ QE□ QE□ QE□
QE□ QE□ QE□ QE□ QE□ QE□ QE□ QE□ QE□ QE□ QE□ QE□ QE□ QE□ QE□ QE□ QE□ QE□
QE□ QE□□‹â□
i~/ÒF›«Å$-
Ë*Ì□HP†□ÁÈúšØ•□(Ö4□U□@ô□•E □QE □QE □QE □QE □QE □QE □QE □QE □QE □QE □QE
□QE □QE □QE □QE □QE □QE □QE !□•□□□□□ã&øKà9ïÍëønÛÎ-¸…wTÏû□¶þ□®ÒŠ Ž□!¶,8
‰"†5
'Æ¡U@è □□IE□ QE□ QE□ QE□ QE□ QE□ QE□ QE□ QE□ QE□ QE□ QE□ QE□ QE□ QE□ QE
□ Tw□ðÝÛÉos□M□ªQã'C+)ê□=EIE qvÿ | k~/bðå·œ□p

îè□û…Šþ•Ú

ĐQE QE QE QE QE QE QE QE QE QE QE QE QE QE QE QE QE gk
Z

•â;□c¬XCylNí’¯Ý> õ□ÜVN□ðïÂ^□¼ûf‘¢Aos,□ÌÍ#.zí.I-
…tôP□E□P□E□P□E□P□E□P□E□P□E□P□E□P□E□P□E□P□E□P□E□P□E□P□E□P□E□P□E□P□E□P□E□
P□E□P□E□P□E□P□E□P□XÞ!ðž…â«tƒ[Ó!¼HÉ(_!“=pÀ,?□[4P□?áß□xkÂm#hšD□’H6´€³¹–›˜
“•l×AE□ QE□ QE□ QE□ QE□ QE□ QE□ QE□ QE□□˜Ÿò□oÇùV•@-c□&ã-
¼ûñÒ§ □Š(□Š(□Š(□Š(□Š(□Š(

¯□øoIñV™ý□-Z}ªÓx‟Ëó□>aœ□©□¹ï\§ü)/‡Ÿô/□àíÇÿ □¯@¢€1nü'¢_øb?□ÝX‰4^ãHÖÜÈã
˜Ú7□»Œ

sOÕ¼/¢k°TZf§¦Áug

<¨œ‟□□□r8ãƒZôP□□¦|)ð>‛z——~-·□¡Ü-+¼¡O¨

Ä]•PEPEPEPEPEPEPEPTtß»7ûõz¢†Ý

□?1ÉÍ KE□P□E□P□E□P□E□P□E□P□E□P□X^&ðn•ã□mâ×¬>Ö–ìZ!çI-Òp□Üažƒ-nÑ@-□ÿ
Káçý
ßù;qÿ Ç+«Ö|7¤x,
Hu[%¹ŽÒež□geØëÀ<□ž½□□«E ex‡ÃzOŠ´ÏìíjÓíV›Äž_˜éó

àåH=Ïzå?áI|<ÿ ¡{ÿ 'n?øåz□□□[O°¶Ò´ëm>Ê/*ÖÚ%Š(÷□ÚŠ0□I$ñëVh¢€
(¢€
(¢€
(¢€
(¢€
(¢€
(¢€
(¢€
(¢€
(¢€
(¢€
(¢€
(¢€
(¢€
(¢€
(¢€
(¢€
(¢€
(¢€
(¢€
(¢€
(¢€
(¢€
(¢€
(¢€
(¢€
(¢€
(¢€
(¢€
(¢€
(¢€
(¢€
(¢€
(¢€
(¢€
(¢€□˜"–=ª/´/¡§Íþ©ª¥ XûBú□>Ð¾†«Ñ@□>Ð¾†•´/¡ªôP□•´/¡£í
èj½□□cí
èhûBú□¯E XûBú□>Ð¾†«Ñ@□>Ð¾†•´/¡ªôP□•´/¡£í
èj½□□cí
èhûBú□¯E XûBú□>Ð¾†«Ñ@□>Ð¾†•´/¡ªôP□•´/¡£í
èj½□□cí
èhûBú□¯E XûBú□>Ð¾†«Ñ@□>Ð¾†•´/¡ªôP□•´/¡£í
èj½□□cí
èhûBú□¯E XûBú□>Ð¾†«Ñ@□>Ð¾†•´/¡ªôP□•´/¡£í
èj½□□cí
èhûBú□¯E XûBú□>Ð¾†«Ñ@□>Ð¾†•´/¡ªôP□•´/¡£í
èj½□□cí
èhûBú□¯E XûBú□>Ð¾†«Ñ@□>Ð¾†•´/¡ªôP□•´/¡£í
èj½□□cí
èhûBú□¯E XûBú□>Ð¾†«Ñ@□>Ð¾†•´/¡ªôP□•´/¡£í
èj½□□cí
èhûBú□¯E XûBú□>Ð¾†«Ñ@□>Ð¾†•´/¡ªôP□•´/¡£í
èj½□□cí
èhûBú□¯E XûBú□>Ð¾†«Ñ@□>Ð¾†•´/¡ªôP□•´/¡£í
èj½□□cí
èhûBú□¯E XûBú□>Ð¾†«Ñ@□>Ð¾†•´/¡ªôP□•´/¡£í
èj½□□cí
èhûBú□¯E XûBú□>Ð¾†«Ñ@□>Ð¾†•´/¡ªôP□•´/¡£í

èj½□□cí
èhûBú□¯E XûBú□>Ð¾†«Ñ@□>Ð¾†•´/¡ªôP□•´/¡£í
èj½□□cí
èhûBú□¯E XûBú□>Ð¾†«Ñ@□>Ð¾†•´/¡ªôP□•´/¡£í
èj½□□cí
èhûBú□¯E XûBú□>Ð¾†«Ñ@□>Ð¾†•´/¡ªôP□•´/¡£í
èj½□□cí
èhûBú□¯E XûBú□>Ð¾†«Ñ@□>Ð¾†•´/¡ªôP□•´/¡£í
èj½□□cí
èhûBú□¯E XûBú□>Ð¾†«Ñ@□>Ð¾†•´/¡ªôP□•´/¡£í
èj½□□cí
èhûBú□¯E XûBú□>Ð¾†«Ñ@□>Ð¾†•´/¡ªôP□•´/¡£í
èj½□□cí
èhûBú□¯E XûBú□>Ð¾†«Ñ@□>Ð¾†•´/¡ªôP□•´/¡£í
èj½□□cí
èhûBú□¯E XûBú□>Ð¾†«Ñ@□>Ð¾†•´/¡ªôP□•´/¡£í
èj½□□cí
èhûBú□¯E XûBú□>Ð¾†«Ñ@□>Ð¾†•´/¡ªôP□•´/¡£í
èj½□□cí
èhûBú□¯E XûBú□>Ð¾†«Ñ@□>Ð¾†•´/¡ªôP□•´/¡£í
èj½□□cí
èhûBú□¯E XûBú□>Ð¾†«Ñ@□>Ð¾†•´/¡ªôP□•´/¡£í
èj½□□cí
èhûBú□¯E XûBú□>Ð¾†«Ñ@□>Ð¾†•´/¡ªôP□•´/¡£í
èj½□□cí
èhûBú□¯E XûBú□>Ð¾†«Ñ@□>Ð¾†•´/¡ªôP□•´/¡£í
èj½□□cí
èhûBú□¯E XûBú□>Ð¾†«Ñ@□>Ð¾†•´/¡ªôP□•´/¡£í
èj½□□cí
èhûBú□¯E XûBú□>Ð¾†«Ñ@□>Ð¾†•´/¡ªôP□•´/¡£í
èj½□□cí
èhûBú□¯E XûBú□>Ð¾†«Ñ@□>Ð¾†•´/¡ªôP□•´/¡£í
èj½□□cí
èhûBú□¯E XûBú□>Ð¾†«Ñ@□>Ð¾†•´/¡ªôP□•´/¡£í
èj½□□cí
èhûBú□¯E XûBú□>Ð¾†«Ñ@□>Ð¾†•´/¡ªôP□•´/¡£í
èj½□□cí
èhûBú□¯E XûBú□>Ð¾†«Ñ@□>Ð¾†•´/¡ªôP□•´/¡£í
èj½□□cí
èhûBú□¯E XûBú□>Ð¾†«Ñ@□>Ð¾†•´/¡ªôP□•´/¡£í
èj½□□cí
èhûBú□¯E XûBú□>Ð¾†«Ñ@□>Ð¾†□´/¡ªôP□□´/¡©TîP}j•\•ýZý(□ÔQE □QE □QE □QE □QE
□QE □QE □QE □QE □QE □QE □QE □QE □QE □QE □QE □QE □QE □QE □QE □QE □QE □Q
E □QE □QE □QE □QE □QE 2oõMU*ÜßêšªP□E□P□E□P□E□P□E□P□E□P□E□P□E□P□E□P□E□P□E□P
E□P□E□P□E□P□E□P□E□P□E□P□E□P□E□P□E□P□E□P□E□P□E□P□E□P□E□P□E□P□E□P□E□P□E□P□E□P
□E□P□E□P□E□P□E□P□E□P□E□P□E□P□E□P□E□P□E□P□E□P□E□P□E□P□E□P□E□P□E□P□E□P□E□P□E□
P□E□P□E□P□E□P□E□P□E□P□E□P□E□P□E□P□E□P□Y#Äú□×N‡ý©ký¨?å×Ì□úg□õÇ8-jù{ÆÖ—
ãâ□‰µ½9ˆ¸Ñ¦†ó□ž□Aœ{□□Ó4□õ□gk□ö•áûU¹Õ¯à³…Ûb´-•ÇÐzÑ k□þ Ð,ukR<«¸V@3•¤õSî

Gá^□ñzö□□k°Ó[?üK</□#0ä4òJ^ÃØòGý³4□ô]½Ä7vÑ\ÛÊ’Á*   #‛

U”Œ,□pEI\>Ÿâm?Â?   4=[Rv□&□l¨‰ËHæ1…Që×ð□×0ß□üUon5[¯ ^G£□□Ë½·*□x•½1ß -
Z õú+›ŸÇ□4>□ÿ „¸I#éžP•l\¹%¶ìÇ÷·□½qžõÁ\|^ñE□¿öµ×€náÑr
™Ýƒ□'†'n =¸Ç=h□Ø□•□»°UQ’IÀ□›□±Í□É□¬‛°Ê²œ,=•y¿Ä□j□¿

□ûÃÚIÔ4íRÊ_´ÌÍƒk□N¤

¤e³Øm®□á□ˆ¼V4m□IO

ïÐó"ÿ ioÇË½É8öl□Â€=®Š(

GÆ¿□ô¯□□Xn¡¸»½°Ï“ml ±□Œœô□àzŸÆ³ôï‹¾□»°²¸¼[»
.îÞÍ!ž"H‛B□Î:□æ''×§□åúç^ü['Æm+P‟ÂÛukkgŽ×O/»Î•÷¿>□□?ð□î|Eâi□>
—Äž□´:•õë'•;n6„:□ÊqÔ,-ƒè(□Õh®7Ç^=-□k□+M6mOV¿b-íb8È□IÀ'¿

yéU|ã_□ë:ãéZÿ „ît—òZd¸;Œm,□9□ïëøP□½¿Œí.<□uá□¶˜\ÛÛ
†œ'°ä)ÀïÑ…tµàž!ñRøCãÎ¯□ò ï§-Ê+x-¡ë$Œ'àwôì
ö®«Eø-©□ÂMe¢ø«Ã3èÏ~ÁmfbpXœ(Žäã
ðHÈô õ□(®'ÆŸ□□ÃZ•¶□¥è×ZÆ³q□œ¶ð,□G'7□□î□□íÉ□□ÛQ^gáÏŠW÷>)·ðçŠ<;6□{t3nìÄ«ž
p0GC,□□óÅMãßŠkà_□XéÓé¦æÚ{V•Ý

□,□□Ó□Q"è}¹ ôj+È£øÃ«é·Ö•â□□ÝiZ]ã„Žå™³-{•TgéÁÇ8=+Ð¼Wâ•?Â-—WÔ
□Ô„Ž4Æé\ôQù□ô□€5/n'ÆÂâî@Ì•DÒ°^¤($ãò¬•□ø¢ßÆ>□‡X¶·-□äfO.R
□N;W™j_□|Bt+‹□SÁ□vš=å»G□Ò³□›Ô…'*□□#□3Ú°_•ßòLlÿ ë¼¿ú□ z5□‹â¿□Ù
øKÃ·:Åê»Ç

□ÆŸzF'□GùéšátÏ‰¾,¸¿±kÿ □^A¦ÞÊˆ"¡f1†
□?/N{ã4□ê"W3ã_□Øx'JŽêæ'¹¹¸□ÖÒ#óÌÿ Ð

Œžz□Zâ□âçˆô–ŽïÄ~□¼±Ò¤`□Â–
Ý□> ŒgØí □]¢¹¯□xÛMðß†`ÖÊÉ{□ÙD³ŽÜdÎÎ¥”□@@'?Ìñ\,¿□üC£É□×ˆü□wa¥LáDÛ›rg×*□>
Çnh□Ôõ]RÓEÒ®u+ùDV¶Ñ™$□@==O`+ˆð·ÅÝ3Å> ¶Ò Òµ+oµ‡û4ó
ØûT±Î□□□Ó<Ö/ÆÝoV>□’ÖÃK□□%ä□K6¤¯ÄgÍR£–ø^□Úö«ÿ

¼Iâ□Ñ-`Ö´□ÓtK-)$‹Qi0$UUÁ ú®[>Ô□éÔW'Ÿ‹°æ³<òxOÁ—z-
Ÿ□•7NXoÇ\ 8>Ù'§□°Ÿ□|C´ñªÝ[5¤°~«fqqe1ÉQœd□□yàä□□ vtW›x□â¤Ö¾#›ÃÞ□Ð§×5□|ý
ÆHH^ê8□8<□À□ŠO

üTš÷Ä'xsÄÚ□ú-§8-G˜NÉO<r□3Ž□
ž(□Ò«šñ_Œ-<%6"□Õ´Ó□Jä[¡^□□ñÉÏÔV'Œ¾%¶ƒ®Åáí□H›YÖ□C´□□□@ŒŒà□N9Ç□□9¯3ñ••µ□□
ëž□Ó5□□ãHÔ¬õ□'’BJ°1P□È□¨>£Ð€>Œ¬í{X·ðþƒ}«]□Ð´…¤#8ÜGE-äà□-h×•üs¼•ü=¤è6ï¶]
Vý□•ï*ö?ð&CøP□U¡øÁgð

-)ñ

qiPÉ□•□bÁP¶□ôÉ,0@□ÎE/‡¾"xsÄÚ‡Ø,.ä[²žbCq
DÒ']Ë‘ÈÇ>¸æ¹•ÖBÓáæ™□¼L,m/ Y□□
□FQŸnƒêEEâ»ë□WâÏ□ßE»·žXD’Ï,

□,□Ï$p□□þ~ô□èÞ"ñ&—
á]%õ=ZãÉ·V□073±èª□S×ò5WÄÚýÎ“à»-{L²û[Ã□Ü,2æ2S,ÄŒd□¹8ö®□NVø¯ñ□µYAo
èR□µF-
Ôýw□Üt<öÚ1É¯Zž□îmå·™□Å*□u=□HÁ□□CÃºÝ¿^ü=a¬[q□ÜBM¹ÎÆèËŸb□ü+N¼«à}Ì¶úf½áÉÜ¼
šF È3Ù[#□þ□Œ□□õZ (¢Š (¢Š (¢Š (¢Š (¢Š (¢Š (¢Š (¢Š (¢Š (¢Š (¢Š (¢Š (¢Š (¢
Š (¢Š (¢Š (¢Š (¢Š (¢Š (¢Š (¢Š (¢Š (¢Š (¢Š (¢Š (¢Š (¢Š (¢Š (¢Š (¢Š (¢Š (
¢Š (¢Š (¢Š (¢Š (¢Š (¢Š (¢Š (¢Š (¢Š (¢Š (¢Š (¢Š (¢Š (¢Š (¢Š (¢Š (¢Š (¢Š
(¢Š (¢Š (¢Š (¢Š *ä□ê×éTêä□ê×é@

¢Š(¢Š(¢Š(¢Š(¢Š(¢Š(¢Š(¢Š(¢Š(¢Š(¢Š(¢Š(¢Š(¢Š(¢Š(¢Š(¢Š(¢Š(
¢Š(¢Š(¢Š(¢Š(¢Š(¢Š(¢Š(¢Š(¢Š(¢Š(□"□ªj©Væÿ TÕR€
(¢€
(¢€
(¢€
(¢€
(¢€
(¢€
(¢€
(¢€
(¢€
(¢€
(¢€
(¢€
(¢€
(¢€
(¢€
(¢€
(¢€
(¢€
(¢€
(¢€
(¢€
(¢€
(¢€
(¢€
(¢€
(¢€
(¢€
(¢€
(¢€
(¢€
(¢€
(¢€
(¢€
(¢€
(¢€
(¢€
(¢€
(¢€
(¢€
(¢€
(¢€
(¢€
(¢€
(¢€
(¢€
(¢€
(¢€
(¢€
(¢€
(¢€
(¢€
(¢€
(¢€
ñï□YÁ¨|lñÕ•Ò□·¸³ò¤CüJÛ□'¯a®;DðTÚOÄ=sÄÏz'E¨Æ¨•□ §ÝÎN□Ûýh□Í|-ã >□è~-
ðÎ£&o4—gÓƒ□õ»ÈQ□é'□Ž¸fô©nü6ú□ìãªÏt Ô5#□åË7-
óM-ÐIç…Áç¹5ØøËáM§‹|g¦ë¯p'E□Õ½€¦~Ðªr9õ#å>ÀWKã□

IâÏ□_èpÜ%³Ü„Û#.à»]_ ÿ w• xßÄ¥□þ□ü<□eò-š□D′7ÝFò‶i?A°°k□‡Ÿ□¯-
e·ŸÇë$□!GC□Ã)□#ñ□»;Ï□Xjþ ²ð¶¨æTµ¶Š%ž1µ-DP¡×9Ç□À‘\d?
|mk□éöß□nãÓÐ□MªáÕG@>n□°4□¥□□à÷Â¯/RuÕ$Žb"□Wj¼ŽÙUç8□□çØ×1â□Gâ¾-àýBòûOÒ¬´©
m□å‹•þQ\′□bAÇ¯>Õè7¿□mµ/‡‘øNÿ Q°¹1•Ë}/Í
‶q`Ø'§$c=8Ïzã¦ø=âmFÀéš§ŽîgÓ£B°À□ˆ$□‶p-
Ð□-ý01Ö€.xgŸÙ²Oû□^□9+{àçü′□□ýÙ¿ôsÖ—†ü"šGÃè|+}0¹•ìòÁ3Æ6îY
□•N□□ËøOá–¿á}^Ð/Œ.$Ðí%i□ÅU”>sò°ÝŒdç¿=²s@-ŸEqÒxGU□‰±xœkÓ□9!(tï›i;
ã®1‶»§_Î»□ òmwþN[Ãö
□ý□â□Œ?ò2ø□þÂgÿ BŠ°•CÁs^üRÒ¼^/Qa±´ksnP-rDƒ9Ï□÷Ÿ§½-5ð\Þ+Õ<=y□ê[•*ïÏud-æ

©Àçfòþ´□□•|
sâyôíWHÔ¿³µ½5‹[LFQ³ÎÖî9□pz□AÏ□~□ñÇˆ-¼e□ƒ|ea□j□Dd¶»¶?,Ê□9#§;[‘ŽF1Z^4ðV»¯
k□j°□Šî´‰¢€BÐ¦ï-ðÄî8#ž{ƒÐzU_
|5¹Ò|H<Iâ□z}gVHÌP³,□□A□□'<□;□“Å cXC□¿´¾ªÒFŽÑéÈÈXd©Ù□Èô8$~4ÿ •
j□ßƒ¥□□.¨□·p \ÿ !ùWSkà¹-þ)^ø¼Þ£Csh°
}‡r□□g9Æ>_Ö□□x._□¾ŠÑ^%·ö}à¸mÈ[xã s×Š ì+Í|cã•fÛÆ□ø_Â□E½î´a□O4ü,IÔ

£±Ï'□Ž 5éUç¾2øks®xŽ/□è:ä°>®±ˆÝÑIY à□,□8àõ□□Ç¨□žkgÆŸð²¼□Þ/□*Æý>Î-
qÓÌMÙÇá]OŒ!Š□Ú□Â 4i"‹BÛ\dd□□?P@4-Ÿ□uaâ}#Ä□ŸŠ¥Ôom.ViŒÑ“”R
¢□ÜsŸnzqÏ[«ø.mOâF‹â•½HâÓàhž□„³çv□9ÿ kô

/□□-ðÝ˜€J^BÊ}□#ù□X_□˜6…à□µœéíq□°'§Ý^¿†êô^>□—Æž□"H†é-
¤iRA#©aòžœTž$ðe□Š<¯"š□û°□¢yS ù£'F□□ùŒz□õ
þ'òG„µ□Ëò~Å6íøÛ□‡®k□ø□ÿ $ÆÏþ»Ëÿ ¡V*ü□×ïí—
MÖüqyu¤D1□²+□Øû¹Ë□ 8õé□Šî¼□ái|□áH4y®RæHäw2"•1Î9
>0ð½·Œ<5s£]JÐ‰pÑÌ£&7□ ã¿¸ô'§Zó†ñ/□~□ýŠ□□-¶¯áö'mÒú□‰c□à-™!A8 ç-
z½□Æž□¼ñF□□□†³s¤\Å:Î—□ùÉ
□´àƒ□›×¨□ÄÅð"[Õ¯mO‹ü_qªØ[H$[@□□□êIãÐœ□,y□□□ñ~ßP½ø›á+kKábe@-
nXec˜É×ë÷?J»©|1ø•«éòØj-:K‹Y€□Dñ6□□□ú□]ßŽ|□§øçJŽÖîG·¹□‹Û]F2Ñ1ëÇpp22:

kŒ

<q:}’çâ=ïØÇ□@ûÈô?0ÏâM iëÚã|/ð&…¥¬
«jÇmµ¢²áYÀûØë□□099□+†ø‡7ÄëŸ□ÝËâ;].ÛK,†X¡*]Ná·□žøï^□âß‡vÞ)ð¾□¥>¡q□Þš□ì×Í
ó¾åP¤·Lç žG jãu/ƒ~&×¬^-oÇ□Þ4@}š7FhÃgï6O\dtÏ={□□□□sû9Çÿ `ë□ý
*±â□p¿³¤fÛw™ý‹j

Þ»vÇ»ÿ □Ít- ðl°ÇÃ1á8¯□9VÚÞ□pÈJ“□Cœg¾ÏÖ¶tÝ□□_
Zh7{.¡ŠÉ,åÊàJ¡□-=

(□□áx´□4/±mò¾ÏómþþNÿ ÇvkŽÓ,ŸÚ_S6XØ4ñö½½3±?®ÏÆ•~□øƒEšh|-
ãk½?M™Ë}□□ÊgÐƒ,}ð□uþ□ð□—
,¡¹”]K□©Þ6ë«Ù†□ùÎ ÉÀÉÉä’zž˜ ãþ□□h|LÓÿ ÈPßÿ ¤îûØç□üwÑñ +xƒÁ+m•í#¨~ë□qº?ë
Œ~5§â…73x’□□xWÄ□è—×$›„@JHÇ’x#©ä,

O<TÞ□ø]&□â$ñ□ˆõË□sU‰q□J□H°ò2Nq"Ž€g¦p@□?€Ê7Æÿ □□¯øý□□îëån□=±²•ñ§Êþ×ð^vyß
Ú<tÝ·)Ÿ|g□³ã-
†Rë~!O□è:Ì°6²□$' %e `□,□8àõ□□Å`¿ÁmFëQ±Õõ/□Í□ªÃu□'É:□¾Rœì^sœý□=(□ØkÈþ0□-
Œ¾-Êÿ ê"QmÄôûðŸèk×+Ìþ8é□ßx□u□`|ý.å.r£$'Ý8üJŸÂ€=-êÖÞöÖKk¨#ž
WkÅ*†V-„-µä-4²Ó-u8| à□2ÒÏUÕ†5
ˆ# ÁmÔ†n □2Fzvù…zv™«¾µáK}_NHÞ[«A41»aw•ÈV#¦□ƒ\çÃÏ□Ýè-
oÖuéRçÄZœ…î&S¸F™ÈE>•Î0:

Â€8Ÿ

xj?□x¯[ðÒêz•Ž•áèÒÚÖ
Iü¢Ò□î•±÷˜°cÓ¸ì1]wÁÝ{QÖü:j—
□ssaxö¢w9i□B□X÷<‘ŸaNÕ<□¬CâËï□x[^□M›QŒ%ÜS[‰T□1½}øÏN¹çœVŽ…¢iÿ

¼ r¢f•-RK»™˜`Í&Ü"ŽÙÀ {

´□É|&ùü□ħ□D?»:–AõÌ‟□þ}ëÖëÌ~□is[ø6ãW°é□µÛÜn=J

□□□çñ¯N □Š(□Š
(□Š(□Š(□Š(□Š(□Š(□Š(□Š(□Š(□Š(□Š(□Š(□Š(□Š(□Š(□Š(□Š(□Š(□
Š(□Š(□®Gþ-~•N®Gþ-~″ ê(¢€
(¢€
(¢€
(¢€
(¢€
(¢€
(¢€
(¢€
(¢€
(¢€
(¢€
(¢€
(¢€
(¢€
(¢€
(¢€
(¢€
(¢€
(¢€
(¢€
(¢€
(¢€
(¢€
(¢€
(¢€
(¢€
(¢€
(¢€□7ú¦ª•noõMU(¢Š(¢Š(¢Š(¢Š(¢Š(¢Š(¢Š(¢Š(¢Š(¢Š(¢Š(¢Š(¢Š(¢Š(
¢Š(¢Š(¢Š(¢Š(¢Š(¢Š(¢Š(¢Š(¢Š(¢Š(¢Š(¢Š(¢Š(¢Š(¢Š(¢Š(¢Š(¢Š
(¢Š(¢Š(¢Š(¢Š(¢Š(¢Š(¢Š(¢Š(¢Š(¢Š(¢Š(¢Š(¢Š(¢Š(¢Š(¢Š(¢Š(¢
Š(¢Š(¢Š(¢Š(¢Š(¢Š(¢Š(¢Š(¢Š(¢Š(¢Š(¢Š(¢Š(¢Š(¢Š(¢Š(¢Š(¢Š(
¢Š(¢Š(¢Š(¢Š(¢Š(¢Š(¨n-`¾³šÒæ5- ãhäFèÊF□?•©¨

Ï□è□>□Ñ¡Ò´Õ'm!,QdränbÇ"îMiÑE □›¯èV^%ÑgÒu□'-'Û¼Få
Ã□□□p+JŠ ,ÊÎßO±,ÊÖ1□½¼k□H:*¨À•©è¢€
(¢€
(¢€
(¢€
(¢€
(¢€
(¢€
(¢€
(¢€
(¢€
(¢€
(¢€
(¢€
(¢€
(¢€
(¢€
(¢€
(¢€
(¢€
(¢€
(¢€
(¢€
(¢€
(¢€
(¢€
(¢€
(¢€
(¢€
(¢€
(¢€
(¢€
(¢€
(¢€
(¢€
(¢€
(¢€
(¢€
(¢€
(¢€
(¢€
(¢€
(¢€
(¢€
(¢€
(¢€
(¢€
(¢€
(¢€
(¢€
(¢€
(¢€
(¢€
(¢€
(¢€
¹úµúU:¹-
úµúP□¨¢Š (¢Š (¢Š (¢Š (¢Š (¢Š (¢Š (¢Š (¢Š (¢Š (¢Š (¢Š (¢Š (¢Š (¢Š (¢Š (¢
Š (¢Š (¢Š (¢Š (¢Š (¢Š (¢Š (¢Š (¢Š (¢Š (¢Š (¢Š B□

-”ß&?îÓè □y1ÿ v•&?îÓè □y1ÿ v•&?îÓè □y1ÿ v•&?îÓè □y1ÿ v•&?îÓè □y1ÿ v•&?î
Óè □y1ÿ v•&?îÓè □y1ÿ v•&?îÓè □y1ÿ v•&?îÓè □y1ÿ v•&?îÓè □y1ÿ v•&?îÓè □y1
ÿ v•&?îÓè □y1ÿ v•&?îÓè □y1ÿ v•&?îÓè □y1ÿ v•&?îÓè □y1ÿ v•&?îÓè □y1ÿ v•&?
îÓè □y1ÿ v•&?îÓè □y1ÿ v•&?îÓè □y1ÿ v•&?îÓè □y1ÿ v•&?îÓè □y1ÿ v•&?îÓè □Ì
Q□’ □Þ™›oï§ýõO¸ÿ •i□Ü?Ê¹Ú ßÍ·÷Óþú£6ßßOûê°(□üÛ□}?ïª3mýôÿ ¾«□Š ßÍ·÷Óþú£6
ßßOûê°(□üÛ□}?ïª3mýôÿ ¾«□Š ßÍ·÷Óþú£6ßßOûê°(□üÛ□}?ïª3mýôÿ ¾«□Š ßÍ·÷Óþú£
6ßßOûê°(□üÛ□}?ïª3mýôÿ ¾«□Š ßÍ·÷Óþú£6ßßOûê°(□üÛ□}?ïª3mýôÿ ¾«□Š ßÍ·÷Óþú
£6ßßOûê°(□üÛ□}?ïª3mýôÿ ¾«□Š ßÍ·÷Óþú£6ßßOûê°(□üÛ□}?ïª3mýôÿ ¾«□Š è„q0È
•Pi|˜ÿ »QXÿ Ç”_OëV(□žL□Ý£É□û´ú(□žL□Ý¤hâQ- □RjJ©©□Ç“}Gó
3mýôÿ ¾¨Í·÷Óþú¬
(□□6ßßOûêŒÛ□}?ïªÀ¢€7ómýôÿ ¾¨Í·÷Óþú¬
(□□6ßßOûêŒÛ□}?ïªÀ¢€7ómýôÿ ¾¨Í·÷Óþú¬
(□□6ßßOûêŒÛ□}?ïªÀ¢€7ómýôÿ ¾¨Í·÷Óþú¬
(□□6ßßOûêŒÛ□}?ïªÀ¢€7ómýôÿ ¾¨Í·÷Óþú¬
(□□6ßßOûêŒÛ□}?ïªÀ¢€7ómýôÿ ¾¨Í·÷Óþú¬
(□□6ßßOûêŒÛ□}?ïªÀ¢€7ómýôÿ ¾©GÙÉÀd$ö□\ýMiÿ -
q□¼(□wÉ•û´y1ÿ vŸE 3É•û´y1ÿ vŸE 3É•û´y1ÿ vŸE 3É•û´y1ÿ vŸE DÉ
}í«ŸSŠnm¿¾Ÿ÷ÕTÕþì_Sý+.€7ómýôÿ ¾¨Í·÷Óþú¬
(□□6ßßOûêŒÛ□}?ïªÀ¢€7ómýôÿ ¾¨Í·÷Óþú¬
(□□6ßßOûêŒÛ□}?ïªÀ¢€7ómýôÿ ¾¨Í·÷Óþú¬
(□□6ßßOûêŒÛ□}?ïªÀ¢€7ómýôÿ ¾¨Í·÷Óþú¬
(□□6ßßOûêŒÛ□}?ïªÀ¢€7ómýôÿ ¾¨Í·÷Óþú¬
(□□6ßßOûêŒÛ□}?ïªÀ¢€7ómýôÿ ¾©Ê°¿Ý*Øô9®z´´•½/Ð□Ž Ñòcþí-L□Ý§Ñ@

òcþí-L□Ý§Ñ@

òcþí-L□Ý§Ñ@

òcþí-L□Ý§Ñ@

òcþí-L□Ý§Ñ@

òcþí-L□Ý§Ñ@

òcþí-L□Ý§Ñ@

òcþí-L□Ý§Ñ@

òcþí-L□Ý§Ñ@

òcþí-L□Ý§Ñ@

òcþí5-□8bª}Û□-dêßëÓýßë@□ómýôÿ ¾¨Í·÷Óþú¬
(□□6ßßOûêŒÛ□}?ïªÀ¢€7ómýôÿ ¾¨Í·÷Óþú¬
(□□6ßßOûêŒÛ□}?ïªÀ¢€7ómýôÿ ¾¨Í·÷Óþú¬
(□□6ßßOûêŒÛ□}?ïªÀ¢€7ómýôÿ ¾¨Í·÷Óþú¬
(□□6ßßOûêŒÛ□}?ïªÀ¢€7ómýôÿ ¾¨Í·÷Óþú¬
(□□6ßßOûêŒÛ□}?ïªÀ¢€7ómýôÿ ¾¨Í·÷Óþú¬
(□□6ßßOûêŒÛ□}?ïªÀ¢€7ómýôÿ ¾¨Í·÷Óþú¬
(□□6ßßOûêŒÛ□}?ïªÀ¢€7ómýôÿ ¾¨Í·÷Óþú¬
(□□6ßßOûêŒÛ□}?ïªÀ¢€:!□dd Aî□/"÷i-Ÿñé□û¢¦ □H•N
>…©3mýôÿ ¾«"ûþ?eú•åUè□□6ßßOûêŒÛ□}?ïªÀ¢€7ómýôÿ ¾¨Í·÷Óþú¬
(□□6ßßOûêŒÛ□}?ïªÀ¢€7ómýôÿ ¾¨Í·÷Óþú¬
(□□6ßßOûêŒÛ□}?ïªÀ¢€7ómýôÿ ¾¨Í·÷Óþú¬
(□□6ßßOûêŒÛ□}?ïªÀ¢€7ómýôÿ ¾¨Í·÷Óþú¬
(□□6ßßOûêŒÛ□}?ïªÀ¢€7ómýôÿ ¾¨Í·÷Óþú¬
(□□6ßßOûêŒÛ□}?ïªÀ¢€7ómýôÿ ¾¨Í·÷Óþú¬
(□□6ßßOûêŒÛ□}?ïªÀ¢€7ómýôÿ ¾¨Í·÷Óþú¬
(□□6ßßOûêž"ŒŒ€□>õÎ×Eoÿ -Ñ□¸?• /"÷hòcþí>Š g"-
÷iŸèÿ ÐOûê¦®mÿ Ö7ÔÐ□îm¿¾Ÿ÷Õ□¶þú□ßU•E oæÛûéÿ }Q›oï§ýõX□P□þm¿¾Ÿ÷Õ□¶þú□ßU•
E oæÛûéÿ }Q›oï§ýõX□P□þm¿¾Ÿ÷Õ□¶þú□ßU•E oæÛûéÿ }Q›oï§ýõX□P□þm¿¾Ÿ÷Õ□¶þú□ßU
•E oæÛûéÿ }Q›oï§ýõX□P□þm¿¾Ÿ÷Õ□¶þú□ßU•E oæÛûéÿ }Q›oï§ýõX□P□þm¿¾Ÿ÷Õ□¶þú□ß
U•E oæÛûéÿ }Q›oï§ýõX□P□þm¿¾Ÿ÷Õ9R□ÎÝ-□Cšç«OHé7áýh□CÉ□û´y1ÿ vŸE 3É•û´y1ÿ
vŸE 3É•û´y1ÿ vŸE 3É•û´à □□)h □Š(□Š(□Š(□Š(□Š(□Š(□Š(□Š(□Š(□Š(□Š
(□Š(□Š(□Š(□Š(□Š(□Š(□Š(□Š(□Š(□Š(□Š(□Š(□Š(□Š(□Š(□Š(□Š(□
Š(□Š(□Š(□Š(□Š(□Š(□Š(□Š(□Š(□Š(□ ¼¼¶Óìå¼¼ž8-¡RÒK#□U-¤Ôõä□¦ŸP—
Âž□ŽVŠ□cP 3)ç□'TQ™3õQ@□KñÇÀMyö□iiBç□cm OåŸÒ½□Þâ□»h®m¥I •CÇ$l□]O
,:Šæï>-x^ëÃrh‹¢ÙEnb1Æé
ïŒã□Ãc;»ç9=ó\□iý¨Ïsà;›
‡,l/^(Áþ□ 6?ï¢ß• zÅ□Q@□□Q@□□Q@□□Q@□□Q@□□Q@□□Q@□□Q@□□Q@□□Q@□Ü□Ç´¿îå\ítW-
ñí/û‡ùW;@□□Q@□□Q@□□Q@□□Q@□□Q@□□Q@□zïŒ<?á§DÕõHm¤q¹cÁw#×j,qïŠ"BñN‡âTvÑõ□n
¼¾]W*Ê=J°□□|W□osgá?ˆzÝ×ˆâ□&§$ocªJ™Œ(\□‹ÿ □-øÎ>•cLŠßÄ?□¡ñ

‰oåé¶−²C=êÇ±/□¸
¿ß
×w¶=(□wTñ¾□¤jRØÏeªÉ$XËAbî‡ -□

-µGþ□n†²Â"ZêÐyÒ,HÓX:)f8□&»:á¯•ü$ß□-4ñóXx}□ÝÇ£\¸ýÚŸ÷F[ó

æ¨júÞ™ Ùý¯T½†Ò

à4••ÇĐ

¤û
¿^{q□¾©ñ-mõ4Ic³Ò„Ö0Ê2»ËüÎ□ê□ÃÚ€:□□Ç>□ñ□ÑµÒµhg¸Á""-□6:à0□ü+¡®□â-”6þ□_□Á□
G©i7□Ï□Ê0ØÞªW=Ç==«¼□Ä±$‹Ñ€aøÐ□¨¢Š (¢Š (¢Š (¢Š Þ±ÿ •(¾ŸÖ¬U{-
øò‹éýjÅ □QE □SRÿ •&ú•çVê¦¥ÿ -MõÎ€1(¢Š (¢Š (¢Š (¢Š (¢Š (¢Š Bp
ç•Jãÿ ádé?ô

×?ð['øWcHHPI □É'µ sZ?Ž´•kX]*□¯á»xÌª—6-□Tu<Õ-wÆ-ðÓ¢jú¤6Ò8Ü±à»‘ëµA8÷Åaø
□{YÖ<a(&;©>Ç§çµ´g□ï6OáY¶÷6~□ø‡-ÝxŽ!□jrFö:¤©˜Â…Á^¿ð□ïŒãé@□-
…â□□Ä¨í£ê0Ýy|°®U”z•`□-ø-ŠóÝ2+□□üJ‡Ä:%¿—¦ÚZÉ

÷«-Ä¼và*ÿ |/]ÞØô¯B

{^ñÏ†ü1{□ž±©
k‰#□ªy2>T’3ò©î□åQèŸ□</â-Etý+SûEÓ)qÙåN□^Y@®s[ñ&“á□‹¦ïX»û4□hI□7-ï-óØã
è□uÚ‹t□n□F§□Ë ËF2®□®Ö ãß□□µE□P□E□P□SZ□ÇÜ_ï
†¦´ÿ •¸¿Þ□□ÐQE□ QE□ QE□ QE□□›«ýØ¾§úV]jjÿ v/©þ•—
@□□Q@□□Q@□□Q@□□Q@□□Q@□□V□□u™<?àÍWS„âha"#èìB©ü

‹Xñï…´
£k©k□Ep¿z4V‘—ê□□~5«¤k:v½`·Ú]ÜwVÌJïLðGPAä-
c\□‡üKà□□éÐØ6¯j×ì ÝÜ$m+K)å‹:©ïžüWAâ□JØ|:Ö5?□K□+ÚË*MkŒg□-‘üCóâ€□¨|GðŽ—
|ÖwzÜ
:6ÖTW•)ô%A ×Ee{k¨ÙÅweq□Å¼£rK□□V-
Zçü□¥iVÞ□Òã²·□¡žÒ7•¶ƒæ³(,[Ôç=zt¬Ÿ□Äš?‹ü]áÛl-•¼ðÝ[Æ:Gæ¦YG □

(□½¬Ýk_Ò¼;eö½Zú+Xs€_$±ôU□'ð□¥^□ã¯

éö¾□ñ.°âK-Bkw+=Ëo0¡?r1Ñ□{sêM w□O□Í¼SÂÛ¢•□£c□□d□'³ôù□tÏúô‹ÿ @□¡@□ii-
z_ þµ›ZZGÞ—
è?- jQE□ QE□ QE□ QE□ QE□ QE□ QE□ QE□ QE□ QE□ VN-þ½?Ýþµ-Y:·úôÿ wúÐ□}□Q@□
□Q@□□Q@□□Q@□□Q@□□Q@□Ïq□-¼"ÜJ'C□-
y$`ª w$ô®^□‰ž□¹¾[8µë□9›hÜ®¨Oûä□ýk7âyY£ðæŸtÅtÛÍZ(îùÀeä…cèOò®£Vðî™«h□i□Z@
¶-□DQ□□.8+èG^(□T□□'@□œ×'?Äÿ □[]›i5è

€à”GuÏûÊ¥□ŽÄðùÔ¼Qð;ì°J□΄-Ò[UbØÝ±Š□Ÿu gÞŸ¡ø»Âv-□‹L»µ[K»x- ô‡¶&g“□
&>}Ç¿¿8 □@µº·½µŽæÖhç,UÜ’FÁ•‡¨"¥®KáÎ•w¢xPAy
¶i®%¸ŽÔœ›ts•O¨□þ5ÕK□M
Å Ê:•aœd-□ s□¿□¼□axm.5Û□8□¤F¯"ƒîÊ□•t-·v÷Ö±ÝZO□öò®ä’6

¬=A□ÌK«x□Ãzt°_Út˜b‹1½ŒE$rzm1Œ³7Ôf¢øe¥Ýé^□1Ý[Éj³]K<□Òýèbcò©□•|{Ð□eE□P□E□
P□E□P□E□P□E□P□E□P□Aiÿ -‘□º*j†Óþ="ÿ tTÔ□ƒ}ÿ -
²ýGòªõbûþ?eú•åUè ¢Š(¢Š(¢Š(¢Š(¢Š(ª×ú…ž—e%åýÌVÖÑŒ´’°P*Íy÷Œc‡Pø•á□/R
úkùó□¤û’Ì«ò,:□z{ã½ mi□□ü'¬ß-□Žµ

_
.v¢:<{• , 'ØWEuuoem%ÍÔñÁ□csÉ#□U-¤ž•Ì|BÐìµ/□êEá□f³·{‹i@□¢t□†ÓÛ8ÇãVôÈm<]à
½&]ZÙ.’âÞ□‰#“î™6ƒÈî3Øñ@□ìþ$xBûP[□}rÝ§fÚ¡•‘Xú□)üë©®7âMž”¿

µAwm Ž□?ÑÆÀ6IÑ6úrGNÕÐø}nSÃzZ^-7kg□˜ ·]û□iûç4□£\æ±ãß
h□F×RÖ Šá~ôh-#/Ô 8üj_□ë2x□Ãš®§
ÄÐÂDGÑØ…Sø□□Éx□À¾ ðn•□ƒjö-~Ê□ÝÂFÒ´²žX³ªžùïÅ w°F³§kÖ
}¥ÝÇulÄ®ôÏ□u□-Aö5z©iW□måŠŸéOo%¬ä¸’ß□\ô'Žü`÷â®Ð□E□P□E□P□E□P□E□P□]□¿ü{Eþà
þUÎ×Eoÿ -Ñ□¸?• IE□P□\Ûÿ ¬o©®’¹·ÿ XßS@□¢Š(¢Š(¢Š(¢Š(¢Š(¢Š(□šÕ<o§i□”¶
3Ùj²I□2ÐX»¡È□†□□-Qÿ …›¡¬°¤Ö°´-t‹□4Ö

ŠYŽ É®Î¸kãÿ 7ÄËM<|Ö-Awqè×.?v§ýÑ-
üè□ªÕõ½3A³û^©{□¤□Ài□□• □Iö□›¢xçÃ^"°6°V-

÷$DU£fÇ\?…s×ÛêŸÖßSD-;=(Mc

£+¼¿Ìà-§ü=ªOŠ¶PÛøa|C□i-¥¤ÜC<□(Ãcz©\÷□ôö

úªjZ¥Ž□f×š□Ü6¶ëÁ’V
3éî}ªÌn%‰$^Œ□□Æ³u»□□â□o5¸íL□Oæ¤—l□q±ã'?/~ô□•¤üAð®¹|¶Z~³

·,p±²<e•¢î□'é]-y—•îô•□¦•¥xvH5□]nâ’;‹<:Ú"Ÿ™ÚEà

vÏ'□W¦Ð□E□P□E□P□ZzGI¿□ëY•§¤t›ðþ´□§E□P□E□P□E□P□E□P□E□P□E□P□E□P□E□P□E
□P□E□P□E□P□E□P□E□P□E□P□E□P□E□P□E□P□E□P□E□P□E□P□E□P□E□P□E□P□E□P□E□P□
E□P□E□P□E□P□E□P□E□P□E□P□E□P□E□P□E□P□E□P□E□P□E□P□E□P□E□P□E□P□E□P□^9ñ½Î™®ø-
Ä□#□K□G30□ÇÍ□•ù#þUìu—â□□é¾(Ñ§Òµ[q5¬¸ÈÎ□HèÊ{□þx
¯µ‹□?D›Xžæ1c□>y˜0*ÉŒ,□|ñŒuÈ¯2ýŸ,¦□Á□Ú„ÈT^ß»Æ{2€□Gü
pü)‘~Ï°□Ê‘Ï-ë□Ø#n[V•@üN;÷À□ê°~Ÿi¥iðXXÀ□ZÀ□"‰□
□ùë@□h¢Š (¢Š (¢Š (¢Š (¢Š (¢Š (¢Š (¢Š (¢Š (¢Š Žãþ=¥ÿ pÿ *çk¢¸ÿ •i□Ü?Ê¹Ú
(¢Š (¢Š (¢Š (¢Š (¢Š (¢Š ó¿□ÚÛø÷PÕu□u•Ô6WòZÛiŽß¹„&0ÎŸÄç'-
Ï·³f³O□xÛC´Ñ''tíbY"ŸLg,‘□3æF□+×žßÓkTø{¤j:¬°□½Ö¥¥_MÌÓi·&□!õa,3øTú□□ô□
P:□’òûQ+°^_Îf‘WÐ□Àü¨□[[Õ ÐôKÍRäþæÖ&□Œýâ:□îN□ãX_□t©ì|6/¯ÇüLµYZúè‘Ègä/¶□□
væµ¼Cáë_□ØEc}$Ël“¤Ï□l —iÈFÈ9\ã8ÁàsZÔ V□ˆü-
¥x□-å¼□ÃwoŸ"îÖS□±ý□Ž›TðÕž-®iZ´òÜ-
Æ˜ÎÐ¬n□1`□Ü1ÏNÄUm□Äöšýìw-¨ê¶□QÇåy°}Ñˆ•É -□êOnô□ÀøãÁZm®•k§□jjú¦³¨ÜG□•¿½i
Lcp.áF□□f’G□¯^D□¢¢ýÕ
ç´□é□□ãßÅö›ÍA×k^ßLf—o '§à+£ □Š(□Š(□Š(□Š(□ë-
øò‹éýjÅW±ÿ •(¾ŸÖ¬P□E□P□U5/øòo¨þunªj_ñäßQüè□□Š(□Š(□Š(□Š(□Š(□Š(□¹□ˆ
š•Ä□

z=ƒcRÖ¦□PcøC}÷ú□Ï=²+¯¬™ü=iuâ{Mzi&{‹H□□"$yi¸üÎ□3¸Ž:ôí@□ô½:ßHÒ-tëUÛ□´K□}
ÆO¿zá¼?koãÝCUÔuÖ7PÙ_Ékm¦;~æ□˜Ã:□□œž[>ÞÞ‰\ž©ð÷HÔuYu;{-KJ¾›™¦ÓnL&CêÃ□gð

Y¬ÓÀÞ6Ðí4Iä];X−H§Ó□Ë$d

ù‘ƒÊõç·ôôjæ´/□é:□ u!%åö¢W`¼¿œÍ"¯ '□ùUÇðÕ›ø¶?□□n>Ù□±¶□‡-^Ü“œc9ç×-Ô□°UKn*
7tÎ+Ï|kgimão□\éÑG□±. □C□□¤·Çï7c¨□×ÔÖÆ©à□□KUŸR‡UÖ´Û›,

¦ÂôÄ□€□HÁì□YÐ|□¤ø~òKè•ÍÞ¡"ìkËÙŒÒíôÉéø
è¨¢Š (¢Š *kOøû‹ýáPÔÖŸñ÷□ûÂ€:
(¢€
(¢€
(¢€
(¢€3u□»□Ôÿ JË-M_îÅõ?Ò²è ¢Š(¢Š(¢Š(¢Š(¢Š(®Câ•¤—
¿□µ¨¢RÌ±,¸-ˆêçôS]}5Ñ%□£‘UÑ□VV□□- Ð□W‡âÒ§ðåœšu-ªY\@®©□j□,:□?Zå>□[Û□¯□[Ù¢
6Šu6H□

ÆNßÞ6ô-˜«GáfŒ†Híu=nÎÆBKXÛ_€ç¨ÛŒãñ®šÛ@Ó,´¢ZÚ¬:y‰¢1FHùXyë""Îs@Ïü+"
ØÉöc]Óm-k[Kò••^'•Røg¥XA«x—SÒQ†—5ÄvÖ®Î_ÍòÔï}Ç'
1æ¯Â¯Ó$-UÎ³â
«.ösê

a#Ó □•Æ»
;;m>Î+K8□
xWlqÆ0□P□õÌ|Fÿ ’y®□ʞ«□J½¡xjÏÃÓjRÚKpí¨\µÌ¾s†
Ç²ð0?3ïXÚÏÃ¸5Ë‹·°ñ'‰□
¦%í#¾□□□ð„*F=¨□¢Ð□ä]Ó?ëÒ/ý V…s^-ðrxvïÎ•^×oPEå-½õç™□Ž0Bí□#□-Ä×K@□ii-
z_ þµ›ZZGÞ—
è?- jQE□ QE□ QE□ QE□ QE□ QE□ QE□ QE□ QE□ QE□ VN-þ½?Ýþµ-Y:·úôÿ wúÐ□}□Q@□
□Q@□□Q@□□Q@□□Q@□□Q@□úÖ‰§ø‡K—NÔíÖ{i0J’A□t ŽA□Äêþ□Ðt-
□â÷W×µë•2Ú2ÆÎçQo)½□ □ç □®Ë^Ðm|E`¶—
RÜÂ□A,rÛJc‘□□□□{□Xv□□ô^oa»Ô/u]bH□t#S»3*-
P¸□óÍ sÖ÷7þ□ø!k,H"¿e□t‹Ä&i3¸•öCt=Åt)ðãÃ·:~/c–
þò\;êRÌÆáŸ³+çå-€qìk¨¿°µÕ,f±½□'¶™vÉ□Ž□W□¿
´è“È·×|GogÐZE¨‘□-˜ÆqøÐ□ß□µ;ë„Öt«ËÆ¿□MëZÃxü´©Ž□-ì:□]„ÓEo
Í4‰□Q©gw`□@êI=□QÒ4-;AÒ—MÓ-…½°Ï
NI=X“É>õODðž›¡xmô□¼ë›□<ÀërÁ‹□ûÀà

9 ½ð‡†uo2[-N™çùža‡|÷ÞsïšçþM,7^"ÑÒêK-3M½ÙË#ï*¤s-îáx•<|-
Ò¡Sž±¯ÙÙž¶vúƒ,XôÁãñ®§FÑ4ïé±éúe²ÛÛ'!FI$õ$žI÷4¡EPEPEPEPEPEPE
PAiÿ -‘*j†Óþ="ÿ tTÔƒ}ÿ -
²ýGòªõbûþ?eú•åUè ¢Š(¢Š(¢Š(¢Š(¢Š(¬xgKñE’Zêp³ß|RÆÅ$‰½U‡JØ¬OøbÓÄk
ng»¿´šØ±Š{+ƒ®ìdg¡è;v

□Æ~

Ò4O
ÝÍ}¬kš¤ò!†ÆÖöý¤□;p›P□’ □-ÕßèðEáÏ□ØÛÞL‘Gcg□M#°
»T□$þ□³tŸ i□^¥-¥4÷ú¥ü_ê®5+“3Gþï@□¾3WüSá‹O□èÿ Ù—×□PÀdY ¶p¬HÎ□È
ŽsÓ°

zÒ ü□«[j ·p¼>□³‟Í± ·•a¯$□&qÙ□ðƒ×©ô®î¸•ðÜ(xÏÆ

□§Oüv·ÓÃÑ□Â/.ƒ=þ¡u

°É
ÜÜM¾v□œ'øê3•Ç@(□#â•¤—¿□µ¨¢RÌ±,¸-^êçôS[-‹JŸÃ-
riÖ¶©eq□°¤Q¨R□è@ýj}+GµÒ4;}"□òÚÁ□'<ò□²ÿ µÆ□å\¹øY£!';]O[³±□'Ö6×Å`9ê6ã8üh□
¿ÃHáŠÿ Åi§í□JêŒ-
,}ÀØùÂöÇÝé^□Tô½*ÇEÓ¡Óôëd·µ^a#_æOR}Í\ □Š(□Š(□Š(□Š(□°+□øö‹ýÁü«□®Šßþ=¢
ÿ p□* 'Š(□¹·ÿ XßS]%soþ±¾¦€□E□P□E□P□E□P□E□P□E□P□E□P□□oVƒCÑ/5K"û›XšB3÷^è
□¹8•a|=Ò§±ðØ¾¿ñ2Õekë¢G!Ÿ•¾Ø\qÛšÖñ□‡-|Ka□□ô"-²N"<q°□]§!□ åsŒã□•ÍkP□□ˆü-
¥x□-å¼□ÃwoŸ"îÖS□±ý□□Žói□x+MµÓ-tïíM_TÖu□^à²□÷-)Œn□Ü(ÀÀPrHã5ßkþ□´×ïc½mGU°
°Ž?+ÍÓîŒD®I ðGR{w¤Ð|□¤h□•□□Úo5□]-{}1š]¾€žŸ€

…□FŠ‹÷T *½âX]¯öuòÛL.□Ÿ³N□¼Å\gå=@Èý*‡^|5gâXlâ½–
â5´¹K”ò□),¹À<-9úûÓ|Gá=+Å□@5□¤□Û’ÐO

†9"'□*GĐuô

SÇÞ□Ð´○

]k°e¼□>¥b□-
÷6j!%²□Ò□ ƒÓ□Ww¥O=Ö□eqr›.%·□å\ck□□□Î¹‹o†°B^Cq¨ßêúÇ□Ûâ□R¼3"□Ð…À□ñÍvT QE□
 QE□ Vž‘ÒoÃúÖeié□&ü?- iÑE□ QE□ QE□ QE□ QE□ QE□ QE□ QE□ QE□ QE□ QE□ QE□
QE□ QE□ QE□ QE□ QE□ QE□ QE□ QE□ QE□ QE□ QE□ QE□ QE□ QE□ QE□ QE□ QE□ QE□
 QE□ QE□ QE□ QE□ QE□ QE□ QE□ QE□ QE□ QE□ QE□ QE□ QE□ QE□ QE□ QE□ QE□ QE
□ QE□ QE□ QE□ QE□ QE□ QE□ Ù□Ì•Ó8Ü□Ígÿ d□ùì□ïšÒ¢€3□²□üö?÷ÍÙ□þ{ûæ´¨

ßì•ÿ =•ýóGö@ÿ žÇþù-*(□7û □Ïcÿ |Ñý•?ç±ÿ ¾kJŠ ÍþÈ-
óØÿ ß4□d□ùì□ïšÒ¢€3□²□üö?÷ÍÙ□þ{ûæ´¨

ßì•ÿ =•ýóGö@ÿ žÇþù-*(□7û □Ïcÿ |Ñý•?ç±ÿ ¾kJŠ ÍþÈ-
óØÿ ß4□d□ùì□ïšÒ¢€3□²□üö?÷ÍÙ□þ{ûæ´¨

ßì•ÿ =•ýóGö@ÿ žÇþù-*(□7û □ïcÿ |Ñý•?ç±ÿ ¾kJŠ ÍþÈ-
óØÿ ß4□d□ùì□ïšÒ¢€3□²□üö?÷ÍÙ□þ{ûæ´¨

ßì•ÿ =•ýóGö@ÿ žÇþù-*(□8"òaXó•½êJ(□Š(□¢¸ƒí□˜÷mÏ|T´P□oö@ÿ žÇþù£û
□ïcÿ |Ö•□□›ý•?ç±ÿ ¾hþÈóØÿ ß5¥E fÿ d□ùì□iš?²□üö?÷ÍiQ@□¿Ù□þ{-
ûæ•ì•ÿ =•ýóZTP□oö@ÿ žÇþù£û □ïcÿ |Ö•□□›ý•?ç±ÿ ¾hþÈ-
óØÿ ß5¥E fÿ d□ùì□iš?²□üö?÷ÍiQ@□¿Ù□þ{ûæ•ì•ÿ =•ýóZTP□oö@ÿ žÇþù£û
□ïcÿ |Ö•□□›ý•?ç±ÿ ¾hþÈóØÿ ß5¥E fÿ d□ùì□iš?²□üö?÷ÍiQ@□¿Ù□þ{-
ûæ•ì•ÿ =•ýóZTP□oö@ÿ žÇþù§Å¦□¥Y<Òvœãm_¢€
(¢€
(¢€
(¢€
(¢€+]Ú
 €¾Ý¹íš-ý•?ç±ÿ ¾kJŠ ÍþÈóØÿ ß4□d□ùì□išÒ¢€3□²□üö?÷ÍÙ□þ{ûæ´¨

ßì•ÿ =•ýóGö@ÿ žÇþù-*(□7û □Ïcÿ |Ñý•?ç±ÿ ¾kJŠ ÍþÈ-
óØÿ ß4□d□ùì□ïšÒ¢€3□²□üö?÷ÍÙ□þ{ûæ´¨

ßì•ÿ =•ýóGö@ÿ žÇþù-*(□7û □Ïcÿ |Ñý•?ç±ÿ ¾kJŠ ÍþÈ-
óØÿ ß4□d□ùì□ïšÒ¢€3□²□üö?÷ÍÙ□þ{ûæ´¨

ßì•ÿ =•ýóV-,Å©rvìvÅZ¢€
(¢€
(¢€
(¢€
(¢€
(¢€
(¢€
(¢€
(¢€
(¢€
(¢€
©ud.•XÉ·□□3Vè

ßì•ÿ =•ýóGö@ÿ žÇþù-*(□7û □Ïcÿ |Ñý•?ç±ÿ ¾kJŠ ÍþÈ-
óØÿ ß4□d□ùì□ïšÒ¢€3□²□üö?÷ÍÙ□þ{ûæ´¨

ßì•ÿ =•ýóGö@ÿ žÇþù-*(□7û □Ïcÿ |Ñý•?ç±ÿ ¾kJŠ ÍþÈ-
óØÿ ß4□d□ùì□ïšÒ¢€3□²□üö?÷ÍÙ□þ{ûæ´¨

ßì•ÿ =•ýóGö@ÿ žÇþù-*(□7û □Ïcÿ |Ñý•?ç±ÿ ¾kJŠ ÍþÈ-
óØÿ ß4□d□ùì□ïšÒ¢€3□²□üö?÷ÍÙ□þ{ûæ´¨

ßì•ÿ =•ýóGö@ÿ žÇþù-*(□7û □Ïcÿ |Ñý•?ç±ÿ ¾kJŠ ÍþÈ-
óØÿ ß4□d□ùì□ìšÒ¢€3□²□üö?÷ÍÙ□þ{ûæ´¨ □E•□Çœí□Í>Š(□Œúhšf"Í#wmµ-
ö@ÿ žÇþù-*(□7û □Ïcÿ |Ñý•?ç±ÿ ¾kJŠ ÍþÈóØÿ ß4□d□ùì□ìšÒ¢€3□²□üö?÷ÍÙ□þ{-
ûæ´¨

ßì•ÿ =•ýóGö@ÿ žÇþù-*(□7û □Ïcÿ |Ñý•?ç±ÿ ¾kJŠ ÍþÈ-
óØÿ ß4□d□ùì□ïšÒ¢€3□²□üö?÷ÍÙ□þ{ûæ´¨

ßì•ÿ =•ýóGö@ÿ žÇþù-*(□7û □Ïcÿ |Ñý•?ç±ÿ ¾kJŠ ÍþÈ-
óØÿ ß4□d□ùì□ïšÒ¢€3□²□üö?÷ÍÙ□þ{ûæ´¨

ßì•ÿ =•ýóGö@ÿ žÇþù-*(□7û □Ïcÿ |Ñý•?ç±ÿ ¾kJŠ ÍþÈ-
óØÿ ß4□d□ùì□ïšÒ¢€3□²□üö?÷ÍhFž\j™ÎÐ□iÔP□E□P□YÍ¤†b|ãÉÏÝ-□(□7û
□Ïcÿ |Ñý•?ç±ÿ ¾kJŠ ÍþÈóØÿ ß4□d□ùì□ïšÒ¢€3□²□üö?÷ÍÙ□þ{ûæ´¨

ßì•ÿ =•ýóGö@ÿ žÇþù-*(□7û □Ïcÿ |Ñý•?ç±ÿ ¾kJŠ ÍþÈ-
óØÿ ß4□d□ùì□ïšÒ¢€3□²□üö?÷ÍÙ□þ{ûæ´¨

ßì•ÿ =•ýóGö@ÿ žÇþù-*(□7û □ïcÿ |Ñý•?ç±ÿ ¾kJŠ ÍþÈ-
óØÿ ß4□d□ùì□ìšÒ¢€3□²□üö?÷ÍÙ□þ{ûæ´¨

ßì•ÿ =•ýóGö@ÿ žÇþù-*(□7û
□Ïcÿ |Õ›KAk¿□»v;b¬Ñ@□□Q@□□Q@□□Q@□□Q@□□Q@□□Q@□□Q@□□Q@□□Q@□□Q@□□Q@□□Q@□□Q
@□□Q@□□Q@□□Q@□□Q@□□Q@□□Q@□□Q@□□Q@□□Q@□□Q@□□Q@□□Q@□□Q@□□Q@□□Q@□□Q@□□Q@□□
Q@□□Q@□□Q@□□Q@□□Q@□□Q@□□Q@□□Q@□□Q@□□Q@□□Q@□□Q@□□Q@□□Q@□□Q@□□Q@□□Q@□□Q@□
□Q@□□Q@□□Q@□□Q@□□Q@□□Q@□‘ü¸□ñ□ªN+7û]¿çˆÿ ¾«Bãþ=¥ÿ pÿ *çh□Kû]¿çˆÿ ¾¨þ×où
â?ïªÍ¢€5#Õ

’¢y@n guiW;oÿ -
1□¾?•tT QE□ QE□ QE□ QE□ QE□ QE□ QE□ QE□ QE□ QE□ QE□ QE□ QE□ QE□ T73ýž□
]Ø#ŒÔÕSRÿ •&ú•ç@□¿µÛþx•ûê•ívÿ ž#þú¬Ú(□Kû]¿ç^ÿ ¾¨þ×oùâ?ïªÍ¢€4¿µÛþx•ûê•-¨
^EO(□Ä

î¬°’□øø□ýñüè□¢¢Š(¢Š(¢Š(¢Š(¢Š(¢Š(¢Š(¢Š(¢Š(¢Š(¢Š(¢Š(¢Š(¢Š(□-
åÙµ□BnÝžøª¿Úíÿ <GýõNÕþì_Sý+.€4¿µÛþx•ûê•ívÿ ž#þú¬Ú(□Kû]¿ç^ÿ ¾¨þ×oùâ?ïªÍ¢
€4¿µÛþx•ûê•ívÿ ž#þú¬Ú(□Kû]¿ç^ÿ ¾©F¬K□äŽ□Ú¬ÊTûëõ

–
Š(□Š(□Š(□Š(□Š(□Š(□Š(□Š(□Š(□Š(□Š(□Š(□Š(□Š(□Š(□Š(□Š(□ª]Þ
›iU□□Ü3œâ-ÖN©ÿ -
1ÿ »ýh□ZŠ(□Š(□Š(□Š(□Š(□Š(□Š(□Š(□Š(□Š(□Š(□Š(□Š(□Š(□Š(□Š(
□Š(

û•HÃ;Gå□·¾j?ívÿ ž#þúª·ßñû/Ô□*¯@□□2y°¤˜ÆáœSê□Oøô‹ýÑSP□E□P□E□P□E□P□E□P□E□P□E□
P□E□P□E□P□E□P□E□P□E□P□E□P□E□P□E□P□E□P□E□P□Y□ª•b<‘ÁÇÞ–:æßýc}M h□k·üñ÷Õ–
Úíÿ <GýõY´P□—
ö»□Ï□ÿ }Qý®ßóÄ□ßU›E nÙÝ□¤f)·□□sVk?Iÿ S'ûÕ¡@□□Q@□□Q@□□Q@□□Q@□□Q@□□Q@□□Q@
□□Q@□□Q@□□Q@□□Q@□□Q@□□Q@□□Q@□□Q@□□Q@□□Q@□□Q@□□Q@□□Q@□□Q@□□Q@□□Q@□□Q@□□Q
@□□Q@□□Q@□□Q@□□Q@□□Q@□□Q@□□Q@□□Q@□□Q@□□Q@□□Q@□□Q@□□Q@□□Q@□□Q@□□Q@□□Q@□□
Q@□□Q@□□Q@□□Q@□□Q@□□Q@□□Q@□□Q@□□Q@□□Q@□cx«Ä□Ã––¸Õ□•ÍóÆUc¶·\¼ŽÄ*□a’9ý□JÙ
¢€<CGño•&øÇ éÞ"eÓíu
y.□L^□□=’í

zîÊg¯åÒ½¾¼ƒÄŸòsž□ÿ °[ÿ +Šõú (¢Š (¢Š (¢Š (¢Š (¢Š (¢Š (¢Š (¢Š (¢Š (¢Š Žãþ
=¥ÿ pÿ *çk¢¸ÿ •i□Ü?Ê¹Ú (¢Š ʼßþ>bÿ |□ːè«•·ÿ •˜¿ß-
Î°* (¢Š (¢Š (¢Š (¢Š ã¼Q®x�So½Óü=¦ùf„É>«z¿¹O—v"^²6?à
õÏJ·ð÷V½×|□£êzŒÞuåÄ□¥"h]Çq□À

•…lk□ò□Ô□ëÖ_ý□×5ð›þIg‡ÿ ëØÿ èM@□x,□j:ÇØô;Ë□LHÃ5ü±
‰¤sœ„Œœ 8Îï^+#F×<Qáÿ □ÙøWÅ□¶Ú¬:œ2Ka¨C …ËF72:

□□ñíÉÏ-†Ì□K1□@É' ¯:Ñ ñ¿Ä□øJ¡□û□FŠK=:_ùú™¸-
Eõ@>P{þt□èÕ‡â=cQÓ□ÞßHÑæÔ¯î‹,c;!‹□ËJÿ Â9éÉ=□nQ@□'ÃÝkÄ□Ž-â‹□□^Ás6□x‘'Ùá□Æ
€¦H^äg»□k»¯>ð□üŽþ?ÿ °”□ú.½□€
(¢€
(¢€
(¢€
(¢€
(¢€
(¢€
©©□Ç“}Gó«uSRÿ •&ú□ç@□”QE □QE □$ññûãùÔu$ññûãùÐ□EE□P□E□P□E□P□^mã¯□x²□-
Z¹ÐlF™g¥£□5□ØÁyÙN1

g,¿í·□°5é5ÈüRÿ ’aâúóoæ(□ Ñn%¼Ð´û™ÛtÓ[G#¶1-*
8-æ¸iõ□□ø»ÅzÎ•áÍR×DÓôy□
n¤¶□□M)□8VùBŽŸçŽÓÃŸò+é?õå□þ€+ÍõÈï§ø‡uqðéäþÙR‘ë-(-
Ùç□□!<ù€cîdŽz□Ð□¾‰-x§CñÝ¯…|M{k«E¨[I=¥ü□□\□å•ÐqŒz{~-‰^Wà‹§ƒÆóGãH®¢ñ•ÌL-
òM´Û< ä-±^□©□æýkÕ(□Î¼Esâ-Wâhðî‡âa¢ÛG£-ó7Ø"¹ÞæfB>~G□ïÛ¥lè□□‹´íQn5Ÿ□ÿ kÙ„
Úÿ eEo’z□êsÇ¥Sñ,µÍGÆ+â-
Å
£Oö□±pl□à²‰□ÿ ˆàrGnÞõ•

»âï□x›IÓ<Syg¬iz´ÿ e,þ□<‰b˜ýÕt-
.□·¿<`€zM□Q@□□Q@□□Q@□□Q@□□Q@□□Q@□°¿Ý‹ê□¥eÖ¦¯÷búŸéYt QE□ QE□ QE□ R§ß
_-%*}õúÐ□KE□P□TõY¯-ô«©tËT°¾XÉ,□•"»ö□ž,®Q@-oy¥üT,ÂMQ<Q¥Iw□™

”šxò□□ì□“¿Û·Ôu®—Ã>-ƒÄ-□µñ;BbŽKw–X×•¥

□¯*qU¼{â7ÑôqşéÉö□wTÍ¶Ÿl¿x¹□.}□AÉ=8«¾□Ðí|!à›-"i£0Y[>Wá
å¤cž‹’ÇžÔ□Æh’|Cñ¾‘□ˆ-
<Mc¡Z]eíl¢°[‟³$□îýøíút-ÿ ø‟WÕßYÒ5øíÿ µtk‘□ÓÛ□#™XeX□Ðàr>•:W□¥Cã□-
oßá□‟þ□‡

Ö±ê□AÜ[æ6»¹Ûéæq×©®ßá□ÎŠÚMí¥Œw°êÑ\□Õ¢Ô□ãèÎÝYÏB

8#Œ□- wUÍxÛÄsx□HŽ=>1>µ¨H-tësüR·ñöT|Äû{×E4ÑÛÁ$óH±Å□—
wc€ª□I'Ò¸O□C/Šõùüs{□-±V¶Ñ!q•.ß?4ØìÒ-
üw□•h□ÇÃ□cXÖ4□Gûrõoo,õIìÌë□Æ□S p □Zí«€øMÿ ¯□ÿ ØÃ{ÿ ¡
ïè ¢Š(¢Š(¢Š(¢Š(¢Š(¢Š(¢Š(¢Š(¢Š(¢Š(¢Š(¬•Sþ>cÿ wúÖµdêŸñó-
û¿Ö€5¨¢Š (¢Š (¢Š (¢Š (¢Š (¢Š *□«□ig=ËG$‹

m!H•³¶□p u>,¦¢€<»QñWŒ—ÄÞ□-{x´}'SÔ□¸°uY.dM¤î•¹□z|«ÈîkÔkÏ¾"ÿ ÈÓà/û

□ì†½□€+ß[½Ý…Å´W2ÚÉ,l‹<Xß□#□†r2:×□ÿ □

µÿ E□Ä?ù
ÿ ‰®ê¹oˆ^!—Ãž□°¸´□µ+’¶-(¿y§“å\{Ž[þ□@□?□dÕîµß□I>»{ªhö—□ÊÒK½¤¼ˆ?zÃ p
z×£Ö'„|?□…¼)§hÑ□ÆÞ
$qür-]¿□$ÖÝ □QE □QE □QE □QE □QE □QE □QE `ß□Çì¿Qüª½X¾ÿ •Ù~£ùUz è-
?ãÒ/÷EMPÚ□Ç¤_îŠš€
(¢€
(¢€
(¢€
(¢€
(¢€8¯‰:¦³§ÙhVÚ-¤4ë-KX†Å®|„›b:¾NÖàò□åÖ ƒÂ¾?Žâ'›âW›□¸/ö□
¼g‘œñŸZÔñÇ…nüUc¦Çaª•2îÂþ;è®□¸›

ŠÀ|¤□Õ³Ï¥rÚÌŸ□¼□§K®O¯Ùx‟Oµ□î-¥±[Y□□ÄP§□□ž□#@-£\·ŽüU?…ô‹oìûU»Õu
¤²±□Î□¥~…¿Ù□þ]:×A§ßC©é–°…±&
¨RhÉ□•`□ý□r?□t□WS±ÒuM

□¸Ô´kô½ŽÙ›h□GÞ@{□Çë@□Z…‡Å□□M—Y_□éÚœ¶èf—
KþÎXã`□YRAó□Œã8Ívþ□×müMá»□jÕJÅw□‟a9(z2þ□□øW&¿□õk¸ü‹O‡ž%7Ä`¥Ô+

ÿ ×V8Ç¾)>□«¯Â□+xÂ™'(=□Î□ėš ô*(¢€
(¢€
(¢€
(¢€
(¢€
(¢€
(¢€
æßýc}Mt•Í¿úÆúš m□Q@□□Q@□ÚOú™?Þ-
ÏÒ□ÔÉþõhP□E□P□E□P□E□P□9â□_[†ö-+ÃúA¸¼š?1¯nr¶¶Ë’2ÄríÇÜ^{ä
Îøa-êÚï†.gÖnÖêî□B{□5bXÁTl

□b»Jóïƒßò+j_ö□»ÿ Ðè□_Æ~(»ÐÆŸ¦èÖqÞë°¤¦+8$l"…□y-
□íQ×□•□•ñ*9#°□□iÒ¾A{&Ò”CŽà8;ÿ □ÆñWöýÏÇ

]□[□nSAg□^«2Ä□b□•GVÀQƒÇ&¥×åø‰à}2O□Üx’Ï_±µ*×vRiélDd€J2œäg¿ëÒ€=N²õýZãGÓ„ö
š]Ö§u$,(m-€ÉcϾcÂ/□±éW¬î¢¾²·»„“□ñ¬¨O÷Xd□:š€<óÂšçŠgø•ªèÞ!¹´)-Ÿ□ÒZÚÆ6@ÌØÆ
ò71ÇRxÏ@+ÐëÏ´¿ù.ú÷ý•íÿ ô:ô□ (¢Š (¢Š (¢Š (¢Š (¢Š (¢Š (¢Š (¢Š (¢Š (¢Š (¢Š
 (¢Š (¢Š (¢Š (¢Š (¢Š (¢Š (¢Š (¢Š (¢Š (¢Š (¢Š (¢Š (¢Š (¢Š (¢Š (¢Š (¢Š (¢
Š (¢Š (¢Š (¢Š (¢Š (¢Š (¢Š (¢Š (¢Š (¢Š (¢Š (¢Š (¢Š (¢Š (¢Š (¢Š (¢Š +-
Å□÷ü#^-¹Õ¾Ás}ämýÅ²åÛ,□éœÖÅ□□óV©ãë□CâÆ□ã□ð®°¶ö□†ÝíÌG{□æò

1ÿ -
□å^ïá□□□ÅY¡.§ý›y§æF□É°]-Çqê9ý□oQ@□□Q@□□Q@□□Q@□□Q@□□Q@□□Q@□□Q@□□Q@□□Q@□□
Q@□Ü□Ç´¿îå\ítW-
ñí/û‡ùW;@□□Q@□[ÿ ÇÌ_ï•ç]□s¶ÿ ñó□ûãù×E@□□Q@□□Q@□□Q@□□Q@□O‹üfúk_hñøWÄÚƒ½¹
U¹°ÓüØ   eãæÝÛ<ñXŸ
¼Gsg¡hž□¼ð§‰-."‰£’îãN1Û)□›—' véÔ×¨Ñ@-Oñ□\ÕîüB¾□“Ãþ$—Ã^Š÷³é6M+Ðd□å□È
œá^9<Ž+oEñí¹šÃH´ð'‹¬-Ë%¼m.“åÃ
’ ,w|ª;ŸJïh

-_]¿Ó¼A£iÖÚ□Õíµû:Ïy□;-□□,Ü-¹îGNôx›Äßð•Co'ö-µªùÌWn—iç´xÇ,20

x-Ú(□Åü/âûÍ□Ä~'Ô.|□ã□‡U¼Ià□é$²¨\□ÙaƒôÍ{:•Ê□□dg□¨¥¢€
(¢€
(¢€
(¢€
(¢€
(¢€
(¢€
©©□Ç"}Gó«uSRÿ •&ú□ç@□"QE □QE □$ññûãùÔu$ññ-
ûãùÐ□EE□P□E□P□E□P□^[ñ□Åw:†ƒ¯xjÓÂ-*žâHÚÞ;˜´ÒÖîs÷ƒ□'¾ø¯R¢€8¯□ø®MNÞÓG—
Ã^"Ód¶³@×□…‡"
□
¤+däž c 5Îh×zïÃkýgO»ðÆ©¬i—
z„·¶×°Tbg"Le]2□#□šõŠ(□Ì Öü}ã=□Q□□^è°>□+Ü™u□□Ï;•ÀE@I□ÔžµÙ>»~ž5‹C□□ÓX=¡œê
€Ÿ)_$y}1ž=sÏNõ»E qšÇŽu□□X¹´ŸÁzõåª□äÝé±-À"□ %r
àä`úV0•\ø…â•□òóB°Ñt□-ãíª/ð·□3•ò□ƒî□×žµé"P□E□P□E□P□E□P□E□P□E□P□E□P□E□P□n
¯÷búŸéYu©«ýØ¾§úV] □QE □QE □QE □©÷×ëIJŸ}~´□ÒÑE□ V_‰5K•□Ã×°•–•>£s□yŠÒ□,ò±
□ãœœ

€Ö¥□□â¾□ñ<°mäúæ¹àÏ□j-"»]³\.Œ|¸□´P,ÿ *□ÌžMww□Iñ
ÀZÅ¤:v©£Ks□-¨š¥¿□û¶,□nOÈIÆ}□uôP□—è~7Öü;¢YèÚÏ€¼B÷-P¥¸“M¶[ˆd□□†

□□ tæ´|□¥ë7ž.Öüa¬iÇJ7ñEombÎ□A□□□˜à□Ç□G?•□E sZeüÞ-µ×´ÍgÃ×V□i4-
@NÌ□Ü$□]H €G¡=x5‰ÿ
Káçý
ßù;qÿ Ç+Ð(□)ø[ðçJÒ.¯õ{□□{]J×T°ŠÊIžU"ßî®□œ0*[

AÏ-zµ□P□E□P□E□P□E□P□E□P□E□P□E□P□E□P□E□P□E□P□E□P□E□P□Y:§ü|Çþïõ-jÉÕ?ãæ?÷□
- kQE□ QE□ QE□ QE□ QE□ QE□ TWSýšÒk□*Y|¤gòâ]Îø□ÂŽäö□-
□□âÞ+ñ}æ»-xfò×Àþ1Hô»ÿ ´Î%ÒH,»q…Ã□Ÿ®+Óü=â#â□6kÁ£júi‰Ê□5;o"GÀ□*2r9Æ}A-ª(□
□ÂzÝ÷ˆt$Ô5□□çF¸i□M¥É;À□ ò□çé\íõ…ï‰>,Ù››IãÑ¼=□Ÿ□’FU..ä□□'†□¼ät?Zïh □Š(□
Š(□Š(□Š(□Š(□Š(□Š(□Š(

□ïøý—ê?•W«□ßñû/Ô□*¯@□□§üzEþè©ª□Oøô‹ýÑSP□E□P□E□P□E□P□E□P□E□P□9â□□j---
Õ-¼5¨jö²□ó¤°Ã<Dc□aå³“Î{W!®ëþ"ñî“?‡´_
êú\WËäÜßë□ˆ□□•Þ*¹%É□□z×©Q̅@□î-u?ƒ<□‡HÒ.uw°†(!³€Ÿ1ÔmNÀž□<□Ò¦ÖõÛ
í+C·Ôm´□ÍFGdó--ˆóbR¤’□ûÄ□

□_jÜ¢€<×Pñ_Š<Ye&"áß êúSÜ©Š]GX^[-²-□•rK¶:c½w:

•máí□ÇH´Ï�around i
Ä¤õl

I÷''ñ–□(¢Š(¢Š(¢Š(¢Š(¢Š(¢Š(¢Š(®mÿ Ö7Ô×I\Ûÿ ¬o© □ÑE□ QE□□–¤ÿ ©"ýê
Ð¬ý'ýLŸïV… □QE □QE □QE s^%ñ•ü#wpÛÿ Â9â□SÍMþf—cç¢sŒ1Ü0}«,ømâkÍ

Ñô‹ï□x-$¼Õ%•gþÌ"(ÖW□.ÄŒ□ßƒ□zö*(□€ñÆ—¬ØøŸFñ-
ƒ`ÚŒÖ1Ék{dŒ□Í□sòz•98ïÇ½døƒYñ□ÄM%ü7¤øWWÒ ¼*—·ú¼"□

Y□¶.Irq□ó'ê´P□9®jw^□Òô¨4-
ïUFž+3□□æ□ñ□1°□□□ĐsÔUï□ëŸð□éŸmþËÔõ/œ'`¦ÛùÒóžväp1Öµh □□³ñ}å¿Ä-OÄ•à□□›;«□
-'□'|ÀÊÙ$•ØÇã^Á§Þ□hiÖ×Ÿg¸¶óâY<‹"Ù,y□ÚëÙ‡qVh □Š(□Š(□Š(□Š(□Š(□Š(□Š(
□Š(□Š(□Š(□Š(□Š(□Š(□Š(□Š(□Š(□Š(□Š(□Š(□Š(□Š(□Š(□Š(□Š(□Š
(□Š(□Š(□Š(□Š(□Š(□Š(□Š(□Š(□Š(□Š(□Š(□Š(□Š(□Š(□Š(□Š(□Š(□
Š(□Š(□Š(□Š(□Š(□Š(□Š(□Š(□Š(□Š(□Š(□Š(□Š(□Š(□Š(□"bÿ t~T´P□l
_î□Ê□‹ýÑùRÑ@
µ□?*Z(□Š(□Š(□Š(□Š(□Š(□Š(□Š(□Š(□Š(□Š(□Š(□Š(□Š(□
Š(□,□□#4Q@ ±□?*6/÷GåKE &Åþèü¨Ø¿Ý•-
□ ›□û£ò£bÿ t~T´P□E□P□E□P□E□P□E□P□E□P□E□P□E□P□E□P□E□P□E□P□E□P□E□P□E□P□E□
P□□□P□□□û£ò¥¢€□bÿ t~Tl_î•Ê-Š M‹ýÑùQ±□?*Z(□6/÷GåFÅþèü©h □Ø¿Ý•□□û£ò¥¢€
(¢€
(¢€
(¢€
(¢€
(¢€
(¢€
(¢€
(¢€
(¢€
(¢€
(¢€
(¢€
(¢€
(¢€
(¢€
(¢€
(¢€
B õ ÒÑ@□□Q@□□Q@□□Q@□□Q@□□Q@□□Q@□□Q@□□Q@□□Q@□□Q@□□Q@□□Q@□□Q@□□Q@□□Q@□□Q@
□□Q@□UIÉQùQ±□?*Z(éÒŠ(□Š(□Š(□Š(□Š(□Š(□Š(□Š(□Š(□Š(□Š(□Š(
□Š(□Š(□Š(□"bÿ t~T´P□l_î□Ê□‹ýÑùRÑ@ ±□?*6/÷GåKE
t RÑE □QE □QE □QE □QE □QE □QE □QE □QE □QE □QE □QE □QE □QE □QE □QE □QE
□QE □QE □QE □QE □QE □QE □QE □QE □QE □QE □QE □QE □QE □QE □QE □QE □QE □QE
□QE □QE □QE □QE □QE □QE □QE □QE □QE □QE □QE □QE □QE □QE □QE □QE □QE □Q
E □QLšh-áy§'"‰□gw`ª w$ô□ ú+ž´ñç„ïïÅ□¯^´ÙnY¶ª-ÂüÄô
z□ô®†€
(¢€
(¢€
(¢€
(¢€
(¢€
(¢€
(¢€
(¢€
(¢€
(¢€
(¢€
(¢€
(¢€
(¢€
(¢€
(¢€
(¢€
(¢€
(¬»Ÿ□è6wŸc°ÖôØ.³•"[¸Õóþé9 □J(□□□9□¡¢€
(¢€
(¢€
(¢€

(¢€
(¢€
(¢€
(¢€
©¨’,Û□□•çVê¦¥ÿ -MõÎ€3¬Y□ìy'©ïí[•…aÿ -
±}Oò-Ú (¢Š (¢Š (¢Š (¢Š (¢Š (¢Š (¬íC_Ñ´‰□=KW°²våVæå#'è□Š½

Ñ\Â"A*K□Œ«£□V- Ž´ ú(¦M4Vð¼ÓÊ'E□-wv
ª□RIè(□ôT6—v×ö±ÝYÜEqo
Ý□°¸tqê□àÔÔ QE□ QE□ QE□ QE□ QE□ QE□ QE□□•«□□,□9?Ò¥Ó
6œœüÆ¡Õþì_Sý*]/þ=?àF€.ÑE□ QE□ QE□ QE□ QE□ QE□ QEf]ø•CÓîÅ¥î³§[
\ž□Ít^çþ□Nh□NŠ@C A□-A□´ QX—>2ðµ•Ì-×^$Ñàž&)$RßD¬Œ:,
d□»¦kZVµ□'iZ□□úFv»ZÎ²…>„©8
ÔQE □QE □QE □QE □QE □QE □QE □QE □QE □QE □QE □QE □QE □QE □QE □QE □QE □QE
 □QE □QE □QP^^ÚiöÍq{u□´
÷¥š@Š>¤ñ@□ÑT´ígKÖ□ŸLÔ¬ïU>ó[N²□õÚM] □Š(□Š(□Š(□Š(□Š(□Š(□Š(□Š(

Ä'ûdŒœsÇáZu˜Ÿò□oÇùV• □QE □QE □QE □QE □QE □QE □QU£Ôlf¿šÆ+Ûy/!PÒÛ¬ªd@z□\ä
□- Y¢Š,òöÓN´’îúê□[hÀ/4ò□EÉÇ,x□‘@□Ñ\ÿ ü'~□ÿ ¡¯Cÿ ÁŒ?üUoG"K□É□«Æà2²œ†□¡□€
□E□P□E□P□E□P□E□P□E□P□E□P□E□P□\ã³y•ó-§½tuÍ¿úÆúš »¥17/’OÉýEkÖF•ÿ -
/þçõ□¯@□□Q@□□Q@□□Q@□□Q@□□Q@□□Q@□□LêÚhÕ□-u
A¨2ï□†eóJúìÎqïŠ¹@□□VLþ(ðýµá³¸×tÈ®□Ç‟%ÜjùôÚNh□ZŠ□□□

AïE □QE □QE □QE □QE □QE □QE □QE □QE □QE □QE □QE □QE □QE □QE □QE □QE □QE
□QE □QE □QE □QE □QE □QE □QE □QE □QE □QE □QE □QE □QE □QE □QE □QE □QE □Q
E □QE □QE □QE □QE □QE □QE □QE □QE □QE □QE □ÏxËÂ□ž5ÑâÒ¯®®`¶[„žAnÁL•Aù

Aãœý@®†£žxmmä¸¸•"†%/$ŽÁUTrI' □□ø£ðÓÁ°□Ã‹ýCOÓ□ÊîÌ!†d'Ë1.«ƒ'wg?…z?€®¯/|□
 Üß³=Ì-Q³»□-
ùxb{'0sï^ky5×Æß□Geh²Ãà□.móÜ□TÞH;/àxô□"É□½¦(£,□†□Xâ□B¢(ÀP8 □J }□Q@□□Q@□□
Q@□□Q@□□Q@□□Q@□□Q@□□Q@□□Q@□□Q@□□Q@□□Q@□□Q@□□Q@□□Q@□□Q@□□Q@□□Q@□wÄývï@ð□
ìús˜ïî□-mÜ□□y□.G¸□#Ü
•NøSàë=□4ë□□ÒòB˜šîx÷M+Ÿ¼Ûþð$äðF*□Æ€Ñø□/v"□-
¡mq&□ð‡□ÿ f□è1È'Æ²FÁ'Àee9□-†€<÷á\·□yñ□„ç•çM
ÿ Êµy

X[¸Ý□“íƒùûW¢W•ü<a{ã□+
ê±□Öòê1[#Ž…¡B-•ûèW¢P□E□P□E□P□E□P□E□P□E□P□E□P□E□P□U5/øòo¨þunªj_ñäßQüè□6Ã
þ?búŸå[µ…aÿ -
±}Oò-Ú (¢Š (¢Š (¢Š (¢Š (¢Š (¢Š ãÓá×ƒl…åæ¡¦ÚÞÍpí5ÅÞ§‰X’s÷›…□§□àVÂ(#·—Å
¥3·†¿´OöY$”<~óa=S8 ÷ÇÖ·<Cð¿Â–)Ô¥Ôµ]-¥¾•UZu¹•

Àà6ÞƒÒ²|□=þƒã-[Á□ßÍ¨i¶-'ÝÙK9□,
Ä□□7~¹-Â€=□¸Ï□xEu³¨_k□„÷Z|□ìÖº`□!G
÷äÇ2□ò3Àô5ÙÕ□oþ@:•ýzËÿ š æ¾□□Ë,ðÿ ý{ý
«³®3á7ü'Ï□ÿ ×±ÿ Ðš»: (¢Š (¢Š (¢Š (¢Š (¢Š (¢Š (¢Š ÍÕþì_Sý*]/þ=
?àF¢Õþì_Sý*]/þ=?àF€.ÑE□ QE□ QE□ QE□ QE□ QE□ É£ó '-î›Ô®ä8eÈê□-r6¿
<□m¦µ"è6·^`>eÅÒù"¹=XÈ~`O±□Ø×□âÍ~÷U¿□□x^Lê'®/¯G)§Bz±?óÐ•º½{ñ@□~□É(ð•ý™žI
ì¬u[›[□$9&ÝHÛÏ~KW¡Ön□¡ÙxoB´Ò4èöZÚÇ±sÕ□RÇÜ'I÷5¥@-7àõð□]ø¤x•|8oÏ^¯B□i□<Ò›
Æ1¿œg>ÝkÔ´m3EÓ-Khv:}-¼ørlbDI=□È0~µJçÀþ□¼šyî|5¤Ë4î^Y^Î2îÄä'ØÎIï\□ƒì!ð¿Å-
oÃz;:è²iÑß□må-
Úráp¹é•IÇÓÐP□§ÑE□ QE□ QE□ QE□ QE□ QE□ QE□ QE□ QE□ QE□ QE□ QE□ QE□ QE□ Q
E□ QE□ QE□ QE□ QE□ QE□ W—Á¥Úü@øŸ®¾µ□»Ò¼?åZÚYÉÌM3.é□—
¡#§=±é^¡^uà□□¿□<□$HvÏöø®,ž¥$L,=°~t□—ãß□ižŸHñ—
‡láÓf´½Š□ØíWËŽ{w;X2Ž3ÓœwöCë5ç□□□Oà^4¤9¸Ôµ□kh□uf.□□ûæ½□€
(¢€
(¢€
(¢€
(¢€
(¢€
(¢€
(¢€
(¢€3□þCMøÿ *Ó¬Äÿ •Ó~?Ê´è ¢Š(¢Š(¢Š(¢Š(¢Š(¢Š(□/_Ó/u}8YÙjÓi›ä-tð
2□ùÊ¡?pž>nq\□„tK□□üdÖôý:□□
¤@Çs□gbù,Ìy,ORkÔ«Ï´¿ù.ú÷ý•íÿ ô: ô□à¾3íÿ …W«oÆß2Û9é•´G]íV¿Óìµ[),µ
Xn-dÆøf@èØ ŒƒÇP□á@□•-›ð»R¸□ö□^□ºœð#,+gcø□ší#•"•c•□□ UU□
□@□q^$øiàËÝ
ì□□Oº×ágK«XV□^••Ù\tÆyâ¬ü/Ôìµ□†Ú%ö¤í%Ü□•w□¼á]•Xú' □÷Í uÔQE □QE □QE □QE □
QE □QE □QE □Í¿úÆúšé+›□ð•õ4□wJÿ •—ÿ súŠ×¬□+þ>_ýÏê+^€
(¢€
(¢€
(¢€
(¢€
(¢€
Çñ?^m¼/áû□Vä□ò†Ø¡_½4‡„E÷'□õ-Šòo□kwfât□¨ø[Äš†•£"Éd4í<Í□×, ™X'□È

ç□'Š ¡¤è□ZGÅ?□_jÌ$×5X¯®µ□□□Œk¶1þÊ.□~5í□â□×•þÓñ7Âú—
ü">+‹ì□]¯ÙeÓvÏ6ä□1¦ï˜

üñ^Ïcuöí>ÚïÈžßÏ‰dòn□d‘î□ÚËÙ†pG- qß□5
Æ‹ GðÖŸvöw:íÙ,K”8h-ÑwJÊ{□¸•VÓü3ð¥Q4kh<7s?ÜØòÅ-Ã-
©%óYÿ □´«□WÆÞ□‡V‹ ÌÓe°¹ ·™7•

Î‰±I□-Jþ5«âŸ‡-

>□Ô€Ð´û#□¬’%Ì0¬rDUI□¼rqŒó@□Å-´6v•ÚÛ Ž□Qc•□ð¨□□ò□-rÿ

/¯5/‡Z□Ýû³ÜÉj»ÝùgÆ@cêH çÞ°Š (¢Š +Œø—
àíGÆþ□□J°Õ□€óÄ"nRVU áN=ðqÓ□a]□□□âúwÂï^šE„6□□□b¶µ„mŽ(!T~_□zo„ôÝcIÐ"´×uo
íKåf-
s³nA<□|VÝ□ QE□ QE□ QE□ QE□ QE□ QE□ QE□ QE□ QE□ QE□ QE□ QE□ QE□ QE□ QE□
QE□ QE□ QE□□WRÓm5}2çN¾„Mks□ŽXÏu?Ëë\

□¼Ecf4½?â-§o£(Ø–æÖ7™□û«6w

□□□é□P□W‡<;§x[DƒIÒâ)m

NXå□□%˜÷$Ö-□P□E□P□E□P□E□P□E□P□E□P□E□P□E□P□U5/øòo¨þun »…®-Ìj@$Ž´□“aÿ -
±}Oò-ÚÍ¶Ó¥†á$gB□Ó5¥@□□Q@□□Q@□□Q@□□Q@□□Q@□! ,□CKE yÜ?

|A¤)¶ðï•õ+□;ø–®–c»òÇ÷Qœ,£Ò·ü'àËO

Ë•µÜê□•ó□»¿°lÉ)□□²ŽÂºZ(□
NÐ¯ìüUªêók·WVw«□Ã§¸>]±P□+É□û□×œÖ_‰|3âÍfúàéž6þËÓ¦•Ë6□ÛQOŽ0ß;□yý+±¢€8/□ø-
ÄÞ□k
Wñ·Û4kE+ö□ì¨ãÜ¤□□3qa,søb»Ú(□Š(□Š(□Š(□Š(□Š(□Š(□Š(

Ý_îÅõ?Ò¥Òÿ ãÓþ□i×Ö¯t□#(Ûž´û8□Þ□ŒA9Ï□□bŠ(□Š(□Š(□Š(□Š(□Š(□/¡šæÂæ
{ƒm<‘2G8]Æ& €ØïƒÎ=«Í4_†~0ðõ£Ûi□□D□É#K!:□.ò;□-gf,ÇÜ“^¥E `xgIñ

–.¿·¼Oý¸dÛäŸ°Gmäã;¾á;³'×¦=èÐ4
ý"M\Þë×z'ß]<Ð‰r>Ê‡¢&Ià{`qÒ·è □>_□øÊÝ|‹?‰wëkÐ–Æ□□òãþ°□œûÖÿ …<-
eáX®ž;‹›ÝBõÄ——×o°YØtÉì□8□«¢¢€
(¢€
(¢€
(¢€
(¢€
(¢€
(¢€
(¢€
(¢€
(¢€
(¢€
(¢€
(¢€
(¢€
(¢€
(¢€
(¢€
(¢€
(¢€
(¢€
(¢€
ãüMà?í•n□□HÖ.tMn(ü"un□ÖXúít<6?Îp1ØQ@□F□ðþxõû}wÄ¾!°×õ□@E§™
Á

□õe•xÝïþ□□½□P□E□P□E□P□E□P□E□P□E□P□E□P□E□P□E□P□b□Èi¿-
åZuMm□j□ãrí=»ôÅ\ □Š(□Š(□Š(□Š(□Š(□Š(

¯□ØjÚŽ™äh°×ö=Þðßiû*\|¼åv±Ç<sí\$_□¼e

½q-Çñn£q
Á,ßØp•È§ mÝ•õ[illegible]5ê[illegible]P[illegible]=¬h:¶-àÑ£§ˆî-
53[illegible]jú-¼[[illegible]™H,ÁU†ÝØ<[illegible]Æiuß[illegible]_êÚ=¥-ŸˆoôÛ»fVû\[illegible]&R[illegible]ƒ½O

s•Zè(□;›áß^5^þÉâO–ßê□kq%-µœvžjÿ u™2H=ÅwÖ––ö□pYÚD°ÛÀ,8£A€ª□ –
…ME □QE □QE □QE □QE □QE □QE □QE □Í¿úÆúšé+%´¹K□½9>ô šWü|¿ûŸÔV½Q²²’Úfve
®8«Ô QE□ QE□ QE□ QE□ QE□ QE□□•¨ødj–2Ñ|Cö³□Ó"ž?#ËÏ™æ¨□Ýž1□CŸjw‰ô+ýv
□ì5Û–%–îÒy–Ü□fEÎc8#ƒŸqÇC[´P□Šü–aâý□´Ûæ–0$Y¡ž□Û$2/Ýu>£'ó®VO†Úæ«
Øx□Çz†§¤□7ÙÇj–æP:+È¤³□Zôj(□+{xm–¢¶·□b,□□Ç□

□P0 -€T´Q@□□Q@□□Q@□□Q@□□Q@□□Q@□□Q@□□Q@□□Q@□□Q@□□Q@□□Q@□□Q@□□Q@□□Q@□□Q@□
□Q@□□Q@□□Q@□□Q@□□Q@□□Q@□□Q@□□Q@□□Q@□□Q@□□Q@□□Q@□□Q@□□Q@□□Q@□□Q@□□Q@□□Q@□□Q@
□□Q@□□Q@□H□$àRy‰ýáùÒMþ©ª¥ \ó□ûÃó£ÌOï□Î©Ñ@□<Äþðüèó□ûÃóªtP□Ï1?¼?:<Äþðüê•□
□sÌOï□Î•1?¼?:§E \ó□ûÃó£ÌOï□Î©Ñ@□<Äþðüèó□ûÃóªtP□Ï1?¼?:<Äþðüê•□□sÌOï□Î•1?
¼?:§E \ó□ûÃó£ÌOï□Î©Ñ@□<Äþðüèó□ûÃóªtP□Ï1?¼?:<Äþðüê•□□sÌOï□Î•1?¼?:§E \ó□û
Ãó£ÌOï□Î©Ñ@□<Äþðüèó□ûÃóªtP□Ï1?¼?:<Äþðüê•□□sÌOï□Î•1?¼?:§E \ó□ûÃó£ÌOï□Î©Ñ
@□<Äþðüèó□ûÃóªtP□Ï1?¼?:<Äþðüê•□□sÌOï□Î•1?¼?:§E \ó□ûÃó£ÌOï□Î©Ñ@□<Äþðüèó□
ûÃóªtP□Ï1?¼?:<Äþðüê•□□sÌOï□Î•1?¼?:§E \ó□ûÃó£ÌOï□Î©Ñ@□<Äþðüèó□ûÃóªtP□Ï1?
¼?:<Äþðüê•□□sÌOï□Î•1?¼?:§E \ó□ûÃó£ÌOï□Î©Ñ@□<Äþðüèó□ûÃóªtP□Ï1?¼?:<Äþðüê•
□□sÌOï□Î•1?¼?:§E \ó□ûÃó£ÌOï□Î©Ñ@□<Äþðüèó□ûÃóªtP□Ï1?¼?:<Äþðüê•□□sÌOï□Î•1
?¼?:§E \ó□ûÃó£ÌOï□Î©Ñ@□<Äþðüèó□ûÃóªtP□Ï1?¼?:<Äþðüê•□□sÌOï□Î•1?¼?:§E \ó□
ûÃó£ÌOï□Î©Ñ@□<Äþðüèó□ûÃóªtP□Ï1?¼?:<Äþðüê•□□sÌOï□Î•1?¼?:§E \ó□ûÃó£ÌOï□Î©
Ñ@□<Äþðüèó□ûÃóªtP□Ï1?¼?:<Äþðüê•□□sÌOï□Î•1?¼?:§E \ó□ûÃó£ÌOï□Î©Ñ@□<Äþðüèó
□ûÃóªtP□Ï1?¼?:<Äþðüê•□□sÌOï□Î•1?¼?:§E \ó□ûÃó£ÌOï□Î©Ñ@□<Äþðüèó□ûÃóªtP□Ï1
?¼?:<Äþðüê•□□sÌOï□Î•1?¼?:§E \ó□ûÃó£ÌOï□Î©Ñ@□<Äþðüèó□ûÃóªtP□Ï1?¼?:<Äþðüê
•□□sÌOï□Î•1?¼?:§E \ó□ûÃó£ÌOï□Î©Ñ@□<Äþðüèó□ûÃóªtP□Ï1?¼?:<Äþðüê•□□sÌOï□Î•
1?¼?:§E \ó□ûÃó£ÌOï□Î©Ñ@□<Äþðüèó□ûÃóªtP□Ï1?¼?:<Äþðüê•□□sÌOï□Î•1?¼?:§E \ó
□ûÃó£ÌOï□Î©Ñ@□<Äþðüèó□ûÃóªtP□Ï1?¼?:<Äþðüê•□□sÌOï□Î•1?¼?:§E \ó□ûÃó£ÌOï□Î
©Ñ@□<Äþðüèó□ûÃóªtP□Ï1?¼?:<Äþðüê•□□sÌOï□Î•1?¼?:§E \ó□ûÃó£ÌOï□Î©Ñ@□<Äþðüè
ó□ûÃóªtP□Ï1?¼?:<Äþðüê•□□sÌOï□Î•1?¼?:§E \ó□ûÃó£ÌOï□Î©Ñ@□<Äþðüèó□ûÃóªtP□Ï
1?¼?:<Äþðüê•□□sÌOï□Î•1?¼?:§E \ó□ûÃó£ÌOï□Î©Ñ@□<Äþðüèó□ûÃóªtP□Ï1?¼?:<Äþðü
ê•□□sÌOï□Î•1?¼?:§E \ó□ûÃó£ÌOï□Î©Ñ@□<Äþðüèó□ûÃóªtP□Ï1?¼?:<Äþðüê•□□sÌOï□Î
•1?¼?:§E \ó□ûÃó£ÌOï□Î©Ñ@□<Äþðüèó□ûÃóªtP□Ï1?¼?:<Äþðüê•□□sÌOï□Î•1?¼?:§E \
ó□ûÃó£ÌOï□Î©Ñ@□<Äþðüèó□ûÃóªtP□Ï1?¼?:<Äþðüê•□□sÌOï□Î•1?¼?:§E \ó□ûÃó£ÌOï□
Î©Ñ@□<Äþðüèó□ûÃóªtP□Ï1?¼?:<Äþðüê•□□sÌOï□Î•1?¼?:§E \ó□ûÃó£ÌOï□Î©Ñ@□<Äþðü
èó□ûÃóªtP□Ï1?¼?:<Äþðüê•□□sÌOï□Î•1?¼?:§E \ó□ûÃó£ÌOï□Î©Ñ@□<Äþðüèó□ûÃóªtP□
Ï1?¼?:<Äþðüê•□□sÌOï□Î•1?¼?:§E \ó□ûÃó£ÌOï□Î©Ñ@□<Äþðüèó□ûÃóªtP□Ï1?¼?:<Äþð
üê•□□sÌOï□Î•1?¼?:§E \ó□ûÃó£ÌOï□Î©Ñ@□<Äþðüèó□ûÃóªtP□Ï1?¼?:<Äþðüê•□□sÌOï□
Î•1?¼?:§E \ó□ûÃó£ÌOï□Î©Ñ@□<Äþðüèó□ûÃóªtP□Ï1?¼?:<Äþðüê•□□sÌOï□Î•1?¼?:§E
\ó□ûÃó£ÌOï□Î©Ñ@□<Äþðüèó□ûÃóªtP□Ï1?¼?:<Äþðüê•□□sÌOï□Î•1?¼?:§E \ó□ûÃó£ÌOï
□Î©Ñ@□<Äþðüèó□ûÃóªtP□Ï1?¼?:p
ŒŠ£W#ÿ V¿J u□Q@□□Q@□□Q@□□Q@□□Q@□□Q@□□Q@□□Q@□□Q@□□Q@□□Q@□□Q@□□Q@□□Q@□□Q@
□□Q@□□Q@□□Q@□□Q@□□Q@□□Q@□□Q@□□Q@□□Q@□□Q@□□Q@□□Q@□□Q@

›ýSUJ·7ú¦ª” QE□ QE□ QE□ QE□ QE□ QE□ QE□ QE□ QE□ QE□ QE□ QE□ QE□ QE□ QE□
QE□ QE□ QE□ QE□ QE□ QE□ QE□ QE□ QE□ QE□ QE□ QE□ QE□ QE□ QE□ QE□ QE□ QE
□ QE□ QE□ QE□ QE□ QE□ QE□ QE□ QE□ QE□ QE□ QE□ QE□ QE□ QE□ QE□ QE□ QE□ Q
E□ QE□ QE□ QE□ QE□ QE□ QE□ QE□ QE□ QEyo„o®æøãâûY.¦{t□JDÒ□ªAAÀè:š õ*(®?Ä
_□<'á‹æ±Ô5-×i÷á,3!O÷ˆàlæ€;
+Ã¾*Ñ|Wd×z5ò\Æ‡lŠ□VCþÒžG□®8¬□□|Mð¯…o□Ž¥¨□x □,□ÌŒ€ôÝŽ□Ó9

¾Šá´‹~□ñ-µo¤éóÜ›«,DbH
,@'¯ÐVþ'â-+\Õµ]2ÆW{□.A□ÈhÊ€Ù#,zò¦€6¨¬[ÿ □éZg^´ý□æW[ýAK@¢2A□=On†³õ_^Þ□Ðõ
›½+SÔEµÍ¬"i7ÆØ ã□p>fÃ□•ïèh□ª¢¸Ý□â—„5ùfŠ×VH¤‰□F[•1|‹É
·□□ž¹ÅC§ü]ð^¥«¦›o«bY□oL^ì{□#•Ç□□ÜQPÝÝÛØZKuw<p[Ä¥¤-F
ª=I5ÂÇñ§ÀÒ^‹aª¸□àLÖî□óÆGâ(□Ð(ª—:¥…ž-
ú□Åä)b'‰MÁq³ièAïœŒc®k□Ó¾0x/TÕcÓíõ7□Êâ8šH□UØœ

8üq@□ÕQÖu‹ –□H¹Õ5□¼«KuÝ#ã=ð □É$□Æ¯W•|b•õKß

xB'e]VùZà¡äF¤□Ëæ'þ□@-"§êvÚž"m©Û³
[ˆVdi□aØFA ôâ¬Cq□ÌbH%ŽXÏ□£`Ãó□å¿□@¸Ö¼□á†&-"þ÷eÌ(J‰□

aSŽß1üÇ¥E¤X[øCãŸö6‹□ƒLÔ4ß:kebR7□Ã`ôû¿øñ □X-h PÓJ‛©!Av □z

k+Ä~'Òü)e□æ-3Ão4ë□°¡`□,FqÐ`-kÎA?□>!□ o h□ç#î^\□'??—Ó}w-
□´d×¼□¬Ø•,ÿ gibÇ]éó®>¤cñ

˜□ŒŽ”W□ð³\m□áÞ•q+nž□6Òó“”;F}Êí?□vT QE□ QE□ QE□ QE□ QE□ QE□ QE□ QE□ QE□
QE□ QE□ QE□ QE□ QE□ QE□ QE□ QE□ QE□ QE□ QE□ QE□ QE□ QE□ QE□ QE□ QE□ QE□
 QE□ QE□ QE□ QE□ QE□ QE□ QE□ QE□ QE□ QE□ QE□ QE□ QE□ QE□ QE□ QE□ QE□ QE
□ QE□ QE□ QE□ QE□ QE□ QE□ QE□ QE□ QE□ QE□ QE□ QE□ QE□ QE□ UÈÿ Õ¯Ò©ÕÈÿ Õ
¯Ò€□E□P□E□P□E□P□E□P□E□P□E□P□E□P□E□P□E□P□E□P□E□P□E□P□E□P□E□P□E□P□E□P□E□P
□E□P□E□P□E□P□E□P□E□P□E□P□E□P□E□P□E□P□E□P□E□P□&ÿ TÕR-Íþ©ª¥ □QE □QE □QE □
QE □QE □QE □QE □QE □QE □QE □QE □QE □QE □QE □QE □QE □QE □QE □QE □QE □QE
□QE □QE □QE □QE □QE □QE □QE □QE □QE □QE □QE □QE □QE □QE □QE □QE □QE □QE
 □QE □QE □QE □QE □QE □QE □QE □QE □QE □QE □QE □QE □QE □QE □QE □QE □QE □Q
E □QE □QE □ä¾□ÿ ’õã/ú÷_æ•ëUâW°x×Âß□5ýsGðÃêv÷È¨Œ[
F□ä□}F1@-¿-^>•¡j□Ñ®é-
-¤™G©U$□*ùÏáýß^`Òî/ì¼□□¾÷S»>¡r7³-ë“ï’}sÍzï…|Eâí□RšÃÄ~□□vžð6éŒ„äð6àõÈ&¹□
3Iø…ðÂâóNÐ´¨uÝ

iŒ°å€t'ŽÄ□p□x#Ž;Ð□¾□hÞ%·ø›{«Üøcû
M»µ+,□àD□mÆÑž¤Œð8É¬•□ë>□Ò<aâñâç³þÑ{ç 5Ì^b•

û,œ□□þ|zW¤ø?UñÕþ¡uuâ-*ÇKÒÒ□bHÛ2□È9?1à□ÙÎ;{×‹x[Ã-!× Ô®<?¢éšŽ•/¤ògÕbO5ÿ à
_{¦ÜŒã$ã½ w□`²½ñ÷‹5m□ÑcÐË…¶o+hÜI?&G□□¸ì□t-□…ÿ òR¾!ÿ ×ÿ þÔ'—
Á~,×4o□Úx#Ä~ÓôÖ¸…¥´:x
˜□‰$□G;[ž

G#œÕm[Cñ—,þ jž!ð¶™-«§êß4öå°Qúž2

s’□ÈÁ Đ□Ÿ□□ÉtðWýr□æÕ“uáÝ7Ä□ʹuü□¤-}½½¤w□□û®Â4 0î9Î;ãž8¤²Ð>
ê¿□4□□øŽÂ4…K□.□R¶ˆ□àòz“ž¤óíŠêl|?ªÅñßT×-ÉÆ™.ž±¥ÎFÖm¨1×9È?• r_□|)£7•¼□o□”
VÉ©Ý}’çìê#Ü□â□€1œ9çééZÿ □|';iß

ÚK□.ÖÖK□cò^(Â°□í ž§9ÉÏR3Zß□¼?ªë-2ð-
æŸdóÛØj□[©□ŒD»á99>ˆß•hüWÒ5□sáõõŽ™j÷7Nñ•‰1’□,zÐ□□ñƒW¿¸ð□„l×|-©ˆå™TàÊÂ5!O
®Kçê□R¿ÿ „ž÷@—G_„¶PÂÑ□ãtA°2F□ƒ×pëœóÞ»¯□ø
çÅ?□ô•1□mµm>□^□ç•" VBFqõ□À¬X<Sñz
t±"ÁÖ"Ý¢…ûK8□Þç

?B Emà¿êß—Ã×15¾©

…á,g °+î□On§□ô□□Gáï□øf□ôÝ□Åþ□‡DÔíÊ□f–ÑDLê@

□□\'×'êk°Ô.¾ Ûø□O¹³±Óî<H%□yo'åù□7
ó

GÉœ\W
®i¿□~'}ƒIÕü;k£ØAp%šé›,0□;A$ž à

¸É□□î5ä~'□_Ú;Â'É÷□O.□¿ïÏó□½p

W‘|SØ□□¼□â-;`IþËs!è‰»<þ

ÿ • wž-ð}‡‹ì-â»–{{‹YDÖ·VÍ¶H\w□ð•ô¯.ñN•ú-°4□-
R¼Õ¼aâ□Ä□ß]8&Ú×ø±□ò,□ž¤□=8¯`ñ□-ý… ^ê□ƒšäÛÆYa…K3·@0□îFOaÍqŸ

|1}□»ñw^T□{X;Ê¸Á·‹øP

ÙÀã°
8Á

SÃ°§‰,¼=«§ƒ#ÒáÑ<<]   °□š[÷@ZG$- Æ@ã□□<qê~□ñ□^/ð□Ž°!ò-
î2$ˆœí`J°úd□{WšÛhþ.ðu¯Š<=§xrMJÓU-W±»ŠtU□Ì]¿>z`cÓ‘ès]n□`ß

¾□É□ÌÈ×□□RÈî□ÊemÌ□÷˜(

□ŏ|f>□¿SÊ□IÈ?öÎ:õªó¿,ZSéŸ□-e‘J½ôÒ]□#□É
?0€þ5è” QE□ QE□ QE□ QE□ QE□ QE□ QE□ QE□ QE□ QE□ QE□ QE□ QE□ QE□ QE□ QE□
 QE□ QE□ QE□ QE□ QE□ QE□ QE□ QE□ QE□ QE□ QE□ QE□ QE□ QE□ QE□ QE□ QE□ QE
□ QE□ QE□ QE□ QE□ QE□ QE□ QE□ QE□ QE□ QE□ QE□ QE□ QE□ QE□ QE□ QE□ QE□ Q
E□ QE□ QE□ QE□ QE□ QE□ QE□ QE□ UÈÿ Õ¯Ò©ÕÈÿ Õ¯Ò€□E□P□E□P□E□P□E□P□E□P□E□P
□E□P□E□P□E□P□E□P□E□P□E□P□E□P□E□P□E□P□E□P□E□P□E□P□E□P□E□P□E□P□E□P□E□P□E□
P□E□P□E□P□E□P□E□P□&ÿ TÕR-Íþ©ª¥ □QE □QE □QE □QE □QE □QE □QE □QE □QE □QE
□QE □QE □QE □QE □QE □QE □QE □QE □QE □QE □QE □QE □QE □QE □QE □QE □QE □QE
 □QE □QE □QE □QE □QE □QE □QE □QE □QE □QE □QE □QE □QE □QE □QE □QE □QE □Q
E □QE □QE □QE □QE □QE □QE □QE □QE □QE □QE □QE □QE □QE □QE □QE 5ÑdFGPÊÃ□
-â¼”|#×ôK‰Ç„¼iu§ØË!qk"’□ŸppO¾□àW®Q@□□„>□□
]o□kZÕÎµ¬ì)□Ód□□□8É$œ□;

□ÅwôQ@□□Q@□□Q@□□Q@□□Q@□s¾8ð¤>3ðµÆ‛,,)□¬•LW>\‹Ðãé‛ô&°*(□–•kue¢ÙZÞÜ
›¨`Hå˜

□
cÞ®ÑE □ËxûÂ×~1ðòhöú,ÙÃ%Â=Ë□Ü^5ÉÚ=ó´þ□ÔÑ@□ÙÚAae□•´b8
•b•□EU□□ò□5□P□E□P□E□P□E□P□E□P□E□P□E□P□E□P□E□P□E□P□E□P□E□P□E□P□E□P□E
□P□E□P□E□P□E□P□E□P□E□P□E□P□E□P□E□P□E□P□E□P□E□P□E□P□E□P□E□P□E□P□E□P□E□P□
E□P□E□P□E□P□E□P□E□P□E□P□E□P□E□P□E□P□E□P□E□P□E□P□E□P□E□P□E□P□E□P□E□P□E□P
□E□P□E□P□E□P□E□P□E□P□E□P□E□P□E□P□E□P□W#ÿ V¿J§W#ÿ V¿J u□Q@□□Q@□□Q@□□Q@□□
Q@□□Q@□□Q@□□Q@□□Q@□□Q@□□Q@□□Q@□□Q@□□Q@□□Q@□□Q@□□Q@□□Q@□□Q@□□Q@□□Q@□□Q@□
□Q@□□Q@□□Q@□□Q@□□Q@□□Q@□uÞ¥sŒÔ_gÿ kô©è □>Ïþ×éGÙÿ Úý*z(□□³ÿ µúQö□¢¿JžŠ ƒ
ìÿ í~"}Ÿý¯Ò§¢€ û?û_¥-
gÿ kô©è □>Ïþ×éGÙÿ Úý*z(□□³ÿ µúQö□¢¿JžŠ ƒìÿ í~"}Ÿý¯Ò§¢€ û?û_¥-
gÿ kô©è □>Ïþ×éGÙÿ Úý*z(□□³ÿ µúQö□¢¿JžŠ ƒìÿ í~"}Ÿý¯Ò§¢€ û?û_¥-
gÿ kô©è □>Ïþ×éGÙÿ Úý*z(□□³ÿ µúQö□¢¿JžŠ ƒìÿ í~"}Ÿý¯Ò§¢€ û?û_¥-
gÿ kô©è □>Ïþ×éGÙÿ Úý*z(□□³ÿ µúQö□¢¿JžŠ ƒìÿ í~"}Ÿý¯Ò§¢€ û?û_¥-
gÿ kô©è □>Ïþ×éGÙÿ Úý*z(□□³ÿ µúQö□¢¿J|ä-¼„□□„ƒøV-
Úî?ç³þt□³ö□¢¿J>Ïþ×éXßk¸ÿ žÏùÑö»•ùìÿ • lýŸý¯Ò•³ÿ µúV7Úî?ç³þt}®ãþ{?ç@□?gÿ
kô£ìÿ í~••ö»•ùìÿ •-
k¸ÿ žÏùÐ□ÏÙÿ Úý(û?û_¥c}®ãþ{?çGÚî?ç³þt□³ö□¢¿J>Ïþ×éXßk¸ÿ žÏùÑö»•ùìÿ • lýŸ
ý¯Ò•³ÿ µúV7Úî?ç³þt}®ãþ{?ç@□?gÿ kô£ìÿ í~••ö»•ùìÿ •-
k¸ÿ žÏùÐ□ÏÙÿ Úý(û?û_¥c}®ãþ{?çGÚî?ç³þt□³ö□¢¿J>Ïþ×éXßk¸ÿ žÏùÑö»•ùìÿ • lýŸ
ý¯Ò•³ÿ µúV7Úî?ç³þt}®ãþ{?ç@□?gÿ kô£ìÿ í~••ö»•ùìÿ •-
k¸ÿ žÏùÐ□ÏÙÿ Úý(û?û_¥c}®ãþ{?çGÚî?ç³þt□³ö□¢¿J>Ïþ×éXßk¸ÿ žÏùÑö»•ùìÿ • lýŸ
ý¯Ò•³ÿ µúV7Úî?ç³þt}®ãþ{?ç@□?gÿ kô£ìÿ í~••ö»•ùìÿ •jéò<-
Û□‹□Ç"@□}Ÿý¯Ò□³ÿ µúTôP□gÿ kô£ìÿ í~•=V¿vŽÑ™□©ÈäP□¾Ïþ×éGÙÿ Úý+□íw-
óÙÿ :>×qÿ =Ÿó □Ÿ³ÿ µúQö□¢¿JÆû]Çüö□Î•µÜ□Ïgüè□giÿ í~"}Ÿý¯Ò±¾×qÿ =Ÿó£íw-
óÙÿ : Ùû?û_¥gÿ kô¬oµÜ□Ïgüèû]Çüö□Î€6~Ïþ×éGÙÿ Úý+□íw-
óÙÿ :>×qÿ =Ÿó □Ÿ³ÿ µúQö□¢¿JÆû]Çüö□Î•µÜ□Ïgüè□giÿ í~"}Ÿý¯Ò±¾×qÿ =Ÿó£íw-
óÙÿ : Ùû?û_¥gÿ kô¬oµÜ□Ïgüèû]Çüö□Î€6~Ïþ×éGÙÿ Úý+□íw-
óÙÿ :>×qÿ =Ÿó □Ÿ³ÿ µúQö□¢¿JÆû]Çüö□Î•µÜ□Ïgüè□giÿ í~"}Ÿý¯Ò±¾×qÿ =Ÿó£íw-
óÙÿ : Ùû?û_¥-
gÿ kô¬oµÜ□Ïgüë^ÁÚKEgbÌIäýh□ßgÿ kô£ìÿ í~•=□□□Ùÿ Úý(û?û_¥OE Aö□¢¿J>Ïþ×éSÑ
@□}Ÿý¯Ò•³ÿ µúTôP□gÿ kô£ìÿ í~•=□□□Ùÿ Úý(û?û_¥WÔå'%•Ër¹Îpk;íw-
óÙÿ : Ùû?û_¥gÿ kô¬oµÜ□Ïgüèû]Çüö□Î€6~Ïþ×éGÙÿ Úý+□íw-
óÙÿ :>×qÿ =Ÿó □Ÿ³ÿ µúQö□¢¿JÆû]Çüö□Î•µÜ□Ïgüè□giÿ í~"}Ÿý¯Ò±¾×qÿ =Ÿó£íw-
óÙÿ : Ùû?û_¥gÿ kô¬oµÜ□Ïgüèû]Çüö□Î€6~Ïþ×éGÙÿ Úý+□íw-
óÙÿ :>×qÿ =Ÿó □Ÿ³ÿ µúQö□¢¿JÆû]Çüö□Î•µÜ□Ïgüè□giÿ í~"}Ÿý¯Ò±¾×qÿ =Ÿó£íw-
óÙÿ : Ùû?û_¥gÿ kô¬oµÜ□Ïgüèû]Çüö□Î€6~Ïþ×éGÙÿ Úý+□íw-
óÙÿ :>×qÿ =Ÿó □Ÿ³ÿ µúQö□¢¿JÆû]Çüö□Î•µÜ□Ïgüè□giÿ í~"}Ÿý¯Ò±¾×qÿ =Ÿó£íw-
óÙÿ : Ùû?û_¥gÿ kôªúd²J²yŽ[□c&¯Ð□-
gÿ kô£ìÿ í~•=□□□Ùÿ Úý(û?û_¥OE Aö□¢¿J>Ïþ×éSÑ@□}Ÿý¯Ò□³ÿ µúTôP□-
gÿ kô£ìÿ í~•=□□□Ùÿ Úý(û?û_¥OE Aö□¢¿J>Ïþ×éSÑ@□}Ÿý¯Ò□³ÿ µúTôP□-
gÿ kô£ìÿ í~•=fjSK□È□FPW±
Ÿgÿ kô£ìÿ í~••ö»•ùìÿ •-
k¸ÿ žÏùÐ□ÏÙÿ Úý(û?û_¥c}®ãþ{?çGÚî?ç³þt□³ö□¢¿J>Ïþ×éXßk¸ÿ žÏùÑö»•ùìÿ • lýŸ
ý¯Ò•³ÿ µúV7Úî?ç³þt}®ãþ{?ç@□?gÿ kô£ìÿ í~••ö»•ùìÿ •-
k¸ÿ žÏùÐ□ÏÙÿ Úý(û?û_¥c}®ãþ{?çGÚî?ç³þt□³ö□¢¿J>Ïþ×éXßk¸ÿ žÏùÑö»•ùìÿ • lýŸ
ý¯Ò•³ÿ µúV7Úî?ç³þt}®ãþ{?ç@□?gÿ kô£ìÿ í~••ö»•ùìÿ •-
k¸ÿ žÏùÐ□ÏÙÿ Úý(û?û_¥c}®ãþ{?çGÚî?ç³þt□³ö□¢¿J>Ïþ×éXßk¸ÿ žÏùÑö»•ùìÿ • lýŸ
ý¯Ò•³ÿ µúV7Úî?ç³þt}®ãþ{?ç@□?gÿ kô£ìÿ í~••ö»•ùìÿ •-
k¸ÿ žÏùÐ□ÏÙÿ Úý(û?û_¥c}®ãþ{?çGÚî?ç³þt□³ö□¢¿J>Ïþ×éXßk¸ÿ žÏùÑö»•ùìÿ • lýŸ
ý¯Ò•³ÿ µúV7Úî?ç³þuwMžYgpò3 ¹Á>ô□siÿ í~"}Ÿý¯Ò§¢€ û?û_¥-
gÿ kô¬ÛË™'îEYX x ûT-
k¸ÿ žÏùÐ□ÏÙÿ Úý(û?û_¥c}®ãþ{?çGÚî?ç³þt□³ö□¢¿J>Ïþ×éXßk¸ÿ žÏùÑö»•ùìÿ • lýŸ
ý¯Ò•³ÿ µúV7Úî?ç³þt}®ãþ{?ç@□?gÿ kô£ìÿ í~••ö»•ùìÿ •-
k¸ÿ žÏùÐ□ÏÙÿ Úý(û?û_¥c}®ãþ{?çGÚî?ç³þt□³ö□¢¿J>Ïþ×éXßk¸ÿ žÏùÑö»•ùìÿ • lýŸ
ý¯Ò•³ÿ µúV7Úî?ç³þt}®ãþ{?ç@□?gÿ kô£ìÿ í~••ö»•ùìÿ •-
k¸ÿ žÏùÐ□ÏÙÿ Úý(û?û_¥c}®ãþ{?çGÚî?ç³þt□³ö□¢¿J>Ïþ×éXßk¸ÿ žÏùÑö»•ùìÿ • lýŸ
ý¯Ò•³ÿ µúV7Úî?ç³þt}®ãþ{?ç@□?gÿ kô£ìÿ í~••ö»•ùìÿ •-
k¸ÿ žÏùÐ□ÏÙÿ Úý(û?û_¥c}®ãþ{?çGÚî?ç³þt□³ö□¢¿J>Ïþ×éXßk¸ÿ žÏùÑö»•ùìÿ • lýŸ

ý¯Ò•³ÿ µúU-6ye¸`ò3□¹Á>âµ(□□³ÿ µúQö□ö¿JžŠ ƒìÿ í~”}Ÿý¯Ò§¬□°¸

ÀLý}h□_ìÿ í~”}Ÿý¯Ò±¾×qÿ =Ÿó£íwóÙÿ : Ùû?û_¥-
gÿ kô¬oµÜ□Ïgüèû]Çüö□Î€6~Ïþ×éGÙÿ Úý+□íw-
óÙÿ :>×qÿ =Ÿó □Ÿ³ÿ µúQö□ŏ¿JÆû]Çüö□Î•µÜ□Ïgüè□gìÿ í~”}Ÿý¯Ò±¾×qÿ =Ÿó£íw-
óÙÿ : Ùû?û_¥gÿ kô¬oµÜ□Ïgüèû]Çüö□Î€6~Ïþ×éGÙÿ Úý+□íw-
óÙÿ :>×qÿ =Ÿó □Ÿ³ÿ µúQö□ŏ¿JÆû]Çüö□Î•µÜ□Ïgüè□gìÿ í~”}Ÿý¯Ò±¾×qÿ =Ÿó£íw-
óÙÿ : Ùû?û_¥gÿ kô¬oµÜ□Ïgüèû]Çüö□Î€6~Ïþ×éGÙÿ Úý+□íw-
óÙÿ :>×qÿ =Ÿó □Ÿ³ÿ µúQö□ŏ¿JÆû]Çüö□Î•µÜ□Ïgüè□gìÿ í~”}Ÿý¯Ò±¾×qÿ =Ÿó£íw-
óÙÿ : Ùû?û_¥gÿ kô¤²v{8Ù‰,s’~µb€ û?û_¥-
gÿ kô©è □>Ïþ×éGÙÿ Úý*z(□□³ÿ µúTÊ»T□JZ(¢Š(¢Š(¢Š(¢Š(¢Š(¢Š(¢Š(¢Š(
¢Š(¢Š(¢Š(¢Š(¢Š(¢Š(¢Š(¢Š(¢Š(¢Š(¢Š(¢Š(¢Š(¢Š(¢Š(¢Š(¢Š(¢Š(¢Š(
¢Š(¢Š(¢Š(¢Š(¢Š(¢Š(¢Š(¢Š(¢Š(¢Š(¢Š(¢Š(¢Šó/Œþ#ÔôÍ-
KÐ´YZ□K\¹û:H§□ À`□bK Ï¡4□èÂúÐÜ›au□œ□Ë! Ýùu©ëÆ®ÿ g½

?□°Ù_ß•i"Ý□ËJ□¼ d□¸áI÷Èõ5Ñ|□ñEç‰ü□•¨ÊÓ^XÎÖ²Jç- □b}pØÏS·&€=

Š(□Š(□Š(□Š(□Š(□Š(□Š(□Š(□Š(□Š(□Š(□î?ãÚ_÷□ò®v°+•øö—
ýÃü«• □Š(□Š(□Š(□Š(□Š(□Š(□Šó□□Dƒâ=ö-«x,k‰ì`½’ÒÎÁ&hâDL|Ä)□±Ïùã□tË6ð
7Ä
□□ÒæwÐõ˜%0[Í!□³Í□Üv"Î
ö÷ö □E¢°uÍ3XÕîâµ·ÔÆŸ¥□ÍÃÛçí26OÊ¬xEÆ>aÏÒ°¾□Æ°øVò□ÎÈõ+…\’N□zš îè¢©jú-®‰¤Ý
jw²l··Bî{Ÿ@=ÉÀ-æ€.Ñ^G¥Yj
ñ3ÃšÎ¬]5□Z+¹¤€"ˆ#□<¸€ö□Ÿrk×(¢Š(¢Š(¢Š(¢Š(-•/þ=?àF±«gKÿ •Oø□
´QE □SRÿ •&ú•çVê¦¥ÿ -MõÎ€1(¢Š (¢Š (¢Š (¢Š (¢Š (¢Š *—öÆ—
ÿ A+?ûþ¿ãW□□□FAê□sŸð¯ü#ÿ BîŸÿ ~E mÁ¨Y\ÉåÛÞ[ÊøÎØåV8ú□VkÍü□¢isøûYÖ4»□-
tý<□g[ˆSh’^²¿¾8QíM°Òcø‘«êÚ†µ5Äš5□ÛÙYXÇ3G□lûÒ6Ò
$ž(□Ò¨¯6M;þ□ï•tk}2âãû
Xv¶’ÒiK¬3c(ÈO#=??lzM □WœkÖÑø"âgü#úÝÝÄ:ZX¬ÖÖ‘Êb[·'æ$Ž[□ñíõÍk□□ÏÁž8ðõ¿…ç¸
…¯¦e»Ó¼ö‘

s!

I□ì□.ô□ê□QE □¹§□Ç’}Oó¬:ÜÓ¿ãÉ>§ùÐ□ª(¢€
(¢€
(¢€
(¢€
(¢€3u□»□Ôÿ JË-M_îÅõ?Ò²è ¢Š(¢Š(¢Š(¢Š(¢Š(¦M4Vñ□f‘#•z»° ~&ŸUµ□:ÏU²{;û
h®m¤Æè¥\©ÁÈãê(□?í□/þ,V□ŧý□Æ¦ŠöÒh^h®¡’$ûî²□□ê{W3¨x7ÁZ^›s□u ié□¼M,‡É□ Éª?

<;o□ƒg−îÆ□Æ¶ïs-®ß‘"~□<zmÇç@□wöÎ—ÿ A+?ûþ¿ãS[ÞÚ]−□×PÌW¨Ž@Øü«□¼□àôRÍáý9T

’b□□°¾□iVM.±âK+8í-5 ÌVQF»@,3´6=XäŸ¥ z□□W•xwÃúo□.õ›¿□Kqw¨[ßI□ØZáã[T□
□©-ü÷Ç®h□Õh¯=ðT□¤øÛ\Ð´ÛÙîô;Xce□Èd□Ó“ÌjßL’;cÖ½
€
(¢€
(¢€54Ÿ¹/ÔV•fé?r_¨-* (¢Š (¢Š (¢Š (¢Š (¢Š (¢Š (¢Š (¢Š +'Vÿ ^Ÿîÿ ZÖ¬•[ýz□›
ýh□>Š(□Š(□Š(□Š(□Š(□Š(□Šã~!jwöözV"¦Üµ¥Î¯|–†å~ôH~ñ_~•BûáN‘

Ÿ$ú4×ö°Ôh^□ávåÚ@27dã□õÀ□□è4W□£øÌMð°?□Þ(i"µf•G□äBT•l˚ýk;Lø{oâ
□µO□Íu©j7J%hLï□Vùä"*‘Œ□: ô*+3BÐ-<=§ý†Éî□¸rè³Jd)œ| žqÇOsWî'ŽÖÚ[‰›lQ!w>€

š ’Šóm□Ã#ÇöCÄ>)šæx/ {=5&hâ,□ü¹
FXŽIÏzëü=á};Ã□Ï

™ö„‚b□É’v‘PŒýÝÄ‘œóô□□µE□P□E□P□E□P□E□P□E□P□E□P□WôŸøø□+?¨ª□□Iÿ •‡ÿ súŠ ×¢Š(□□
ûþ?eú•åUêÅ÷ü~Ëõ-
Ê«Ð□E□P□E□P□E□P□E□P□E□P□E□ÃxÖ{ÍOÄš□„í¯f²·Ô□³^M□m□Æƒ;□öÏ9ÿ õŠ îh¯2ñ-
€,|1¢\ëÞ□’êÃSÓÐÜ□□

ë:¯,®□œ,□®Ö=B÷Wð¥¾¡£‹t»»¶Žh~ÒI□7€NqÉÀ'ŽäP□Å□æPi□é?□´&»Ô®u
Ë‹□‡žyŽ□#² á□z
ôÚ (ª°•µÅæ›qmkvÖsÊ…Rá□1Œžà-õÄß|+Ð—Ošâ;-E5TBë©½ã™C□□Çœuö□□è□W5ðÿ V»×<
¥j7Í°êXÙdcÕŠ».ãîBƒø×K@□□Q@□□Q@□□Q@□□Q@□ô¯øù□+?¨-zÈÒ¿ãåÿ Üþ¢µè
¢Š(®mÿ Ö7Ô×I\Ûÿ ¬o© □ÑE□ QE□ QE□ QE□ QE□ QE□ Q^q¯[GâO‰Ÿð•ëww□éib³[ZG)
‰nÜŸ˜’9lsÇ·×5®4□?□xãÃÖþ□žâ□¾™-
ïNóÚD0□Ì„1$c±ü»Ð□¨QXúõ¦µ|□[iWÑXBäý¦çfùUxÀŒ-2yäôì□rß

l“N×ü_g“J‛_FÍ!wo“’Ìz“@-ƒEËxßW¿²³±Ó4‰Z¦-r-
`”Œù+Œ¼˜ï•üè©¢¸døQá·A%çÛï/°µì·’y¥½x8ý+´¶€[ZÃn-G" ys62Osï@ÑE QE Q
E»§ÿ ÇŒ•ó5f«iÿ ñã-
ãüÍY Š(Š(Š(Š(Š(Š(Š(Š(Š(Š(Š(Š(Š(Š(Š(Š(Š
(Š(Š(Š(Š(Š(Š(Š(Š(Š(Š(Š(Š(Š(Š(Š(Š(Š(
Š(Š(Š(Š(Š(Š(Š(¼wã-·Žþ]ÊÁ`R,ìz(BOéü«Ø«’ø‰àˆ<yá£¦´ÂÞê)
ÖÓ‛•®>Ä?#Î(«wXÑ•Ø*¨É' ä-
³Â3xWY¹ ˆåÔ˜(ôÂ©ÿ Ù…U›Â¿5=4èÚö˜°{§“5Ð9’Hú•»•-
B{žMz‡„¼1gàÿ Zh¶D¼p^F299f?Sù

Ô□ ·E□P□E□P□E□P□E□P□E□P□E□P□E□P□E□P□E□P□E□P□E□P□w-
ñí/û‡ùW;]□Çü{KþáþUÎÐ□E□P□E□P□E□P□E□P□E□P□E□P□□|+â□□ê×÷>□Ôtß±_În$²Ô‘öÇ#}
â¬œóéÅdËeâ)~'øTkš••Ìñ-Ìÿ g³„¤vè□□Ü~fÜH□úWI}mñ□;û•¦ê□

¶RHÍ□»†E’%'…ù88é“Ö-xoÂÒéW·Z¶«¨6¥-]¨I.
lXã□ˆã^ËŸÏ- tµÃü-ÿ ‘rÿ þÂ·?ú□lkÿ ð—ý¦/øG?°þÏ³÷ŸÚ>níÙí³ŒcÖ¹¯
è->ðà□†O□=Œ·□qpwNdÃ°.□€:g□üh□ÑkË<_âí□ü}□•¯_}—KÒv\É□…ßí7□□€…Sò¨
óÔœs]Ö©m¯K®iRé·Öðé±³›ødL¼£□nÓƒŽýÇã[4□ä□·Äo
]|Aðî©

«°ÊÎ□•žO³Ê6□P□,¹9ö□ë□wp_ÙAyjþe½Äk,O,7+

ƒƒÈàÖ.¥ ÜÞxÛCÖ£’□oa□ÂJŒNö2(□o□íÎH®†€
(¢€
(¢€
(¢€
(¢€
ÙÒÿ_ãÓþ□k□¶t¿øôÿ •□ »E□P□U5/øòo¨þunªj_ñäßQüè□□Š(□Š(□Š(□Š(□Š(□Š(□¹
ï□k¯áï
]][,×²âÞÑ□V™ø\zã¯á]□sZ¿‡®õ□□è÷×□Cý“¦+L°äï{ƒÂ’1Œ(ä□ç=¨□ç…4$ðß†ltµ!ž□ó+ÿ
~CËŸÄ“\ïÂ,□Ã7öçýl□¥ÌrŽá·□ýEwuÃÞxSÄ□O^/uo
j□1. ÂK«□A□Ädþú•ä□ßÿ Õ€□ü@ýî¹à»TæfÖ□`;íA−?¨®ê¸ý□Â°£x…|Eâ}B
½B□ÌV°Z¡X−•ûÅsÉ'¦OëÆ5ÐÛ^>-Žá/-Æ,-Š½©OÞ□²~lã¦1ßðï@□¯^í□□õëŸ

ÛEo□¶”È×:‹¦é£‘†à□ò0p9cùU8¬.¾□êé}4«ªéZ•ÂA=åÂÿ ¦@ÍÂî□ãN=¿ÇgQð¦»câKÍwÂ°•¤□
ß□û]•ôlÐÈÊ0□□ä□□Z‰¼–â□□_Y¿‹5–8XZL·□ËM□öÊë÷w³ó□é@□Ý□Q@□nißñäŸSüë

·4ïøòO©þt□jŠ(□Š(□Š(□Š(□Š(

Ý_îÅõ?Ò²ëSWû±}Oô¬° (¢Š (¢Š (¢Š (¢Š (¢Š (¢Š á¼ví-êZGƒ¡c‹ù~Ñ}´ýÛhÎH>›˜ >•
Ü*ª EPª£ □€□sz‡®í¼U-ëúœ•ÉqvË□ªÄI□[¯AÈ□$òG##-t´□ÇüEÔ'M□□

Á±¨ës
(±ü□□Ö?Ð/ó®›N°ƒKÓm¬-Wl□Ñ,Q□`1X‰áë»□ˆ□x,úHZÖÚÔ[éñ!%”·2;□0□a,x®-
€□$‰□o$Œ□□□b{□^_□‡dø“qÿ 8•´KgWŽÒK/-
êt□]Ò¾qŽ8\tï]¾□g®#j‰¯^[]Á5Ã□D‰1åÀz+p2□P©®fÃÃ-3ð¬'OðÞ©¤ÝiJìÐEªG
xA9Ú□:ò{Ð□ü!=Ï…õ”ðV£okÌ□âÊòÖ?,\(8o1{?r{ÿ >ú¹□□ÂÚœ~ □□x□Q†óSò~Ï□VÑ”†Ý
ÉÛžI>§ÿ Õ×P□E□P□E□P□¦“÷%úŠÒ¬Ý'îKõ□¥@□□Q@□□Q@□□Q@□□Q@□□Q@□□Q@□
□Q@□□Q@□dêßëÓýßëZÕ“«□¯O÷□+ gÑE□ QE□ QE□ QE□ QE□ QE□□Ïø¿Ã□Äú\0ÅvÖw¶³-Í¥Ê
®Lr/BGqX•oñ□[:Þë>-³¶• ’ò
y-P rÛ[
œWO¯A-O`£B¼¶¶¼I□fæ2ñ°ààN9□AÈô®^ãÃž2ñ$□bñ-¯¦ÚéŒ□□□“□‡□□º]ùP{â€
9?±Ï□ìÕ0!·È¾v□]¦ä□ÿ ß<×Y□Âÿ

ê□q\ß5þ£q$aÅÝÅì…ù□ÈÁ ~U×]i□Wz□°;Â□ÆH□¿-œmL`□é•Ò¸Û-□â

•dšNŸ-hÓØD¾\[illegible]7PÉö^Đt[illegible])ÀõÍ Xøwqy

°öƒuw-äzMï“o<Í¹Ìdd)=ÈÇë]□‹UßÁšâG’í§Ü□Ç¯-
Ø¨|1á¨ü3¤Km□ËÜÞ\HÓÜÝÊ>i¥n¬GaíRøvÓZ‡C□þ%»¶¾¾,áä…0…
àchíí@□O„¾□ø□\ð~‘{ª5î£$¶‘□&»p±q□¨□€ éøUÿ □Ç>□ã}kÂ©w=Î™□¼wV¢wÞ
Ðnà¦}9à{}i,|/ã?
¤šw†õM&m#{4□êI'™n□çj”ê2{Öï…|/&„÷·ú…é¿Ö5□□urWhààQGe-
ç° □%□Q@□□Q@□□Q@□□Q@□□Q@□_Ò□âáÿ Üþ¢¨Uý'þ>ýÏê(□^Š(

□ïøý—ê?•W«□ßñû/Ô□*¯@□□Q@□□Q@□□Q@□□Q@□□Q@□rþ-
ðµÎ·q§jzUø°Ö4Öf·™"r2°Ã#□Cþ>µÔV'^`ñ$‹o'‡o,!'2ÞlW±3$ ã□¯#□=:çÚ€8ß□GãÁá=VM
cVÐ¬ì£´"Ì60Hï(ÚFÏÞp»°dz×gàûY,¼□¢ÛJ□';(CƒØì□□•ÿ □¯^üEs□ñ~§bÖ□È%þÎÓca□Ì

Fö~H□øz□ëµ?íìÙÿ ²~Ëöý¿¹û^ï+9þ-¼ã□é@□--ÿ %‡Ã¿ö□¹®â¼Òã@ø•sâK-
uäð¨°´…á•□®6□~¹□Î□□i£□Ärø^hîfÓ¡×š)□Kl□À□ÎÃ‡□úg ÷ □šâüWª\kwOàý
ÿ Òç\j□KÊÙ@zäÿ }‡□zóž:Ö¬Öž%o□□Hïí?á!0□7EH‹~yl□ôÎ>^½«”Ð´□^~□Óþ
Çbž□Ã1yf•®ZIœõwnìh□Ð4Ý:ÛIÓ-
´û4Ùom□Ç□û□ßÞ-V^ƒý½ö□ÿ „‡û7ížaÛýŸ¿ËÙ□Œïç9Ï·JÔ □Š(□Š(□Š(□Š(
úWü|¿ûŸÔV½di_ñòÿ î□QZô QE□ W6ÿ ë□êk¤®mÿ Ö7ÔÐ□h¢Š (¢Š (¢Š (¢Š (¢Š óÏ
□Ûë×>□¶ŠÞ□m)‛®u□MÓG#□Á!ä`àrÇòªqX]|1ÕÒúiWUÒµ+„,{Ë…ÿ L□›…Üÿ Æœ{□ŽÎ£áMvÇÄ—
šï…u+H%¾
ö»;èÙ¡‘”`0+È8þµ□x[Äþ"¾³□□jZp°´™n□-›□í•×îïgç□Ò€;°áü□ÿ #□•¿ì!þ•[þ ÿ „—
Êƒþ□¿ìŸ3qó¿´|Ìc¶Ý•úõ®7KÐ>#i□-©}□ž□iu)„Ó □à… cåÀ□\Ð□¥×•|D°þÑñ—
,ížîæÒ□e°•¦¶}Ž¤¢à□íœ□øšêüAmâ
›{!¡_[ZJ·(×&dÜ-.w*ðyéëõ□□‹¼2<O¥Å

wMg}k2ÜÚ]*äÅ"ô8î?Ïj æµ□…ú□¦‘w{¦5í–§□M,w©y!}Ê
□Ëc□sÒ°o□j·□ßƒ4-Fï›‰ □FÆ70È'ñÆ□□ç®´□-ë¶m¥ë□Î‘m§Ê6O.Ÿ□ùò§uù¾UÏ
·ÿ Z»{
□}3O·±´•Ë··•b•}□

±E□P□E□P□E□P□îŸÿ -1þ?ÌÕš-§ÿ ÇŒ□□ó5f€
(¢€
(¢€
(¢€
(¢€
(¢€
(¢€
(¢€
(¢€
(¢€
(¢€
(¢€
(¢€
(¢€
(¢€
(¢€
(¢€
(¢€
(¢€
(¢€
(¢€
(¢€
(¢€
(¢€
(¢€
(¢€
(¢€
(¢€
(¢€
(¢€
(¢€
(¢€
(¢€
(¢€
(¢€
(¢€
(¢€
(¢€
(¢€
(¢€
(¢€
(¢€
(¢€
(¢€
(¢€
(¢€
(¢€
(¢€
(¢€
(¢€
(¢€
(¢€
(¢€
(¢€□2—,E^¥H•c□g\ÿ Ï1ÿ }
Ü¢€0ÿ ³®□ç˜ÿ ¾…Ù×?óÌ□ßB·(

?ìëŸùæ?ï¡GöuÏüó÷Ð-Ê(□□û:çþy•ûèQý•sÿ <Çýô+rŠ ÃþÎ¹ÿ žcþú□□g\ÿ Ï1ÿ }
Ü¢€0ÿ ³®□ç˜ÿ ¾…Ù×?óÌ□ßB·(

?ìëŸùæ?ï¡GöuÏüó÷Ð-Ê(□□û:çþy•ûèQý•sÿ <Çýô+rŠ ÃþÎ¹ÿ žcþú□□g\ÿ Ï1ÿ }
Ü¢€0ÿ ³®□ç˜ÿ ¾…Ù×?óÌ□ßB·(

?ìëŸùæ?ï¡GöuÏüó÷Ð-Ê(□□û:çþy•ûèQý•sÿ <Çýô+rŠ ÃþÎ¹ÿ žcþú□□g\ÿ Ï1ÿ }
Ü¢€0ÿ ³®□ç˜ÿ ¾…Ù×?óÌ□ßB·(

?ìëŸùæ?ï¡GöuÏüó-
÷Ð-Ê(□□û:çþy□ûèV□Œ/□¾É□□q=jÍ□ QE□ U{ØžkfD□bGz±E aÿ g\ÿ Ï1ÿ }
?³®□ç˜ÿ ¼…nQ@□□Û×?óÌ□ßB□ìëŸùæ?ï¡[”P□öuÏüó-
÷Ð£û:çþy□ûèVå□□‡ý□sÿ <Çýô(þÎ¹ÿ žcþú□¹E aÿ g\ÿ Ï1ÿ }
?³®□ç˜ÿ ¼…nQ@□□Û×?óÌ□ßB□ìëŸùæ?ï¡[”P□öuÏüó-
÷Ð£û:çþy□ûèVå□□‡ý□sÿ <Çýô(þÎ¹ÿ žcþú□¹E aÿ g\ÿ Ï1ÿ }
?³®□ç˜ÿ ¼…nQ@□□Û×?óÌ□ßB□ìëŸùæ?ï¡[”P□öuÏüó-
÷Ð£û:çþy□ûèVå□□‡ý□sÿ <Çýô+RÊ'†ÕQÆ□gùÕŠ(¢Š(¢Š(¢Š(¢Š(¢Š(□-¡o-
À•Ë\ã9ç□Cû:çþy□ûèVå□□‡ý□sÿ <Çýô(þÎ¹ÿ žcþú□¹E aÿ g\ÿ Ï1ÿ }
?³®□ç˜ÿ ¼…nQ@□□Û×?óÌ□ßB□ìëŸùæ?ï¡[”P□öuÏüó-
÷Ð£û:çþy□ûèVå□□‡ý□sÿ <Çýô(þÎ¹ÿ žcþú□¹E aÿ g\ÿ Ï1ÿ }
?³®□ç˜ÿ ¼…nQ@□□Û×?óÌ□ßB□ìëŸùæ?ï¡[”P□öuÏüó-
÷Ð£û:çþy□ûèVå□□‡ý□sÿ <Çýô(þÎ¹ÿ žcþú□¹E aÿ g\ÿ Ï1ÿ }
?³®□ç˜ÿ ¼…nQ@□□Û×?óÌ□ßB□ìëŸùæ?ï¡[”P□öuÏüó-
÷Ð£û:çþy□ûèVå□□KO·’Ýd□.2F9Í]¢Š (¢Š (¢Š (¢Š (¢Š (¢Š (¢Š (¢Š (¢Š +?PµšyU£
\€¸ëŠÐ¢€0ÿ ³®□ç˜ÿ ¼…Ù×?óÌ□ßB·(

?ìëŸùæ?ï¡GöuÏüó÷Ð-Ê(□□û:çþy•ûèQý•sÿ <Çýô+rŠ ÃþÎ¹ÿ žcþú□□g\ÿ Ï1ÿ }
Ü¢€0ÿ ³®□ç˜ÿ ¾…Ù×?óÌ□ßB·(

?ìëŸùæ?ï¡GöuÏüó÷Ð-Ê(□□û:çþy•ûèQý•sÿ <Çýô+rŠ ÃþÎ¹ÿ žcþú□□g\ÿ Ï1ÿ }
Ü¢€0ÿ ³®□ç˜ÿ ¾…Ù×?óÌ□ßB·(

?ìëŸùæ?ï¡GöuÏüó÷Ð-Ê(□□û:çþy•ûèQý•sÿ <Çýô+rŠ ÃþÎ¹ÿ žcþú□□g\ÿ Ï1ÿ }
Ü¢€0ÿ ³®□ç˜ÿ ¾…Ù×?óÌ□ßB·(

?ìëŸùæ?ï¡GöuÏüó÷Ð-Ê(□□û:çþy•ûèQý•sÿ <Çýô+rŠ ÃþÎ¹ÿ žcþú□oOµš
™¤\□¸ëZ4P□E□P□MÝ”òÝ;¢□¤ñÈ¨□³®□ç˜ÿ ¾…nQ@□□Û×?óÌ□ßB□ìëŸùæ?ï¡[”P
□öuÏüó÷Ð£û:çþy□ûèVå□□‡ý□sÿ <Çýô(þÎ¹ÿ žcþú□¹E aÿ g\ÿ Ï1ÿ }
?³®□ç˜ÿ ¾…nQ@□□Û×?óÌ□ßB□ìëŸùæ?ï¡[”P□öuÏüó-
÷Ð£û:çþy□ûèVå□□‡ý□sÿ <Çýô(þÎ¹ÿ žcþú□¹E aÿ g\ÿ Ï1ÿ }
?³®□ç˜ÿ ¾…nQ@□□Û×?óÌ□ßB□ìëŸùæ?ï¡[”P□öuÏüó-
÷Ð£û:çþy□ûèVå□□‡ý□sÿ <Çýô(þÎ¹ÿ žcþú□¹E aÿ g\ÿ Ï1ÿ }
?³®□ç˜ÿ ¾…nQ@□□Û×?óÌ□ßB□ìëŸùæ?ï¡[”P□öuÏüó-
÷Ð£û:çþy•ûèVå□□•§ÚM□ÌÒ.□\u÷-□(□Š(□±_O¹.Ä Á?Þ□µE aÿ g\ÿ Ï1ÿ }
?³®□ç˜ÿ ¾…nQ@□□Û×?óÌ□ßB□ìëŸùæ?ï¡[”P□öuÏüó-
÷Ð£û:çþy□ûèVå□□‡ý□sÿ <Çýô(þÎ¹ÿ žcþú□¹E aÿ g\ÿ Ï1ÿ }
?³®□ç˜ÿ ¾…nQ@□□Û×?óÌ□ßB□ìëŸùæ?ï¡[”P□öuÏüó-
÷Ð£û:çþy□ûèVå□□‡ý□sÿ <Çýô(þÎ¹ÿ žcþú□¹E aÿ g\ÿ Ï1ÿ }
?³®□ç˜ÿ ¾…nQ@□□Û×?óÌ□ßB□ìëŸùæ?ï¡[”P□öuÏüó-
÷Ð£û:çþy□ûèVå□□‡ý□sÿ <Çýô(þÎ¹ÿ žcþú□¹E aÿ g\ÿ Ï1ÿ }
?³®□ç˜ÿ ¾…nQ@□ZFÑZ¢8Ã

ä~5=□P□E□P□E□P□E□P□E□P□E□P□E□P□E□P□E□P□E□P□E□P□E□P□E□P□E□P□E□P□E□P□E□P□
E□P□E□P□E□P□E□P□E□P□E□P□E□P□E□P□E□P□E□P□E□P□E□P□E□P□E□P□E□P□E□P□E□P□E□P
□E□P□E□P□E□P□E□P□E□P□E□P□E□P□E□P□E□P□E□P□E□P□E□P□E□P□E□P□E□P□E□P□E□P□E□
P□E□P□3□BÍĐ

š-ý£mÿ =□ýòjk•øö—ýÃü«• □øná□öFÄæg¡©ë-
Jÿ •¦ÿ pÿ 1[□ QE□ QE□ QE□ QE□ QE□ QE□ QE□ QE□ QE□ QE□ QE□ QE□ QE□ QE□ Q
E□ S%•!Bîp£ÚŸU5/øòo¨þt ¿Ú6ßóÐÿ ß&•í□oùè□î“XtP□çö□·üô?÷É£ûFÛþz-
ûäÖ□□□¹ý£mÿ =□ýòhþÑ¶ÿ ž‡þù5‡E n□hÛ□ÏCÿ |šš□ã•KFr□ÇJçk_Iÿ •wÿ □úP□ú(¢€
(¢€
(¢€
(¢€
(¢€
(¢€
(¢€
(¢€
(¢€
(¢€
(¢€
(¢€
(¢€"šâ+p<ÆÆzqš‹ûFÛþzûäÕ}_îÅõ?Ò²è□sûFÛþzûäÑý£mÿ =□ýòk

Š ÜþÑ¶ÿ ž‡þù4□hÛ□ÏCÿ |šÃ¢€7?´m¿ç¡ÿ ¾MXG□ u9□dW7]□¯üzEþà
¨¢Š (¢Š (¢Š (¢Š (¢Š (¢Š (¢Š (¢Š (¢Š (¢Š (¢Š (¢Š (¢Š (¢Š (¢Š (
¢Š (¢Š (¢Š *□n¢□,ÈØ'‘ÅMY:§ü|Çþïõ □j(¢€
(¢€
(¢€
(¢€
(¢€
(¢€
(¢€
(¢€
(¢€
(¢€
(¢€
(¢€
(¢€
(¢€
(¢€
(¢€
(¢€+É{□R□w!‡^□7ûFÛþzûäÖ]÷ü~ËõÊ«Ð□üWpÎû#b[□èjzÆÒÿ ãïþ□kf€
(¢€
(¢€
(¢€
(¢€
(¢€
(¢€
(¢€
(¢€
(¢€
(¢€
(¢€
(¢€
(¢€
(¢€
(¢€
ªu
`H.r?Ù5j¹·ÿ XßS@□_Ú6ßóÐÿ ß&•í□oùè□ï“XtP□çö□·üô?÷É£ûFÛþz-
ûäÖ□□□ÑC<s©1¶@8<b¤¬ý'ýLŸïV… □QE □QE □QE □QE □QE □QE □QE □QE □QE □QE □QE
 □QE □QE □QE □QE □QE □QE □QE □QE □QE □QE □QE □QE □QE □QE □QE □QE □QE □Q
E □QE □QE □QE □QE □QE □QE □QE □QE □QE □QE □QE □QE □QE □QE □QE □QE □QE □
QE □QE □QE □QE □QE □QE □OUÕ¬4=6mGS°ŽÖÒ□°Id<□ñ' □“W*-
«¤iúÝ‹Yjv‘ÝZ³+˜¤□RTä~¢€<ßCøÊž#ø…§è□□<©¦Þ,†;Ë,Uä
®w*ã□r„uü°Wª×ŽëñG□í-á□¢•c•4-TD□
¸À□°¯b □Š(□Š(□Š(□Š(□Š(□Š(□Š(□Š(□Š(□Š(□î?ãÚ_÷□ò®v°+•øö—ýÃü«•
ÚWü}7û‡ùŠØ¬}+þ>›ýÃüÅlP□E□P□E□P□E□P□E□P□E□P□\O^üm¬é¾.O

è^:ÕÏØEô‡ûA-¶!rŸÆ<ß¿Jí«Î<EŠt¯ŠðhžmjÚMl˜
èíÂ8™œòÝxÇnô· kþ.ÔuE·Ö|ý'fP"uý«Æè6(Ï>µÖW¢üD{•~ÄZæ©Ü,m-
wYbŸBÈ¼íþ"»Š æ¼OâÖÐnml,´{í_T°Vx--T
;ždã5"¥üC»-"´ÐüOá«-îû"ÍÞá.!™‡ðï\ ÞÞãÔg»¯9ñ»Çþðõ˜ó'°½]^ñ×¤G÷C-Û‰Æ>
"èÔQE QE QE QE QE QE QE SRÿ •&ú•çVê¦¥ÿ -Mõ-
Î€1(¢Š (¢Š (¢Š +_Iÿ •wÿ úVEké?ñîÿ ïÿ J ¿EPEP\ÿ 5›Ë›…ð×,uZÒÞF•îå
•-QÊœ/.2w´Ù$H£ì$uD@Y™Ž ©&€9ïøÆÏÆÃoqgwi),îÎåq$

ÇÛĐû□ÍÕ|s©¦±u¦xsÂWúÜÖm²âc*Û@-€v‡□¼FF@□Cá¢¶©-x³Å‘¡K□bõ□Ï#-dp©O0□F$þU»â•□
Ÿ

ÜÇ ðßˆ5C$^g›¦ÙyÑ¯$a›#□Œôé@□xGÇ□ø-
öûK»Ó.t□fÃi¸²¸!ˆVèÊÃ†-þãÖ°Êòÿ ‡7ðx«ÆZßŠæž□/Þ□²],□□[D§9”0□±>ƒ□¦Mz… aø‡Åz
g‡□1\´‟ß\qmalždóŸöTv÷8□Ö«ø#Åmã□

MEôö°xî¤¶h□Q!□

óÜÖïØí~Ýöß³Åö¿/Êóöû3»°ã$œW
ð{þEmKþÃu z Q@ Q@ Q@ Q@ Q@ Q@ Q@ °¿Ý‹ê¥eÖ¦¯÷búŸéYt QE QE W
Akÿ -‛¸+Ÿ®,×þ="ÿ pPÔQE QE Ù$H£i$uD@Y™Ž ©&•QÏW6ò[ÏK

ªRHÜeYHÁ□w□P□
/Å+□|C¥iú^•q}c□wöOí<ùpoÇ"2Gï1ŽHÀ÷5ÝO<VÖòO<‹□1)wv8
d’}1^□ãøb·ñ'Ãøa□#‰5p¨ˆ *€‡ □ÐWEãË
½OÀZí•Š³ÝMe"Æ‹ÕÎ>è÷=?□ æâø•–êÑ=÷†¼
¨jšH$-ä—Ine à²#□Xz□Jê|'â«□□èßÚ□I,L’47□ó®Ù •~ò0õä~uÌø+â„□,´¨¥Öì,&´´Ž
-nfXž7E
Ãk□O#µgü6ÕôýWâOŽ¦Ñ§□i“µ¬èê¥TÉ±ƒ‘•:°<÷Å z•p□□u□ÝJòÓÂ~□»×b²”Á=×ÚRÚ-
0}åFlîÇùìk¿¯□‹Ä3|(×u□□-N»§\Ë-ô1ií¾îÓw%eOîú6zdû

çÂ^7•Ä·—°eÞ™u¤ë6!ZâÆç□…=□XpËïî=k«¯;øu³Äš¦¡ã©ï,å°¾‰-
cµ´"x³…yØä€K"Éà{q^‰@□□Q@□□Q@□□Q@□□Q@□□Q@□□Q@□□Q@□□Q@□□Q@□□Q@□□Q@□dêŸñó-
û¿Öµ«'Tÿ •˜ÿ Ýþ´□-E□P□E□P□E□P□E□P□E□P□E□P□M'DŠ6'GTD□™˜à :'iÕ□ðEso%¼ñ¤°Ê
¥$□ÆU"Œ□GpE p²üR±—Ä:VŸ¥é×□Ö7÷□dþÓÏ—
□ür#$~ó□äŒ□s]õyß□áŠßÄŸ□á†4Ž$ÕÂ¢"€ª□□ □A^‰@□ïšé,.□Æ8¥»□±…%b¨ÏŽ□ □

×□ý£ñ7þ€--ÿ Àù?øšî«ÅZü-□ð¾¡-\`-¬%•Oñ¿E_Åˆ• `ø3Åzî¹â□oJÕôÛ+□i¿-
-kIšD20ÎÌ□2@ëé]µrŸ

´□ô□@/²Ú¥óµõû·VšO˜çÜ

/á]] □QE □QE □QE □QE □QE □QE □QE `ß□Çì¿Qüª½X¾ÿ •Ù~£ùUz »¥ÿ Çßü□ÖÍci□ñ÷ÿ
□5³@□□Q@□□Q@□□Q@□□Q@□□Q@□^#¹ñ=¹·ÿ „sOÓ®ÁÝçý²á£ÛÓnÜ□žõËjÞ%ø‡¢i□z¥ö‡áèím
bido·HN íòò{□ë^□^yãæ>$ñ&…àxŽènûCT□µ´G…>Îø€

¿Ãz•Þ¯á½;Q¾µ□·7Vé3À

vn□□ŸlV¥ 0□ ¬Ÿ□x,×Âþ°Õ®Áe…~H—ïJç…E÷'□€9¯□|KƒÁ·°YÃ¦¾¥pUe¹   7--
¢g□¬Ç□'Ç {Ww^+âß□Ýi_ µ-OX!õíZöÖæýÇð-
56D?ÙEà~5íT QE□ QE□ QE□ QE□ QE□ QE□ QE□ W6ÿ ë□êk¤®mÿ Ö7ÔÐ□h¢Š (¢Š ÖÒ□ÔÉ
þõhV~"þ¦O÷«B€
(¢€
(¢€
(¢€0üCâ½3Ã,□®ZIï®8¶°¶O2yÏû*;{œ□ëUü□â¶ñ†‡&¢ú{X<wR[4□(□,‡□•□ùîkwìv¿nûoÙâû
_—åyû□ý™ÎÝÝq'N+…ø=ÿ "¶¥ÿ a‹¿ý

€:□□x£Mðž•öýE¤!œE

0®ùg□ôD^ä×3□Ž<\ÒG4ß□õ□ì□•Þ-
ôM0□¹‡¯ášÆñ¶¶öŸ□´(?²ouf²Òäºµ´´@ÇÎ'Ênlð *Ÿ˜ôâ´n~%ë:□ŽçÅ>□½Òt§p□{□äw",œ□ê
ƒ*3þM z=QÕµ•;AÓ¤Ô5K¸-mcûÒHqÏ`□RO`95u□]□Ñƒ+

’

A□□Í□–ç•ö«x¦òd□Åæ mŽ2□

ô#'Ÿz å<3ãïøI|S}£□□îÆ;{T°Š[£µåF8□ËÆT-£'8ì+²¯>Òÿ ä»ëßö□·ÿ ÐëÐh ¢Š(¢Š(¢
Š(¢Š(¢Š(¢Š(¢Š(¢Š(¢Š(¢Š(¢Š(¢Š(¢Š(¢Š(¢Š(¢Š(¢Š(¢Š(¢Š(
¢Š(¢Š(¢Š(¢Š(¢Š(¢Š(¢Š(¢Š(¢Š(¢Š(¢Š(¢Š(¢Š(¢Š(¢Š(¢Š(¢Š(¢Š(
¢Š(¢Š(¢Š(¢Š(¢Š(¢Š(¢Š(ªz¦«a¢éòßêWQZÚE□òÊp£'□õ5r©êšU†·§Éa©ÚÅui.7Å
È89¨ □

×|iá»••þ□ÖáÖ-ŸL·ÓÚ)®A;□ˆŸƒÿ }/ç^Ý¤kZf¿`/´›Øo-‹□ó"l•Ã¨ú×?ÿ
³Àßô,Ø□ß'ük ÑôM3@°□:M”6vÁ‹ùq.□ãÔýh□ý□Q@□□Q@□□Q@□□Q@□□Q@□□Q@□□Q@□□Q@□□Q@
□□Q@□Ü□Ç´¿îå\ítW-
ñí/û‡ùW;@□´¯øúo÷□ó□±XúWü}7û‡ùŠØ □Š(□Š(□Š(□Š(□Š(□¹ë•□øVÏU¹Ó/5û
[Ëf
,W2ˆ¶’□□¶□àŽ™®†³u□□hº´•õ-O½qÀk›d•ÿ ãÀÐ□žx‡YÓ¼wão
i~□•/ÿ ²õ□Ôoo ù¢,4□.þ„±ã□Ò½□Pñ-“¥jÚv—{x°Þê,Ëk□V>a\g□0:Ž¸«v:}—™n-
ì,íí!□"8"X×ò□□O§Y]][Ý\YÛÍqlI,i"Vx‰ë´‘•Ïµ r~<ñí¿…E¶™o-§öÕøÅ¸»˜E

+ÐË+’ QƒÇV#□©øKPð_†ífy<e¢ßj×¯æß_I¨C¾y?ï®□tU□□]v¥áÍ□Y™&Õ4m:úT]Š÷V©+*õÀ,

□5Hx□Â äxWCÏýƒ¢ÿ âh□ ¢Š(¢Š(¢Š(¢Š(¢Š(¢Š(¢Š(ªš—
üy7Ô□·U5/øòo¨þt□‰E□P□E□P□E□P□ZúOü{¿ûÿ Ò²+_Iÿ •wÿ □iP□ú(¢€
(¢€
ò-cÆZ7ŽõiôFñ□Ÿ¥øbÖM—
²Íz•Í¨0ë□`•D^-ü]□□¯]¬□ð7„]‹7…tBÄä"§Å'□iš ŸD×<7z©§hZ®—r-
â□mì®c□.1€>U' d□Ê² ø—á7Ôotë-Z□:òÎg†Xu□
9SŒ©n□8ÈÁèElé¾□Ð´k†¸Òô]:Æf]•%-ªDÅr

P

2□-Õ&£ èú»+jzM•ë/
nm’B>›• □;±¿°ñ_ÆÛMWÃŒ'´Ó´ù!Ôo¡»•›îG»£□×ðö¯@o□é)âDðó^(Õ^□´-
¾ÖÉ•$g8ÇcÆsWm,¯íl-ÖÞÎÚ□h□îÇ

a[illegible]} âšté#¨[illegible]DÙÛ›å[illegible]Ê[illegible]&%ó[illegible]g;wc8Ïj «©x‹CÑ¥Hµ]gO±'×r%ÕÒDXtÈ

FEyçÂO□è6ú□åœÚÞ›□Ôú½É†□»@ò□q·j""žØë^‡©xwCÖfIuM□N¾'□j=ÕªJTuÀ,

□V‡Á~□¶ž9àðÎ□□Ñ°xäŽÂ%d`r□!r□=è□rŠ(□Š(□Š(□Š(□Š(□Š(□Š(

Ý_îÅõ?Ò²ëSWû±}Oô¬° (¢Š (¢Š + µÿ •H¿Ü□Ï×Akÿ -‘□¸(□j(¢€
(¢€
d²Ç□O,®±ÆŠYÝÎ□□É$ö□úd±G<O□±¬‘°•tq□ÀðA□Å yO□<YáËÏ□x*[_□iSÇoªù‟¼W‘°‰vŸ™^<
□s^□□ˆ´[-*ëTµÕ,î¬mUšyí¦YU□®æÉ\ò□8ëT¿á□ð□ý
šþ
¡ÿ âkONÑô½"ÙíôÍ6ÎÊ□mÏ□´
□±Æ2B€ À• qz—‰¾□É□j÷·>□»›□÷ùQM;÷p_?…OðæÂêiu¿□ÞÙ5”šÜèÖö®»Z+h—
d[‡bFN=Åt□x[ÃÖ×,òßAÒâ°□"hìãWÏ®à3ZÔ□•aâ-?_‹T‹C¼K‹«□-ÞQµ€Ž`□□ dg¸È¯=øYâ¯

i-□{=cQµÓ|D'"ê£Q□E4"o<-|nà□¥z•¦□e`Óµ□□½»\He˜Ã□¡'ÏVl

O¹ªÚ‡‡tM^Q.¥£i÷²□€÷6©!‹□@-w¡^i°ÇÆ¹µ□
²É§.œË«\[ŒA4Å¾Nz3÷ÈíŸzõj,ÒÊÖÂÝmìí¡¶•~ìpÆ□GÐ

*z (¢Š (¢Š (¢Š (¢Š (¢Š (¢Š (¢Š (¢Š (¢Š (¢Š (¢Š +'Tÿ •˜ÿ Ýþµ-Y:§ü|Çþïõ
j(¢€
(¢€
(¢€
(¢€
(¢€
(¢€
d²Ç O,®±ÆŠYÝÎ É$ö úd±G<O ±¬'°•tq ÀðA Å yO <YáËÏ x*[_ iSÇoªù"¼W'°‰vŸ™^<
 s^—§ë:^-k%Ö›©YÞÛÆJ¼¶Ó¬ª¤

•J’□Á□³□á□ð□ý
šþ
¡ÿ âkONÑôÍ-ÝíôÍ:ÒÆ□mí□´
□³c□!@□à□Ê€!Ð|A¥x›L]KG»□VŒì,@Œ¼Ž□Ã k•ñaÿ „¯â&‰á4ù¬tüjÚ˜ìvœC□ú±É-„-ÕÞXéö
Ze¨µÓìíí-Ô’"·ˆF€žIÀ Q

Ÿeo{s{□¤□Ý\íóçHÀyvŒ.æêp8□é@□h¢Š (¢Š (¢Š (¢Š (¢Š (¢Š (¢Š (¢Š Á¾ÿ •Ù~£ùUz
±}ÿ ²ýGòªô□wKÿ •¿ø -šÆÒÿ ãïþ□kf€
(¢€
(¢€
(¢€
(¢€
(¢€□4ÑÛÁ$ó:¤Q©wv8
É&¸□†qI-M¬øâí□eÖ§Ùf¬9ŽÒ3µ□¶H$úà□ï®-á»¶-Úæ$š P¤'È¡•ÔŒ□Aê□íE½¼□-
Ñ[[C□0D¡#Ž5
¨£€ □□@□µÍsMðæ".©«]
k(Š‡"«6

□□y# ®sÆ>□½ñ£hwúWˆ†š¶2□¨‰³[”‘È□k0□Qœd-
½Ú°ËÛ□=JÑí/í`°¶|o†xÃ£`äeO□‘š□UQ□"…U□

(□Ä~&øwÆv>□¸ŸTñßö• ž ÖßÙ□Ã’dP

å9ààã¾+Õü9§k:fœðëšïöÍÑ”²Ü}‘-
¶¦□□j□□□N}ý«FòÆÓQ¶6÷¶°\ÀHc□ñ‡RAÈ8<p@5= □QE □QE □QE □QE □QE □QE □QE □Í¿ú
Æúšé+›□ð•õ4 Ú(¢€
(¢€5´Ÿõ2□½Z□Ÿ¤ÿ ©“ýêÐ □Š(□Š(□Š(

ÍKÄZ-•*Eªë:}ŒŽ»'.®'"Ã¦@b2+Ï>□x—
A·Ð¯,æÖôØî§ÕîL0=Ú□•3□»TœœöÇZô=KÃ°-³2KªhÚuô^»Qî-RR£®□`p*´>
ð-´ñÏ□†th¦□ƒÇ$v□+#□□A
•Aï@□□Œï#ð—Å□□Å—Á"I¸³"K°¸
H€-Þ…±Ø"ú□<â?Œô-oÂW>□Ð¯-µ•[V
omme —□°%˜®B€□yÿ □zlöðÝ@ð\C□Ð¸ÃÇ"†V-„-µKNÐ4m□ÙôÍ&ÂÉœašÚÙ#-
õÚ□ Q›WÒü□¡é□ÚÍúÂ□•c□…Y¼É6àt□□ÁäñZš†©§é□¿iÔ¯-¬-÷□ónfXÓ' Ë□3N¼Ó¬µ□‰o¬íî-
)□±‰âW□ã£

Ž□õ¦ê□^Ÿ«Úý›R±¶½·Ü□Ê¹…dLŽ‡

Í yŽ•âÏ

'Æ•jýüA¥-œšT□¥Á¼ŒFÌ□•□œ□=+Õmî
»¶ŽæÚhæ,U□□±°eu<,□àƒëX□ð,x?þ…M□ÿ □Ðÿ ñ5¹oo□¥´vÖÐÇ

□(Hâ•Bª(à □ {P□"QE □QE □QE □QE □QE □QE □QE □QE □QE □QE □QE □QE □QE □QE
□QE □QE □QE □QE □QE □QE □QE □QE □QE □QE □QE □QE □QE □QE □QE □QE □QE □QE
□QE □QE □QE □QE □QE □QE □QE □QE □QE □QE □QE □QE □QE □QE □QE □QE □QE □Q
E □QE □QE □QE □QE □QE □QE □QE #¨te=□`Õ_ìÛoî·ýõVè □!³†□ß□
ã□šžŠ(¢Š(¢Š(¢Š(¢Š(¢Š(¢Š(¢Š(¢Š(¢Š(¢Š(¢Š(¢Š(¢Š(¢Š(¢Š(¦K□M
□G□iô§Ñ@□?³m¿°ß÷ÕÙ¶ßÝoûê–Ñ@□?³m¿°ß÷ÕÙ¶ßÝoûê–Ñ@□?³m¿°ß÷Õ–
Ù¶ßÝoûê–Ñ@□?³m¿°ß÷ÕM□□Û©XÁ œòjZ(¢Š(¢Š(¢Š(¢Š(¢Š(¢Š(¢Š(¢Š(¢Š(¢Š
(¢Š(¢Š(¢Š(□)í£¸
æ□vôÁ¨□³m¿°ß÷Õ[¢€*□fÛ□u¿ïª?³m¿°ß÷Õ[¢€*□fÛ□u¿ïª?³m¿°ß÷Õ[¢€*□fÛ□u¿ïª²^#EE
è£□•E □QE □QE □QE □QE □QE □QE □QE □QE □QE □QE □QE □QE □QE □QE □QE □QE □
QE □QE □□Ö±Náœ□@ÀÁ©è □Š(□Š(□Š(□Š(□Š(□Š(□Š(□Š(□Š(□Š(□Š(□Š(□Š
(□Š(□Š(□Š(□Š(
ÒXÁ,…Ü□Ç¯4ßìÛoî·ýõVè
ðÙÃ□ï@AÆ95bŠ(¢Š(¢Š(¢Š(¢Š(¢Š(¢Š(¢Š(¢Š(¢Š(¢Š(¢Š(¢Š(¢Š(¢Š(¢
Š(ª‡N·$'–"ïVè
ŸÙ¶ßÝoûê•ìÛoî·ýõVè
ŸÙ¶ßÝoûê•ìÛoî·ýõVè □ ·ŽÝHŒ□ ÉÉ©h¢€
(¢€
(¢€
(¢€
(¢€
(¢€
(¢€
(¢€
(¢€
(¢€
(¢€
(¢€
(¢€
(¢€
(¢€
(¢€
(¢€
(¢€
(¢€
(¢€
(¢€
(¢€
(¢€
(¢€
(¢€
(¢€
(¢€
(¢€
(¢€
(¢€
(¢€
(¢€
(¢€
(¢€
(¢€
(¢€
(¢€
(¢€
(¢€
(¢€
(¢€
(¢€
(¢€

(¢€
(¢€
(¢€
(¢€
(¢€
(¢€
(¢€
(¢€
(¢€
(¢€
(¢€
(¢€
(¢€
(¢€
(¢€
(¢€
(¢€
(¢€
(¢€
(¢€
(¢€
(¢€
(¢€□3□,F□UI-
•cÿ i\ÿ y□ìš×¸ÿ •i□Ü?Ê¹Ú é‡J(□□□ QE□ QE□ QE□ QE□ QE□ QE□ QE□ QE□ QE□ QE□ QE
□ QE□ QE□ QE□ QE□ QE□ U{É^□ft8`EXªš—
üy7Ô□: ÏþÒ¹þòÿ ß55-ôòÜ¢9□O^=«:¬Ø□Çì_Sü¨□vŠ(□Š(□Š(□Š(□Š(□Š(□Š(□Š(
□Š(□Š(□Š(□Š(□Š(□Š(□Š(□Š(
Z…Ì–â?,•»9È©,fyí÷¹□î#•Uµ□»□Ôÿ J—Kÿ •Oø□
´QE □QE □QE □QE □QE □QE □QE □QE □QE □QE □QE □QE □QE □QE □QE □QE □QE □QE
□QE □QE □QE □BúêX&EŒ€□ÉÈ÷«õ"ª□ÇÌ□îÿ Z Ö¢Š(¢Š(¢Š(¢Š(¢Š(¢Š(¢Š(¢Š(
¢Š(¢Š(¢Š(¢Š(¢Š(¢Š(¢Š(¢Š(¢Š(□'ÜÈu#□FÏL{UÚÌOù□7ãü«N€
(¢€
(¢€
(¢€
(¢€
(¢€
(¢€
(¢€
(¢€
(¢€
(¢€
(¢€
(¢€
(¢€
(¢€
(¢€
(¢€
Ë,úw»XØ•¥±Òµ+
×þB þù □Ú(¢€
(¢€
(¢€
(¢€
(¢€
(¢€
(¢€
(¢€
(¢€
(¢€

(¢€
(¢€
(¢€
(¢€
(¢€
(¢€
(¢€
(¢€
(¢€
(¢€
(¢€
(¢€
(¢€
(¢€
(¢€
(¢€
(¢€
(¢€
(¢€
(¢€
(¢€
(¢€
(¢€
(¢€
(¢€
(¢€
(¢€
(¢€
(¢€
(¢€
(¢€
(¢€
(¢€
(¢€
(¢€
(¢€
(¢€
(¢€
(¢€
(¢€
(¢€
(¢€
(¢€
(¢€
æüqeâmG@[O
ÞÁe{,ê²ÜJpc‡□q^□Í□¿†qÍt”P□,ø¯À>%ð7‡n<Saã½RæöÏl“¬Ä…”□□»□õèÙÍ{□„µ™<Cá□+W•
□Kwl’Hª0□cœ{g8¯6ø•-Ïã□b/‡-□a,’HU°Q˜àE í'ØàŸp□© z¶•¦ÛèÚEž™j□,Ò□†<õÂŒ□}ø
”QE □QE □QE □QE □QE □QE □QE □QE □QE □QE Gqÿ -Òÿ ¸□•sµÑ\□Ç´¿î-
å\í tÃ ¢□ÐQ@□□Q@□□Q@□□Q@□□Q@□□Q@□r^ ðÖ¿â□chñ=Î“¢¤j□□7
q#ó’Ò□òŽ˜□9®¶°|M¥kú¤Vë ø—û□Ð±•¾Ã□Ïš

0>r6ãžž´Çèç[ðÀË

Í¯^ëZV§i,Ñ›ö□=»§9/Ô©éøûséÕäz4:Ÿ•> Z□□˜µ‰µÖû%¶¸¥„‘°äBÑ’U□ž›1ïžÞ¹@□ï¯-´Ë
‹ëÉ-
□kxÚIdnŠ dšòM□Wñ-¡ñ;Ã°¥ýõå½†¹□Ü¶úQ□ˆâ·D-Qeè]³¸ŸqZ~6×ô•oÆVþ□Ôuk□□&Ëeæ¬n®
R/´-□;q¸ŒƒÃ6;c¥W×|Wá©>,x>î□□I{;{kÅ-
d¼ŒÇ□d□C08□íš õŠ*8'†êÞ;‹yRXePñÉ□□WR2□#,□ïRP□E□P□E□P□E□P□E□P□E□P□E□P□U5/
øòo¨þunªj_ñäßQüè□□¬Ø□Çì_SüªµY°ÿ •Ø¾§ùP□í□Q@□□Q@□□Q@□□Q@□□Q@□□Q@-j¯¯|EÖu
!c®\è¾□°¸kD’Ç
qy*ýö

~êƒÀÇ_åÒxkÂ—~□¼™ÛÄúÆ«k${D□”¢fFÏÞ□€zdcÞ¼×á❑„uo□x

ÒâãÆ□½…¡–m¶šc,

•æ6âÒ`–$óì1]6…>·á/ˆðxRÿ Z°Öt½FÍî-&¼ÃO
¡ù”¿ñ

□Nœä□Ò«ŠÕ|#â□□k□RÞø°ÿ NÓ□bÒ×Ga
…Àù¤□,I'<t®Ö¨ë:Å†□¤Üjz□ÂÁi□îwoÐ□Üž€w

#Á÷°Þ‹ãíKÁz®«.¯n-+¨Y]Î□œ¨\!G?Ärzû{àz-
p¾□Ò¯ï5]OÆšÌ□m{«□ŽÖÑþõµªýÅoö›ï□ô÷□ÝP□E□P□E□P□E□P□E□P□E□P□E□P□E□P□n¯÷búŸ
éRé□ħéÿ □5□¯÷búŸéRé□ħéÿ □4□vŠ(□Š(□Š(□Š(□Š(□Š(□Mæù□y□<Ý§füíÝŽ3ŽÙ¯>
?□¼G¨@nµ?ˆ□Ôz£□Àiì!µŒúyxù€÷ šôJæ¼eâ¡áÝ>8,âû^·|L:u’òÒÉýãè‹Ôž˜
?

üA©ëÞ□°Me’MGL¿›OžxÆ□f•-
8-á‡å]□sž□ðÁðŸ…àÓ¥›Ï¼vk‹Éÿ ç¤ÎrÇéØ{□]□ y□‡<1yã□Ï□_^xÇÅV†ß\»´Š□□HÇ□FŒ6€¤
□uÇ-•è>□ð×ü#vóÃý·¬ê¾s†ßª]yì˜□Âœ

æ¤øS□¾Ô.ì|[â}<ÐÝIvÐÙÞ^â□9É;BóøžۀQàíS]Ó<k©x/]Ô?µ

□‹ }e|È□F^¶Ò®□R
ëì□ □A¢Š(¢Š(¢Š(¢Š(¢Š(¢Š(¢Š(¢Š(¢Š(¢Š(¢Š(¢Š(¬•Sþ>cÿ
wúÖµdêŸñóû¿Ö€5¨¢Š (¢Š (¢Š (¢Š (¢Š *□©e,Îy`□®%HÙ'□`¦F□!A<

ž2jj(□Éu™üamâ¿□^jú¯Ù□ÿ S□□*ÅÈ†8ö‷‰¬-Ó?Â;
õªóï^¿ò4ø
þÃû!¯A
÷Öqj□□□S—□O□Fæ7(Ø#□

9□ÜW□ÿ
—Ã□ôÛXÿ ÁœßüUwUÇ|KÖî4¯
5žœs«jò®Ÿd ó¾N
{arsØâ€0~□i'□iø‹Y±¸»m□KŸ±éé=ÃÊ□báåË□□ÍÓÓ□½>³|=¢ÛøwÃÖ□= ýÍ¤+□8Æâ:±÷''ñ-*
(¢Š (¢Š (¢Š (¢Š (¢Š (¢Š (¢Š ÌOù□7ãü«N³□þCMøÿ *Ó □Š(□Š(□Š(□Š(□Š(□Š
(

½~çX¶Ó‡ö□„7—
ÒH#Q<¾\q□œÈýÈ□è99®#Â/®Ú|UÖtÍc]¸Ô¶é•ÎT••£³såÆ8P□□ê{šô°óí/þK¾½ÿ `{□ý

€=□¸¯‹7×šwÃ]RæÂî{K□Ð*Í□…□CLŠpÃ‘Á"»ZÆñW‡-|[áË-□òiá,à¡i
:•uqŒ,:¨í@□Çü*ÏúŸ|sÿ ƒ□þÂ»Ëx¾Ïm□>d’yh□|□-l

džæ¼çPø}¯iVS_x□Çž"{èPÈ‘jw"æ□□□ÚT□ŒôÏ8®«Àþ"o□x/KÖä‰b-ê#æ"ô

¬U±í•8

÷ □:(¢€
(¢€
(¢€
(¢€
(¢€
(¢€
ŠêI¢´šKx|ù‛□£‹x_1€árx□<f¥¢€<□ÄS|C×õ]□÷þ□ç‛ý“yö-ŸÛVíæñ□¹ão×šôo□jZæ¥¦Í6³á
ñ£]«•ŽÜÞ¥ÀuÀ!· Āä‛□jÚ¢€1<'uâ□½ %ñE…µŽ¦d`ÐÛ>ä
Ÿ”çss•□ð¬i4-KWø«-¯¨[yzF•i³NË©ó§“ïÉ€r0¿/
r□□ÚQ@□□Q@□□Q@□□Q@□□Q@□□Q@□□Q@□□Q@□□Q@□‰ÿ !¦ü□•iÖrÅ'ö¹}□³ûØã¥hÐ□E□P□E□P
□E□P□E□P□E□P□E□P□W^oõm;Lóô]□ûbïx_³}©-
þ^rÛ›Ž8ãÞ¼ÒÖo^vÞ;¿ñ7ü+Íßk³Ž×ìßÛVāfÓ•Û»çÓ□ì□P□=¬_ø•|□/t}-Üëí□mö
‰Ã"1#z—□C`gœŒâ—]°ñM¾□i>‡§X]ßnSum<Å□Ý§!□¦wc¯jè(□8½¿ø—
â□9tÈ|1eáñ:˜ä¿ŸRK’Šx%□□wc¦k³ðæ…máŸ

ØèÖd˜-"□†n¬z->ä’□□Ô¢€
(¢€
(¢€
(¢€
(¢€
(¢€
(¢€
(¢€
Âµÿ •,□¾kv±-à•o•ŒN□qä©Å lÑE□ QE□ QE□ QE□ QE□ QE□ Wœ|M¶ñ†©=-
™£h□¨hN»õ□†þ;Y'äþës□ªô'□□q‘^□E yÕŸ^¼wck□¥¯Â¨íí¡P‘Ç-»n□□t □®£Ä÷^$µ,ÄøoOµ
½•îÑ.–áö^à9ÜÃæ□Ž=~†·h

kÅ□Ð–Âöw~□□□
□ñucwòùàãiGtŒ-¼sß□Êë□üBñÎœú□Þ•gáÍ>ç
wt÷ës#GžDjƒ,}ëÓè □,í!°±·³·]°ÛÆ±F=□F□è*z(□Š(□Š(□Š(□Š(□Š(
□Š(□Š(□Š(□Š(□Š(□Š(□Š(□Š(□Š(□Š(□Š(□Š(□Š(□Š(□Š(□Š(□Š(□Š(
□Š(□Š(□Š(□Š(□Š(□Š(□Š(□Š(□Š(□Š(□Š(□Š(□Šd¤^‰□□[{□kþt□rŠ§½¿¼□
:7·÷•ç@□(ª{ÛûÇó£{□kþt□rŠ§½¿¼□:7·÷•ç@□(ª{ÛûÇó£{□kþt□rŠ§½¿¼□:7·÷•ç@□(ª{Ûû
Çó£{□kþt□rŠ§½¿¼□:7·÷•ç@□(ª{ÛûÇó£{□kþt□rŠ§½¿¼□:7·÷•ç@□(ª{ÛûÇó£{□kþt□rŠ§½
¿¼□:7·÷•ç@□(ª{ÛûÇó£{□kþt□rŠ§½¿¼□:7·÷•ç@□(ª{ÛûÇó£{□kþt□rŠ§½¿¼□:7·÷•ç@□(ª
{ÛûÇó£{□kþt□rŠ§½¿¼□:7·÷•ç@□(ª{ÛûÇó£{□kþt□rŠ§½¿¼□:7·÷•ç@□(ª{ÛûÇó£{□kþt□r
Š§½¿¼□:7·÷•ç@□(ª{ÛûÇó£{□kþt□rŠ§½¿¼□:7·÷•ç@□(ª{ÛûÇó£{□kþt□rŠ§½¿¼□:7·÷•ç@
□(ª{ÛûÇó£{□kþt□rŠ§½¿¼□:7·÷•ç@□(ª{ÛûÇó£{□kþt□rŠ§½¿¼□:7·÷•ç@□(ª{ÛûÇó£{□kþ
t□rŠ§½¿¼□:7·÷•ç@□(ª{ÛûÇó£{□kþt□rŠ§½¿¼□:7·÷•ç@□(ª{ÛûÇó£{□kþt□rŠ§½¿¼□:7·÷
•ç@□(ª{ÛûÇó£{□kþt□rŠ§½¿¼□:7·÷•ç@□(ª{ÛûÇó£{□kþt□rŠ§½¿¼□:7·÷•ç@□(ª{ÛûÇó£{
□kþt□rŠ§½¿¼□:7·÷•ç@□(ª{ÛûÇó£{□kþt□rŠ§½¿¼□:7·÷•ç@□(ª{ÛûÇó£{□kþt□rŠ§½¿¼□:
7·÷•ç@□(ª{ÛûÇó£{□kþt□rŠ§½¿¼□:7·÷•ç@□(ª{ÛûÇó£{□kþt□rŠ§½¿¼□:7·÷•ç@□(ª{ÛûÇ
ó£{□kþt□rŠ§½¿¼□:7·÷•ç@□(ª{ÛûÇó£{□kþt□rŠ§½¿¼□:7·÷•ç@□(ª{ÛûÇó£{□kþt□rŠ§½¿
¼□:7·÷•ç@□(ª{ÛûÇó£{□kþt□rŠ§½¿¼□:7·÷•ç@□(ª{ÛûÇó£{□kþt□rŠ§½¿¼□:7·÷•ç@□(ª{
ÛûÇó£{□kþt□rŠ§½¿¼□:7·÷•ç@□(ª{ÛûÇó£{□kþt□rŠ§½¿¼□:7·÷•ç@□(ª{ÛûÇó£{□kþt□rŠ
§½¿¼□:7·÷•ç@□(ª{ÛûÇó£{□kþt□rŠ§½¿¼□:7·÷•ç@□(ª{ÛûÇó£{□kþt□rŠ§½¿¼□:7·÷•ç@□
(ª{ÛûÇó£{□kþt□rŠ§½¿¼□:7·÷•ç@□(ª{ÛûÇó£{□kþt□rŠ§½¿¼□:7·÷•ç@□(ª{ÛûÇó£{□kþt
□rŠ§½¿¼□:7·÷•ç@□(ª{ÛûÇó£{□kþt□rŠ§½¿¼□:7·÷•ç@□(ª{ÛûÇó£{□kþt□rŠ§½¿¼□:7·÷•
ç@□(ª{ÛûÇó£{□kþt□rŠ§½¿¼□:7·÷•ç@□(ª{ÛûÇó£{□kþt□rŠ§½¿¼□:7·÷•ç@□(ª{ÛûÇó£{□
xþt□rŠ§½¿¼□:7·÷•ç@□(ª{ÛûÇó£{□kþt□rŠ§½¿¼□:7·÷•ç@□(ª{ÛûÇó£{□kþt□rŠ§½¿¼□:7
·÷•ç@□(ª{ÛûÇó£{□kþt□rŠ§½¿¼□:7·÷•ç@□(ª{ÛûÇó£{□kþt□rŠ§½¿¼□:7·÷•ç@□(ª{ÛûÇó
£{□kþt□rŠ§½¿¼□:7·÷•ç@□(ª{ÛûÇó£{□kþt□rŠ§½¿¼□:7·÷•ç@□(ª{ÛûÇó£{□kþt□rŠ§½¿¼
□:7·÷•ç@□(ª{ÛûÇó£{□kþt□rŠ§½¿¼□:7·÷•ç@□(ª{ÛûÇó£{□kþt□rŠ§½¿¼□:7·÷•ç@□(ª{Û
ûÇó£{□kþt□rŠ§½¿¼□:7·÷•ç@□(ª{ÛûÇó£{□kþt□rŠ§½¿¼□:7·÷•ç@□(ª{ÛûÇó£{□kþt□rŠ§
½¿¼□:7·÷•ç@□(ª{ÛûÇó£{□kþt□rŠ§½¿¼□:7·÷•ç@□(ª{ÛûÇó£{□kþt□rŠ§½¿¼□:7·÷•ç@□(
ª{ÛûÇó£{□kþt□rŠ§½¿¼□:7·÷•ç@□(ª{ÛûÇó£{□kþt□rŠ§½¿¼□:7·÷•ç@□(ª{ÛûÇó£{□kþt□
rŠ§½¿¼□:7·÷•ç@□(ª{ÛûÇó£{□kþt□rŠ§½¿¼□:7·÷•ç@□(ª{ÛûÇó£{□kþt□rŠ§½¿¼□:7·÷•ç
@□(ª{ÛûÇó£{□kþt□rŠ§½¿¼□:7·÷•ç@□(ª{ÛûÇó£{□kþt□rŠ§½¿¼□:7·÷•ç@□(ª{ÛûÇó£{□k
þt□rŠ§½¿¼□:7·÷•ç@□(ª{ÛûÇó£{□kþt□rŠ§½¿¼□:µ□ÌkŸJ u□Q@□□Q@□□Q@□□Q@□□Q@□□Q@
□□Q@□□Q@□□Q@□□Q@□□Q@□□Q@□□Q@□□Q@□□Q@□□Q@□□Q@□□Q@□□Q@□□Q@□□Q@□□Q@□□Q@□□Q
@□□Q@□□Q@□□Q@□□Q@

›ýSUJ·7ú¦ª" QE QE QE QE QE QE QE QE QE QE QE QE QE QE QE
 QE QE QE QE QE QE QE QE QE QE QE QE QE QE QE QE QE QE
 QE QE QE QE QE QE QE QE QE QE QE QE QE QE QE QE QE Q
E QE QE QE QE QE QE QE QE QE QE QPÝÌmì§•@-
là-ø¯ð×Š>,ø»L}KI:Aµ4_¼@§#ãÓš ÷*+"ðWü&ž]ïü&`Ý"û7Ù:ã•Û»w•t—Ö-
xûMÔnéæÈ?• OEV·Ô,®Ü¥µå¼ÎJÇ*±×ƒN¸¾´³ÇÚn ƒwO6@¹üèz*µ¾¡evå-¯-
æp2V9Uˆ-¼|——°Ì°Ës
Jÿ uÀcô5VŒuÑáŸjš¾Fûx ‹#
È~TÏ¶â(f9¢›•*>Æ*ÛX¤uÐÓëÍ¾-ÛÂðŠMná

·SÛËªÏ–
É□•Ü£>ê□ñ&¹W×¼okà□□ˆmâ/3Ì˜4šY·Q „ÈP(=sÓž¸=r2@=Î'ÝcFw`¨£%‰À□Ö¸ÿ □xîßÃ¾
‹X□|Ë»èÔX['"#°äd

Ã9?—qSøkEÕ.<
ý•âË¹/o/¢qt□ËY□<±•ØÏ8ã□□Ô$‰,k$n®Œ2¬§
•cN¯2ø3¨O□"«xZõ÷]h7•□{□Ë61ëó+þ□W¦Ð□E□P□E□P□E□P□E□P□E□P□E□P□E□P□E□P□E□P□
E□P□E□P□E□P□E□P□E□P□E□P□E□P□E□P□E□P□E□P□E□P□E□P□E□P□E□P□E□P□E□P□E□P□E□P
□E□P□E□P□E□P□E□P□E□P□E□P□E□P□E□P□E□P□E□P□E□P□E□P□E□P□E□P□E□P□E□P□E□P□E□
P□E□P□E□P□E□P□E□P□E□P□E□P□E□P□E□P□E□P□E□P□E□P□E□P□E□P□E□P□E□P□E□P□W#ÿ V
¿J§W#ÿ V¿J u□Q@□□Q@□□Q@□□Q@□□Q@□□Q@□□Q@□□Q@□□Q@□□Q@□□Q@□□Q@□□Q@□□Q@□□Q@
□□Q@□□Q@□□Q@□□Q@□□Q@□□Q@□□Q@□□Q@□□Q@□□Q@□□Q@□□Q@□□Q@

›ýSUJ·7ú¦ª” QE□ QE□ QE□ QE□ QE□ QE□ QE□ QE□ QE□ QE□ QE□ QE□ QE□ QE□ QE□
QE□ QE□ QE□ QE□ QE□ QE□ QE□ QE□ QE□ QE□ QE□ QE□ QE□ QE□ QE□ QE□ QE□ QE
□ QE□ QE□ QE□ QE□ QE□ QE□ QE□ QE□ QE□ QE□ QE□ QE□ QE□ QE□ QE□ QE□ QE□ Q
E□ QE□ QE□ QE□ QE□ QE□ QE□ QE□ QE□ QE□ QE□□WSÿ •Mçýp□ý□×Ï¿

¼Qã=#ÂÒZè^□þÔ³ûT•ö•Ä|Ä.Wðãó¯ µ N•x É0?þ,kÁþ□üOðß„|#&™ªËr—?k’\G
a´…Ç?• □^ðö·®_xZãQÖ´OìÛø¼Â¶»÷oU□□Û'#-Õäž□ð

™ñ'DÔ<GâMJöãQžéÑŒs□òp□□□}x□ Ç□é°_□´¿□èzÓørí’êÎÙ□™s-
±³+lbO□Êœý+æÍ□4y4Õ7□Þ#

Ä¥ÓØ²´RŒä

cŽ□È$ú÷Å zoÀý"ÊÊ/□ë1D×7v

ðA:±Ù2c8
õQÿ }T-□ð

™ñ'DÔ<GâMJöãQžéÑŒs□òp□□□}x□ Ç□Öü?ñ□†'ð^§¤x?ÌÓïlí¥”®¡µ_~ÜyÎÝ□Î2{qÀ□¯

Ñ□G‟MSqmâ0ìJ]=‹ +E(Î@Æ8àŒ,O¯|P□§|

Ðíâ□ÄZ•šyú□£½--Îò#‘HÈã§U□?íV□ƒ´□□ø ^Aã□bþÓÅ□pë ¸›Ê9Î□

□ž0yö¯FøuâßՈÛxGP´ðÝ¥äo¥À÷SÙÎ£í□`rü□18□¶8□□□ÈxËâ□Ãß□h□&]□íµ¶ˆ-³ý□VE“□R][
•□±ÏÒ€=ÇFÓWGÑl´ÕžIÖÖ□^K)Ë0QŒšó¿•·/□ÃØ"F!n5□¢|w]®ßÍEtÿ □mu;/‡z5¾°%[Ô„îY~
ò®âQO¦□hÇn•Ï|w°{¿†í:t²¼Šá¾œ§þÎ(□·“E,ëÂ–¡É•·–
Ëì„§P¥6ñøW•Oà□□Eà¥ð†¥w¤[øròC,Ú˜vó

ÅÈÚF2:þ□žçÖt□åÕ<;¦ß©âæÖ)□i¥□ú×□xûQ°ñ§Š-
þ-hÒ”‡"mbå?åœc□g×§âTzÐ□'iâ+=CÅ□^*Ôlnî4ûy>Ãá-&□Üó2`y˜<qÇ<üÄ□□•ë^□ñÅŸ‰¯/t
æ³»Óµ[,□ì®Ô+€z0ÇQÈçÜzŠâ<iiiàß□x#UhŒ-±•ìË*-X

}wg=NÓSø:x¼Gñ«Ä-$Ò‰—HK$µûR©
,˜□□ž¸Ú□!ê(□ž□ÿ Bý¢¼Wi□Md²²ŽîD-
Ÿüy¿:õ°ò?□gVøíã=Z>a¶ˆY±ÿ l□_ý¤Õë” QE□ QE□ QE□ QE□ QE□ QE□ QE□ QE□ QE□ Q
E□ QE□ QE□ QE□ QE□ QE□ QE□ QE□ QE□ QE□ QE□ QE□ QE□ QE□ QE□ QE□ QE□ QE□
QE□ QE□ QE□ QE□ QE□ QE□ QE□ QE□ QE□ QE□ QE□ QE□ QE□ QE□ QE□ QE□ QE□ QE□
 QE□ QE□ QE□ QE□ QE□ QE□ QE□ QE□ QE□ QE□ QE□ QE□ QE□ QE□ QE□ QE□ UÈÿ Õ¯
Ò©ÕÈÿ Õ¯Ò€□E□P□E□P□E□P□E□P□E□P□E□P□E□P□E□P□E□P□E□P□E□P□E□P□E□P□E□P□E□P□E□P□E□P□
E□P□E□P□E□P□E□P□E□P□E□P□E□P□E□P□E□P□E□P□E□P□E□P□E□P□&ÿ TÕR-Íþ©ª¥ □QE □Q
E □QE □QE □QE □QE □QE □QE □QE □QE □QE □QE □QE □QE □QE □QE □QE □QE □QE □
QE □QE □QE □QE □QE □QE □QE □QE □QE □QE □QE □QE □QE □QE □QE □QE □QE □QE
□QE □QE □QE □QE □QE □QE □QE □QE □QE □QE □QE □QE □QE □QE □QE □QE □QE □QE
 □QE □QE □QE □QE □QE □QE □Dè°S□N™fIä‟□ÿ …^¢€1u¯□[ê-□Õ4›(á³kÛw‹|q… •@'□G
ô¯,Ð.þ'ø'G‡Ãðø>Òö
bË

ñ¿Þ□‰É!¹ëè□-{m□□ä:Ãï□ë7>&×¼JÖö□¦µbÖqÛÀr±,□nl□ÇÈ£□'□çš¡]üOðN□□‡áð}¥ì□Å–
□ã□¾
□’Cs×ÐZöÚ(□Í<□á?□§‹uO□ø¦;{]Fú□□Ú[□B/ËÉÁ=‘qÉ=sÍz□±´□h□°
ÿ ç¨ŒnüúÕŠ(ª□Þ"□»¡ßiW<Ew
DÇ□Û‘Á-ààþ□~Š â>□ÙkzGƒÆ•®Y¼□X\I

.X0-<î

□¦I@+¨±ÑtÍ2îòîÊÆ□./_Ì¹−4ÃJÜòO~§ó5~Š l‘G4m□¨®Œ0U†Aü*•óI¦è·Ri¶BY¡•Ú
hÀPî□!Ga“W¨

□áO…¯<7áy&ÕQ—
VÔgk›-ä□^Ê¤Ž¼dýX×wE□ QE□ QE□ QE□ QE□ QE□ QE□ QE□ QE□ QE□ QE□ QE□ QE□ QE
□ QE□ QE□ QE□ QE□ QE□ QE□ QE□ QE□ QE□ QE□ QE□ QE□ QE□ QE□ QE□ QE□ QE□ Q
E□ QE□ QE□ QE□ QE□ QE□ QE□ QE□ QE□ QE□ QE□ QE□ QE□ QE□ QE□ QE□ QE□ QE□
QE□ QE□ QE□ QE□ QE□ QE□ QE□ QE□ QE□ QE□ QE□ QE□ QE□ UÈÿ Õ¯Ò©ÕÈÿ Õ¯Ò€□E□
P□E□P□E□P□E□P□E□P□E□P□E□P□E□P□E□P□E□P□E□P□E□P□E□P□E□P□E□P□E□P□E□P□E□P□E
□P□E□P□E□P□E□P□E□P□E□P□E□P□E□P□E□P□E□P□E□P□E□P□$RÑ•:Ô-LŸÝýjÕ□□WÉ
“û¿--LŸÝýjÕ□□WÉ“û¿--LŸÝýjÕ□□WÉ“û¿--LŸÝýjÕ□□WÉ“û¿--LŸÝýjÕ□□WÉ“û¿--LŸÝýjÕ
□□WÉ“û¿--LŸÝýjÕ□□WÉ“û¿--LŸÝýjÕ□□WÉ“û¿--LŸÝýjÕ□□WÉ“û¿--LŸÝýjÕ□□WÉ“û¿--LŸ
ÝýjÕ□□WÉ“û¿--LŸÝýjÕ□□WÉ“û¿--LŸÝýjÕ□□WÉ“û¿--LŸÝýjÕ□□WÉ“û¿--LŸÝýjÕ□□WÉ“û¿
--LŸÝýjÕ□□WÉ“û¿--LŸÝýjÕ□□WÉ“û¿--LŸÝýjÕ□□WÉ“û¿--LŸÝýjÕ□□WÉ“û¿--LŸÝýjÕ□□W
É“û¿--LŸÝýjÕ□□WÉ“û¿--LŸÝýjy_Ë‰Ü

y□¡@□|™?»úÑäÉýßÖ ¸Ô^□‡Œ"□½ÍEý-'üóOÖ€.y2□wõ£É“û¿-SþÖ“þy§ëGö´ŸóÍ?Z ¹äÉýßÖ
•&OîþµOûZOùæŸ-ÚÒ□Ï4ýh□ç“'÷□Ž<™?»úÕ?íi?çš~´□kIÿ <Óõ
žLŸÝýhòdþïëTÿ µ¤ÿ žiúÑý-'üóOÖ€.y2□wõ£É“û¿-SþÖ“þy§ëGö´ŸóÍ?Z ¹äÉýßÖ•&Oîþµ
OûZOùæŸ-ÚÒ□Ï4ýh□ç“'÷□Ž<™?»úÕ?íi?çš~´□kIÿ <Óõ
žLŸÝýhòdþïëTÿ µ¤ÿ žiúÑý-'üóOÖ€.y2□wõ£É“û¿-SþÖ“þy§ëGö´ŸóÍ?Z ¹äÉýßÖ•&Oîþµ
OûZOùæŸ-ÚÒ□Ï4ýh□ç“'÷□Ž<™?»úÕ?íi?çš~´□kIÿ <Óõ
žLŸÝýhòdþïëTÿ µ¤ÿ žiúÑý-'üóOÖ€.y2□wõ£É“û¿-SþÖ“þy§ëGö´ŸóÍ?Z ¹äÉýßÖ•&Oîþµ
OûZOùæŸ-ÚÒ□Ï4ýh□ç“'÷□Ž<™?»úÕ?íi?çš~´□kIÿ <Óõ
žLŸÝýhòdþïëV"mñ#‛,Ê□:€*ù2□wõ£É“û¿-Z¬¦ÕdV#Ë^

(□ß"'÷□Ž<™?»úÕ?íi?çš~´□kIÿ <Óõ
žLŸÝýhòdþïëTÿ µ¤ÿ žiúÑý-'üóOÖ€.y2□wõ£É"û¿-SþÖ"þy§ëGö´ŸóÍ?Z ¹äÉýßÖ•&OîþµOûZOùæŸ-ÚÒ□Ï4ýh□ç"'÷□Ž<™?»úÕ?íi?çš~´□kIÿ <Óõ
žLŸÝýhòdþïëTÿ µ¤ÿ žiúÑý-'üóOÖ€.y2□wõ£É"û¿-SþÖ"þy§ëGö´ŸóÍ?Z ¹äÉýßÖ•&OîþµOûZOùæŸ-ÚÒ□Ï4ýh□ç"'÷□Ž<™?»úÕ?íi?çš~´□kIÿ <Óõ
žLŸÝýhòdþïëTÿ µ¤ÿ žiúÑý-'üóOÖ€.y2□wõ£É"û¿-SþÖ"þy§ëGö´ŸóÍ?Z ¹äÉýßÖ•&OîþµOûZOùæŸ-ÚÒ□Ï4ýh□ç"'÷□Ž<™?»úÕ?íi?çš~´□kIÿ <Óõ
žLŸÝýhòdþïëTÿ µ¤ÿ žiúÑý-'üóOÖ€.y2□wõ£É"û¿-SþÖ"þy§ëGö´ŸóÍ?Z ¹äÉýßÖ•&OîþµOûZOùæŸ-Y³½{™Y□□ ¹â€äÉýßÖ¬
! =iÔP□E□P□E□P□E□P□E□P□E□P□E□P□E□P□E□P□E□P□E□P□E□P□E□P□E□P□E□P□E□P□E□P□E□P□E□P□E□P□E□P□E□P□E□P□E□P□E□P□E□P□E□P□E□P□E□P□E□P□E□P□E□P□E□P□E□P□E□P□E□P□E□P□E□P□E□P□E□P□E□P□E□P□E□P□E□Å|MñÃøÃIqi
Ï©]Ê ´‰¹□ˆÉb:•=□r(□µ¢¼JëDøÍc¤>°Þ(·–ê8ŒÒiё□ž ÉP6m-
ì?□kÐ¾□xÀxßÁöú³F']□hn£LíYW□Æ{□AÇlãš êè¢Š (¢Š (¢Š (¢Š (¢Š (¢Š (¢Š (¢Š (¢Š (¢Š Žãþ=¥ÿ pÿ *çk¢¸ÿ •i□Ü?Ê¹Ú (¢Š (¢Š (¢Š (¢Š (¢Š (¢Š (®□5_□øÇVÔcðþ£o£é6□□kö··□Ë<«÷°-ÀQþ}□•
[×tß□□

x−x.ä¸€Ïc□

~_š□i#(à0□ñýh□¶¢ªêZ□®'¦Üj□²□í-ã2HÇ°×Ú¸_□ø‹Äz—Œîmµ™□;[□8_ÛÙ^Ô□ui6¨-
Œ"·"žæ€=□Š+□ñV»«¦½¦xoAkxo¯'æ{«…Ü°Æ¾‹Ý□ùë□□×Ñ^qªÞøËÁ+m©êŽÝ¶¹¦4é□ÄFÍ`'□□e
6õç×ÿ Õèô QE□ QE□ QE□ QE□ QE□□«¤ýÉ~¢´k;Iû'ýEhÐ□U5/øòo¨þunªj_ñäßQüè□□Š(
□Š(□Š(□Š(□Š(□Š(□Šk°Ç□;°UPI'°¯<Ó/¼cã‹wÕô½^ßBÒ]Ùlôú,òÌªHŰû¸\•z□ðÈ□¢Ñ
\‡„õýZ]cQðß^D□ªXªÊ—□□©s
tlv à¯µ.¥iãmST¹K□NËDÓ¢m°È-ÅÌÓq÷^o•F□□ ë¨®/Â°Þ¸ž'Ô|/â□ °°¶•na¼•6
c'-
2ô□'·¿Ö»J (®'ZÖ5ýOÅïáŸ□\ÛiæÚÙn.¯g‹Ía¸áU□ð}r□LsTj^+ð§^´›=oS¶ÖtíR□ᵝ,Ël –
)□ãå^□ÿ <w ô
(¢€
(¢€
(¢€
(¢€
(¢€□>úýk¥®i>úýk¥ □Š(

Ý_îÅõ?Ò²ëSWû±}Oô¬° (¢Š (¢Š (¢Š (¢Š (¢Š (¢³<E¬'‡ü=□«<fQk • 8Ü{

öç□□§Eyý¥‡Ä=SO‡S-(°³’xÄ©b-
ñ¨#!Kœ·×□-o
kWž-ð•Ã]fÇPI%³šKn‰"ñ½3ŸP{ó@□U□Ç□Â□«□Ð ï®□à?þ&ªx□õ;□c_’mfïQÒí§□-
Ïs·/"ÿ ¬a€8□€=h□¼¢□Œ,2F{Šãÿ á
Õ¿èw×?ò-
ÿ □@□□□ç¾□MY>!ê□#Ä□úŽ›§[*Ü□□¤□ß□£ t^O¿□èT QE□ QE□ QE□ U-;þ?Sè□•U«Zwü~§Ð
ÿ * Ü¢Š(¢Š(¢Š(¢Š(¢Š(¢Š(¢Š(¬•[ýz□›ýkZ²uoõéþïõ

ú(¢€
(¢€
(¢€
(¢€
(¢€
(¢€
(®WÄZÆ½æÝØèv-OÙâ2M©]¯î‟åÝˆ×¬••À-´□ÕQ\ÿ •õ;½gÁZV¡☐⁄›u<;¤}¡w□‘Ð □JÊÖu½sUñ
L¾□ðÌ¶ö•i□Ë☐˙M™å□åQW¡b9çúP□kEyëê¾)ðn³§Gâ□JßWÒ5
Å¯ÚÖØA$□7ÝÊ¯□Oøþ:ž(ñ□ªºå—†|:˚□Rê#q-Äãr[B

7□Ü‟Àÿ ëÐ□]EyÎ«}ã?□Ã-¯©êöúî□²*Þ ³X$…Xãríë,G_þ¸ôHÝe□dF

Œ□)□□4 ê(¢€
(¢€
(¢€
(¢€
(¢€
(¢€-ißñúŸCü«r°ôïøýO¡þU¹@□7ßñû/Ô□*¯V/¿ãö_¨þU^€
(¢€
(¢€
(¢€
(¢€
(¢€
(®SÅþ"Ô4ûÍ3DÐá†McTw□4ùòáE□gluÀè>½z□

®Šó•Z_□ø>ÈëwZÕ¶¹c□□wiö%□•3,Q—
®=ëkÇ□ýÍ‡Ãë□gF¹□Ìë□A6ÀØW‘□pÃ□□½ u”W□ÿ □ÏŽ□e¡ÿ å□□ñ®Ê□’8#IdódU□äÛ·qÇ'□³@
□QP^ÞA§ØÏyrâ8 □¤‘□e□$×□c'Ž<[l°¥®©máÝ>a¾Ö²-ÄÏ□èÏ¸àdsÇj ô
+3BƒW¶Óü-nöÞòé\íž□¼°ÉÆ2½□ëÓŽ•§@□□Q@□□Q@□□Q@□□Q@□□Q@□□¿ü{EþàþU%Goÿ -Ñ□¸?
•I@□soþ±¾¦°Jæßýc}M 6Š(□Š(□Š(□Š(□Š(□Š(□Šáõ□kÄ□÷Š¯t□□\[ØC§*□ÝBh¼Ö

ã!□□□Ž¹ý;Åm«x—Âþ&Ó´¿□ßÁªiú›˜mï’□
Ç0□
Ê¼`ô-
ýj ïh¬□oÃ×Ú-êÏmâ=KNA□C□¶Í¤ä□Ü,sÏé\o‹´½s@Ó-¾Åã□fãQ½¹ŽÖÒ□1á□□$ás€2sô □Q¢£
•-;xã’C#ª€ÎF
□95•¯h—zÁ€Úë—Úg•»pµÛûÌã®Aé□Ö€6h¯5ñN‘©økÃ—š£xÓ[‘â\C□îÿ y!8Uû½É†k¹Ð
¾¶ðý„:œæ{õ□~Ñ#u/Ž□^?
 Ñ¢Š(¢Š(¢Š(¢Š(¢Š(«úWü|¿ûŸÔU
¿¥□ÇËÿ ¹ýE kÑE□ QE□ QE□ QE□ QE□ QE□ QE□ QE□ QE□ QE□ QE□ QE□ QE□ QE□ QE□
 QE□ QE□ QE□ QE□ QE□ QE□ QE□ QE□ QE□ QE□ QE□ QE□ QE□ QE□ QE□ QE□ QE□ QE
□ QE□ QE□ QE□ QE□ QE□ QE□ W□|`ùþ |7ŽAû£©ò□CûØsúW°×œüað†£â]□Êÿ DRÚ¾“?Ú
U8fS•Á□ÚÊ©ÿ €ã© □Fë^;û<dxcZEÿ P5□Ùÿ |□éŠ«sñsÅš-’úU□□uHuécòŒ›dLF
€W#×□àw'□Ý|/ð|¾
ðT□uÑS}4•su´ä
□□□è□•¨4□ÙQE□ QE□ QE□ QE□ QE□ QE□ QE□ QE□ QE□ QE□□□Çü{KþáþUÎ×
Eqÿ -Òÿ ¸□•s´ QE□ QE□ QE□ QE□ QE□ QE□□æšUÎ±ðþûUÓçðö¥ªiw7’]ÚÜé±‰˜□êŒ¹□Æ:
ýjµÞ³¨ëŸ□ü□,°□Ð—
=×”×eD’•+æÊ

T

:õÍt·Þ6½Ó¯î-&ð~½1I□bšÒ□4r®xmÙ□r9Çjg‡ôÝ_Vñ+x£_µ□&8M¾Ÿa¼;BŒrÎäq¸ð1ØP□Z—
üV~.]□~m□G‘e¿?Ã=ÇT‡Ü/Þaë€im¿äµ^ÿ Ø

?ý□S^|-ðmýì÷—:6û‹‰□Y_í3□ÌÇ$à>□'µs□ü-ðÿ ü,+‹VÐf□*éË$mæÍ³Ïó0~}Ü•½³øP□«×□ñ
m'°Ób³7Ÿð”¡i4ß°□æ(èÅ÷|¢>Ç?‡zèõ=bòÃ[Ò¬-ô{‹»{Æušî2v[□
pzç¹□;×7¯[ë:□•□Š,t©uk)ì…¥Ä6äyÑa·□PzƒÇ□Þ€0î'Ö-5-:□‰□îÓ!tkil°m□~Í8ûÙÏC÷sÛ
□ë ,□□ ×šøƒZÖ<m£O i~□Õ->Ùµ%°Õa□G
ä□Àd-<v¯D³¶□v6öªÅ–□Ö0Ç©ÀÆ□J šŠ(□Š(□Š(□Š(□Š(□]'îKõ□£YÚOÜ—ê+F€
©©□Ç‟}Gó«uSRÿ •&ú□ç@□”QE □QE □QE □QE □QE □QE Gq□ÜÛK□çl¨PãÐŒWšøoXÖü□¤Gáí
SÂú½ð´f[{½2□2J…‰□ò6õ¯N®!¼{©FZ <□â¶□€©
´$ÿ ×\ã-ø

•□PÔ5oŒkww¤M¥ÿ Ä□,Å3†v□Îá˜□°s‛ƒé]_ˆ¿á1óÓþ□³¢□<¿Þ}¿Íß¿'îíã□ÇZ-á=□S]CPñ□¼
‛Çª_…•m£mËk
ýÔÏs“’zf«\xÃ]Ò5
«}OÂ:□Ô□Vû5Î”,q$yùw.r§□Ï={P□o,''ËÅ°…—
ˆíç□Å□‛‰MÃº¼SÂ¼□□Ð□□ýÞ¼r}=□¸="×Wñ/Ž-
üK¨iréV□□ï□¤□□yÒ³ðÌÊ>èÇjèßY¼_□Ç£•-á¬ÚØÌu□O-
-’6tÆxõÏ=;Ð□%ã43ø®Üø]®G‹a„□h□ù+□9ÅÆî1Ü

¾Ý*®›s{mã{Y<}□-û1‹Iš,□□f□!{‰□O›Ÿ§□r_íŸ□øÏZÔÓD»Õô½XÇ&û□-h]□□Ju#"úT:ÅÆ¯ñ□
ôý6ÛÃºŽ™§Åw□ÍÍÞ¥□‰”!Î□2I'Ö€=*Š(□Š(□Š(□Š(□Š(□O¾¿ZékšO¾¿Zéh ¢Š(□7Wû±
}Oô¬°ÔÕþì_Sý+.€
(¢€
(¢€
(¢€
(¢€
(¢€
Î×¤Ó"Ðo›Yh×MòX\y□6□,8ç<ñŽsÒ´k™ñöƒwâ?□]XX2
-É,Jç
å□6ÓõÇçŠ á-!ñå¾…$z□Þ□eE²\”]A`ïåö□tÝótÀ®÷À×□□Þ□,=□dŽÚ□häŠa‰c—9a
?Ç""õô¬˜þ ji□ŠïÀþ"□Àa-□pðîö"==ñW< £jV)«jš´ ky«]›"jŒ□B¸À□Ž
uÍ [ñÆ¹&ƒák‰-r×÷□ZÙ êÓ?
•§'ð«ž□Ðãðç†ìt¨È&□À'Çñ¹å›ñbMa^Y]ëß□-Lö³&"¡Ãç#É□ =ËŽ6"Ã□^ã¡®Ò€
Íñ

³□‡¼?}«\ce´EÂ"÷›¢¯âH-
•A¡k7š´Ú’]è÷□rÚ\´14ÄâáGñ¯□ƒø•zÄñe•ß^|Q¡è_e›û*'û}ôÆ3å¾Ã„•wBKu-˜4□wÀZ<úO†
"′÷'R¿v½¼b92ÉÎ□Ð`~□ÓÑE □QE □QE □QE □kNÿ ¯•ÔúåUjÖ•ÿ -
©ô?Ê€7(¢Š (¢Š (¢Š (¢Š (¢Š (¢Š (¢Š +'Vÿ ^ŸîÿZÖ¬•[ýz□›ýh□>Š(□Š(□Š(□Š(
□Š(□Š(□©êÿ ò□¿ÿ ¯y?ô□W+‘ñGŠÛOkÍ-<7â□×x
-Å□□™ ,¿ÞÏlóÅ ?áŸü"•□þ¸□iÆ¨x□æñg••¿Ö□ETÿ °□íªŸ

µë‹]□Hðåß†üAm<q”{™ìJ@¤nnXœ•Û§Z—PƒWð‡•oõÛ□&ãTÒud•íPZ ÓC*

□Uþ F□□Û□ □|\ñ@ÌÃýjÜÀcÿ {Ìýz4¿›ã>¼_ï&™□§û¤,□Ž©tu□ˆ□¦›nú%î“ ÙÜ-ÝÃê□#-á—
î Lž3Ôÿ ‡7<Oa«hþ/µñn□`ú,}˜ÙßÙÄq#G»r°z□{{□R@□¯Ä□Fø□®‰1□±ÈyõÇ-
®*÷…Ë·„tS&w›□
g×Ë\×□-_ë~?³]□ÇÃúž“cpëöëÍJ!
XÁ□ª.Nâqþs‘èÐÄ•A□1(XãPª£°□ P□è¢Š (¢Š (¢Š (¢Š (¢Š (¢Š µ§□Çê}□ò
-ÊÃÓ¿ãõ>‡ùVå `ß□Çì¿Qüª½X¾ÿ •Ù~£ùUz (¢Š (¢Š (¢Š (¢Š (¢Š +ˆñž□«[ø‡EñF□doß
O□Eqh-‡’7□Êú‘ÏJíë□Ä:íÞ„¶òÁ¡ßê□ÈXJl€w‹□ÇÉÔçŸ¦=è□□ñ•/õO
jÖÖþ□ÖâW²™f-þ%·Ž$Øw6I;°2p:Ö…·‡¦ñ/ÁÝ/G7?e-
k□R%dß·nÇ□dv\UmJ÷^ñå¿ö5¦‡□£iS□/o5□□ÊcÎJ"dœž™é]Nµ¨\ø{L¶m7D¸Ô¢F□46¬□Å□S,□
ûÝ Ç½ swšwÄ=-ÖKë□□Zk□□.Ö—□zC¼

H

‡9ôé]O†µÈ|IáÛ□^□1¥Ì{Š□•¬

3ß□□\½çŒõÍ^Ò[□□ÁúÔ7‟)ŒO©B-â‹<n$‟œuÅméZkø3ÀqYÚÀ÷óXZ³□¢á§‟-
!zõbqÖ€3þ+Jðü2ÖZ2C□•N=

¨□èMV‡EñõÅ¤R/Š4í;j□.ÒÛO□Æ□8□Üîý+^úÎ_□x□kk»I4éõ
^` ›–…ú®î□à€z
ÀÓ<e¯éZ\:f§àÝjãS·ŒEæÚÄ- ˆ□□æg□=úÐ□Ï,|E□¬Á¨Øë□E-«¥Ü›kƒ□Ü"û®3Ó<þ]°

ª¹O□èz†™o¨êZÀ•5MVäÜÏ□g+□Æ□3ß□¿¿ã]] □QE □QE □QE □QE □QE tVÿ ñí□ûƒùT•□¿ü{
EþàþU% □Í¿úÆúšé+›□ð•õ4 Ú(¢€
(¢€
(¢€
(¢€
(¢€
(¢€<êoí□□xÓXÔ¢Ñ/5m#W1ÊMŠï–
□`,□ÁÉ?•ex»Ä:Ž¿7†Ñ|7¨éÖË®Z²_…□Ì™8Q-Ié“Ÿjìµ•□]èÚ¤¶¯ámjöÜ□1\ØB
&WÈ□ÈÈ+ƒ‘Ï¦k:Æ×Yño‰,u•_N}/JÓKIig3□4Ò‘•1Àû

àuÍ w5ÂØŸøIþ&Ý_Ÿ›Oðò□X=□åÇï□□°>_Ê°o□j□:W‡o¯l-f°»Ž#äC

eÙœð¼

HÉ□û□Tü□ ·‡|+ge1ÝvÀÍtää´ÏË□{óÇà(□ ¢±¼C¬ÞhÐÙ½–•q©´÷)
¬□æ%9Ëž□□-ÃÜV•åÇØì§¹ò¤—É□¤òâRÎø□Â□ÔŸJ ã5ƒÿ
7Ä};F_šÇDQ¨^z□•□¡÷□·Ò»ªä¾-
iWvz,úž©□GªêóµåÊ¸Ã&~â`ò0½»dŠëh ¢Š(¢Š(¢Š(¢Š(¢Š(«úWü|¿ûŸÔU
¿¥□ÇËÿ ¹ýE kÑE□ QE□ QE□ QE□ QE□ QE□ QE□ QE□ QE□ QE□ QE□ QE□ QE□ QE□ QE□
QE□ QE□ QE□ QE□ QE□ QE□ QE□ QE□ QE□ QE□ QE□ QE□ QE□ QE□ QE□ QE□ QE□ QE
□ QE□ QE□ QE□ QE□ QE□ QE□ QE□ QE□ QE□ QE□ QE□ QE□ QE□ QE□ QE□ QE□ QE□ Q
E□□□Çü{KþáþUÎ×ME s4WME s4WME s4WME s4WME s4WME s4WME s4WME s4WME s4WME
s4WME s4WME s4WME s4WME s4WME s4WME gi?r_¨-□(□ªj_ñäßQüêÝ□□ÌÑ]5□□ÌÑ]5□□
ÌÑ]5□□ÌÑ]5□□ÌÑ]5□□ÌÑ]5□□ÌÑ]5□□ÌÑ]5□□ÌÑ]5□□ÌÑ]5□□ÌÑ]5□□ÌÑ]5□□ÌÑ]5□□ÌÑ]5□
□ÌÑ]5□□Í§ß_-t”Q@□□Q@□°·Ý‹ê□¥e×ME s4WME s4WME s4WME s4WME s4WME s4WME s4
WME s4WME s4WME s4WME s4WME s4WME s4WME s5oNÿ •Ôú-
å[tP□E□P□E□P□E□P□E□P□E□P□E□P□E□P□E□P□Y:·úôÿ wúÖµ□□ÌÑ]5□□ÌÑ]5□□ÌÑ]5□□ÌÑ]5□□Ì
Ñ]5□□ÌÑ]5□□ÌÑ]5□□ÌÑ]5□□ÌÑ]5□□ÌÑ]5□□ÌÑ]5□□ÌÑ]5□□ÌÑ]5□□ÌÑ]5□□ÌÑ]5□□ÌÑ]5□□
‰§□Çê}□ò-°(

+ïøý—
ê?•V®šŠ æh®šŠ æh®šŠ æh®šŠ æh®šŠ æh®šŠ æh®šŠ æh®šŠ æh®šŠ æh®šŠ æh®šŠ æh®šŠ æh®šŠ æh®šŠ æh®šŠ æh®šŠ Žßþ=¢ÿ p□*’Š(®mÿ Ö7Ô×IE s4WME s4WME s4WME s4WME s4WME s4WME s4WME s4WME s4WME s4WME s4WME s4WME s4WME s4WME s4WME s5□Jÿ •—ÿ súŠ×¢€
(¢€
(¢€
(¢€
(¢€
(¢€
(¢€
(¢€
(¢€
(¢€
(¢€
(¢€
(¢€
(¢€
(¢€
(¢€
(¢€
(¢€
(¢€
(¢€
(¢€
(¢€
(¢€
(¢€
(¢€
(¢€
(¢€
(¢€
(¢€
(¢€
(¢€
(¢€
(¢€
(¢€
(¢€
(¢€
(¢€
(¢€
(¢€
(¢€
(¢€
(¢€
(¢€
(¢€
(¢€
(¢€
(¢€
(¢€
(¢€
(¢€
(¢€
(¢€
2=j;•øö—ýÃü«•

›4V>•ÿ -
Mþáþb¶(¢Š(¢Š(¢Š(¢Š(¢Š(¢Š(¢Š(¢Š(¢Š(¢Š(¢Š(¢Š(¢Š(¢Š(¢Š(¢Š©
©□Ç"}Gó
y-´W3[šwüy'Ôÿ : µE□P□E□P□E□P□E□P□E□P□E□P□E□P□E□P□E□P□E□P□E□P□E□P□E□P□E□
P□E□P□E□P□FG-fêÿ v/©þ•—
@□6G-□-µÌÑ@□5□^Çþ<¢ú□Ž±@□□Q@□□Q@□□Q@□□Q@□□Q@□□Q@□□Q@□□Q@□□Q@□□Q@□□Q@□□Q
@□□Q@□□Q@□□Q@□□Q@□□Q@□□Q@□□Q@□□Q@□□Q@□□Q@□□Q@□□Q@□□Q@□□Q@□□Q@□□Q@□□Q@□□
Q@□□Q@□□Q@□□Q@□□Q@□□Q@□□Q@□□Q@□□Q@□G-□ƒ}ÿ -
²ýGò-‹Oøô‹ýÑ@□QE□ QE□ QE□ QE□ QE□ QE□ QE□ QE□ QE□ QE□ QE□ QE□ QE□ QE□ Q
E□ QE□ Q'ëEaZÿ ÈA?ß4□»E□P□E□P□E□P□E□P□E□P□E□P□E□P□E□P□E□P□E□P□E□P□E□P□E
□P□E□P□E□P□E□P□E□P□E□P□E□P□E□P□E□P□E□P□E□P□E□P□E□P□E□P□E□P□E□P□E□P□E□P□
E□P□E□P□E□P□E□P□E□P□E□P□E□P□E□P□E□P□E□P□E□P□E□P□E□P□E□P□E□P□E□P□E□P□E□P
□E□P□E□P□E□P□E□P□E□P□E□P□E□P□E□•â- øŸÃ×□GÛîlRà¨'[s‡(□,¿B2?-ý(□>-
ˆž□°ñl^□µÔVãR"pÛ
—@UK□.8Î□þ]k©¯ ÿ „gJðŸí
á□3G¶òm×Mwlœ´ŽEÆY•rp?/J÷j (¢Š (¢Š (¢Š (¢Š (¢Š (¢Š (¢Š (¢Š (¢Š
(¢Š Žãþ=¥ÿ pÿ *çk¢¸ÿ •i□Ü?Ê¹Ú ½¥□ÇÓ□¸□-ŠÇÒ¿ãé¿Ü?ÌVÅ □QE □QE □QE □QE □
QE □Ìx"â…|#□□Ž¹ª‹[™"□,~D²e 'H□ƒùWO^Uâ□□h¾□øÚ×°íïÙ-
åðêD□å;å¾ÐÇ□PO@h□©Ð>%øGÅ□¢éš6¯ö›ÆBâ?³Jœ

§, ~µÖV□‡¼iáÏ□o□&¯ovè74c*àzí`□-ø-ê Ì×<E£øjÌ]ë:Œ□P"…2¶
E□Xý□gø{ÇÞ□ñ]ÃÛèºÄ□S¨Üb*Ñ¹□ÈW 'î+Jï@Òoõ[mNòÂ
‹Ëd)□²®ï,□"´-□ã¯Zá~"Ú[□ÂqàIl¢EÖŸU□'1‡kU□"□u□õé"êh□Òè¢Š (¢Š (¢Š (¢Š (¢Š
(¢Š (¢Š *¦¥ÿ -Mõ-
Î-ÕMKþ<›ê?• bVæ•ÿ -Iõ?Î°ësNý •$úŸç@□¨¢Š (¢Š (¢Š (¢Š (¢Š (¢Š †îîÞÂÎk»©V□
x□É,Žp□@É'ðª-ñ&'â-4ê:-à°µ□□Œž['Ì1'† ÷□Êx™›Æ¾*‹ÁÖìN•e²ë['O

3˜íóþÑ□□°¤øHªšG^UT*¯^/ `□¹h□Ðk7[ñ□‘áË/¶k□„□P□€Ò¶
E□Iö□¥^4¾#ðœ¿□|A¯ø»Q´Q¥Üÿ fé–Óƒ!‹`□I□`□Ic÷±Æ1@□î•ñ□Â^(¿û

•¬Åqu,Â#□ÆÌ□\□QŸÂ°Šæ<=âÿ □x°ì>•□gywn¤€c)*)à□□□Ç8Èõ®ž€
(¢€
(¢€
(¢€
(¢€
(¢€
(¢€
(¢€3u□»□Ôÿ JË-M_îÅõ?Ò²è ¢Š(□zÇþ<¢ú□Ž±Uì□ãÊ/§õ«□ QE□ QE□ QE□ QECu□¹³ž□šH
□XÙ□±□□21¹Iî:Š Ì¾ñfƒ¦ë-z-Ö§
jwŒ□□a-rOL€

Ð}N+f¼›Ä¾□Òü3¯ø□□:□-]kt÷□±y§m‡æw<±ÿ #□ë4□□®ø³AðÓÛ¦¯©Ãk%Ë„†3-
w$ã…PN3ß□-šò^Þ□Ó<?àãu□-çQ¹ÔíMÅýÛù"ËûÁÁnÊ;(Àã¥zõ □•¯ø—Fð½‡Ûu-B□8
Â—Éf>Š£%□Ð□Õ¯4ø‰á-
2ÏÁÞ+×$□ÞjsÛ9[‹§Þ`BxŽ!Ñ□{r{"@-•oq□Ý´W0>øeA"61•# óíRVg‡?äWÒ□ëÊ-
ý V• □QE □QE □QE □QE □QE □QE □QE □QE □QE □QE □QE □QE □QE □QE □QE □QE □Q
E □QE □QE □"¯x›Eð½¢ÝkZŒ□Q9Ây‡,çý•□'ð□-^káËH<MñcÅ:Æ¡□Ü□□¢°°I□å‡å&F ÿ □ný
y4□ÔxwÇž□ñ\Ï

‹¬Au2□Æ,4o•P¬□#ÜWE^gñrÆ□.ÏJñ…¤I

¥¥j□³LŠ□I□6ÖF=ÁÈý}kÓ(¢Š(¢Š(¢Š(¢Š(¢Š(¢Š(¢Š(¢Š(□□ûþ?eú□å[□Ÿñé□û¢±
ï¿ãö_¨þU±iÿ -‘□º(□j(¢€
(¢€
(¢€
(¢€
(¢€
(¢€)êš¶Ÿ¢X½ö©{□¥ªpe™ÂŒúsÔûV□‰ñ+ÁÞ"ÔFŸ¥ë°MvÇ
□#Æ\ú.ð7¦kkSÐ´-jKWÔìa»6’□a□.åVÆ3·¡?^•Â|i³µ□ Y<□ Ö¿´-
âÒäE□E”¸áHç□□Žœ□A@-™Q\ÜÁgm%ÍÌÑÃ□JZI$`ª u$ž ©kÍþ.”ž/
i—ŽSI¾Ö¡Š÷œ+§$#-
Byü=¨□VÛâÏ□.õ□±‹ÄvÞ{6Ñ½□□Ÿ÷Ê…ýk³¬]kÂúN³á¹ôIì-ÖÍâ1Æ«□□#Ž□□ºGQ•JÃøGªO«|1Ñ
¦°róÄ•nÌNIòÝ•□ã P□mE□P□E□P□E□P□E□P□E□P□E□P□E□P□XV¿ò□O÷ÍnÖ□¯ü„□ýó@□´QE □
QE □QE □QE □QE □QE □™®x‹GðÕ˜»Öu□,¡'
el□>Š:±ú
Ó¬ë½□I¿Õmµ;Ë□./-•¤□Ê»¼°NNÐx□Ž½h□7ÃÞ>ð·Šî-ßEÖ °•Fã□V•ÈîB¸□•q]□y§Ä[KoøN<
-”H°Óê£2F0íj£2ƒŽ£-½2}Mz] RÕu};C°{íRö
;TàË3…□ô-§ØV□‹ñ3Á¾!ÔWOÓ5Ø&»s„‰Ñã.}□z□ÇØV®¿§è□0A}â□¬ÚÞÁüä’ñ€Š&?.ã¸íïÆkÏ>
Þèž5M/Fð¼¶Ú-¶—°Ë□ÍŽ$[(Ôå□¤^□c¶y8ãŠ õª(¢€
(¢€
(¢€
(¢€
(¢€
(¢€
(¢€
(¢€
(¢€
(¢€
(¢€
(¢€
(¢€
(¢€
(¢€
(¢€
(¢€
(¢€
(¢€
(¢€
(¢€
(¢€
(¢€
(¢€
(¢€
(¢€
(¢€
(¢€
(¢€
(¢€
(¢€
(¢€
(¢€
(¢€
(¢€
(¢€
(¢€
(¢€
(¢€
(¢€
(¢€
(¢€

(¢€
(¢€
(¢€
(¢€
(¢€
(¬□□h?ð’øzçIû}Í□Ÿ·÷öÍ‡\0?®1@-qâOù9Ï ÿ Ø-
ÿ •ÅzýxÓþÏÖÒ]%Óø·Xk„□RS,ê9à□äu?□z?„<5ÿ □ž„°gö□æ£‰□O:é·0Ïaè?ÄÐ□õ□Q@□□Q@□
□Q@□□Q@□□Q@□□Q@□□Q@□□Q@□□Q@□□Q@□Ü□Ç´¿îå\ítW-
ñí/û‡ùW;@□´¯øúo÷□ó□±XúWü}7û‡ùŠØ □Š(□Š(□Š(□Š(□Š(□•¢-
ÜT□Æ3ŽikŒÖ>□iÚ®±sªÁ¬ëÚUÕÉ□fÓoÚ ä ‘,:□Ò€0ü}ceiñ□Á□zdQC®M©„•Â¡^K\-
4¶:€;ŸS^Ÿ\¿‡<□¢øjúMF□w{©È»-ÿ PœÍ1_@O } «z¿…,5¯□hÚÕÌ×Is¤³¼ □□Q‹

áŽzv#ñ |Iâ]?ÂúY½¾gbÌ#,Þ!º[‰□ÝD^ìk□Â~□Ô%Õ§ño‰UFµu-
•□ª•Éa□Q□žìz³zð=ßâ¯‡v>,Ö¬µiõ}fÂîÎ#□-§Ü¬[rNHÊ’   Î   □p□P
0Aÿ „óÇ□□Ž¯ÿ ØP□□E□P□E□P□E□P□E□P□E□P□E□P□E□P□U5/øòo¨þunªj_ñäßQüè□□·4ïøò
O©þu‡[šwüy'Ôÿ : µE□P□E□P□E□P□E□P□E□P□TõY/aÑïeÓ`YïÒ□kh□,‡□)Ú¤œ
          Å\¢€<‹Âsøÿ ÂúCZÿ Â¸kËÉå{‹ËÇ×mÕ®&c’Ä`ã°□<□MøC«x…çÔí$ðÇ—
§Í¬]És}öøÏÙ¥<˜ü¼eð@□‡□çµzýdx□Ã-~□·½†Éçu»¼–öO9•!ä9 `

8ãùÐ□½y‡Ã›□>ßÆÞ:¶¹´·:´z»Ü¬□□ó<‰~eÁ<íëÓÖ»-;Â–□gŠµ_□C5Ó^jk□L’H

j 6ŒdtîO¶*‰>-èþ$ÔâÕZ{ý;T•<±}¦Ü%+èN?-
h•ñ½½¬¼.Ÿi¬Iu/œb ;Ûù‹ã¨ÆqŸzôÚæ<5à=Ã"_À×w°œË²KýBs4ì¿ÝÜz ®ž€
(¢€
(¢€
(¢€
(¢€
(¢€
(¢€
(¢€3u›Ôÿ JË-M_îÅõ?Ò²è ¢Š(zÇþ<¢úŽ±UìåÊ/§õ« QE QE QE QEEuÚm&·óe
‹ÍFO2&Úé'Œ©ìGc@Ä_ù|ÿ a•ý•× ×›Üü³¼–Þ[¯xÆy-ßÌåÔÃ›ûÊJp}Åuþðÿ ü#-
Úÿ ljÚ§™)"ÍÕ.|ù€6ƒ…ã8õ&€9ŒŸò$Aÿ a+_ý+ÐkÎµ/„z°d¾ñç…¤óD2jA'X,¡Æ
;zWIáŸ
Â4×'û]Õ|ð£-çž#Æ~çÏ?A@r?¿ä˜x‡þ¼ÛùŠÑðï…,<3>«5"×R6§v×syò
íÔ. ÀúäûÖ

»ð¾ÛÄ77²]ø¯ÅIovÄÉg□¢□¸□øB□#oµ u-□ÿ ‛_Iÿ ¯(□ô□ZuÊxcÀ±ø^óÏ‹Äž"Ô#□ù+m¨_y°
ã□WhÁ□Àö&°° (¢Š (¢Š (¢Š (¢Š (¢Š (¢Š (¢Š (¢Š (¢Š (¢Š (¢Š (¢Š (¢Š (¢Š (¢Š
(¢Š (¢Š (¢Š (¢Š +Î<□*éß□|s£NBO=ÄWð)ÿ –
‛°üÄzà•□Ö½-¹Ÿ□x□Gñ]Í½åÑ°´Ôm†Øo¬f0Î‹é¸väõ□Ï©

□ã□‰yáÍ;Ã±□×šÆ¥□□D:•

□›–ƒ□'ÜW£W%áÿ ‡z>□ª□XÜj□¦©³bÞêw&yQ{…8 ~Yæ°Ú (¢Š (¢Š (¢Š (¢Š (¢Š (¢Š (¢Š
(¢Š Á¾ÿ •Ù~£ùVÅ§üzEþè¬{ïøý—ê?•lZ□Ç¤_îŠ šŠ(□Š(□Š(□Š(□Š(□Š(
:Î³a iSêz□Ê[Ú@¹woÐ□Üž€

µÈx□ÌÔ<Sâ□|aâ□gµŠ□#GÓ$ûÐ+ušAÿ =□vþ□ïÓWÆ^□±ñªX-
î¡©Y›□LÐµŒË□Þq†9SÈÇ□`ŒšÇÿ …Yÿ SïŽ□ðqÿ ØP□ Vf½ iž&Ò%ÒõkU¸´—
□©$□GB□ä□ëTµ¿□ÙøƒÁÿ ð□_Þ_½±Ž(ÚàL<÷ØA

ÌA□’¼œs‟Rk~□±×t;}*æ{È£¶dhg¶¸1ÊŒªT0aß□õõ

'[ø{áÏ

è7Wú×^üIu¤ÚÆXØÝj□ä¶: P□9à□žõÐü+ÑæÐ¾□èÖw1˜çhÚwB0TÈÅÀ#±□€ü*½□Â½□
ø/5+ýc]−
Ý·Â5{Ó:FÞ¡p□üs]Í □QE □QE □QE □QE □QE □QE □QE □…kÿ !□ÿ |ÖíaZÿ ÈA?ß4□»E□P
□E□P□E□P□E□P□E□P□E□P□Xþ$ñ.Ÿá},Þß3±f□Áo□Ý-Ä‡î¢/v5±\□$¾□Øø³Z²Õ§Õõ›
»8Œp¶Ÿr±mÉ9#*H'8$□À□ Ï øwP—VŸÅ¾%U□ÕÔ~T□ªw%„□Dj{±êÍëÀ÷ìë€
0Aÿ „óÇ□□Ž¯ÿ ØWEâ□ Øx²
□oæ°□l®Òî3o B]s€r□□û-
z Ñ¾M6õ□ᵝ5□µ□nTŸ²Ü□a*©□ù□Þ ‘ÛŽ+Î~$øCÃš□„¯<E¤ZÛèz®ž¢[[«□□□ù !
€Á³Œ□Þ»/□ø3Fñ|6ëªE(šÕ‹[ÜA)ŽXIÆJ°ú

¹éX–Ÿ
4D¾,ëSÔµ½sìî-□µkã<q°èBà□øæ€:í□ââóCÓînÓË¹šÚ9%Lck•□‡æM]¢Š (¢Š (¢Š (¢Š (¢Š
 (¢Š (¢Š (¢Š (¢Š (¢Š (¢Š (¢Š (¢Š (¢Š (¢Š (¢Š (¢Š (¢Š (¢Š (¢Š (¢Š (¢Š (¢
Š (¢Š (¢Š (¢Š (¢Š (¢Š (¢Š (¢Š (¢Š (¢Š (¢Š (¢Š (¢Š (¢Š (¢Š (¢Š (¢Š (¢Š (
¢Š (¢Š (¢Š (¢Š (¢Š (¢Š (¢Š (¢Š (¢Š (¢Š (¢Š (¢Š (¢Š (¢Š (¢Š (¢Š (¢Š (¢Š
l©æDéœnR3YŸÙ/ÿ =Wò-Z(□•¥‹[L\¸l®0□]¢Š (¢Š (¢Š (¢Š (¢Š (¢Š (¢Š (¢Š (¢Š (¢
Š (¢Š (¢Š (¢Š (¢Š (¢Š (¢Š *□¨MÄ□0@$ŽMME e□d¿üõ_Ê¯ÚÂmàXÉ□Œò*j(¢Š(¢Š(¢
Š(¢Š(¢Š(¢Š(¢Š(¢Š(¢Š(¢Š(¢Š(¢Š(¢Š(¢Š(¢Š(¢Š(□-í¡°□□□Ûž¢ª□d¿üõ
_Êµh

¯ì—
ÿ ž«ùQý’ÿ óÕ□*Õ¢€#·ˆÃ□FNJŽµ%□P□E□P□E□P□E□P□E□P□E□P□E□P□E□P□E□P□E□P□E□P□E□P□E□P□E□P□E□P□E□P□E□P□E□P□E□P□E□P□E□P□E□P□E□P□E□P□E□P□E□P□E□P□E□P□E□P□E□P□E□P□E□P□E□P□E□P□E□P□E□P□E□P□E□P□E□P□E□P□}ÆœÓNò
□Û□v□ü¨R2s´c4ú(¢Š(¢Š(¢Š(¢Š(¢Š(¢Š(¢Š(¢Š(¢Š(¢Š(¢Š(¢Š(¢Š(¢Š(¢Š(¢Š(¬ø´æŽäJd□□œb´(□Š(□¢¸˜[Âd+•;TµSRÿ •&ú□ç@□Ã(š□□

□íRU{-
øò‹éýjÅ □QE □QE □QE □QE □QE □QE □QE □QE □QE □QE □QE □QE □QE □QE □QE □QE
C=À•ãR¤ï8úTÕGPÿ]kþÿ øUê (¢Š (¢Š (¢Š (¢Š (¢Š (¢Š (¢Š (¢Š (¢Š (¢Š (¢Š (
¢Š (¢Š (¢Š (¢Š (¢Š (¢Š (¢Š (¢Š (¢Š (¢Š (¢Š (¢Š (¢Š (¢Š (¢Š (¢Š (¢Š (¢Š
(¢Š (¢Š (¢Š (¢Š (¢Š (¢Š (¢Š (¢Š (¢Š (¢Š ¬.Á¼6û

G|ûf¬Öb□Èi¿-
åZt QE□ QE□ QE□ QE□ QE□ QE□ QE□ QE□ QE□ QE□ QE□ QE□ QE□ QE□ QE□ QE□ QE□
 QE□ QE□ QE□ QE□ QE□ QE□ QE□ QE□ QE□ QE□ QE□ QE□ QE□ QE□ QE□ QE□ QE□ QE
□ QE□ QE□ QE□ QE□ QE□ QE□ QE□ QE□ QE□ QE□ QE□ QE□ QE□ QE□ QE□ QE□ QE□ Q
E□ QE□ QE□ QE□ QE□ QE□ QE□ QE□ QE□ QE□ QE□ QE□ QE□ QE□ QE□ QE□ QE□ QE□
QE□ QE□ W;ã_□Yø'@þÔ»,k,ò¬□A□ËI#□@Ïa…<ûwé]□5ãI6ïEm§rî□Áõ-ô□ã"|dñ6•#¿×ü□{
e¤HÀ□·6èÁõÜ¯gÐ□¹¯]°¾·Ôôëkû9□-×1,±8□Ü¬2□ë^yñÃZ·²ð□úF<ÛýYÒ
h□nfÃ«□•l□õ"°ï□i3h^
Ñô»ŸõöÖ¨'Œç□Œ°Ï|□E nÑE□ QE□ QE□ QE□ QE□ QE□ QE□ QE□ QE□ QE□ QE□ QE□ QE□
 QE□ QE□ QE□ QE□ QE□□^úúÛL°¸¾¼™a¶·ŒÉ,•ÑT

‟\□□□|A□ký§¤|>Ôï4r7%ËÝG□’'÷-□
□{c-Kñ•Ù¼□¶A^Këûki0qò—□ÿ è5è□Æ□Ä‘F□#E
ª£ □Ð
ÉðÇ‰´ÿ □èpêÚk?’ä«$ƒ□□Ž¨Ã±□±^uðõ□ŸŽ¾ i±□¶ñê□ÜªŽ□¥BÍü…z-
□QE □QE □QE □QE □QE □QE □QE □SRÿ •&ú•çVê¦¥ÿ -MõÎ€-
cÿ -Q}?-Xªö?ñå□ÓúÕŠ (¢Š (¢Š (¢Š (¢Š (¢Š (¢Š â<]ñ/OðÜw±XÚM¬_ÙGæ\Álp-
ëë,˜!=‡'Ú°í:ïûCL´½ ³í□¤»3□»”□gñ®OâM□-ŸÃ□□}šÞ(|ëy%‟Ë@»Ü•K□u'Ö°/

□È¯¤ÿ ×”?ú □:àu□□ø‹þ□½_DÐ<□uq¥˜Dó□R;~d@ãåuúŽ
é]õy„‡Æ>□øƒâ•GKðsk□Z£Z´s□F+|□á
xl“É>□(□«ðÖ³âmRæt×|%ý‰□ 1Éý£□Ï˜Ùé„□Zé+□ðÇ□b×5‰´=KJ»Ñu¸£óM□Ö□‘:nG□0ÿ =Ž;
(¢Š (¢Š (¢Š (¢Š (¢Š (¢Š (¢Š £¨□®µÿ □ü*õQÔ?×Zÿ ¿þ□z€
(¢€
(¢€
(¢€
(¢€
(¢€
(¢€
óÿ □üVÓ´‰6iv3k
□Ê[Ý\BÛ-àf`6™0C??usîEz□y¿Å›;]?áý--□¼Vöé©Z„Š□□«ûÁÐ

(□Ò(¢Š à§ø•¬Þ\Ü/†¼□¨jÖ–
ò4ow,éjŽTàù{ùq□kkÁþ1³ñ……Ä°ÛÜYÝÚJ`»³¹\I□ƒ±öô>Æ°□$H£i$uD@Y™Ž □©&¼÷á¢¶©-x³
Å'¡K□bõ□Ï#-dp©O0□F$þT□è"QE □QE □QE □QE □QE □QE □QE □QE □QE □QE □QE □QE
□QE □QE □QE □QE □QE □QE □QE □QE □ÉxŸÇ
¡ê¶ú&›¥\ë:Üñù«elÁvGœo'Ï□3Åuµç^□□óâ□□µ)FéÅìVŠÇª¤i€□ éùP□½+â□Ïü
$□°‰ü7u ^^äZ;Î³Ã3□á□. ooñ□î«Î¾5
‹À±êJ1q§j□×0·u`áxÿ ¾«Ñh ¢Š(¢Š(¢Š(¢Š(¢Š(¢Š(¢Š(¢Š(□1?ä4ß•ò-:ÌOù□7ã
ü«N€
(¢€
(¢€
(¢€
(¢€
(¢€
(¢€(êÚÆ• éÒj□¥ÜV¶±ýé$8ç°□©'°□šæü3ãïøI|S}£□□îÆ;{T°Š[£µåF8□ËÆT-£'8ì+«¹³µ¼
ò¾Õo□ÐL,X¼Ä□±Æ@až„dóï\.—
ÿ %ß^ÿ °=¿þ‡@-ƒX>3ñ/ü">□¼ÖþÆo□¹Œ□□ž^òî¨>l□}ìô-êä>'é□†»ð÷QÓô«cs{#@ÑÂ□WvÙ
'•,@è□ g□Â[ñ□þ‰‡þWíÿ Â»Ëw'Khžh¼™Y□x÷nØØäduÇ-yíÏÄ_□hÑ□Ï□|=¾³Ó"™n-
o¢°1¯v* `

ü×y§j6š¶›m¨XÌ³ZÜÆ$ŠEèÊz□ı¨□Õ□Q@□□Q@□□Q@□□Q@□□Q@□□Q@□□Q@□□Q@□□Q@□□Q@□□Q@
□□Q@□□Q@□□Q@□□Q@□Ì¨¥™,ªŒ'N □ç‹ñ+UÕÚiü)àËÝkL‰Ê}¹®'Ù%
à˜Ã□\f¶>'^IcðÏÄ3ÄÅ_ìm□#¨□…?£V§„ì¢Ó¼-
£ZB¡cŠÊ□ □̧2□□Í Uð□Œ,¼_c<°A=¥Ý¤¦□»+•Û,□z□éè}□¡®Š¼ëJAeñó],□µ/´hn¥□¡tp€ýq
^‹@□□Q@□□Q@□□Q@□□Q@□□Q@□□Q@□□Q@□□Q@□□Q@□□Q@□□Q@□□Q@□□Q@□□Q@□□Q@□□Q@□□Q@
□□Q@□□Q@□□Q@□□Q@□□Q@□□Q@□□Q@□□Q@□□Q@□□Q@□□Q@□□Q@□□Q@□□Q@□□Q@□□Q@□□Q@□□Q
@□□Q@□□Q@□□Q@□□Q@□□Q@□□Q@□□Q@□□Q@□□Q@□□Q@□□Q@□s~8ñ¦Ÿào□>©|-#³yvð'Yd
•3Øq'{{œ□ÒVnµáý#Äv©k¬ið^Â•½RUÎÖÆ2?: ñ-
□kž□Ô¼@|qã•□iï¬7üyXî;,Ó·-¾ƒ¶rrÇ•rÒu•;]°[í.ò+»W%D±6FG□W=ÿ
³Àßô,Ø□ß'ük¡Òt};B°[□.Î+KT%„Q

žI
ÔQE □QE □QE □QE □QE □QE □QE □QE □QE □QE □QE □QE □QE □QE □QE □QE □QE □QE
q□□tkÍkÀ□©§Fd¾µxîàE□,Ñ°b îvîÀõ¤°ø¯àË½□59µÛ;Såî-
ÖY š6Ç+³ï□□□□žÕÚÖT¾□Ð&¾ût°->%Ù;¼÷´ŒÉŸ]ØÎh□”ø_muvš÷Š®íä¶:õñžÞ)□□[¨Û□#ÔŒþ
‡½z□□P□E□P□E□P□E□P□E□P□E□P□E□P□E□P□U5/øòo¨þunªj_ñäßQüè□ö?ñå□ÓúÕŠ¯cÿ -Q}
?-X □Š(□Š(□Š(□Š(□Š(□Š(□<ø£âm ø□ÄZ`×4Ó¨}□£û/Úãówd|»3œûb·¼-
â□□RÑtÛ+□cO°»ŠÊ#$□\£È€*ƒ•□#□â-]x;Â÷·R\ÝøoGžâV-
$²ØÄÎçÔ’¹&§Ó¼3 è÷&çLÑ4Û□Ê”2ÛZG□□<ã*□Ç□•j -<G¤ßë·Ú%µâÉ¨Ø*µÌ□X□

29#□¨èN+9>!ø=§¸…¼I¦Ã-¼□□©q8…•”□F□äv-È´ë(/g½†ÎÞ;»€□Ó¤J$•

□æ□'□³Tï|1áýJs=þ…¦]LNL‟ÚFì□□(□„¶Ôm|oñ□IÔô&7□nƒk:Üß¢‘□’J»V5oâÆwqÅz…Emkog
n°ZÁ□□§
□H□Wè□□- □QE □QE □QE □QE □QE □QE □QE QÔ?×Zÿ ¿þ□z¨êë-□ßÿ
½@□□Q@□□Q@□□Q@□□Q@□□Q@□□Q@□y?Å¯□øzûÂQÚÚkÚ]ÅÂj6ìÑEy□°…□n$□ž;úW¬V□ø-
Â.ìïám□™ŽK□>"Iÿ ¾h□þ™¯húÑ”iZµ…ù‹-gÙnR]™Î3´œg□ò¨ô□□é:ô·Ñé—‹pö□›{□□‡—
ê9□?Q‘Riš

•¢™N•¤ØX□qæ□[d‹~3Œí□8Éüê{M:ÊǺ§k;;{v¸□Ë1†%C#ž¬Ø□Ÿs@-W¬xËFñÞ->ˆÞ"Óô¿

ZÉ²öY¯R□µ□□bŒ□□‹Õ¿‹ ã5è°&¹á»ÕM;BÕt»‘o□Ûoes□ùqŒ□ò©8□
~U□x□Â.Å›Â°!brIÓâÉ?÷Í[Ó|7¡h×□q¥è°uŒÌ»□K[T‰Šä□□ □d

=¨□RŠ(□Š(□Š(□Š(□Š(□Š(□Š(□Š(□Š(□Š(□Š(□Š(□Š(□Š(□Š(□Š(□Š(
□Š(□Š(□Š(□¼®ÏW³ð□ÅO□Ûë‟-ž›¯yW-Wrñ□˜«¶Dfè

Nyì□¨¯Tª×Ú}-
§nmïìííà'&9âY□?B1@-eã}{Nñåæ"àï□]Å©4÷'\j□[7™□□ñ•Ç.8É8ÀÏoq^-Tôý'MÒ"h´Ý>ÒÊ
69)m
Æ ú(□r€
(¢€
(¢€
(¢€
(¢€
(¢€
(¢€
(¢€
(¢€3□þCMøÿ *Ó¬Äÿ •Ó~?Ê´è ¢Š(¢Š(¢Š(¢Š(¢Š(¢Š(□¦¡ªiúE¯Úu+ëk+}Á|Û™–
4Éè2Ä

×˜éÞ,ðâ|hÖ¯ßÄ□RÙÉ¥A□\□ÈÄlÁ¹PÙÁ#Ò½;PÒôý^×ìÚ••µí¾àÞUÌ+"dt8`Fk+þ□O□ÿ Ð©¡ÿ
à°þ&€-ê~%Ñ´}□ë—°„I¥íF□)™□ƒ□□®Ðr

GJf«â-□C±´½Ôõ□-mo□$□I•¬J-□ãŽ□98«’é□lú`Ó%Óí$ÓÕU□«Â¦
«□£f1□□Ž8ÅIqciwkö[›X&·À-T‘†L

œ-(□•ñ□Ä¿□Ùè—&□bÇR¸–&Ž□;9Vw™Ø`&ÕÏRqÍ]øk¢Ýø{áÞ•¦_©[¨¡-"-¨]™öŸq»-
…kXøkAÒî>Ñ§èšm¤ßóÒÞÑ#oÌ kR€
(¢€
(¢€
(¢€
(¢€
(¢€
(¢€
(¢€
(¢€
(¢€
(¢€
(¢€
(¢€
(¢€
(¢€
(¢€0üe£Iâ□k□L8ó®m]"Éã~2¿¨□Ëx+âO‡[ÂVVúÆ«k¦jv□-µå-ì,)□HÆÓ€Ø'8Ï□ëŽµèµ™{á½
R°□Wú.□up:K=ª;þdf€8¯□JÞ(ñÏ^<k□R&™,Qéút')S4hrî□çilcüEzE5□cEDP¨£
ª0 ô□ê (¢Š (¢Š (¢Š (¢Š (¢Š (¢Š (¢Š (¢Š (¢Š (¢Š (¢Š (¢Š (¢Š (¢Š (¢Š (¢Š
(¢Š (¢Š (¢Š (¢Š (¢Š (¢Š (¢Š (¢Š (¢Š (¢Š (¢Š (¢Š (¢Š (¢Š (¢Š (¢Š (¢Š (¢Š
(¢Š (¢Š Fe_¼À}M7Í•þz/ç^□ûM□Î-ÿ o□ûF¼□€>üócÿ ž‹ùÑæÇÿ =□ó¯€è □¿<Øÿ ç¢þty
±ÿ ÏEüëà:(□ïÏ6?ùè¿•-l□ôÑ□:ø

Š ûóÍ□þz/çG›-
üô_Î¾□¢€>üócÿ ž‹ùÑæÇÿ =□ó¯€è □¿<Øÿ ç¢þty±ÿ ÏEüëà:(□ïÏ6?ùè¿•-l□ốÑ□:ø

Š ûóÍ□þz/çG›-
üô_Î¾□¢€>üócÿ ž‹ùÑæÇÿ =□ó¯€è □¿<Øÿ ç¢þty±ÿ ÏEüëà:(□ïÏ6?ùè¿•-l□óÑ□ø

Š ûóÍ□þz/çG›-
üô_Î¾□¢€>üócÿ ž‹ùÑæÇÿ =□ó¯€è □¿<Øÿ ç¢þty±ÿ ÏEüëà:(□ïÏ6?ùè¿•-l□óÑ□ø

Š ûóÍ□þz/çG›-
üô_Î¾□¢€>üócÿ ž‹ùÑæÇÿ =□ó¯€è □¿<Øÿ ç¢þty±ÿ ÏEüëà:(□ïÏ6?ùè¿•-l□óÑ□:ø

Š ûóÍ□þz/çG›-
üô_Î¾□¢€>üócÿ ž‹ùÑæÇÿ =□ó¯€è □¿<Øÿ ç¢þty±ÿ ÏEüëà:(□ïÏ6?ùè¿•-l□óÑ□:ø

Š ûóÍ□þz/çG›-
üô_Î¾□¢€>üócÿ ž‹ùÑæÇÿ =□ó¯€è □¿<Øÿ ç¢þty±ÿ ÏEüëà:(□ïÏ6?ùè¿•-l□óÑ□:ø

Š ûóÍ□þz/çG›-
üô_Î¾□¢€>üócÿ ž‹ùÑæÇÿ =□ó¯€è □¿<Øÿ ç¢þty±ÿ ÏEüëà:(□ïÏ6?ùè¿•-l□óÑ□:ø

Š ûóÍ□þz/çG›-
üô_Î¾□¢€>üócÿ ž‹ùÑæÇÿ =□ó¯€è □¿<Øÿ ç¢þty±ÿ ÏEüëà:(□ïÏ6?ùè¿•-l□óÑ□:ø

Š ûóÍ□þz/çG›-
üô_Î¾□¢€>üócÿ ž‹ùÑæÇÿ =□ó¯€è □¿<Øÿ ç¢þty±ÿ ÏEüëà:(□ïÏ6?ùè¿•Er±ÜBcóUsß9¯
,(□¾ òá…cóTíïš"Í•þz/ç_□Ñ@~y±ÿ ÏEüèócÿ ž‹ù×ÀtP□ßžl□ó̃Ñ□:<Øÿ ç¢þuð□□□÷ç›-
üô_Î•6?ùè¿•|□E }ùæÇÿ =□ó£Í•þz/ç_□Ñ@-
~y±ÿ ÏEüèócÿ ž‹ù×ÀtP□ßžl□ó̃Ñ□:<Øÿ ç¢þuð□□□÷ç›-
üô_Î•6?ùè¿•|□E }ùæÇÿ =□ó£Í•þz/ç_□Ñ@-
~y±ÿ ÏEüèócÿ ž‹ù×ÀtP□ßžl□ó̃Ñ□:<Øÿ ç¢þuð□□□÷ç›-
üô_Î•6?ùè¿•|□E }ùæÇÿ =□ó£Í•þz/ç_□Ñ@-
~y±ÿ ÏEüèócÿ ž‹ù×ÀtP□ßžl□ó̃Ñ□:<Øÿ ç¢þuð□□□÷ç›-
üô_Î•6?ùè¿•|□E }ùæÇÿ =□ó£Í•þz/ç_□Ñ@zÜG□ï□yÊ¾[g×5?›-
üô_Î¾□¢€>üócÿ ž‹ùÑæÇÿ =□ó¯€è □¿<Øÿ ç¢þty±ÿ ÏEüëà:(□ïÏ6?ùè¿•-l□ó̃Ñ□:ø

Š ûóÍ□þz/çG›-
üô_Î¾□¢€>üócÿ ž‹ùÑæÇÿ =□ó¯€è □¿<Øÿ ç¢þty±ÿ ÏEüëà:(□ïÏ6?ùè¿•-l□óÑ□:ø

Š ûóÍ□þz/çG›-
üô_Î¾□¢€>üócÿ ž‹ùÑæÇÿ =□ó¯€è □¿<Øÿ ç¢þty±ÿ ÏEüëà:(□ïÏ6?ùè¿•-l□óÑ□ø

Š ûóÍ□þz/çG›-
üô_Î¾□¢€>üócÿ ž‹ùÑæÇÿ =□ó¯€è □¿<Øÿ ç¢þty±ÿ ÏEüëà:(□ïÏ6?ùè¿•-l□óÑ□:ø

Š ûóÍ□þz/çG›-
üô_Î¾□¢€>üócÿ ž‹ùÑæÇÿ =□ó¯€è □¿<Øÿ ç¢þty±ÿ ÏEüëà:(□ïÏ6?ùè¿•-l□óÑ□:ø

Š ûóÍ□þz/çG›-
üô_Î¾□¢€>üócÿ ž‹ùÑæÇÿ =□ó¯€è □¿<Øÿ ç¢þty±ÿ ÏEüëà:(□ïÏ6?ùè¿•-l□óÑ□:ø

Š ûóÍ□þz/çG›-
üô_Î¾□¢€>üócÿ ž‹ùÑæÇÿ =□ó¯€è □¿<Øÿ ç¢þty±ÿ ÏEüëà:(□ïÏ6?ùè¿•-l□óÑ□:ø

Š ûóÍ□þz/çG›-
üô_Î¾□¢€>üócÿ ž‹ùÑæÇÿ =□ó¯€è □¿<Øÿ ç¢þty±ÿ ÏEüëà:(□ïÏ6?ùè¿•-l□óÑ□:ø

Š ûóÍ□þz/çG›-
üô_Î¾□¢€>üócÿ ž‹ùÑæÇÿ =□ó¯€è □¿<Øÿ ç¢þty±ÿ ÏEüëà:(□ïÏ6?ùè¿•-l□óÑ□ø

Š ûóÍ□þz/çG›-
üô_Î¾□¢€>üócÿ ž‹ùÑæÇÿ =□ó¯€è □¿<Øÿ ç¢þty±ÿ ÏEüëà:(□ïÏ6?ùè¿•-l□óÑ□ø

Š ûÌA□½7-zóü?†=jÏ›-
üô_Î¾□¢€>üócÿ ž‹ùÑæÇÿ =□ó¯€è □¿<Øÿ ç¢þty±ÿ ÏEüëà:(□ïÏ6?ùè¿•-l□óÑ□:ø

Š ûóÍ□þz/çG›-
üô_Î¾□¢€>üócÿ ž‹ùÑæÇÿ =□ó¯€è □¿<Øÿ ç¢þty±ÿ ÏEüëà:(□ïÏ6?ùè¿•-l□óÑ□ø

Š ûóÍ□þz/çG›-
üô_Î¾□¢€>üócÿ ž‹ùÑæÇÿ =□ó¯€è □¿<Øÿ ç¢þty±ÿ ÏEüëà:(□ïÏ6?ùè¿•-l□óÑ□:ø

Š ûóÍ□þz/çG›-
üô_Î¾□¢€>üócÿ ž‹ùÑæÇÿ =□ó¯€è □¿<Øÿ ç¢þty±ÿ ÏEüëà:(□ïÏ6?ùè¿•-l□óÑ□ø

Š ûóÍ□þz/çG›-
üô_Î¾□¢€>üócÿ ž‹ùÑæÇÿ =□ó¯€è □¿<Øÿ ç¢þty±ÿ ÏEüëà:(□ïÏ6?ùè¿•-l□óÑ□ø

Š ûóÍ□þz/çG›-
üô_Î¾□¢€>üócÿ ž‹ùÑæÇÿ =□ó¯€è □¿<Øÿ ç¢þty±ÿ ÏEüëà:(□ïÏ6?ùè¿•-l□óÑ□:ø

Š ûóÍ□þz/çG›-
üô_Î¾□¢€>üócÿ ž‹ùÑæÇÿ =□ó¯€è □¿<Øÿ ç¢þty±ÿ ÏEüëà:(□ïÏ6?ùè¿•-l□óÑ□ø

Š ûóÍ□þz/çG›-
üô_Î¾□¢€>üócÿ ž‹ùÑæÇÿ =□ó¯€è □¿<Øÿ ç¢þty±ÿ ÏEüëà:(□ïÏ6?ùè¿•-l□óÑ□:ø

Š ûóÍ□þz/çG›-
üô_Î¾□¢€>üócÿ ž‹ùÑæÇÿ =□ó¯€è □¿<Øÿ ç¢þtðA□□"¾ ¯´¾□ÿ É1ðïýy¥ u´QE □QE □Q
E □QE □QE □QE □QE □QE □QE □QE □QE □QE □QE □QE □QE □QE □QE □QE □QE □QE □
QE □QE □QE □QE □QE □QE □QE □QE p?□>□/Ä_ì½Ú±ÓþÁæô·ówïÙþÒã□=ú×□ÿ

Íý
•ÿ ,ÿ þÙ^ûE x□ü34□ô67þ
ÿ ûeðÌÑÿ ÐØßø/ÿ í•ï´P□•□Ã3Gÿ Cc□â¿ÿ ¶Qÿ

Íý
•ÿ ,ÿ þÙ^ûE x□ü34□ô67þ
ÿ ûeðÌÑÿ ÐØßø/ÿ í•ï´P□•□Ã3Gÿ Cc□â¿ÿ ¶Qÿ

Íý
•ÿ ,ÿ þÙ^ûE x□ü34□ô67þ
ÿ ûeðÌÑÿ ÐØßø/ÿ í•ï´P□•□Ã3Gÿ Cc□â¿ÿ ¶Qÿ

Íý
•ÿ ,ÿ þÙ^ûE x□ü34□ô67þ
ÿ ûeðÌÑÿ ÐØßø/ÿ í•ï´P□•□Ã3Gÿ Cc□â¿ÿ ¶Qÿ

Íý
•ÿ ,ÿ þÙ^ûE x□ü34□ô67þ
ÿ ûeðÌÑÿ ÐØßø/ÿ í•ï´P□•□Ã3Gÿ Cc□à¿ÿ ¶Qÿ

Íý
•ÿ ,ÿ þÙ^ûE x□ü34□ô67þ
ÿ ûeðÌÑÿ ÐØßø/ÿ í•ï´P□•□Ã3Gÿ Cc□â¿ÿ ¶Qÿ

Íý
•ÿ ,ÿ þÙ^ûE x□ü34□ô67þ
ÿ ûeðÌÑÿ ÐØßø/ÿ í•ï´P□•□Ã3Gÿ Cc□à¿ÿ ¶Qÿ

Íý
•ÿ ,ÿ þÙ^ûE x□ü34□ô67þ
ÿ ûeðÌÑÿ ÐØßø/ÿ í•ï´P□•□Ã3Gÿ Cc□à¿ÿ ¶Qÿ

Íý
•ÿ ,ÿ þÙ^ûE x□ü34□ô67þ
ÿ ûeðÌÑÿ ÐØßø/ÿ í•ï´P□•□Ã3Gÿ Cc□à¿ÿ ¶Qÿ

Íý
•ÿ ,ÿ þÙ^ûE x□ü34□ô67þ
ÿ ûeðÌÑÿ ÐØßø/ÿ í•ï´P□•□Ã3Gÿ Cc□â¿ÿ ¶Qÿ

Íý
•ÿ ,ÿ þÙ^ûE x□ü34□ô67þ
ÿ ûeðÌÑÿ ÐØßø/ÿ í•ï´P□•□Ã3Gÿ Cc□â¿ÿ ¶Qÿ

Íý
•ÿ ,ÿ þÙ^ûE x□ü34□ô67þ
ÿ ûeðÌÑÿ ÐØßø/ÿ í•ï´P□•□Ã3Gÿ Cc□â¿ÿ ¶Qÿ

Íý
•ÿ ,ÿ þÙ^ûE x□ü34□ô67þ
ÿ ûeðÌÑÿ ÐØßø/ÿ í•ï´P□•□Ã3Gÿ Cc□â¿ÿ ¶Qÿ

Íý
•ÿ ,ÿ þÙ^ûE x□ü34□ô67þ
ÿ ûeðÌÑÿ ÐØßø/ÿ í•ï´P□•□Ã3Gÿ Cc□â¿ÿ ¶Qÿ

Íý
•ÿ ,ÿ þÙ^ûE x□ü34□ô67þ
ÿ ûeðÌÑÿ ÐØßø/ÿ í•ï´P□•□Ã3Gÿ Cc□à¿ÿ ¶Qÿ

Íý
•ÿ ,ÿ þÙ^ûE x□ü34□ô67þ
ÿ ûeðÌÑÿ ÐØßø/ÿ í•ï´P□•□Ã3Gÿ Cc□à¿ÿ ¶Qÿ

Íý
•ÿ ,ÿ þÙ^ûE x□ü34□ô67þ
ÿ ûeðÌÑÿ ÐØßø/ÿ í•ï´P□•□Ã3Gÿ Cc□à¿ÿ ¶Qÿ

Íý
•ÿ ,ÿ þÙ^ûE x□ü34□ô67þ
ÿ ûeðÌÑÿ ÐØßø/ÿ í•ï´P□•□Ã3Gÿ Cc□à¿ÿ ¶Qÿ

Íý
•ÿ ,ÿ þÙ^ûE x□ü34□ô67þ
ÿ ûeðÌÑÿ ÐØßø/ÿ í•ï´P□•□Ã3Gÿ Cc□â¿ÿ ¶Qÿ

Íý
•ÿ ,ÿ þÙ^ûE x□ü34□ô67þ
ÿ ûeðÌÑÿ ÐØßø/ÿ í•ï´P□•□Ã3Gÿ Cc□à¿ÿ ¶Qÿ

Íý
•ÿ ,ÿ þÙ^ûE x□ü34□ô67þ
ÿ ûeðÌÑÿ ÐØßø/ÿ í•ï´P□•□Ã3Gÿ Cc□â¿ÿ ¶Qÿ

Íý
•ÿ ,ÿ þÙ^ûE x□ü34□ô67þ
ÿ ûeðÌÑÿ ÐØßø/ÿ í•ï´P□•□Ã3Gÿ Cc□à¿ÿ ¶Qÿ

Íý
•ÿ ,ÿ þÙ^ûE x□ü34□ô67þ
ÿ ûeðÌÑÿ ÐØßø/ÿ í•ï´P□•□Ã3Gÿ Cc□â¿ÿ ¶Qÿ

Íý
•ÿ ,ÿ þÙ^ûE x□ü34□ô67þ
ÿ ûeðÌÑÿ ÐØßø/ÿ í•ï´P□•□Ã3Gÿ Cc□â¿ÿ ¶Qÿ

Íý
•ÿ ,ÿ þÙ^ûE x□ü34□ô67þ
ÿ ûeðÌÑÿ ÐØßø/ÿ í•ï´P□•□Ã3Gÿ Cc□à¿ÿ ¶Qÿ

Íý
•ÿ ,ÿ þÙ^ûE x□ü34□ô67þ
ÿ ûeðÌÑÿ ÐØßø/ÿ í•ï´P□•□Ã3Gÿ Cc□â¿ÿ ¶Qÿ

Íý
•ÿ ,ÿ þÙ^ûE x□ü34□ô67þ
ÿ ûeðÌÑÿ ÐØßø/ÿ í•ï´P□•□Ã3Gÿ Cc□â¿ÿ ¶Qÿ

Íý
•ÿ ,ÿ þÙ^ûE x□ü34□ô67þ
ÿ ûeðÌÑÿ ÐØßø/ÿ í•ï´P□•□Ã3Gÿ Cc□à¿ÿ ¶Qÿ

Íý
•ÿ ,ÿ þÙ^ûE x□ü34□ô67þ
ÿ ûeðÌÑÿ ÐØßø/ÿ í•ï´P□•□Ã3Gÿ Cc□â¿ÿ ¶Qÿ

Íý
•ÿ ,ÿ þÙ^ûE x□ü34□ô67þ
ÿ ûeðÌÑÿ ÐØßø/ÿ í•ï´P□•□Ã3Gÿ Cc□â¿ÿ ¶Qÿ

Íý
•ÿ ,ÿ þÙ^ûE x□ü34□ô67þ
ÿ ûeðÌÑÿ ÐØßø/ÿ í•ï´P□•□Ã3Gÿ Cc□à¿ÿ ¶Qÿ

Íý
•ÿ ,ÿ þÙ^ûE x□ü34□ô67þ
ÿ ûeðÌÑÿ ÐØßø/ÿ í•ï´P□•□Ã3Gÿ Cc□â¿ÿ ¶Qÿ

Íý
•ÿ ,ÿ þÙ^ûE x□ü34□ô67þ
ÿ ûeðÌÑÿ ÐØßø/ÿ í•ï´P□•□Ã3Gÿ Cc□â¿ÿ ¶Qÿ

Íý
•ÿ ,ÿ þÙ^ûE x□ü34□ô67þ
ÿ ûeðÌÑÿ ÐØßø/ÿ í•ï´P□•□Ã3Gÿ Cc□à¿ÿ ¶Qÿ

Íý
•ÿ ,ÿ þÙ^ûE x□ü34□ô67þ
ÿ ûeðÌÑÿ ÐØßø/ÿ í•ï´P□•□Ã3Gÿ Cc□à¿ÿ ¶Qÿ

Íý
•ÿ ,ÿ þÙ^ûE x□ü34□ô67þ
ÿ ûeðÌÑÿ ÐØßø/ÿ í•ï´P□•□Ã3Gÿ Cc□à¿ÿ ¶Qÿ

Íý
•ÿ ,ÿ þÙ^ûE x□ü34□ô67þ
ÿ ûeðÌÑÿ ÐØßø/ÿ í•ï´P□•□Ã3Gÿ Cc□à¿ÿ ¶Qÿ

Íý
•ÿ ,ÿ þÙ^ûE x□ü34□ô67þ
ÿ ûeðÌÑÿ ÐØßø/ÿ í•ï´P□•□Ã3Gÿ Cc□â¿ÿ ¶Qÿ

Íý
•ÿ ,ÿ þÙ^ûE x□ü34□ô67þ
ÿ ûeðÌÑÿ ÐØßø/ÿ í•ï´P□•□Ã3Gÿ Cc□à¿ÿ ¶Qÿ

Íý
•ÿ ,ÿ þÙ^ûE x□ü34□ô67þ
ÿ ûeðÌÑÿ ÐØßø/ÿ í•ï´P□•□Ã3Gÿ Cc□à¿ÿ ¶Qÿ

Íý
•ÿ ,ÿ þÙ^ûE x□ü34□ô67þ
ÿ ûeðÌÑÿ ÐØßø/ÿ í•ï´P□•□Ã3Gÿ Cc□â¿ÿ ¶Qÿ

Íý
•ÿ ,ÿ þÙ^ûE x□ü34□ô67þ
ÿ ûeðÌÑÿ ÐØßø/ÿ í•ï´P□•□Ã3Gÿ Cc□à¿ÿ ¶Qÿ

Íý
•ÿ ,ÿ þÙ^ûE x□ü34□ô67þ
ÿ ûeðÌÑÿ ÐØßø/ÿ í•ï´P□•□Ã3Gÿ Cc□â¿ÿ ¶Qÿ

Íý
•ÿ ,ÿ þÙ^ûE x□ü34□ô67þ
ÿ ûeðÌÑÿ ÐØßø/ÿ í•ï´P□•□Ã3Gÿ Cc□â¿ÿ ¶Qÿ

Íý
•ÿ ,ÿ þÙ^ûE x□ü34□ô67þ
ÿ ûeðÌÑÿ ÐØßø/ÿ í•ï´P□•□Ã3Gÿ Cc□à¿ÿ ¶Qÿ

Íý
•ÿ ,ÿ þÙ^ûE x□ü34□ô67þ
ÿ ûeðÌÑÿ ÐØßø/ÿ í•ï´P□•□Ã3Gÿ Cc□à¿ÿ ¶Qÿ

Íý
•ÿ ,ÿ þÙ^ûE x□ü34□ô67þ
ÿ ûeðÌÑÿ ÐØßø/ÿ í•ï´P□•□Ã3Gÿ Cc□â¿ÿ ¶Qÿ

Íý
•ÿ ,ÿ þÙ^ûE x□ü34□ô67þ
ÿ ûeðÌÑÿ ÐØßø/ÿ í•ï´P□•□Ã3Gÿ Cc□â¿ÿ ¶Qÿ

Íý
•ÿ ,ÿ þÙ^ûE x[illegible]ü34[illegible]ô67þ
ÿ ûeðÌÑÿ ÐØßø/ÿ í•ï´P[illegible]•[illegible]Ã3Gÿ Cc[illegible]à¿ÿ ¶Qÿ

Íý
•ÿ ,ÿ þÙ^ûE x□ü34□ô67þ
ÿ ûeðÌÑÿ ÐØßø/ÿ í•ï´P□•□Ã3Gÿ Cc□â¿ÿ ¶Qÿ

Íý
•ÿ ,ÿ þÙ^ûE x□ü34□ô67þ
ÿ ûeðÌÑÿ ÐØßø/ÿ í•ï´P□•□Ã3Gÿ Cc□à¿ÿ ¶Qÿ

Íý
•ÿ ,ÿ þÙ^ûE x□ü34□ô67þ
ÿ ûeðÌÑÿ ÐØßø/ÿ í•ï´P□•□Ã3Gÿ Cc□à¿ÿ ¶Qÿ

Íý
•ÿ ,ÿ þÙ^ûE x□ü34□ô67þ
ÿ ûeðÌÑÿ ÐØßø/ÿ í•ï´P□•□Ã3Gÿ Cc□â¿ÿ ¶Qÿ

Íý
•ÿ ,ÿ þÙ^ûE x□ü34□ô67þ
ÿ ûeðÌÑÿ ÐØßø/ÿ í•ï´P□•□Ã3Gÿ Cc□å¿ÿ ¶Qÿ

Íý
•ÿ ,ÿ þÙ^ûE x□ü34□ô67þ
ÿ ûeðÌÑÿ ÐØßø/ÿ í•ï´P□•□Ã3Gÿ Cc□â¿ÿ ¶Qÿ

Íý
•ÿ ,ÿ þÙ^ûE x□ü34□ô67þ
ÿ ûeðÌÑÿ ÐØßø/ÿ í•ï´P□•□Ã3Gÿ Cc□à¿ÿ ¶Qÿ

Íý
•ÿ ,ÿ þÙ^ûE x□ü34□ô67þ
ÿ ûeðÌÑÿ ÐØßø/ÿ í•ï´P□•□Ã3Gÿ Cc□à¿ÿ ¶Qÿ

Íý
•ÿ ,ÿ þÙ^ûE x□ü34□ô67þ
ÿ ûeðÌÑÿ ÐØßø/ÿ í•ï´P□•□Ã3Gÿ Cc□à¿ÿ ¶Qÿ

Íý
•ÿ ,ÿ þÙ^ûE x[illegible]ü34[illegible]ô67þ
ÿ ûeðÌÑÿ ÐØßø/ÿ í•ï´P[illegible]•[illegible]Ã3Gÿ Cc[illegible]à¿ÿ ¶Qÿ

Íý
•ÿ ,ÿ þÙ^ûE x□ü34□ô67þ
ÿ ûeðÌÑÿ ÐØßø/ÿ í•ï´P□•□Ã3Gÿ Cc□â¿ÿ ¶Qÿ

Íý
•ÿ ,ÿ þÙ^ûE x□ü34□ô67þ
ÿ ûeðÌÑÿ ÐØßø/ÿ í•ï´P□•□Ã3Gÿ Cc□à¿ÿ ¶Qÿ

Íý
•ÿ ,ÿ þÙ^ûE x□ü34□ô67þ
ÿ ûeðÌÑÿ ÐØßø/ÿ í•ï´P□•□Ã3Gÿ Cc□â¿ÿ ¶Qÿ

Íý
•ÿ ,ÿ þÙ^ûE x□ü34□ô67þ
ÿ ûeðÌÑÿ ÐØßø/ÿ í•ï´P□•□Ã3Gÿ Cc□à¿ÿ ¶Qÿ

Íý
•ÿ ,ÿ þÙ^ûE x□ü34□ô67þ
ÿ ûeðÌÑÿ ÐØßø/ÿ í•ï´P□•□Ã3Gÿ Cc□â¿ÿ ¶Qÿ

Íý
•ÿ ,ÿ þÙ^ûE x□ü34□ô67þ
ÿ ûeðÌÑÿ ÐØßø/ÿ í•ï´P□•□Ã3Gÿ Cc□à¿ÿ ¶Qÿ

Íý
•ÿ ,ÿ þÙ^ûE x□ü34□ô67þ
ÿ ûe{?†4Aá¿

éÚ0¸û@³„EæìÙ¿□ñ‟•Îµ¨ □Š(□Š(□Š(□Š(□Š(□Š(□Š(□Š(□Š(□Š(□Š(□Š(□Š
(□Š(□Š(□Š(□Š(□Š(□Š(□Š(□Š(□Š(□Š(□Š(□Š(□Š(□Š(□Š(□Š(□Š(□
Š(□Š(□Š(□Š(□Š(□Š(□Š(□Š(□Š(□Š(□Š(□Š(□Š(□Š(□Š(□Š(□Š(□Š(
□Š(□Š(□Š(□Š(□Š(□Š(□Š(□Š(□Š(□Š(□Š(□Š(□Š(□Š(□Š(□Š(□Š(□Š(
□Š(□Š(□Š(□Š(□Š(□Š(□Š(□Š(□Š(□Š(□Š(□Š(□Š(□Š(□Š(□Š(□Š(□Š
(□Š(□Š(□Š(□Š(□Š(□Š(□Š(□Š(□Š(□Š(□Š(□Š(□Š(□Š(□Š(□Š(□Š(□
Š(□Š(□Š(□Š(□Š(□Š(□Š(□Š(□Š(□Š(□Š(□Š(□Š(□Š(□Š(□Š(□Š(□Š(
□Š(□Š(□Š(□Š(□Š(□Š(□Š(□Š(□Š(□Š(□Š(□Š(□Š(□Š(□Š(□Š(□Š(□Š(
□Š(□Š(□Š(□Š(□Š(□Š(□Š(□Š(□Š(□Š(□Š(□Š(□Š(□Š(□Š(□Š(□Š(□Š
(□Š(□Š(□Š(□Š(□Š(□Š(□Š(□Š(□Š(□Š(□Š(□Š(□Š(□Š(□Š(□Š(□Š(□
Š(□Š(□Š(□Š(□Š(□Š(□Š(□Š(□Š(□Š(□Š(□Š(□Š(□Š(□Š(□Š(□Š(□Š(
□Š(□Š(□Š(□Š(□Š(□Š(□Š(□Š(□Š(□Š(□Š(□Š(□Š(□Š(□Š(□Š(□Š(□Š(
□Š(□Š(□Š(□Š(□Š(□Š(□Š(□Š(□Š(□Š(□Š(□Š(□Š(□Š(□Š(□Š(□Š(□Š
(□Š(□Š(□Š(□Š(□Š(□Š(□Š(□Š(□Š(□Š(□Š(□Š(□Š(□Š(□Š(□Š(□Š(□
Š(□Š(□Š(□Š(□Š(□Š(□Š(□Š(□Š(□Š(□Š(□Š(□Š(□Š(□Š(□Š(□Š(□Š(
□Š(□Š(□Š(□Š(□Š(□Š(□Š(□Š(□Š(□Š(□Š(□Š(□Š(□Š(□Š(□Š(□Š(□Š(
□Š(□Š(□Š(□Š(□Š(□Š(□Š(□Š(□Š(□Š(□Š(□Š(□Š(□Š(□Š(□Š(□Š(□Š
(□Š(□Š(□Š(□Š(□Š(□Š(□Š(□Š(□Š(□Š(□Š(□Š(□Š(□Š(□Š(□Š(□Š(□
Š(□Š(□Š(□Š(□Š(□Š(□Š(□Š(□Š(□Š(□Š(□Š(□Š(□Š(□Š(□Š(□Š(□Š(
□Š(□Š(□Š(□Š(□Š(□Š(□Š(□Š(□Š(□Š(□Š(□Š(□Š(□Š(□Š(□Š(□Š(□Š(
□Š(□Š(□Š(□Š(□Š(□Š(□Š(□Š(□Š(□Š(□Š(□Š(□Š(□Š(□Š(□Š(□Š(□Š
(□Š(□Š(□Š(□Š(□Š(□Š(□Š(□Š(□Š(□Š(□Š(□Š(□Š(□Š(□Š(□Š(□Š(□
Š(□Š(□Š(□Š(□Š(□Š(□Š(□Š(□Š(□Š(□Š(□Š(□Š(□Š(□Š(□Š(□Š(□Š(
□Š(□Š(□Š(□Š(□Š(□Š(□Š(□Š(□Š(□Š(□Š(□Š(□Š(□Š(□Š(□Š(□Š(□Š(
□Š(□Š(□Š(□Š(□Š(□Š(□Š(□Š(□Š(□Š(□Š(□Š(□Š(□Š(□Š(□Š(□Š(□Š
(□Š(□Š(□Š(□Š(□Š(□Š(□Š(□Š(□Š(□Š(□Š(□Š(□Š(□Š(□Š(□Š(□Š(□
Š(□Š(□Š(□Š(□Š(□Š(□Š(□Š(□Š(□Š(□Š(□Š(□Š(□Š(□Š(□Š(□Š(□Š(
□Š(□Š(□Š(□Š(□Š(□Š(□Š(□Š(□Š(□Š(□Š(□Š(□Š(□Š(□Š(□Š(□Š(□Š(
□Š(□Š(□Š(□Š(□Š(□Š(□Š(□Š(□Š(□Š(□Š(□Š(□Š(□Š(□Š(□Š(□Š(□Š
(□Š(□Š(□Š(□Š(□Š(□Š(□Š(□Š(□Š(□Š(□Š(□Š(□Š(□Š(□Š(□Š(□Š(□
Š(□Š(□Š(□Š(□Š(□Š(□Š(□Š(□Š(□Š(□Š(□Š(□Š(□Š(□Š(□Š(□Š(□Š(
□Š(□Š(□Š(□Š(□Š(□Š(□Š(□Š(□Š(□Š(□Š(□Š(□Š(□Š(□Š(□Š(□Š(□Š(
□Š(□Š(□Š(□Š(□Š(□Š(□Š(□Š(□Š(□Š(□Š(□Š(□Š(□Š(□Š(□Š(□Š(□Š
(□Š(□Š(□Š(□Š(□Š(□Š(□Š(□Š(□Š(□Š(□Š(□Š(□Š(□Š(□Š(□Š(□Š(□
Š(□Š(□Š(□Š(□Š(□Š(□Š(□Š(□Š(□Š(□Š(□Š(□Š(□Š(□Š(□Š(□Š(□Š(
□Š(□Š(□Š(□Š(□Š(□Š(□Š(□Š(□Š(□Š(□Š(□Š(□Š(□Š(□Š(□Š(□Š(□Š(
□Š(□Š(□Š(□Š(□Š(□Š(□Š(□Š(□Š(□Š(□Š(□Š(□Š(□Š(□Š(□Š(□Š(□Š
(□Š(□Š(□Š(□Š(□Š(□Š(□Š(□Š(□Š(□Š(□Š(□Š(□Š(□Š(□Š(□Š(□Š(□
Š(□Š(□Š(□Š(□Š(□Š(□Š(□Š(□Š(□Š(□Š(□Š(□Š(□Š(□Š(□Š(□Š(□Š(
□Š(□Š(□Š(□Š(□Š(□Š(□Š(□Š(□Š(□Š(□Š(□Š(□Š(□Š(□Š(□Š(□Š(□Š(
□Š(□Š(□Š(□Š(□Š(□Š(□Š(□Š(□Š(□Š(□Š(□Š(□Š(□Š(□Š(□Š(□Š(□Š
(□Š(□Š(□Š(□Š(□Š(□Š(□Š(□Š(□Š(□Š(□Š(□Š(□Š(□Š(□Š(□Š(□Š(□
Š(□Š(□Š(□Š(□Š(□Š(□Š(□Š(□Š(□Š(□Š(□Š(□Š(□Š(□Š(□Š(□Š(□Š(
□Š(□Š(□Š(□Š(□Š(□Š(□Š(□Š(□Š(□Š(□Š(□Š(□Š(□Š(□Š(□Š(□Š(□Š(
□Š(□Š(□Š(□Š(□Š(□Š(□Š(□Š(□Š(□Š(□Š(□Š(□Š(□Š(□Š(□Š(□Š(□Š
(□Š(□Š(□Š(□Š(□Š(□Š(□Š(□Š(□Š(□Š(□Š(□Š(□Š(□Š(□Š(□Š(□Š(□
Š(□Š(□Š(□Š(□Š(□Š(□Š(□Š(□Š(□Š(□Š(□Š(□Š(□Š(□Š(□Š(□Š(□Š(
□Š(□Š(□Š(□Š(□Š(□Š(□Š(□Š(□Š(□Š(□Š(□Š(□Š(□Š(□Š(□Š(□Š(□Š(
□Š(□Š(□Š(□Š(□Š(□Š(□Š(□Š(□Š(□Š(□Š(□Š(□Š(□Š(□Š(□Š(□Š(□Š
(□Š(□Š(□Š(□Š(□Š(□Š(□Š(□Š(□Š(□Š(□Š(□Š(□Š(□Š(□Š(□Š(□Š(□
Š(□Š(□Š(□Š(□Š(□Š(□Š(□Š(□Š(□Š(□Š(□Š(□Š(□Š(□Š(□Š(□Š(□Š(
□Š(□Š(□Š(□Š(□Š(□Š(□Š(□Š(□Š(□Š(□Š(□Š(□Š(□Š(□Š(□Š(□Š(□Š(
□Š(□Š(□Š(□Š(□Š(□Š(□Š(□Š(□Š(□Š(□Š(□Š(□Š(□Š(□Š(□Š(□Š(□Š
(□Š(□Š(□Š(□Š(□Š(□Š(□Š(□Š(□Š(□Š(□Š(□Š(□Š(□Š(□Š(□Š(□Š(□
Š(□Š(□Š(□Š(□Š(□Š(□Š(□Š(□Š(□Š(□Š(□Š(□Š(□Š(□Š(□Š(□Š(□Š(
□Š(□Š(□Š(□Š(□Š(□Š(□Š(□Š(□Š(□Š(□Š(□Š(□Š(□Š(□Š(□Š(□Š(□Š(
□Š(□Š(□Š(□Š(□Š(□Š(□Š(□Š(□Š(□Š(□Š(□Š(□Š(□Š(□Š(□Š(□Š(□Š
(□Š(□Š(□Š(□Š(□Š(□Š(□Š(□Š(□Š(□Š(□Š(□Š(□Š(□Š(□Š(□Š(□Š(□

Š (□Š (□Š (□Š (□Š (□Š (□Š (□Š (□Š (□Š (□Š (□Š (□Š (□Š (□Š (□Š (□Š (□Š (
□Š (□Š (□Š (□Š (□Š (□Š (□Š (□Š (□Š (□Š (□Š (□Š (□Š (□Š (□Š (□Š (□Š (□Š (
□Š (□Š (□Š (□Š (□Š (□Š (□Š (□Š (□Š (□Š (□Š (□Š (□Š (□Š (□Š (□Š (□Š (□Š
(□Š (□Š (□Š (□Š (□Š (□Š (□Š (□Š (□Š (□Š (□Š (□Š (□Š (□Š (□Š (□Š (□Š (□
Š (□Š (□Š (□Š (□Š (□Š (□Š (□Š (□Š (□Š (□Š (□Š (□Š (□Š (□Š (□Š (□Š (□Š (
□Š (□Š (□Š (□Š (□Š (□Š (□Š (□Š (□Š (□Š (□Š (□Š (□Š (□Š (□Š (□Š (□Š (□Š (
□Š (□Š (□Š (□Š (□Š (□Š (□Š (□Š (□Š (□Š (□Š (□Š (□Š (□Š (□Š (□Š (□Š (□Š
(□Š (□Š (□Š (□Š (□Š (□Š (□Š (□Š (□Š (□Š (□Š (□Š (□Š (□Š (□Š (□Š (□Š (□
Š (□Š (□Š (□Š (□Š (□Š (□Š (□Š (□Š (□Š (□Š (□Š (□Š (□Š (□Š (□Š (□Š (□Š (
□Š (□Š (□Š (□Š (□Š (□Š (□Š (□Š (□Š (□Š (□Š (□Š (□Š (□Š (□Š (□Š (□Š (□Š (
□Š (□Š (□Š (□Š (□Š (□Š (□Š (□Š (□Š (□Š (□Š (□Š (□Š (□Š (□Š (□Š (□Š (□Š
(□Š (□Š (□Š (□Š (□Š (□Š (□Š (□Š (□Š (□Š (□Š (□Š (□Š (□Š (□Š (□Š (□Š (□
Š (□Š (□Š (□Š (□Š (□Š (□Š (□Š (□Š (□Š (□Š (□Š (□Š (□Š (□Š (□Š (□Š (□Š (
□Š (□Š (□Š (□Š (□Š (□Š (□Š (□Š (□Š (□Š (□Š (□Š (□Š (□Š (□Š (□Š (□Š (□Š (
□Š (□Š (□Š (□Š (□Š (□Š (□Š (□Š (□Š (□Š (□Š (□Š (□Š (□Š (□Š (□Š (□Š (□Š
(□Š (□Š (□Š (□Š (□Š (□Š (□Š (□Š (□Š (□Š (□Š (□Š (□Š (□Š (□Š (□Š (□Š (□
Š (□Š (□Š (□Š (□Š (□Š (□Š (□Š (□Š (□Š (□Š (□Š (□Š (□Š (□Š (□Š (□Š (□Š (
□Š (□Š (□Š (□Š (□Š (□Š (□Š (□Š (□Š (□Š (□Š (□Š (□Š (□Š (□Š (□Š (□Š (□Š (
□Š (□Š (□Š (□Š (□Š (□Š (□Š (□Š (□Š (□Š (□Š (□Š (□Š (□Š (□Š (□Š (□Š (□Š
(□Š (□Š (□Š (□Š (□Š (□Š (□Š (□Š (□Š (□Š (□Š (□Š (□Š (□Š (□Š (□Š (□Š (□
Š (□Š (□Š (□Š (□Š (□Š (□Š (□Š (□Š (□Š (□Š (□Š (□Š (□Š (□Š (□Š (□Š (□Š (
□Š (□Š (□Š (□Š (□Š (□Š (□Š (□Š (□Š (□Š (□Š (□Š (□Š (□Š (□Š (□Š (□Š (□Š (
□Š (□Š (□Š (□Š (□Š (□Š (□Š (□Š (□Š (□Š (□Š (□Š (□Š (□Š (□Š (□Š (□Š (□Š
(□Š (□Š (□Š (□Š (□Š (□Š (□Š (□Š (□Š (□Š (□Š (□Š (□Š (□Š (□Š (□Š (□Š (□
Š (□Š (□Š (□Š (□Š (□Š (□Š (□Š (□Š (□Š (□Š (□Š (□Š (□Š (□Š (□Š (□Š (□Š (
□Š (□Š (□Š (□Š (□Š (□Š (□Š (□Š (□Š (□Š (□Š (□Š (□Š (□Š (□Š (□Š (□Š (□Š (
□Š (□Š (□Š (□Š (□Š (□Š (□Š (□Š (□Š (□Š (□Š (□Š (□Š (□Š (□Š (□Š (□Š (□Š
(□Š (□Š (□Š (□Š (□Š (□Š (□Š (□Š (□Š (□Š (□Š (□Š (□Š (□Š (□Š (□Š (□Š (□
Š (□Š (□Š (□Š (□Š (□Š (□Š (□Š (□Š (□Š (□Š (□Š (□Š (□Š (□Š (□Š (□Š (□Š (
□Š (□Š (□Š (□Š (□Š (□Š (□Š (□Š (□Š (□Š (□Š (□Š (□Š (□Š (□Š (□Š (□Š (□Š (
□Š (□Š (□Š (□Š (□Š (□Š (□Š (□Š (□Š (□Š (□Š (□Š (□Š (□Š (□Š (□Š (□Š (□Š
(□Š (□Š (□Š (□Š (□Š (□Š (□Š (□Š (□Š (□Š (□Š (□Š (□Š (□Š (□Š (□Š (□Š (□
Š (□Š (□Š (□Š (□Š (□Š (□Š (□Š (□Š (□Š (□Š (□Š (□Š (□Š (□Š (□Š (□Š (□Š (
□Š (□Š (□Š (□Š (□Š (□Š (□Š (□Š (□Š (□Š (□Š (□Š (□Š (□Š (□Š (□Š (□Š (□Š (
□Š (□Š (□Š (□Š (□Š (□Š (□Š (□Š (□Š (□Š (□Š (□Š (□Š (□Š (□Š (□Š (□Š (□Š
(□Š (□Š (□Š (□Š (□Š (□Š (□Š (□Š (□Š (□Š (□Š (□Š (□Š (□Š (□Š (□Š (□Š (□
Š (□Š (□Š (□Š (□Š (□Š (□Š (□Š (□Š (□Š (□Š (□Š (□Š (□Š (□Š (□Š (□Š (□Š (
□Š (□Š (□Š (□Š (□Š (□Š (□Š (□Š (□Š (□Š (□Š (□Š (□Š (□Š (□Š (□Š (□Š (□Š (
□Š (□Š (□Š (□Š (□Š (□Š (□Š (□Š (□Š (□Š (□Š (□Š (□Š (□Š (□Š (□Š (□Š (□Š
(□Š (□Š (□Š (□Š (□Š (□Š (□Š (□Š (□Š (□Š (□Š (□Š (□Š (□Š (□Š (□Š (□Š (□
Š (□Š (□Š (□Š (□Š (□Š (□Š (□Š (□Š (□Š (□Š (□Š (□Š (□Š (□Š (□Š (□Š (□Š (
□Š (□Š (□Š (□Š (□Š (□Š (□Š (□Š (□Š (□Š (□Š (□Š (□Š (□Š (□Š (□Š (□Š (□Š (
□Š (□Š (□Š (□Š (□Š (□Š (□Š (□Š (□Š (□Š (□Š (□Š (□Š (□Š (□Š (□Š (□Š (□Š
(□Š (□Š (□Š (□Š (□Š (□Š (□Š (□Š (□Š (□Š (□Š (□Š (□Š (□Š (□Š (□Š (□Š (□
Š (□Š (□Š (□Š (□Š (□Š (□Š (□Š (□Š (□Š (□Š (□Š (□Š (□Š (□Š (□Š (□Š (□Š (
□Š (□Š (□Š (□Š (□Š (□Š (□Š (□Š (□Š (□Š (□Š (□Š (□Š (□Š (□Š (□Š (□Š (□Š (
□Š (□Š (□Š (□Š (□Š (□Š (□Š (□Š (□Š (□Š (□Š (□Š (□Š (□Š (□Š (□Š (□Š (□Š
(□Š (□Š (□Š (□Š (□Š (□Š (□Š (□Š (□Š (□Š (□Š (□Š (□Š (□Š (□Š (□Š (□Š (□
Š (□Š (□Š (□Š (□Š (□Š (□Š (□Š (□Š (□Š (□Š (□Š (□Š (□Š (□Š (□Š (□Š (□Š (
□Š (□Š (□Š (□Š (□Š (□Š (□Š (□Š (□Š (□Š (□Š (□Š (□Š (□Š (□Š (□Š (□Š (□Š (
□Š (□Š (□Š (□Š (□Š (□Š (□Š (□Š (□Š (□Š (□Š (□Š (□Š (□Š (□Š (□Š (□Š (□Š
(□Š (□Š (□Š (□Š (□Š (□Š (□Š (□Š (□Š (□Š (□Š (□Š (□Š (□Š (□Š (□Š (□Š (□
Š (□Š (□Š (□Š (□Š (□Š (□Š (□Š (□Š (□Š (□Š (□Š (□Š (□Š (□Š (□Š (□Š (□Š (
□Š (□Š (□Š (□Š (□Š (□Š (□Š (□Š (□Š (□Š (□Š (□Š (□Š (□Š (□Š (□Š (□Š (□Š (
□Š (□Š (□Š (□Š (□Š (□Š (□Š (□Š (□Š (□Š (□Š (□Š (□Š (□Š (□Š (□Š (□Š (□Š
(□Š (□Š (□Š (□Š (□Š (□Š (□Š (□Š (□Š (□Š (□Š (□Š (□Š (□Š (□Š (□Š (□Š (□
Š (□Š (□Š (□Š (□Š (□Š (□Š (□Š (□Š (□Š (□Š (□Š (□Š (□Š (□Š (□Š (□Š (□Š (
□Š (□Š (□Š (□Š (□Š (□Š (□Š (□Š (□Š (□Š (□Š (□Š (□Š (□Š (□Š (□Š (□Š (□Š (

Š (□Š (□Š (□Š (□Š (□Š (□Š (□Š (□Š (□Š (□Š (□Š (□Š (□Š (□Š (□Š (□Š (□Š (
□Š (□Š (□Š (□Š (□Š (□Š (□Š (□Š (□Š (□Š (□Š (□Š (□Š (□Š (□Š (□Š (□Š (□Š (
□Š (□Š (□Š (□Š (□Š (□Š (□Š (□Š (□Š (□Š (□Š (□Š (□Š (□Š (□Š (□Š (□Š (□Š
(□Š (□Š (□Š (□Š (□Š (□Š (□Š (□Š (□Š (□Š (□Š (□Š (□Š (□Š (□Š (□Š (□Š (□
Š (□Š (□Š (□Š (□Š (□Š (□Š (□Š (□Š (□Š (□Š (□Š (□Š (□Š (□Š (□Š (□Š (□Š (
□Š (□Š (□Š (□Š (□Š (□Š (□Š (□Š (□Š (□Š (□Š (□Š (□Š (□Š (□Š (□Š (□Š (□Š (
□Š (□Š (□Š (□Š (□Š (□Š (□Š (□Š (□Š (□Š (□Š (□Š (□Š (□Š (□Š (□Š (□Š (□Š
(□Š (□Š (□Š (□Š (□Š (□Š (□Š (□Š (□Š (□Š (□Š (□Š (□Š (□Š (□Š (□Š (□Š (□
Š (□Š (□Š (□Š (□Š (□Š (□Š (□Š (□Š (□Š (□Š (□ÿÙ
